# Supplementary material for: Tandem dehydrogenation-olefination-decarboxylation of cycloalkyl carboxylic acids via multifold C–H activation
Source: Nat Commun. 2024 Jun 25;15:5370. doi: 10.1038/s41467-024-49359-x (PMC11199700; doi:10.1038/s41467-024-49359-x)
Supplement: Supplementary file 1 — Supplementary Information [file 41467_2024_49359_MOESM1_ESM.pdf]

Supplementary Information

Tandem Dehydrogenation-Olefination-Decarboxylation of Cycloalkyl Carboxylic Acids via Multifold C–H Activation

Tanay Pal[1], Premananda Ghosh[1,2], Minhajul Islam[1,2], Srimanta Guin[1], Suman Maji[1], Suparna Dutta[1], Jayabrata Das[1], Haibo Ge[3]* & Debabrata Maiti[1,2]*

[1]Department of Chemistry, Indian Institute of Technology Bombay, Powai, Mumbai 400076, India

[2]IITB-Monash Research Academy, Indian Institute of Technology Bombay, Mumbai, India.

[3]Department of Chemistry and Biochemistry, Texas Tech University, USA

List of Contents

1. Supplementary Notes 3

 1.1 General Consideration 3

2. Supplementary Methods 3

 2.1 Optimization Details 3-13

 2.2 General procedure for decarboxylative functionalizations 13-15

 2.3 Preparation and characterization data of the starting materials 15-22

 2.4 Characterization data of the products 22-60

 2.5 Applications 60-66

 2.6 Synthesis of probable intermediates for control experiments 66-68

3. Supplementary Discussion 68

 3.1 Detailed mechanistic investigation 68-82

 3.2 Characterization data of side product 83

 3.3 Unsuccessful substrate 83-84

 3.4 Analysis of the crude reaction mixture 85

 3.5 NMR Spectra of the starting materials 86-93

 3.6 NMR spectra of the products 94-199

4. Supplementary References 200-202

1. Supplementary Notes

1.1 General Considerations

Reagent Information. Unless otherwise stated, all reactions were carried out in screw cap reaction tubes. All the solvents were bought from commercial sources and were used without further purification. Palladium salts, cycloalkyl carboxylic acids and olefins were purchased from Johnson Matthey, BLDpharm, Chempure, Zeta Scientific, Aldrich and TCI-India. Silica gel (100–200 mesh) obtained from SRL Co. was used for column chromatography. Products and starting materials were visualized on TLC plate (Merck, TLC silica gel 60 F_{254}) using UV-light or by staining with $KMnO_4$ solution, followed by heating. A gradient elution using petroleum ether and ethyl acetate was performed, based on Merck aluminium TLC sheets (silica gel 60 F_{254}).

Analytical Information. All compounds are characterized by ^{1}H NMR, ^{13}C NMR spectroscopy, and HR-MS. Copies of the ^{1}H NMR, ^{13}C NMR and ^{19}F can be found in the Supporting Information. Unless otherwise stated, all Nuclear Magnetic Resonance spectra were recorded on a Bruker 500 MHz / 400 MHz instrument. All ^{1}H NMR experiments are reported in units, parts per million (ppm), and were measured relative to the signals for residual chloroform (7.26 ppm) in the deuterated solvent, unless otherwise stated. All ^{13}C NMR spectra were reported in ppm relative to deuterochloroform (77.23 ppm), unless otherwise stated, and all were obtained with ^{1}H decoupling. High-resolution mass spectra (HRMS) were recorded on a micro-mass ESI TOF (time of flight) mass spectrometer.

2. Supplementary Methods

2.1 Optimization Details

General procedure for optimization studies: A clean, oven-dried screw cap reaction tube (volume 20 mL) with previously placed magnetic stirbar was charged with 4-^{t}Bu-cyclohexyl carboxylic acid (9.2 mg, 0.05 mmol, 1 *equiv.*), ethyl acrylate (11 µl, 0.1 mmol, 2 *equiv.*), palladium-catalysts, ligands, Ag-salts and alkali salts under air followed by addition of HFIP (0.8 mL). The reaction mixture was vigorously stirred for 24 h in a preheated oil bath at 110 °C. After stipulated time, the reaction mixture was quenched and cooled to room temperature. 1,3,5-trimethoxybenzene was then added to the reaction mixture as the internal standard. Finally, NMR was recorded.

Yields were determined by ^{1}H NMR analysis of the crude reaction mixture using TMB as the internal standard.

Supplementary Table 1: Pd-catalyst optimization

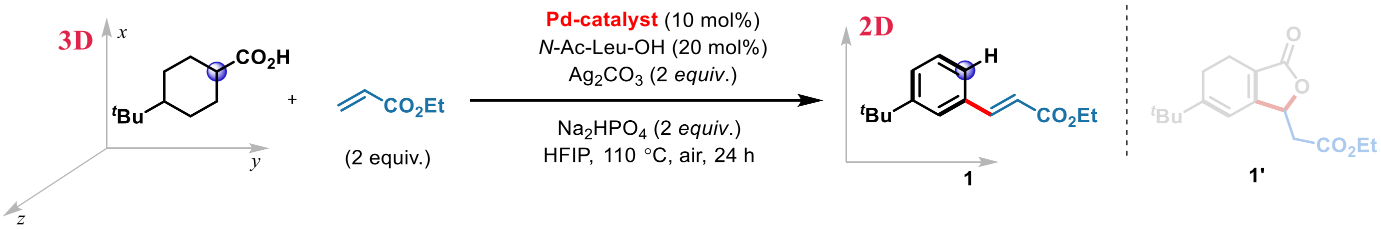

Entry	Catalyst	NMR Yield (1) (%)	Side Product (1') (%)
1	$Pd(OAc)_2$	38	16
2	$Pd(PPh_3)_2Cl_2$	28	12
3	$Pd(PhCN)_2Cl_2$	20	4
4	$[Pd(allyl)Cl]_2$	11	0
**5**	**$[Pd(\pi\text{-cinnamyl})Cl]_2$**	**53**	**0**
6	$Pd(OPiv)_2$	20	16
7	$Pd(COD)Cl_2$	53	12
8	$PdCl_2$	10	trace
9	$Pd(acac)_2$	7	trace
10	$Pd(CH_3CN)_2Cl_2$	31	7
11	$Pd_2(dba)_3$	58	7
12	$Pd(dba)_2$	43	6
13	$Pd(dppf)_2Cl_2$	19	5
14	$Pd(MeCN)_4BF_4$	trace	trace
15	Pd/C	n.r.	n.r.

Supplementary Table 2: Catalyst-amount optimization

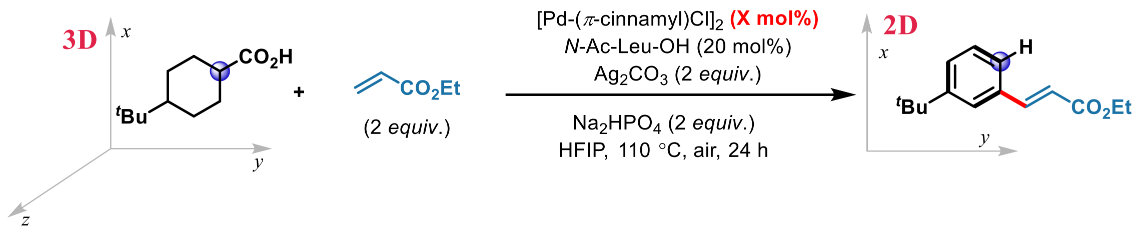

Entry	Catalyst loading (mol%)	NMR Yield (%)
1	1	17
2	2	28
3	5	33
**4**	**10**	**53**

Supplementary Table 3: Ligand optimization

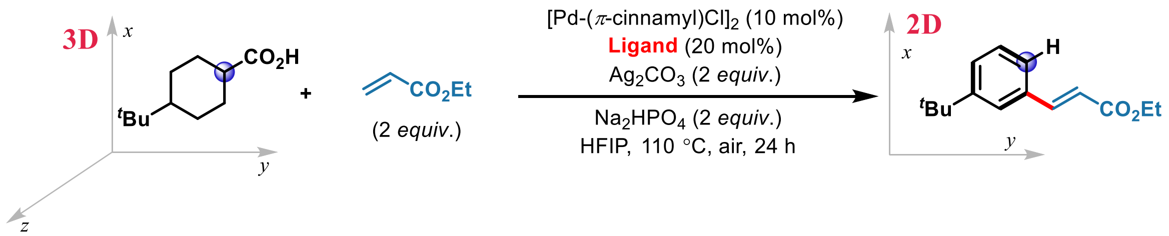

Entry	Ligand	NMR Yield (%)
1	*N*-Ac-Leu-OH	53
2	*N*-Ac-^{t}Leu-OH	39
3	*N*-Ac-Ala-OH	32
4	*N*-Boc-Met-OH	n.r.
**5**	***N*-Ac-Val-OH**	**61**
6	*N*-Ac-Phe-OH	21
7	*N*-Ac-Trp-OH	trace

8	*N*-Ac-Gly-OH	28
9	*N*-Ac-β-Ala-OH	n.r.
10	*N*-Ac-Glu-OH	38
11	*N*-Ac-His-OH	n.r.
12	*N*-Ac-Nle-OH	30
13	*N*-Cbz-Gly-OH	26
14	*N*-Boc-Gly-OH	14
15	*N*-Fmoc-Gly-OH	17
16	*N*-Boc-Tyr-OH	28
17	*N*-Boc-Leu-OH	24
18	*N*-Boc-^{t}Leu-OH	21
19	*N*-Boc-Val-OH	20
20	*N*-Boc-Ile-OH	35
21	*N*-Cbz-Val-OH	29
22	*N*-Boc-Pro-OH	13
22	2-hydroxy-5(trifluoromethyl) pyridine	23
23	2-hydroxy-5 nitro pyridine	17
24	8-nitro quinoline	35
25	2-acetamido-3-methyl-*N*-phenylbutanamide	28
26	2-acetamido-*N*-(4-methoxyphenyl)-3-methylbutanamide	37
27	2-acetamido-*N*-(4-methoxyphenyl)-4-methylpentanamide	27

Supplementary Table 4: Ligand amount optimization

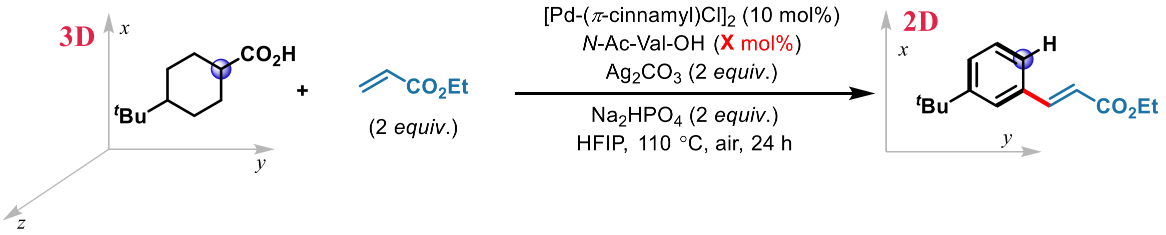

Entry	Ligand amount (mol%)	NMR Yield (%)
1	5	45
2	10	46
3	15	53
**4**	**20**	**61**

Supplementary Table 5: Temperature optimization

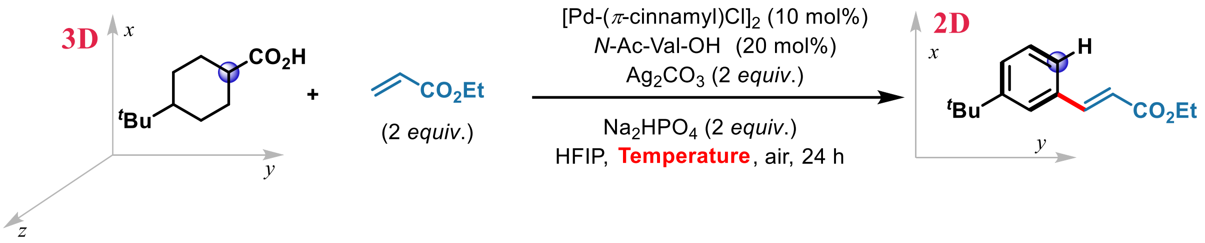

Entry	Temperature (°C)	NMR Yield (%)
1	80	Trace
2	90	45
3	100	56
**4**	**110**	**61**
5	120	50
6	130	48

Supplementary Table 6: Olefin amount optimization

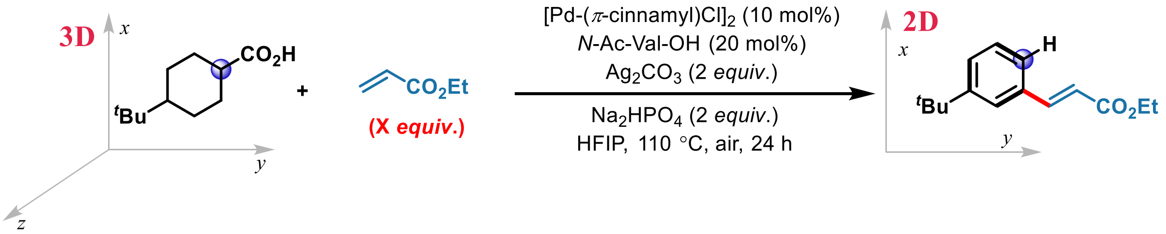

Entry	Olefin amount (*equiv.*)	NMR Yield (%)
1	1.0	34
2	1.5	45
**3**	**2.0**	**61**
4	2.5	36

Supplementary Table 7: Oxidant optimization

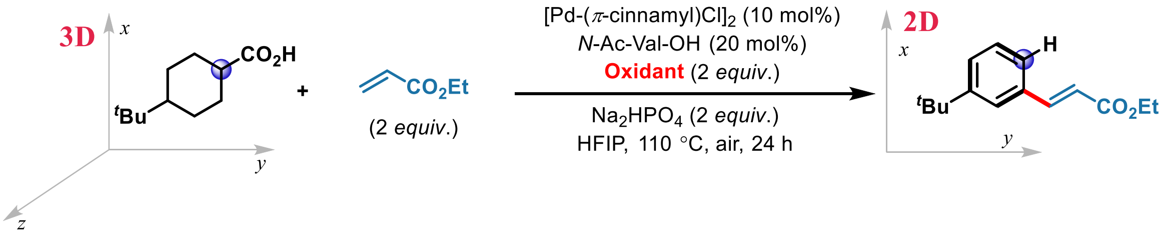

Entry	Oxidant	NMR Yield (%)
1	AgOAc	trace
**2**	**Ag_2CO_3**	**61**
3	Ag_2O	26
4	AgBr	trace
5	AgTFA	n.r.

6	Ag_3PO_4	trace
7	$Cu(OAc)_2$	trace
8	AgF	n.r.
9	AgI	n.r.
10	Ag_2CO_3 + Benzoquinone (2:1)	42
11	Ag_2CO_3 + $K_2S_2O_8$ (2:1)	37
12	Ag_2CO_3 + SeO_2 (2:1)	39
13	$AgSbF_6$	n.r.
14	Ag_2CO_3 under argon atmosphere	trace
15	Ag_2CO_3 under nitrogen atmosphere	28%
16	4.0 *equiv.* Ag_2CO_3 under nitrogen atmosphere	44%

Supplementary Table 8: Oxidant amount optimization

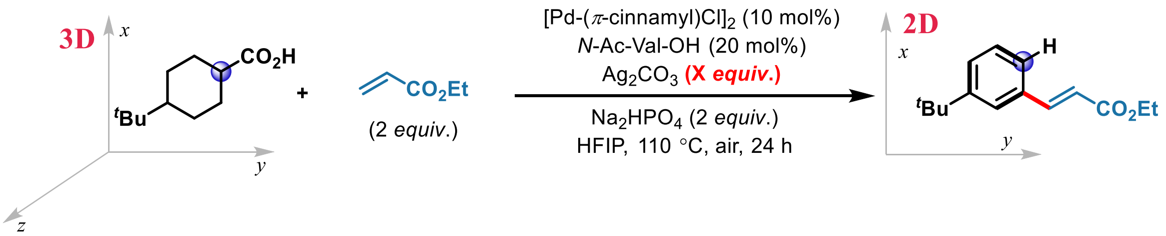

Entry	Oxidant amount (*equiv.*)	NMR Yield (%)
1	without	trace

2	0.25	16
3	0.50	21
4	1.0	25
5	1.5	32
**6**	**2.0**	**61**
7	2.5	62
8	3.0	56
9	4.0	58

From the oxidant optimizations (oxidant amount, with air, with inert atmosphere etc.), it is evident that Ag(I) salt alone is not effective as an oxidant in this particular transformation. Notably, experiments conducted under inert atmosphere revealed that even a super stoichiometric amount of silver carbonate failed to provide similar yield (**Supplementary Table 7**: Entry 14, 15, 16) compared to the reaction using 2 *equiv.* of Ag_2CO_3 under air (**Supplementary Table 7**: Entry 2). Hence, it is reasonable to conclude that, the synergistic action of Ag(I) and aerial oxygen serves as an oxidant system in this reaction. Such observation is also supported by the literature, where dehydrogenation / cyclic ring aromatization was facilitated using Pd-catalyst in combination with O_2 oxidant.[1-2]

Supplementary Table 9: Base optimization

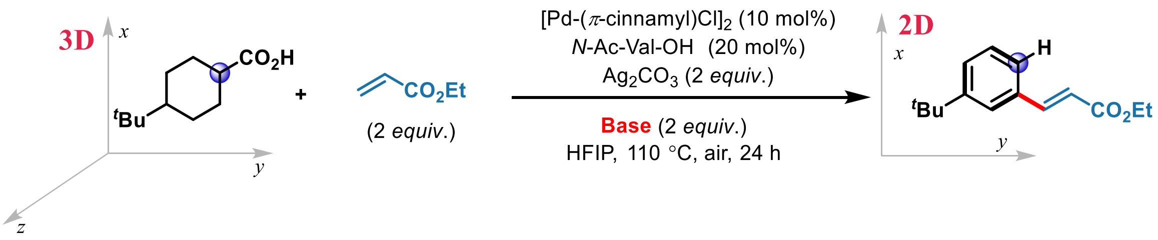

Entry	Base	NMR Yield (%)
1	without	18
2	without base, with 4 *equiv.* Ag_2CO_3	22

3	$NaHSO_4.H_2O$	29
4	K_2CO_3	n.r.
5	K_2HPO_4	59
6	Na_2CO_3	trace
7	NaHCO3	38
8	KH_2PO_4	22
9	$NaH_2PO_2.H_2O$	n.r.
10	Na_3PO_4	44
11	$NaH_2PO_4.H_2O$	33
12	Cs_2CO_3	16
13	$NaOAc.3H_2O$	36
14	NaOAc	n.r.
15	$Na_2HPO_4.7H_2O$	38
16	LiCl	30
17	K_3PO_4	53
18	CsCl	trace
19	$KHCO_3$	22
20	$Li_2SO_4.H_2O$	34
21	Na_2HPO_4 (0.5 *equiv.*)	24
22	Na_2HPO_4 (1.0 *equiv.*)	39
23	Na_2HPO_4 (1.5 *equiv.*)	45
**24**	**Na_2HPO_4 (2 *equiv.*)**	**61**
25	Na_2HPO_4 (2.5 *equiv.*)	55
26	Na_2HPO_4 (3.0 *equiv.*)	53

Supplementary Table 10: Solvent optimization

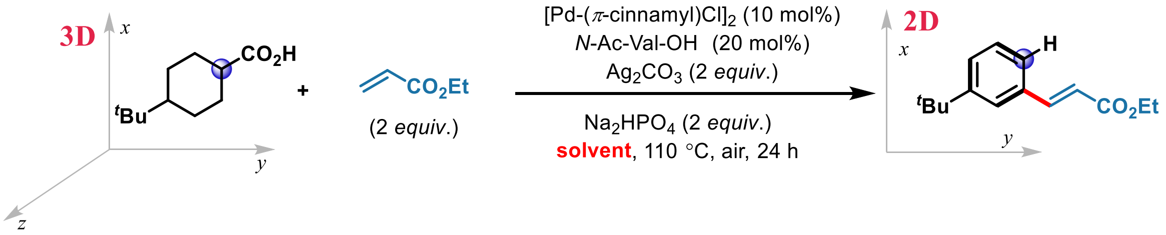

Entry	Solvent	NMR Yield (%)
1	TFE	21
2	DMA	n.r.
3	DCE	5
4	DMF	14
5	1,4-dioxane	trace
6	DCM	9
7	MeCN	8
8	*p*-xylene	n.r*
9	^{t}BuOH	n.r.
10	DMSO	n.r.
11	TFT	trace
**12**	**HFIP**	**61**
13	TAA	n.r.

Olefination of Xylene took place

Based on the solvent optimization results, it is clear that HFIP stands out as the most effective solvent for this reaction. The primary reasons attributing to the exceptional performance of HFIP solvent in this C–H activation reaction are as follows: a) HFIP possesses polar characteristics and is a potent hydrogen bond donor. This unique property enables HFIP to effectively coordinate with the substrate, thereby enhancing the reactivity of the reaction protocols. Furthermore, this enhanced coordination capability aids in stabilizing transition

states. b) HFIP lessens the pH of the media, thereby helping in solubilization of acidic substrates.[3]

Supplementary Table 11: Time optimization

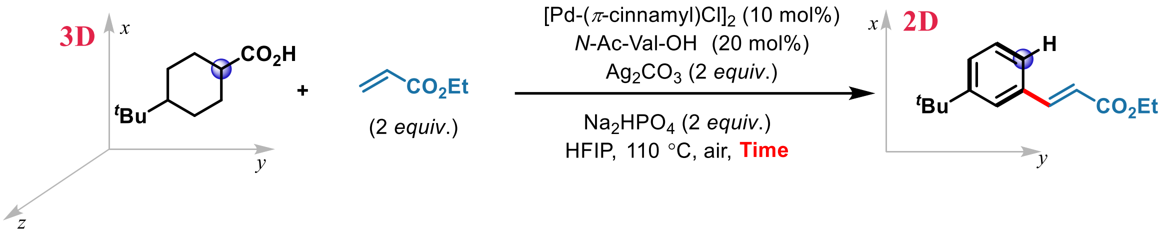

Entry	Time (h)	NMR Yield (%)
1	4	25
2	8	31
3	12	33
4	16	38
5	20	43
6	24	61
7	30	65
**8**	**36**	**69**
9	42	65
10	48	57

2.2 General procedure for decarboxylative functionalizations

a. General procedure for decarboxylative aromatization with olefinic coupling partners (GP1):

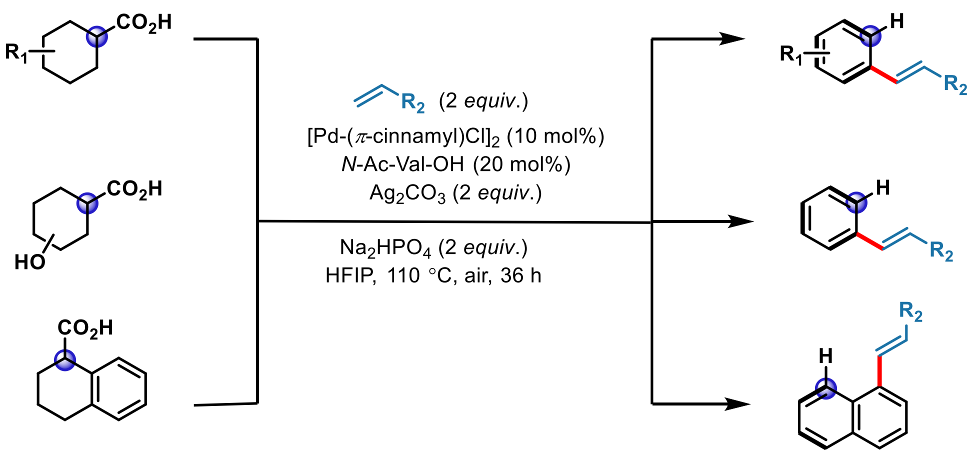

A clean, oven-dried screw cap reaction tube with previously placed magnetic stir–bar was charged with aliphatic cyclohexane carboxylic acid (0.1 mmol, 1 *equiv.*), olefinic partner (0.2 mmol, 2 *equiv.*), [Pd(π-cinnamyl)Cl]$_2$ (0.01 mmol, 10 mol%, 5.1 mg), *N*-Ac-Val-OH (0.02 mmol, 20 mol%, 3.2 mg), silver carbonate (0.2 mmol, 2 *equiv.*, 55 mg) and disodium hydrogen phosphate (0.2 mmol, 2 *equiv.*, 28 mg) under air followed by addition of HFIP (1 mL). The reaction mixture was vigorously stirred for 36 h in a preheated oil bath at 110 °C. After stipulated time, the reaction mixture was cooled to room temperature and filtered through a celite bed using ethyl acetate as the eluent (15 mL). The diluted ethyl acetate solution of the reaction mixture was subsequently washed with saturated brine solution (2 x 10 mL) followed by water (2 x 10 mL). The ethyl acetate layer was dried over anhydrous Na_2SO_4 and the volatiles were removed under vacuum. The crude reaction mixture was purified by column chromatography using silica gel and petroleum-ether /ethyl acetate as the eluent to give the desired olefinated-arene as the product.

b. General procedure for aromatization without olefinic partner (GP2):

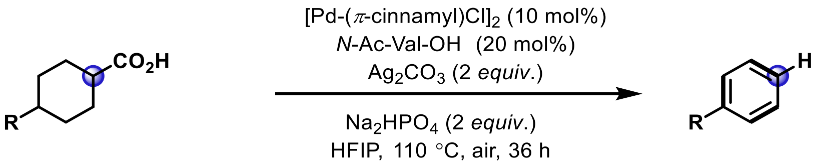

A clean, oven-dried screw cap reaction tube with a previously placed magnetic stir–bar was charged with aliphatic cyclohexane carboxylic acid (0.1 mmol, 1 *equiv.*), [Pd(π-cinnamyl)Cl]$_2$ (0.01 mmol, 10 mol%, 5.1 mg), *N*-Ac-Val-OH (0.02 mmol, 20 mol%, 3.2 mg), silver carbonate (0.2 mmol, 2 *equiv.*, 55 mg) and disodium hydrogen phosphate (0.2 mmol, 2 *equiv.*, 28 mg) under air followed by addition of HFIP (1 mL). The reaction mixture was vigorously stirred for 36 h in a preheated oil bath at 110 °C. After the stipulated time, the reaction mixture was

cooled to room temperature and filtered through a celite bed using ethyl acetate as the eluent (15 mL). The diluted ethyl acetate solution of the reaction mixture was subsequently washed with saturated brine solution (2 x 10 mL) followed by water (2 x 10 mL). The ethyl acetate layer was dried over anhydrous Na_2SO_4 and the volatiles were removed under vacuum. The crude reaction mixture was purified by column chromatography using silica gel and petroleum-ether / ethyl acetate as the eluent to give the desired arenes as the product.

For some cases (entry **64-68**, **70**) yields were determined from crude reaction mixture after the stipulated reaction course, by GC using *n*-decane as internal standard.

c. General procedure for multicomponent difunctionalization reaction (GP3):

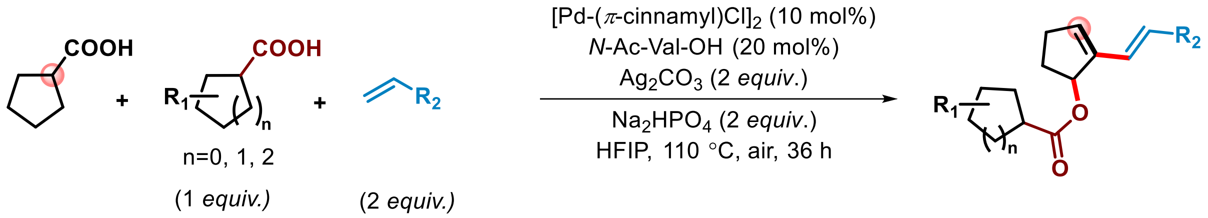

A clean, oven-dried screw cap reaction tube with a previously placed magnetic stir–bar was charged with cyclopentane carboxylic acid (0.1 mmol, 1 *equiv.*), cycloalkyl carboxylic acid (4 to 6 membered cycloalkyl carboxylic acid) (0.1 mmol, 1 *equiv.*), olefinic partner (0.2 mmol, 2 *equiv.*), [Pd(π-cinnamyl)Cl]$_2$ (0.01 mmol, 10 mol%, 5.1 mg), *N*-Ac-Val-OH (0.02 mmol, 20 mol%, 3.2 mg), silver carbonate (0.2 mmol, 2 *equiv.*, 55 mg) and disodium hydrogen phosphate (0.2 mmol, 2 *equiv.*, 28 mg) under air followed by addition of HFIP (1 mL). The reaction mixture was vigorously stirred for 36 h in a preheated oil bath at 110 °C. After stipulated time, the reaction mixture was cooled to room temperature and filtered through a celite bed using ethyl acetate as the eluent (15 mL). The diluted ethyl acetate solution of the reaction mixture was subsequently washed with saturated brine solution (2 x 10 mL) followed by water (2 x 10 mL). The ethyl acetate layer was dried over anhydrous Na_2SO_4 and the volatiles were removed under vacuum. The crude reaction mixture was purified by column chromatography using silica gel and petroleum-ether / ethyl acetate as the eluent to give the desired difunctionalized cyclopentene derivative.

2.3 Preparation and characterization data of the starting materials

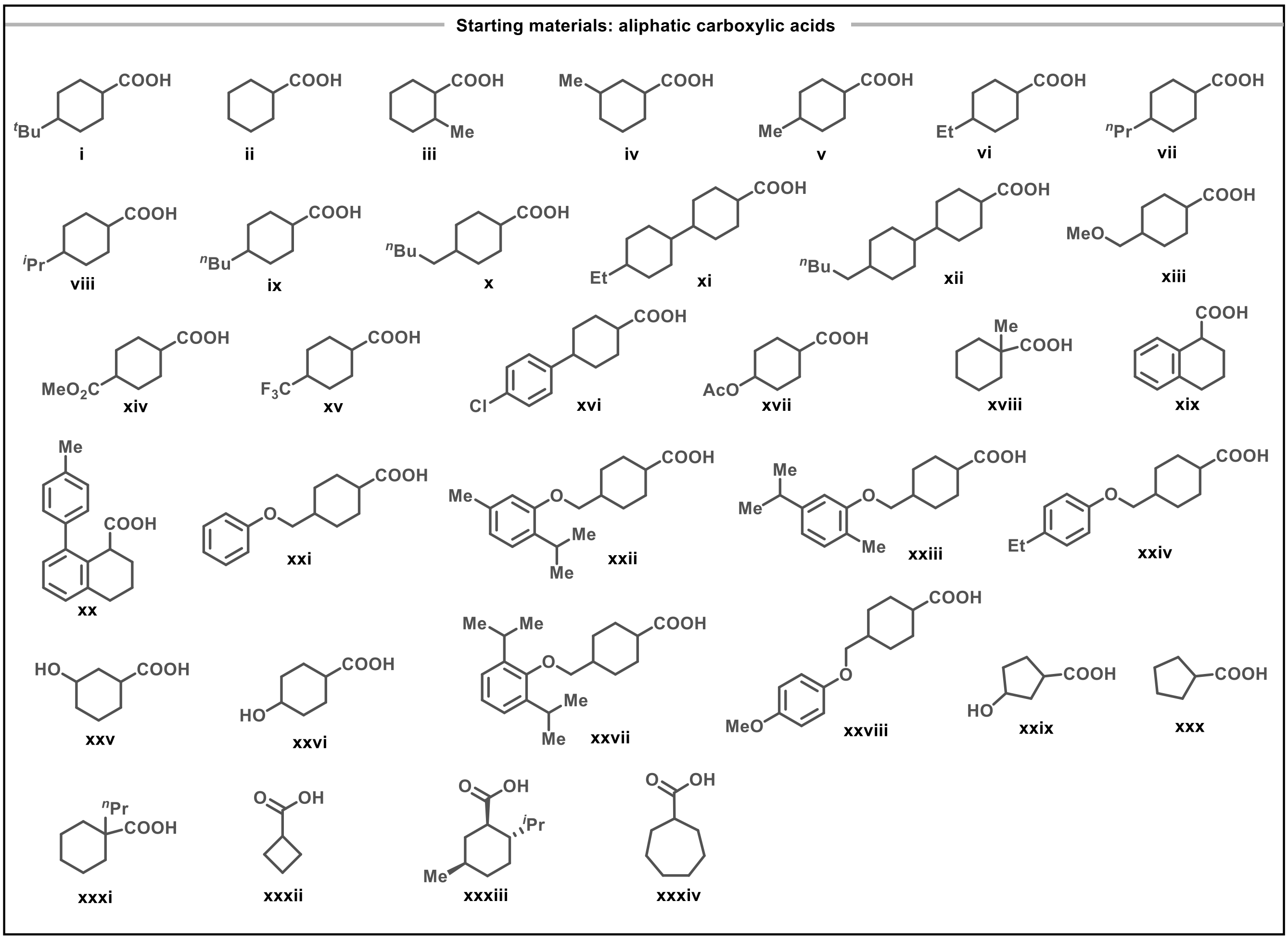

Acid starting materials: **i - xvi**, **xviii**, **xix**, **xxv**, **xxvi**, **xxix** – **xxx**, **xxxii** – **xxxiv** and all the olefinic coupling partners are commercially available and purchased from various sources (as described in **Section 1. General Considerations: Reagent Information**). Acid **xxxi** has been synthesized according to the literature report and reported data is in good agreement with those reported in the literature. Acid **xvii** is prepared by acetylation of hydroxyl group of 4-hydroxy-substituted cyclohexyl carboxylic acid.[4] Acid **xx** has been synthesized via arylation of 1,2,3,4-tetrahydro-1-naphthoic acid.[5] Remaining acids **xxi** – **xxiv**, **xxvii** and **xxviii** have been prepared with minor modifications of a procedure reported in literature.[6] The characterization data of these synthesized starting material acids are provided in the acid preparation section.

Preparatory routes

a. Acetylation of hydroxyl group of 4-hydroxy-substituted cyclohexyl carboxylic acid

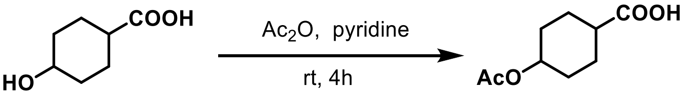

A solution of trans 4-hydroxy carboxylic acid (2.55g, 17.69 mmol) in pyridine (26 mL) was treated with acetic anhydride (12.8 mL, 135 mmol) and was stirred at room temperature for 4 hours. Upon completion all volatiles were removed and the desired product 4-acetoxycyclohexane-1-carboxylic acid was isolated by separation over silica eluting a gradient of ethyl acetate in hexanes containing 1% acetic acid. Yield: 2.56 g (78%).

b. Pd-catalyzed arylation of 1,2,3,4-tetrahydro-1-naphthoic acid

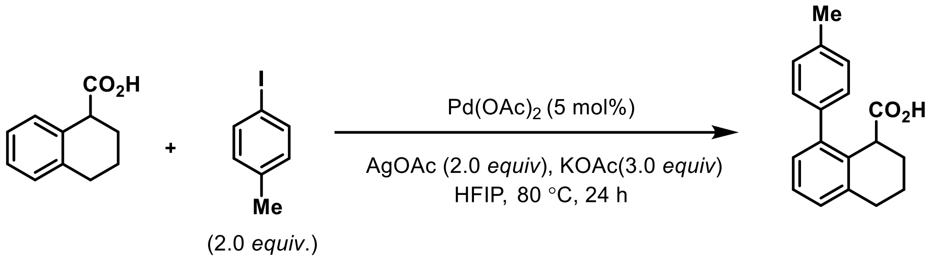

An oven-dried reaction tube was charged with 1,2,3,4-tetrahydronaphthalene-1-carboxylic acid (0.5 mmol, 1.0 *equiv*., 88 mg), 4-iodotoluene (1.0 mmol, 2.0 *equiv*., 218 mg), $Pd(OAc)_2$ (0.025 mmol, 5 mol %, 5.7 mg), AgOAc (1.0 mmol, 2.0 *equiv*., 167 mg), KOAc (1.5 mmol, 3.0 *equiv*., 147 mg), and HFIP (4 mL). The vial was closed and stirred at 80 °C for 24 h. After the stipulated time, the reaction mixture was cooled to room temperature and filtered through a celite bed using ethyl acetate as the eluent (15 mL). The diluted ethyl acetate solution of the reaction mixture was subsequently washed with saturated brine solution (2 x 15 mL) followed by water (2 x 15 mL). The ethyl acetate layer was dried over anhydrous Na_2SO_4, and the volatiles were removed under vacuum. The crude reaction mixture was purified by column chromatography using silica gel and petroleum-ether/ethyl acetate as the eluent to give the desired product in 60% yield.

c. Preparation of 4-aryloxymethyl-substituted cyclohexyl acid substrates

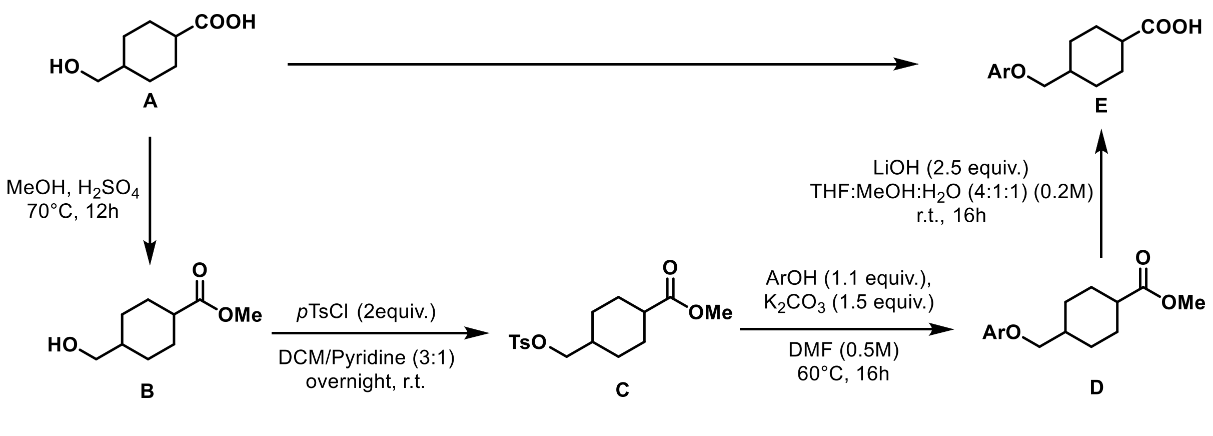

Step 1: Esterification

To a solution of the 4-hydroxymethyl cyclohexane carboxylic acid (1.0 mmol, 158 mg) (A) in MeOH (0.33M) was added conc. H_2SO_4 (4M) and the reaction mixture was stirred at 70 °C for 12 hrs. The reaction mixture was concentrated under reduced pressure and the desired product (B) was directly used in the next step.

Step 2: Tosyl Protection

To an ice-cold solution of B in DCM/pyridine (3:1) was added *p*TsCl (0.5ml). The mixture was stirred at room temperature overnight, cooled to 0 °C, quenched with 2N HCl (35ml) and extracted with DCM thrice. The combined organic layers were dried over Na_2SO_4, filtered and concentrated to afford C in the range of 70-95% yield.

Step 3: Phenol Substitution

To a solution of C dissolved in DMF (0.5M), the corresponding Phenol derivatives (1 *equiv*.) were added, followed by addition of K_2CO_3 (1.5 equiv.) at 60 °C for 16hrs. The reaction mixture was extracted through an ice-cold brine/EtOAc workup (3x) and the organic layers were concentrated under reduced pressure. Flash column chromatography resulted in the isolation of the pure product (D) in the range of 60-80% yield.

Step 4: Hydrolysis

To a solution of methyl ester (D) in THF:MeOH:H_2O (0.2M) was added lithium hydroxide (2.5 *equiv*.). The reaction mixture was stirred for 1 hour at room temperature. The solvent was removed under reduced pressure and the aqueous phase was acidified until *p*H reaches 2 and then extracted with ethyl acetate. The organic layer was washed with brine, dried, filtered and the solvent was removed under reduced pressure to give the corresponding carboxylic acids (E) as white solid or colorless gummy liquid which was used for the next step without further purification.

d. Spectral Data

4-acetoxycyclohexane-1-carboxylic acid (xvii)

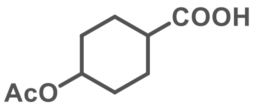

Physical appearance: White solid.

Column material: 100-200 mesh silica.

Eluent: petroleum ether / ethyl acetate (95/5, v/v).

Yield: 78% (2.56 g).

^{1}H NMR (400 MHz, $CDCl_3$) δ 4.76 – 4.60 (m, 1H), 2.38 – 2.25 (m, 1H), 2.18 – 1.95 (m, 7H), 1.67 – 1.47 (m, 2H), 1.47 – 1.31 (m, 2H). **^{13}C NMR** (101 MHz, $CDCl_3$) δ 181.53, 170.84, 72.08, 41.90, 30.61, 26.69, 21.55. **HRMS** (ESI-TOF) m/z: $[M + H]^+$ Calcd. for $C_9H_{15}O_4$ 187.0970; Found 187.0972.

8-(p-tolyl)-1,2,3,4-tetrahydronaphthalene-1-carboxylic acid (xx)

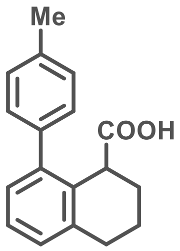

Physical appearance: White solid.

Column material: 100-200 mesh silica.

Eluent: petroleum ether/ ethyl acetate (95/5, v/v).

Yield: 60% (80 mg).

^{1}H NMR (400 MHz, $CDCl_3$) δ 11.18 (s, 1H), 7.54 – 6.86 (m, 7H), 4.06 – 3.76 (m, 1H), 3.13 – 2.81 (m, 2H), 2.47 (s, 3H), 2.34 – 2.20 (m, 1H), 2.09 – 1.79 (m, 3H). **^{13}C NMR** (101 MHz, $CDCl_3$) δ 181.82, 143.17, 138.41, 137.73, 136.81, 131.61, 129.27, 128.96, 128.61, 127.80, 127.00, 42.92, 29.43, 27.31, 21.37, 19.81. **HRMS** (ESI-TOF) m/z: $[M + H]^+$ Calcd. for $C_{18}H_{19}O_2$ 267.1385; Found 267.1386.

4-(phenoxymethyl)cyclohexane-1-carboxylic acid (xxi)

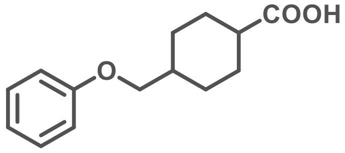

Physical appearance: Colorless gummy liquid.

Column material: 100-200 mesh silica.

Eluent: petroleum ether/ ethyl acetate (95/5, v/v).

Overall yield: 74% (173 mg).

^{1}H NMR (400 MHz, $CDCl_3$) δ 7.35 – 7.19 (m, 2H), 6.98 – 6.82 (m, 3H), 3.81 (d, *J* = 6.8 Hz, 2H), 2.65 (p, *J* = 4.9 Hz, 1H), 2.13 – 2.00 (m, 2H), 2.00 – 1.86 (m, 1H), 1.78 – 1.56 (m, 4H), 1.51 – 1.38 (m, 2H). **^{13}C NMR** (101 MHz, $CDCl_3$) δ 180.48, 159.35, 129.62, 120.75, 114.75,

72.02, 40.17, 36.13, 26.40, 26.10. **HRMS** (ESI-TOF) m/z: $[M + H]^+$ Calcd. for $C_{14}H_{19}O_3$ 235.1334; Found 235.1332.

4-((2-isopropyl-5-methylphenoxy)methyl)cyclohexane-1-carboxylic acid (xxii)

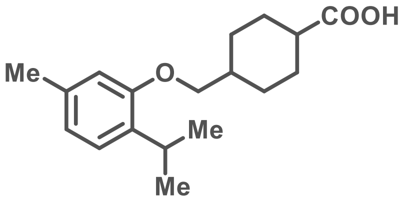

Physical appearance: White solid.

Column material: 100-200 mesh silica.

Eluent: petroleum ether/ ethyl acetate (95/5, v/v).

Overall yield: 48% (140 mg).

^{1}H NMR (500 MHz, $CDCl_3$) δ 7.08 (d, *J* = 7.7 Hz, 1H), 6.72 (d, *J* = 7.6 Hz, 1H), 6.63 (s, 1H), 3.80 (d, *J* = 6.5 Hz, 2H), 3.27 (p, *J* = 6.9 Hz, 1H), 2.68 (t, *J* = 4.8 Hz, 1H), 2.31 (s, 3H), 2.12 – 2.04 (m, 2H), 2.01 – 1.87 (m, 1H), 1.82 – 1.62 (m, 4H), 1.56 – 1.43 (m, 2H), 1.19 (d, *J* = 6.9 Hz, 6H). **^{13}C NMR** (126 MHz, $CDCl_3$) δ 180.98, 156.29, 136.49, 134.20, 125.98, 121.06, 112.22, 71.87, 40.15, 36.35, 26.85, 26.41, 26.17, 22.95, 21.56. **HRMS** (ESI-TOF) m/z: $[M + H]^+$ Calcd. for $C_{18}H_{27}O_3$ 291.1960; Found 291.1964.

4-((5-isopropyl-2-methylphenoxy)methyl)cyclohexane-1-carboxylic acid (xxiii)

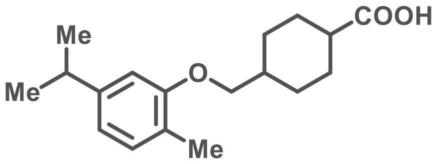

Physical appearance: Colorless gummy liquid.

Column material: 100-200 mesh silica.

Eluent: petroleum ether/ ethyl acetate (95/5, v/v).

Overall yield: 52% (150 mg).

^{1}H NMR (400 MHz, $CDCl_3$) δ 7.04 (d, *J* = 7.6 Hz, 1H), 6.71 (dd, *J* = 7.6, 1.7 Hz, 1H), 6.66 (s, 1H), 3.81 (d, *J* = 6.6 Hz, 2H), 2.85 (p, *J* = 6.9 Hz, 1H), 2.68 (t, *J* = 4.9 Hz, 1H), 2.17 (s, 3H), 2.14 – 1.89 (m, 3H), 1.83 – 1.72 (m, 2H), 1.71 – 1.60 (m, 2H), 1.56 – 1.43 (m, 2H), 1.24 (d, *J* = 6.9 Hz, 6H). **^{13}C NMR** (126 MHz, $CDCl_3$) δ 180.45, 157.29, 148.09, 130.54, 124.33, 118.03, 109.58, 71.90, 40.14, 36.33, 34.38, 26.42, 26.18, 24.37, 16.03. **HRMS** (ESI-TOF) m/z: $[M + H]^+$ Calcd. for $C_{18}H_{27}O_3$ 291.1960; Found 291.1961.

4-((4-ethylphenoxy)methyl)cyclohexane-1-carboxylic acid (xxiv)

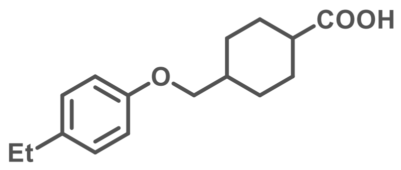

Physical appearance: Colorless gummy liquid.

Column material: 100-200 mesh silica.

Eluent: petroleum ether/ ethyl acetate (95/5, v/v).

Overall yield: 64% (d.r. 4.2:1, 168 mg).

^{1}H NMR (500 MHz, $CDCl_3$) δ 7.09 (d, *J* = 8.6 Hz, 2H), 6.81 (d, *J* = 8.6 Hz, 2H), 3.78 (d, *J* = 6.8 Hz, 2H), 2.66 (p, *J* = 4.8 Hz, 1H), 2.58 (q, *J* = 7.6 Hz, 2H), 2.16 – 2.04 (m, 2H), 1.97 – 1.87 (m, 1H), 1.79 – 1.57 (m, 4H), 1.54 – 1.37 (m, 2H), 1.20 (t, *J* = 7.6 Hz, 3H). **^{13}C NMR** (126 MHz, $CDCl_3$) δ 181.36, 157.38, 136.55, 136.50, 128.90, 128.88, 114.60, 114.52, 73.18, 72.16, 43.22, 40.24, 37.24, 36.14, 29.00, 28.42, 28.18, 26.41, 26.05, 16.13. **HRMS** (ESI-TOF) m/z: $[M + H]^+$ Calcd. for $C_{16}H_{23}O_3$ 263.1647; Found 263.1645.

4-((2,6-diisopropylphenoxy)methyl)cyclohexane-1-carboxylic acid (xxvii)

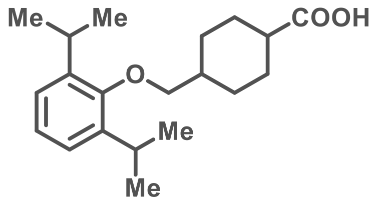

Physical appearance: Colorless gummy liquid.

Column material: 100-200 mesh silica.

Eluent: petroleum ether/ ethyl acetate (95/5, v/v).

Overall yield: 70% (d.r. 3.7:1, 223 mg).

^{1}H NMR (400 MHz, $CDCl_3$) δ 7.08 (s, 3H), 3.57 (d, *J* = 6.4 Hz, 2H), 3.45 – 3.07 (m, 2H), 2.87 – 2.57 (m, 1H), 2.21 – 2.05 (m, 2H), 2.03 – 1.89 (m, 1H), 1.90 – 1.75 (m, 2H), 1.75 – 1.62 (m, 2H), 1.57 – 1.40 (m, 2H), 1.22 (d, J = 6.8 Hz, 12H). **^{13}C NMR** (126 MHz, $CDCl_3$) δ 182.74, 182.15, 153.35, 141.98, 124.60, 124.15, 79.85, 79.05, 43.33, 40.25, 38.26, 37.54, 29.91, 29.01, 28.50, 26.60, 26.54, 26.25, 24.29. **HRMS** (ESI-TOF) m/z: $[M + H]^+$ Calcd. for $C_{20}H_{31}O_3$ 319.2273; Found 319.2274.

4-((4-methoxyphenoxy)methyl)cyclohexane-1-carboxylic acid (xxviii)

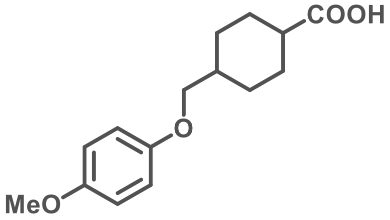

Physical appearance: White solid.

Column material: 100-200 mesh silica.

Eluent: petroleum ether/ ethyl acetate (95/5, v/v).

Overall yield: 86% (d.r. 1.5:1, 227 mg).

^{1}H NMR (400 MHz, $CDCl_3$) δ 6.82 (d, *J* = 1.2 Hz, 4H), 4.00 – 3.62 (m, 5H), 2.65 (p, *J* = 4.9 Hz, 0.4H), 2.39 – 2.26 (m, 0.6H), 2.16 – 1.84 (m, 4H), 1.85 – 1.71 (m, 1H), 1.69 – 1.59 (m, 1H), 1.56 – 1.36 (m, 2H), 1.19 – 1.04 (m, 1H). **^{13}C NMR** (101 MHz, $CDCl_3$) δ 182.08, 181.56, 153.93, 153.91, 153.54, 153.52, 115.69, 115.60, 114.85, 114.83, 73.86, 72.85, 55.97, 43.26, 40.27, 37.29, 36.17, 28.99, 28.64, 28.42, 26.39, 26.05. **HRMS** (ESI-TOF) m/z: $[M + H]^+$ Calcd. for $C_{15}H_{21}O_4$ 265.1440; Found 265.1442.

2.4 Characterization data of arene and cyclopentene derivatives

Ethyl (E)-3-(3-(tert-butyl)phenyl)acrylate (1)

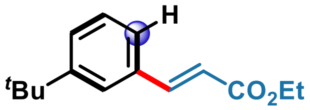

This compound was synthesized by following procedure GP1.

Physical appearance: Colorless gummy liquid.

Column material: 100-200 mesh silica.

Eluent: petroleum ether / ethyl acetate (99/1, v/v).

Yield: 68% (15.8 mg).

^{1}H NMR (400 MHz, $CDCl_3$) δ 7.70 (d, *J* = 16.0 Hz, 1H), 7.54 (t, *J* = 1.9 Hz, 1H), 7.42 (dt, *J* = 7.3, 1.8 Hz, 1H), 7.38 – 7.29 (m, 2H), 6.44 (d, *J* = 16.0 Hz, 1H), 4.27 (q, *J* = 7.1 Hz, 2H), 1.34 (d, *J* = 4.4 Hz, 12H). **^{13}C NMR** (126 MHz, $CDCl_3$) δ 167.36, 152.03, 145.45, 134.37, 128.84, 127.69, 125.48, 125.32, 118.07, 60.69, 34.92, 31.45, 14.55. **HRMS** (ESI-TOF) m/z: $[M + H]^+$ Calcd. for $C_{15}H_{21}O_2$ 233.1542; Found 233.1544.

Ethyl cinnamate (2)[7]

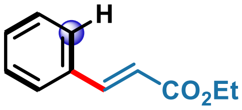

This compound was synthesized by following procedure GP1.

Physical appearance: Colorless gummy liquid.

Column material: 100-200 mesh silica.

Eluent: petroleum ether/ ethyl acetate (98/2, v/v).

Yield: 67% (11.8 mg).

^{1}H NMR (400 MHz, $CDCl_3$) δ 7.69 (d, *J* = 16.0 Hz, 1H), 7.57 – 7.49 (m, 2H), 7.39 (dt, *J* = 4.5, 2.7 Hz, 3H), 6.44 (d, *J* = 16.0 Hz, 1H), 4.27 (q, *J* = 7.1 Hz, 2H), 1.34 (t, *J* = 7.1 Hz, 3H). **^{13}C NMR** (101 MHz, $CDCl_3$) δ 167.23, 144.80, 134.69, 130.43, 129.09, 128.26, 118.51, 60.73, 14.54.

Ethyl (E)-3-(m-tolyl)acrylate (3)

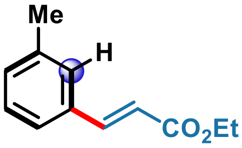

This compound was synthesized by following procedure GP1.

Physical appearance: Colorless gummy liquid.

Column material: 100-200 mesh silica.

Eluent: petroleum ether/ ethyl acetate (98/2, v/v).

Yield: 30% (5.7 mg).

^{1}H NMR (400 MHz, $CDCl_3$) δ 7.66 (d, *J* = 16.0 Hz, 1H), 7.38 – 7.25 (m, 4H), 7.19 (d, *J* = 7.4 Hz, 1H), 6.42 (d, *J* = 16.0 Hz, 1H), 4.26 (q, *J* = 7.1 Hz, 2H), 2.37 (s, 3H), 1.34 (t, *J* = 7.1 Hz, 3H). **^{13}C NMR** (101 MHz, $CDCl_3$) δ 167.32, 144.99, 138.75, 134.63, 131.27, 128.97, 128.94, 125.45, 118.26, 60.68, 21.54, 14.54. **HRMS** (ESI-TOF) m/z: $[M + H]^+$ Calcd. for $C_{12}H_{15}O_2$ 191.1072; Found 191.1076.

Ethyl (E)-3-(p-tolyl)acrylate (4)[7]

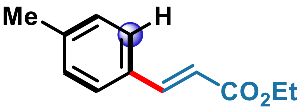

This compound was synthesized by following procedure GP1.

Physical appearance: Colorless gummy liquid.

Column material: 100-200 mesh silica.

Eluent: petroleum ether/ ethyl acetate (98/2, v/v).

Yield: 62% (11.8 mg).

^{1}H NMR (400 MHz, $CDCl_3$) δ 7.66 (d, *J* = 16.0 Hz, 1H), 7.42 (d, *J* = 8.2 Hz, 2H), 7.19 (d, *J* = 7.9 Hz, 2H), 6.39 (d, *J* = 16.0 Hz, 1H), 4.26 (q, *J* = 7.1 Hz, 2H), 2.37 (s, 3H), 1.33 (t, *J* = 7.1 Hz, 3H). **^{13}C NMR** (126 MHz, $CDCl_3$) δ 167.44, 144.81, 140.84, 131.96, 129.82, 128.26, 117.40, 60.63, 21.68, 14.56.

Ethyl (E)-3-(m-tolyl)acrylate

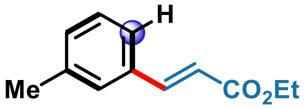

This compound was synthesized by following procedure GP1 (using 4-Me-cyclohexane carboxylic acid)

Yield: 63% (12 mg).

Spectral data: Identical as entry (3)

Ethyl (E)-3-(3-ethylphenyl)acrylate (5)

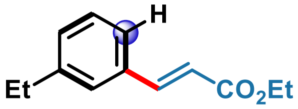

This compound was synthesized by following procedure GP1.

Physical appearance: Colorless gummy liquid.

Column material: 100-200 mesh silica.

Eluent: petroleum ether/ ethyl acetate (98/2, v/v).

Yield: 64% (13.1 mg).

^{1}H NMR (500 MHz, $CDCl_3$) δ 7.68 (d, *J* = 16.0 Hz, 1H), 7.38 – 7.27 (m, 3H), 7.22 (d, *J* = 7.5 Hz, 1H), 6.43 (d, *J* = 16.0 Hz, 1H), 4.26 (q, *J* = 7.1 Hz, 2H), 2.67 (q, *J* = 7.6 Hz, 2H), 1.34 (t, *J* = 7.1 Hz, 3H), 1.30 – 1.20 (m, 3H). **^{13}C NMR** (126 MHz, $CDCl_3$) δ 167.31, 145.11, 145.08, 134.74, 130.15, 129.07, 127.79, 125.70, 118.26, 60.67, 28.94, 15.67, 14.55. **HRMS** (ESI-TOF) m/z: $[M + H]^+$ Calcd. for $C_{13}H_{17}O_2$ 205.1229; Found 205.1236.

Ethyl (E)-3-(3-propylphenyl)acrylate (6)

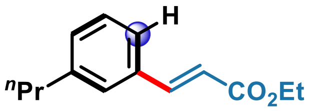

This compound was synthesized by following procedure GP1.

Physical appearance: Colorless gummy liquid.

Column material: 100-200 mesh silica.

Eluent: petroleum ether/ ethyl acetate (98/2, v/v).

Yield: 69% (15.1 mg).

^{1}H NMR (400 MHz, $CDCl_3$) δ 7.67 (d, *J* = 16.0 Hz, 1H), 7.39 – 7.25 (m, 3H), 7.23 – 7.16 (m, 1H), 6.43 (d, *J* = 16.0 Hz, 1H), 4.26 (q, *J* = 7.1 Hz, 2H), 2.64 – 2.56 (m, 2H), 1.70 – 1.60 (m, 2H), 1.34 (t, *J* = 7.1 Hz, 3H), 0.94 (t, *J* = 7.3 Hz, 3H). **^{13}C NMR** (101 MHz, $CDCl_3$) δ 167.32, 145.11, 143.55, 134.61, 130.74, 128.96, 128.41, 125.70, 118.20, 60.67, 38.06, 24.65, 14.55, 13.98. **HRMS** (ESI-TOF) m/z: $[M + Na]^+$ Calcd. for $C_{14}H_{18}NaO_2$ 241.1204; Found 241.1208.

Ethyl (E)-3-(3-isopropylphenyl)acrylate (7)

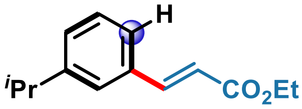

This compound was synthesized by following procedure GP1.

Physical appearance: Colorless gummy liquid.

Column material: 100-200 mesh silica.

Eluent: petroleum ether/ ethyl acetate (98/2, v/v).

Yield: 61% (13.3 mg).

^{1}H NMR (400 MHz, $CDCl_3$) δ 7.68 (d, *J* = 16.0 Hz, 1H), 7.42 – 7.27 (m, 3H), 7.25 (d, *J* = 7.0 Hz, 1H), 6.44 (d, *J* = 16.0 Hz, 1H), 4.27 (q, *J* = 7.1 Hz, 2H), 2.92 (p, *J* = 6.9 Hz, 1H), 1.34 (t, *J* = 7.1 Hz, 3H), 1.26 (d, *J* = 7.0 Hz, 6H). **^{13}C NMR** (101 MHz, $CDCl_3$) δ 167.34, 149.73, 145.19, 134.65, 129.07, 128.79, 126.43, 125.80, 118.15, 60.68, 34.24, 24.10, 14.55. **HRMS** (ESI-TOF) m/z: $[M + Na]^+$ Calcd. for $C_{14}H_{18}NaO_2$ 241.1204; Found 241.1217.

Ethyl (E)-3-(3-butylphenyl)acrylate (8)

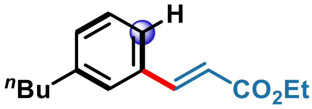

This compound was synthesized by following procedure GP1.

Physical appearance: Colorless gummy liquid.

Column material: 100-200 mesh silica.

Eluent: petroleum ether/ ethyl acetate (98/2, v/v).

Yield: 68% (15.9 mg).

^{1}H NMR (400 MHz, $CDCl_3$) δ 7.67 (d, *J* = 16.0 Hz, 1H), 7.38 – 7.25 (m, 3H), 7.20 (dt, *J* = 7.4, 1.6 Hz, 1H), 6.43 (d, *J* = 16.0 Hz, 1H), 4.26 (q, *J* = 7.1 Hz, 2H), 2.68 – 2.54 (m, 2H), 1.68 –

1.52 (m, 2H), 1.40 – 1.22 (m, 5H), 0.93 (t, *J* = 7.3 Hz, 3H). **^{13}C NMR** (126 MHz, $CDCl_3$) δ 167.33, 145.11, 143.79, 134.62, 130.70, 128.97, 128.34, 125.67, 118.19, 60.67, 35.70, 33.73, 22.53, 14.55, 14.15. **HRMS** (ESI-TOF) m/z: $[M + H]^+$ Calcd. for $C_{15}H_{21}O_2$ 233.1542; Found 233.1546.

Ethyl (E)-3-(3-pentylphenyl)acrylate (9)

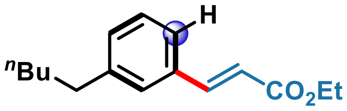

This compound was synthesized by following procedure GP1.

Physical appearance: Colorless gummy liquid.

Column material: 100-200 mesh silica.

Eluent: petroleum ether/ ethyl acetate (98/2, v/v).

Yield: 67% (16.5 mg).

^{1}H NMR (400 MHz, $CDCl_3$) δ 7.67 (d, *J* = 16.0 Hz, 1H), 7.38 – 7.25 (m, 3H), 7.23 – 7.13 (m, 1H), 6.43 (d, *J* = 16.0 Hz, 1H), 4.26 (q, *J* = 7.1 Hz, 2H), 2.61 (dd, *J* = 8.8, 6.7 Hz, 2H), 1.63 (q, *J* = 7.8 Hz, 2H), 1.38 – 1.30 (m, 5H), 1.30 – 1.23 (m, 2H), 0.89 (t, *J* = 6.8 Hz, 3H). **^{13}C NMR** (101 MHz, $CDCl_3$) δ 167.34, 145.12, 143.83, 134.61, 130.70, 128.97, 128.34, 125.66, 118.18, 60.68, 35.99, 31.66, 31.28, 22.74, 14.55, 14.23. **HRMS** (ESI-TOF) m/z: $[M + H]^+$ Calcd. for $C_{16}H_{23}O_2$ 247.1698; Found 247.1705.

Ethyl (E)-3-(3-((1r,4r)-4-ethylcyclohexyl)phenyl)acrylate (10)

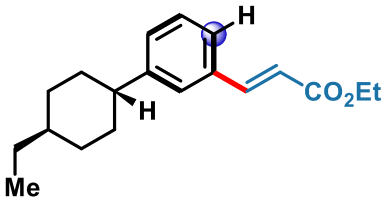

This compound was synthesized by following procedure GP1.

Physical appearance: Colorless gummy liquid.

Column material: 100-200 mesh silica.

Eluent: petroleum ether/ ethyl acetate (98/2, v/v).

Yield: 67% (19.2 mg).

^{1}H NMR (400 MHz, $CDCl_3$) δ 7.68 (d, *J* = 16.0 Hz, 1H), 7.41 – 7.26 (m, 3H), 7.27 – 7.19 (m, 1H), 6.43 (d, *J* = 16.1 Hz, 1H), 4.26 (q, *J* = 7.1 Hz, 2H), 2.48 (tt, *J* = 12.2, 3.3 Hz, 1H), 1.95 – 1.84 (m, 4H), 1.52 – 1.40 (m, 3H), 1.34 (t, *J* = 7.1 Hz, 3H), 1.31 – 1.27 (m, 2H), 1.12 – 0.99 (m, 2H), 0.91 (t, *J* = 7.3 Hz, 3H). **^{13}C NMR** (101 MHz, $CDCl_3$) δ 167.34, 148.77, 145.23,

134.64, 129.21, 129.02, 126.87, 125.83, 118.14, 60.66, 44.73, 39.28, 34.45, 33.32, 30.17, 22.91, 14.55, 14.34, 11.73. **HRMS** (ESI-TOF) m/z: $[M + H]^+$ Calcd. for $C_{19}H_{27}O_2$ 287.2011; Found 287.2056.

Ethyl (E)-3-(3-((1s,4r)-4-pentylcyclohexyl)phenyl)acrylate (11)

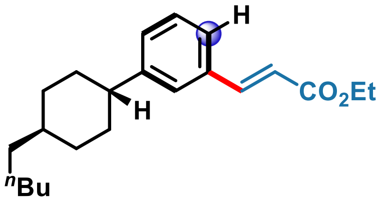

This compound was synthesized by following procedure GP1.

Physical appearance: colorless liquid

Column material: 100-200 mesh silica.

Eluent: petroleum ether/ ethyl acetate (98/2, v/v).

Yield: 44% (14.4 mg).

^{1}H NMR (400 MHz, $CDCl_3$) δ 7.68 (d, *J* = 16.0 Hz, 1H), 7.37 (s, 1H), 7.36 – 7.30 (m, 2H), 7.24 – 7.22 (m, 1H), 6.43 (d, *J* = 16.0 Hz, 1H), 4.26 (q, *J* = 7.2 Hz, 2H), 2.54 – 2.42 (m, 1H), 1.93 – 1.78 (m, 4H), 1.51 – 1.39 (m, 4H), 1.38 – 1.26 (m, 10H), 1.13 – 0.98 (m, 2H), 0.90 (t, *J* = 6.9 Hz, 3H). **^{13}C NMR** (101 MHz, $CDCl_3$) δ 167.35, 148.78, 145.23, 134.61, 129.22, 129.02, 126.86, 125.83, 118.10, 60.67, 44.72, 37.55, 37.48, 34.46, 33.73, 32.42, 30.52, 29.92, 26.86, 22.94, 14.55, 14.35. **HRMS** (ESI-TOF) m/z: $[M + H]^+$ Calcd. for $C_{22}H_{33}O_2$ 329.2481; Found 329.2510.

Ethyl (E)-3-(3-(methoxymethyl)phenyl)acrylate (12)

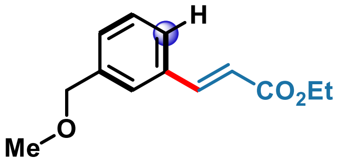

This compound was synthesized by following procedure GP1.

Physical appearance: Colorless gummy liquid.

Column material: 100-200 mesh silica.

Eluent: petroleum ether/ ethyl acetate (96/4, v/v).

Yield: 54% (11.9 mg).

^{1}H NMR (400 MHz, $CDCl_3$) δ 7.69 (d, *J* = 16.0 Hz, 1H), 7.51 (s, 1H), 7.45 (dt, *J* = 7.0, 2.0 Hz, 1H), 7.41 – 7.30 (m, 2H), 6.46 (d, *J* = 16.1 Hz, 1H), 4.47 (s, 2H), 4.26 (q, *J* = 7.1 Hz, 2H), 3.41 (s, 3H), 1.34 (t, *J* = 7.1 Hz, 3H). **^{13}C NMR** (101 MHz, $CDCl_3$) δ 167.20, 144.63, 139.24,

134.88, 129.64, 129.18, 127.61, 127.33, 118.74, 74.49, 60.73, 58.51, 14.54. **HRMS** (ESI-TOF) m/z: $[M + H]^+$ Calcd. for $C_{13}H_{17}O_3$ 221.1178; Found 221.1194.

Methyl (E)-3-(3-ethoxy-3-oxoprop-1-en-1-yl)benzoate (13)

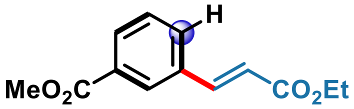

This compound was synthesized by following procedure GP1.

Physical appearance: Colorless gummy liquid.

Column material: 100-200 mesh silica.

Eluent: petroleum ether/ ethyl acetate (98/2, v/v).

Yield: 59% (13.9 mg).

^{1}H NMR (500 MHz, $CDCl_3$) δ 8.18 (d, J = 1.8 Hz, 1H), 8.02 (dt, J = 7.8, 1.4 Hz, 1H), 7.76 – 7.61 (m, 2H), 7.44 (t, J = 7.8 Hz, 1H), 6.49 (d, J = 16.0 Hz, 1H), 4.25 (q, J = 7.1 Hz, 2H), 3.92 (s, 3H), 1.32 (t, J = 7.1 Hz, 3H). **^{13}C NMR** (126 MHz, $CDCl_3$) δ 166.80, 166.62, 143.49, 134.98, 132.34, 131.17, 131.07, 129.18, 129.12, 119.77, 60.81, 52.47, 14.45. **HRMS** (ESI-TOF) m/z: $[M + H]^+$ Calcd. for $C_{13}H_{15}O_4$ 235.097; Found 235.1060.

Ethyl (E)-3-(3-(trifluoromethyl)phenyl)acrylate (14)[8]

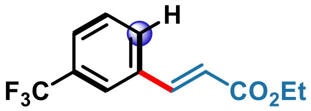

This compound was synthesized by following procedure GP1.

Physical appearance: Colorless gummy liquid.

Column material: 100-200 mesh silica.

Eluent: petroleum ether/ ethyl acetate (97/3, v/v).

Yield: 65% (15.9 mg).

^{1}H NMR (500 MHz, $CDCl_3$) δ 7.77 (s, 1H), 7.74 – 7.67 (m, 2H), 7.63 (d, J = 7.9 Hz, 1H), 7.52 (t, J = 7.8 Hz, 1H), 6.50 (d, J = 16.0 Hz, 1H), 4.28 (q, J = 7.1 Hz, 2H), 1.35 (t, J = 7.1 Hz, 3H). **^{13}C NMR** (126 MHz, $CDCl_3$) δ 166.66, 142.96, 135.49, 131.68 (d, J = 32.3 Hz), 131.26, 129.66, 126.81 (d, J = 3.7 Hz), 124.79 (d, J = 3.9 Hz), 124.05 (d, J =284.7 Hz), 120.50, 60.99, 14.50. **^{19}F NMR** (471 MHz, $CDCl_3$) δ -62.93. **HRMS** (ESI-TOF) m/z: $[M + H]^+$ Calcd. for $C_{12}H_{12}F_3O_2$ 245.0789; Found 245.0795.

Ethyl (E)-3-(4'-chloro-[1,1'-biphenyl]-3-yl)acrylate (15)

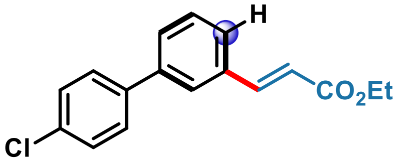

This compound was synthesized by following procedure GP1.

Physical appearance: Colorless gummy liquid.

Column material: 100-200 mesh silica.

Eluent: petroleum ether/ ethyl acetate (98/2, v/v).

Yield: 62% (17.8 mg).

^{1}H NMR (400 MHz, $CDCl_3$) δ 7.74 (d, *J* = 16.0 Hz, 1H), 7.69 (t, *J* = 1.8 Hz, 1H), 7.58 – 7.50 (m, 4H), 7.49 – 7.40 (m, 3H), 6.50 (d, *J* = 16.0 Hz, 1H), 4.28 (q, *J* = 7.1 Hz, 2H), 1.35 (t, *J* = 7.1 Hz, 3H). **^{13}C NMR** (101 MHz, $CDCl_3$) δ 167.10, 144.47, 140.97, 139.11, 135.36, 134.09, 129.67, 129.28, 129.00, 128.60, 127.29, 126.89, 119.13, 60.82, 14.54. **HRMS** (ESI-TOF) m/z: $[M + H]^+$ Calcd. for $C_{17}H_{16}ClO_2$ 287.0839; Found 287.0843.

Ethyl (E)-3-(3-(phenoxymethyl)phenyl)acrylate (16)

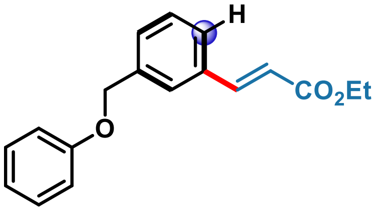

This compound was synthesized by following procedure GP1.

Physical appearance: Colorless gummy liquid.

Column material: 100-200 mesh silica.

Eluent: petroleum ether/ ethyl acetate (98/2, v/v).

Yield: 57% (16.1 mg).

^{1}H NMR (500 MHz, $CDCl_3$) δ 7.70 (d, *J* = 16.0 Hz, 1H), 7.60 (d, *J* = 1.8 Hz, 1H), 7.52 – 7.37 (m, 3H), 7.34 – 7.28 (m, 2H), 7.04 – 6.94 (m, 3H), 6.46 (d, *J* = 16.0 Hz, 1H), 5.08 (s, 2H), 4.27 (q, *J* = 7.1 Hz, 2H), 1.34 (t, *J* = 7.1 Hz, 3H). **^{13}C NMR** (126 MHz, $CDCl_3$) δ 167.14, 158.79, 144.47, 138.14, 135.07, 129.77, 129.37, 127.78, 127.15, 121.37, 118.95, 115.05, 69.68, 60.77, 14.54. **HRMS** (ESI-TOF) m/z: $[M + H]^+$ Calcd. for $C_{18}H_{19}O_3$ 283.1334; Found 283.1336.

Ethyl (E)-3-(3-((4-ethylphenoxy)methyl)phenyl)acrylate (17)

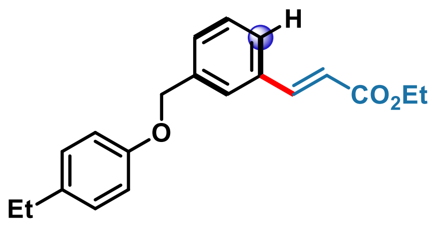

This compound was synthesized by following procedure GP1.

Physical appearance: Colorless gummy liquid.

Column material: 100-200 mesh silica.

Eluent: petroleum ether/ ethyl acetate (97/3, v/v).

Yield: 56% (17.4 mg).

^{1}H NMR (400 MHz, $CDCl_3$) δ 7.70 (d, *J* = 16.0 Hz, 1H), 7.60 (s, 1H), 7.52 – 7.35 (m, 3H), 7.12 (d, *J* = 8.4 Hz, 2H), 7.00 – 6.80 (m, 2H), 6.46 (d, *J* = 16.0 Hz, 1H), 5.06 (s, 2H), 4.27 (q, *J* = 7.1 Hz, 2H), 2.60 (q, *J* = 7.6 Hz, 2H), 1.34 (t, *J* = 7.1 Hz, 3H), 1.21 (t, *J* = 7.6 Hz, 3H). **^{13}C NMR** (101 MHz, $CDCl_3$) δ 167.16, 156.88, 144.52, 138.36, 137.16, 135.04, 129.37, 129.34, 129.02, 127.72, 127.14, 118.90, 114.93, 69.86, 60.76, 28.20, 16.05, 14.54. **HRMS** (ESI-TOF) m/z: $[M + Na]^+$ Calcd. for $C_{20}H_{22}NaO_3$ 333.1467; Found 333.1471.

Ethyl (E)-3-(3-((2-isopropyl-5-methylphenoxy)methyl)phenyl)acrylate (18)

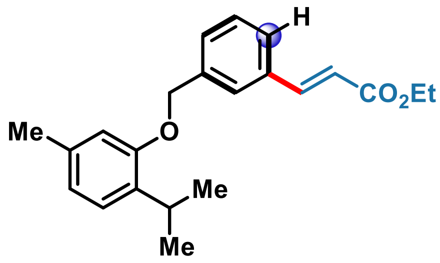

This compound was synthesized by following procedure GP1.

Physical appearance: Colorless gummy liquid.

Column material: 100-200 mesh silica.

Eluent: petroleum ether/ ethyl acetate (97/3, v/v).

Yield: 66% (22.3 mg).

^{1}H NMR (400 MHz, $CDCl_3$) δ 7.72 (d, *J* = 16.0 Hz, 1H), 7.61 (s, 1H), 7.54 – 7.39 (m, 3H), 7.14 (d, *J* = 7.7 Hz, 1H), 6.79 (d, *J* = 7.6 Hz, 1H), 6.74 (s, 1H), 6.46 (d, *J* = 16.0 Hz, 1H), 5.08 (s, 2H), 4.28 (q, *J* = 7.1 Hz, 2H), 3.37 (p, *J* = 6.9 Hz, 1H), 2.33 (s, 3H), 1.35 (t, *J* = 7.1 Hz, 3H), 1.24 (d, *J* = 6.9 Hz, 6H). **^{13}C NMR** (101 MHz, $CDCl_3$) δ 167.19, 155.83, 144.60, 138.69, 136.60, 134.94, 134.57, 129.31, 129.08, 127.46, 126.93, 126.25, 121.87, 118.81, 112.87, 69.75,

60.77, 26.83, 23.03, 21.57, 14.54. **HRMS** (ESI-TOF) m/z: $[M + H]^+$ Calcd. for $C_{22}H_{27}O_3$ 339.1960; Found 339.1964.

Ethyl (E)-3-(3-((5-isopropyl-2-methylphenoxy)methyl)phenyl)acrylate (19)

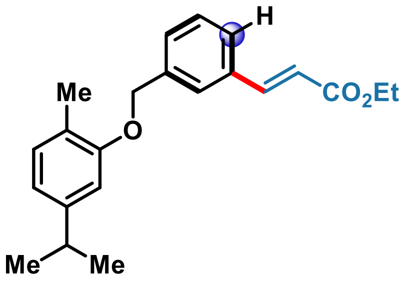

This compound was synthesized by following procedure GP1.

Physical appearance: Colorless gummy liquid.

Column material: 100-200 mesh silica.

Eluent: petroleum ether/ ethyl acetate (97/3, v/v).

Yield: 74% (25.1 mg).

^{1}H NMR (500 MHz, $CDCl_3$) δ 7.71 (d, *J* = 16.0 Hz, 1H), 7.62 (s, 1H), 7.49 (t, *J* = 6.2 Hz, 2H), 7.41 (t, *J* = 7.6 Hz, 1H), 7.10 (d, *J* = 7.4 Hz, 1H), 6.77 (d, *J* = 8.5 Hz, 2H), 6.46 (dd, *J* = 16.0, 0.8 Hz, 1H), 5.09 (s, 2H), 4.28 (q, *J* = 7.3 Hz, 2H), 2.87 (p, *J* = 6.9 Hz, 1H), 2.26 (s, 3H), 1.35 (t, *J* = 7.1 Hz, 3H), 1.24 (d, *J* = 6.9 Hz, 6H). **^{13}C NMR** (126 MHz, $CDCl_3$) δ 167.19, 156.86, 148.16, 144.61, 138.69, 134.94, 130.82, 129.28, 129.18, 127.51, 127.00, 124.61, 118.81, 118.79, 110.21, 69.69, 60.77, 34.32, 24.33, 16.21, 14.54. **HRMS** (ESI-TOF) m/z: $[M + H]^+$ Calcd. for $C_{22}H_{27}O_3$ 339.1960; Found 339.1967.

Ethyl (E)-3-(3-((2,6-diisopropylphenoxy)methyl)phenyl)acrylate (20)

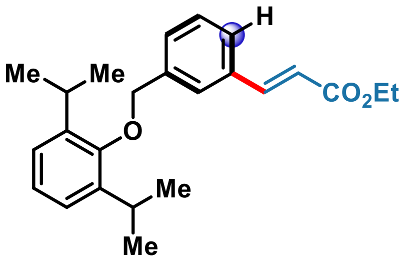

This compound was synthesized by following procedure GP1.

Physical appearance: Colorless gummy liquid.

Column material: 100-200 mesh silica.

Eluent: petroleum ether/ ethyl acetate (97/3, v/v).

Yield: 62% (22.7 mg).

^{1}H NMR (400 MHz, $CDCl_3$) δ 7.74 (d, *J* = 16.0 Hz, 1H), 7.65 (s, 1H), 7.55 – 7.49 (m, 2H), 7.44 (t, *J* = 7.5 Hz, 1H), 7.14 (s, 3H), 6.49 (d, *J* = 16.0 Hz, 1H), 4.82 (s, 2H), 4.28 (q, *J* = 7.1 Hz, 2H), 3.35 (dq, *J* = 13.4, 6.7 Hz, 2H), 1.35 (t, *J* = 7.1 Hz, 3H), 1.25 (d, *J* = 6.9 Hz, 12H). **^{13}C NMR** (126 MHz, $CDCl_3$) δ 167.19, 153.20, 144.61, 142.08, 138.81, 135.04, 129.34, 129.22, 127.70, 126.99, 125.11, 124.35, 118.90, 76.03, 60.78, 26.86, 24.31, 14.55. **HRMS** (ESI-TOF) m/z: $[M + H]^+$ Calcd. for $C_{24}H_{31}O_3$ 367.2273; Found 367.2281.

Ethyl (E)-3-(naphthalen-1-yl)acrylate (21)[9]

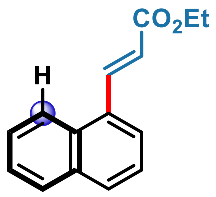

This compound was synthesized by following procedure GP1.

Physical appearance: Colorless gummy liquid.

Column material: 100-200 mesh silica.

Eluent: petroleum ether/ ethyl acetate (99/1, v/v).

Yield: 57% (12.9 mg).

^{1}H NMR (400 MHz, $CDCl_3$) δ 8.53 (d, *J* = 15.8 Hz, 1H), 8.20 (dd, *J* = 8.5, 1.2 Hz, 1H), 7.97 – 7.86 (m, 2H), 7.76 (dt, *J* = 7.2, 0.9 Hz, 1H), 7.61 – 7.45 (m, 3H), 6.53 (d, *J* = 15.8 Hz, 1H), 4.32 (q, *J* = 7.1 Hz, 2H), 1.38 (t, *J* = 7.1 Hz, 3H). **^{13}C NMR** (101 MHz, $CDCl_3$) δ 167.13, 141.87, 133.90, 132.09, 131.65, 130.67, 128.94, 127.07, 126.44, 125.69, 125.23, 123.65, 121.20, 60.84, 14.59. **HRMS** (ESI-TOF) m/z: $[M + Na]^+$ Calcd. for $C_{15}H_{14}NaO_2$ 249.0891; Found 249.0899.

Ethyl (E)-3-(8-(p-tolyl)naphthalen-2-yl)acrylate (22)

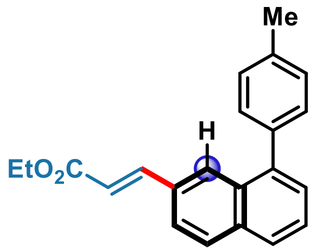

This compound was synthesized by following procedure GP1.

Physical appearance: Colorless gummy liquid.

Column material: 100-200 mesh silica.

Eluent: petroleum ether/ ethyl acetate (98/2, v/v).

Yield: 42% (C7:others 2:1, 13.3 mg).

^{1}H NMR (400 MHz, $CDCl_3$) δ 8.00 (s, 1H), 7.89 (d, *J* = 8.6 Hz, 1H), 7.83 (d, *J* = 8.2 Hz, 1H), 7.74 (d, *J* = 16.0 Hz, 1H), 7.68 (dd, *J* = 8.6, 1.8 Hz, 1H), 7.58 – 7.51 (m, 1H), 7.44 (dd, *J* = 7.1, 1.3 Hz, 1H), 7.38 (d, *J* = 8.1 Hz, 2H), 7.33 (d, *J* = 8.0 Hz, 2H), 6.46 (d, *J* = 16.0 Hz, 1H), 4.26 (q, *J* = 7.1 Hz, 2H), 2.48 (s, 3H), 1.33 (t, *J* = 7.1 Hz, 3H). **^{13}C NMR** (101 MHz, $CDCl_3$) δ 167.29, 145.21, 142.35, 141.27, 137.54, 137.38, 134.81, 132.23, 131.84, 130.10, 129.40, 129.29, 128.79, 127.93, 127.45, 126.99, 123.40, 118.37, 60.72, 21.49, 14.57. **HRMS** (ESI-TOF) m/z: $[M + H]^+$ Calcd. for $C_{22}H_{21}O_2$ 317.1542; Found 317.1537.

Ethyl (E)-3-(o-tolyl)acrylate (23)

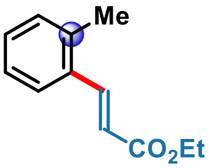

This compound was synthesized by following procedure GP1.

Physical appearance: Colorless gummy liquid.

Column material: 100-200 mesh silica.

Eluent: petroleum ether/ ethyl acetate (98/2, v/v).

Yield: 36% (6.9 mg).

^{1}H NMR (400 MHz, $CDCl_3$) δ 7.98 (d, *J* = 15.9 Hz, 1H), 7.66 – 7.49 (m, 1H), 7.29 – 7.24 (m, 1H), 7.21 (t, *J* = 6.8 Hz, 2H), 6.36 (d, *J* = 15.9 Hz, 1H), 4.27 (q, *J* = 7.1 Hz, 2H), 2.44 (s, 3H), 1.34 (t, *J* = 7.1 Hz, 3H). **^{13}C NMR** (101 MHz, $CDCl_3$) δ 167.30, 142.49, 137.85, 133.64, 130.98, 130.16, 126.60, 126.53, 119.50, 60.71, 20.02, 14.54. **HRMS** (ESI-TOF) m/z: $[M + H]^+$ Calcd. for $C_{12}H_{15}O_2$ 191.1075; Found 191.1074.

Ethyl 2-(3a-methyl-3-oxo-1,3,3a,4,5,6-hexahydroisobenzofuran-1-yl)acetate & ethyl-2-(1-oxo-2-oxaspiro[4.5]decan-3-yl)acetate (inseparable mixture of products) (23')

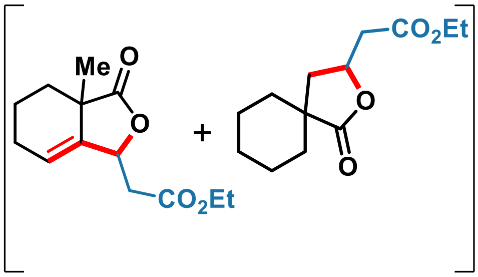

This compound was synthesized by following procedure GP1.

Physical appearance: Colorless gummy liquid.

Column material: 100-200 mesh silica.

Eluent: petroleum ether/ ethyl acetate (98/2, v/v).

Overall yield: 16% (4 mg).

^{1}H NMR (400 MHz, $CDCl_3$) δ 5.59 (td, J = 3.5, 2.0 Hz, 1H), 5.42 (tdd, J = 5.8, 2.9, 1.0 Hz, 1H), 4.80 (dq, J = 9.7, 6.5 Hz, 1H), 4.18 (dq, J = 8.5, 7.2 Hz, 4H), 2.88 – 2.76 (m, 2H), 2.74 – 2.45 (m, 3H), 2.28 – 1.97 (m, 3H), 1.95 – 1.56 (m, 11H), 1.56 – 1.40 (m, 3H), 1.35 (s, 3H), 1.27 (td, J = 7.1, 1.9 Hz, 6H). **^{13}C NMR** (101 MHz, $CDCl_3$) δ 181.06, 179.69, 170.13, 169.88, 139.51, 120.66, 76.66, 73.23, 61.30, 61.19, 45.01, 41.56, 40.71, 39.39, 37.99, 34.43, 31.80, 28.77, 25.44, 24.20, 22.42, 22.33, 22.25, 17.27, 14.34. **HRMS** (ESI-TOF) m/z: $[M + H]^+$ Calcd. for $C_{13}H_{19}O_4$ 239.1283; Found 239.1288 & for $C_{13}H_{21}O_4$ 241.1440; Found 242.1442.

Ethyl (E)-3-(2-propylphenyl)acrylate (24)

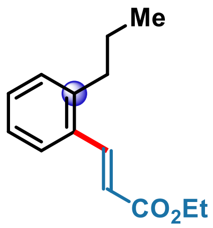

This compound was synthesized by following procedure GP1.

Physical appearance: Colorless gummy liquid.

Column material: 100-200 mesh silica.

Eluent: petroleum ether/ ethyl acetate (98/2, v/v).

Yield: 43% (9.4 mg).

^{1}H NMR (400 MHz, $CDCl_3$) δ 8.01 (d, J = 15.8 Hz, 1H), 7.56 (dd, J = 7.9, 1.5 Hz, 1H), 7.36 – 7.28 (m, 1H), 7.25 – 7.16 (m, 2H), 6.36 (d, J = 15.8 Hz, 1H), 4.27 (q, J = 7.1 Hz, 2H), 2.78 – 2.62 (m, 2H), 1.67 – 1.57 (m, 2H), 1.34 (t, J = 7.1 Hz, 3H), 0.96 (t, J = 7.4 Hz, 3H). **^{13}C NMR** (101 MHz, $CDCl_3$) δ 167.35, 142.54, 142.51, 133.19, 130.32, 130.13, 126.72, 126.55, 119.53, 60.69, 35.52, 24.92, 14.55, 14.14. **HRMS** (ESI-TOF) m/z: $[M + H]^+$ Calcd. for $C_{14}H_{19}O_2$ 219.1385; Found 219.1390.

Ethyl 2-(3-oxo-3a-propyl-1,3,3a,4,5,6-hexahydroisobenzofuran-1-yl)acetate (24')[10]

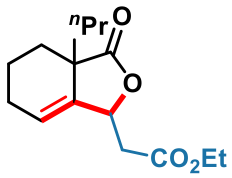

This compound was synthesized by following procedure GP1.

Physical appearance: Colorless gummy liquid.

Column material: 100-200 mesh silica.

Eluent: petroleum ether/ ethyl acetate (98/2, v/v).

Yield: 5% (1.5 mg).

^{1}H NMR (400 MHz, $CDCl_3$) δ 5.65 – 5.55 (m, 1H), 5.46 – 5.36 (m, 1H), 4.23 – 4.15 (m, 2H), 2.82 (dd, *J* = 16.4, 4.6 Hz, 1H), 2.66 (dd, J = 16.3, 7.6 Hz, 1H), 2.21 – 1.98 (m, 3H), 1.75 – 1.64 (m, 4H), 1.47 – 1.39 (m, 3H), 1.31 – 1.24 (m, 3H), 0.91 (t, *J* = 7.2 Hz, 3H). Spectral data of this isolated compound matches with the literature report.[10]

Methyl (E)-3-(3-(tert-butyl)phenyl)acrylate (25)

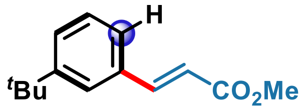

This compound was synthesized by following procedure GP1.

Physical appearance: Colorless gummy liquid.

Column material: 100-200 mesh silica.

Eluent: petroleum ether/ ethyl acetate (98/2, v/v).

Yield: 66% (14.5 mg).

^{1}H NMR (500 MHz, $CDCl_3$) δ 7.72 (d, *J* = 16.0 Hz, 1H), 7.54 (t, *J* = 1.8 Hz, 1H), 7.43 (dt, *J* = 7.6, 1.7 Hz, 1H), 7.38 – 7.31 (m, 2H), 6.45 (d, *J* = 16.0 Hz, 1H), 3.81 (s, 3H), 1.34 (s, 9H). **^{13}C NMR** (126 MHz, $CDCl_3$) δ 167.74, 152.02, 145.71, 134.26, 128.84, 127.75, 125.52, 125.27, 117.58, 51.87, 34.89, 31.43. **HRMS** (ESI-TOF) m/z: $[M + H]^+$ Calcd. for $C_{14}H_{19}O_2$ 219.1385; Found 219.1390.

Butyl (E)-3-(3-(tert-butyl)phenyl)acrylate (26)

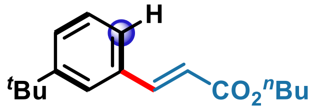

This compound was synthesized by following procedure GP1.

Physical appearance: Colorless gummy liquid.

Column material: 100-200 mesh silica.

Eluent: petroleum ether/ ethyl acetate (98/2, v/v).

Yield: 76% (19.8 mg).

^{1}H NMR (400 MHz, $CDCl_3$) δ 7.70 (d, *J* = 16.0 Hz, 1H), 7.54 (t, *J* = 1.9 Hz, 1H), 7.42 (dt, *J* = 7.3, 1.8 Hz, 1H), 7.39 – 7.28 (m, 2H), 6.44 (d, *J* = 16.0 Hz, 1H), 4.22 (t, *J* = 6.7 Hz, 2H), 1.78 – 1.63 (m, 2H), 1.49 – 1.40 (m, 2H), 1.34 (s, 9H), 0.97 (t, *J* = 7.4 Hz, 3H). **^{13}C NMR** (101 MHz, $CDCl_3$) δ 167.45, 152.03, 145.41, 134.39, 128.84, 127.68, 125.46, 125.34, 118.10, 64.62,

34.93, 31.47, 31.02, 19.43, 13.98. **HRMS** (ESI-TOF) m/z: $[M + H]^+$ Calcd. for $C_{17}H_{25}O_2$ 261.1855; Found 261.1867.

Cyclohexyl (E)-3-(3-(tert-butyl)phenyl)acrylate (27)

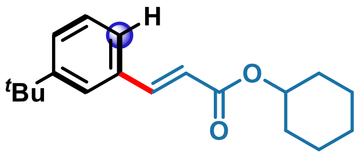

This compound was synthesized by following procedure GP1.

Physical appearance: Colorless gummy liquid.

Column material: 100-200 mesh silica.

Eluent: petroleum ether/ ethyl acetate (98/2, v/v).

Yield: 62% (17.8 mg).

^{1}**H NMR** (500 MHz, $CDCl_3$) δ 7.68 (d, *J* = 16.0 Hz, 1H), 7.54 (d, *J* = 1.9 Hz, 1H), 7.41 (dt, *J* = 7.5, 1.7 Hz, 1H), 7.38 – 7.28 (m, 2H), 6.43 (d, *J* = 16.0 Hz, 1H), 4.89 (dt, *J* = 9.3, 5.0 Hz, 1H), 1.93 (dd, *J* = 11.8, 5.6 Hz, 2H), 1.77 (ddd, *J* = 12.9, 8.9, 4.4 Hz, 2H), 1.48 (dd, *J* = 12.9, 9.7 Hz, 2H), 1.45 – 1.38 (m, 2H), 1.33 (s, 9H), 1.30 – 1.24 (m, 2H). ^{13}**C NMR** (126 MHz, $CDCl_3$) δ 166.80, 151.99, 145.12, 134.46, 128.81, 127.57, 125.41, 125.35, 118.69, 72.95, 34.93, 32.00, 31.47, 25.67, 24.07. **HRMS** (ESI-TOF) m/z: $[M + H]^+$ Calcd. for $C_{19}H_{27}O_2$ 287.2011; Found 287.2045.

Benzyl cinnamate (28)

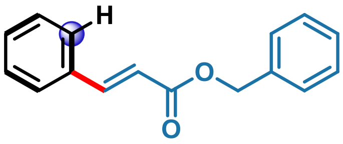

This compound was synthesized by following procedure GP1.

Physical appearance: Colorless gummy liquid.

Column material: 100-200 mesh silica.

Eluent: petroleum ether/ ethyl acetate (98/2, v/v).

Yield: 61% (14.5 mg).

^{1}**H NMR** (400 MHz, $CDCl_3$) δ 7.76 (d, *J* = 16.0 Hz, 1H), 7.58 – 7.50 (m, 2H), 7.46 – 7.33 (m, 8H), 6.51 (d, *J* = 16.0 Hz, 1H), 5.28 (s, 2H). ^{13}**C NMR** (101 MHz, $CDCl_3$) δ 166.97, 145.36, 136.22, 134.50, 130.53, 129.06, 128.78, 128.46, 128.44, 128.28, 118.02, 66.54. **HRMS** (ESI-TOF) m/z: $[M + H]^+$ Calcd. for $C_{16}H_{15}O_2$ 239.1072; Found 239.1077.

Benzyl (E)-3-(naphthalen-1-yl)acrylate (29)

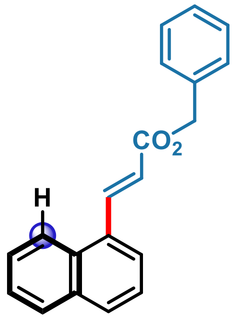

This compound was synthesized by following procedure GP1.

Physical appearance: Colorless liquid.

Column material: 100-200 mesh silica.

Eluent: petroleum ether

Yield: 40% (11.5 mg).

^{1}H NMR (400 MHz, $CDCl_3$) δ 8.58 (d, J = 15.8 Hz, 1H), 8.24 – 8.15 (m, 1H), 7.98 – 7.86 (m, 2H), 7.80 – 7.72 (m, 1H), 7.62 – 7.51 (m, 2H), 7.49 – 7.32 (m, 6H), 6.59 (d, J = 15.7 Hz, 1H), 5.31 (s, 2H). **^{13}C NMR** (101 MHz, $CDCl_3$) δ 166.93, 142.46, 136.30, 133.90, 131.94, 131.63, 130.82, 128.95, 128.85, 128.54, 128.51, 127.12, 126.46, 125.68, 125.31, 123.61, 120.73, 66.68. **HRMS** (ESI-TOF) m/z: $[M + H]^+$ Calcd. for $C_{20}H_{17}O_2$ 289.1229; Found 289.1234.

4-chlorobenzyl (E)-3-(3-(tert-butyl)phenyl)acrylate (30)

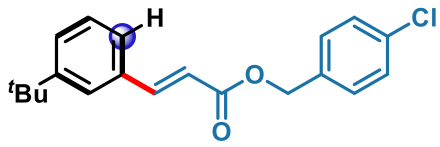

This compound was synthesized by following procedure GP1.

Physical appearance: Colorless gummy liquid.

Column material: 100-200 mesh silica.

Eluent: petroleum ether/ ethyl acetate (97/3, v/v).

Yield: 61% (20.1 mg).

^{1}H NMR (400 MHz, $CDCl_3$) δ 7.74 (d, J = 16.0 Hz, 1H), 7.53 (t, J = 1.8 Hz, 1H), 7.48 – 7.40 (m, 1H), 7.39 – 7.29 (m, 6H), 6.48 (d, J = 16.0 Hz, 1H), 5.22 (s, 2H), 1.33 (s, 9H). **^{13}C NMR** (101 MHz, $CDCl_3$) δ 166.99, 152.10, 146.30, 134.85, 134.37, 134.18, 129.88, 129.01, 128.89, 127.93, 125.56, 125.41, 117.42, 65.69, 34.93, 31.45. **HRMS** (ESI-TOF) m/z: $[M + H]^+$ Calcd. for $C_{20}H_{22}ClO_2$ 329.1308; Found 329.1313.

2,2,3,3,4,4,5,5,6,6,7,7-dodecafluoroheptyl (E)-3-(3-(tert-butyl)phenyl)acrylate (31)

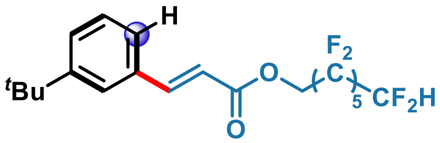

This compound was synthesized by following procedure GP1.

Physical appearance: Colorless gummy liquid.

Column material: 100-200 mesh silica.

Eluent: petroleum ether/ ethyl acetate (98/2, v/v).

Yield: 69% (35.8 mg).

^{1}H NMR (400 MHz, $CDCl_3$) δ 7.81 (dd, *J* = 16.0, 5.6 Hz, 1H), 7.56 (dt, *J* = 4.1, 2.0 Hz, 1H), 7.47 (dtd, *J* = 6.0, 3.3, 1.7 Hz, 1H), 7.42 – 7.27 (m, 2H), 6.49 (dd, *J* = 16.0, 5.7 Hz, 1H), 6.28 – 5.80 (m, 1H), 4.95 – 4.46 (m, 2H), 1.61 – 1.13 (m, 9H).**13C NMR** (101 MHz, $CDCl_3$) δ 165.54, 152.23, 148.15, 133.77, 128.98, 128.45, 125.80, 125.64, 115.61, 107.78, 60.07, 59.80, 59.53, 34.96, 31.44. **^{19}F NMR** (376 MHz, $CDCl_3$) δ -119.45 (d, *J* = 14.6 Hz), -122.15 (d, *J* = 14.0 Hz), -123.39, -129.45 (d, *J* = 13.9 Hz), -137.00 (d, *J* = 51.7 Hz). **HRMS** (ESI-TOF) m/z: $[M + H]^+$ Calcd. for $C_{20}H_{19}F_{12}O_2$ 519.1193; Found 519.1224.

Methyl-5-phenylcyclopent-1-ene-1-carboxylate (32)

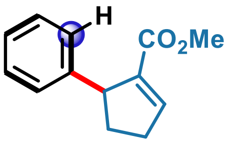

This compound was synthesized by following procedure GP1.

Physical appearance: Colorless gummy liquid.

Column material: 100-200 mesh silica.

Eluent: petroleum ether/ ethyl acetate (98/2, v/v).

Yield: 54% (10.9 mg).

^{1}H NMR (400 MHz, $CDCl_3$) δ 7.34 – 7.24 (m, 2H), 7.23 – 7.14 (m, 3H), 7.00 (q, *J* = 2.2 Hz, 1H), 4.15 (dt, *J* = 6.6, 1.5 Hz, 1H), 3.61 (s, 3H), 2.76 – 2.62 (m, 1H), 2.59 – 2.46 (m, 2H), 2.11 – 1.79 (m, 1H). **^{13}C NMR** (101 MHz, $CDCl_3$) δ 165.40, 145.30, 145.08, 139.39, 128.60, 127.16, 126.39, 51.51, 50.27, 34.28, 32.41. **HRMS** (ESI-TOF) m/z: $[M + H]^+$ Calcd. for $C_{13}H_{15}O_2$ 203.1072; Found 203.1077.

Methyl-1,4,5,6-tetrahydro-[1,1'-biphenyl]-2-carboxylate (33)

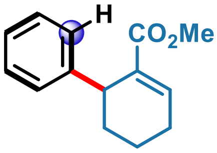

This compound was synthesized by following procedure GP1.

Physical appearance: Colorless gummy liquid.

Column material: 100-200 mesh silica.

Eluent: petroleum ether/ ethyl acetate (98/2, v/v).

Yield: 28% (6.1 mg).

^{1}H NMR (400 MHz, $CDCl_3$) δ 7.31 – 7.23 (m, 3H), 7.22 – 7.07 (m, 3H), 3.93 (s, 1H), 3.59 (s, 3H), 2.57 – 2.17 (m, 2H), 1.92 (ddt, J = 12.7, 8.7, 6.0 Hz, 1H), 1.77 (dq, J = 12.9, 4.0 Hz, 1H), 1.52 (dq, J = 10.4, 5.5, 5.1 Hz, 2H). **^{13}C NMR** (101 MHz, $CDCl_3$) δ 167.79, 145.19, 141.84, 132.08, 128.35, 127.94, 126.15, 51.71, 39.73, 31.58, 26.10, 17.14. **HRMS** (ESI-TOF) m/z: $[M + H]^+$ Calcd. for $C_{14}H_{17}O_2$ 217.1229; Found 217.1231.

(E)-4-(3-(tert-butyl)phenyl)but-3-en-2-one (34)

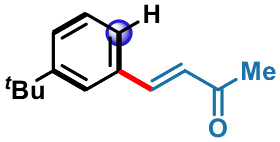

This compound was synthesized by following procedure GP1.

Physical appearance: Colorless gummy liquid.

Column material: 100-200 mesh silica.

Eluent: petroleum ether/ ethyl acetate (92/8, v/v).

Yield: 73% (14.8 mg).

^{1}H NMR (400 MHz, $CDCl_3$) δ 7.61 – 7.50 (m, 2H), 7.48 – 7.42 (m, 1H), 7.41 – 7.30 (m, 2H), 6.72 (d, J = 16.3 Hz, 1H), 2.39 (s, 3H), 1.34 (s, 9H). **^{13}C NMR** (101 MHz, $CDCl_3$) δ 198.77, 152.15, 144.38, 134.32, 128.94, 128.01, 127.17, 125.70, 125.48, 34.93, 31.44, 27.58. **HRMS** (ESI-TOF) m/z: $[M + H]^+$ Calcd. for $C_{14}H_{19}O$ 203.1436; Found 203.1469.

(E)-3-(3-(tert-butyl)phenyl)acrylonitrile (35)

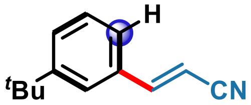

This compound was synthesized by following procedure GP1.

Physical appearance: Colorless gummy liquid.

Column material: 100-200 mesh silica.

Eluent: petroleum ether/ ethyl acetate (98/2, v/v).

Yield: 55% (10.2 mg).

^{1}H NMR (500 MHz, $CDCl_3$) δ 7.47 (d, *J* = 7.8 Hz, 1H), 7.45 – 7.39 (m, 2H), 7.34 (t, *J* = 7.7 Hz, 1H), 7.28 (d, *J* = 7.8 Hz, 1H), 5.88 (d, *J* = 16.6 Hz, 1H), 1.33 (s, 9H). **^{13}C NMR** (126 MHz, $CDCl_3$) δ 152.43, 151.42, 133.52, 129.10, 128.71, 124.86, 124.47, 118.52, 96.13, 31.42, 27.39. **HRMS** (ESI-TOF) m/z: $[M + H]^+$ Calcd. for $C_{13}H_{16}N$ 186.1283; Found 186.1290.

Cinnamonitrile (36)

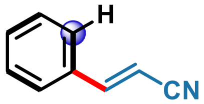

This compound was synthesized by following procedure GP1.

Physical appearance: Colorless gummy liquid.

Column material: 100-200 mesh silica.

Eluent: petroleum ether/ ethyl acetate (98/2, v/v).

Yield: 69% (9 mg).

^{1}H NMR (500 MHz, $CDCl_3$) δ 7.47 – 7.35 (m, 6H), 5.88 (d, *J* = 16.7 Hz, 1H). **^{13}C NMR** (126 MHz, $CDCl_3$) δ 150.73, 133.66, 131.37, 129.26, 127.51, 118.31, 96.48. **HRMS** (ESI-TOF) m/z: $[M + H]^+$ Calcd. for C_9H_8N 130.0657; Found 130.0672.

N,N-dimethylcinnamamide (37)

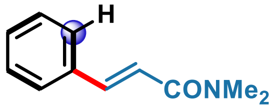

This compound was synthesized by following procedure GP1.

Physical appearance: Colorless gummy liquid.

Column material: 100-200 mesh silica.

Eluent: petroleum ether/ethyl acetate (80/20, v/v).

Yield: 72% (12.7 mg).

^{1}H NMR (400 MHz, CDCl3) δ 7.65 (d, *J* = 15.4 Hz, 1H), 7.55 – 7.46 (m, 2H), 7.40 – 7.30 (m, 3H), 6.87 (d, *J* = 15.5 Hz, 1H), 3.14 (s, 3H), 3.04 (s, 3H). **^{13}C NMR** (101 MHz, $CDCl_3$) δ 166.84, 142.47, 135.44, 129.66, 128.89, 127.90, 117.51, 37.55, 36.06. **HRMS** (ESI-TOF) m/z: $[M + H]^+$ Calcd for $C_{11}H_{14}NO$ 176.1075; Found 176.1083.

(E)-3-(3-(tert-butyl)phenyl)-N,N-dimethylacrylamide (38)

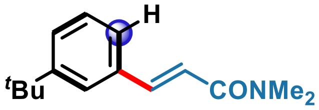

This compound was synthesized by following procedure GP1.

Physical appearance: Colorless gummy liquid.

Column material: 100-200 mesh silica.

Eluent: petroleum ether/ethyl acetate (80/20, v/v).

Yield: 77% (17.8 mg).

^{1}H NMR (500 MHz, $CDCl_3$) δ 7.67 (d, *J* = 15.4 Hz, 1H), 7.51 (t, *J* = 1.9 Hz, 1H), 7.38 (tt, *J* = 7.8, 1.5 Hz, 2H), 7.30 (t, *J* = 7.7 Hz, 1H), 6.87 (d, *J* = 15.4 Hz, 1H), 3.18 (s, 3H), 3.07 (s, 3H), 1.33 (s, 9H). **^{13}C NMR** (101 MHz, $CDCl_3$) δ 167.06, 151.91, 143.18, 135.27, 128.68, 126.92, 125.54, 124.65, 117.27, 37.66, 36.14, 34.88, 31.46. **HRMS** (ESI-TOF) m/z: $[M + H]^+$ Calcd for $C_{15}H_{22}NO$ 232.1701; Found 232.1735.

(E)-N,N-dimethyl-3-(naphthalen-1-yl)acrylamide (39)

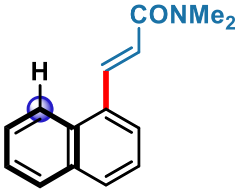

This compound was synthesized by following procedure GP1.

Physical appearance: colorless liquid

Column material: 100-200 mesh silica.

Eluent: petroleum ether/ ethyl acetate 85/15, v/v).

Yield: 46% (10.4 mg).

^{1}H NMR (400 MHz, $CDCl_3$) δ 8.49 (d, *J* = 15.2 Hz, 1H), 8.22 (d, *J* = 8.1 Hz, 1H), 8.05 – 7.78 (m, 2H), 7.71 (d, *J* = 7.2 Hz, 1H), 7.63 – 7.38 (m, 3H), 6.96 (d, *J* = 15.1 Hz, 1H), 3.21 (s, 3H), 3.12 (s, 3H). **^{13}C NMR** (101 MHz, $CDCl_3$) δ 168.88, 139.97, 133.85, 133.39, 131.72, 129.96, 128.77, 126.86, 126.37, 125.58, 124.74, 124.04, 120.78, 37.69, 36.20. **HRMS** (ESI-TOF) m/z: $[M + H]^+$ Calcd. for $C_{15}H_{16}NO$ 226.1232; Found 226.1245.

(E)-3-phenyl-1-(pyrrolidin-1-yl)prop-2-en-1-one (40)[11]

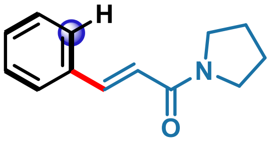

This compound was synthesized by following procedure GP1.

Physical appearance: Colorless gummy liquid.

Column material: 100-200 mesh silica.

Eluent: petroleum ether/ ethyl acetate (80/20, v/v).

Yield: 67% (13.5 mg).

^{1}H NMR (400 MHz, $CDCl_3$) δ 7.70 (d, J = 15.5 Hz, 1H), 7.60 – 7.47 (m, 2H), 7.42 – 7.32 (m, 3H), 6.73 (d, J = 15.5 Hz, 1H), 3.61 (dt, J = 14.4, 6.8 Hz, 4H), 2.05 – 1.96 (m, 2H), 1.96 – 1.81 (m, 2H). **^{13}C NMR** (126 MHz, $CDCl_3$) δ 164.94, 141.93, 135.55, 129.72, 128.96, 128.03, 119.03, 46.80, 46.27, 26.35, 24.55. **HRMS** (ESI-TOF) m/z: $[M + H]^+$ Calcd. for $C_{13}H_{16}NO$ 202.1232; Found 202.1224.

(E)-3-phenyl-1-(piperidin-1-yl)prop-2-en-1-one (41)[11]

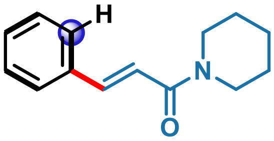

This compound was synthesized by following procedure GP1.

Physical appearance: Colorless gummy liquid.

Column material: 100-200 mesh silica.

Eluent: petroleum ether/ ethyl acetate (75/25, v/v).

Yield: 65% (14 mg).

^{1}H NMR (500 MHz, $CDCl_3$) δ 7.64 (d, J = 15.4 Hz, 1H), 7.55 – 7.49 (m, 2H), 7.41 – 7.31 (m, 3H), 6.90 (d, J = 15.4 Hz, 1H), 3.70 – 3.54 (m, 4H), 1.65 (dq, J = 31.6, 6.0, 5.5 Hz, 6H). **^{13}C NMR** (126 MHz, $CDCl_3$) δ 165.59, 142.36, 135.72, 129.61, 128.96, 127.88, 117.97, 47.24, 43.56, 26.97, 25.82, 24.86. **HRMS** (ESI-TOF) m/z: $[M + H]^+$ Calcd. for $C_{14}H_{18}NO$ 216.1388; Found 216.1402.

Diethyl (E)-(3-methylstyryl)phosphonate (42)

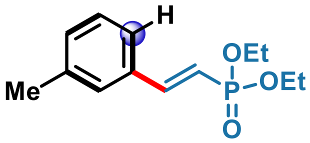

This compound was synthesized by following procedure GP1.

Physical appearance: Colorless gummy liquid.

Column material: 100-200 mesh silica.

Eluent: petroleum ether/ ethyl acetate (75/25, v/v).

Yield: 67% (17.1 mg).

^{1}H NMR (400 MHz, $CDCl_3$) δ 7.45 (dd, *J* = 22.6, 17.5 Hz, 1H), 7.33 – 7.21 (m, 3H), 7.19 – 7.12 (m, 1H), 6.21 (t, J = 17.7 Hz, 1H), 4.33 – 3.96 (m, 4H), 2.33 (s, 3H), 1.32 (t, *J* = 7.1 Hz, 6H). **^{13}C NMR** (101 MHz, $CDCl_3$) δ 149.12, 149.06, 138.62, 135.00, 134.77, 131.18, 128.85, 128.41, 125.05, 114.62, 112.72, 21.42, 16.54, 16.48. **HRMS** (ESI-TOF) m/z: $[M + Na]^+$ Calcd. for $C_{13}H_{19}NaO_3P$ 277.0970; Found 277.0983.

Diethyl (E)-styrylphosphonate (43)[12]

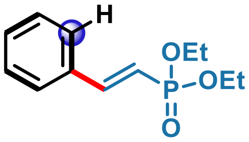

This compound was synthesized by following procedure GP1.

Physical appearance: Colorless gummy liquid.

Column material: 100-200 mesh silica.

Eluent: petroleum ether/ ethyl acetate (75/25, v/v).

Yield: 64% (15.4 mg).

^{1}H NMR (400 MHz, $CDCl_3$) δ 7.59 – 7.45 (m, 3H), 7.39 (dt, *J* = 4.6, 2.9 Hz, 3H), 6.26 (t, *J* = 17.6 Hz, 1H), 4.18 – 4.07 (m, 4H), 1.35 (t, *J* = 7.0 Hz, 6H). **^{13}C NMR** (101 MHz, $CDCl_3$) δ 148.95, 135.20, 134.97, 130.46, 129.07, 127.93, 115.13, 113.23, 62.10, 62.05, 16.66.

Diethyl (E)-(3-(tert-butyl)styryl)phosphonate (44)

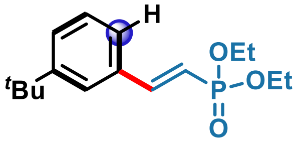

This compound was synthesized by following procedure GP1.

Physical appearance: Colorless gummy liquid.

Column material: 100-200 mesh silica.

Eluent: petroleum ether/ ethyl acetate (75/25, v/v).

Yield: 72% (21.3 mg).

^{1}H NMR (500 MHz, $CDCl_3$) δ 7.55 – 7.47 (m, 2H), 7.42 (dt, *J* = 6.4, 2.2 Hz, 1H), 7.32 (d, *J* = 6.4 Hz, 2H), 6.25 (t, *J* = 17.7 Hz, 1H), 4.24 – 4.05 (m, 4H), 1.35 (t, *J* = 7.1 Hz, 6H), 1.33 (s, 9H). **^{13}C NMR** (126 MHz, $CDCl_3$) δ 152.04, 149.67, 149.62, 134.84, 134.66, 128.81, 127.67, 125.19, 124.92, 114.33, 112.81, 62.06, 34.92, 31.44, 16.60. **HRMS** (ESI-TOF) m/z: $[M + Na]^+$ Calcd. for $C_{16}H_{25}NaO_3P$ 319.1439; Found 319.1450.

(E)-(2-(phenylsulfonyl)vinyl)benzene (45)[13]

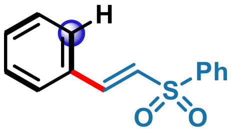

This compound was synthesized by following procedure GP1.

Physical appearance: Colorless gummy liquid.

Column material: 100-200 mesh silica.

Eluent: petroleum ether/ ethyl acetate (85/15, v/v).

Yield: 65% (15.9 mg).

^{1}H NMR (500 MHz, $CDCl_3$) δ 7.95 (d, *J* = 7.7 Hz, 2H), 7.69 (d, *J* = 15.4 Hz, 1H), 7.62 (t, *J* = 7.5 Hz, 1H), 7.55 (t, *J* = 7.6 Hz, 2H), 7.51 – 7.46 (m, 2H), 7.39 (q, *J* = 6.8, 6.3 Hz, 3H), 6.86 (d, *J* = 15.4 Hz, 1H). **^{13}C NMR** (126 MHz, $CDCl_3$) δ 142.71, 140.92, 133.59, 132.56, 131.43, 129.54, 129.29, 128.78, 127.85, 127.49.

Dimethyl 2-(3-(tert-butyl)phenyl)maleate (46)

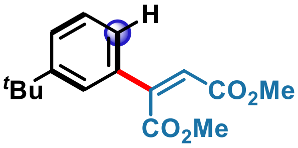

This compound was synthesized by following procedure GP1.

Physical appearance: Colorless gummy liquid.

Column material: 100-200 mesh silica.

Eluent: petroleum ether/ ethyl acetate (97/3, v/v).

Yield: 40% (*Z:E* = 10:1, 11.1 mg).

^{1}H NMR (400 MHz, $CDCl_3$) δ 7.51 – 7.44 (m, 2H), 7.34 (td, *J* = 7.7, 0.6 Hz, 1H), 7.31 – 7.27 (m, 1H), 6.31 (s, 1H), 3.95 (s, 3H), 3.79 (s, 3H), 1.32 (s, 9H). **^{13}C NMR** (101 MHz, $CDCl_3$) δ 168.69, 165.73, 152.28, 149.83, 133.13, 128.98, 128.15, 124.31, 123.77, 117.01, 52.89, 52.25, 35.05, 31.42. **HRMS** (ESI-TOF) m/z: $[M + H]^+$ Calcd. for $C_{16}H_{21}O_4$ 277.1440; Found 277.1473.

(E)-1-(3-(tert-butyl)styryl)-2,3,4,5,6-pentafluorobenzene (47)

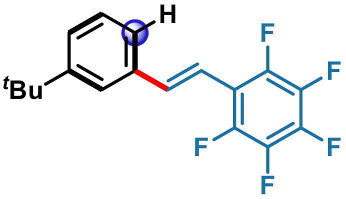

This compound was synthesized by following procedure GP1.

Physical appearance: White solid

Column material: 100-200 mesh silica.

Eluent: petroleum ether

Yield: 83% (27.1 mg).

^{1}H NMR (500 MHz, $CDCl_3$) δ 7.51 (s, 1H), 7.46 (d, *J* = 16.8 Hz, 1H), 7.42 – 7.31 (m, 3H), 6.98 (d, *J* = 16.8 Hz, 1H), 1.38 (d, *J* = 1.2 Hz, 9H). **^{13}C NMR** (126 MHz, $CDCl_3$) δ 152.04, 145.04 (dm, *J* = 257.1 Hz), 139.9 (dm, *J* = 239.4 Hz), 138.07 (dt, *J* = 9.2, 4.5 Hz), 136.96 (d, *J* = 16.4 Hz), 136.40, 128.83, 126.43, 124.63, 123.84, 112.77 (td, *J* = 13.7, 4.2 Hz), 112.52 (d, *J* = 3.2 Hz), 34.94, 31.51. **^{19}F NMR** (471 MHz, $CDCl_3$) δ -142.84 (dd, *J* = 22.6, 8.9 Hz), -156.83 (t, *J* = 21.2 Hz), -163.06 (td, *J* = 22.1, 8.6 Hz). **HRMS** (ESI-TOF) m/z: $[M + H]^+$ Calcd. for $C_{18}H_{16}F_5$ 327.1172; Found 327.1180.

(E)-1-(2-(perfluorophenyl)vinyl)naphthalene (48)[14]

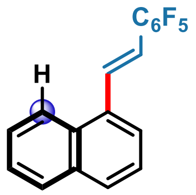

This compound was synthesized by following procedure GP1.

Physical appearance: White solid.

Column material: 100-200 mesh silica.

Eluent: petroleum ether

Yield: 70% (22.4 mg).

^{1}H NMR (500 MHz, $CDCl_3$) δ 8.23 (d, *J* = 16.5 Hz, 1H), 8.14 (d, *J* = 8.3 Hz, 1H), 7.94 – 7.86 (m, 2H), 7.77 (d, *J* = 7.2 Hz, 1H), 7.61 – 7.46 (m, 3H), 7.03 (d, *J* = 16.5 Hz, 1H). **^{13}C NMR** (126 MHz, $CDCl_3$) δ 145.11 (d, *J* = 251.4 Hz), 140.06 (d, *J* = 262.9 Hz), 138.03 (d, *J* = 247.0 Hz), 135.46 – 134.73 (m), 134.51, 133.87, 131.42, 129.52, 128.93, 126.83, 126.35, 125.86, 124.16, 123.63, 115.71, 112.78. **^{19}F NMR** (471 MHz, $CDCl_3$) δ -141.07 – -146.09 (m), -156.23, -162.79 (d, *J* = 7.3 Hz). **HRMS** (ESI-TOF) m/z: $[M + Na]^+$ Calcd. for $C_{18}H_9NaF_5$ 343.0522; Found 343.0524.

(E)-1-(tert-butyl)-3-(4-nitrostyryl)benzene (49)

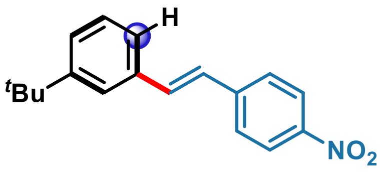

This compound was synthesized by following procedure GP1.

Physical appearance: Colorless gummy liquid.

Column material: 100-200 mesh silica.

Eluent: petroleum ether/ ethyl acetate (99.5/0.5, v/v).

Yield: 66% (18.6 mg).

^{1}H NMR (500 MHz, $CDCl_3$) δ 8.22 (d, *J* = 8.4 Hz, 2H), 7.64 (d, *J* = 8.4 Hz, 2H), 7.55 (s, 1H), 7.45 – 7.27 (m, 4H), 7.14 (d, *J* = 16.3 Hz, 1H), 1.37 (s, 9H). **^{13}C NMR** (126 MHz, $CDCl_3$) δ 152.03, 146.91, 144.25, 136.08, 134.16, 128.86, 127.01, 126.30, 126.14, 124.58, 124.36, 124.17, 34.95, 31.53. **HRMS** (ESI-TOF) m/z: $[M + H]^+$ Calcd. for $C_{18}H_{20}NO_2$ 282.1494; Found 282.1513.

(E)-1,2-difluoro-4-styrylbenzene (50)[15]

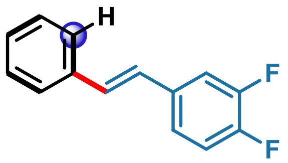

This compound was synthesized by following procedure GP1.

Physical appearance: Colorless gummy liquid.

Column material: 100-200 mesh silica.

Eluent: petroleum ether/ ethyl acetate (99.5/0.5, v/v).

Yield: 60% (13 mg).

^{1}H NMR (400 MHz, $CDCl_3$) δ 7.52 – 7.43 (m, 2H), 7.40 – 7.27 (m, 4H), 7.22 – 7.09 (m, 2H), 7.01 (s, 2H). **^{13}C NMR** (101 MHz, $CDCl_3$) δ 151.66 (dd, *J* = 79.5, 12.9 Hz), 149.07 (dd, *J* = 80.8 Hz, 13.1 Hz), 136.91, 134.88, 131.58, 130.42 – 129.46 (m), 128.99, 128.26, 126.77 (d, *J* = 6.0 Hz), 122.95 (dd, *J* = 6.1, 3.5 Hz), 117.63 (d, *J* = 17.4 Hz), 114.85 (d, *J* = 17.8 Hz). **^{19}F NMR** (376 MHz, $CDCl_3$) δ -137.78 (ddd, *J* = 19.6, 11.7, 8.0 Hz), -138.38 – -139.79 (m). **HRMS** (ESI-TOF) m/z: $[M + H]^+$ Calcd. for $C_{14}H_{11}F_2$ 217.0829; Found 217.0835.

(E)-1-fluoro-4-styrylbenzene (51)[16]

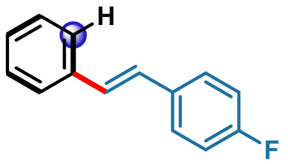

This compound was synthesized by following procedure GP1.

Physical appearance: Colorless gummy liquid.

Column material: 100-200 mesh silica.

Eluent: petroleum ether

Yield: 61% (12.1 mg).

^{1}H NMR (400 MHz, $CDCl_3$) δ 7.54 – 7.45 (m, 4H), 7.36 (dd, *J* = 8.4, 6.8 Hz, 2H), 7.30 – 7.23 (m, 1H), 7.11 – 6.98 (m, 4H). **^{13}C NMR** (126 MHz, $CDCl_3$) δ 162.56 (d, *J* = 246.9 Hz), 137.39, 133.74 (d, *J* = 3.8 Hz), 128.93, 128.72 (d, *J* = 2.5 Hz), 128.20 (d, *J* = 7.5 Hz), 127.89, 127.71, 126.66, 115.83 (d, *J* = 21.4 Hz). **^{19}F NMR** (376 MHz, $CDCl_3$) δ -114.27. **HRMS** (ESI-TOF) m/z: $[M + H]^+$ Calcd. for $C_{14}H_{12}F$ 199.0923; Found 199.0917.

(E)-1-chloro-4-styrylbenzene (52)

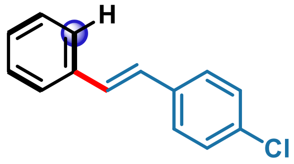

This compound was synthesized by following procedure GP1.

Physical appearance: Colorless gummy liquid.

Column material: 100-200 mesh silica.

Eluent: petroleum ether/ ethyl acetate (99.5/0.5, v/v).

Yield: 62% (13.4 mg).

^{1}H NMR (400 MHz, $CDCl_3$) δ 7.55 – 7.49 (m, 2H), 7.47 – 7.42 (m, 2H), 7.42 – 7.27 (m, 5H), 7.08 (d, *J* = 2.8 Hz, 2H). **^{13}C NMR** (101 MHz, $CDCl_3$) δ 137.16, 136.03, 133.36, 129.50, 129.04, 128.94, 128.07, 127.86, 127.55, 126.75. **HRMS** (ESI-TOF) m/z: $[M + H]^+$ Calcd. for $C_{14}H_{12}Cl$ 215.0628; Found 215.0631.

(E)-4-styrylphenyl acetate (53)

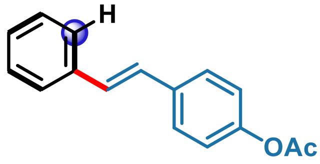

This compound was synthesized by following procedure GP1.

Physical appearance: Colorless gummy liquid.

Column material: 100-200 mesh silica.

Eluent: petroleum ether/ ethyl acetate (99.5/0.5, v/v).

Yield: 56% (13.4 mg).

^{1}H NMR (500 MHz, $CDCl_3$) δ 7.51 (dd, *J* = 7.9, 5.8 Hz, 4H), 7.36 (t, *J* = 7.6 Hz, 2H), 7.30 – 7.22 (m, 1H), 7.13 – 7.01 (m, 4H), 2.31 (s, 3H). **^{13}C NMR** (126 MHz, $CDCl_3$) δ 169.69, 150.28, 137.39, 135.39, 129.18, 128.92, 127.93, 127.88, 127.64, 126.73, 122.02, 21.38. **HRMS** (ESI-TOF) m/z: $[M + H]^+$ Calcd. for $C_{16}H_{15}O_2$ 239.1072; Found 239.1077.

(E)-1,2-diphenylethene (54)[17]

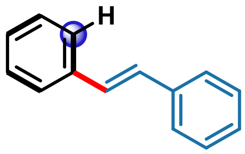

This compound was synthesized by following procedure GP1.

Physical appearance: Colorless gummy liquid.

Column material: 100-200 mesh silica.

Eluent: petroleum ether.

Yield: 26% (4.7 mg).

^{1}H NMR (400 MHz, $CDCl_3$) δ 7.63 – 7.52 (m, 4H), 7.42 (dd, *J* = 8.4, 6.9 Hz, 4H), 7.36 – 7.30 (m, 2H), 7.18 (s, 2H). Spectral data of this isolated compound matches with the literature report.[17]

Ethene-1,1,2-triyltribenzene (55)

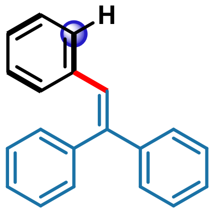

This compound was synthesized by following procedure GP1.

Physical appearance: white solid.

Column material: 100-200 mesh silica.

Eluent: petroleum ether

Yield: 16% (4.1 mg).

^{1}H NMR (400 MHz, $CDCl_3$) δ 7.36 – 7.27 (m, 8H), 7.23 – 7.18 (m, 2H), 7.16 – 7.09 (m, 3H), 7.03 (dd, *J* = 7.8, 1.9 Hz, 2H), 6.97 (s, 1H). **^{13}C NMR** (101 MHz, $CDCl_3$) δ 143.66, 142.82, 140.59, 137.61, 130.61, 129.76, 128.84, 128.42, 128.38, 128.18, 127.83, 127.72, 127.62, 126.96. **HRMS** (ESI-TOF) m/z: $[M + Na]^+$ Calcd. for $C_{20}H_{16}Na$ 279.1150; Found 279.1157.

(E)-4-(3-(tert-butyl)phenyl)but-3-en-2-one

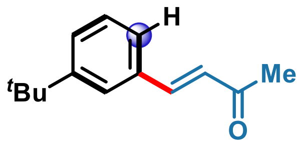

This compound was synthesized by following procedure GP1 (using 4-^{t}Bu-cyclohexane carboxylic acid with 3-buten-2-ol).

Yield: 46% (9.3 mg).

Spectral data: Identical as entry (34)

(E)-4-phenylbut-3-en-2-one (56)

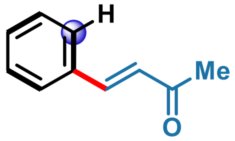

This compound was synthesized by following procedure GP1.

Physical appearance: colorless liquid

Column material: 100-200 mesh silica.

Eluent: petroleum ether/ ethyl acetate (95/5, v/v).

Yield: 45% (6.6 mg).

^{1}H NMR (400 MHz, $CDCl_3$) δ 7.54 – 7.48 (m, 3H), 7.48 – 7.37 (m, 3H), 6.70 (d, J = 16.3 Hz, 1H), 2.36 (s, 3H). **^{13}C NMR** (101 MHz, $CDCl_3$) δ 198.53, 143.57, 134.53, 130.64, 129.09, 128.37, 127.26, 27.62. **HRMS** (ESI-TOF) m/z: $[M + H]^+$ Calcd for $C_{10}H_{11}O$ 147.0810; Found 147.0815.

(E)-4-(m-tolyl)but-3-en-2-one (57)

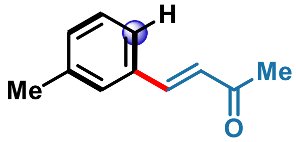

This compound was synthesized by following procedure GP1.

Physical appearance: colorless liquid

Column material: 100-200 mesh silica.

Eluent: petroleum ether/ ethyl acetate (95/5, v/v).

Yield: 42% (6.8 mg).

^{1}H NMR (500 MHz, $CDCl_3$) δ 7.48 (d, J = 16.3 Hz, 1H), 7.34 (dt, J = 8.1, 1.4 Hz, 2H), 7.27 (td, J = 7.4, 1.1 Hz, 1H), 7.24 – 7.17 (m, 1H), 6.69 (d, J = 16.3 Hz, 1H), 2.36 (s, 6H). **^{13}C NMR** (126 MHz, $CDCl_3$) δ 198.62, 143.82, 138.77, 134.49, 131.52, 129.05, 128.99, 127.10, 125.61, 27.61, 21.45. **HRMS** (ESI-TOF) m/z: $[M + H]^+$ Calcd. for $C_{11}H_{13}O$ 161.0966; Found 161.0968.

(E)-4-(p-tolyl)but-3-en-2-one (58)

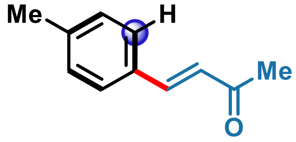

This compound was synthesized by following procedure GP1.

Physical appearance: colorless liquid

Column material: 100-200 mesh silica.

Eluent: petroleum ether/ ethyl acetate (95/5, v/v).

Yield: 48% (7.7 mg).

^{1}**H NMR** (500 MHz, $CDCl_3$) δ 7.49 (d, *J* = 16.3 Hz, 1H), 7.46 – 7.34 (m, 2H), 7.20 (d, *J* = 7.9 Hz, 2H), 6.68 (d, *J* = 16.3 Hz, 1H), 2.38 (s, 3H), 2.37 (s, 3H). ^{13}**C NMR** (126 MHz, $CDCl_3$) δ 198.76, 143.77, 141.25, 131.85, 129.92, 128.47, 126.45, 27.62, 21.69. **HRMS** (ESI-TOF) m/z: $[M + H]^+$ Calcd. for $C_{11}H_{13}O$ 161.0966; Found 161.0972.

(E)-4-(3-(trifluoromethyl)phenyl)but-3-en-2-one (59)[18]

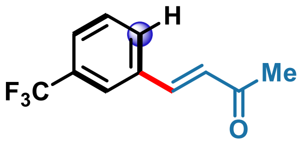

This compound was synthesized by following procedure GP1.

Physical appearance: colorless liquid

Column material: 100-200 mesh silica.

Eluent: petroleum ether/ ethyl acetate 98/2, v/v).

Yield: 40% (8.6 mg).

^{1}**H NMR** (400 MHz, $CDCl_3$) δ 7.79 (td, *J* = 1.7, 0.9 Hz, 1H), 7.72 (d, *J* = 7.7 Hz, 1H), 7.64 (dt, *J* = 1.8, 0.9 Hz, 1H), 7.60 – 7.47 (m, 2H), 6.77 (d, *J* = 16.3 Hz, 1H), 2.40 (s, 3H). ^{13}**C NMR** (101 MHz, CDCl3) δ 198.12, 141.61, 135.48, 131.83, 131.41, 129.76, 128.74 (d, *J* = 1 Hz), 127.18 – 127.02 (m), 125.04 (d, *J* = 1 Hz), 123.96 (d, *J* = 274.7 Hz), 28.07. ^{19}**F NMR** (471 MHz, $CDCl_3$) δ -62.93. **HRMS** (ESI-TOF) m/z: $[M + H]^+$ Calcd. for $C_{11}H_{10}F_3O$ 215.0679; Found 215.0713.

(E)-4-(3-pentylphenyl)but-3-en-2-one (60)

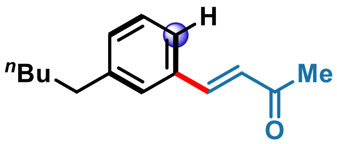

This compound was synthesized by following procedure GP1.

Physical appearance: colorless liquid

Column material: 100-200 mesh silica.

Eluent: petroleum ether/ ethyl acetate (95/5, v/v).

Yield: 38% (8.3 mg).

^{1}H NMR (500 MHz, $CDCl_3$) δ 7.50 (d, *J* = 16.3 Hz, 1H), 7.37 (d, *J* = 7.7 Hz, 2H), 7.31 (t, *J* = 7.5 Hz, 1H), 7.22 (d, *J* = 7.5 Hz, 1H), 6.71 (d, *J* = 16.3 Hz, 1H), 2.62 (t, *J* = 7.8 Hz, 2H), 2.38 (s, 3H), 1.48 – 1.12 (m, 6H), 0.90 (t, *J* = 6.7 Hz, 3H). **^{13}C NMR** (126 MHz, $CDCl_3$) δ 198.78, 144.06, 143.95, 134.56, 131.03, 129.08, 128.55, 127.19, 125.88, 35.97, 31.65, 31.27, 27.66, 22.73, 14.23. **HRMS** (ESI-TOF) m/z: $[M + H]^+$ Calcd. for $C_{15}H_{21}O$ 217.1592; Found 217.1589.

Methyl (E)-3-(3-oxobut-1-en-1-yl)benzoate (61)

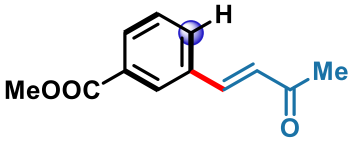

This compound was synthesized by following procedure GP1.

Physical appearance: colorless liquid

Column material: 100-200 mesh silica.

Eluent: petroleum ether/ ethyl acetate (95/5, v/v).

Yield: 41% (8.4 mg).

^{1}H NMR (400 MHz, $CDCl_3$) δ 8.21 (s, 1H), 8.05 (dt, *J* = 7.7, 1.5 Hz, 1H), 7.71 (dt, *J* = 7.8, 1.5 Hz, 1H), 7.53 (d, *J* = 16.3 Hz, 1H), 7.47 (t, *J* = 7.8 Hz, 1H), 6.78 (d, *J* = 16.2 Hz, 1H), 3.93 (s, 3H), 2.39 (s, 3H). **^{13}C NMR** (101 MHz, $CDCl_3$) δ 198.34, 166.63, 142.27, 134.98, 132.53, 131.46, 131.16, 129.40, 129.32, 128.26, 52.56, 27.93. **HRMS** (ESI-TOF) m/z: $[M + H]^+$ Calcd. for $C_{12}H_{13}O_3$ 205.0865; Found 205.0872.

(E)-4-(4'-chloro-[1,1'-biphenyl]-3-yl)but-3-en-2-one (62)

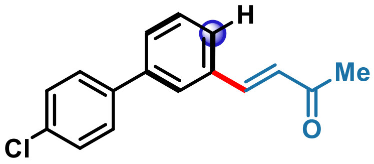

Physical appearance: colorless liquid

Column material: 100-200 mesh silica.

Eluent: petroleum ether/ethyl acetate (95/5, v/v).

Yield: 36% (9.3 mg).

^{1}H NMR (400 MHz, $CDCl_3$) δ 7.71 (t, *J* = 1.9 Hz, 1H), 7.64 – 7.51 (m, 5H), 7.49 (d, *J* = 7.6 Hz, 1H), 7.46 – 7.41 (m, 2H), 6.78 (d, *J* = 16.3 Hz, 1H), 2.41 (s, 3H). **^{13}C NMR** (101 MHz, $CDCl_3$) δ 198.49, 143.26, 141.09, 139.02, 135.33, 134.16, 129.77, 129.31, 128.60, 127.85, 127.47, 127.11, 27.84. **HRMS** (ESI-TOF) m/z: $[M + H]^+$ Calcd for $C_{16}H_{14}ClO$ 257.0733; Found 257.0742.

Ethyl cinnamate

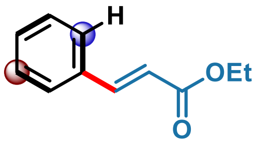

This compound was synthesized by following procedure GP1 (using 4-hydroxy-cyclohexane carboxylic acid with ethyl acrylate).

Yield: 60% (10.6 mg).

Spectral data: Identical as entry (2)

Ethyl cinnamate

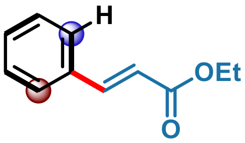

This compound was synthesized by following procedure GP1 (using 3-hydroxy-cyclohexane carboxylic acid with ethyl acrylate).

Yield: 43% (7.6 mg).

Spectral data: Identical as entry (2)

Benzyl cinnamate

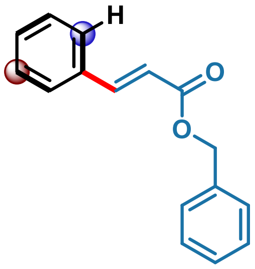

This compound was synthesized by following procedure GP1 (using 4-hydroxy-cyclohexane carboxylic acid with benzyl acrylate).

Yield: 51% (12.2 mg).

Spectral data: Identical as entry (28)

(E)-(2-(phenylsulfonyl)vinyl)benzene

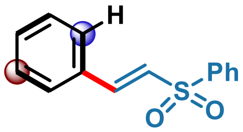

This compound was synthesized by following procedure GP1 (using 4-hydroxy-cyclohexane carboxylic acid with phenyl vinyl sulfone).

Yield: 46% (11.2 mg).

Spectral data: Identical as entry (45)

(E)-1,2,3,4,5-pentafluoro-6-styrylbenzene (63)[14]

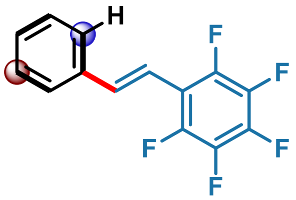

This compound was synthesized by following procedure GP1 (using 4-hydroxy-cyclohexane carboxylic acid with pentafluorostyrene).

Physical appearance: White solid

Column material: 100-200 mesh silica.

Eluent: petroleum ether

Yield: 65% (17.6 mg).

^{1}H NMR (500 MHz, $CDCl_3$) δ 7.59 – 7.51 (m, 2H), 7.47 – 7.38 (m, 3H), 7.35 (t, *J* = 7.3 Hz, 1H), 6.99 (d, *J* = 16.8 Hz, 1H). **^{13}C NMR** (126 MHz, $CDCl_3$) δ 145.03 (dtd, *J* = 250.6, 8.0, 4.0 Hz), 139.94 (dm, *J* = 248.8 Hz), 137.39 (td, *J* = 8.4, 2.7 Hz), 137.18 – 136.79 (m), 136.67, 129.20, 129.07, 127.10, 112.89 (d, *J* = 3.1 Hz), 112.60 (td, *J* = 13.8, 4.3 Hz). **^{19}F NMR** (471 MHz, $CDCl_3$) δ -142.76 (dd, *J* = 22.5, 9.0 Hz), -156.56 (t, *J* = 21.4 Hz), -162.97 (td, *J* = 22.1, 8.7 Hz). **HRMS** (ESI-TOF) m/z: $[M + H]^+$ Calcd. for $C_{14}H_8F_5$ 271.0546; Found 271.0552.

Ethyl cinnamate

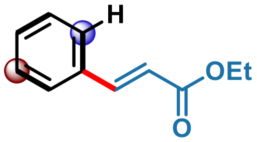

This compound was synthesized by following procedure GP1 (using 4-acetoxycyclohexane-1-carboxylic acid with ethyl acrylate).

Yield: 42% (7.4 mg).

Spectral data: Identical as entry (2)

4-chloro-1,1'-biphenyl (69)

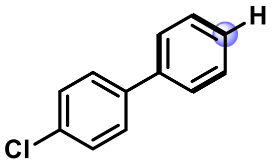

This compound was synthesized by following procedure GP2.

Physical appearance: white solid.

Column material: 100-200 mesh silica.

Eluent: petroleum ether

Yield: 17% (3.3 mg).

^{1}H NMR (400 MHz, $CDCl_3$) δ 7.58 – 7.50 (m, 4H), 7.48 – 7.39 (m, 4H), 7.39 – 7.33 (m, 1H). **^{13}C NMR** (126 MHz, $CDCl_3$) δ 140.17, 139.85, 133.57, 129.23, 129.08, 128.58, 128.40, 127.78, 127.17. **HRMS** (ESI-TOF) m/z: $[M + H]^+$ Calcd. for $C_{12}H_{10}Cl$ 189.0471; Found 189.0492.

1-(p-tolyl)naphthalene (22')

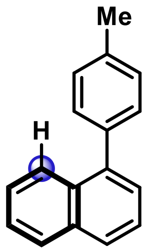

This compound was synthesized by following procedure GP2.

Physical appearance: white solid.

Column material: 100-200 mesh silica.

Eluent: petroleum ether

Yield: 44% (9.6 mg).

^{1}H NMR (400 MHz, $CDCl_3$) δ 7.91 (td, *J* = 8.0, 1.1 Hz, 2H), 7.85 (dd, *J* = 8.1, 1.2 Hz, 1H), 7.56 – 7.37 (m, 6H), 7.31 (d, *J* = 7.7 Hz, 2H), 2.46 (s, 3H). **^{13}C NMR** (101 MHz, $CDCl_3$) δ 140.45, 138.02, 137.13, 134.01, 131.92, 130.16, 129.18, 128.45, 127.64, 127.09, 126.31, 126.13, 125.92, 125.61, 21.46. **HRMS** (ESI-TOF) m/z: $[M + Na]^+$ Calcd. for $C_{17}H_{14}Na$ 241.0993; Found 241.1027.

(E)-2-(3-ethoxy-3-oxoprop-1-en-1-yl)cyclopent-2-en-1-yl cyclopentanecarboxylate (71)

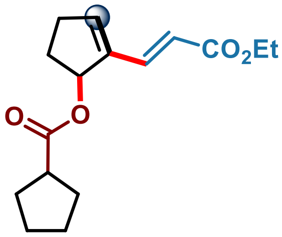

This compound was synthesized by following procedure GP3.

Physical appearance: Colorless gummy liquid.

Column material: 100-200 mesh silica.

Eluent: petroleum ether/ ethyl acetate (95/5, v/v).

Yield: 34% (9.5 mg).

^{1}H NMR (500 MHz, $CDCl_3$) δ 7.40 (d, *J* = 16.0 Hz, 1H), 6.43 (t, *J* = 2.2 Hz, 1H), 5.93 (dd, *J* = 7.3, 2.8 Hz, 1H), 5.81 (d, *J* = 16.0 Hz, 1H), 4.20 (qd, *J* = 7.2, 2.4 Hz, 2H), 2.76 – 2.56 (m, 2H), 2.51 – 2.34 (m, 2H), 1.91 – 1.81 (m, 3H), 1.84 – 1.71 (m, 2H), 1.73 – 1.63 (m, 2H), 1.61 – 1.49 (m, 2H), 1.28 (t, *J* = 7.1 Hz, 3H). **^{13}C NMR** (101 MHz, $CDCl_3$) δ 176.78, 167.37, 144.85, 139.51, 138.28, 119.55, 77.47, 60.59, 44.17, 31.74, 31.53, 30.23, 30.07, 26.00, 25.93, 14.49. **HRMS** (ESI-TOF) m/z: $[M + H]^+$ Calcd. for $C_{16}H_{23}O_4$ 279.1596; Found 279.1666.

(E)-2-(3-butoxy-3-oxoprop-1-en-1-yl)cyclopent-2-en-1-yl cyclopentanecarboxylate (72)

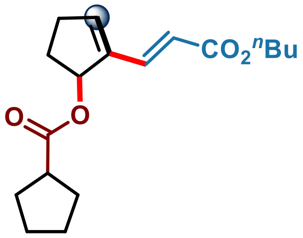

This compound was synthesized by following procedure GP3.

Physical appearance: Colorless gummy liquid.

Column material: 100-200 mesh silica.

Eluent: petroleum ether/ ethyl acetate (95/5, v/v).

Yield: 40% (12.3 mg).

^{1}H NMR (400 MHz, $CDCl_3$) δ 7.39 (d, *J* = 15.9 Hz, 1H), 6.43 (t, *J* = 2.6 Hz, 1H), 5.93 (dd, *J* = 6.7, 3.2 Hz, 1H), 5.81 (dd, *J* = 16.0, 0.9 Hz, 1H), 4.14 (t, *J* = 6.6 Hz, 2H), 2.86 – 2.57 (m, 2H), 2.51 – 2.33 (m, 2H), 1.95 – 1.73 (m, 5H), 1.71 – 1.60 (m, 4H), 1.58 – 1.52 (m, 2H), 1.45 – 1.31 (m, 2H), 0.93 (t, *J* = 7.4 Hz, 3H). **^{13}C NMR** (101 MHz, $CDCl_3$) δ 176.79, 167.46, 144.84, 139.47, 138.24, 119.53, 77.47, 64.51, 44.15, 31.70, 31.53, 30.91, 30.26, 30.01, 25.98, 25.91, 19.35, 13.91. **HRMS** (ESI-TOF) m/z: $[M + H]^+$ Calcd. for $C_{18}H_{27}O_4$ 307.1904; Found 307.1846.

(E)-2-(3-(cyclohexyloxy)-3-oxoprop-1-en-1-yl)cyclopent-2-en-1-yl cyclopentanecarboxylate (73)

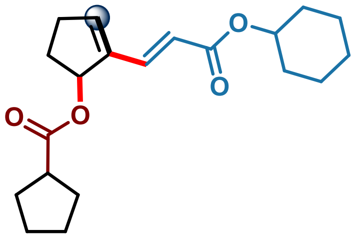

This compound was synthesized by following procedure GP3.

Physical appearance: Colorless gummy liquid.

Column material: 100-200 mesh silica.

Eluent: petroleum ether/ ethyl acetate (95/5, v/v).

Yield: 38% (12.6 mg).

^{1}H NMR (400 MHz, $CDCl_3$) δ 7.41 (d, *J* = 16.0 Hz, 1H), 6.45 (t, *J* = 2.6 Hz, 1H), 5.96 (dd, *J* = 6.7, 3.3 Hz, 1H), 5.82 (dd, *J* = 16.0, 0.9 Hz, 1H), 4.96 – 4.77 (m, 1H), 2.82 – 2.60 (m, 2H), 2.55 – 2.34 (m, 2H), 1.93 – 1.67 (m, 12H), 1.59 – 1.53 (m, 2H), 1.51 – 1.34 (m, 5H). **^{13}C NMR** (101 MHz, $CDCl_3$) δ 176.83, 166.81, 144.61, 139.51, 137.94, 120.18, 77.43, 72.77, 44.16, 31.86, 31.71, 31.53, 30.26, 30.03, 25.99, 25.93, 25.63, 23.92. **HRMS** (ESI-TOF) m/z: $[M + H]^+$ Calcd. for $C_{20}H_{29}O_4$ 333.2066; Found 333.2082.

(E)-2-(3-methoxy-3-oxoprop-1-en-1-yl)cyclopent-2-en-1-yl cyclopentanecarboxylate (74)

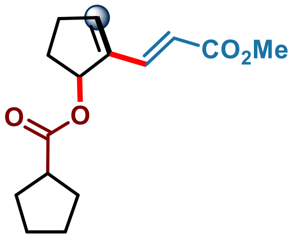

This compound was synthesized by following procedure GP3.

Physical appearance: Colorless gummy liquid.

Column material: 100-200 mesh silica.

Eluent: petroleum ether/ ethyl acetate (95/5, v/v).

Yield: 36% (9.6 mg).

^{1}H NMR (400 MHz, $CDCl_3$) δ 7.44 (d, *J* = 16.0 Hz, 1H), 6.47 (t, *J* = 2.6 Hz, 1H), 5.95 (dd, *J* = 6.7, 3.1 Hz, 1H), 5.85 (dd, *J* = 16.0, 0.9 Hz, 1H), 3.77 (s, 3H), 2.88 – 2.59 (m, 2H), 2.55 – 2.36 (m, 2H), 1.98 – 1.66 (m, 7H), 1.61 – 1.52 (m, 2H). **^{13}C NMR** (101 MHz, $CDCl_3$) δ 176.78, 167.83, 145.17, 139.45, 138.61, 119.01, 77.43, 51.84, 44.15, 31.74, 31.55, 30.24, 30.07, 26.01, 25.93. **HRMS** (ESI-TOF) m/z: $[M + Na]^+$ Calcd. for $C_{15}H_{20}NaO_4$ 287.1259; Found 287.1277.

(E)-2-(3-(benzyloxy)-3-oxoprop-1-en-1-yl)cyclopent-2-en-1-yl cyclopentanecarboxylate (75)

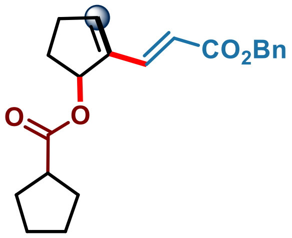

This compound was synthesized by following procedure GP3.

Physical appearance: Colorless gummy liquid.

Column material: 100-200 mesh silica.

Eluent: petroleum ether/ ethyl acetate (95/5, v/v).

Yield: 40% (13.6 mg).

^{1}H NMR (400 MHz, $CDCl_3$) δ 7.45 (d, *J* = 16.0 Hz, 1H), 7.39 – 7.30 (m, 5H), 6.45 (t, *J* = 2.7 Hz, 1H), 5.96 – 5.90 (m, 1H), 5.87 (dd, *J* = 16.0, 1.0 Hz, 1H), 5.20 (s, 2H), 2.85 – 2.59 (m, 2H), 2.54 – 2.28 (m, 2H), 1.91 – 1.64 (m, 7H), 1.60 – 1.47 (m, 2H). **^{13}C NMR** (101 MHz, $CDCl_3$) δ 176.79, 167.15, 145.39, 139.41, 138.91, 136.27, 128.75, 128.38, 128.34, 119.08, 77.43, 66.40, 44.11, 31.72, 31.58, 30.23, 30.05, 25.99, 25.92. **HRMS** (ESI-TOF) m/z: [M + Na]$^+$ Calcd. for $C_{21}H_{24}NaO_4$ 363.1572; Found 363.1588.

(E)-2-(3-oxobut-1-en-1-yl)cyclopent-2-en-1-yl cyclopentanecarboxylate (76)

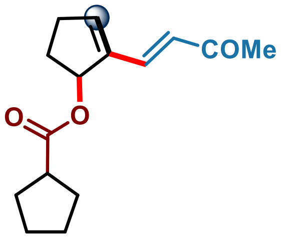

This compound was synthesized by following procedure GP3.

Physical appearance: Colorless gummy liquid.

Column material: 100-200 mesh silica.

Eluent: petroleum ether/ ethyl acetate (95/5, v/v).

Yield: 42% (10.4 mg).

^{1}H NMR (500 MHz, $CDCl_3$) δ 7.25 (d, *J* = 16.1 Hz, 1H), 6.50 (t, *J* = 2.6 Hz, 1H), 6.09 (d, *J* = 16.2 Hz, 1H), 5.93 (dd, *J* = 7.4, 3.0 Hz, 1H), 2.80 – 2.60 (m, 2H), 2.53 – 2.37 (m, 2H), 2.27 (s, 3H), 1.93 – 1.81 (m, 3H), 1.81 – 1.73 (m, 2H), 1.73 – 1.63 (m, 2H), 1.61 – 1.50 (m, 2H). **^{13}C NMR** (126 MHz, $CDCl_3$) δ 198.80, 176.75, 146.08, 139.76, 136.95, 128.07, 77.37, 44.13, 31.73, 31.69, 30.27, 30.06, 27.93, 26.01, 25.93. **HRMS** (ESI-TOF) m/z: [M + H]$^+$ Calcd. for $C_{15}H_{21}O_3$ 249.1485; Found 249.1424.

(E)-2-(3-ethoxy-3-oxoprop-1-en-1-yl)cyclopent-2-en-1-yl cyclobutanecarboxylate (77) & (E)-2-(3-ethoxy-3-oxoprop-1-en-1-yl)cyclopent-2-en-1-yl cyclopentanecarboxylate (71) (inseparable mixture of products)

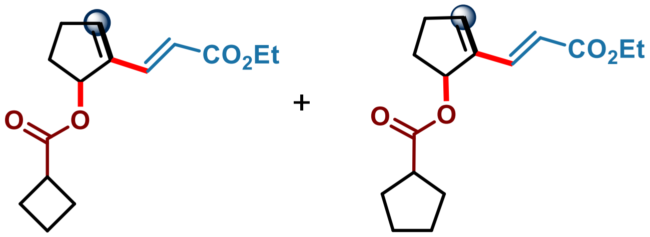

This compound was synthesized by following procedure GP3.

Physical appearance: Colorless gummy liquid.

Column material: 100-200 mesh silica.

Eluent: petroleum ether/ ethyl acetate (95/5, v/v).

Overall yield: 47% (product mixture = 1.14:1, 13 mg).

^{1}H NMR (500 MHz, $CDCl_3$) δ 7.40 (dd, *J* = 16.0, 1.8 Hz, 2H), 6.61 – 6.31 (m, 2H), 6.03 – 5.89 (m, 2H), 5.82 (d, *J* = 16.0 Hz, 2H), 4.32 – 4.08 (m, 4H), 3.19 – 3.03 (m, 1H), 2.80 – 2.56 (m, 3H), 2.58 – 2.39 (m, 4H), 2.35 – 2.11 (m, 4H), 1.99 – 1.65 (m, 10H), 1.57 – 1.51 (m, 2H), 1.29 (t, *J* = 7.1 Hz, 6H). **^{13}C NMR** (126 MHz, $CDCl_3$) δ 176.78, 175.51, 167.37, 144.89, 144.83, 139.53, 139.46, 138.29, 119.56, 77.56, 77.43, 60.60, 44.18, 38.42, 31.76, 31.54, 30.23, 30.08, 29.92, 29.58, 26.00, 25.94, 25.39, 25.36, 18.62, 14.50, 14.33. **HRMS** (ESI-TOF) m/z: $[M + H]^+$ Calcd. for $C_{15}H_{21}O_4$ 265.1440; Found 265.1457.

2-((E)-3-ethoxy-3-oxoprop-1-en-1-yl)cyclopent-2-en-1-yl 2-methylcyclohexane-1-carboxylate (78)

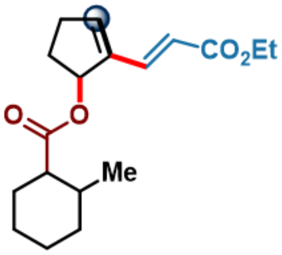

This compound was synthesized by following procedure GP3.

Physical appearance: Colorless gummy liquid.

Column material: 100-200 mesh silica.

Eluent: petroleum ether/ ethyl acetate (95/5, v/v).

Yield: 40% (d.r. 1.2:1, 12.3 mg).

^{1}H NMR (400 MHz, $CDCl_3$) δ 7.40 (d, *J* = 16.0 Hz, 1H), 6.44 (t, *J* = 2.7 Hz, 1H), 6.02 – 5.89 (m, 1H), 5.83 (ddd, *J* = 15.9, 4.4, 0.9 Hz, 1H), 4.28 – 4.11 (m, 2H), 2.74 – 2.55 (m, 1H), 2.56 – 2.31 (m, 3H), 2.17 – 2.02 (m, 1H), 1.96 – 1.78 (m, 1H), 1.77 – 1.63 (m, 2H), 1.51 – 1.42 (m, 2H), 1.40 – 1.15 (m, 7H), 0.92 (dd, *J* = 7.1, 3.9 Hz, 3H). **^{13}C NMR** (101 MHz, $CDCl_3$) δ 175.20,

175.13, 167.37, 167.34, 144.88, 139.57, 139.54, 138.28, 138.22, 119.68, 119.59, 77.17, 77.03, 60.59, 60.54, 46.48, 46.04, 31.95, 31.92, 31.84, 31.82, 31.57, 31.52, 31.41, 31.33, 24.62, 24.54, 24.28, 24.06, 21.87, 21.69, 14.49, 14.47. **HRMS** (ESI-TOF) m/z: $[M + H]^+$ Calcd. for $C_{18}H_{27}O_4$ 307.1909; Found 307.1945.

2-((E)-3-ethoxy-3-oxoprop-1-en-1-yl)cyclopent-2-en-1-yl (1R,2S,5R)-2-isopropyl-5-methylcyclohexane-1-carboxylate (79)

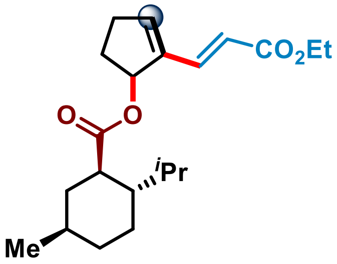

This compound was synthesized by following procedure GP3.

Physical appearance: Colorless gummy liquid.

Column material: 100-200 mesh silica.

Eluent: petroleum ether/ ethyl acetate (95/5, v/v).

Yield: 52% (d.r. 1.1:1, 18.2 mg)

^{1}H NMR (400 MHz, $CDCl_3$) δ 7.40 (d, J = 16.0 Hz, 1H), 6.49 – 6.40 (m, 1H), 5.95 (dd, J = 5.9, 3.2 Hz, 1H), 5.81 (dd, J = 16.0, 3.9 Hz, 1H), 4.39 – 4.08 (m, 2H), 2.75 – 2.56 (m, 1H), 2.55 – 2.34 (m, 2H), 2.28 (tt, J = 11.7, 3.6 Hz, 1H), 1.92 – 1.75 (m, 2H), 1.76 – 1.60 (m, 4H), 1.59 – 1.44 (m, 1H), 1.27 (t, J = 7.2 Hz, 3H), 1.24 – 1.08 (m, 1H), 1.03 – 0.81 (m, 8H), 0.76 (dd, J = 17.5, 6.9 Hz, 3H). **^{13}C NMR** (101 MHz, $CDCl_3$) δ 176.36, 176.24, 167.34, 144.98, 144.93, 139.52, 139.42, 138.22, 138.12, 119.61, 119.60, 77.16, 77.06, 60.55, 60.53, 48.25, 48.08, 44.53, 44.47, 38.94, 38.92, 34.70, 34.67, 32.28, 32.27, 31.80, 31.66, 31.57, 31.56, 29.55, 29.51, 23.85, 23.80, 22.45, 21.52, 21.49, 16.04, 15.86, 14.48. **HRMS** (ESI-TOF) m/z: $[M + H]^+$ Calcd. for $C_{21}H_{33}O_4$ 349.2379; Found 349.2389.

2-((E)-3-oxobut-1-en-1-yl)cyclopent-2-en-1-yl (2S,5R)-2-isopropyl-5-methylcyclohexane-1-carboxylate (80)

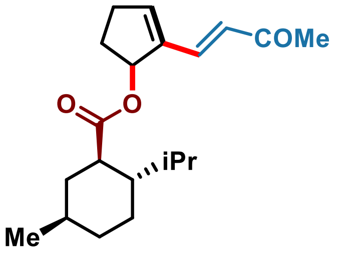

This compound was synthesized by following procedure GP3.

Physical appearance: Colorless gummy liquid.

Column material: 100-200 mesh silica.

Eluent: petroleum ether/ ethyl acetate (95/5, v/v).

Yield: 46% (d.r. 1.2:1, 14.7 mg).

^{1}H NMR (500 MHz, $CDCl_3$) δ 7.26 (d, *J* = 16.0 Hz, 1H), 6.54 – 6.51 (m, 1H), 6.11 (dd, *J* = 16.2, 6.0 Hz, 1H), 6.03 – 5.84 (m, 1H), 2.75 – 2.61 (m, 1H), 2.53 – 2.37 (m, 2H), 2.34 – 2.20 (m, 4H), 1.95 – 1.75 (m, 2H), 1.75 – 1.58 (m, 4H), 1.56 – 1.45 (m, 1H), 1.20 – 1.11 (m, 1H), 1.04 – 0.85 (m, 8H), 0.76 (dd, *J* = 22.3, 6.9 Hz, 3H). **^{13}C NMR** (101 MHz, $CDCl_3$) δ 198.57, 198.43, 176.29, 176.27, 146.25, 146.19, 139.84, 139.73, 136.75, 136.62, 127.93, 127.81, 77.44, 48.15, 48.10, 44.52, 44.43, 39.05, 38.99, 34.68, 34.66, 32.29, 32.26, 31.81, 31.73, 31.69, 29.56, 28.27, 28.20, 23.86, 23.83, 22.48, 21.52, 16.08, 15.99. **HRMS** (ESI-TOF) m/z: $[M + Na]^+$ Calcd for $C_{20}H_{30}NaO_3$ 341.2093; Found 341.2098.

Note: (From compound no. 71 to compound no. 80) These compounds are found to be slightly volatile in nature.)

2.5 Applications

a. Drug synthesis & selective late-stage olefination of OMe-protected Monobenzone in a single step

Ethyl (E)-3-(3-((4-methoxyphenoxy)methyl)phenyl)acrylate (81)

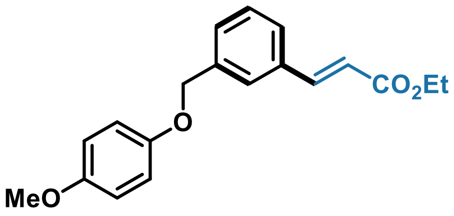

This compound was synthesized by following procedure GP1.

Physical appearance: Colorless gummy liquid.

Column material: 100-200 mesh silica.

Eluent: petroleum ether/ ethyl acetate (97/3, v/v).

Yield: 72% (22.5 mg).

^{1}H NMR (500 MHz, $CDCl_3$) δ 7.69 (d, *J* = 16.0 Hz, 1H), 7.59 (s, 1H), 7.51 – 7.35 (m, 3H), 6.91 (d, *J* = 9.1 Hz, 2H), 6.84 (d, *J* = 9.1 Hz, 2H), 6.46 (d, *J* = 16.0 Hz, 1H), 5.03 (s, 2H), 4.27 (q, *J* = 7.1 Hz, 2H), 3.77 (s, 3H), 1.34 (t, *J* = 7.1 Hz, 3H). **^{13}C NMR** (126 MHz, $CDCl_3$) δ 167.15, 154.34, 152.96, 144.50, 138.38, 135.03, 129.36, 129.32, 127.73, 127.14, 118.91,

116.10, 114.93, 70.52, 60.76, 55.94, 14.53. **HRMS** (ESI-TOF) m/z: $[M + Na]^+$ Calcd. for $C_{19}H_{20}NaO_4$ 335.1259; Found 335.1267.

b. Procedure for reduction of (E)-1-(pyrrolidin-1-yl)-3-(p-tolyl)prop-2-en-1-one to prepare synthon of Triprolidine

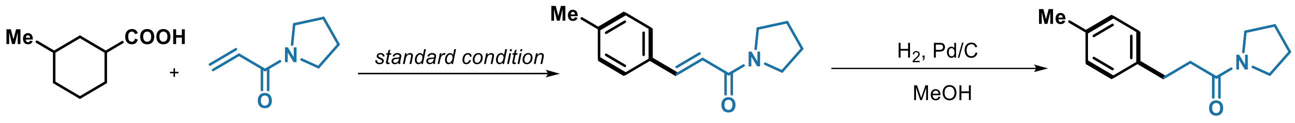

(E)-1-(pyrrolidin-1-yl)-3-(p-tolyl)prop-2-en-1-one had been synthesized with 68% yield utilizing the standard condition GP1 (in a 0.2 mmol scale) using 3-methyl cyclohexane carboxylic acid (28.4 mg) and 1-(pyrrolidin-1-yl)prop-2-en-1-one (50 mg). After that, in an oven-dried round bottom flask already charged with a magnetic stir-bar, 0.1 mmol of alkene (22 mg) dissolved in methanol and 10 wt% palladium on charcoal had been added, followed by continuous purging of H_2 gas from a balloon and stirred vigorously for 8 h. Upon completion, reaction mixture was filtered through celite and filtrate was evaporated under reduced pressure and 1-(pyrrolidin-1-yl)-3-(p-tolyl)propan-1-one had been obtained in 92% yield after flash column chromatography.

(E)-1-(pyrrolidin-1-yl)-3-(p-tolyl)prop-2-en-1-one (82)

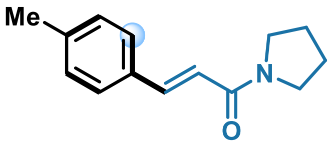

This compound was synthesized by following procedure GP1.

Physical appearance: Colorless gummy liquid.

Column material: 100-200 mesh silica.

Eluent: petroleum ether/ ethyl acetate (75/25, v/v).

Yield: 68% (29.2 mg).

^{1}H NMR (400 MHz, $CDCl_3$) δ 7.67 (d, *J* = 15.5 Hz, 1H), 7.42 (d, *J* = 8.0 Hz, 2H), 7.17 (d, *J* = 7.9 Hz, 2H), 6.68 (d, *J* = 15.5 Hz, 1H), 3.60 (dt, *J* = 13.8, 6.8 Hz, 4H), 2.36 (s, 3H), 2.05 – 1.96 (m, 2H), 1.94 – 1.83 (m, 2H). **^{13}C NMR** (101 MHz, $CDCl_3$) δ 165.14, 141.89, 139.99, 132.76, 129.67, 128.01, 117.94, 46.78, 46.23, 26.34, 24.55, 21.59. **HRMS** (ESI-TOF) m/z: $[M + Na]^+$ Calcd. for $C_{14}H_{17}NNaO$ 238.1208; Found 238.1210.

1-(pyrrolidin-1-yl)-3-(p-tolyl)propan-1-one (83)

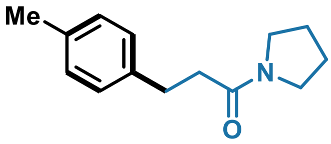

Physical appearance: colorless liquid

Column material: 100-200 mesh silica.

Eluent: petroleum ether/ ethyl acetate (70/30, v/v).

Yield: 92% (20.1 mg).

^{1}H NMR (400 MHz, $CDCl_3$) δ 7.22 – 6.83 (m, 4H), 3.46 (t, *J* = 6.7 Hz, 2H), 3.29 (t, *J* = 6.6 Hz, 2H), 2.94 (dd, *J* = 9.2, 6.7 Hz, 2H), 2.54 (dd, *J* = 9.2, 6.9 Hz, 2H), 2.31 (s, 3H), 2.00 – 1.69 (m, 4H). **^{13}C NMR** (101 MHz, $CDCl_3$) δ 171.09, 138.62, 135.74, 129.30, 128.51, 46.75, 45.84, 37.14, 30.94, 26.25, 24.58, 21.19. **HRMS** (ESI-TOF) m/z: $[M + H]^+$ Calcd for $C_{14}H_{20}NO$ 218.1545; Found 218.1553.

c. Procedure for post synthetic modification of 6-aryl substituted cyclohexene derivative

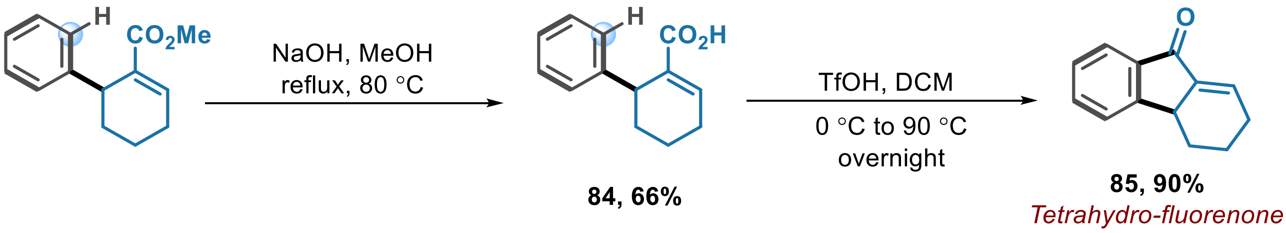

In an oven-dried round bottom flask was charged with magnetic stir-bar, methyl-1,4,5,6-tetrahydro-[1,1'-biphenyl]-2-carboxylate (0.1 mmol, 1.0 *equiv.*, 21.6 mg), sodium hydroxide (3.0 *equiv.*, 12 mg) in methanol and refluxed at 80 °C for 8 hours. Upon completion the mixture was diluted with ethyl acetate, followed by acidic work up. The organic layer was washed with brine solution two times and dried on anhydrous sodium sulfate. The solvent was evaporated under reduced pressure and the crude mixture was purified by column chromatography using silica (100-200 mesh size) and petroleum ether/ ethyl acetate as the eluent to yield corresponding cyclohexene carboxylic acid in 66%.

With the arylated cyclohexene-acid (0.05 mmol, 10.1 mg) dissolved in dry DCM, in a reaction vessel, triflic acid (5 *equiv.*, 38 mg) was added at 0 °C. The mixture was subjected to stirring for 5 minutes, followed by heating to room temperature for an additional 25 minutes. Subsequently, the mixture was maintained at a temperature of 90 °C for 12 h. After the reaction time elapsed, the resulting mixture was quenched by ice water and subjected to extractions with DCM (15 mL). The organic phase was subsequently dried using dry Na_2SO_4, filtered, and

concentrated under vacuum. Finally, the residue obtained was purified using column chromatography to yield 90% Friedel-Crafts acylated product.

1,4,5,6-tetrahydro-[1,1'-biphenyl]-2-carboxylic acid (84)

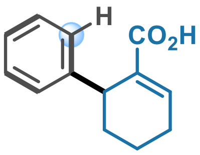

Physical appearance: colorless liquid

Column material: 100-200 mesh silica.

Eluent: petroleum ether/ ethyl acetate (90/10, v/v).

Yield: 66% (13.4 mg).

^{1}H NMR (400 MHz, $CDCl_3$) δ 7.36 (ddd, J = 4.5, 3.3, 1.0 Hz, 1H), 7.31 – 7.22 (m, 2H), 7.21 – 7.15 (m, 1H), 7.15 – 7.08 (m, 2H), 3.90 (q, J = 4.0, 3.2 Hz, 1H), 2.44 – 2.17 (m, 2H), 1.99 – 1.83 (m, 1H), 1.82 – 1.67 (m, 1H), 1.60 – 1.38 (m, 2H). **^{13}C NMR** (101 MHz, $CDCl_3$) δ 171.78, 144.85, 144.54, 131.27, 128.39, 127.96, 126.23, 39.21, 31.38, 26.30, 16.72. **HRMS** (ESI-TOF) m/z: $[M + H]^+$ Calcd for $C_{13}H_{15}O_2$ 203.1072; Found 203.1076.

2,3,4,4a-tetrahydro-9H-fluoren-9-one (85)

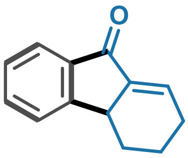

Physical appearance: colorless liquid

Column material: 100-200 mesh silica.

Eluent: petroleum ether/ ethyl acetate (90/10, v/v).

Yield: 90% (8.3 mg).

^{1}H NMR (500 MHz, $CDCl_3$) δ 7.84 (d, J = 7.6 Hz, 1H), 7.61 (t, J = 7.4 Hz, 1H), 7.53 (d, J = 7.6 Hz, 1H), 7.40 (t, J = 7.4 Hz, 1H), 6.90 (q, J = 3.3 Hz, 1H), 3.65 – 3.46 (m, 1H), 2.59 – 2.43 (m, 2H), 2.37 – 2.22 (m, 1H), 2.14 – 2.00 (m, 1H), 1.92 – 1.73 (m, 1H), 1.31 – 1.04 (m, 1H). **^{13}C NMR** (126 MHz, $CDCl_3$) δ 193.04, 153.27, 142.02, 138.39, 134.55, 134.20, 134.18, 127.77, 124.89, 124.24, 39.86, 27.04, 25.81, 22.71. **HRMS** (ESI-TOF) m/z: $[M + H]^+$ Calcd. for $C_{13}H_{13}O$ 185.0966; Found 185.0979.

d. Procedure for post synthetic modification of 5-aryl substituted cyclopentene derivative 32

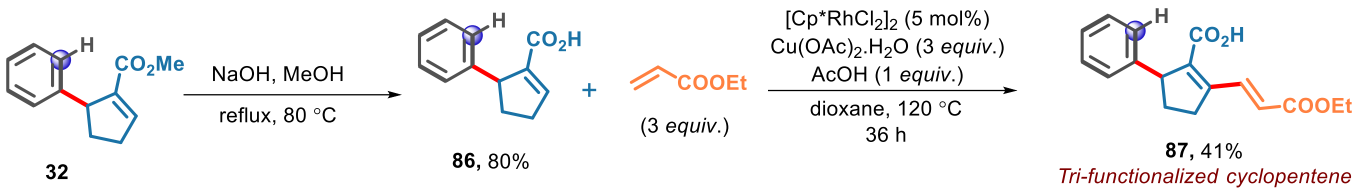

In an oven-dried round bottom flask was charged with magnetic stir-bar, methyl-5-phenylcyclopent-1-ene-1-carboxylate (0.1 mmol, 1.0 *equiv.*, 20.4 mg), sodium hydroxide (3.0 *equiv.*, 12 mg) in methanol and refluxed at 80 °C for 8 hours. Upon completion the mixture was diluted with ethyl acetate, followed by acidic work up. The organic layer was washed with brine solution two times and dried on anhydrous sodium sulfate. The solvent was evaporated under reduced pressure and the crude mixture was purified by column chromatography using silica (100-200 mesh size) and petroleum ether/ ethyl acetate as the eluent to yield corresponding cyclopentene carboxylic acid (**86**) in 80%.

With the arylated cyclopentene-acid (86, 0.05 mmol, 9.5 mg) in a reaction vessel, $[Cp^*RhCl_2]_2$ (5 mol%, 1.6 mg), $Cu(OAc)_2.H_2O$ (3.0 *equiv.*, 30 mg), acetic acid (1.0 *equiv.*, 3 mg), ethyl acrylate (3.0 *equiv.*, 16 mg) and dioxane (0.5 mL) were added.[19] The mixture was then stirred at 120 °C for 36 hours. After the reaction time elapsed, the resulting mixture was cooled down to room temperature and subjected to extractions with ethyl acetate (15 mL). The organic phase was subsequently dried using dry Na_2SO_4, filtered, and concentrated under vacuum. Finally, the residue obtained was purified using column chromatography to yield 41% desired product (87).

5-phenylcyclopent-1-ene-1-carboxylic acid (86)

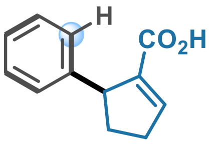

Physical appearance: Colorless liquid
Column material: 100-200 mesh silica.
Eluent: petroleum ether/ ethyl acetate (90/10, v/v).
Yield: 80% (15.1 mg).
^{1}H NMR (500 MHz, $CDCl_3$) δ 7.28 (t, J = 7.6 Hz, 2H), 7.23 – 7.05 (m, 4H), 4.45 – 3.90 (m, 1H), 2.79 – 2.62 (m, 1H), 2.60 – 2.45 (m, 2H), 2.01 – 1.81 (m, 1H). **^{13}C NMR** (126 MHz, $CDCl_3$) δ 170.02, 148.09, 144.98, 138.94, 128.67, 127.14, 126.48, 49.86, 34.42, 32.54. **HRMS** (ESI-TOF) m/z: $[M + H]^+$ Calcd. for $C_{12}H_{13}O_2$ 189.0916; Found 189.0921.

(E)-2-(3-ethoxy-3-oxoprop-1-en-1-yl)-5-phenylcyclopent-1-ene-1-carboxylic acid (87)

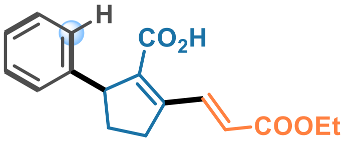

Physical appearance: colorless liquid

Column material: 100-200 mesh silica.

Eluent: petroleum ether/ ethyl acetate (90/10, v/v).

Yield: 41% (5.9 mg).

^{1}H NMR (400 MHz, $CDCl_3$) δ 8.36 (d, *J* = 16.0 Hz, 1H), 7.31 – 7.23 (m, 2H), 7.19 (t, *J* = 7.3 Hz, 1H), 7.12 (d, *J* = 7.6 Hz, 2H), 6.13 (d, *J* = 16.0 Hz, 1H), 4.26 (q, *J* = 7.1 Hz, 2H), 4.19 – 4.07 (m, 1H), 2.96 – 2.82 (m, 1H), 2.80 – 2.64 (m, 1H), 2.57 – 2.40 (m, 1H), 2.39 – 2.25 (m, 1H), 1.33 (t, J = 3.5 Hz, 3H). **^{13}C NMR** (101 MHz, $CDCl_3$) δ 168.09, 166.74, 151.31, 144.56, 138.68, 137.74, 128.86, 127.23, 126.79, 125.69, 60.98, 53.18, 33.19, 32.24, 14.28. **HRMS** (ESI-TOF) m/z: $[M + H]^+$ Calcd. for $C_{17}H_{19}O_4$ 287.1283; Found 287.1309.

e. Procedure for post synthetic modification of ethyl (E)-3-(3-(phenoxymethyl)phenyl)acrylate

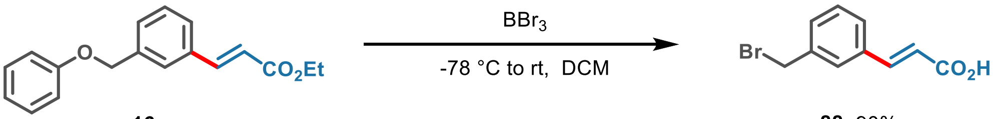

16 **88**, 90%

A solution containing ethyl (E)-3-(3-(phenoxymethyl)phenyl)acrylate (0.1 mmol, 28.3 mg) in 2 ml of dry DCM was prepared under an argon atmosphere at -78 °C. To this solution, BBr_3 (3.0 *equiv*., 75 mg) was added. The resulting mixture was stirred for 1 hour at the same temperature and then allowed to warm to room temperature, followed by stirring for 12 hours. Next, the mixture was quenched with a brine solution, and ice-cold water was added. The aqueous layer was extracted twice using CH_2Cl_2. The combined organic extract was dried with Na_2SO_4 and concentrated under reduced pressure. The crude product obtained was further purified through column chromatography, resulting in a 90% yield of the *meta*-olefinated benzyl bromide derivative.

(E)-3-(3-(bromomethyl)phenyl)acrylic acid (88)

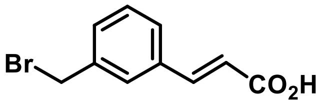

Physical appearance: colorless liquid

Column material: 100-200 mesh silica.

Eluent: petroleum ether/ ethyl acetate (70/30, v/v).

Yield: 90% (21.6 mg).

^{1}H NMR (500 MHz, $CDCl_3$) δ 7.77 (d, *J* = 16.0 Hz, 1H), 7.57 (s, 1H), 7.52 – 7.34 (m, 3H), 6.48 (d, *J* = 16.0 Hz, 1H), 4.50 (s, 2H). **^{13}C NMR** (126 MHz, $CDCl_3$) δ 171.63, 146.41, 138.91, 134.89, 131.42, 129.71, 129.03, 128.46, 118.20, 32.82. **HRMS** (ESI-TOF) m/z: $[M + Na]^+$ Calcd. for $C_{10}H_9BrNaO_2$ 262.9684; Found 262.9688.

2.6 Synthesis of probable intermediates for control experiments

a. Synthesis of ortho-olefinated-benzoic acid

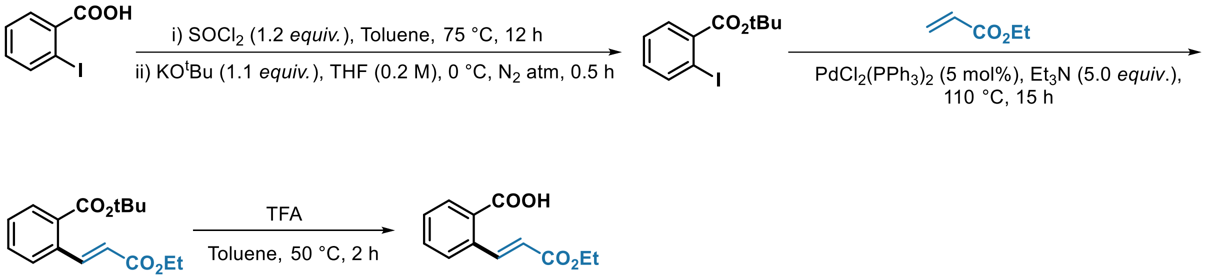

A solution of 2-iodobenzoic acid (2 mmol, 500 mg) in toluene (10 mL) was prepared. To this solution, N,N-dimethylformamide (20 µl) and thionyl chloride (2 mmol, 145 µl) were added. The reaction mixture was stirred at 75 ºC overnight. Afterwards, the reaction mixture was concentrated under vacuum, resulting in the formation of 2-iodobenzoyl chloride. This compound was then slowly added dropwise to a solution of potassium *tert*-butoxide in tetrahydrofuran (THF) at 0 ºC under a nitrogen atmosphere. The reaction mixture was kept under these conditions for 0.5 hours. The resulting mixture was then filtered through a celite pad and the solvent was removed under vacuum to obtain the product, tert-butyl 2-iodobenzoate as a colorless oil.

In the next step, a solution of $PdCl_2$ (0.1 mmol, 17 mg) and PPh_3 (0.2 mmol, 53 mg) in dry triethylamine (Et_3N) (4 mL) was prepared under a nitrogen (N_2) atmosphere. To this solution, *tert*-butyl 2-iodobenzoate and ethyl acrylate (1.1 *equiv.*, 2.2 mmol, 240 µl) were added. The reaction mixture was then stirred at 110 ºC in a sealed tube overnight. After the reaction was completed, the mixture was cooled and concentrated under vacuum. The resulting residue was subjected to purification using flash column chromatography, leading to the formation of the desired Heck coupling product as a yellow oil.

Following that, trifluoroacetic acid (0.8 mL) was added to a solution of tert-butyl 2-(3-ethoxy-3-oxoprop-1-enyl)benzoate in toluene (5 mL). The reaction mixture was then stirred at 50 °C for 2 hours. After the reaction time, the mixture was concentrated under vacuum and the resulting residue was subjected to purification using flash column chromatography. This process yielded the desired product ((E)-2-(3-ethoxy-3-oxoprop-1-en-1-yl)benzoic acid) as a colorless oil, with an overall yield of 42%.[20]

(E)-2-(3-ethoxy-3-oxoprop-1-en-1-yl)benzoic acid[20]

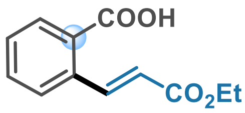

Physical appearance: colorless liquid

Column material: 100-200 mesh silica.

Eluent: petroleum ether/ ethyl acetate (90/10, v/v).

Overall yield: 42% (186 mg).

^{1}H NMR (400 MHz, $CDCl_3$) δ 8.54 (d, *J* = 15.9 Hz, 1H), 8.11 (dd, *J* = 7.8, 1.3 Hz, 1H), 7.68 – 7.54 (m, 2H), 7.48 (td, *J* = 7.4, 1.7 Hz, 1H), 6.33 (d, *J* = 15.9 Hz, 1H), 4.29 (q, *J* = 7.1 Hz, 2H), 1.35 (t, *J* = 7.0 Hz, 3H). **^{13}C NMR** (101 MHz, $CDCl_3$) δ 171.49, 166.89, 143.96, 137.48, 133.42, 131.89, 129.66, 128.79, 128.41, 121.65, 60.90, 14.51. **HRMS** (ESI-TOF) m/z: $[M + H]^+$ Calcd for $C_{12}H_{13}O_4$ 221.0814; Found 221.0821.

b. Synthesis of cyclohex-2-ene-1-carboxylic acid

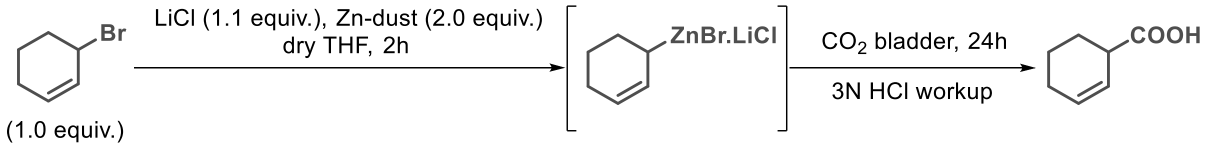

To an oven-dried, 50 mL round-bottomed flask equipped with a magnetic stir bar and a bend adapter, was charged with LiCl (466 mg, 11.0 mmol, 1.1 *equiv*.). The flask was heated to 450 °C for 10 min under high vacuum followed by the addition of zinc dust (1.31 g, 20.0 mmol, 2.0 *equiv*.) and 10 mL of THF. The adapter was replaced with septum and the flask was evacuated and refilled with argon. The activated Zn powder was then activated with 1,2-dibromoethane (0.2 µl) and TMSCl (0.4 µl). Subsequently, a solution of 3-bromocyclohexene (1.15ml, 10.0 mmol, 1.0 equiv.) was added dropwise as a solution in THF (10 mL) over a period of 15 min using a syringe pump and the mixture was stirred for additional 2 h at 23 °C. After the formation of allyl zinc bromide, the flask was evacuated and refilled with CO_2. The

reaction mixture was allowed stir for additional 24 h at r.t., while the CO_2 supply was maintained at 1 atm using a rubber bladder. Excess of activated zinc dust was filtered with a filter paper and the reaction mixture was quenched with 5 mL of 3 M aq. HCl. The aqueous layer was extracted with ethyl acetate (20 mL x 3) and combined organic layers were dried over anhydrous Na_2SO_4, filtered and concentrated. The crude product was purified by flash chromatography system to afford cyclohex-2-ene-1-carboxylic acid (570 mg, 46% yield) as a colorless liquid.[21]

Cyclohex-2-ene-1-carboxylic acid[21]

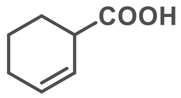

Physical appearance: colorless liquid

Column material: 100-200 mesh silica.

Eluent: petroleum ether/ ethyl acetate (90/10, v/v).

Yield: 46% (585 mg).

^{1}H NMR (500 MHz, $CDCl_3$) δ 6.02 – 5.84 (m, 1H), 5.80 – 5.71 (m, 1H), 3.23 – 3.03 (m, 1H), 2.16 – 1.97 (m, 2H), 1.99 – 1.72 (m, 3H), 1.68 – 1.51 (m, 1H). **^{13}C NMR** (101 MHz, $CDCl_3$) δ 181.06, 130.36, 123.82, 41.02, 25.31, 24.79, 20.87. **HRMS** (ESI-TOF) m/z: $[M + H]^+$ Calcd. for $C_7H_{11}O_2$ 127.0759; Found 127.0763.

3. Supplementary Discussion

3.1 Detailed mechanistic investigation

With the knowledge of dehydrogenative C–H activation in aliphatic acid from previous reports, we hypothesized multiple routes to the product conversion. The cyclohexyl carboxylic acid substrate initially binds with the Pd-catalyst with the help of bidentate MPAA ligand. Next, the alkali metal Na^+ displaces Pd(II) from κ^2 coordination with the cyclic acid to κ^1 coordination and assists in C–H activation to generate Int-A. After this intermediate formation, *β*-hydride elimination can take place. However, considering Int-A, there are two probabilities of *β*-hydride eliminations exist leading to two different intermediates (Supplementary Figure 1).

a. Probable pathways leading to product formation:

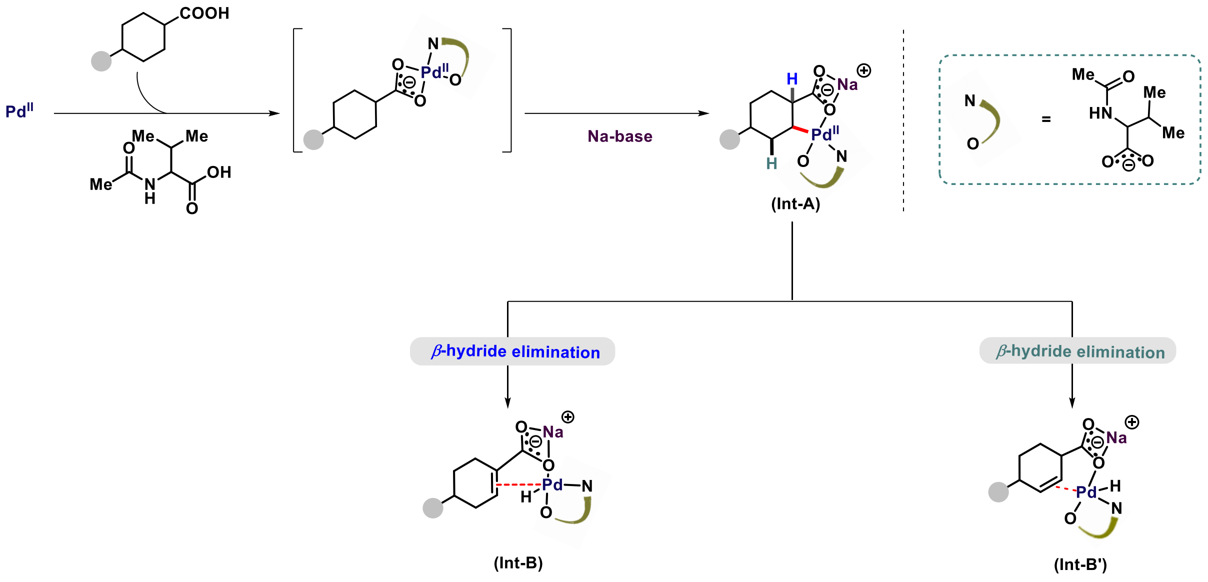

Supplementary Figure 1. Potential *β*-hydride elimination routes.

Taking consideration of (blue) *β*-hydride elimination first, as it would lead to an intermediate (Int-B) with the double bond placed in a more substituted position (hence, more stable) than the other one generated from (green) *β*-hydride elimination (Int-B'), we started our investigation.

From this intermediate (Int-B) after (blue) *β*-hydride elimination, two pathways may be followed. One pathway may follow aromatization at early stage and olefination at later stage, while other pathway may follow olefination at early stage and aromatization at later stage.

Pathway A (aromatization at early stage): From Int-B there is a chance of further allylic C–H activation, for which Pd-catalyst can activate through allylic palladation. Considering aromatization at early stages which may involve sequential allylic palladation, followed by dehydrogenation may produce Int-Ar. Int-Ar may undergo benzoic acid-directed ortho olefination to produce Int-Ar', which again may follow a metal-mediated decarboxylation pathway to generate the product. Lastly, Pd(0) regained its oxidation state with the help of aerial oxygen and silver carbonate (Supplementary Figure 2).

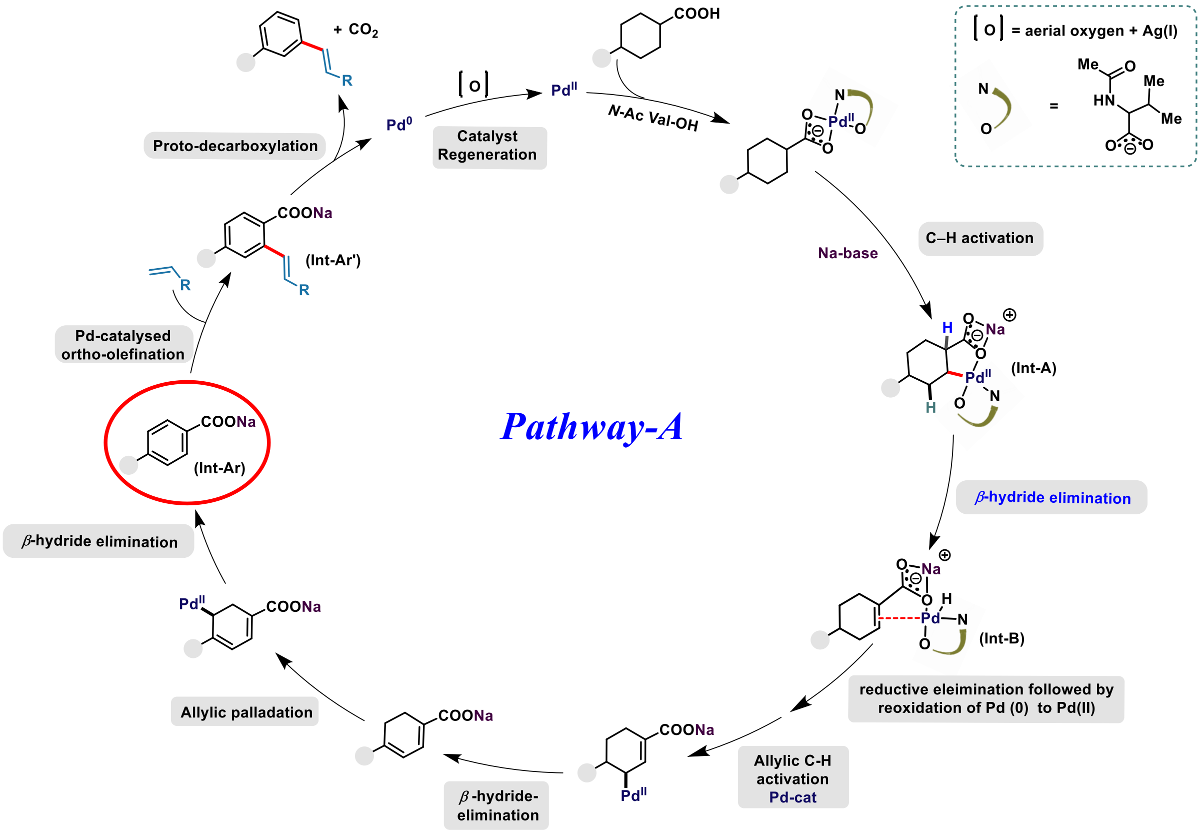

Supplementary Figure 2. Pathway-A: Aromatization precedes olefination & decarboxylation

To check the viability of this hypothesized mechanism, Int-Ar had been subjected to react under the standard condition with an olefinic coupling partner, but it failed to be converted to our desired product, rather producing phthalide derivative. Hence, benzoic acid complex is not an intermediate and the reaction does not follow pathway-A. Also, it indicated that aromatization may not precede over olefination and decarboxylation.

Control experiment - I

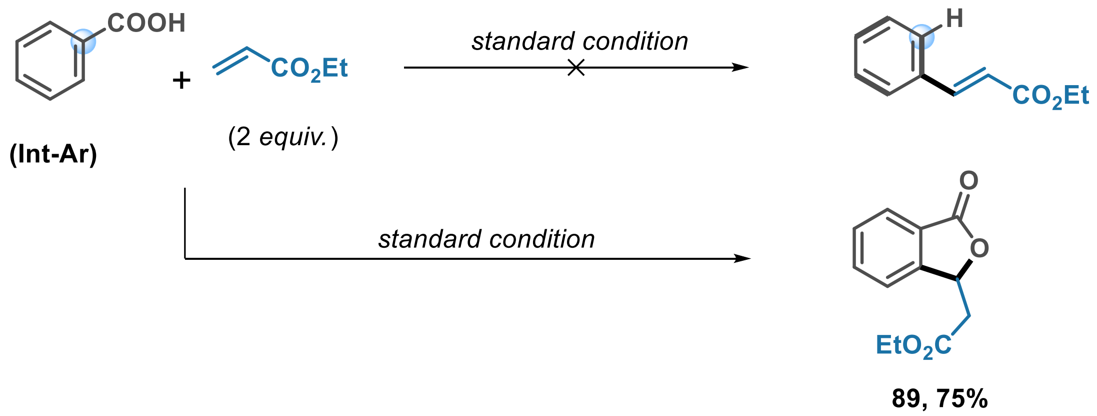

Pathway B (olefination at early stage): In our next hypothesis, we consider acid directed *β*-C(*sp*^{2})–H olefination from Int-B initially, which could lead to Int-D formation *via* another

intermediate i.e. Int-C. As similar with the previous case, Int-D may undergo multiple allylic palladation and sequential *β*-hydride eliminations to generate Int-Ar'. From Int-Ar', a metal-mediated decarboxylative pathway would give rise to the product formation (Supplementary Figure 3).

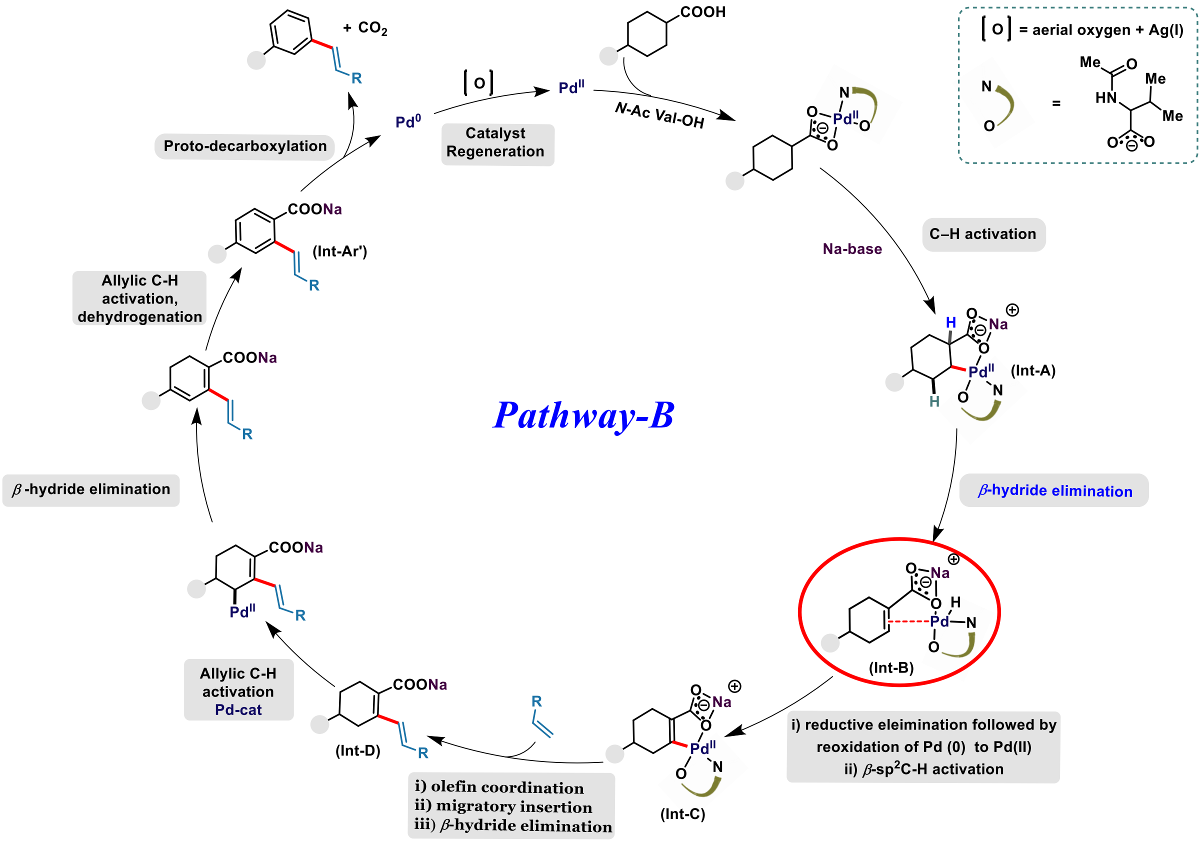

Supplementary Figure 3. Pathway-B: Olefination precedes aromatization & decarboxylation

Int-B being the common intermediate of both pathway-A and Pathway-B, we tested cyclohex-1-ene-1-carboxylic acid under our standard reaction condition with ethyl acrylate as coupling partner. However, the *α,β*-unsaturated cyclohexene carboxylic acid did not produce our desired olefinated-aryl product, rather it yielded unsaturated lactone derivative with 74% yield, which proves that cyclohex-1-ene-1-carboxylic acid is not an intermediate involved in the mechanistic cycle and the reaction does not follow pathway-B either.

Control experiment - II

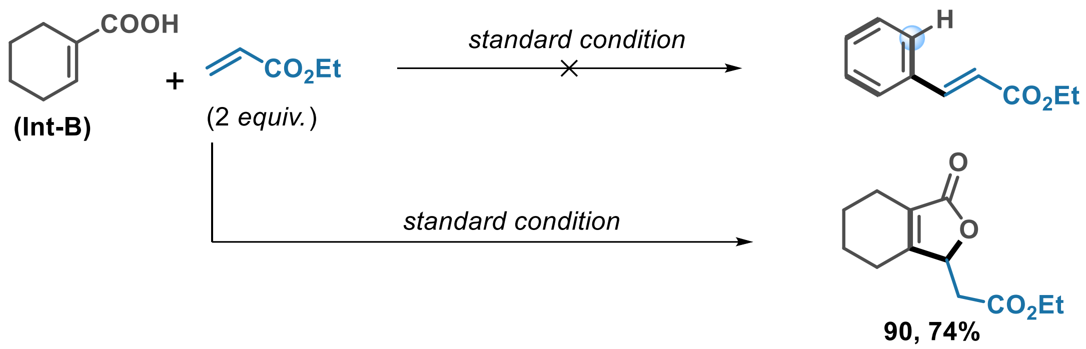

By eliminating these two mechanistic pathways, we concluded that although (blue) *β*-hydride elimination may lead to a thermodynamically stable tri-substituted alkene intermediate, the reaction may be proceeding through the elimination of alternative *β*-hydride.

Aliphatic carboxylic acid directed *β* C(sp^3)–H activation followed by (green) *β*-hydride elimination from Int-A would lead to the formation of Int-B'. Like the previous instances, two potential scenarios can be considered for this particular case as well: one pathway may follow aromatization at early stage and olefination at later stage and vice-versa.

Pathway C (aromatization at early stage): From Int-B', Pd-mediated sequential dehydrogenation may produce Int-Ar, which can undergo for further olefination and decarboxylation respectively to achieve desired product (Supplementary Figure 4).

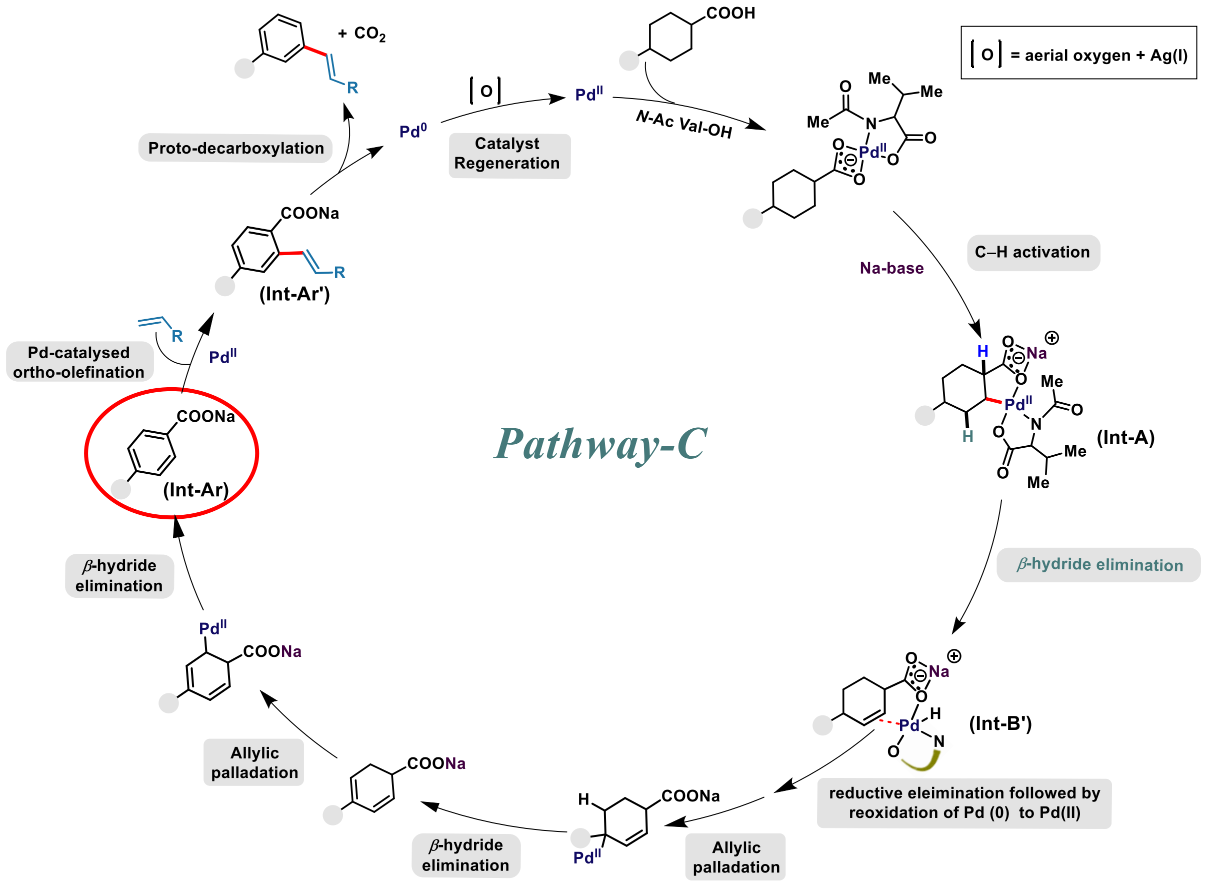

Supplementary Figure 4. Pathway-C: Aromatization precedes olefination & decarboxylation

However, from the previous controlled experiments, it had been already proved that Int-Ar leading to different product, could not be a potential intermediate and thus, Pathway-C automatically got ruled out.

Pathway D (olefination at early stage):

An alternative possibility following the formation of Int-B' may involve early-stage olefination and subsequent aromatization in the penultimate stages (Supplementary Figure 5). Similarly, we revisit the acid-directed β-C(sp^2)–H olefination starting from Int-B', which could potentially result in the formation of Int-D' through the involvement of another intermediate, Int-C'. In this scenario, Int-D' would undergo multiple allylic palladation and sequential β-hydride eliminations, ultimately generating Int-Ar'. From Int-Ar', a metal-mediated decarboxylative pathway would then facilitate the formation of the desired product.

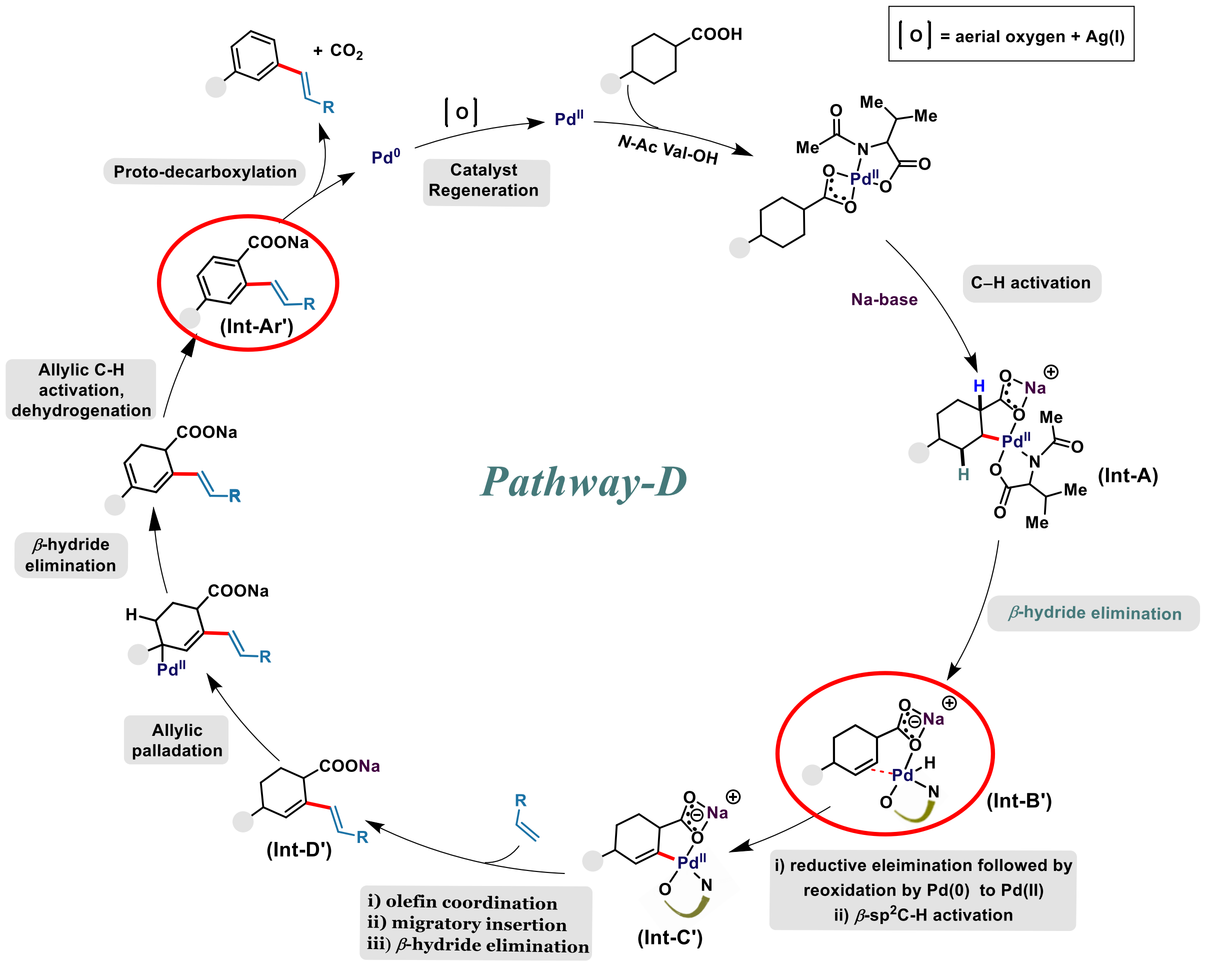

Supplementary Figure 5. Pathway-D: Olefination precedes aromatization & decarboxylation

To assess the feasibility of the suggested mechanism, we conducted experiments with the intermediates involved in this cycle. Initially, we synthesized *β,γ*-unsaturated cyclohexene carboxylic acid (Int-B') through independent means and proceeded to test it under standard conditions. To our satisfaction, it furnished the desired olefinated benzene derivative with 42% yield. This outcome strongly indicates the involvement of Int-B' in the proposed reaction pathway.

Control experiment - III

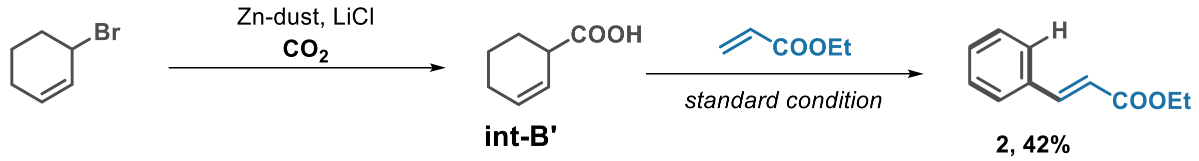

Subsequently, we synthesized another intermediate, namely ortho-olefinated benzoic acid (Int-Ar') in order to evaluate its potential role as one of the intermediates and to gain a comprehensive understanding of the complete mechanistic cycle. But upon subjecting it to our current reaction protocol it gave the benzolactone product instead of the anticipated

decarboxylated olefinated arene product. This outcome effectively eliminates the possibility of Pathway-D as well.

Control experiment - IV

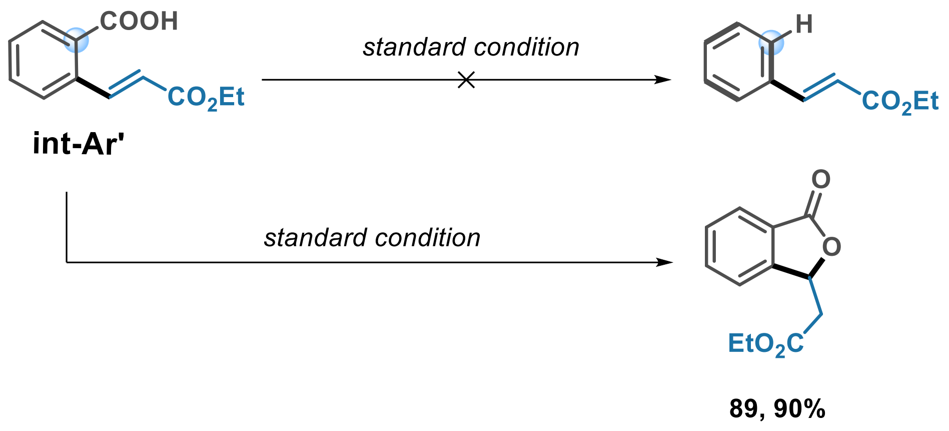

Based on the cumulative control experiments conducted, the following conclusions can be drawn: (1) Decarboxylation does not occur subsequent to the aromatization step. (2) Olefination takes place prior to the aromatization step, indicating a sequential order rather than the reverse. (3) Aromatization is likely the final phenomenon in the reaction sequence. (4) *β,γ*-unsaturated cyclohexene carboxylic acid (Int-B') most probably is involved in the mechanistic cycle through an alternative pathway.

These findings provide valuable insights into the mechanistic pathway and the sequential order of the key transformations involved.

b. Perturbing the reaction course in search of intermediates:

Interestingly, while perturbing the reaction course for 4-CF_3-cyclohexyl carboxylic acid with ethyl acrylate, under the standard condition within 20 h, we have observed two product formations. The trifluoro-methylated acid substrate with olefin provides desired aryl-derivative 14 with 36% yield and also a decarboxylated-olefinated-cyclohexene 91 with 12% yield. Expecting the decarboxylated-olefinated-cyclohexene (91) to be an intermediate, we tested the side product under our standard reaction protocol and to our delight, we found our desired product (14) formation with quantitative yield, confirming involvement of side product 91 as an intermediate of the reaction protocol. Also, traces of similar intermediates had been found in other acid substrates as well with ethyl acrylate being used as olefinic partner and isolated with desired product as inseparable mixture.

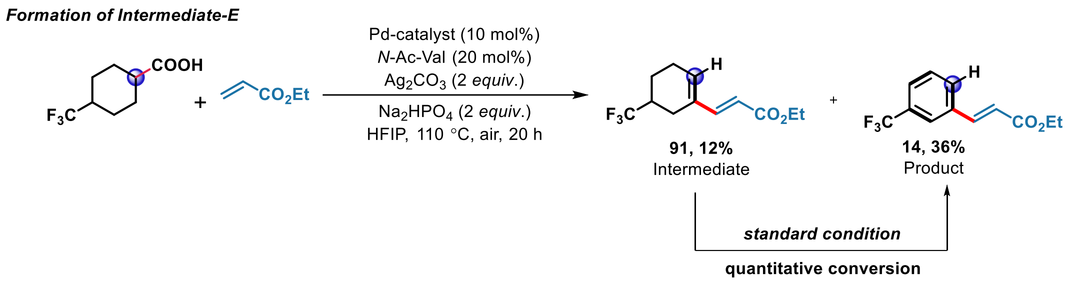

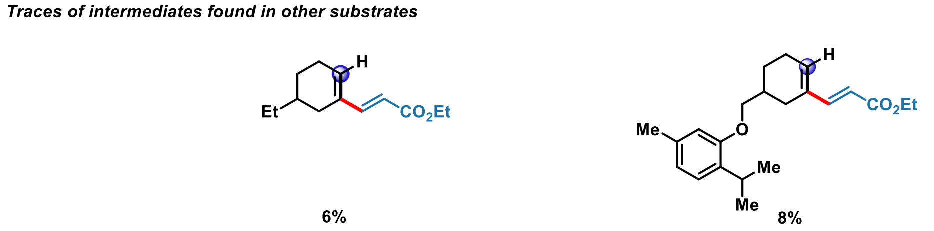

With the confirmation of the involvement of Int-B' and the identification of side product 91 as intermediate in this reaction, a general question arises regarding the mechanism by which Int-B' converts into 91 (Int-F). And so, we hypothesize two pathways: (a) Pd-catalyzed olefination at the β–C(sp2)–H centre, followed by decarboxylation, or (b) decarboxylation followed by olefination.

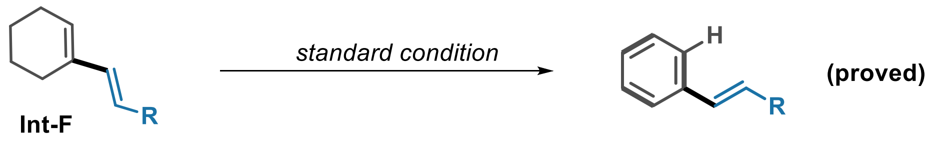

Probable pathways for *int-F* formation:

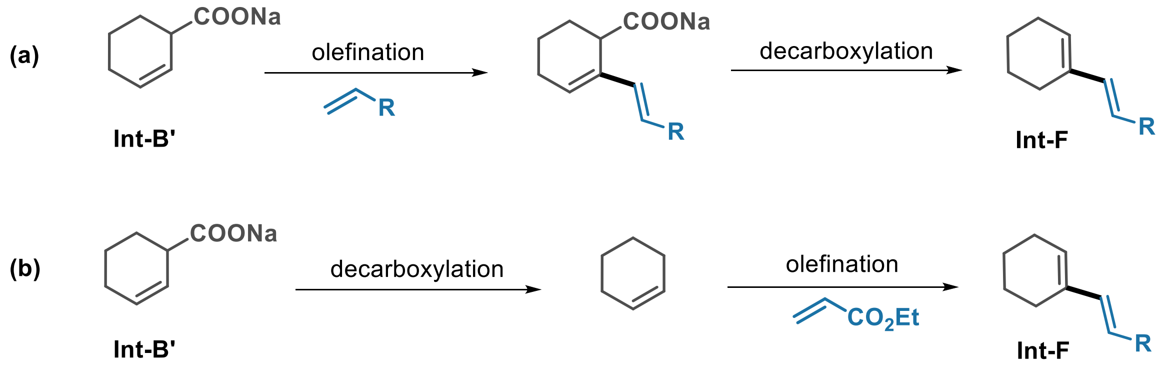

To check for a probable pathway for Int-F formation, we took cyclohexene and subjected it for olefination under the standard protocol. Failure of this reaction again confirmed that olefination was happening first and de-carboxylation is happening after olefination (pathway a). Also, from Figure 4 (Figure **4.b** from main manuscript, without coupling partner approach), the low yield of the aromatized products indicated decarboxylative aromatization became feasible after olefination took place at the β-site.

Control experiment - V

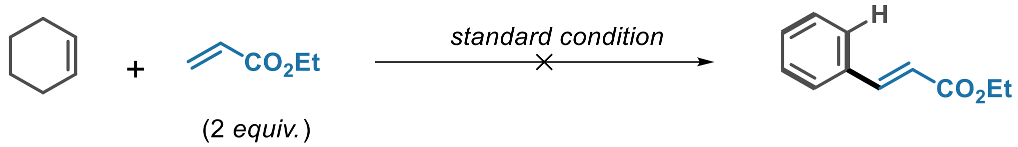

Later, while doing scope with methyl vinyl ketone, we found another side product 92 with 12% yield, the acid version of which was already considered as a potential intermediate (Int-D') for our reaction, along with the formation of 53% desired product (34) formation within 20 h. This side product formation further strengthened our hypothesis for the mechanistic cycle.

Formation of Intermediate-D'

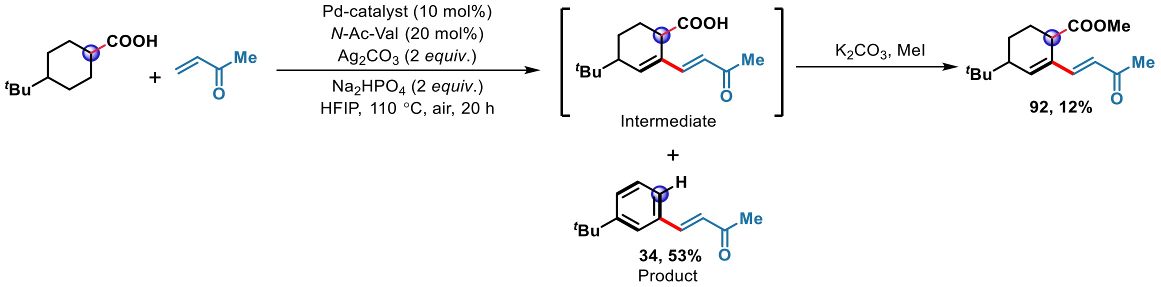

c. Plausible reaction mechanism for olefinated arene formation from cyclohexane carboxylic acid derivatives

Based on these control experiments performed and probable intermediate formations, we have proposed a plausible mechanistic cycle for our reaction (Supplementary Figure 6). The acid substrate initially binds with the catalyst with the help of bidentate ligand. Next, the alkali metal Na^+ displaces Pd(II) from κ^2 coordination to κ^1 coordination and assists in C–H activation to generate int-A. Next, β-hydride elimination from palladacycle int-A delivers β,γ-dehydrogenation product int-B'. After the regeneration of the Pd(II) species by oxidant system (aerial oxygen and Ag(I)), ligand-promoted C(sp^2)–H activation may provide vinyl palladacycle int-C'. This intermediate can be subsequently coupled with olefin through olefin coordination, migratory insertion and β-hydride elimination to generate int-D'. Int-D' may undergo subsequent proto-decarboxylation with an olefinic shift to form Int-E. Thereafter int-E, in presence of Pd(II), undergoes consecutive allylic C(sp^3)–H activations followed by sequential dehydrogenation and reductive elimination steps to deliver the olefinated benzene derivatives with re-oxidation of Pd(0) to Pd(II) by oxidant system, thereby closing the catalytic cycle.

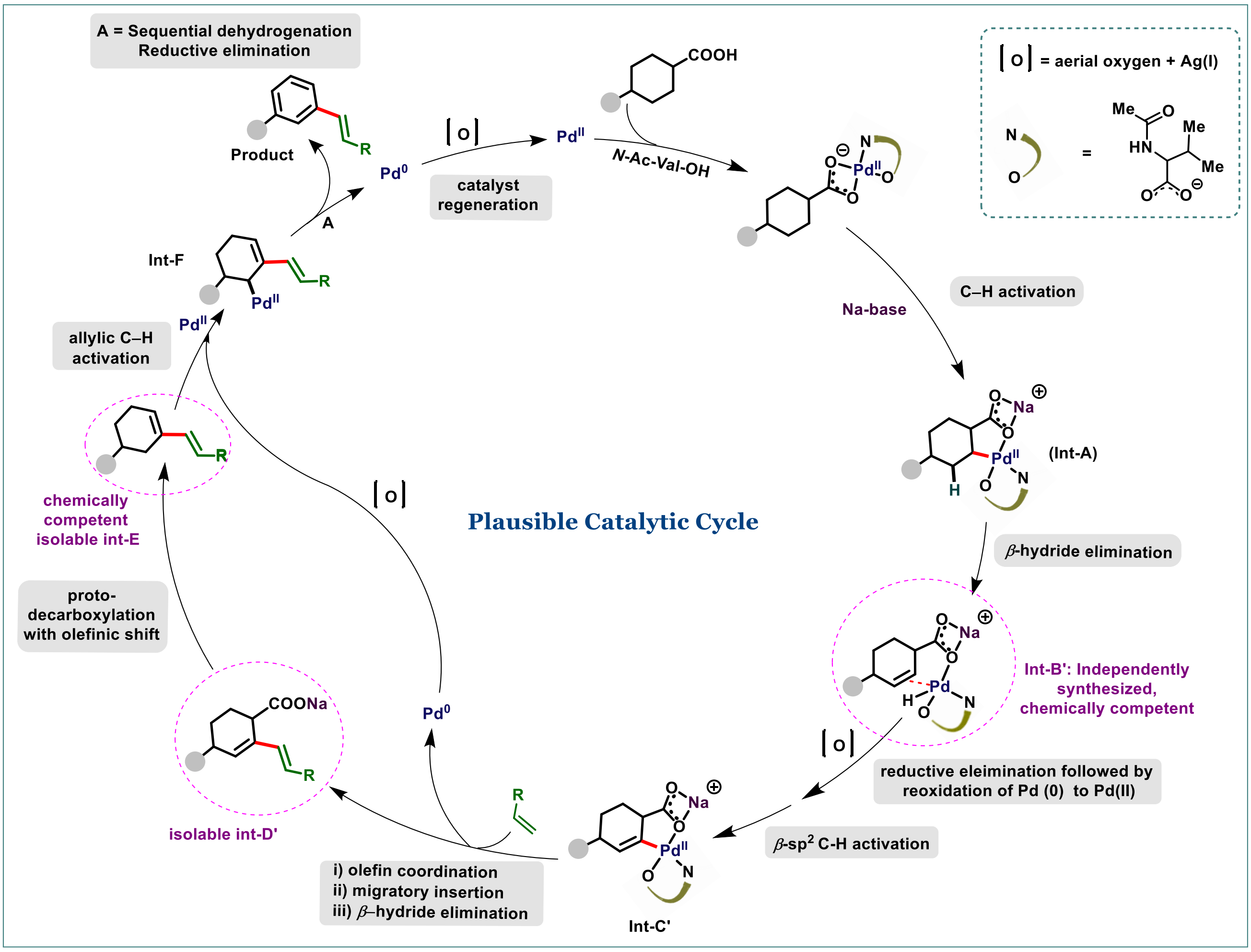

Supplementary Figure 6. Plausible catalytic cycle for olefinated arene formation

d. Investigation on site-selective dehydrogenation

However, to obtain a clear vision on the preference for *β,γ*-unsaturation than the *α,β*-unsaturation, we reasoned that the catalytic system (Pd-catalyst and the ligand involved) controlled site-selective *β*-hydride elimination. To investigate the site selectivity in dehydrogenation, we performed a couple of reactions with 4-(*tert*-butyl)cyclohexane carboxylic acid using quinoline-pyridone ligand (L_D) instead of *N*-Ac-Val-OH, keeping reaction parameters same as that of our optimized conditions.

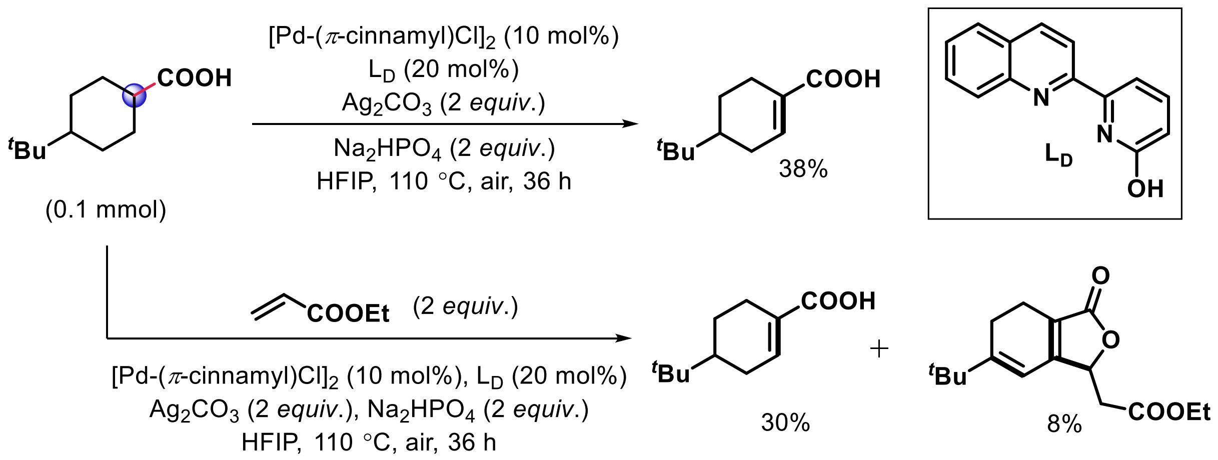

It was observed that in absence of olefin coupling partner, the substrate provided 4-(*tert*-butyl)cyclohex-1-ene-1-carboxylic acid (i.e. having *α,β*-unsaturation) in 38% yield. While in presence of ethyl acrylate, the reaction provided 30% of the *α,β*-unsaturated acid along with 8% formation of doubly dehydrogenated *γ*-lactone derivative.

Also from the literature report, it is evident that the ligand, together with the catalyst, governs selective *β*-hydride elimination.[10,22-23] This further concludes the catalytic system enabled site selective dehydrogenation of aliphatic carboxylic acid.

e. Plausible reaction mechanism for formation of difunctionalized cyclopentene derivatives

For the five-membered aliphatic carboxylic acids, the initial sequence of dehydrogenation-olefination-decarboxylation may remain same as demonstrated for six-membered carboxylic acids. After decarboxylation, palladium catalyst can be directly involved in *π*-allyl palladium complex formation via Pd-catalyzed allylic C(sp^3)−H activation and Int-F' can be generated. At this stage instead of undergoing a further *β*-hydride elimination to give the cyclopentadiene product, the catalytic system controls the dehydrogenation and follows an alternative allylic acyloxylation with another acid partner (either self-coupling or cross-coupling) to afford the difunctionalized cyclopentene derivatives (Supplementary Figure 7). However, the acyloxylation with an acid partner (4 to 6-membered cyclic acid) may take place from both the sides of *π*-allylic complex, eventually producing the same products (due to symmetric cyclopentene-*π*-allylic intermediate).

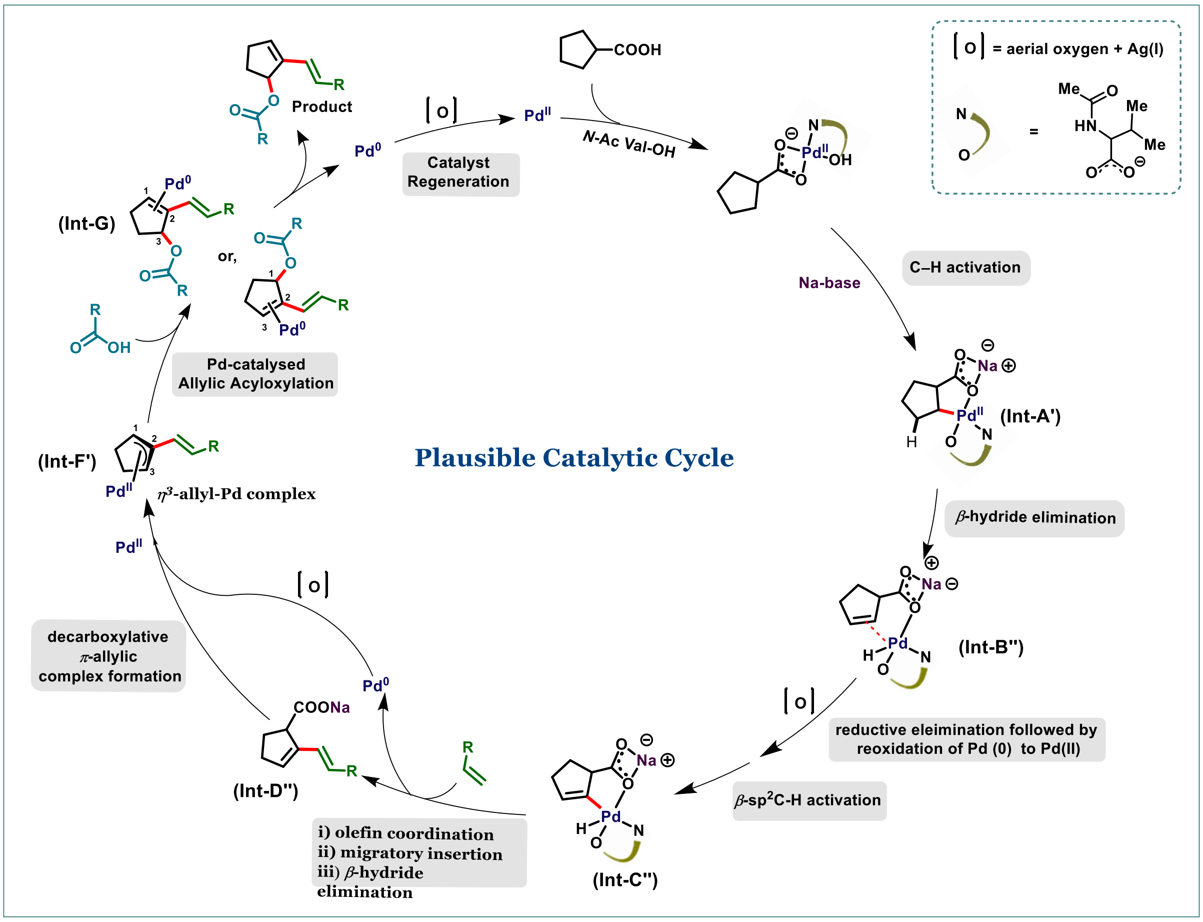

Supplementary Figure 7. Plausible catalytic cycle for difunctionalized cyclopentene formation

Ethyl (S)-2-(3-oxo-1,3-dihydroisobenzofuran-1-yl)acetate (89)

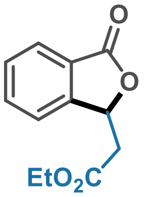

This compound was synthesized by following procedure GP1 using benzoic acid and ethyl acrylate.

Physical appearance: Colorless gummy liquid.

Column material: 100-200 mesh silica.

Eluent: petroleum ether/ ethyl acetate (85/15, v/v).

Yield: 75% (16.5 mg).

^{1}H NMR (400 MHz, $CDCl_3$) δ 7.98 – 7.81 (m, 1H), 7.67 (td, J = 7.5, 1.1 Hz, 1H), 7.57 – 7.42 (m, 2H), 5.87 (t, J = 6.5 Hz, 1H), 4.19 (q, J = 7.1 Hz, 2H), 2.88 (dd, J = 6.6, 2.1 Hz, 2H), 1.24 (t, J = 7.1 Hz, 3H). **^{13}C NMR** (101 MHz, $CDCl_3$) δ 170.07, 169.39, 148.92, 134.45, 129.70,

126.08, 125.94, 122.25, 77.17, 61.41, 39.68, 14.24. **HRMS** (ESI-TOF) m/z: $[M + H]^+$ Calcd. for $C_{12}H_{13}O_4$ 221.0814; Found 221.0813.

Ethyl (S)-2-(3-oxo-1,3,4,5,6,7-hexahydroisobenzofuran-1-yl)acetate (90)[19]

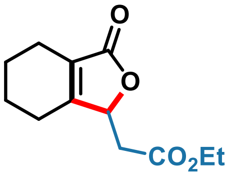

This compound was synthesized by following procedure GP1 using cyclohex-1-ene-1-carboxylic acid with ethyl acrylate.

Physical appearance: Colorless gummy liquid.

Column material: 100-200 mesh silica.

Eluent: petroleum ether/ ethyl acetate (85/15, v/v).

Yield: 74% (16.6 mg).

^{1}H NMR (500 MHz, $CDCl_3$) δ 5.28 – 5.09 (m, 1H), 4.16 (q, *J* = 7.2 Hz, 2H), 2.70 (dd, *J* = 15.9, 5.2 Hz, 1H), 2.58 (dd, *J* = 16.0, 7.6 Hz, 1H), 2.34 – 2.07 (m, 4H), 1.81 – 1.60 (m, 4H), 1.25 (t, *J* = 7.1 Hz, 3H). **^{13}C NMR** (126 MHz, $CDCl_3$) δ 173.06, 169.46, 162.74, 127.43, 79.01, 61.40, 37.75, 23.27, 21.67, 21.56, 20.06, 14.30.

Ethyl (E)-3-(5-(trifluoromethyl)cyclohex-1-en-1-yl)acrylate (91)

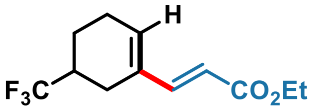

This compound was synthesized by following the procedure GP1 while perturbing the reaction time for 20 h.

Physical appearance: Colorless gummy liquid.

Column material: 100-200 mesh silica.

Eluent: petroleum ether/ ethyl acetate (97/3, v/v).

Yield: 12% (3 mg).

^{1}H NMR (400 MHz, $CDCl_3$) δ 7.31 (d, *J* = 15.8 Hz, 1H), 6.25 – 6.13 (m, 1H), 5.81 (d, *J* = 15.8 Hz, 1H), 4.21 (q, *J* = 7.1 Hz, 2H), 2.49 – 2.25 (m, 4H), 2.24 – 2.12 (m, 1H), 2.09 – 1.97 (m, 1H), 1.57 – 1.45 (m, 1H), 1.30 (t, *J* = 7.1 Hz, 3H). **^{13}C NMR** (101 MHz, $CDCl_3$) δ 167.49, 146.61, 137.24 (d, *J* = 4.0 Hz), 132.58, 127.87 (d, *J* = 279.7 Hz), 116.07, 60.56, 38.61 (d, *J* = 27.2 Hz), 25.49, 23.57 (d, *J* = 3.0 Hz), 21.13 (d, *J* = 2.0 Hz), 14.51. **^{19}F NMR** (376 MHz, CDCl3) δ -73.72 (d, *J* = 8.4 Hz). **HRMS** (ESI-TOF) m/z: $[M + H]^+$ Calcd. for $C_{12}H_{16}F_3O_2$ 249.1102; Found 249.1110.

Methyl (E)-4-(tert-butyl)-2-(3-oxobut-1-en-1-yl)cyclohex-2-ene-1-carboxylate (92)

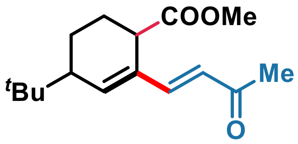

This compound was synthesized by following procedure GP1 while perturbing the reaction time for 20 h.

Physical appearance: Colorless gummy liquid.

Column material: 100-200 mesh silica.

Eluent: petroleum ether/ ethyl acetate (85/15, v/v).

Yield: 12% (3.2 mg).

^{1}H NMR (400 MHz, $CDCl_3$) δ 7.15 (d, *J* = 16.3 Hz, 1H), 6.42 (d, *J* = 2.7 Hz, 1H), 5.99 (d, *J* = 16.2 Hz, 1H), 3.67 (s, 3H), 3.32 (d, *J* = 6.2 Hz, 1H), 2.50 – 2.43 (m, 1H), 2.28 (s, 3H), 2.24 – 2.03 (m, 2H), 1.82 – 1.58 (m, 2H), 0.94 (s, 9H). **^{13}C NMR** (101 MHz, $CDCl_3$) δ 199.09, 174.19, 146.71, 144.93, 133.54, 124.94, 52.32, 47.82, 39.91, 33.69, 31.22, 27.53, 26.64, 20.41. **HRMS** (ESI-TOF) m/z: $[M + H]^+$ Calcd. for $C_{16}H_{25}O_3$ 265.1804; Found 265.1781.

4-(tert-butyl)cyclohex-1-ene-1-carboxylic acid[22]

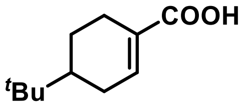

This compound was synthesized by following procedure GP2 using quinoline-pyridone ligand system.

Physical appearance: Colorless gummy liquid.

Column material: 100-200 mesh silica.

Eluent: petroleum ether/ ethyl acetate (98/2, v/v).

Yield: 38% (7 mg).

A little amount of unreacted starting material (4-(tert-butyl)cyclohexane carboxylic acid) also came as insperable mixture with the product, while performing the separation through column chromatography.

^{1}H NMR (500 MHz, $CDCl_3$) δ 7.13 (dd, *J* = 5.5, 2.7 Hz, 1H), 2.49 (dq, *J* = 15.6, 2.9 Hz, 1H), 2.34 – 2.21 (m, 1H), 2.11 (dddd, *J* = 20.1, 10.5, 5.0, 2.6 Hz, 1H), 2.00 – 1.89 (m, 2H), 1.30 – 1.23 (m, 1H), 1.14 (qd, *J* = 12.4, 5.0 Hz, 1H), 0.89 (s, 9H). Spectral data of this isolated compound matches with the literature report.[22]

3.2 NMR data of the side product observed (see, Optimization Details: Supplementary Table 1)

Ethyl-2-(6-(tert-butyl)-3-oxo-1,3,4,5-tetrahydroisobenzofuran-1-yl)acetate (side product, 1')

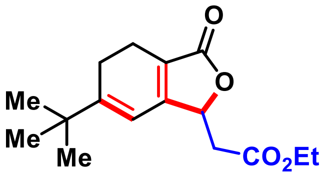

This compound was synthesized by following procedure GP1 using $Pd(OAc)_2$ as catalyst.

Physical appearance: colorless liquid

Column material: 100-200 mesh silica.

Eluent: petroleum ether/ ethyl acetate (80/20, v/v).

Yield: 16% (4.5 mg).

^{1}**H NMR** (500 MHz, $CDCl_3$) δ 5.92 (s, 1H), 5.33 (dt, *J* = 9.8, 3.6 Hz, 1H), 4.20 (q, *J* = 7.2 Hz, 2H), 2.71 (dd, *J* = 16.1, 5.3 Hz, 1H), 2.63 (dd, *J* = 16.1, 8.0 Hz, 1H), 2.46 – 2.35 (m, 4H), 1.28 (t, *J* = 7.1 Hz, 3H), 1.13 (s, 9H). ^{13}**C NMR** (126 MHz, $CDCl_3$) δ 172.44, 169.68, 161.40, 160.03, 120.79, 110.23, 77.30, 61.46, 38.31, 36.95, 28.64, 24.86, 18.21, 14.38. **HRMS** (ESI-TOF) m/z: $[M + H]^+$ Calcd. for $C_{16}H_{23}O_4$ 279.1596; Found 279.1635.

3.3 Unsuccessful substrate

Ethyl (E)-3-(5-methoxycyclohex-1-en-1-yl)acrylate (93)

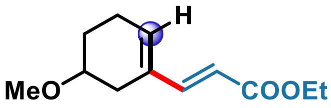

This compound was synthesized by following procedure GP1 using 4-methoxy-substituted cyclohexyl carboxylic acid.

Physical appearance: Colorless gummy liquid.

Column material: 100-200 mesh silica.

Eluent: petroleum ether/ ethyl acetate (98/2, v/v).

Yield: 28% (5.9 mg).

^{1}**H NMR** (400 MHz, $CDCl_3$) δ 7.31 (d, *J* = 15.8 Hz, 1H), 6.14 (t, *J* = 4.0 Hz, 1H), 5.78 (d, *J* = 15.8 Hz, 1H), 4.20 (q, *J* = 7.2 Hz, 2H), 3.63 – 3.48 (m, 1H), 3.39 (s, 3H), 2.55 – 2.09 (m, 5H), 1.89 (dtd, *J* = 8.8, 3.0, 1.5 Hz, 1H), 1.29 (t, *J* = 7.1 Hz, 3H). ^{13}**C NMR** (101 MHz, $CDCl_3$) δ 167.71, 147.48, 137.82, 132.69, 115.39, 75.23, 60.44, 56.14, 30.33, 26.70, 24.32, 14.53. **HRMS** (ESI-TOF) m/z: $[M + H]^+$ Calcd. for $C_{12}H_{19}O_3$ 211.1334; Found 211.1336.

2-oxabicyclo[2.2.1]heptan-3-one (94)

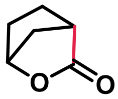

This compound was synthesized by following procedure GP1 using 3-hydroxy cyclopentane carboxylic acid and ethyl acrylate as olefin. Unfortunately, neither C–H activation nor olefination occurred; instead, an intramolecular lactonization reaction took place.

Physical appearance: Colorless gummy liquid. The compound is highly volatile.
Column material: 100-200 mesh silica.
Eluent: petroleum ether/ ethyl acetate (98/2, v/v).
Yield: 70% (7.9 mg).
^{1}H NMR (400 MHz, $CDCl_3$) δ 4.92 (d, *J* = 2.4 Hz, 1H), 2.90 (dd, *J* = 3.7, 1.6 Hz, 1H), 2.33 – 2.13 (m, 3H), 1.96 – 1.82 (m, 2H), 1.61 (dd, *J* = 10.3, 1.1 Hz, 1H). **^{13}C NMR** (126 MHz, $CDCl_3$) δ 178.60, 81.05, 42.30, 40.15, 28.45, 23.11. **HRMS** (ESI-TOF) m/z: $[M + H]^+$ Calcd. for $C_6H_9O_2$ 113.0603; Found 113.0594.

3.4 Analysis of the crude reaction mixture

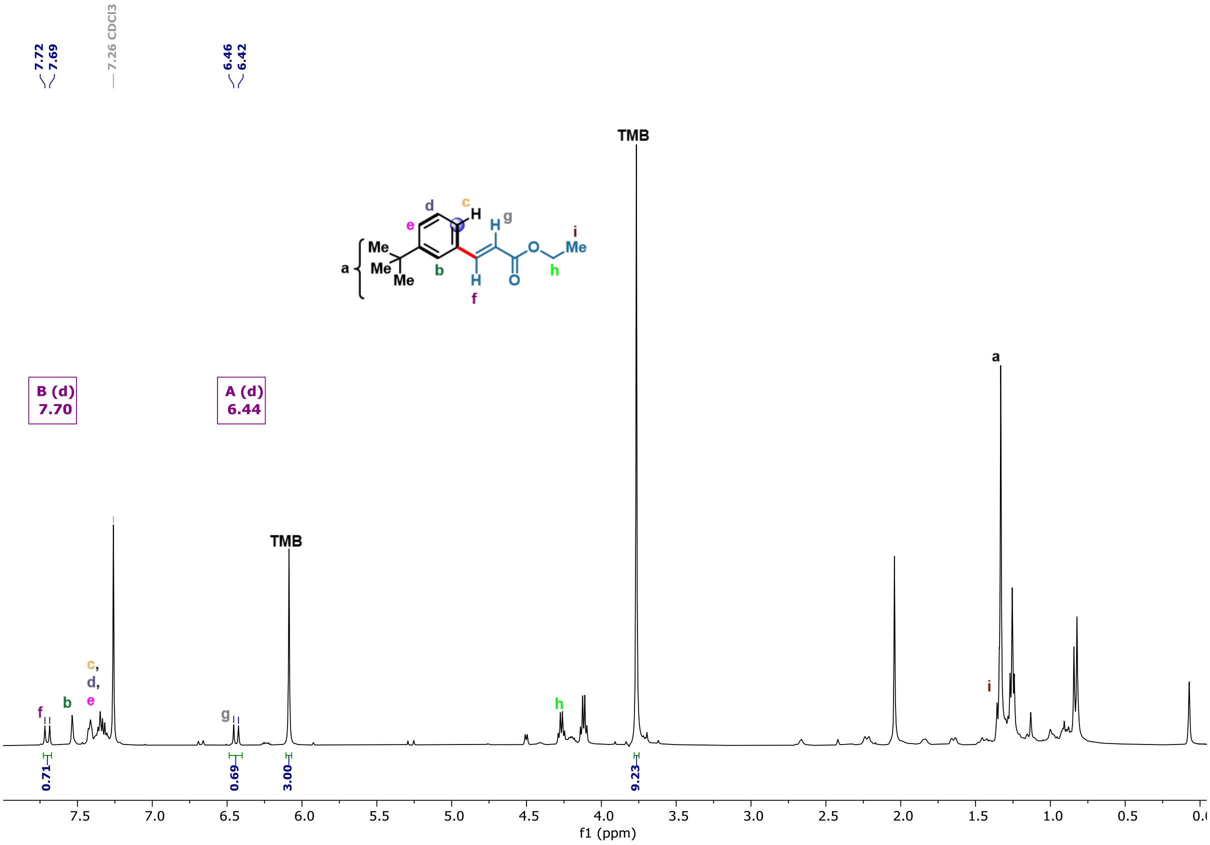

3.5 NMR Spectra of the starting materials

4-acetoxycyclohexane-1-carboxylic acid

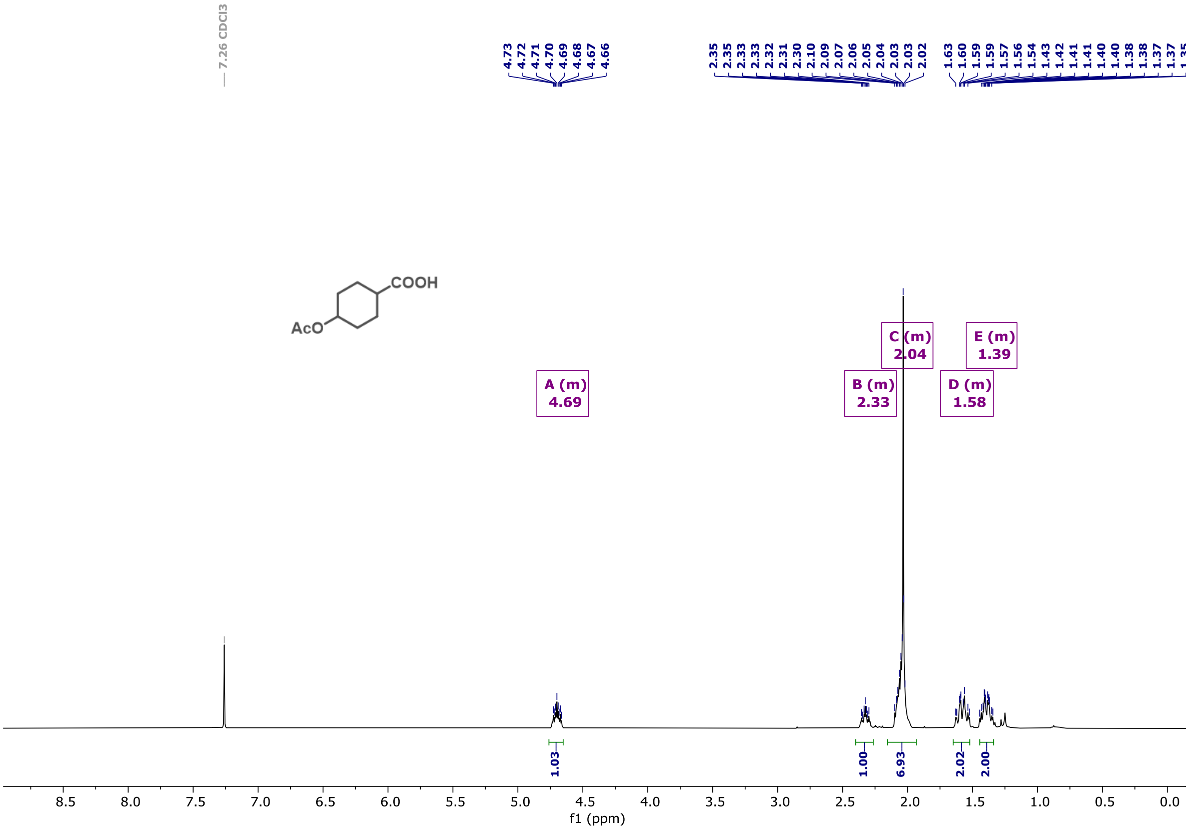

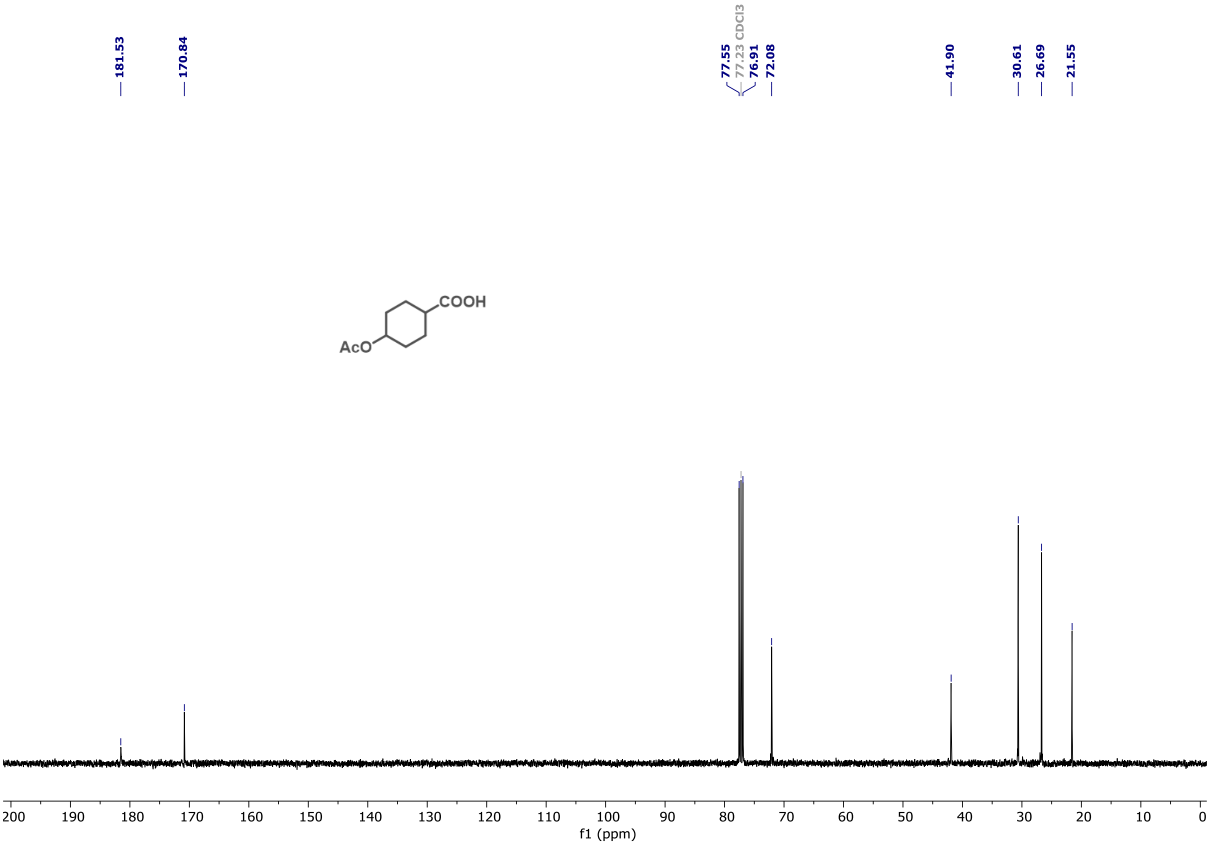

8-(p-tolyl)-1,2,3,4-tetrahydronaphthalene-1-carboxylic acid

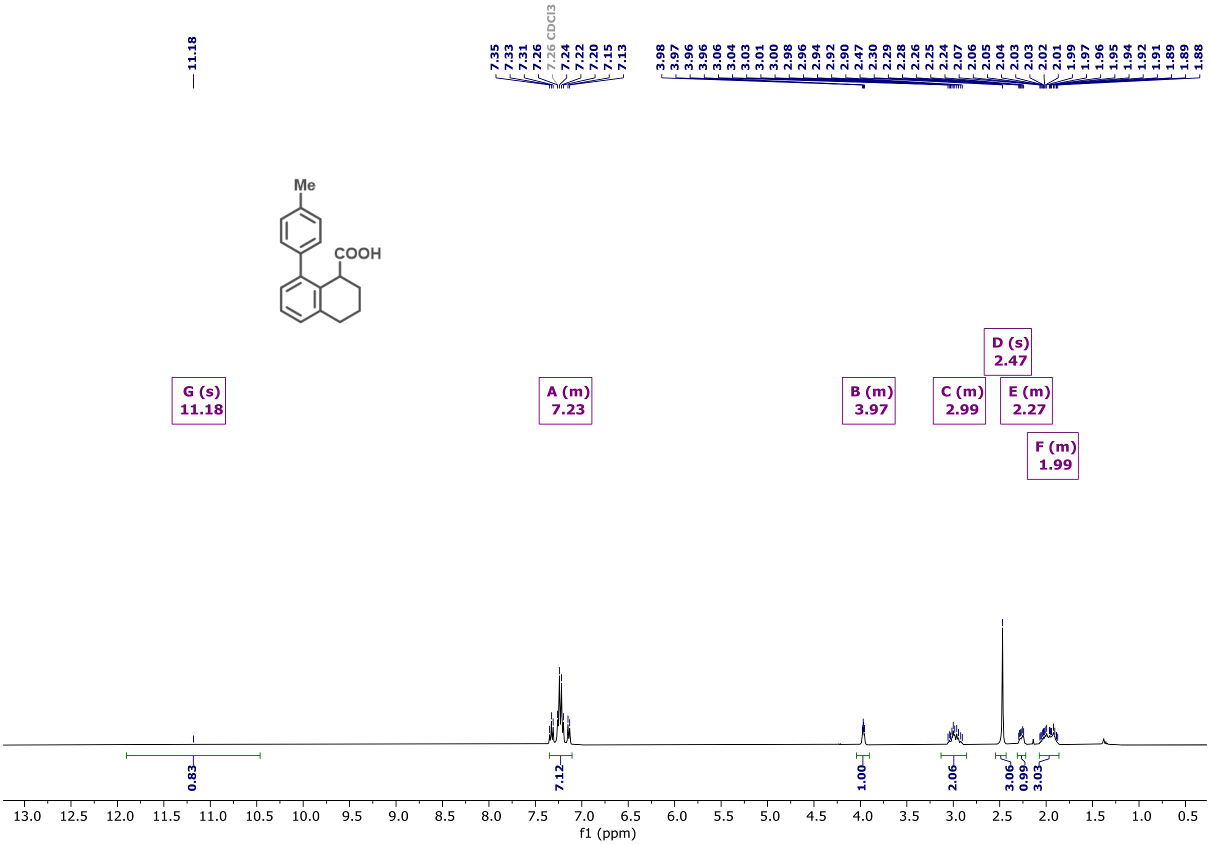

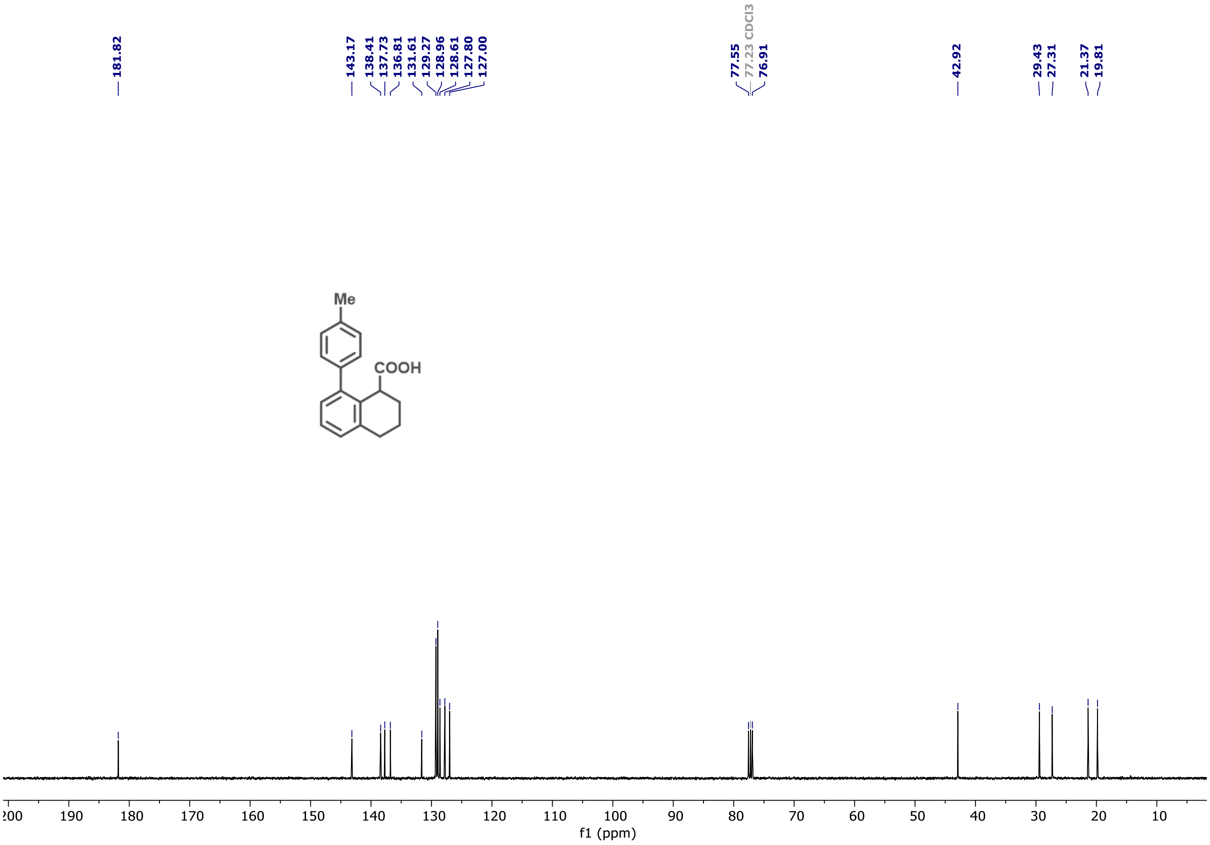

4-(phenoxymethyl)cyclohexane-1-carboxylic acid

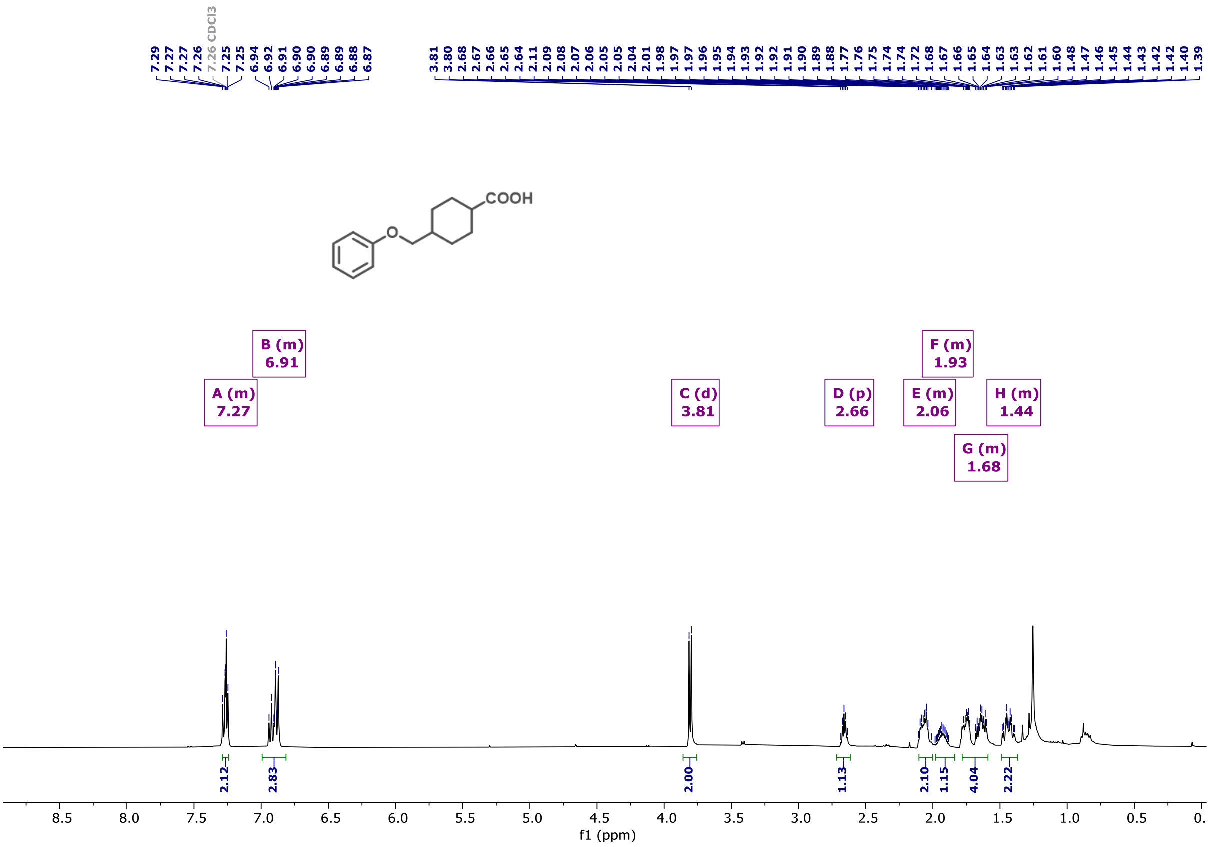

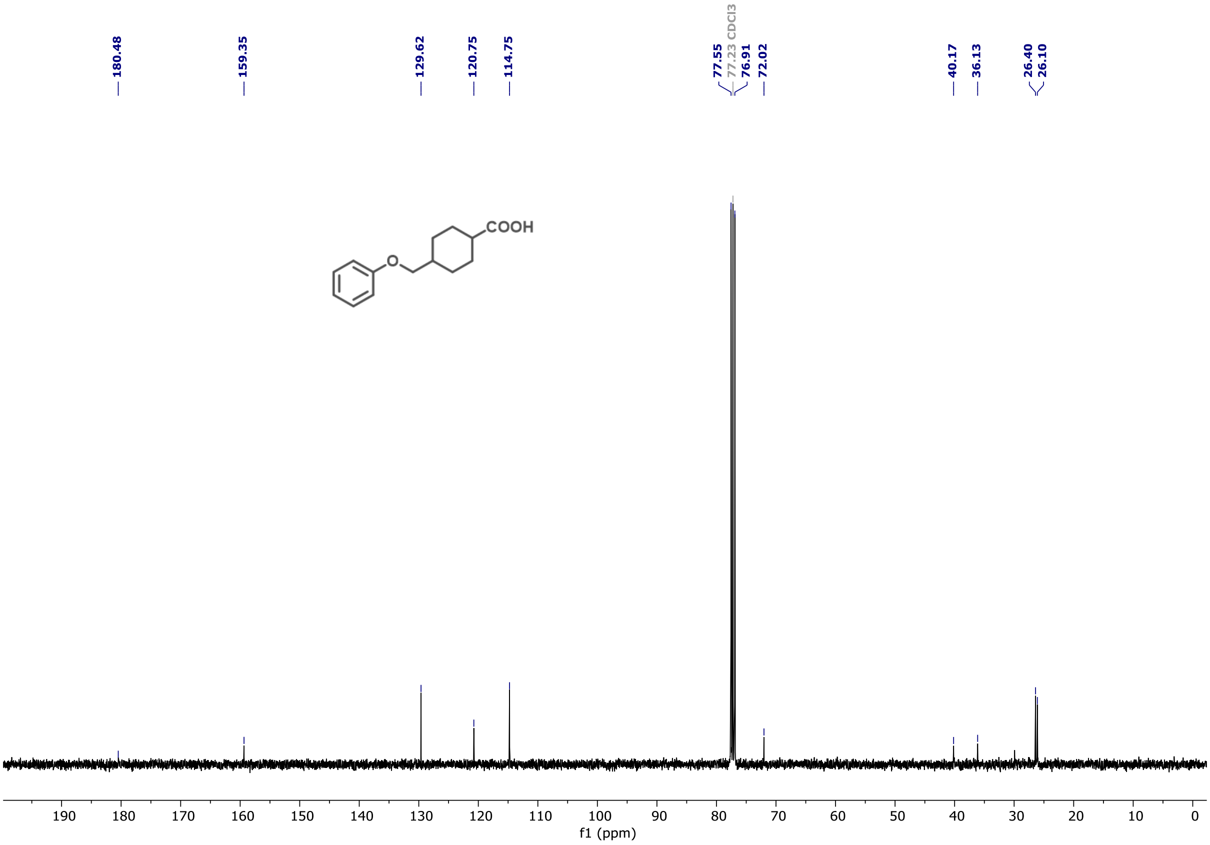

4-((2-isopropyl-5-methylphenoxy)methyl)cyclohexane-1-carboxylic acid

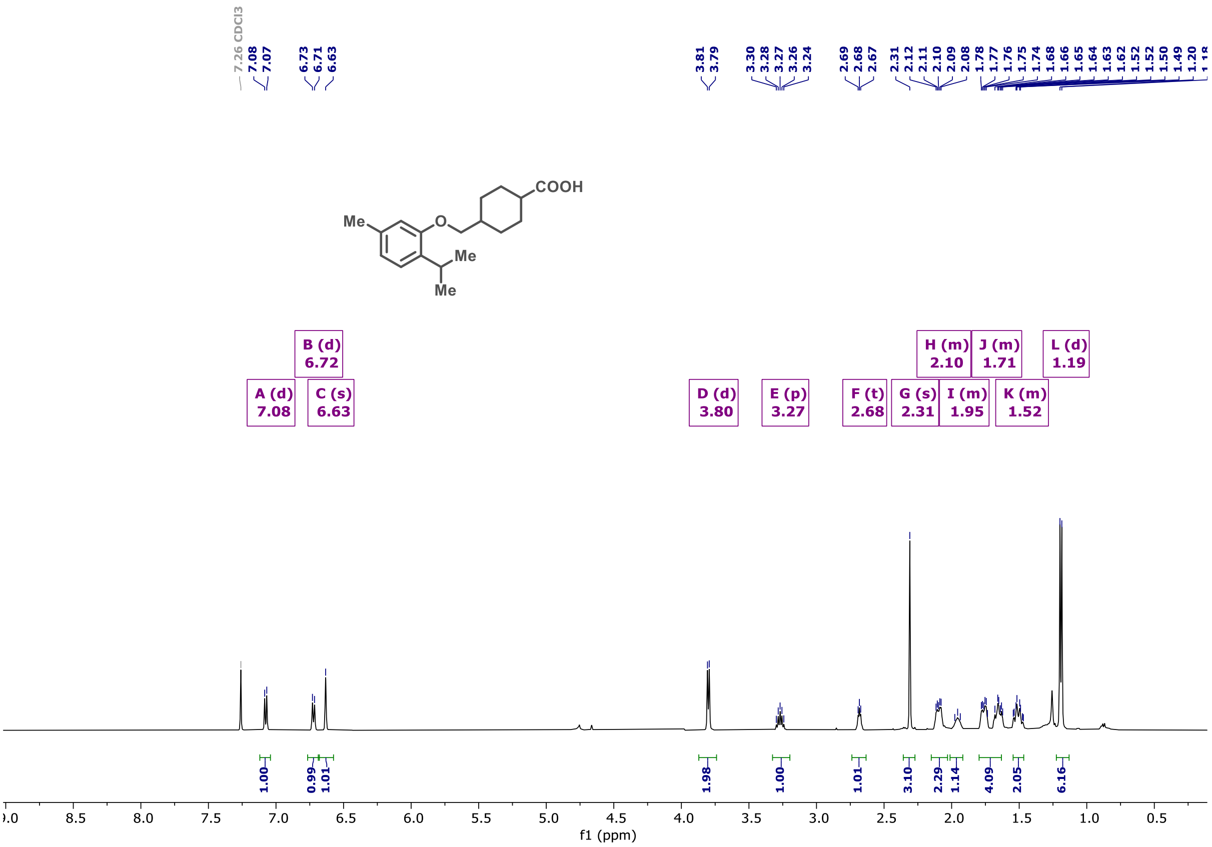

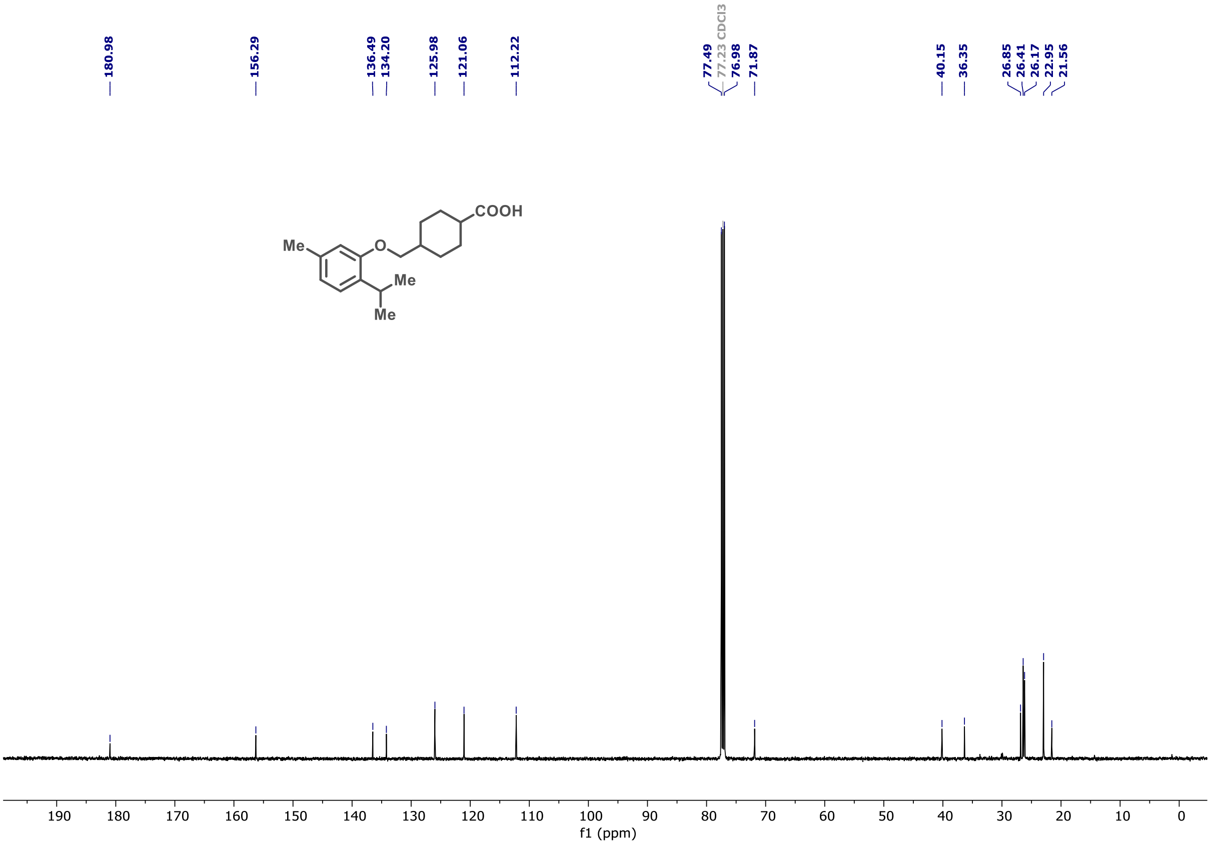

4-((5-isopropyl-2-methylphenoxy)methyl)cyclohexane-1-carboxylic acid

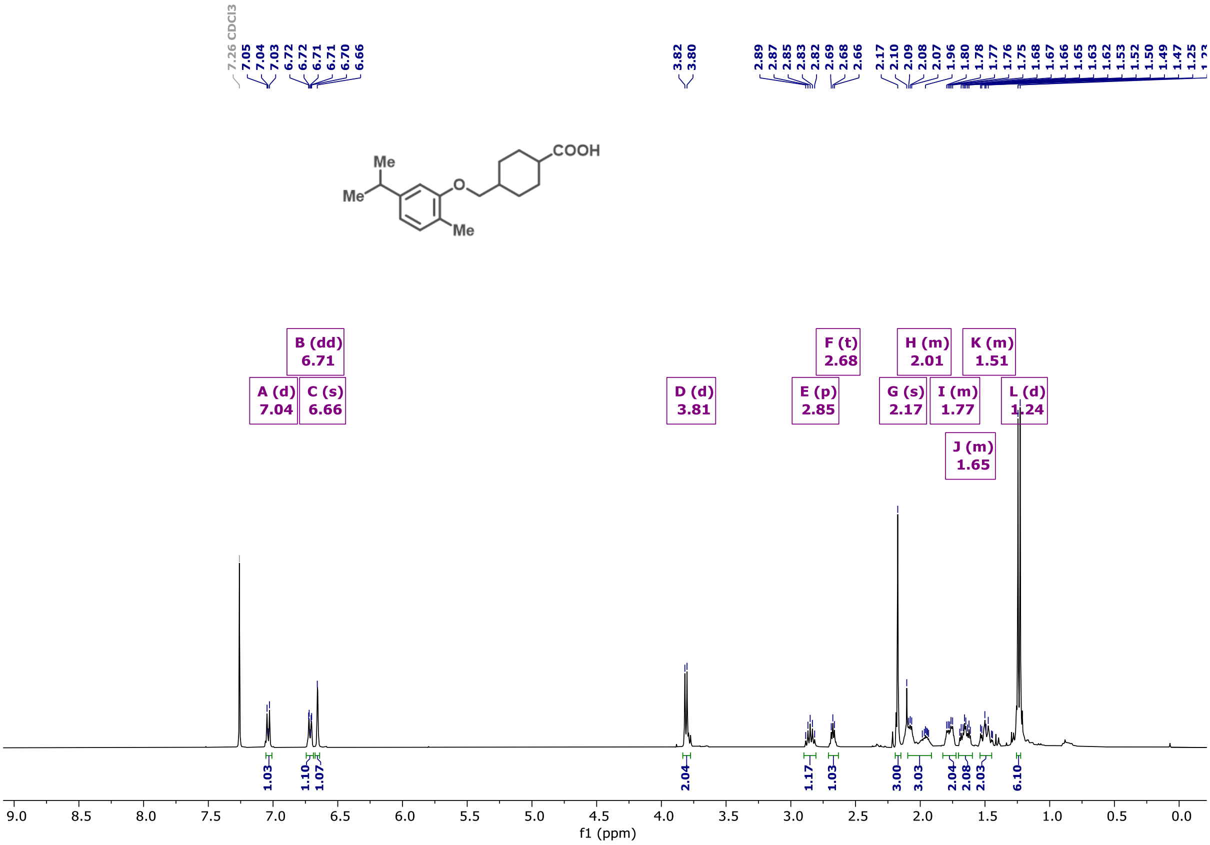

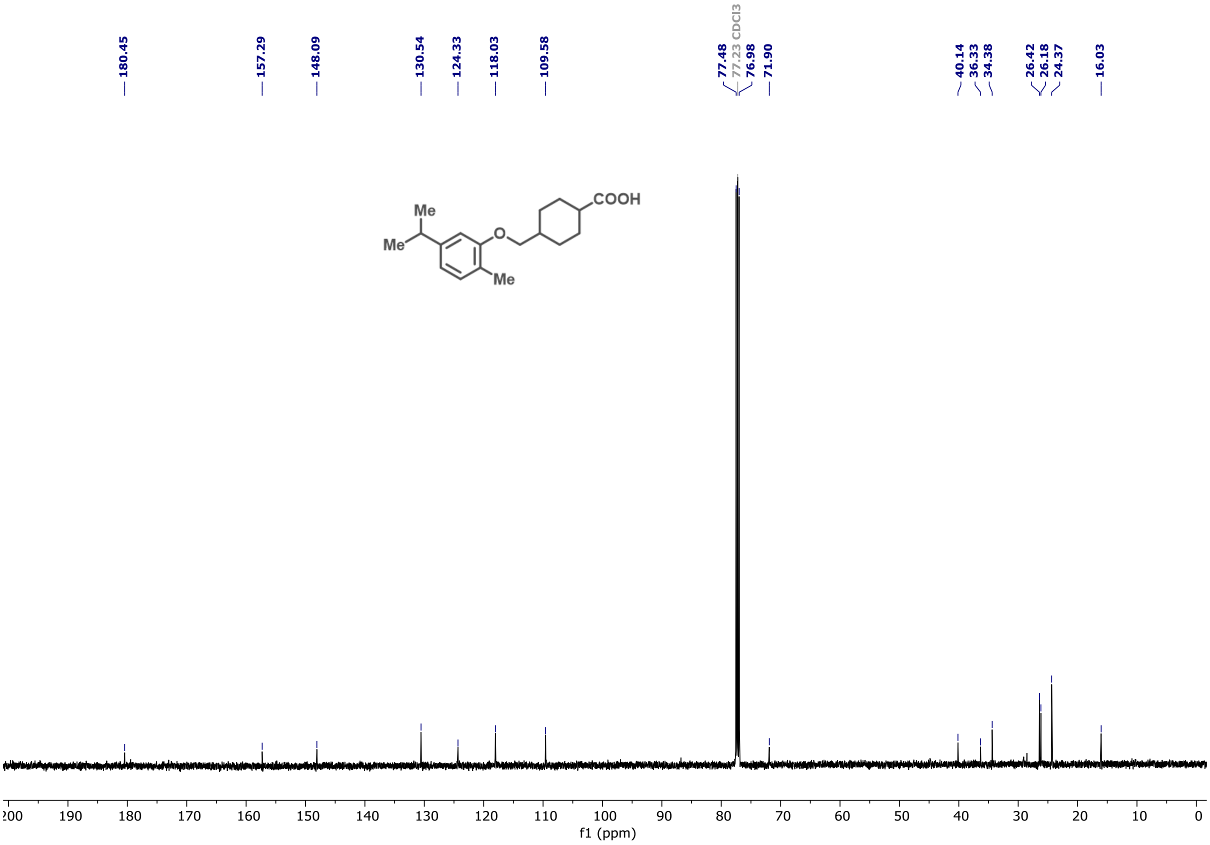

4-((4-ethylphenoxy)methyl)cyclohexane-1-carboxylic acid

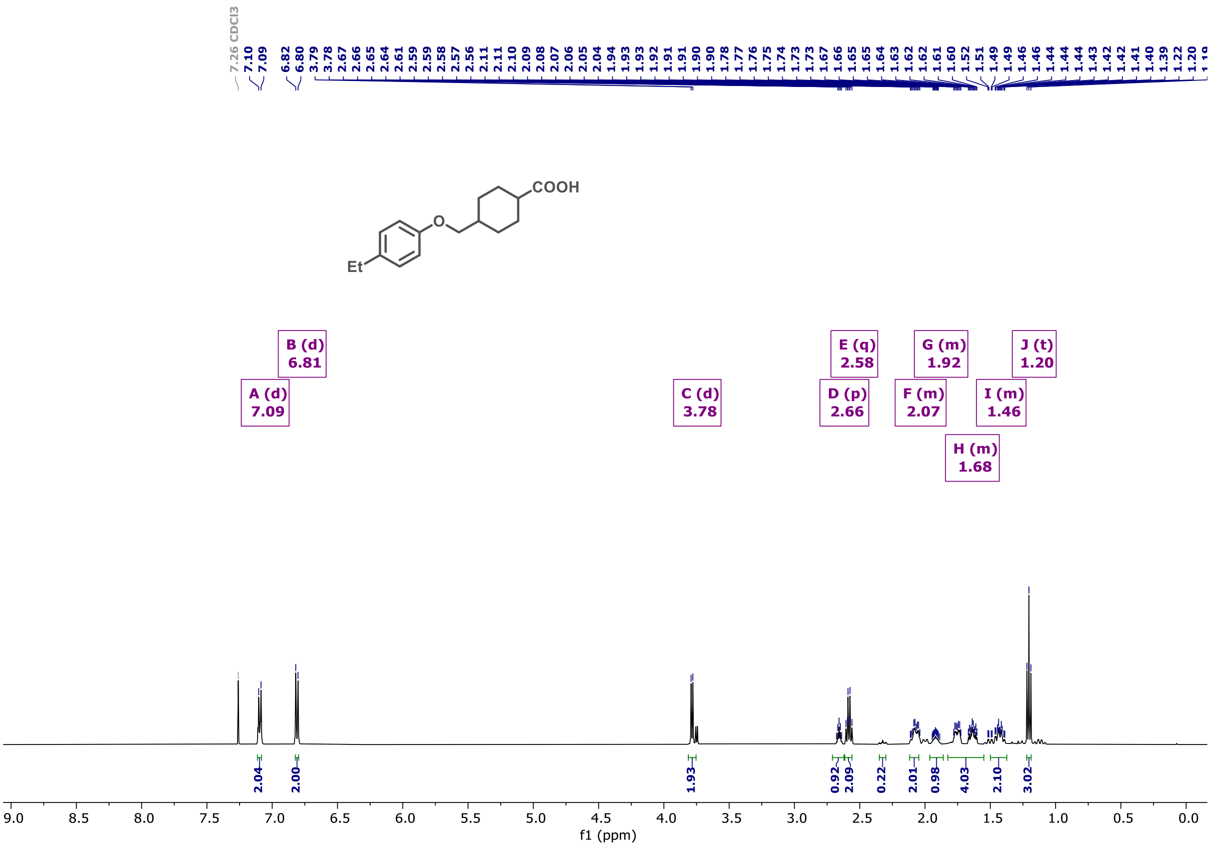

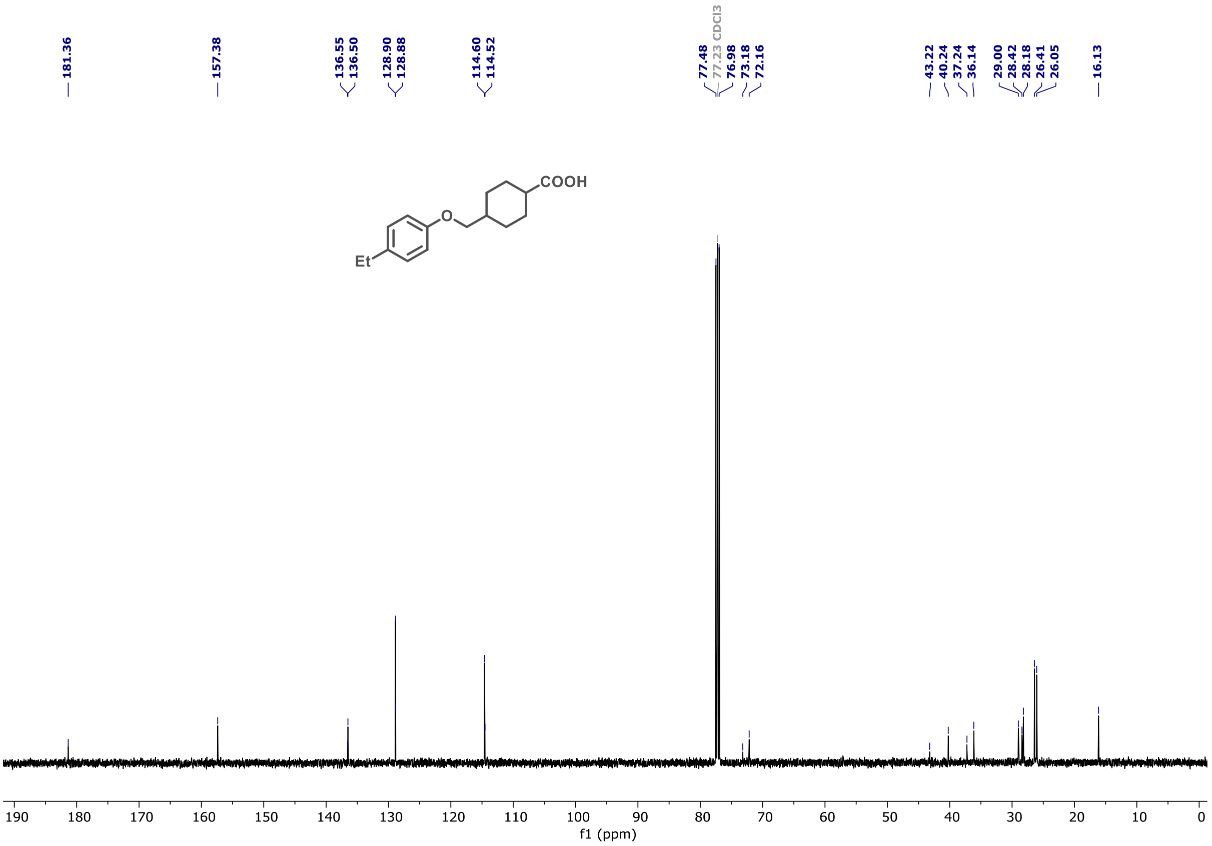

4-((2,6-diisopropylphenoxy)methyl)cyclohexane-1-carboxylic acid

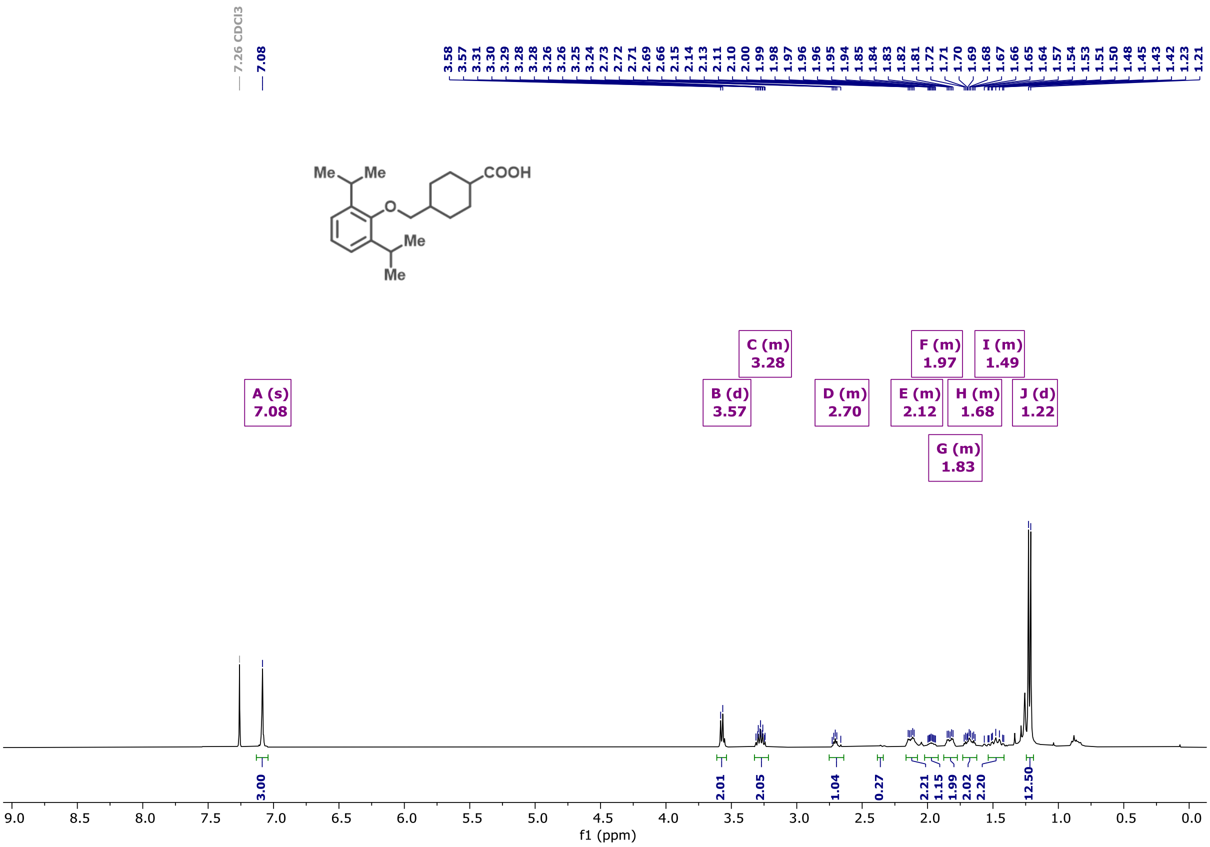

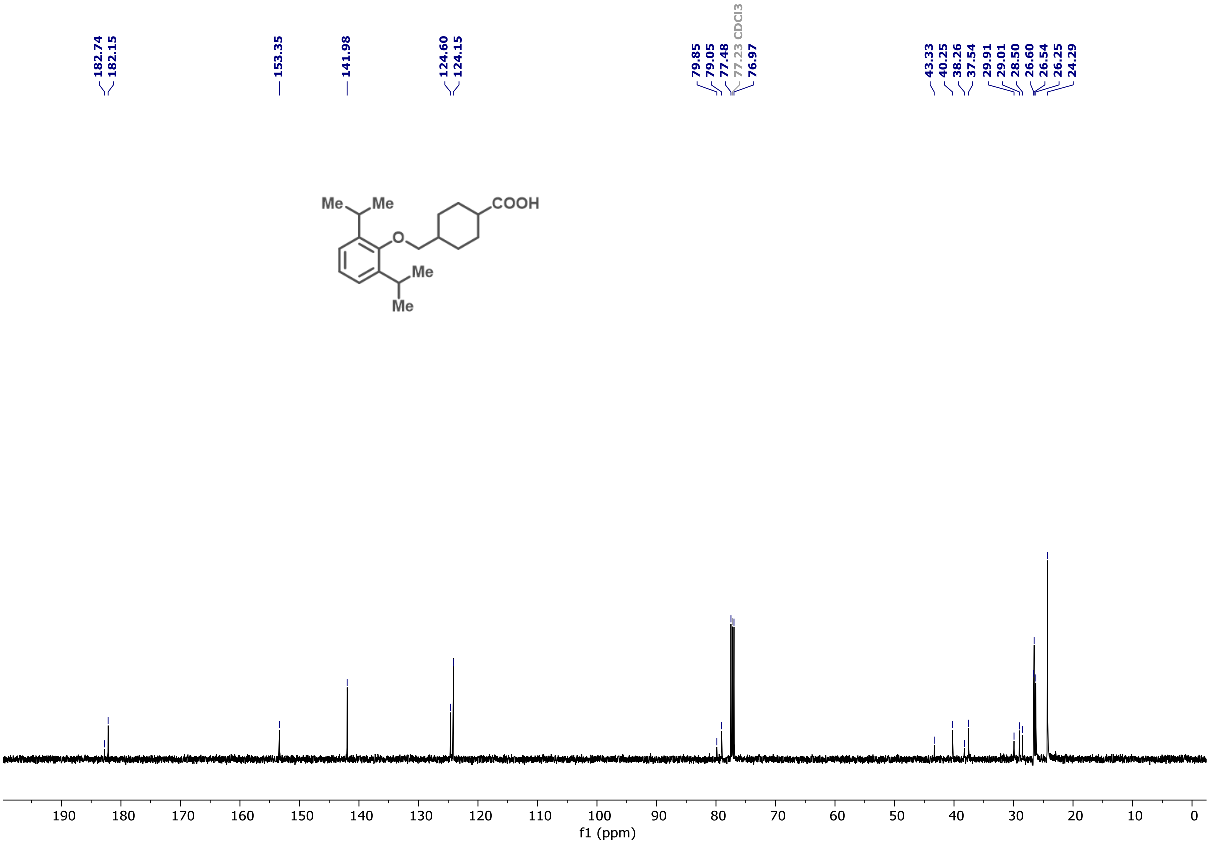

4-((4-methoxyphenoxy)methyl)cyclohexane-1-carboxylic acid

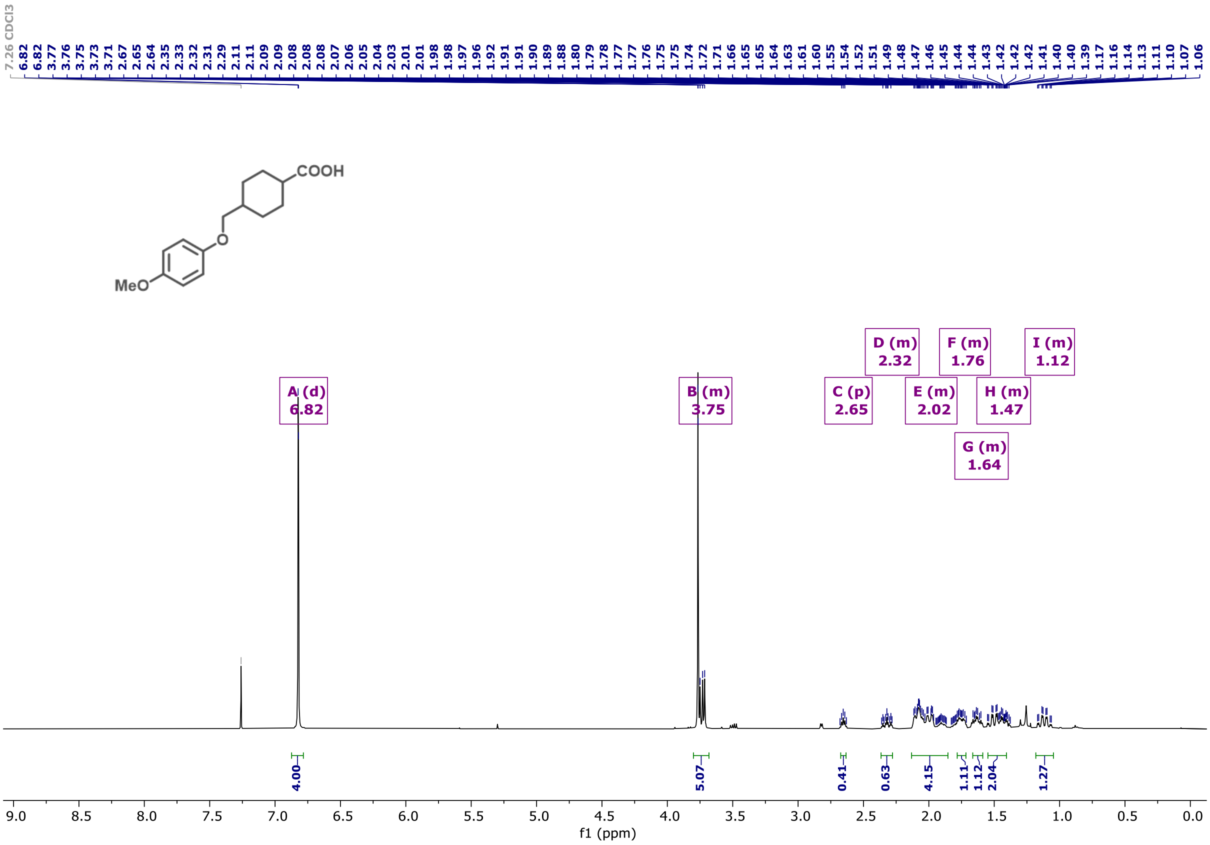

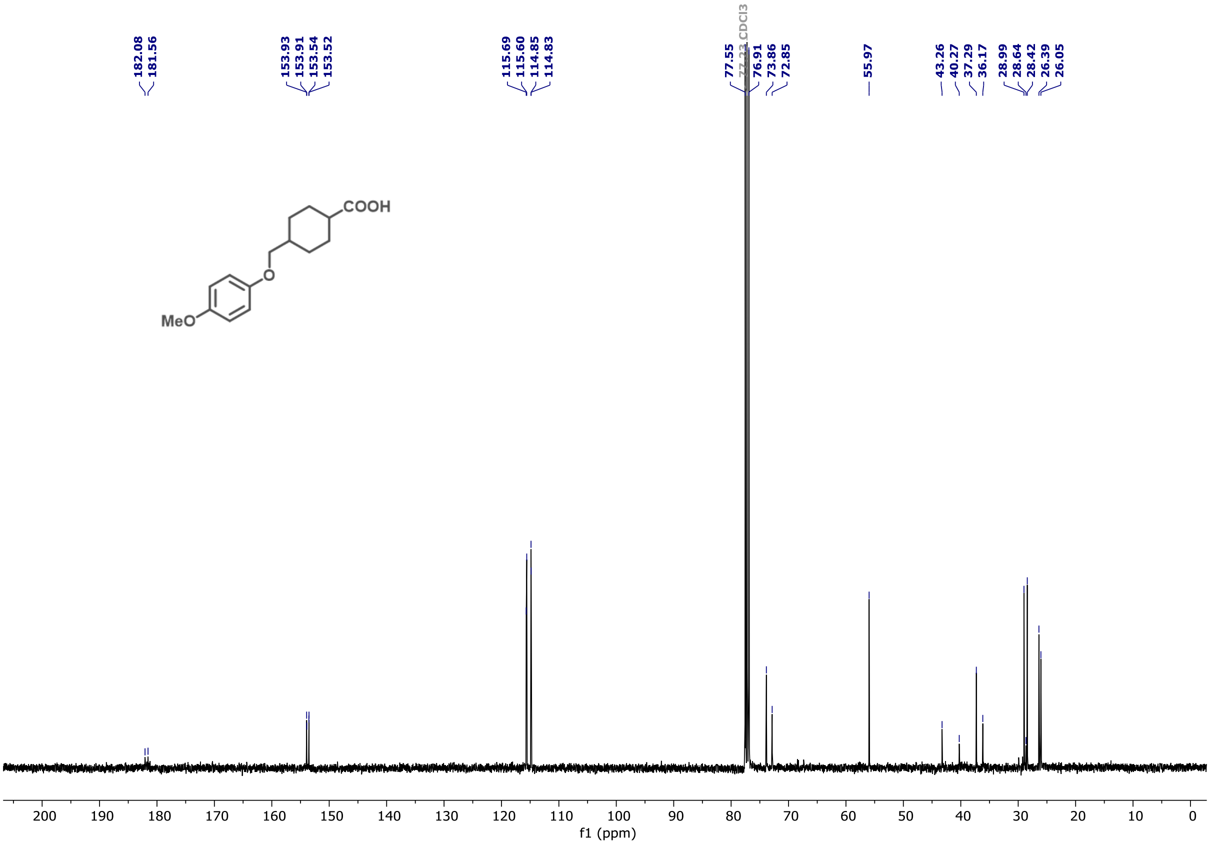

3.6 NMR Spectra of the products

Ethyl (E)-3-(3-(tert-butyl)phenyl)acrylate (1)

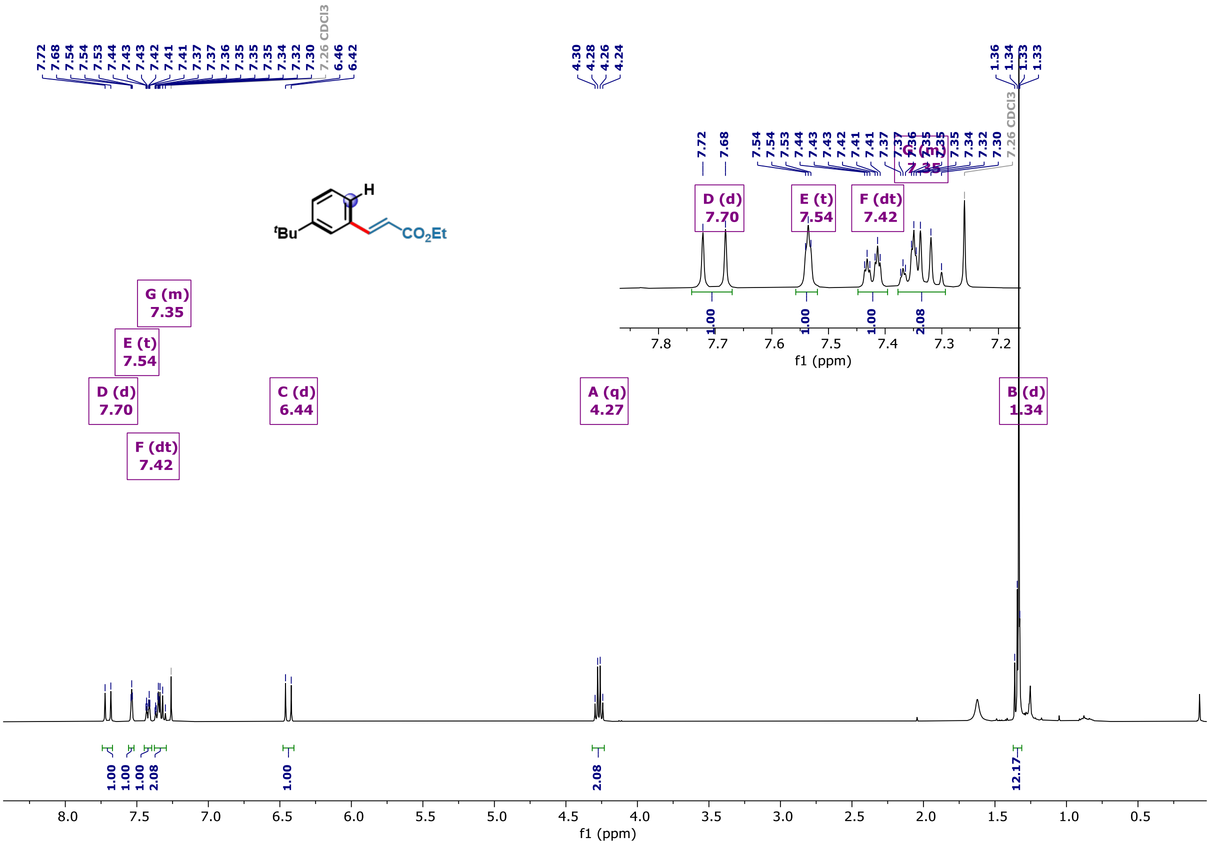

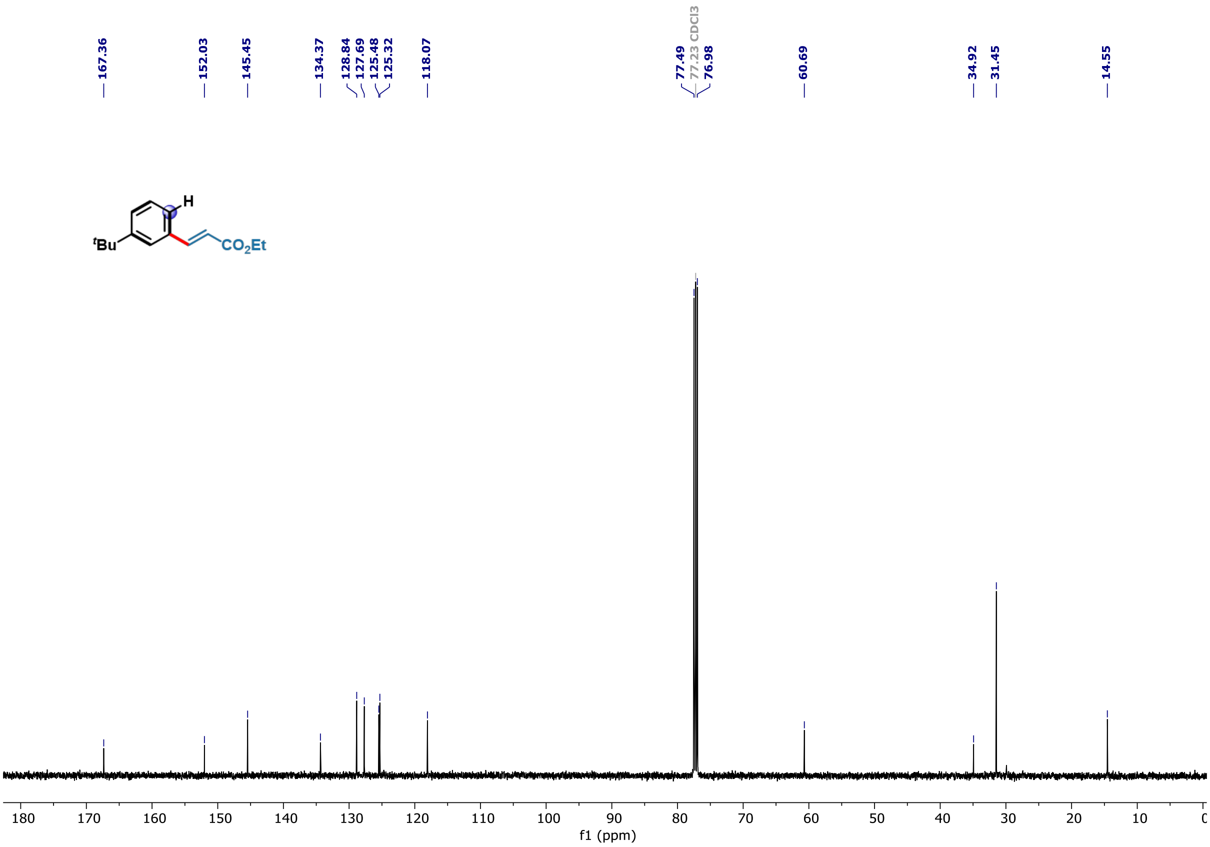

Ethyl cinnamate (2)

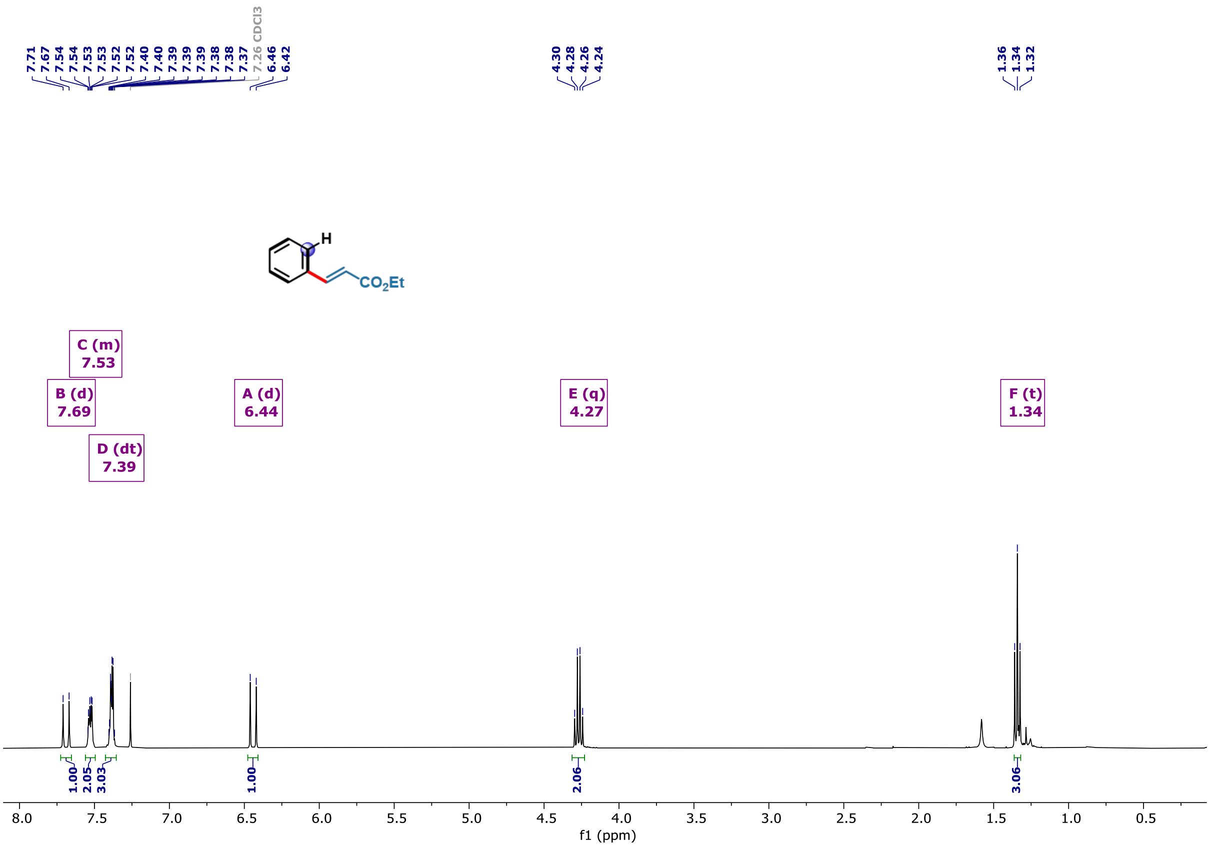

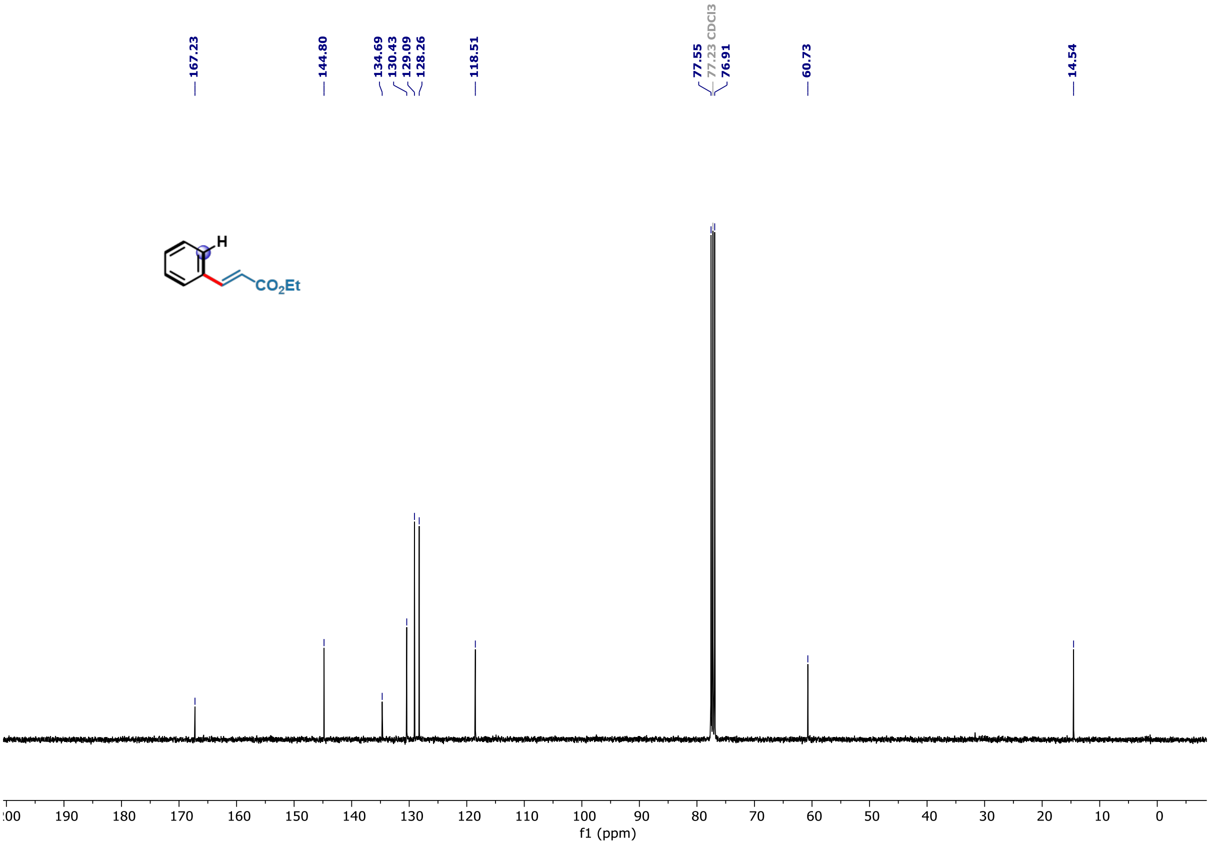

Ethyl (E)-3-(m-tolyl)acrylate (3)

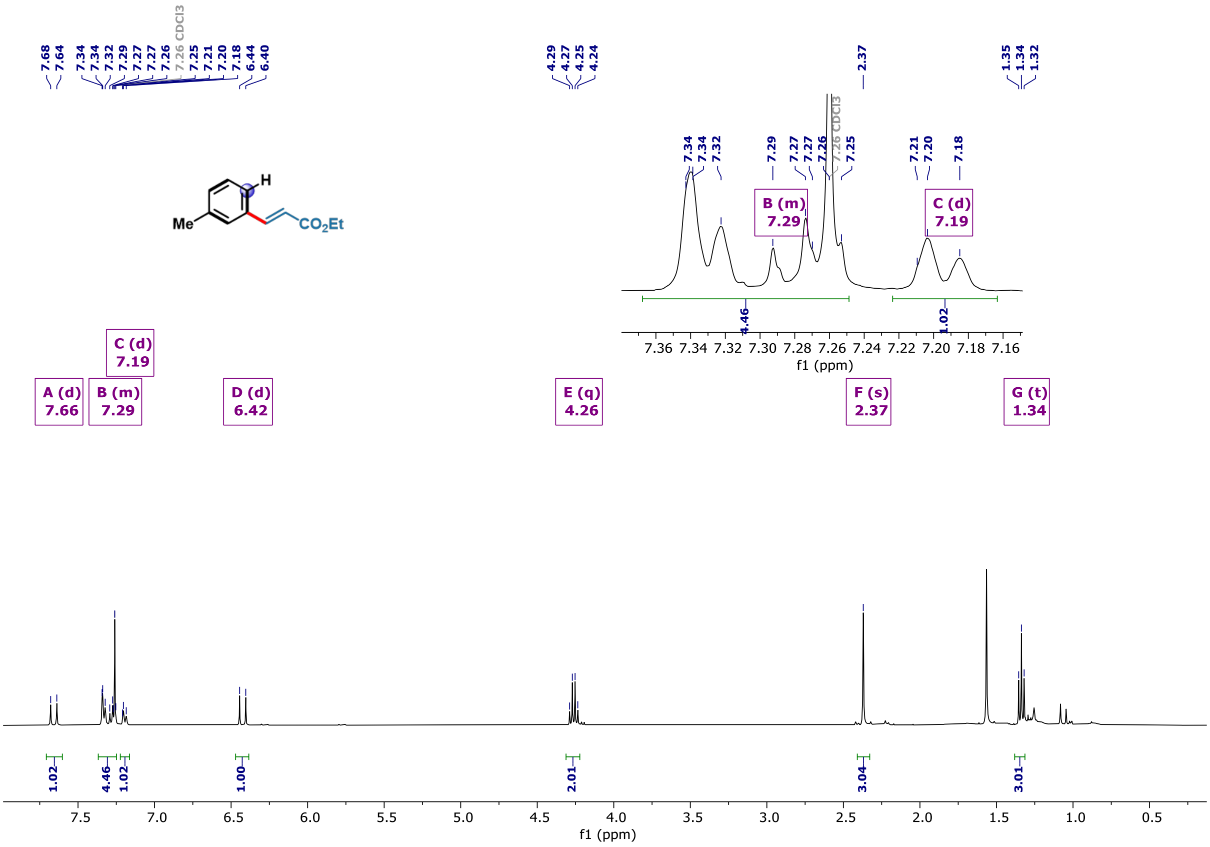

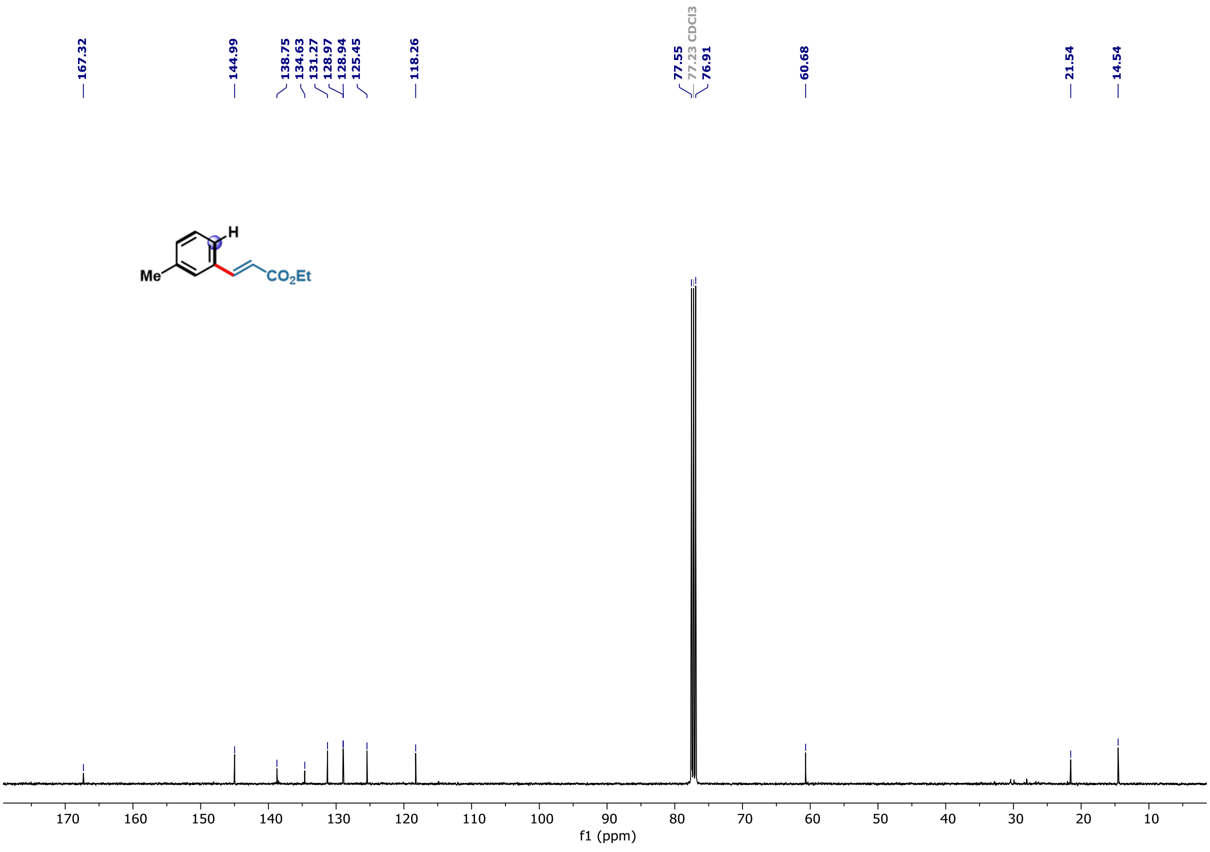

Ethyl (E)-3-(p-tolyl)acrylate (4)

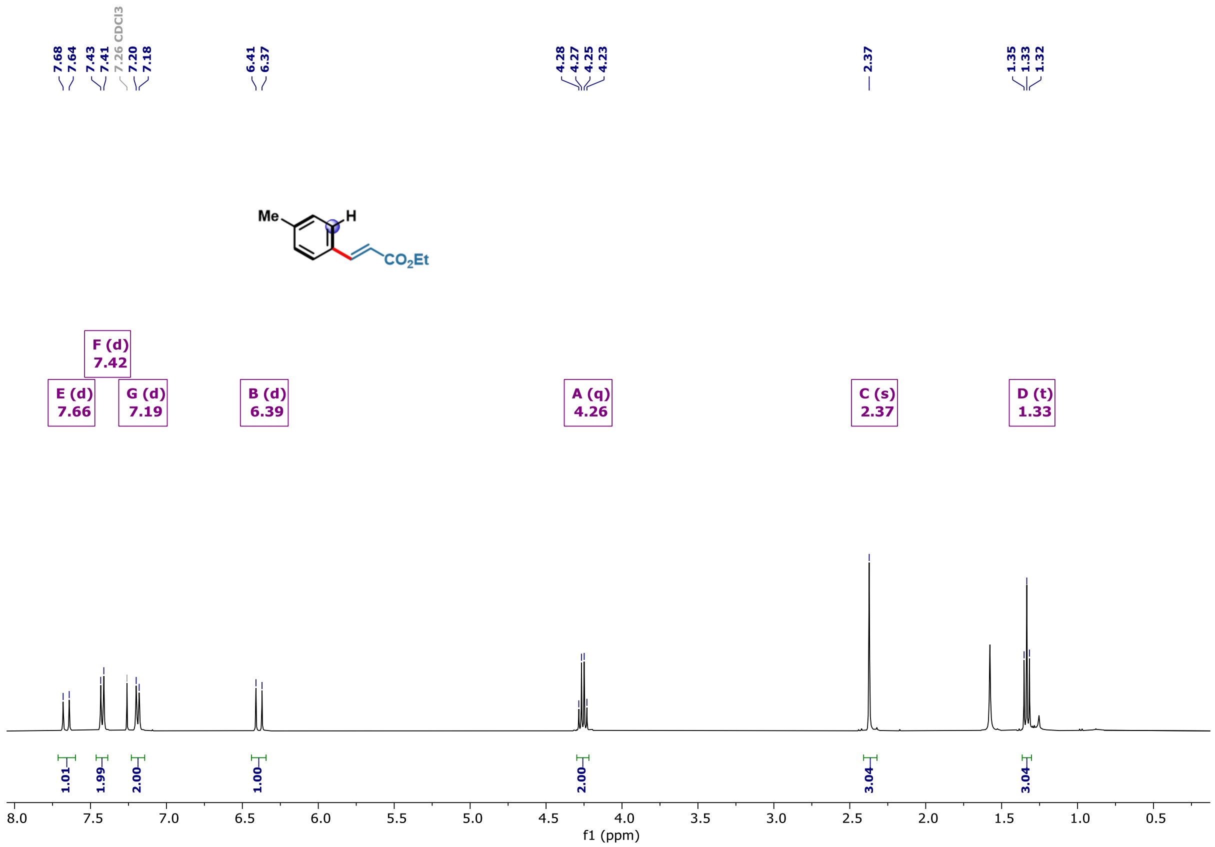

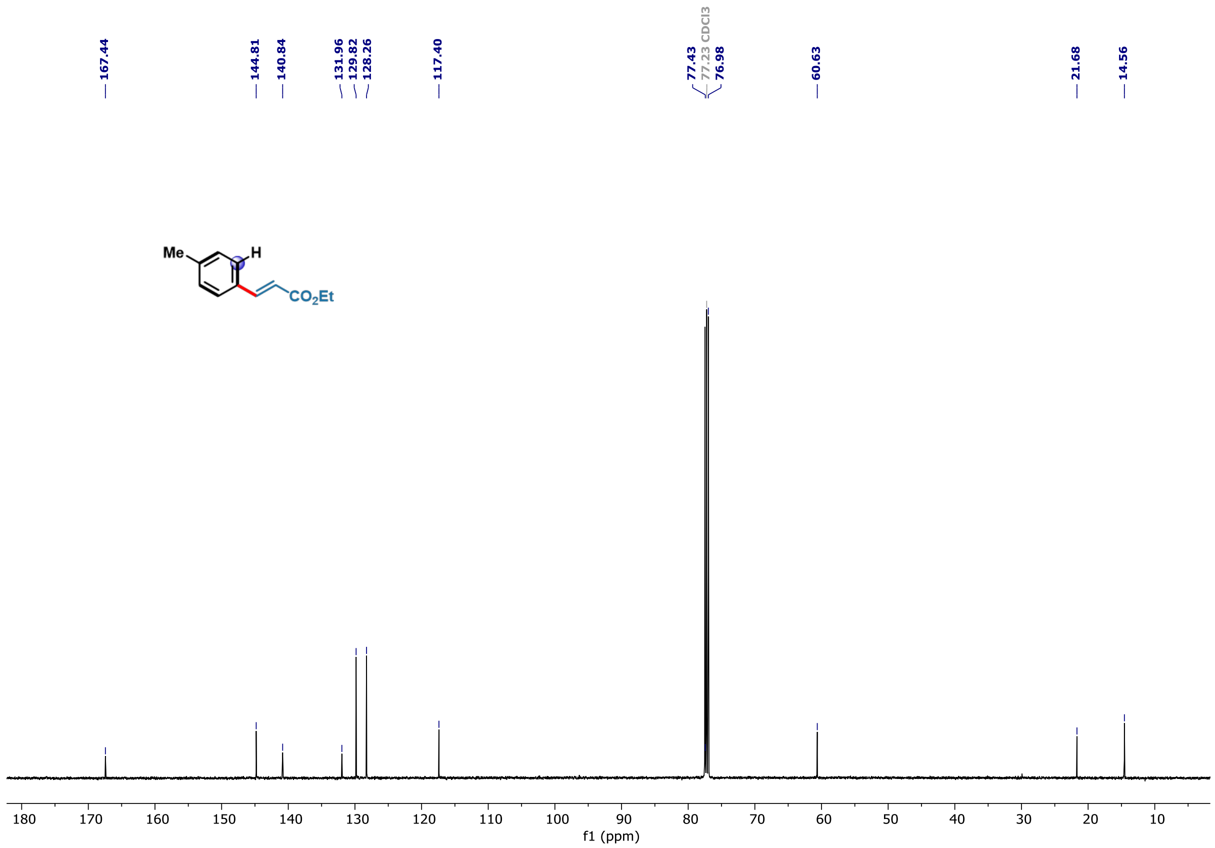

Ethyl (E)-3-(3-ethylphenyl)acrylate (5)

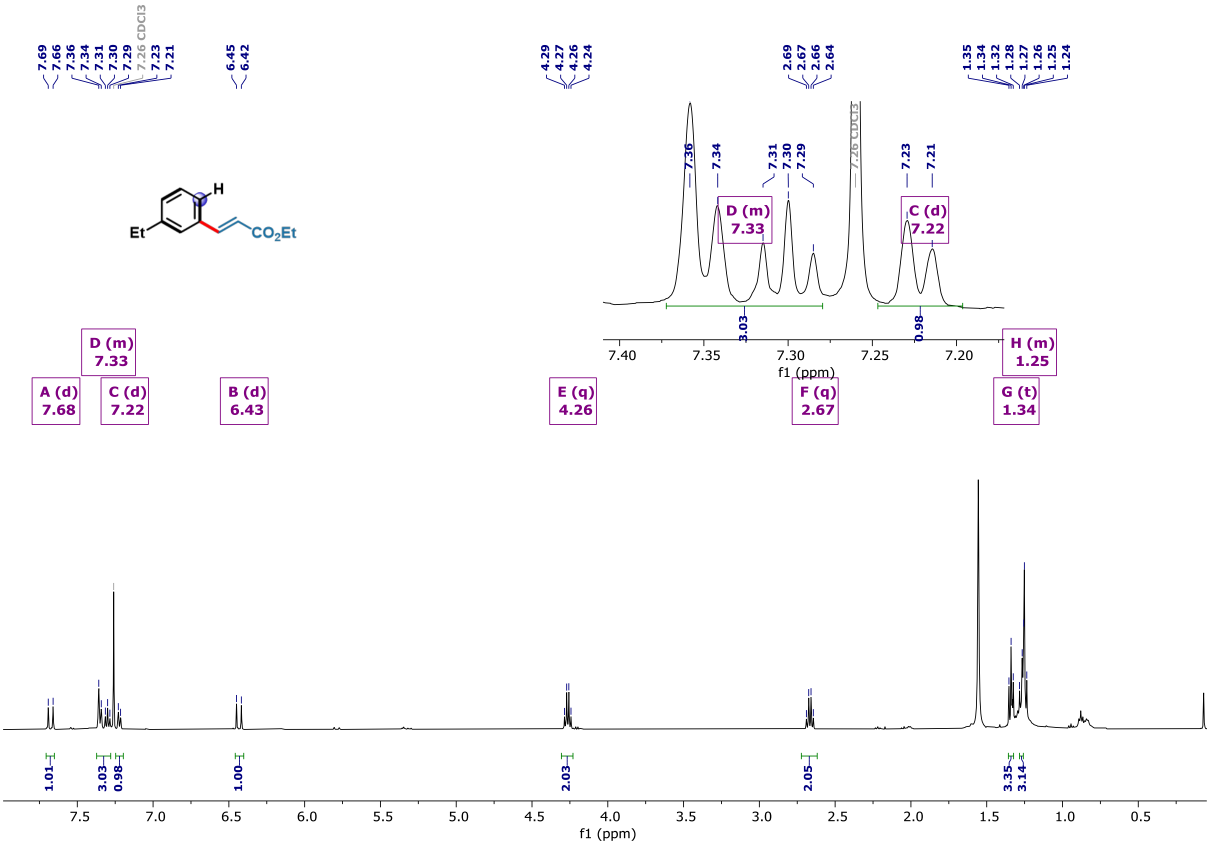

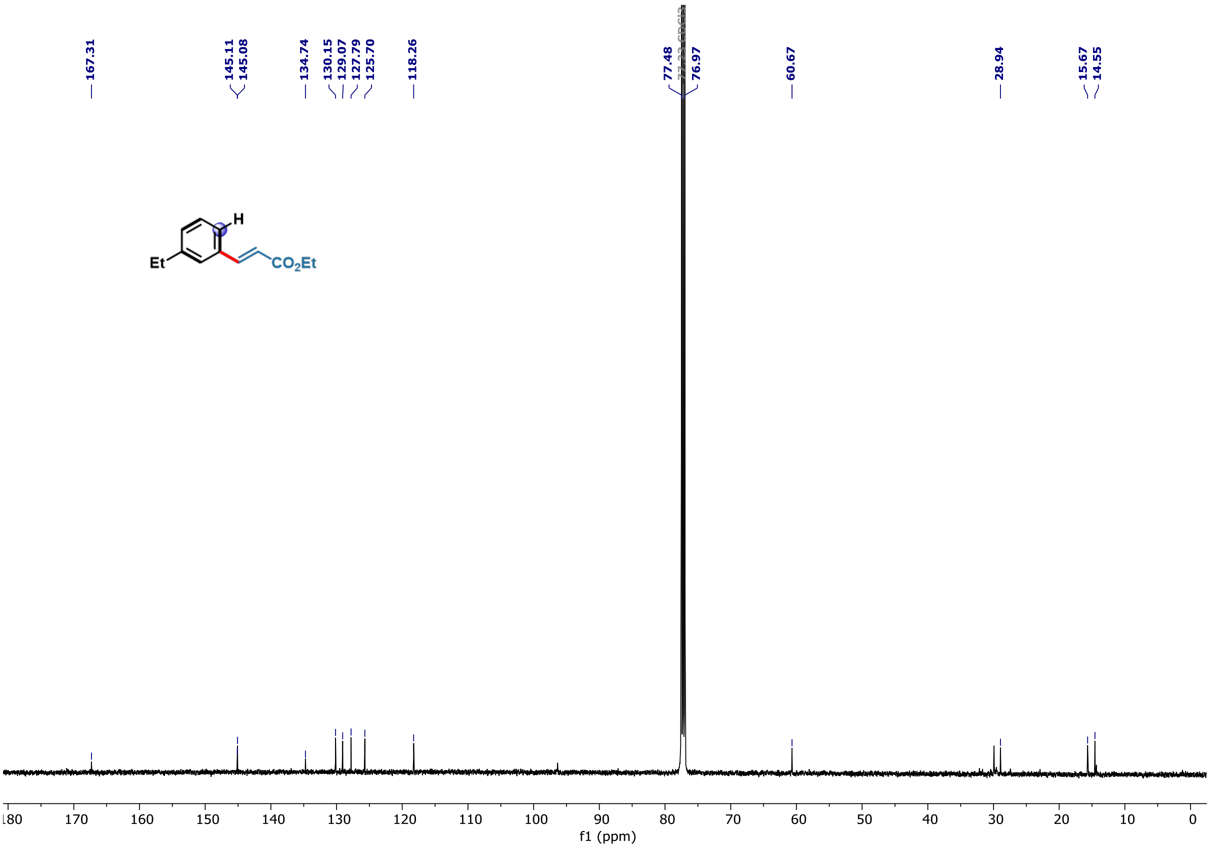

Ethyl (E)-3-(3-propylphenyl)acrylate (6)

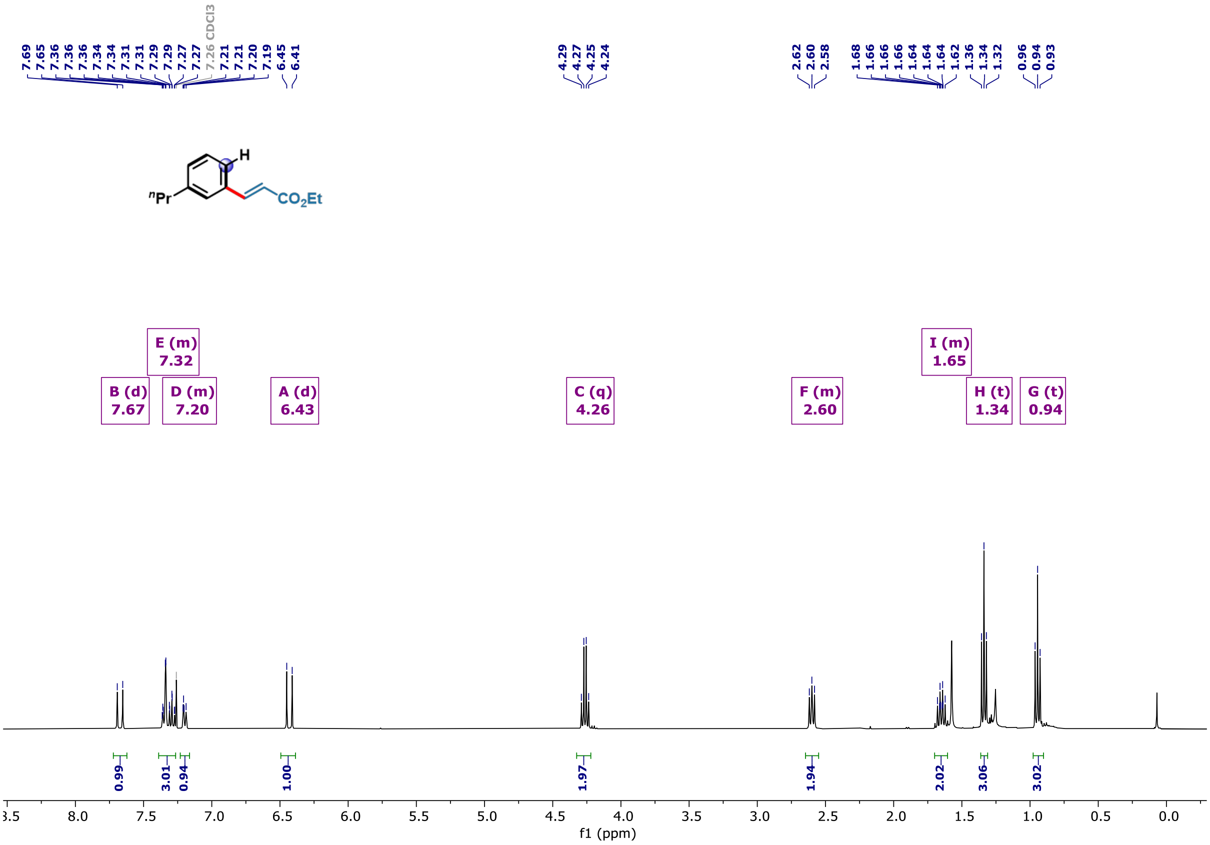

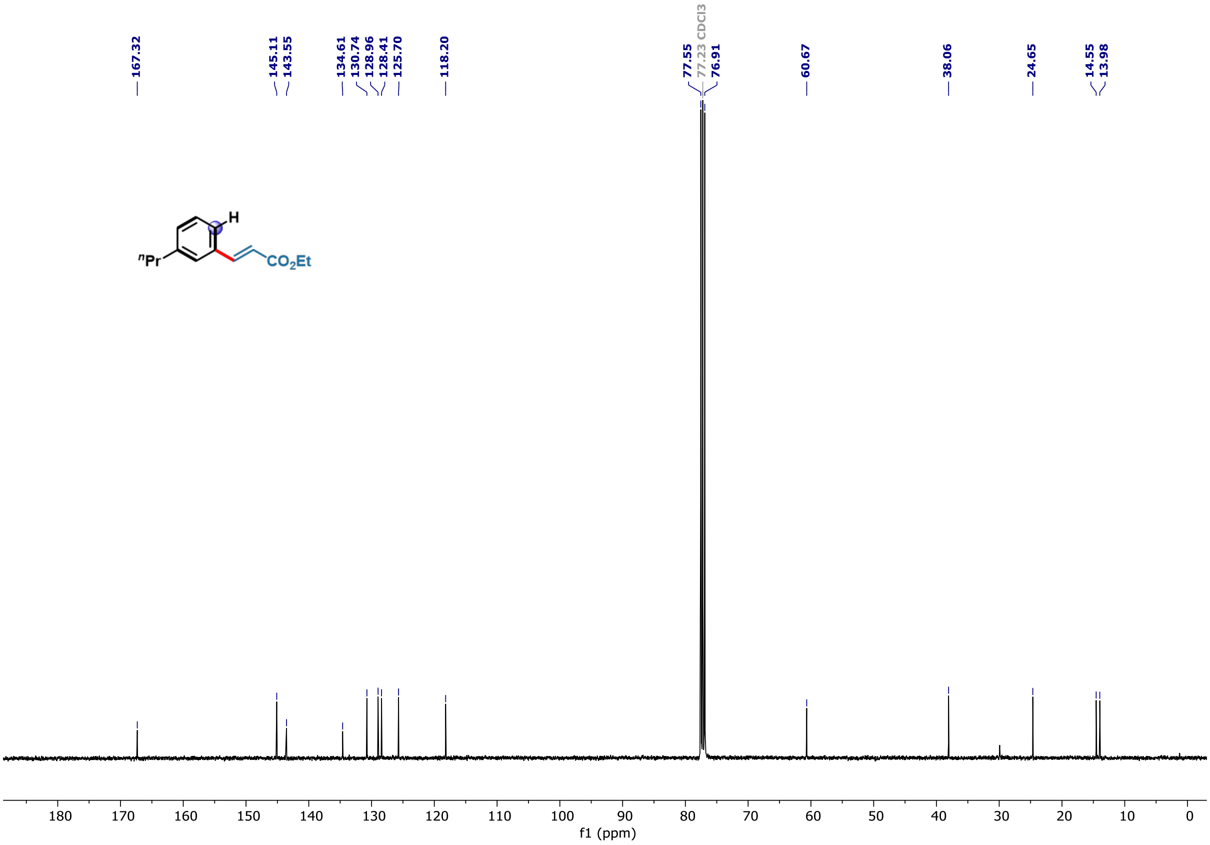

Ethyl (E)-3-(3-isopropylphenyl)acrylate (7)

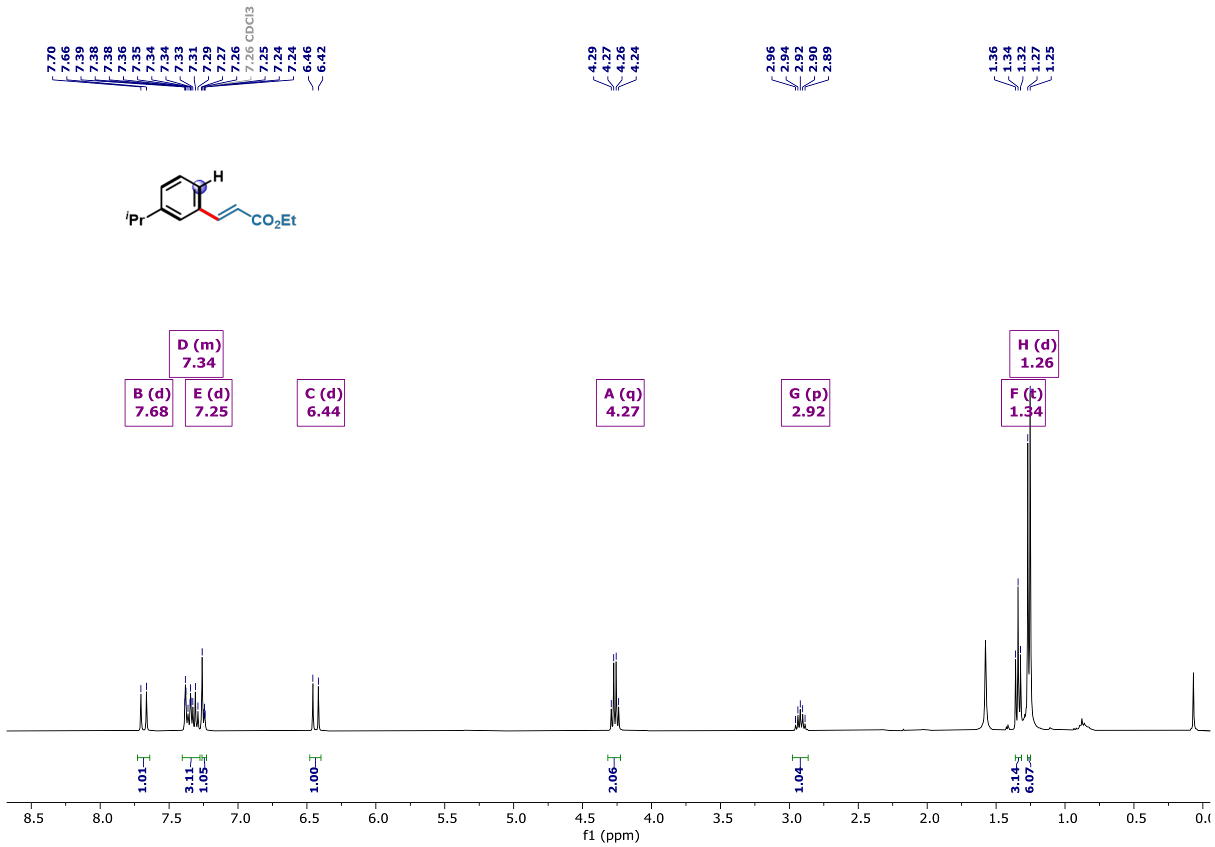

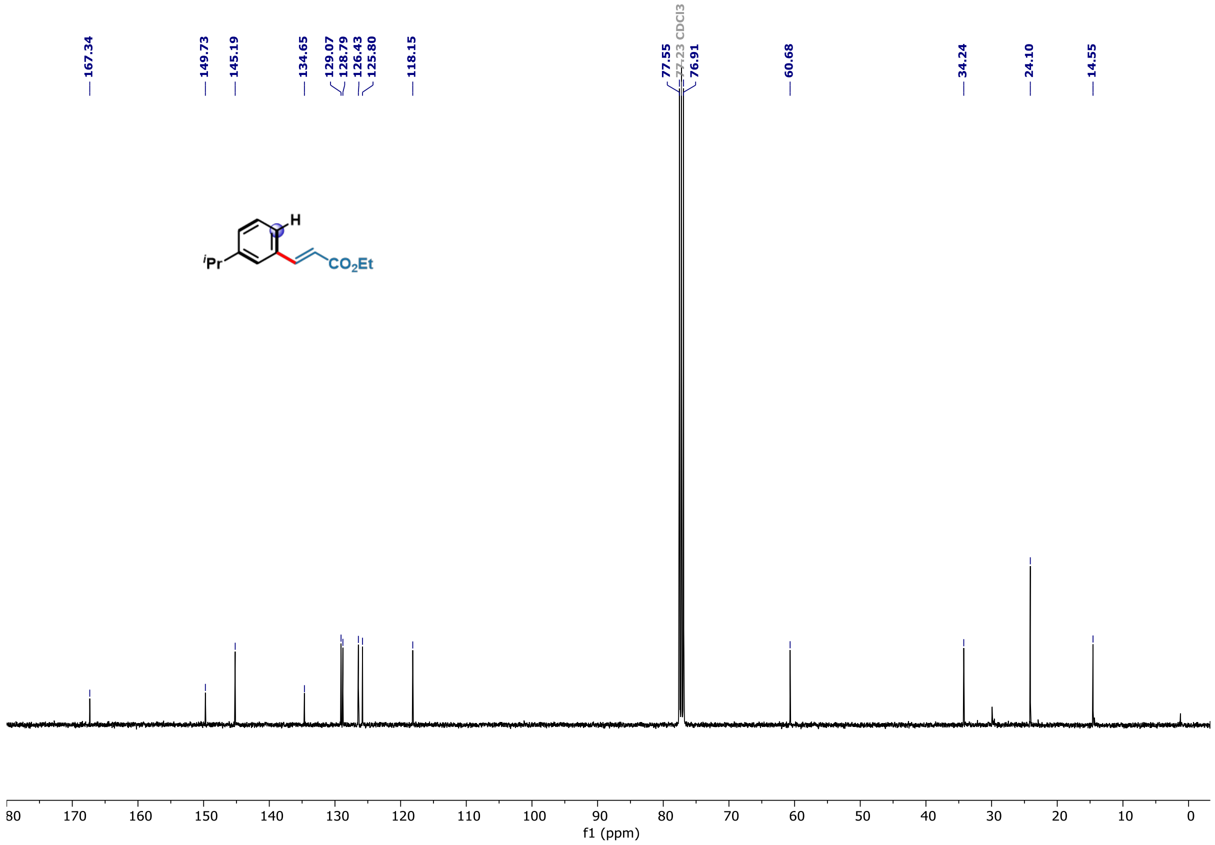

Ethyl (E)-3-(3-butylphenyl)acrylate (8)

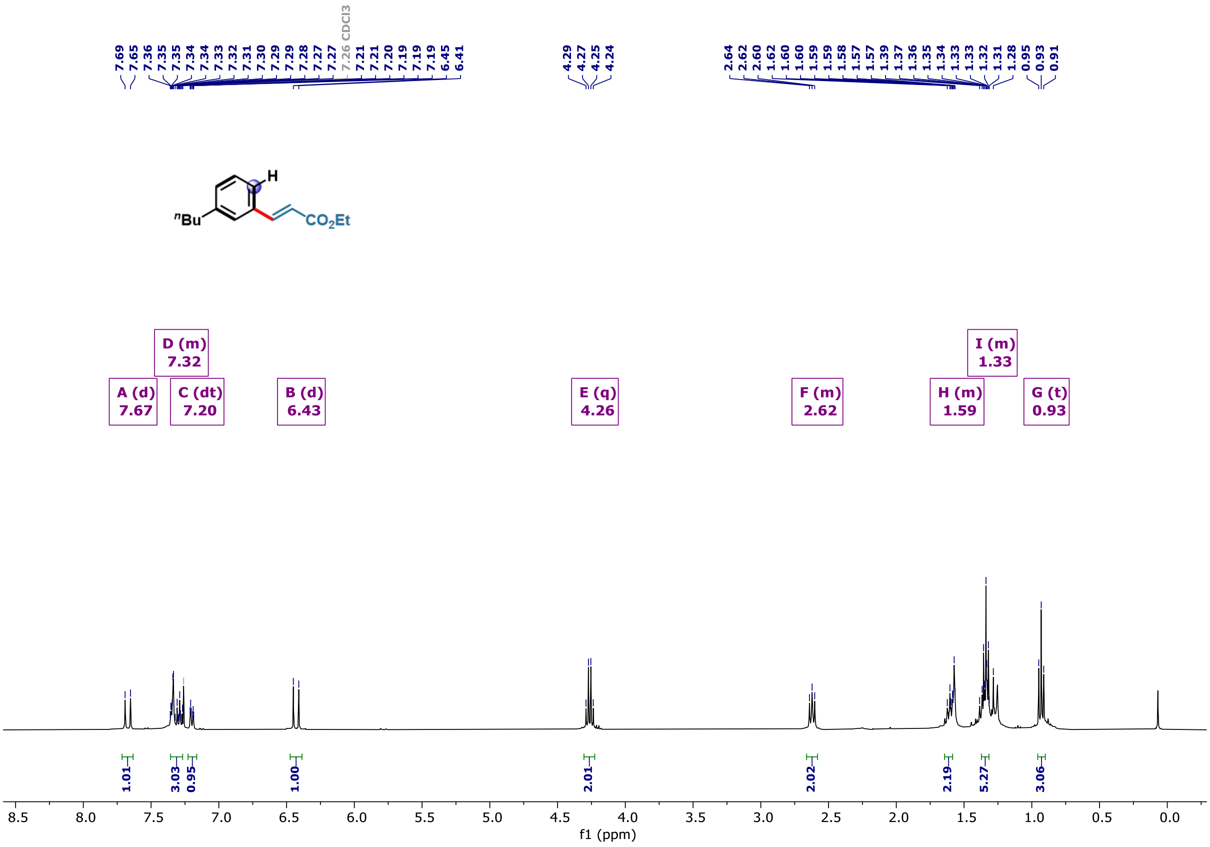

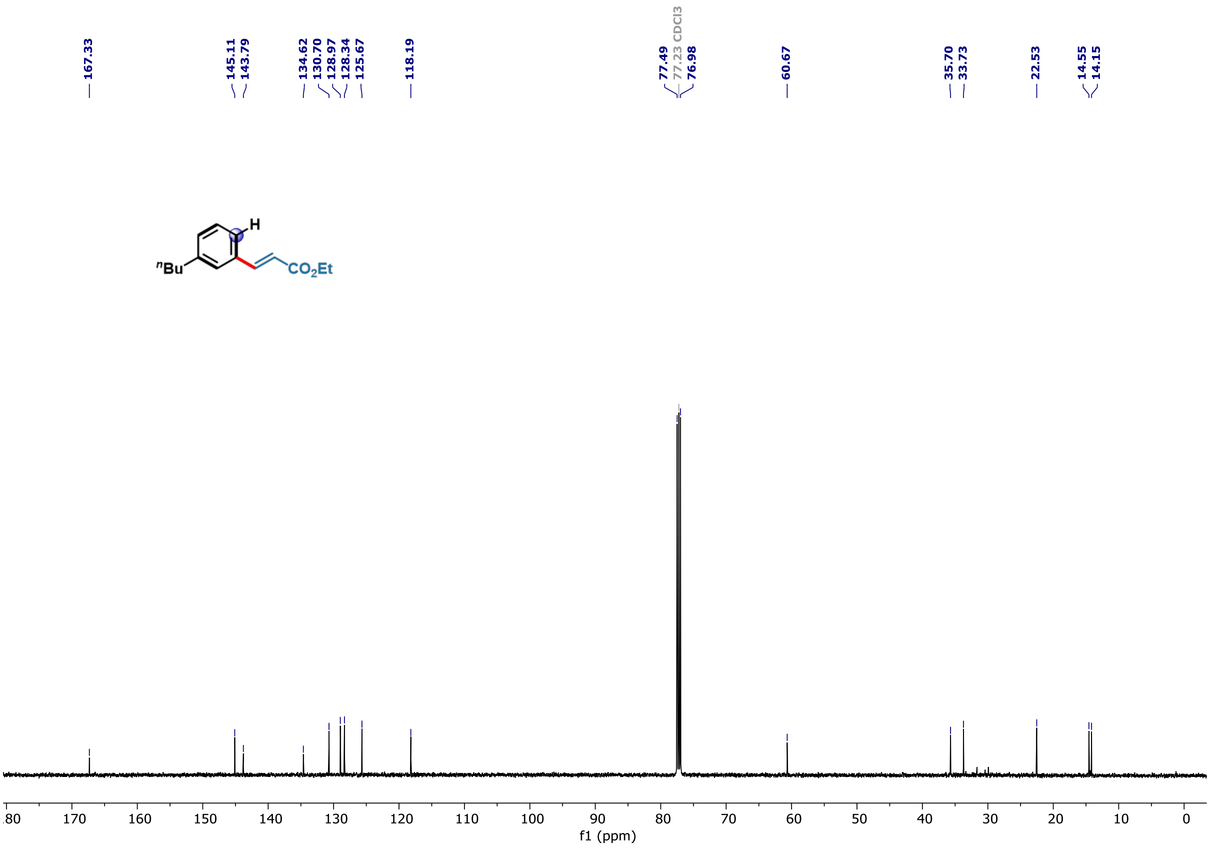

Ethyl (E)-3-(3-pentylphenyl)acrylate (9)

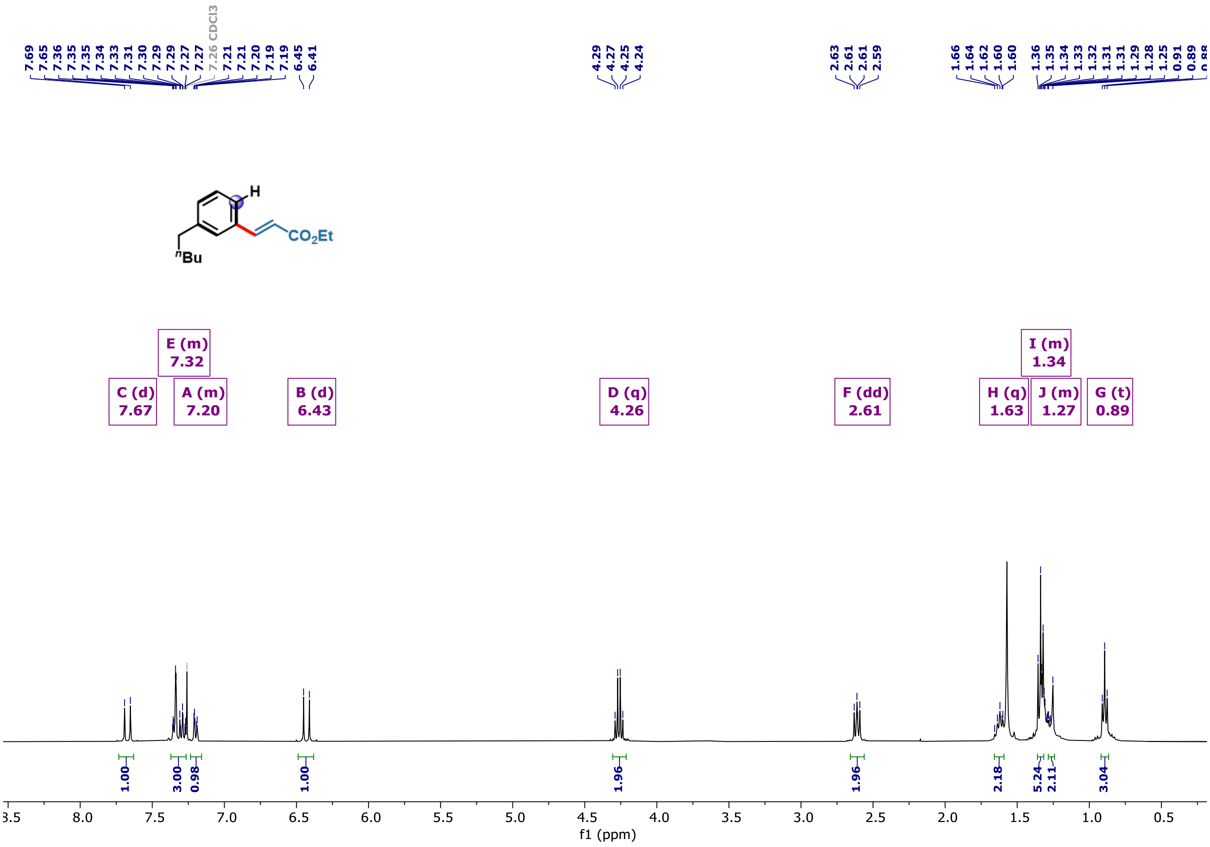

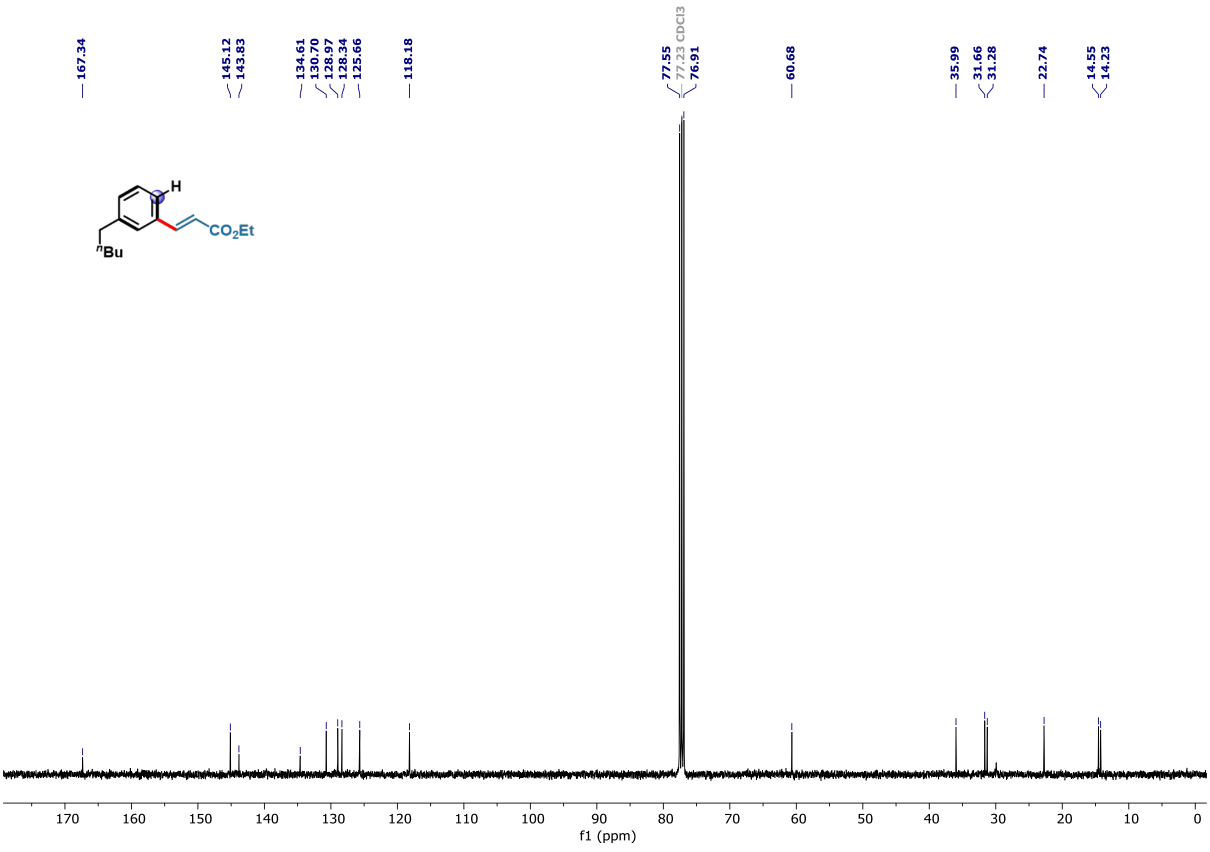

Ethyl (E)-3-(3-(4-ethylcyclohexyl)phenyl)acrylate (10)

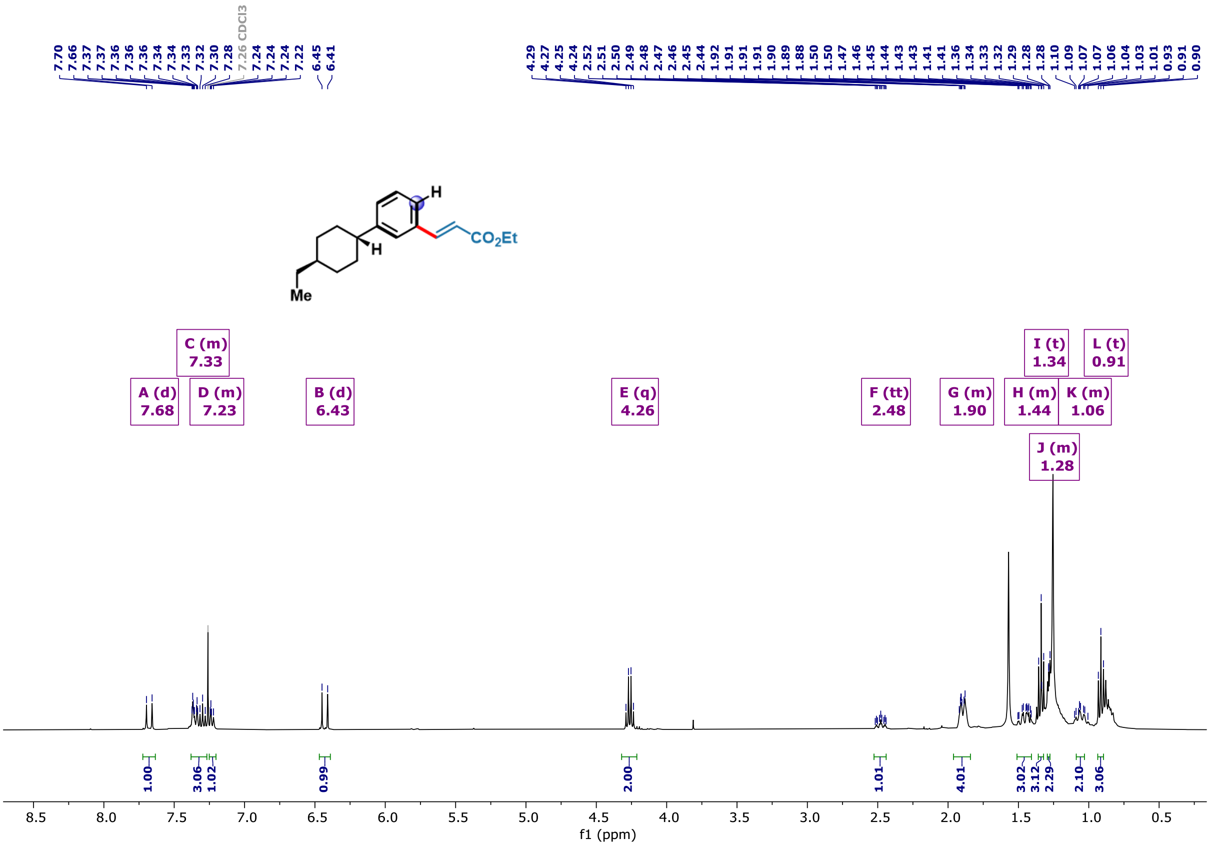

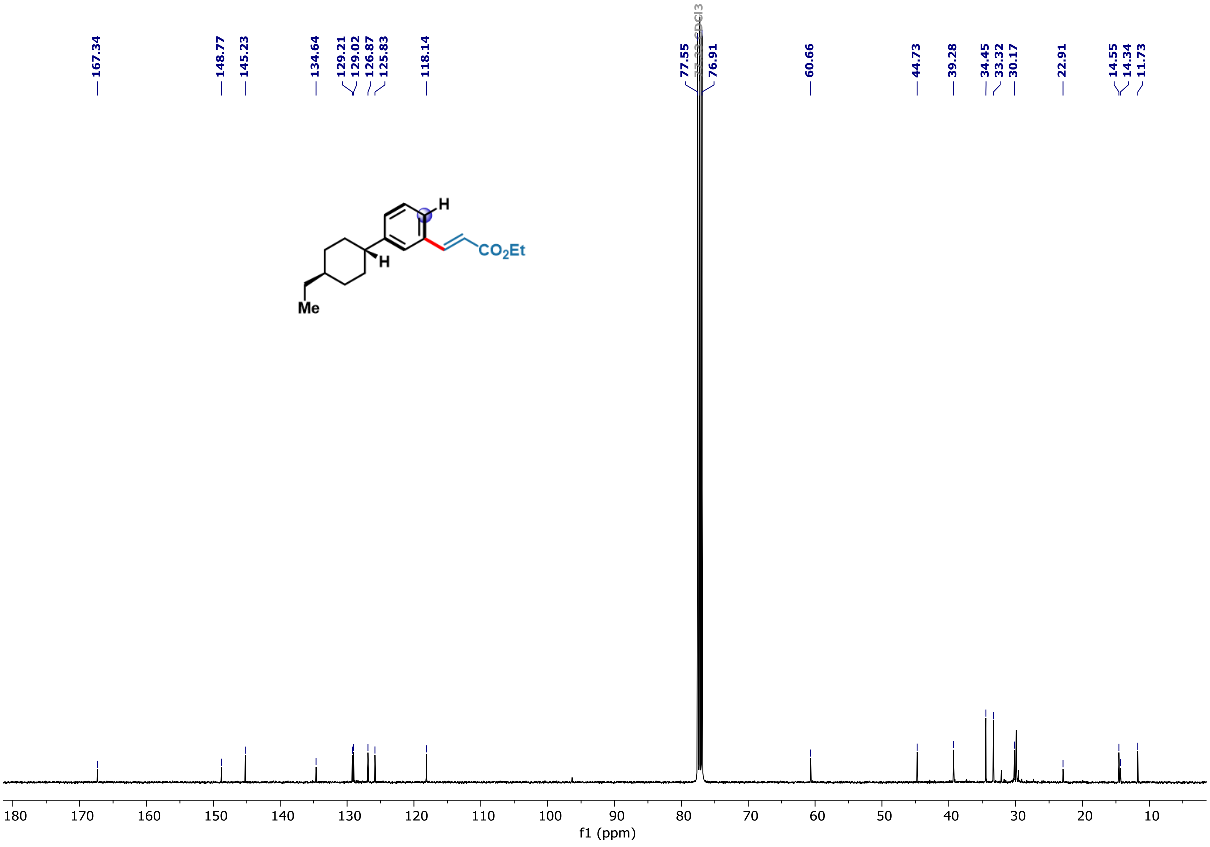

Ethyl (E)-3-(3-(4-pentylcyclohexyl)phenyl)acrylate (11)

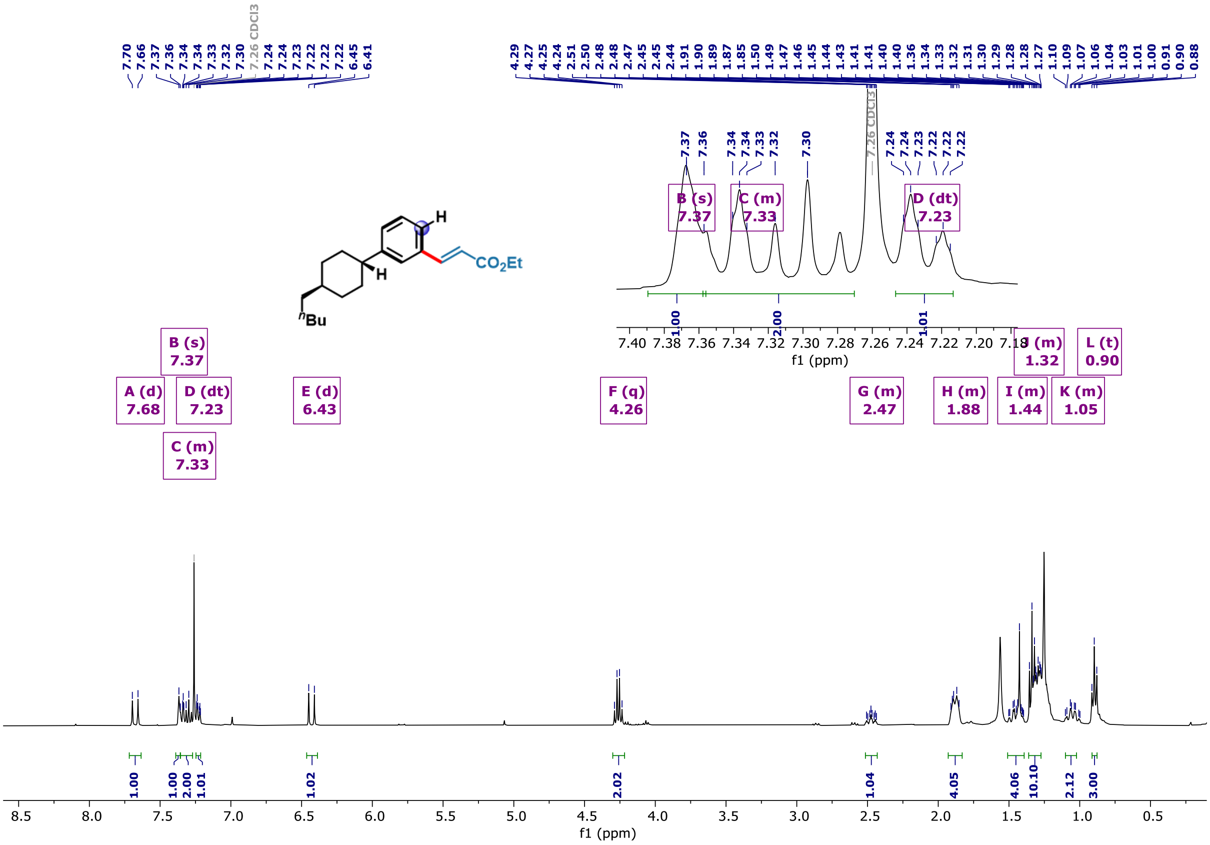

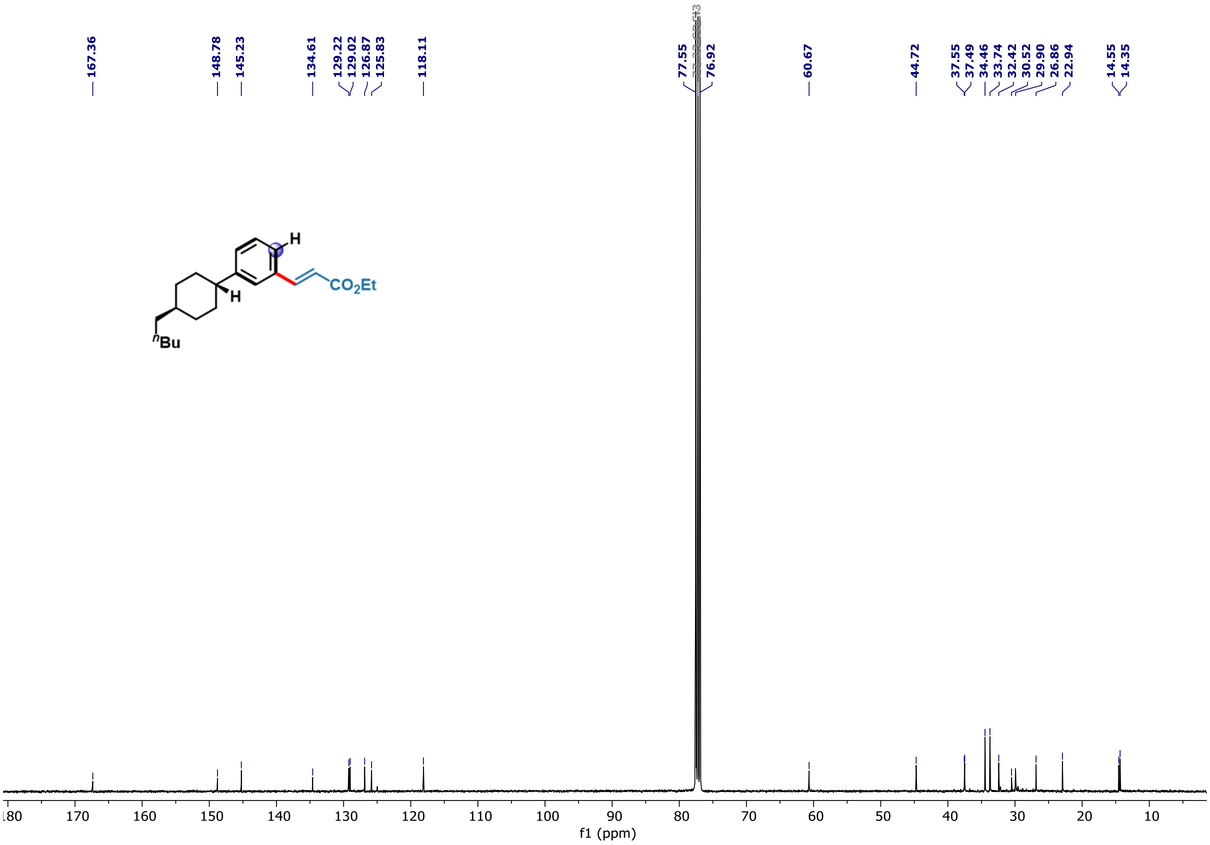

Ethyl (E)-3-(3-(methoxymethyl)phenyl)acrylate (12)

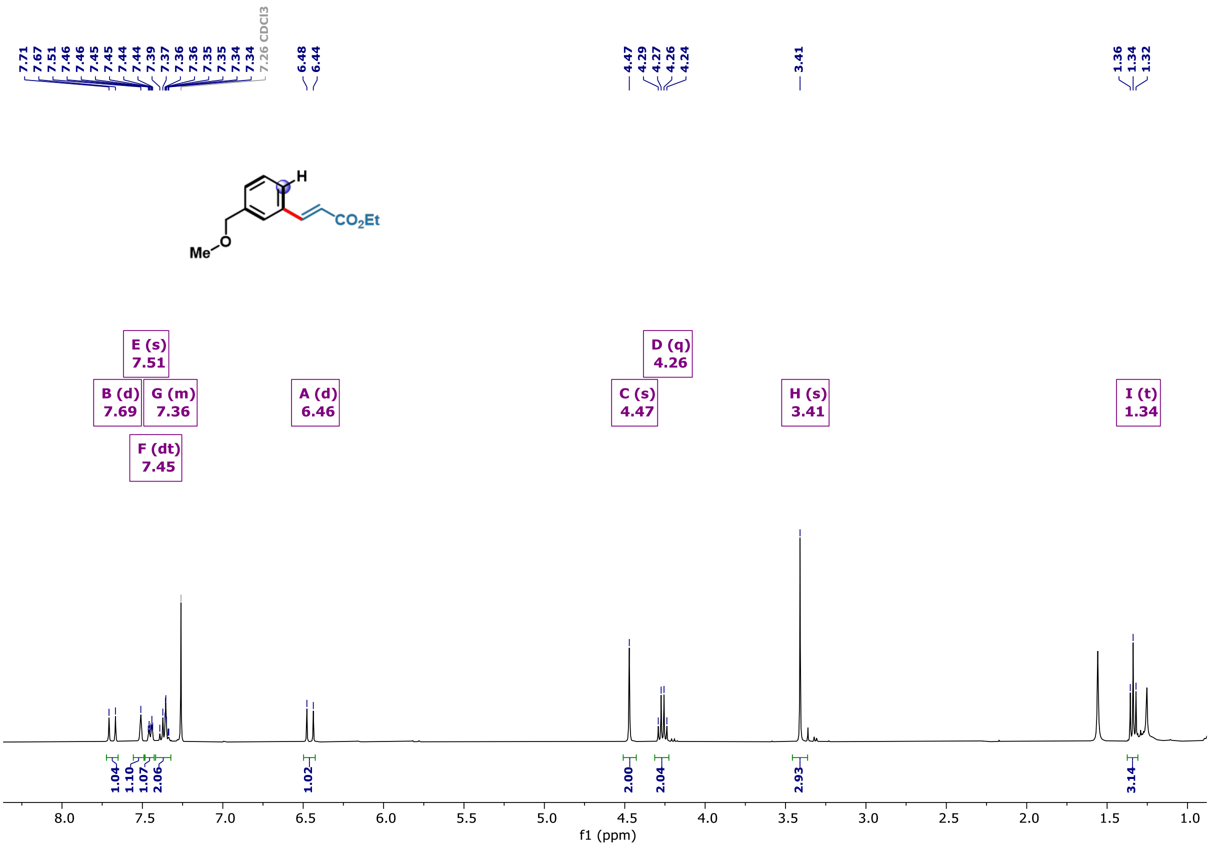

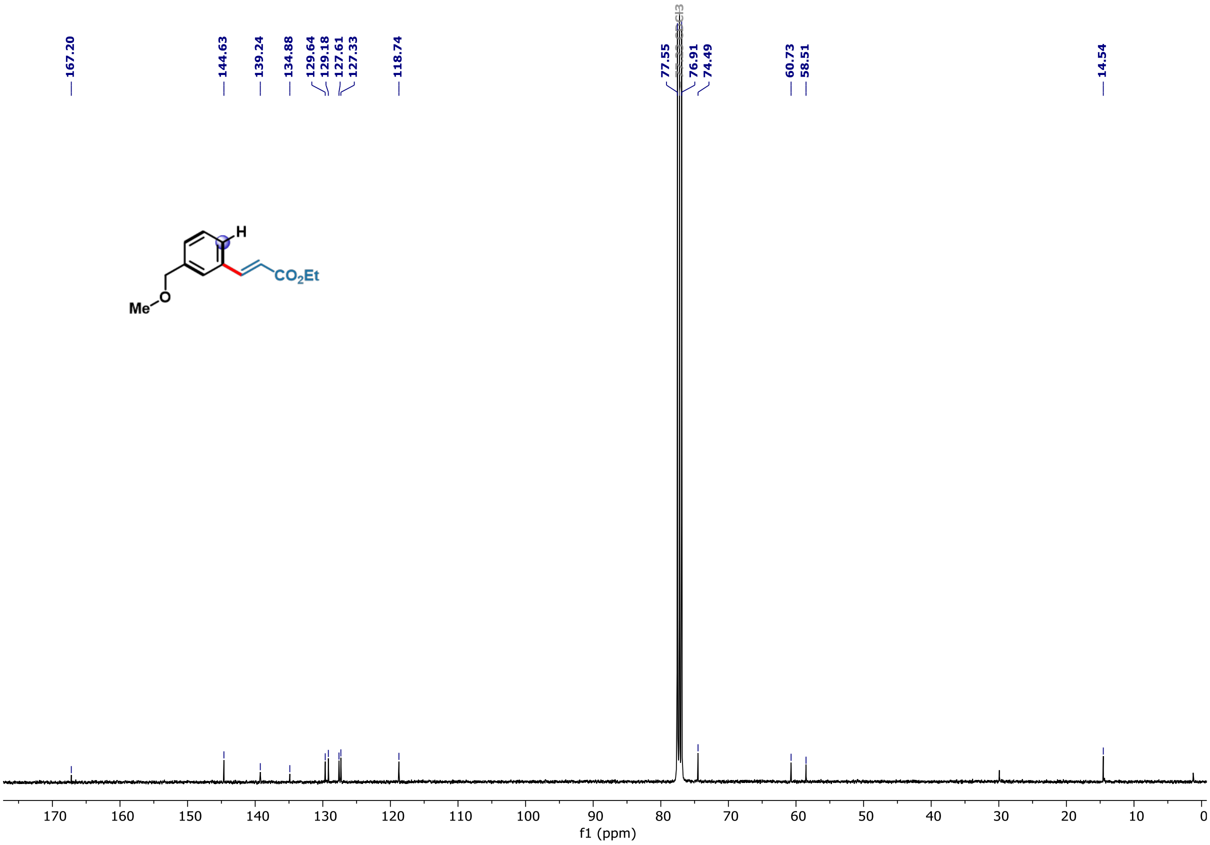

Methyl (E)-3-(3-ethoxy-3-oxoprop-1-en-1-yl)benzoate (13)

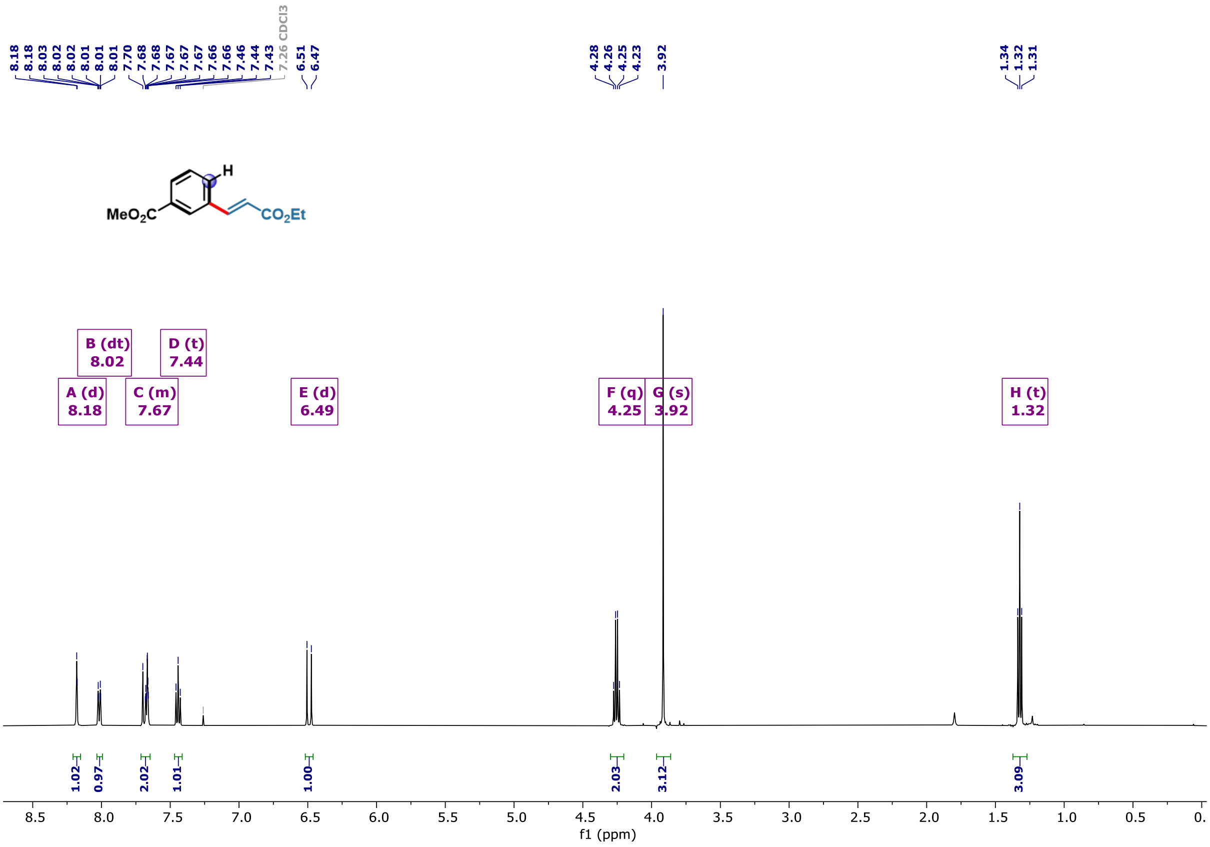

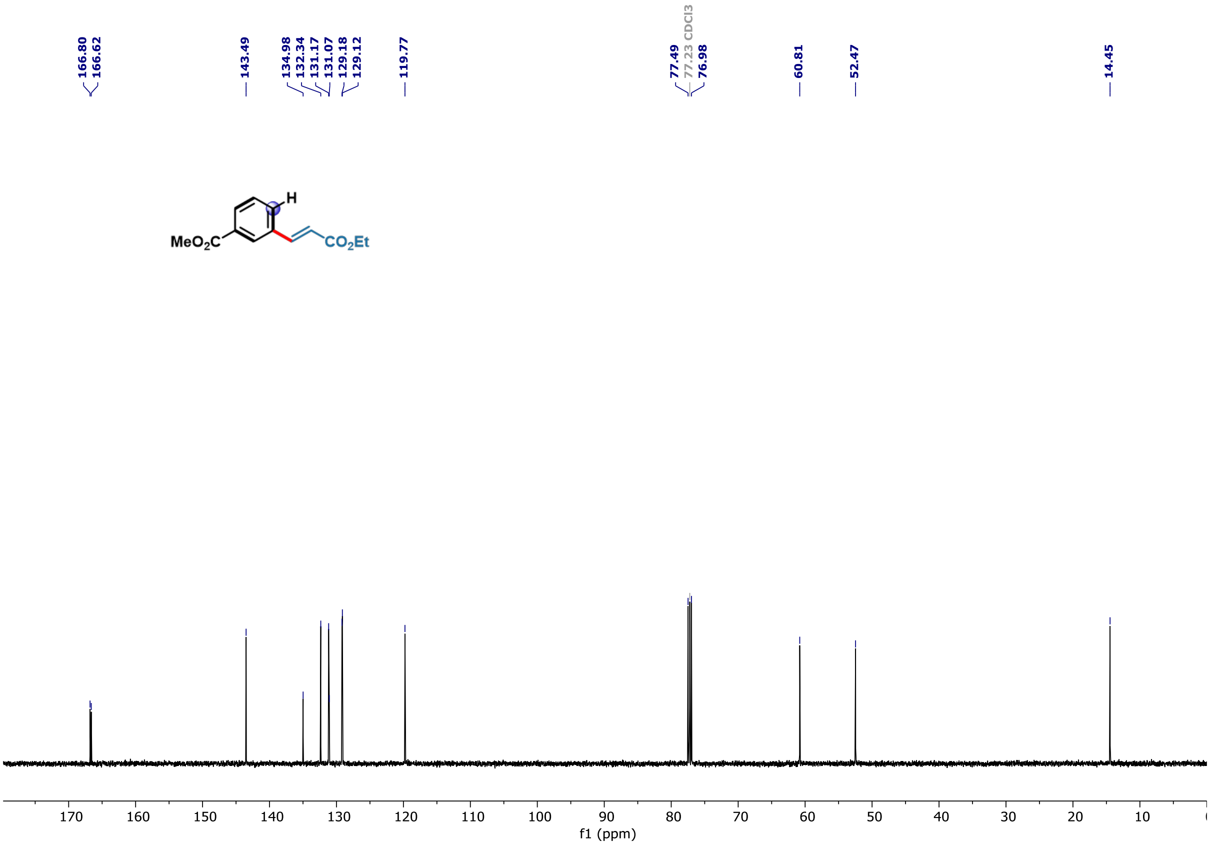

Ethyl (E)-3-(3-(trifluoromethyl)phenyl)acrylate (14)

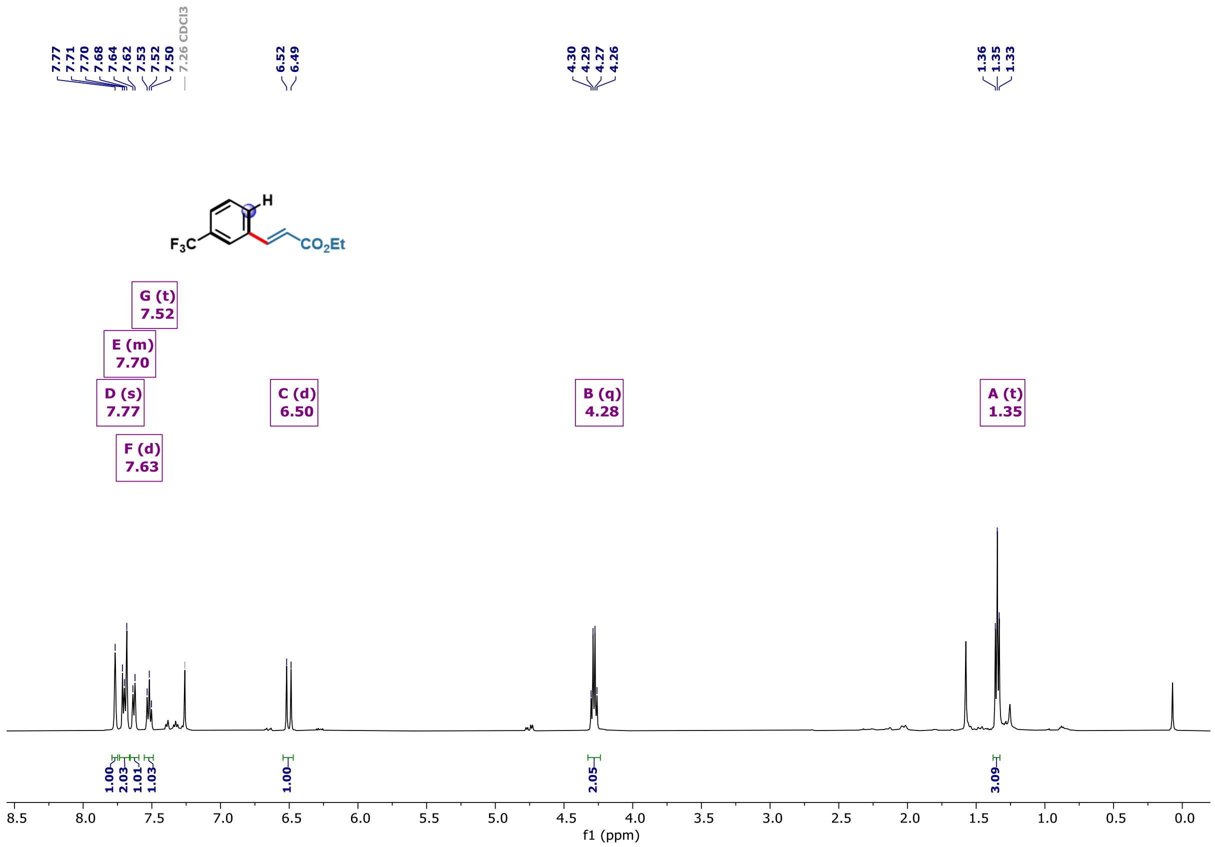

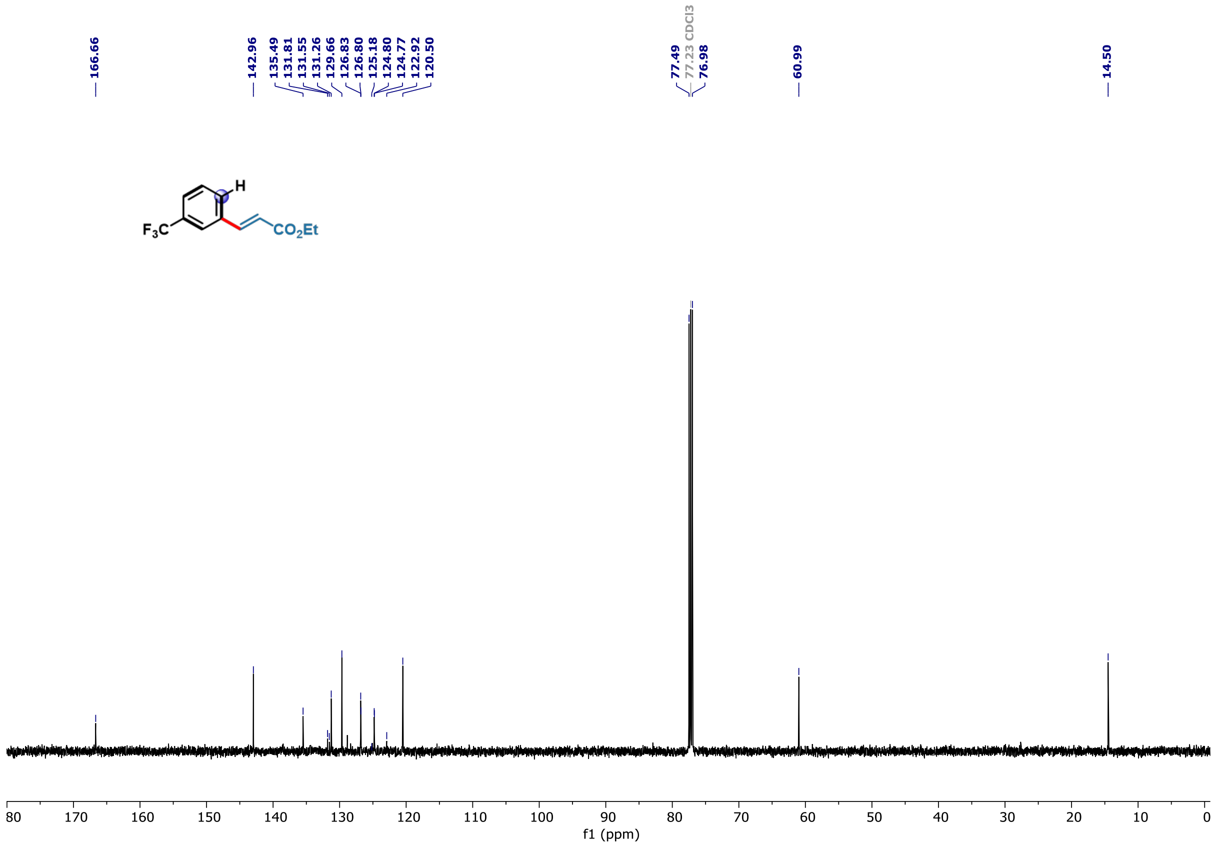

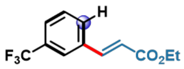
H
F3C
CO2Et

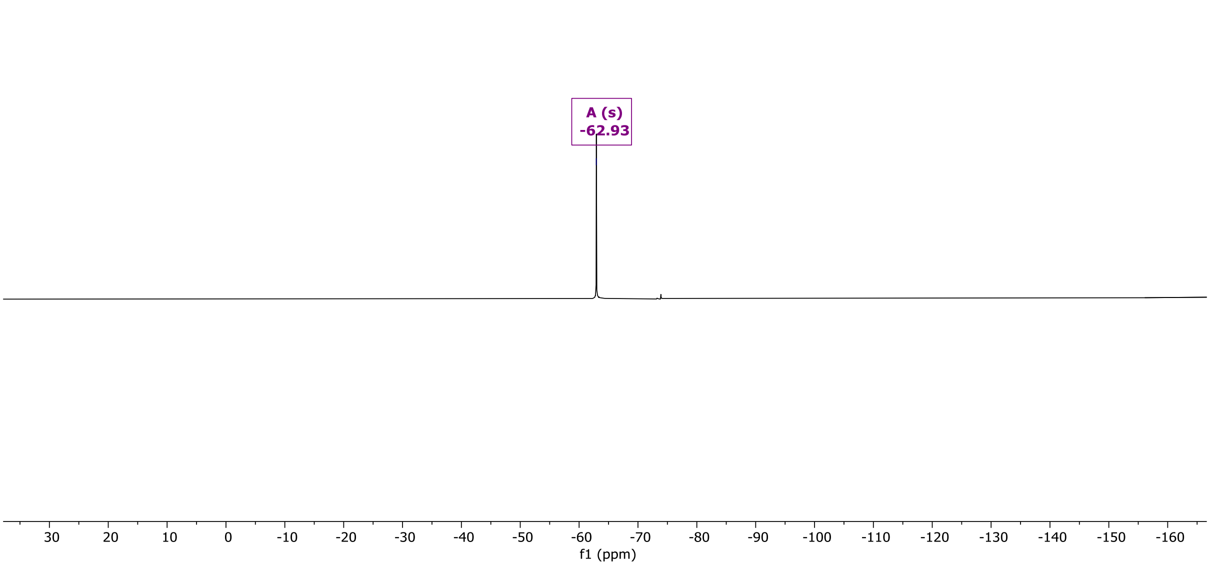
-62.93
A (s)
-62.93
30
20
10
0
-10
-20
-30
-40
-50
-60
-70
-80
-90
-100
-110
-120
-130
-140
-150
-160
f1 (ppm)

Ethyl (E)-3-(4'-chloro-[1,1'-biphenyl]-3-yl)acrylate (15)

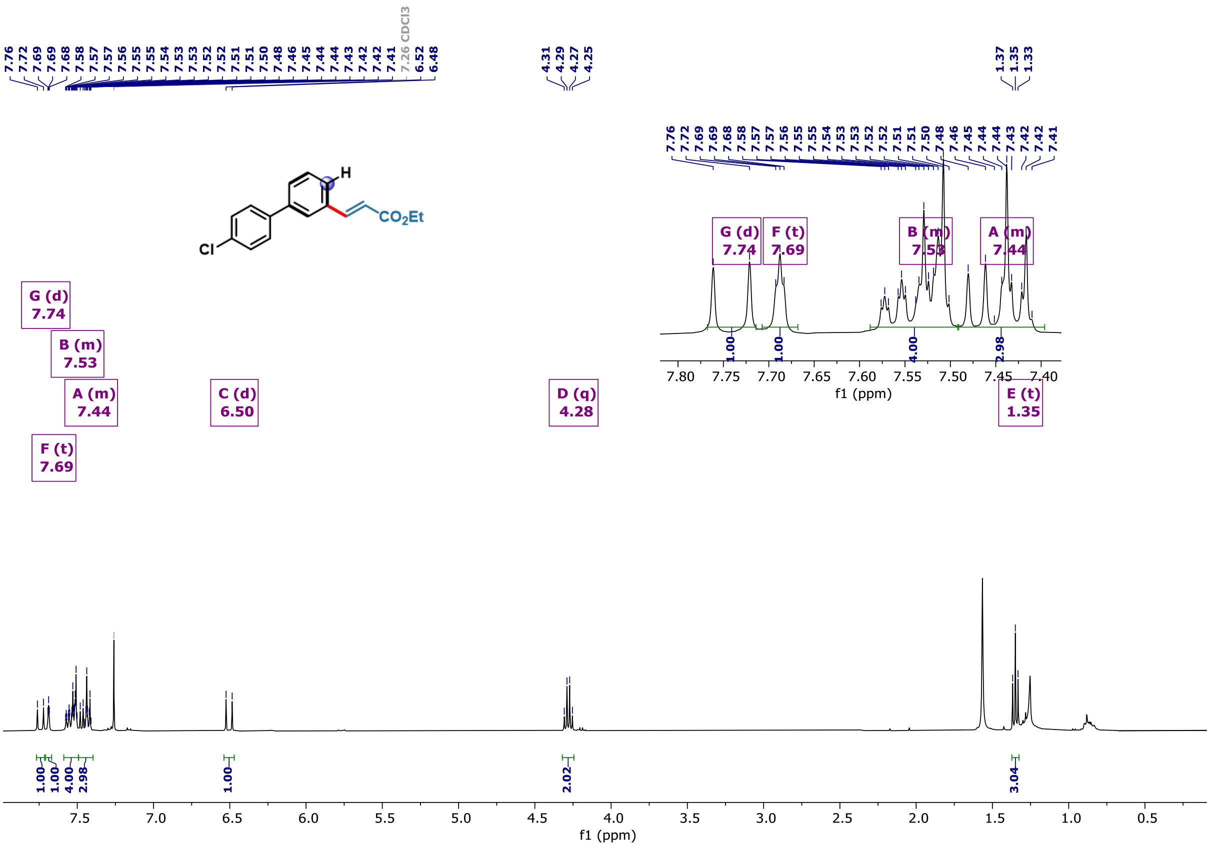

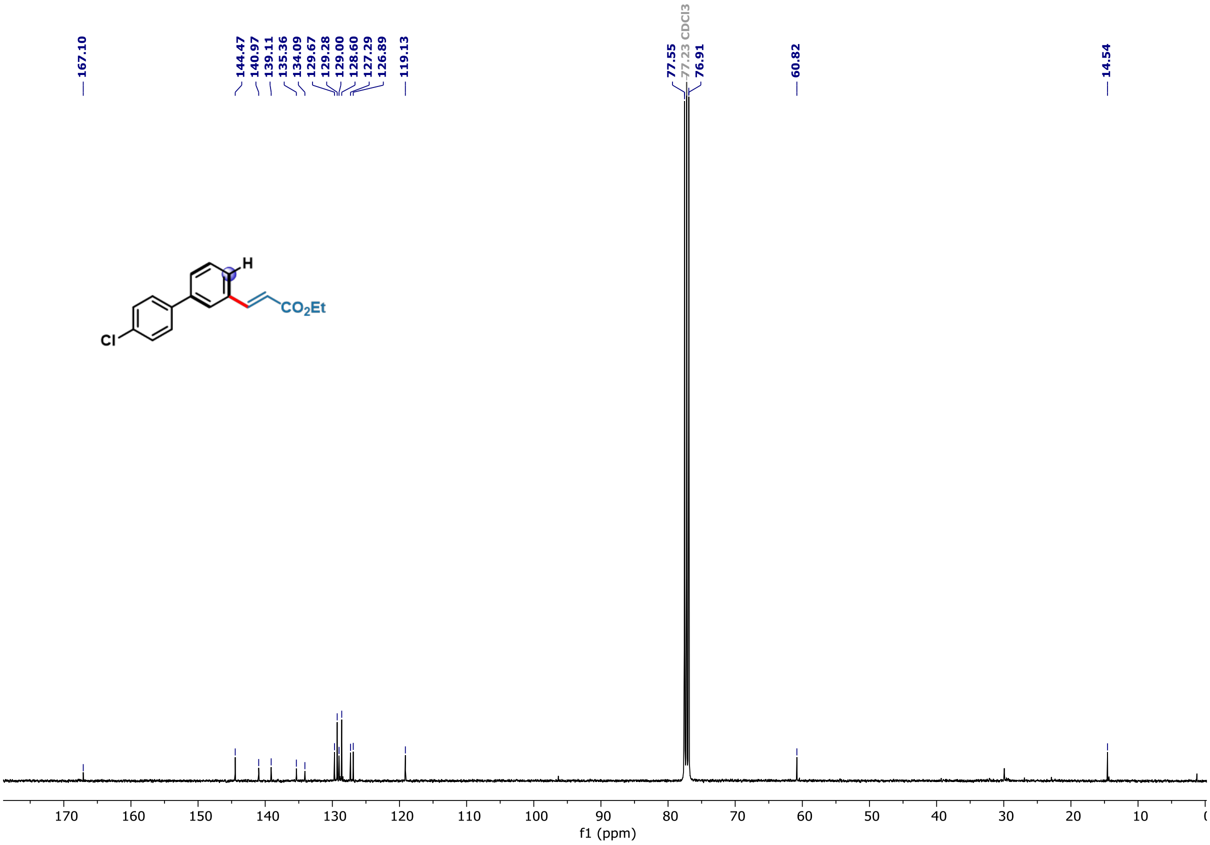

Ethyl (E)-3-(3-(phenoxymethyl)phenyl)acrylate (16)

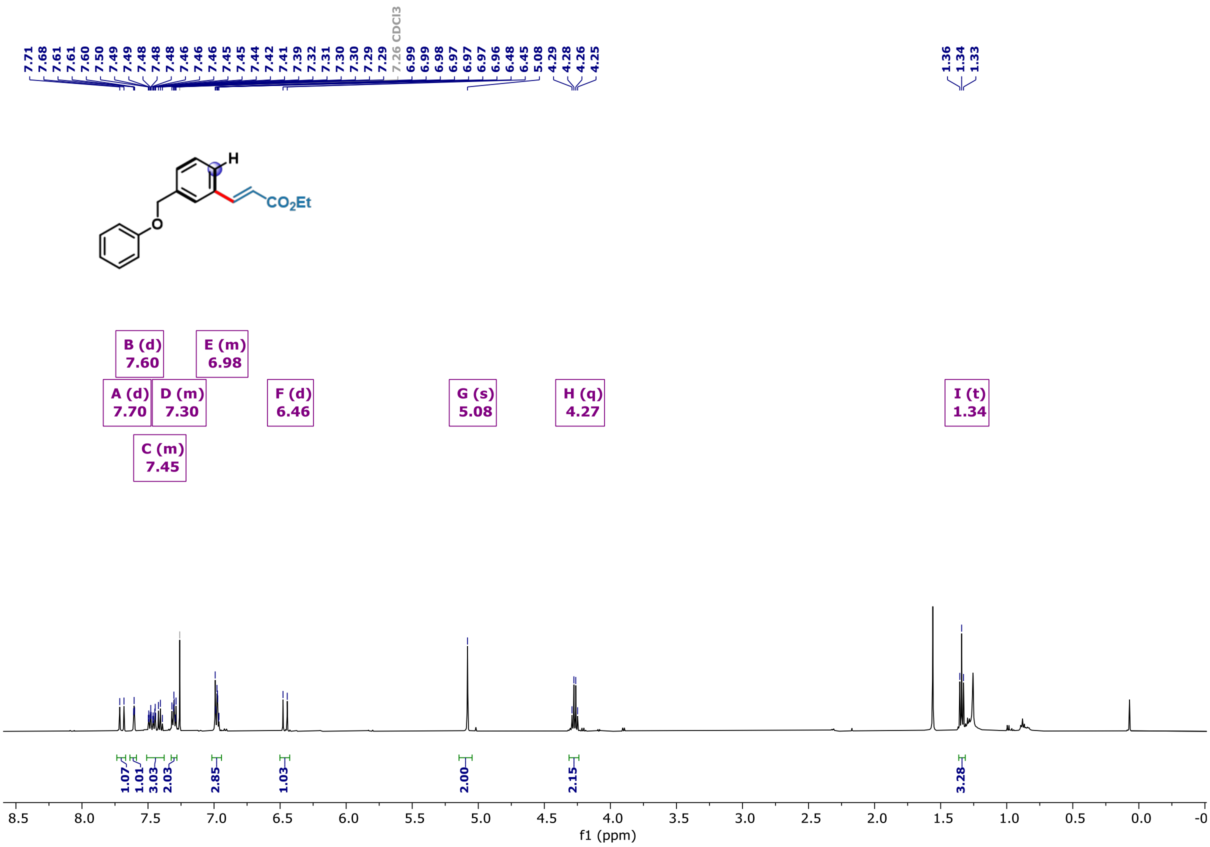

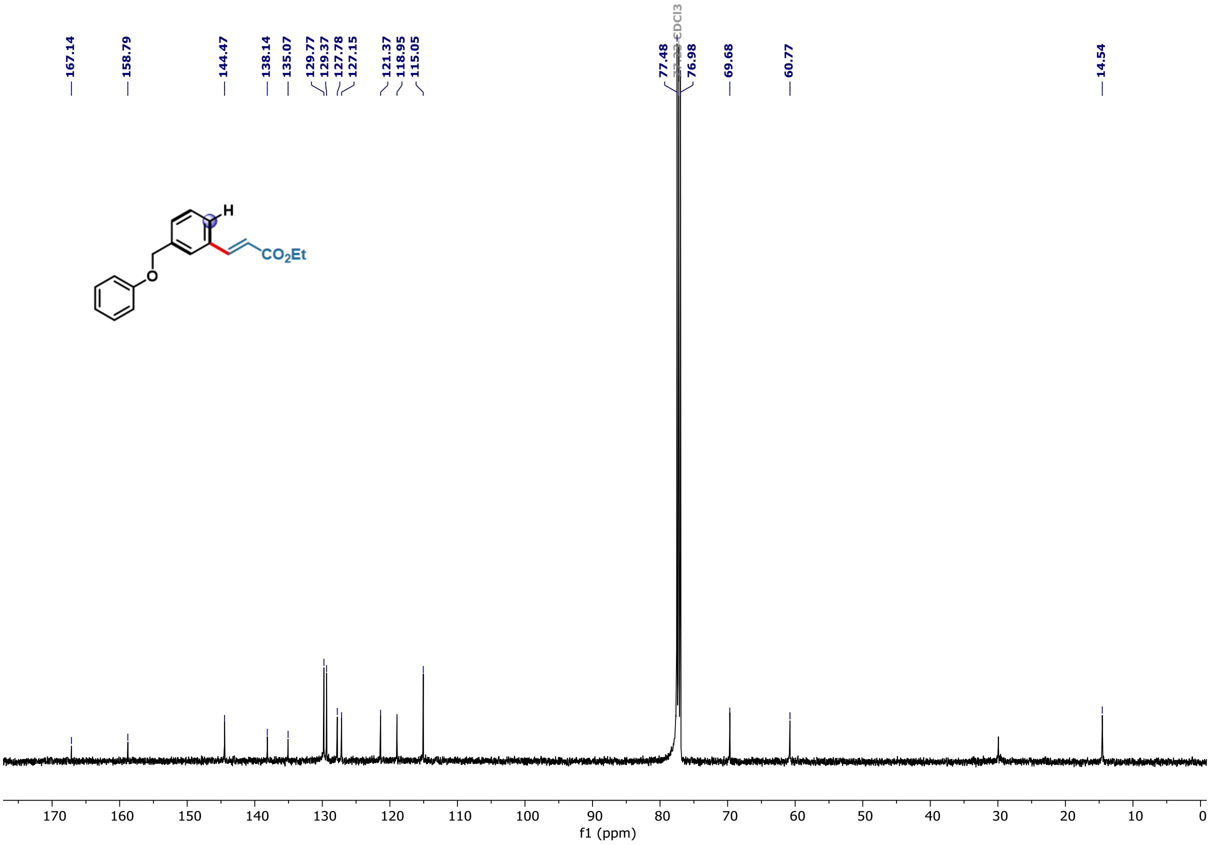

Ethyl (E)-3-(3-((4-ethylphenoxy)methyl)phenyl)acrylate (17)

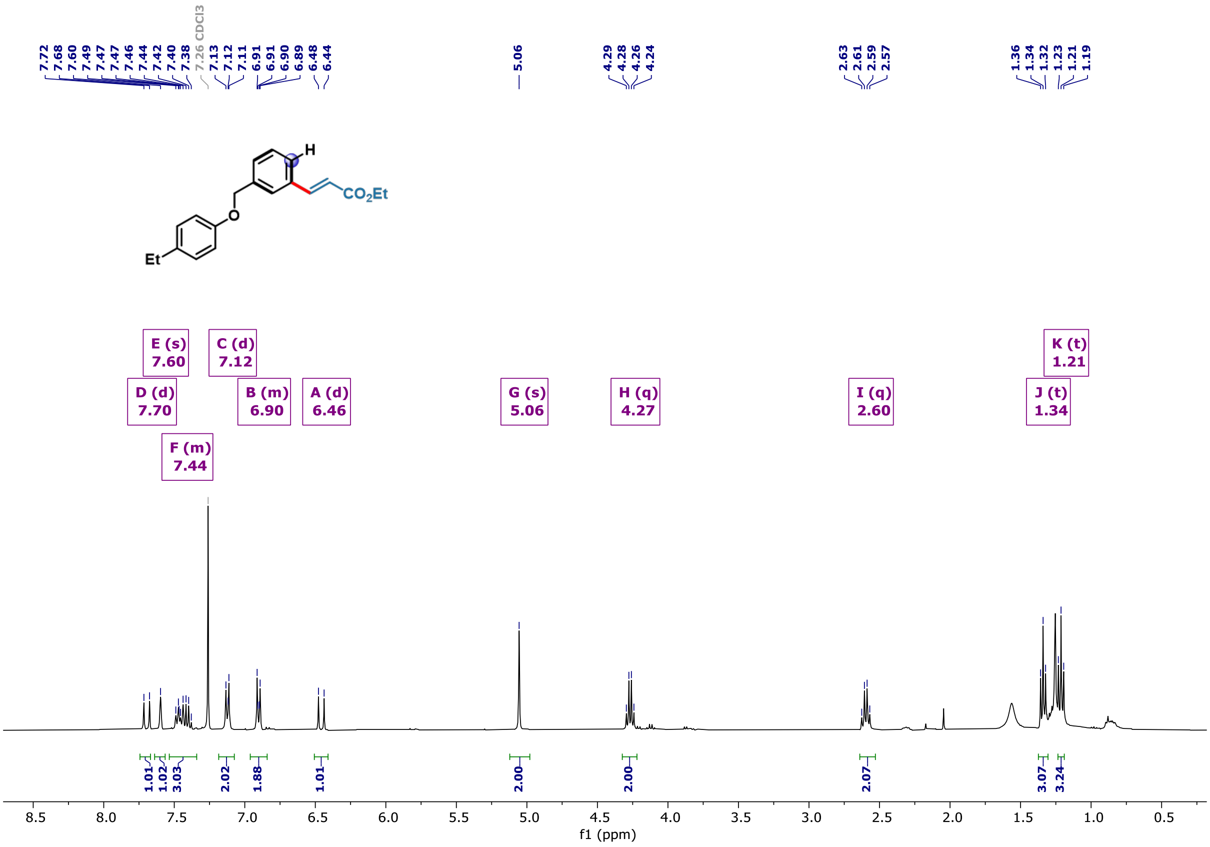

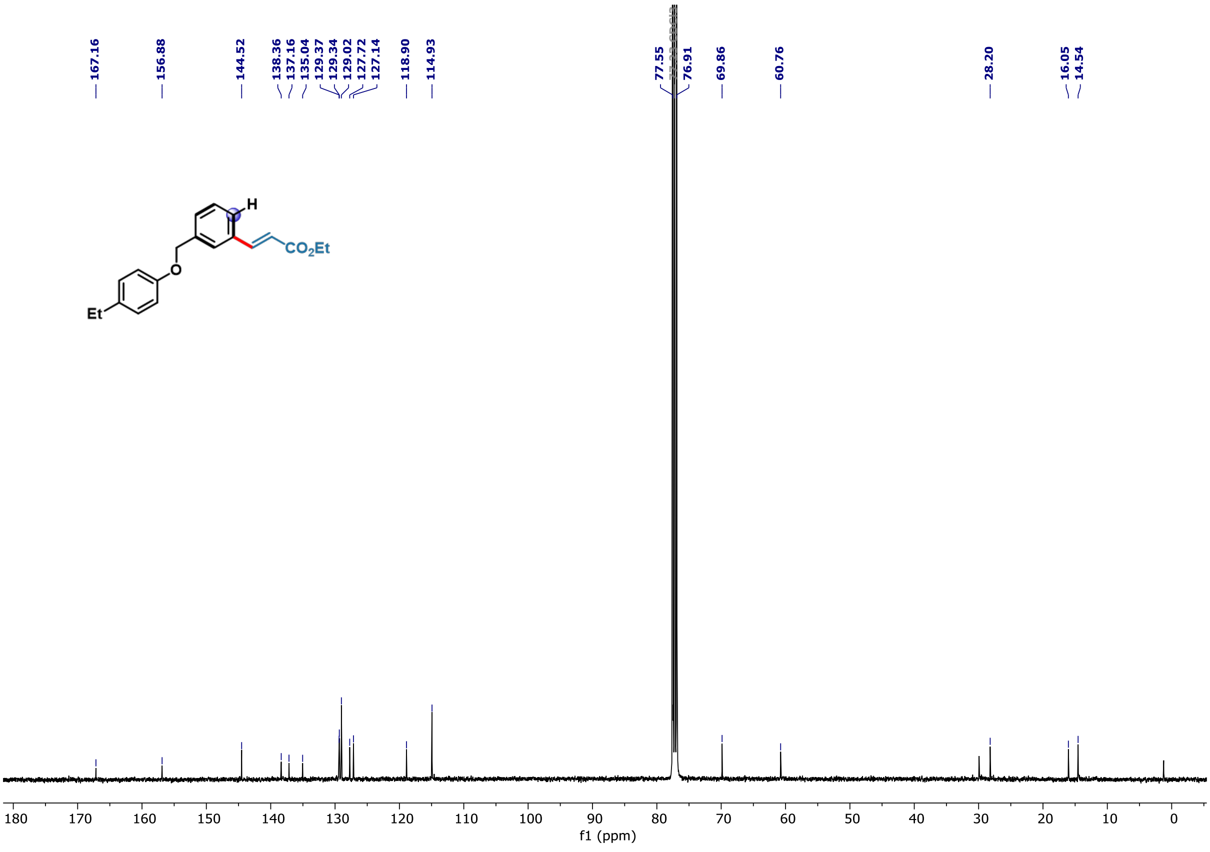

Ethyl (E)-3-(3-((2-isopropyl-5-methylphenoxy)methyl)phenyl)acrylate (18)

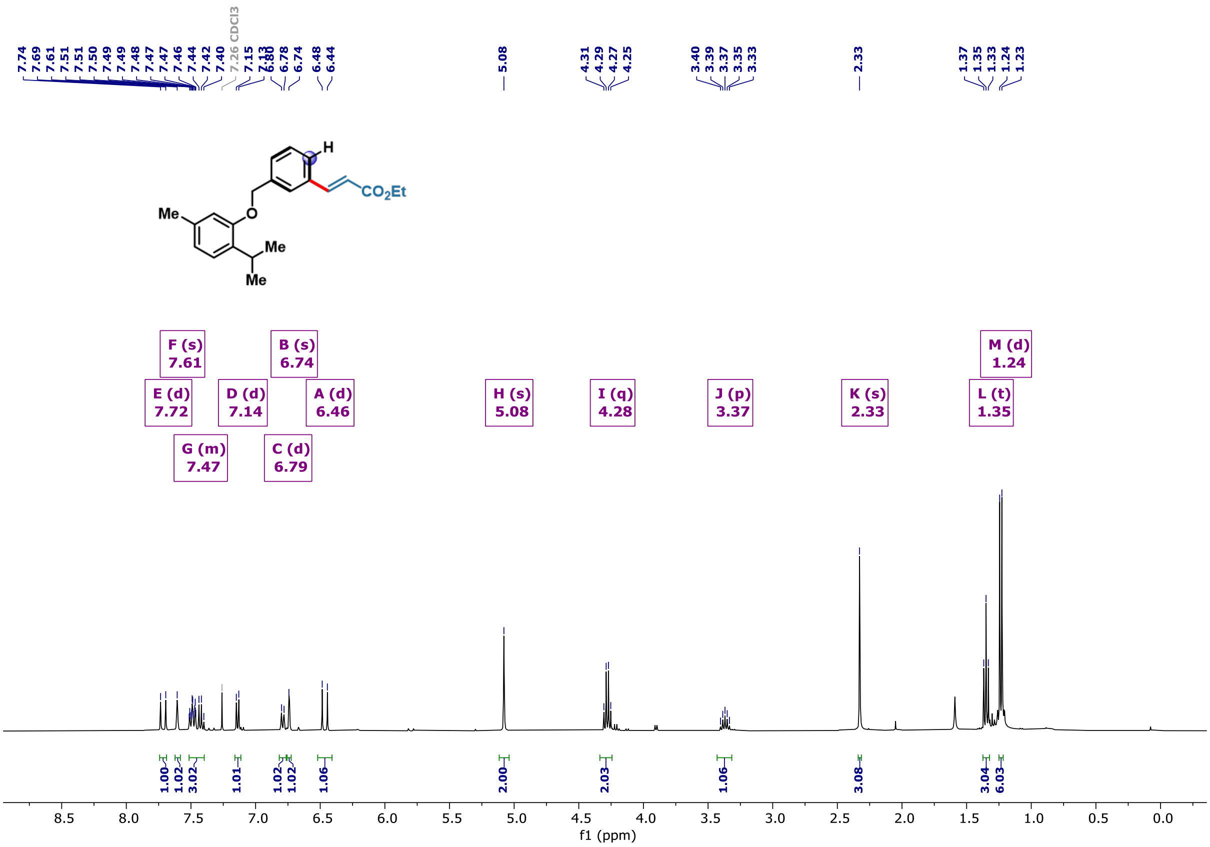

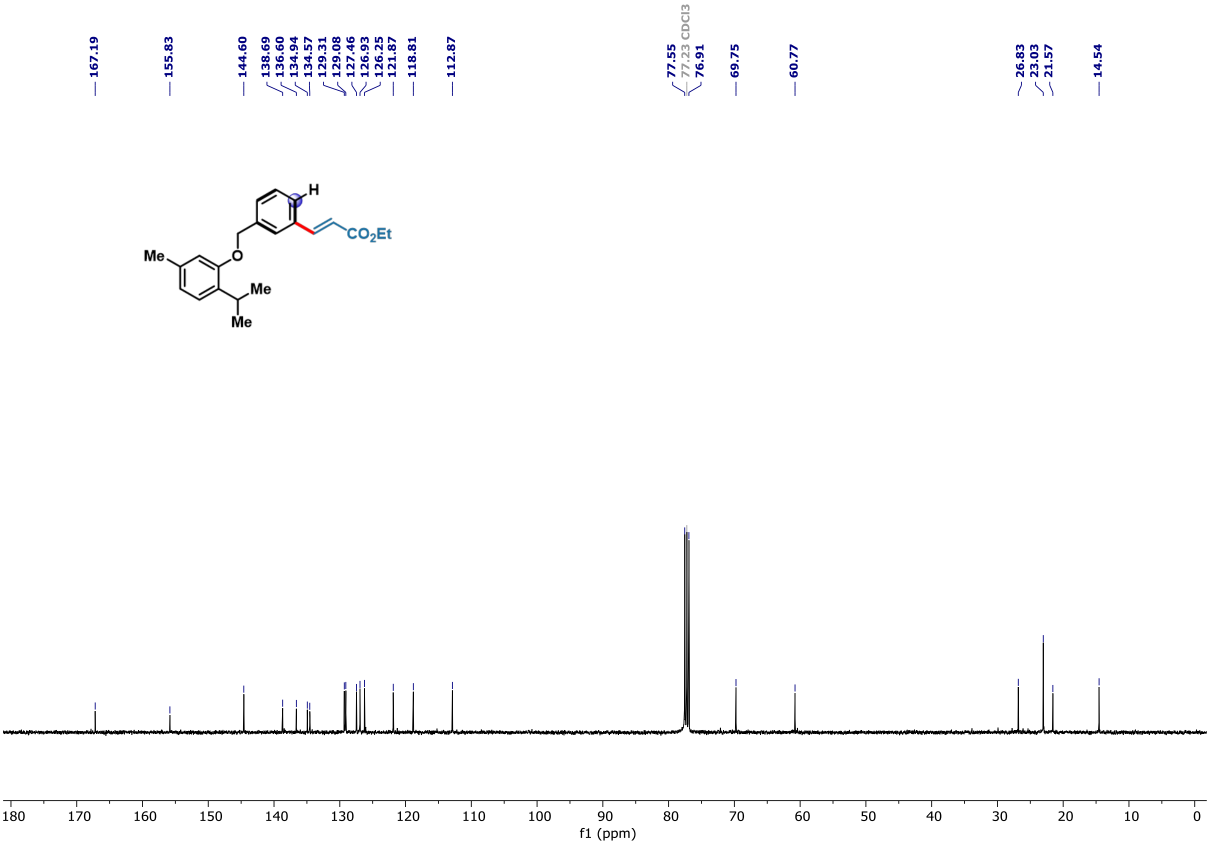

Ethyl (E)-3-(3-((5-isopropyl-2-methylphenoxy)methyl)phenyl)acrylate (19)

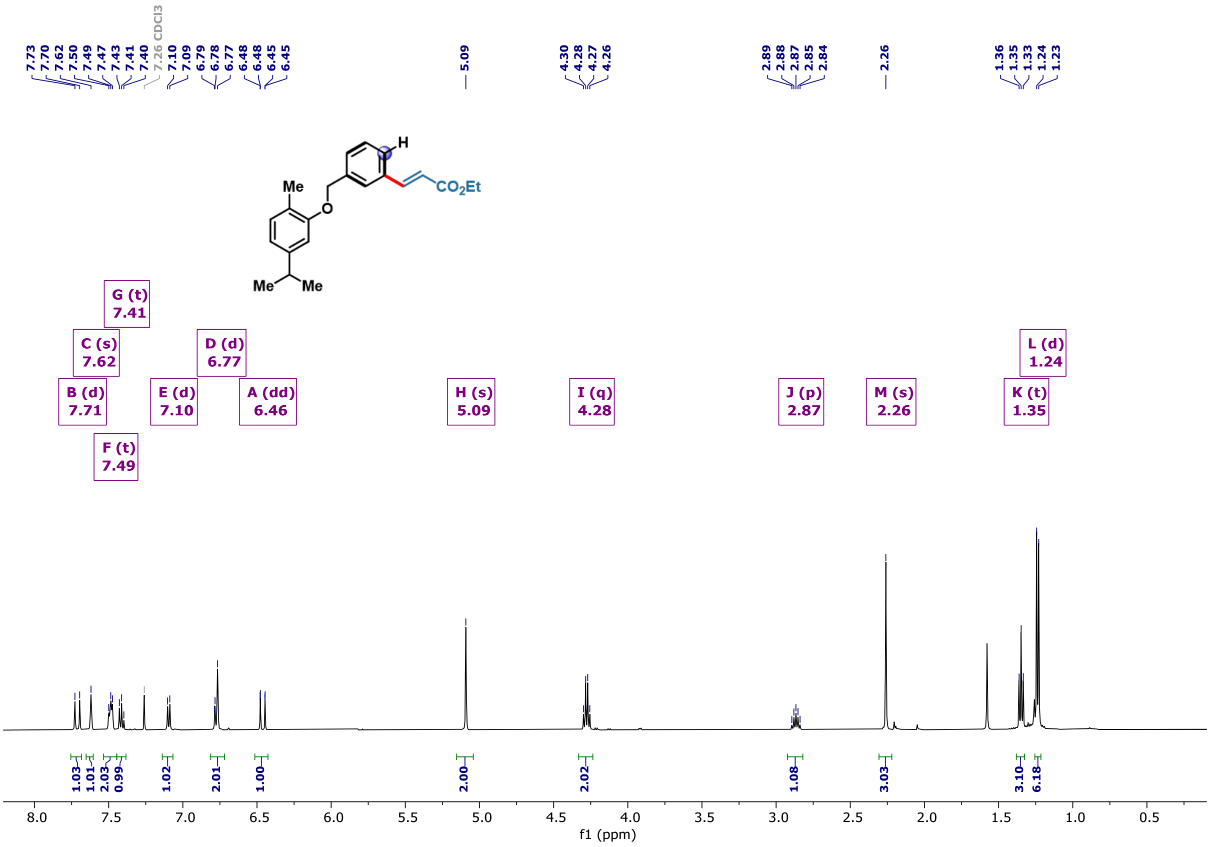

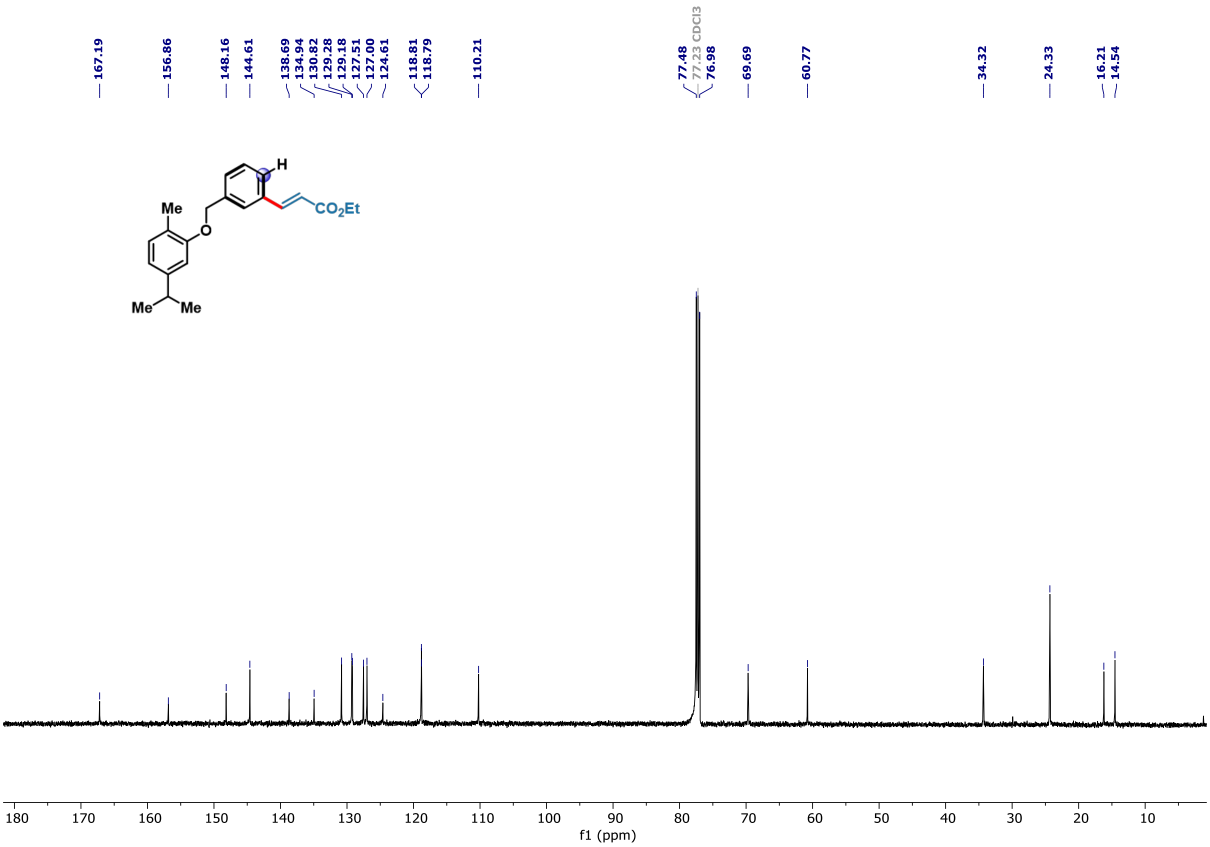

Ethyl (E)-3-(3-((2,6-diisopropylphenoxy)methyl)phenyl)acrylate (20)

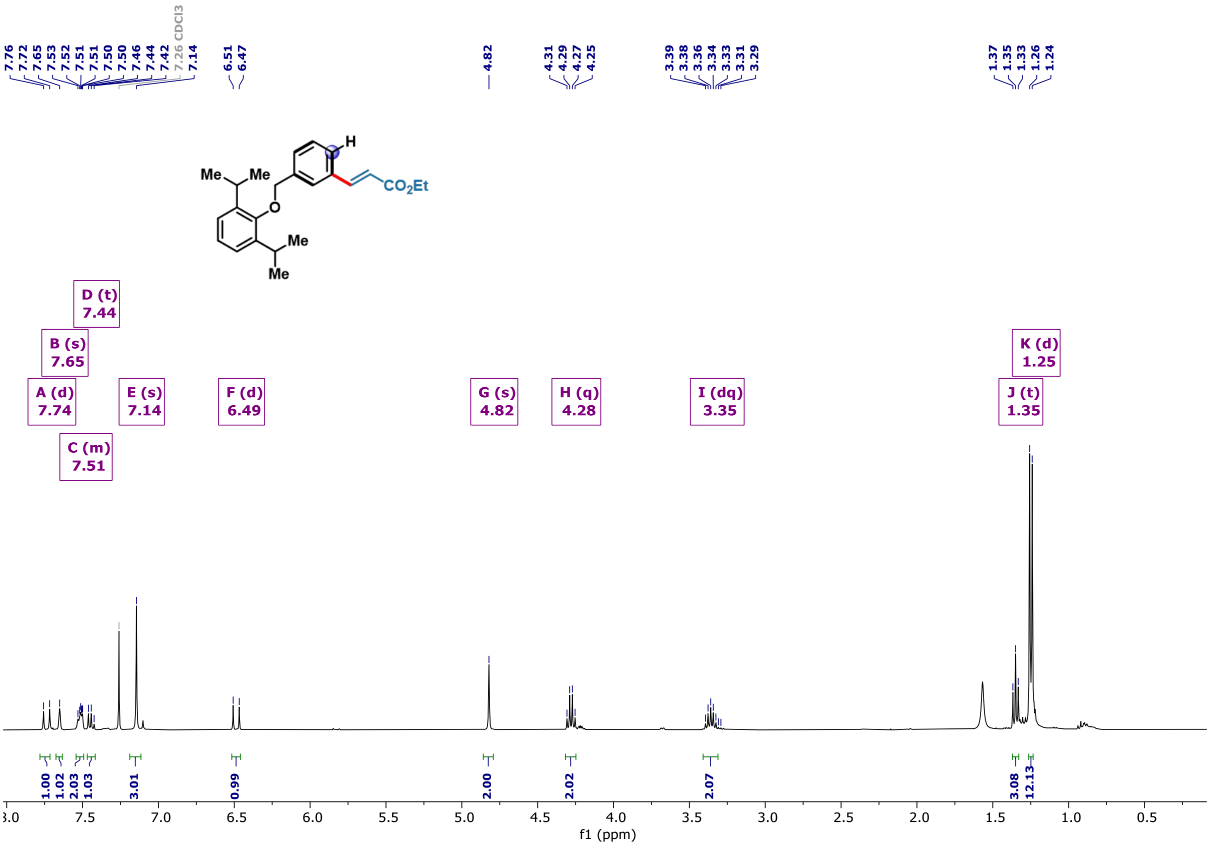

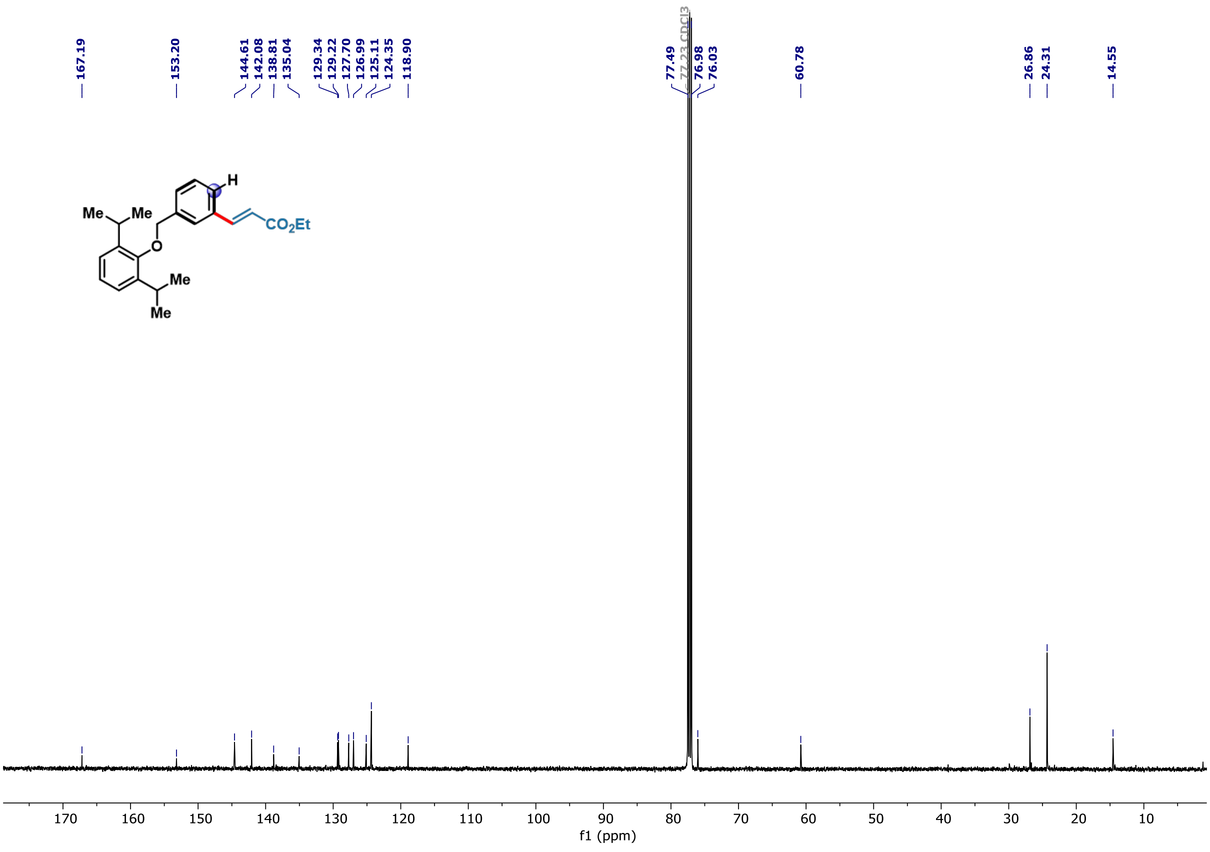

Ethyl (E)-3-(naphthalen-1-yl)acrylate (21)

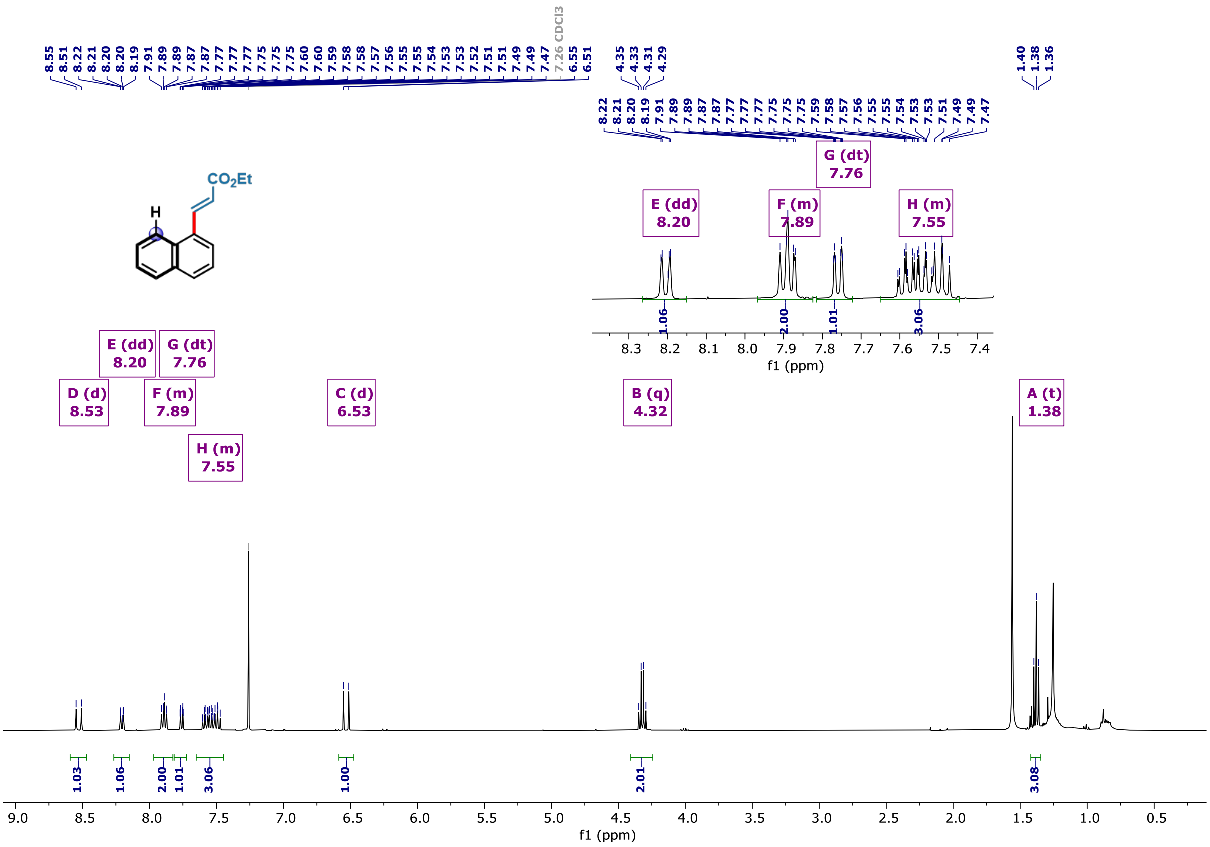

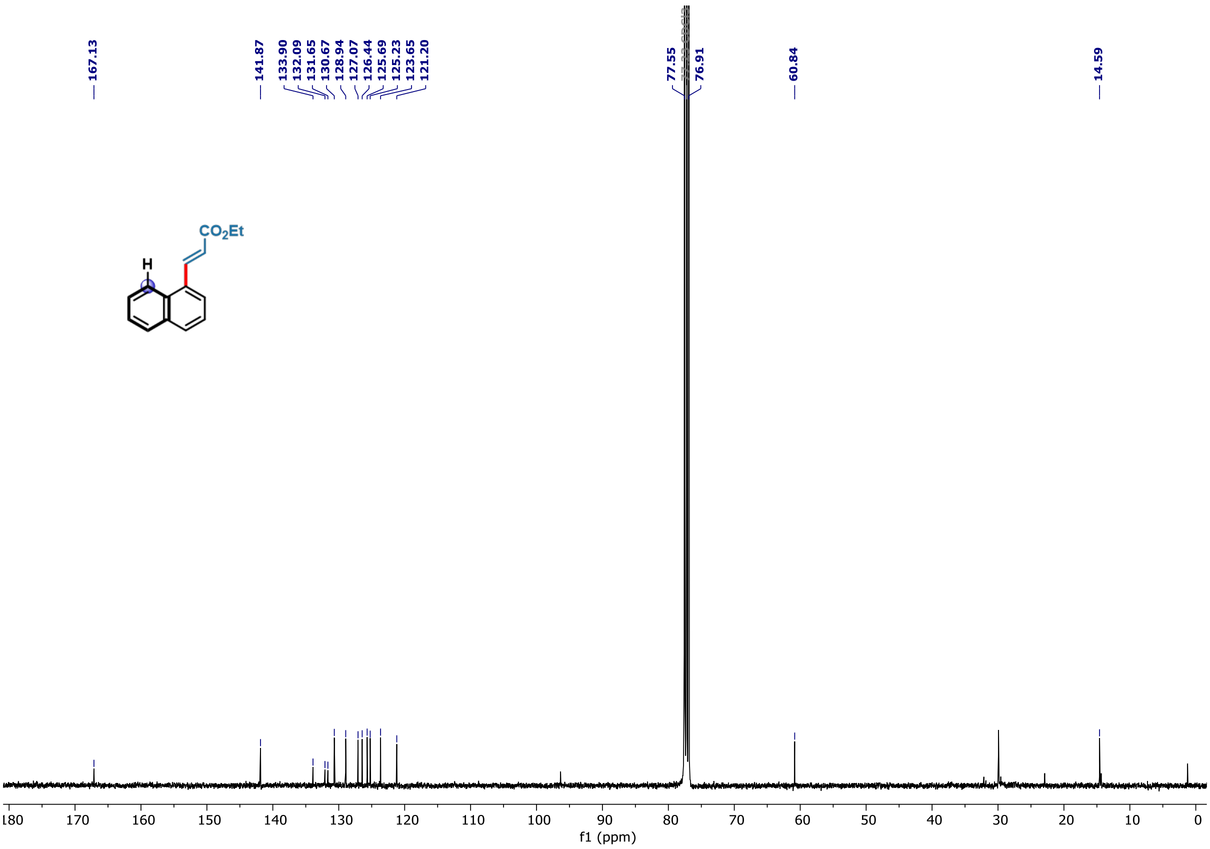

Ethyl (E)-3-(8-(p-tolyl)naphthalen-2-yl)acrylate (22)

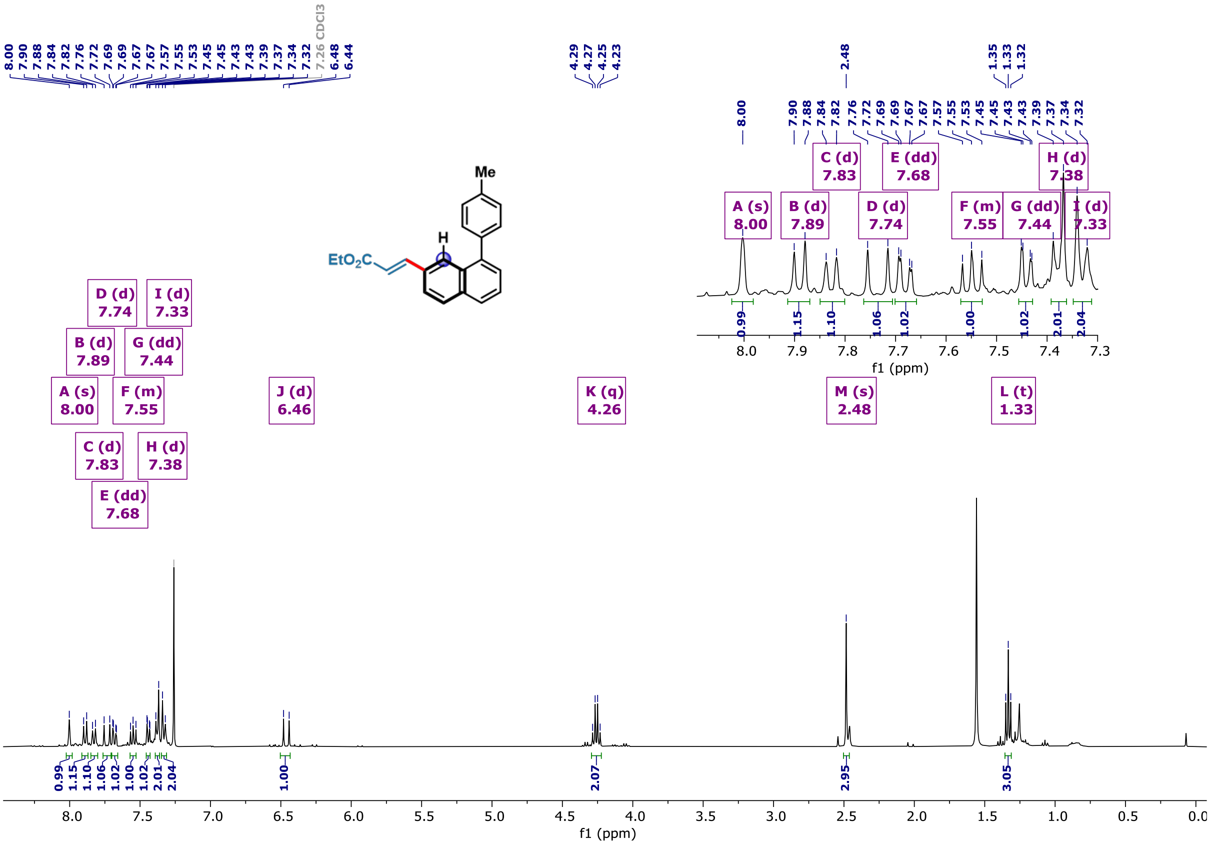

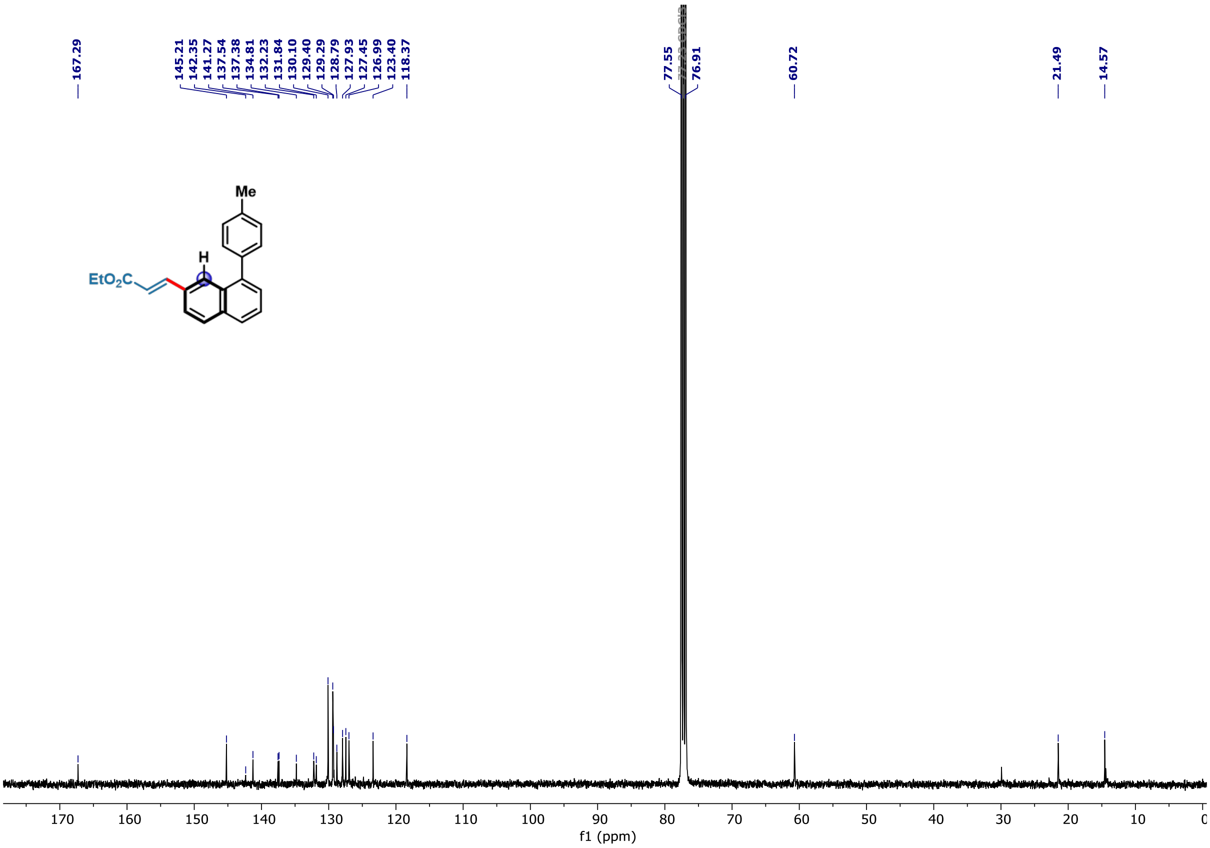

Ethyl (E)-3-(o-tolyl)acrylate (23)

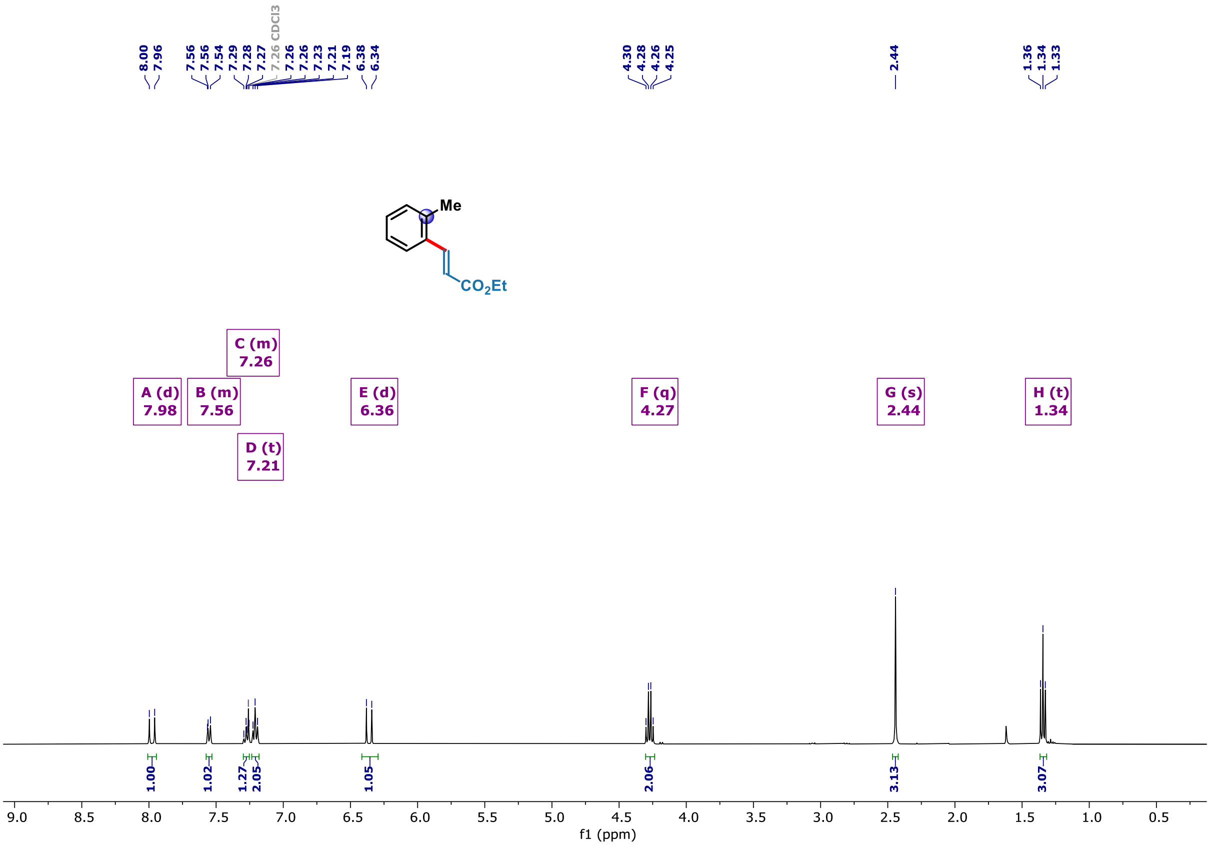

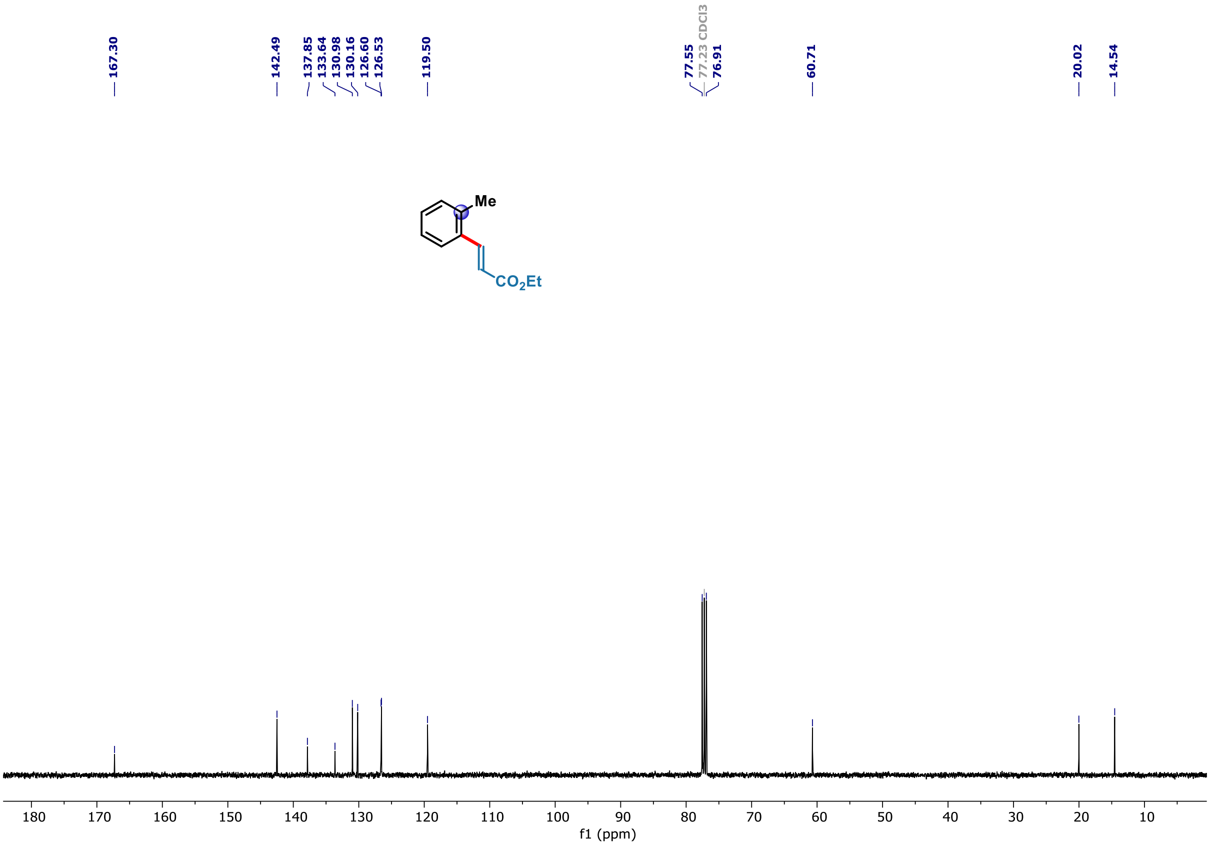

Ethyl 2-(3a-methyl-3-oxo-1,3,3a,4,5,6-hexahydroisobenzofuran-1-yl)acetate & ethyl-2-(1-oxo-2-oxaspiro[4.5]decan-3-yl)acetate (inseparable mixture of products) (23’)

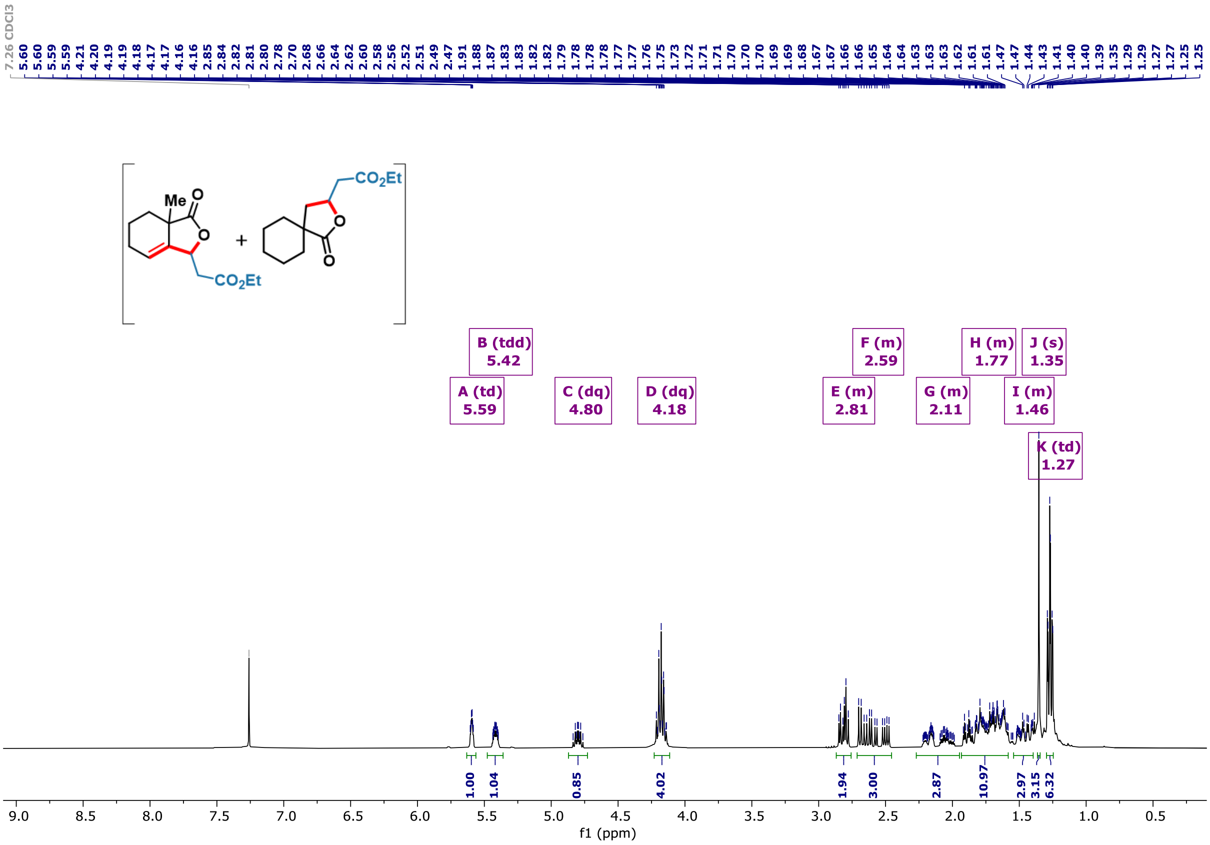

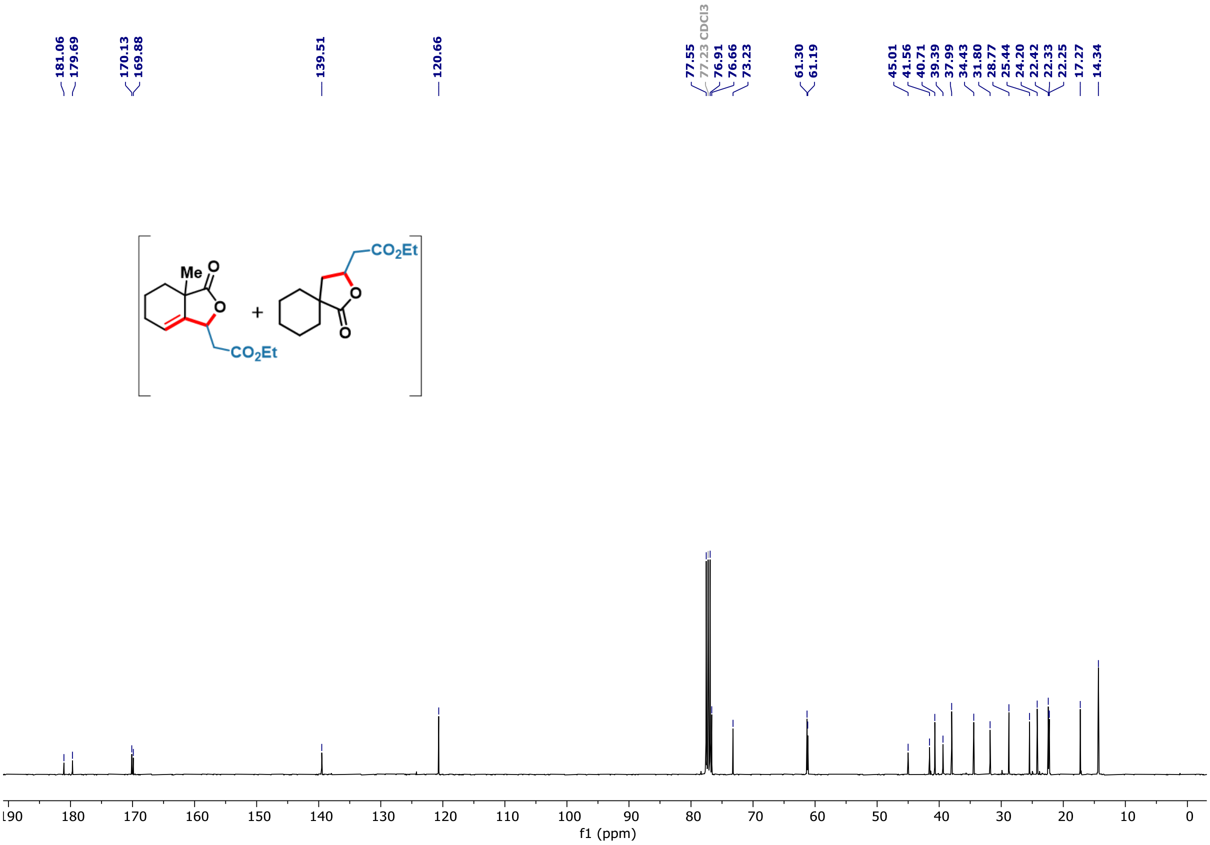

Ethyl (E)-3-(2-propylphenyl)acrylate (24)

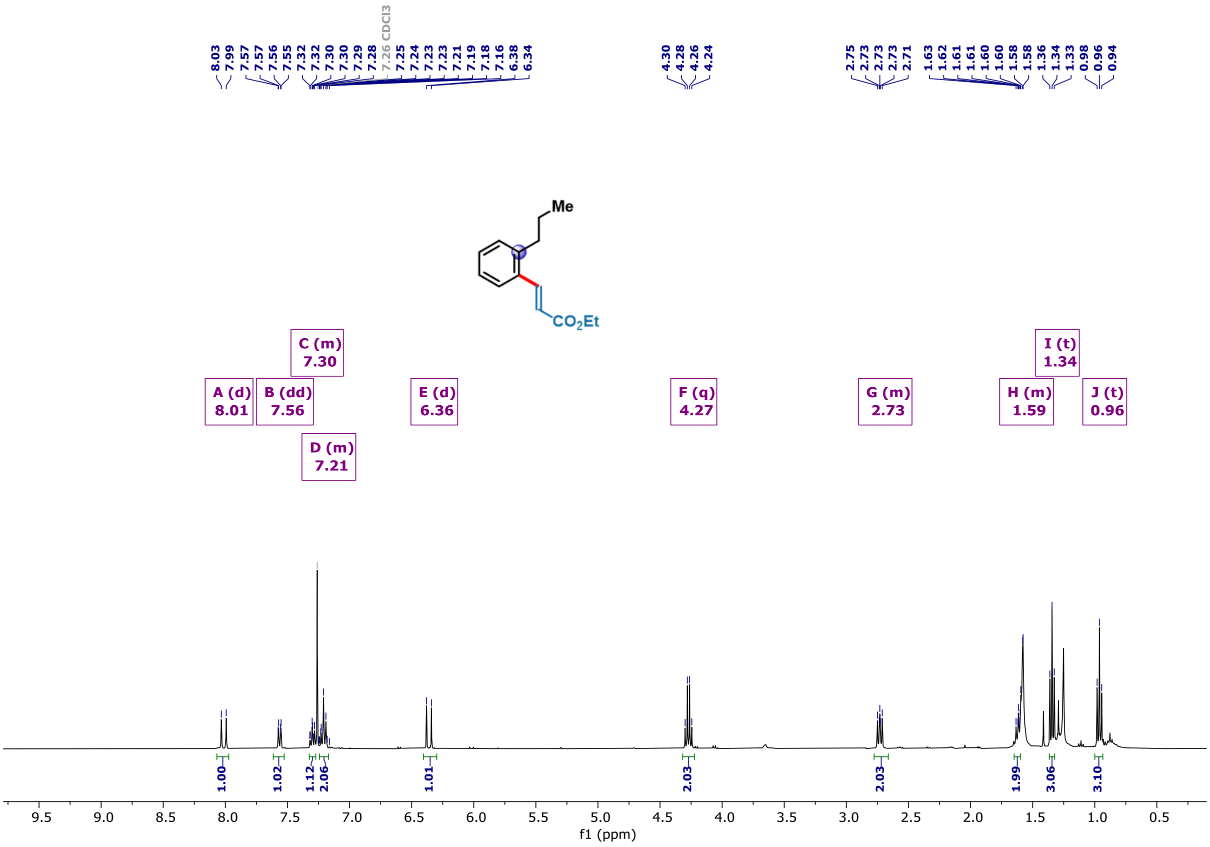

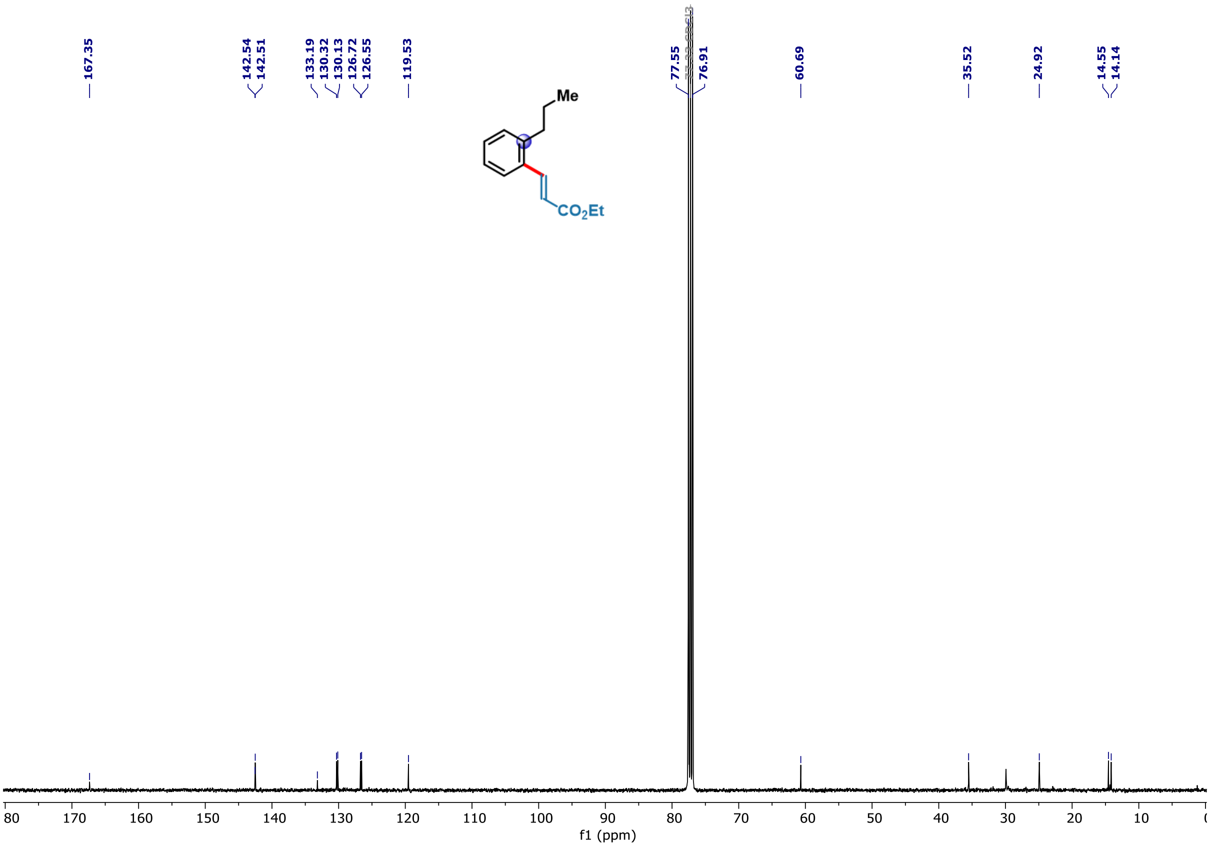

Ethyl 2-(3-oxo-3a-propyl-1,3,3a,4,5,6-hexahydroisobenzofuran-1-yl)acetate (24')

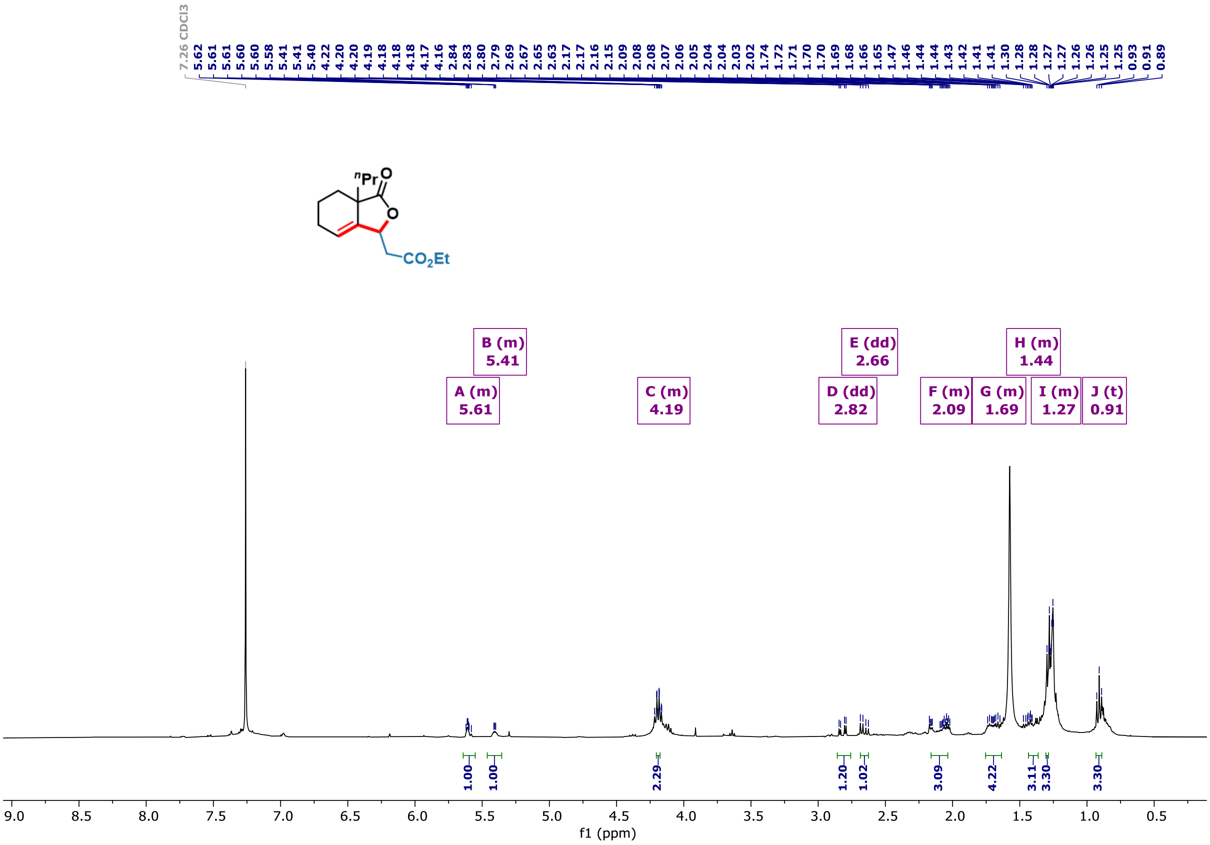

Methyl (E)-3-(3-(tert-butyl)phenyl)acrylate (25)

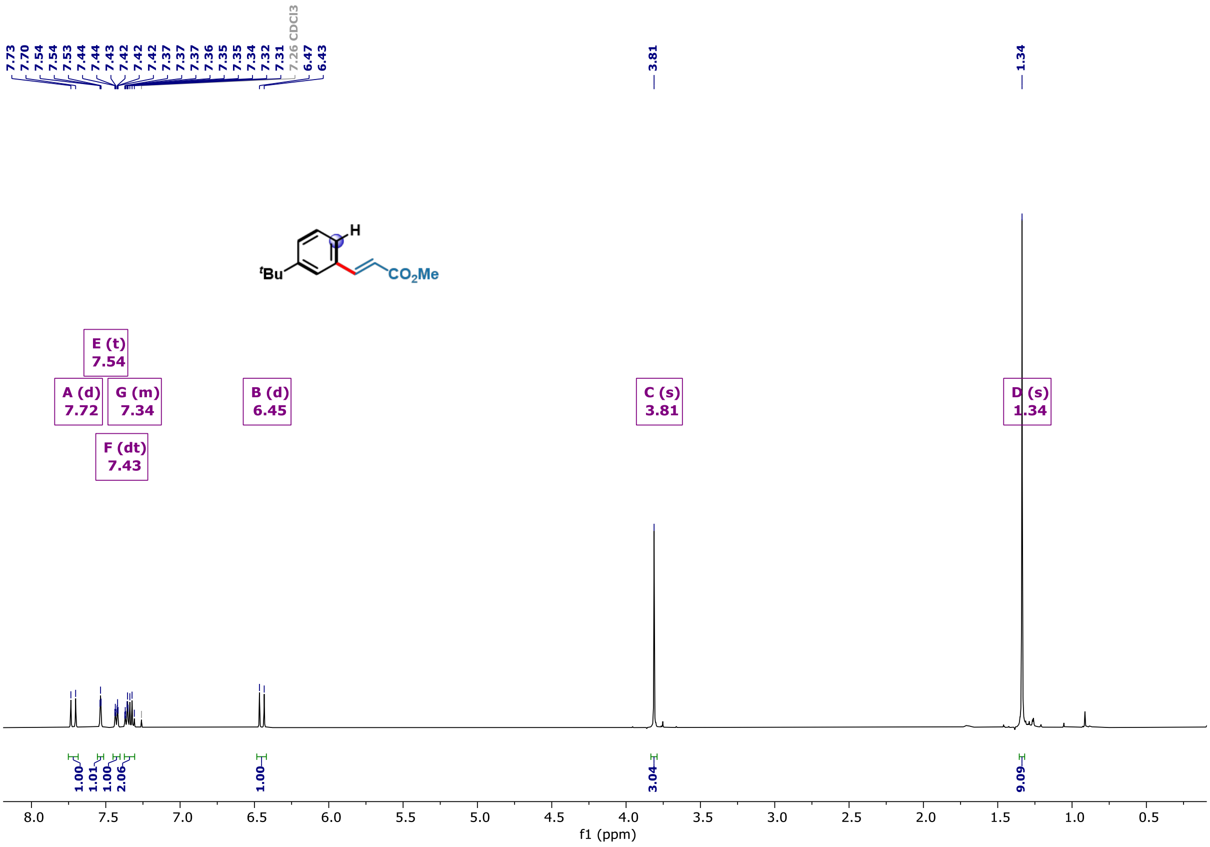

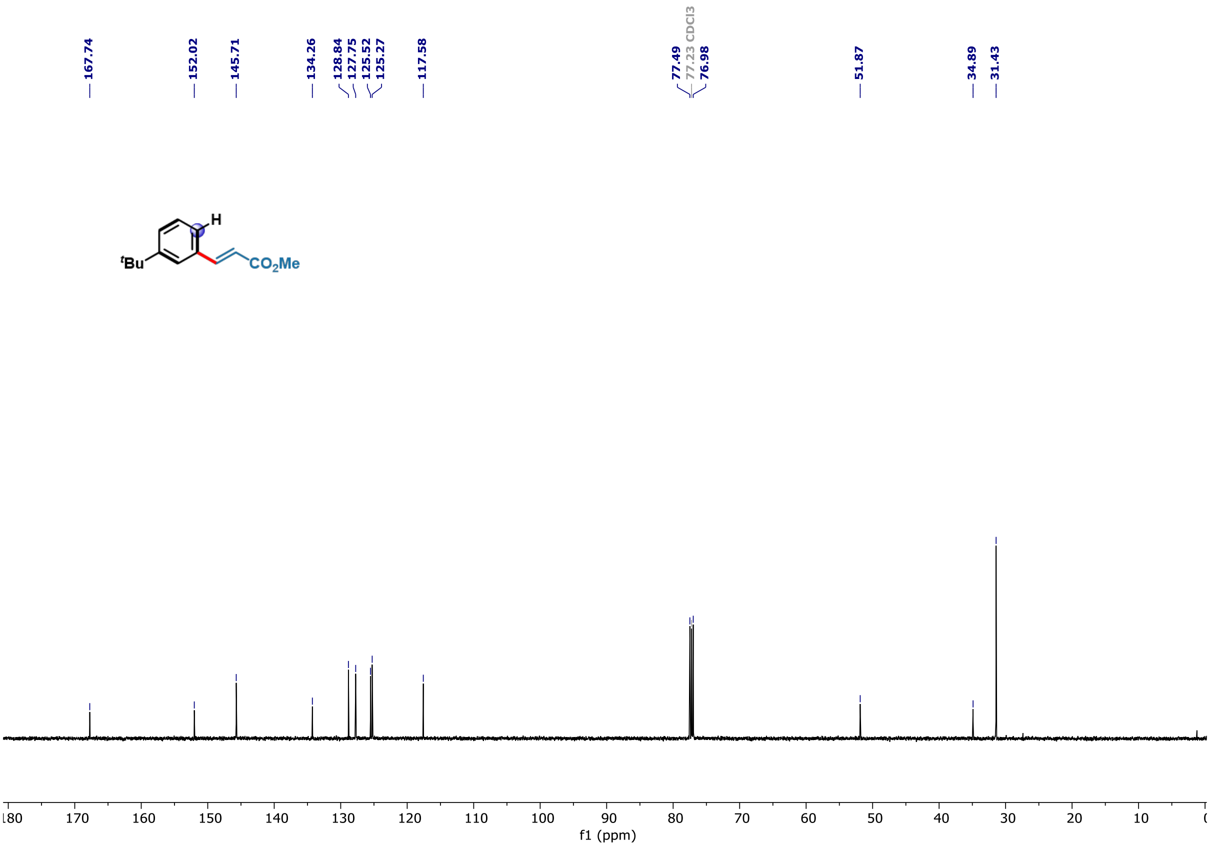

Butyl (E)-3-(3-(tert-butyl)phenyl)acrylate (26)

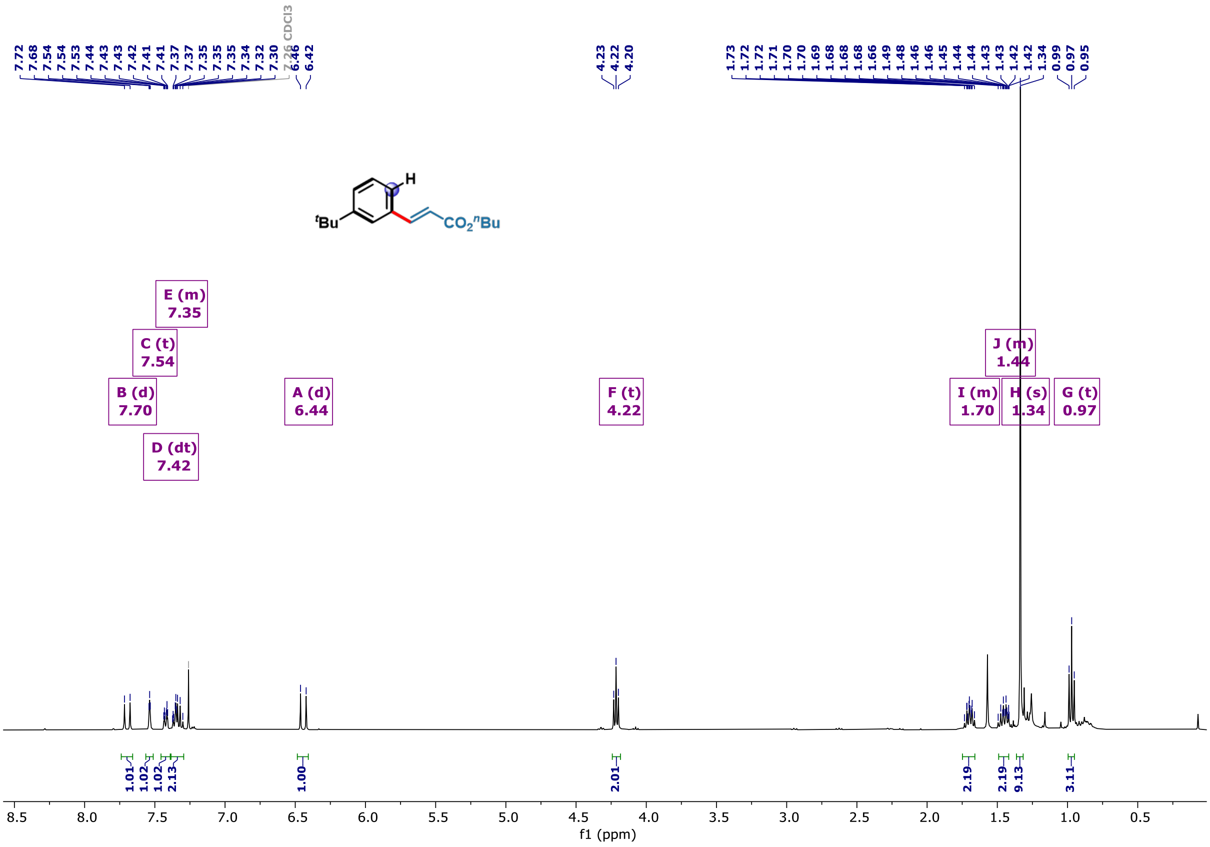

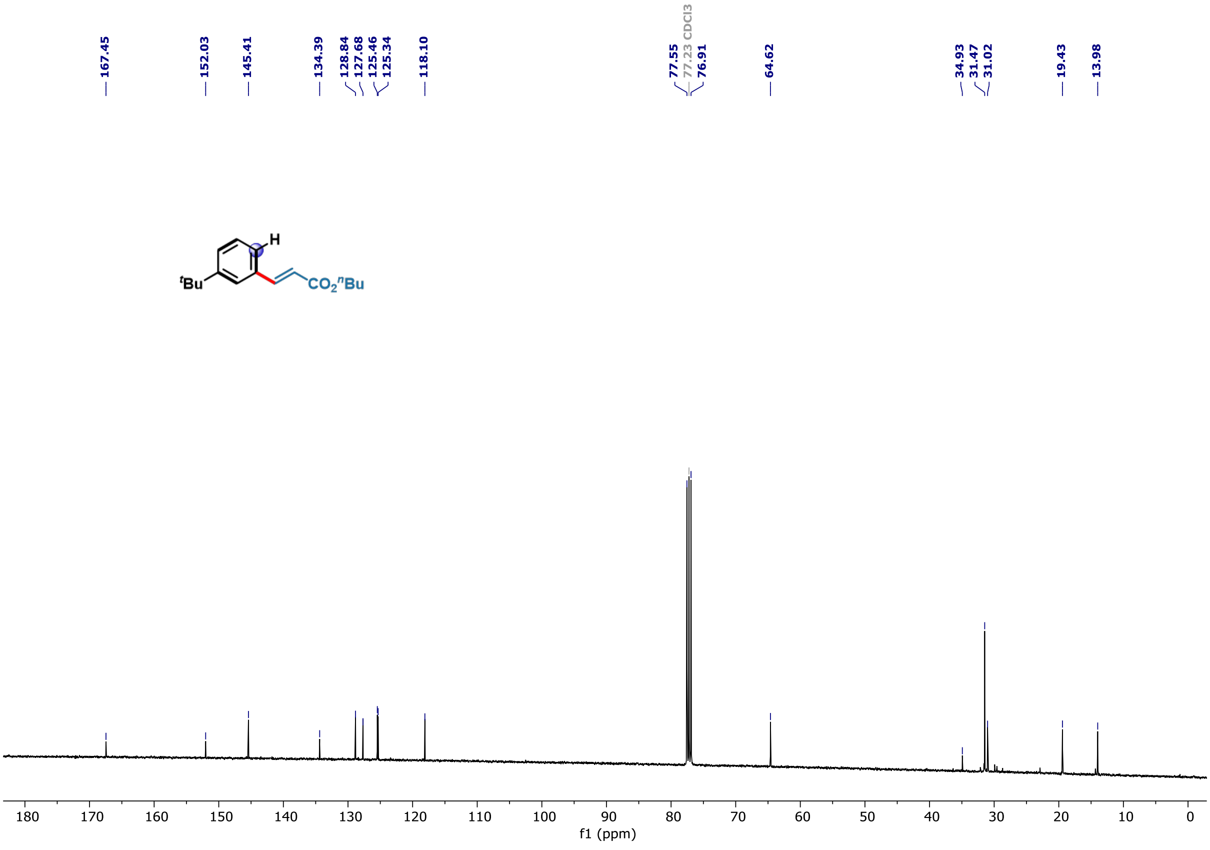

Cyclohexyl (E)-3-(3-(tert-butyl)phenyl)acrylate (27)

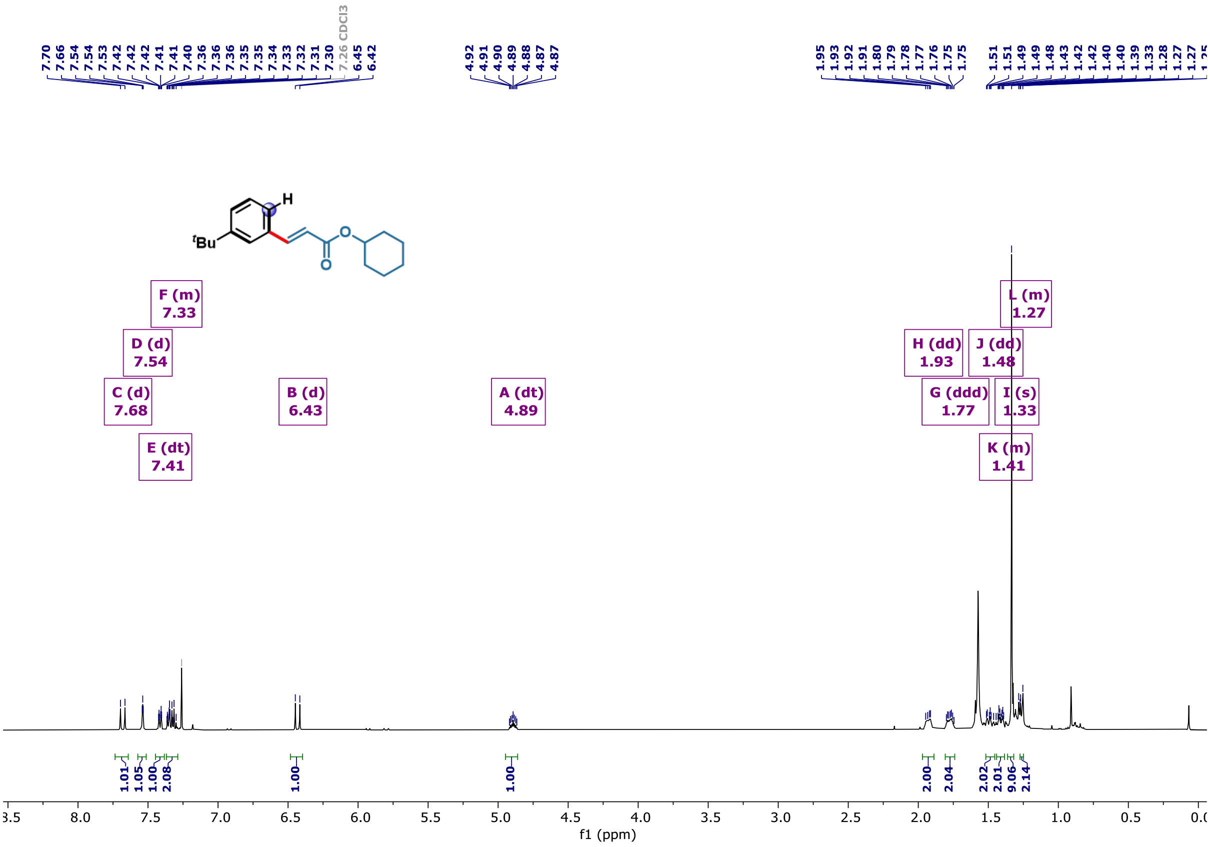

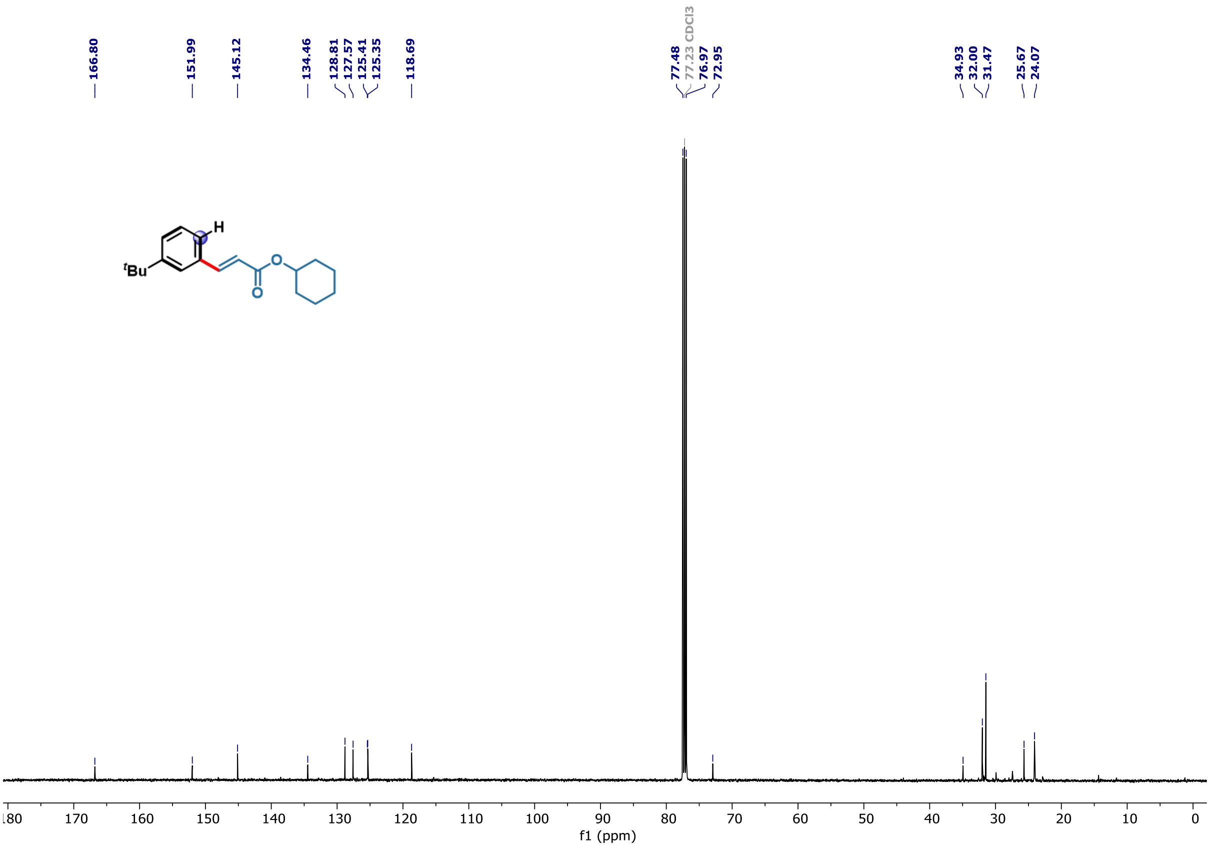

Benzyl cinnamate (28)

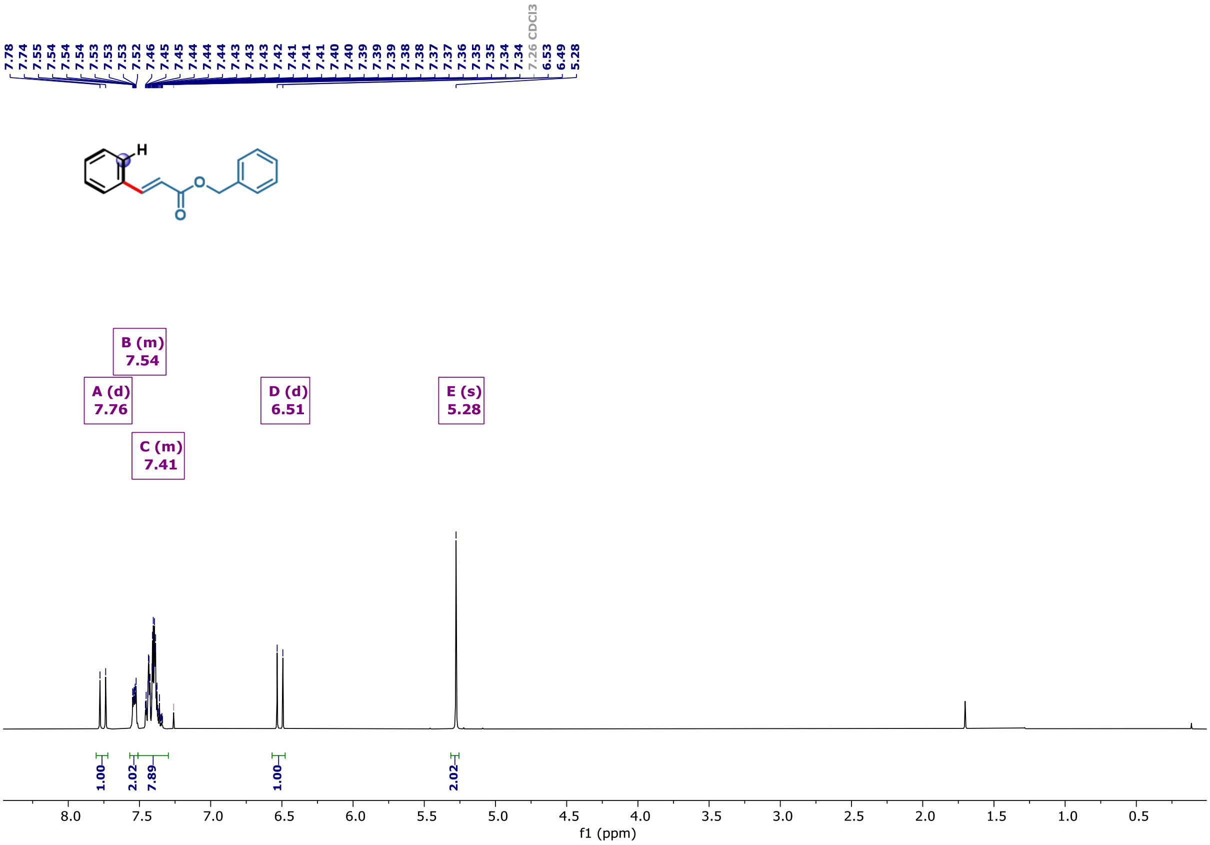

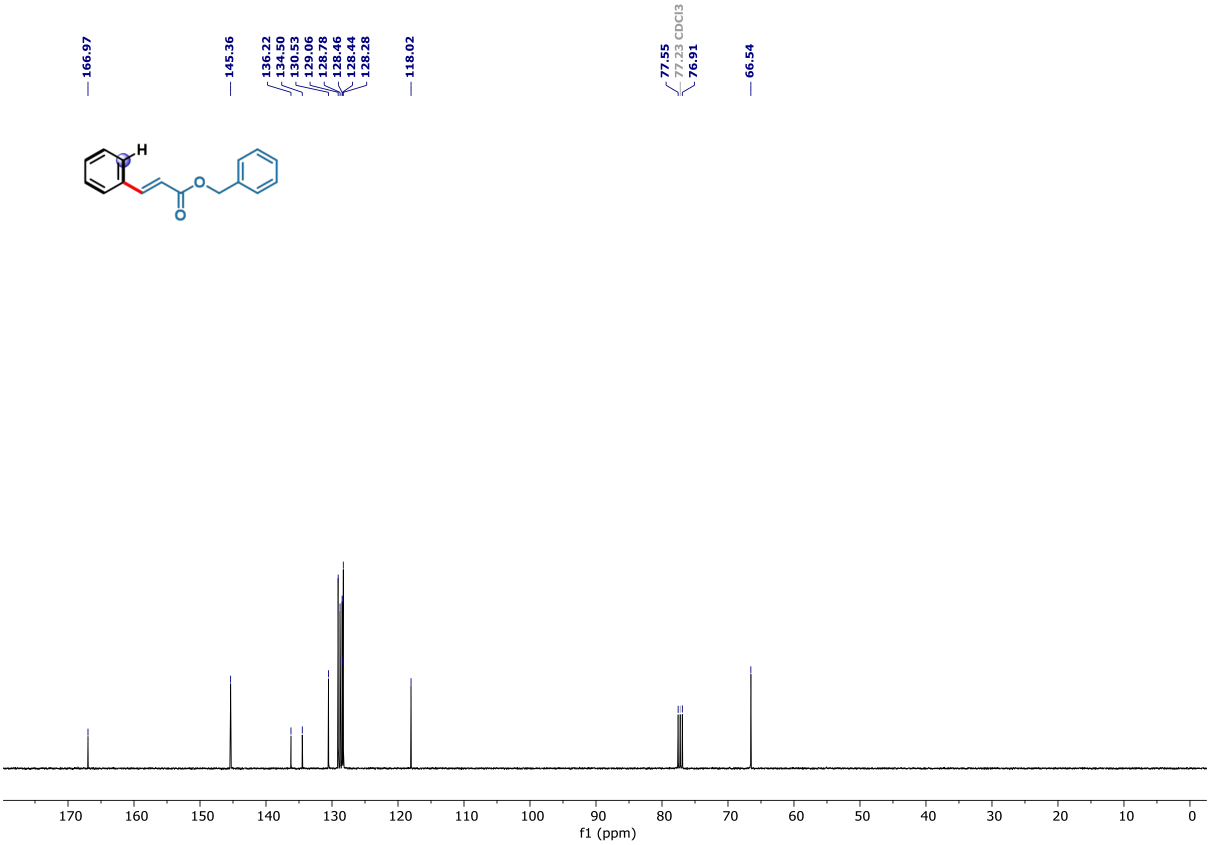

Benzyl (E)-3-(naphthalen-1-yl)acrylate (29)

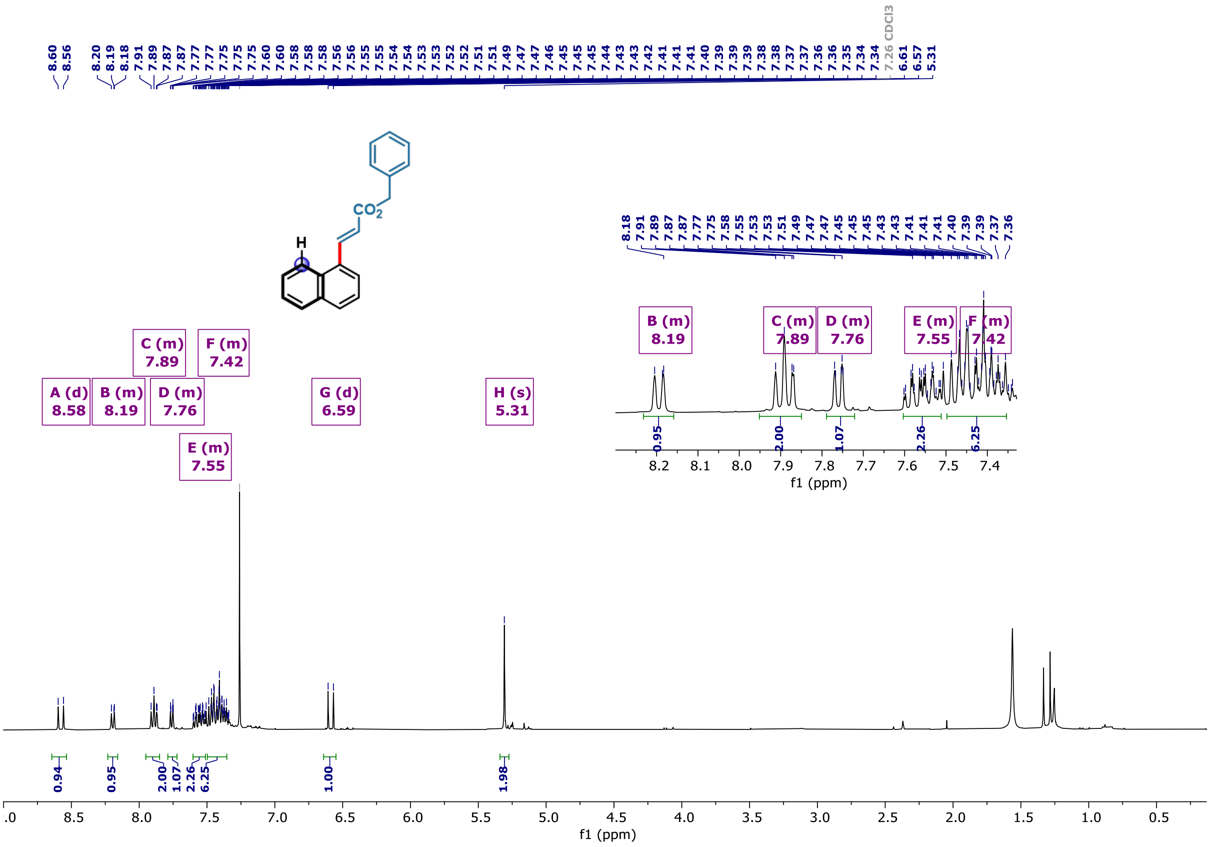

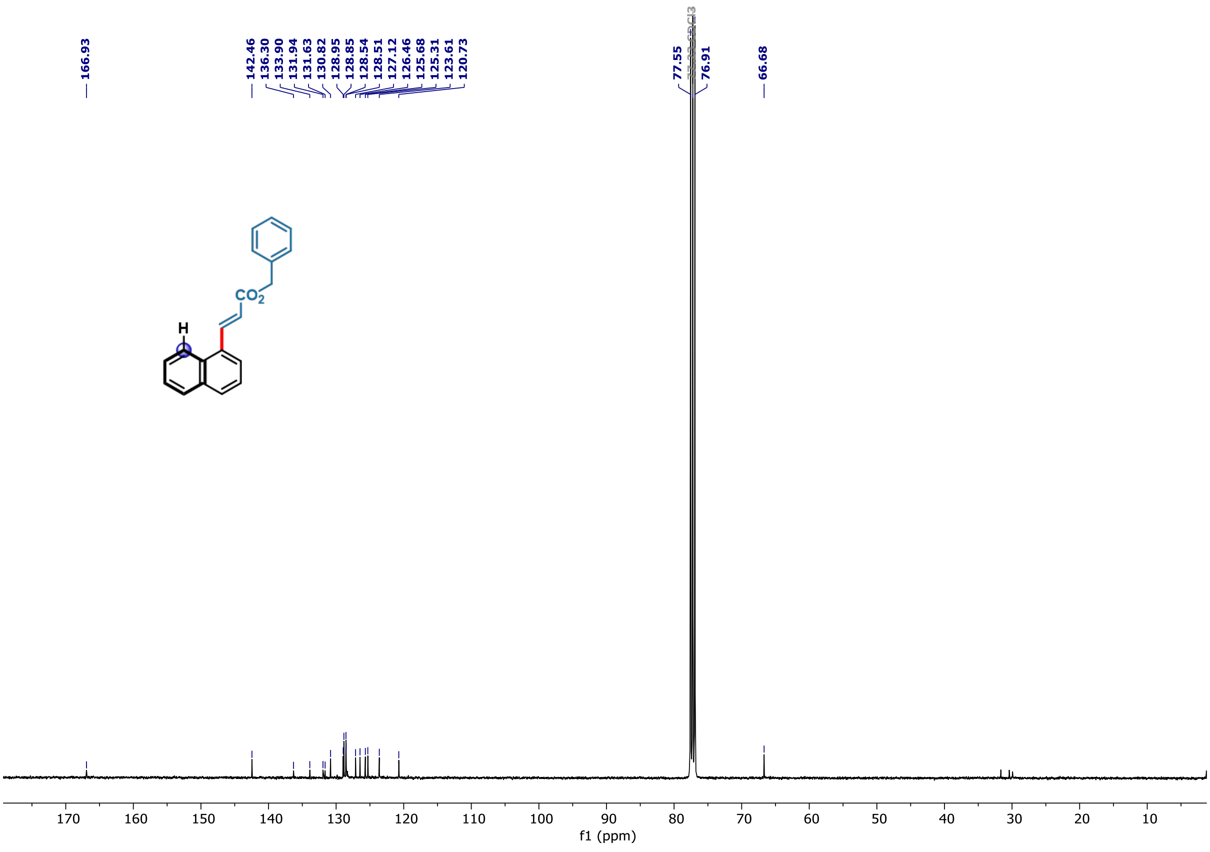

4-chlorobenzyl (E)-3-(3-(tert-butyl)phenyl)acrylate (30)

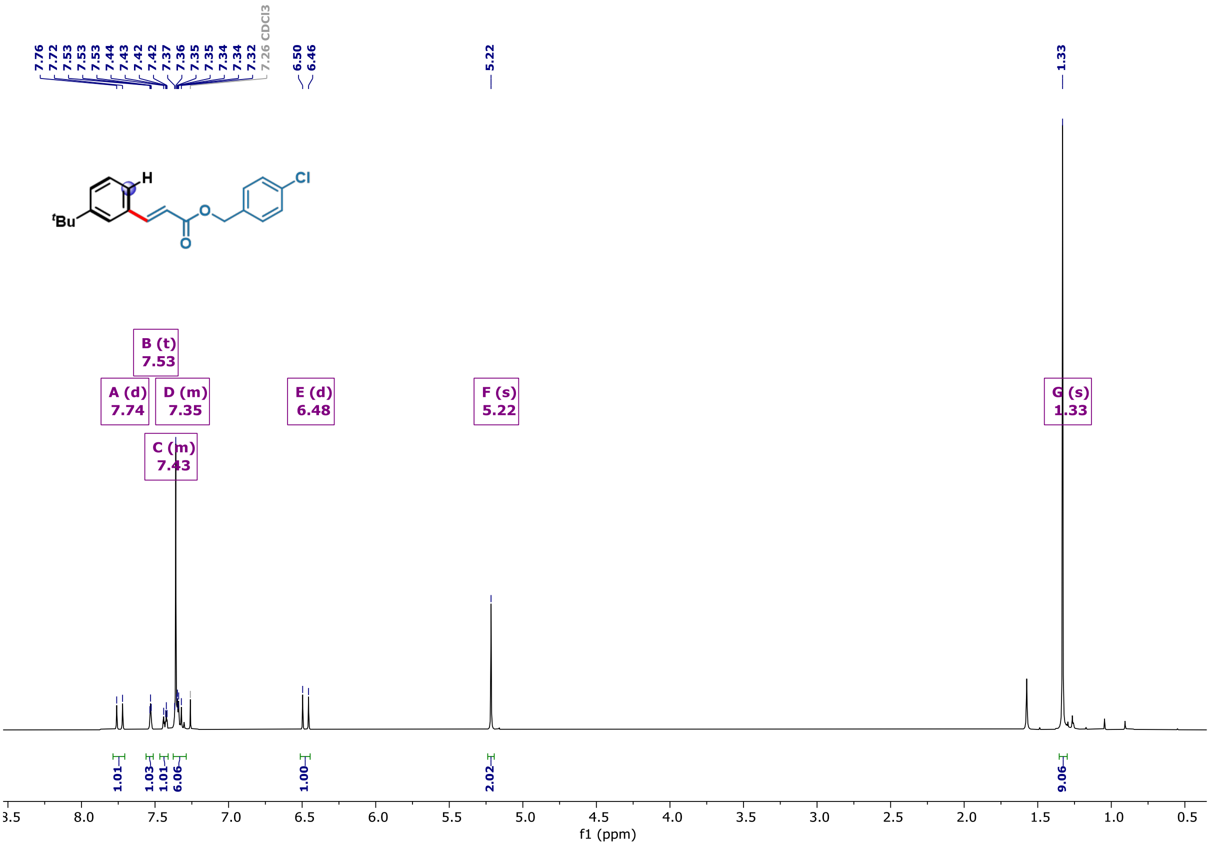

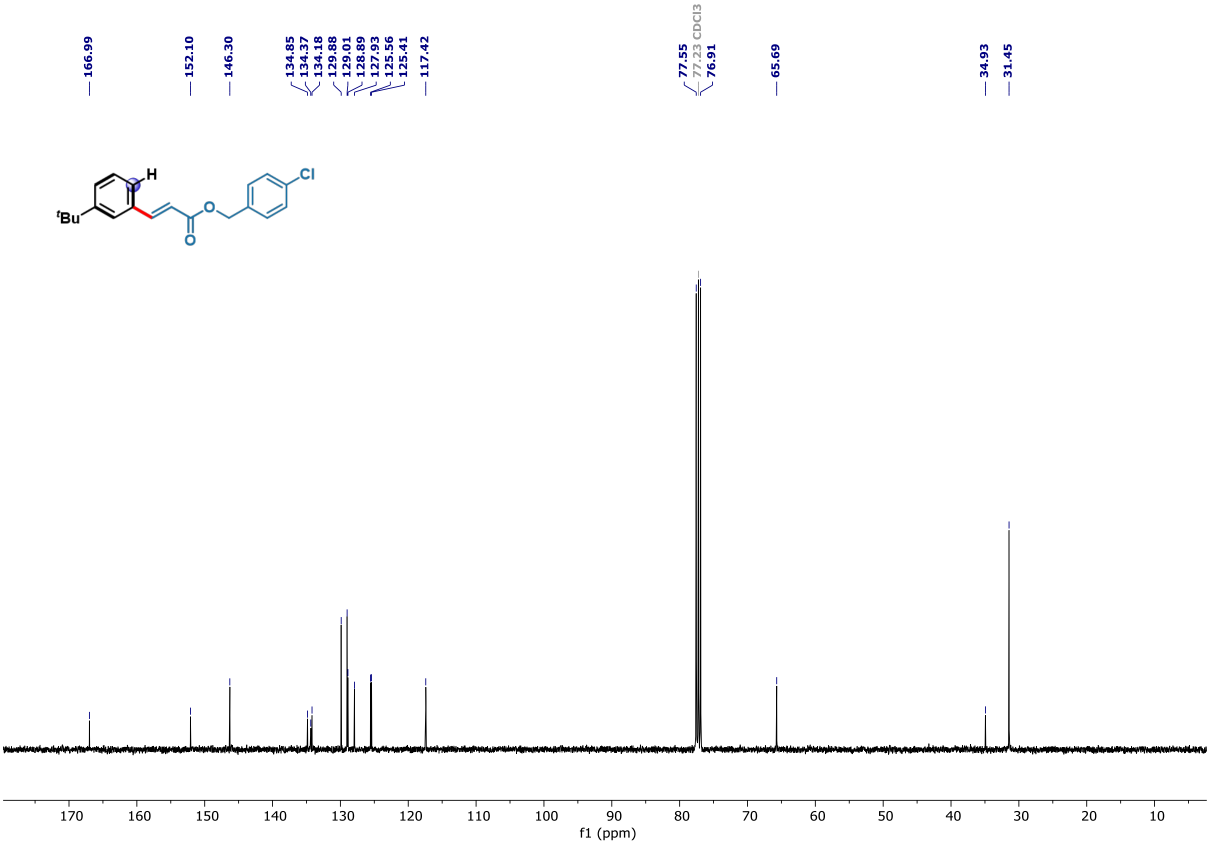

2,2,3,3,4,4,5,5,6,6,7,7-dodecafluoroheptyl (E)-3-(3-(tert-butyl)phenyl)acrylate (31)

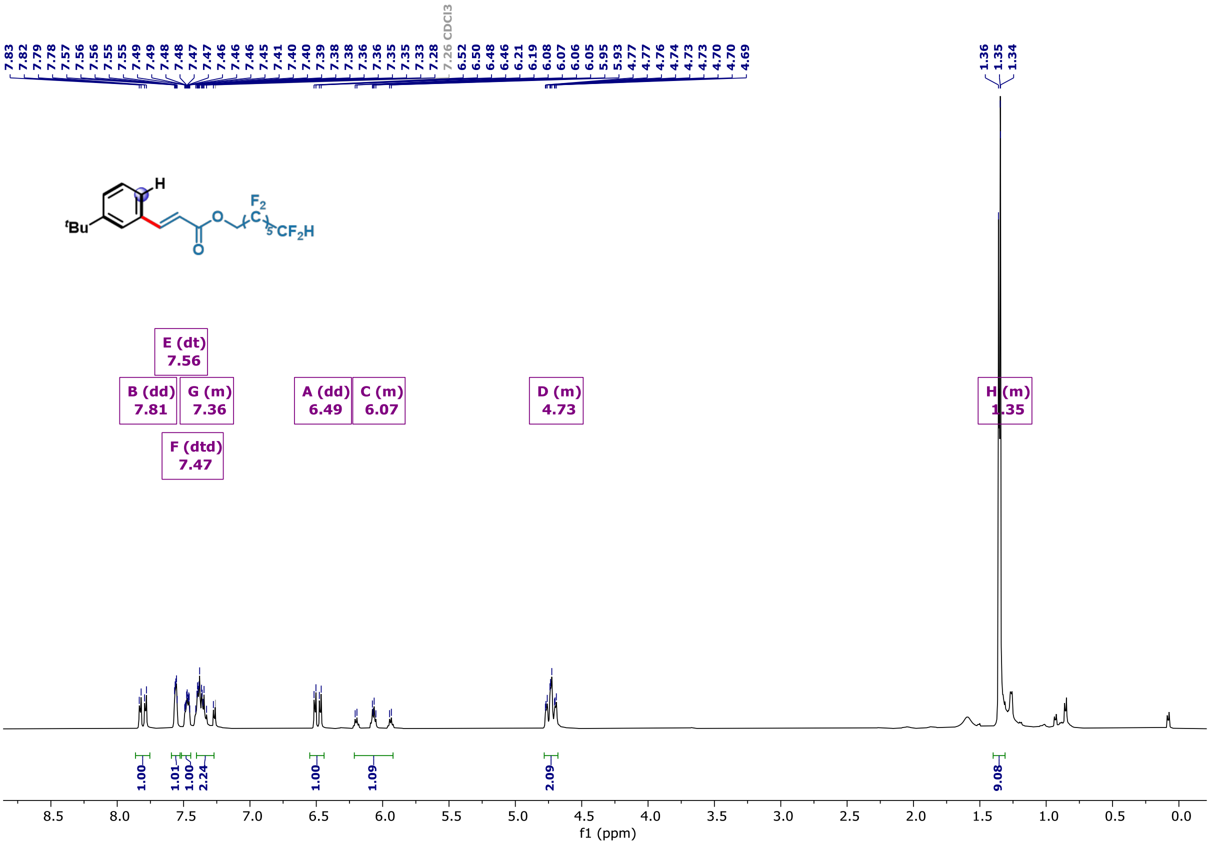

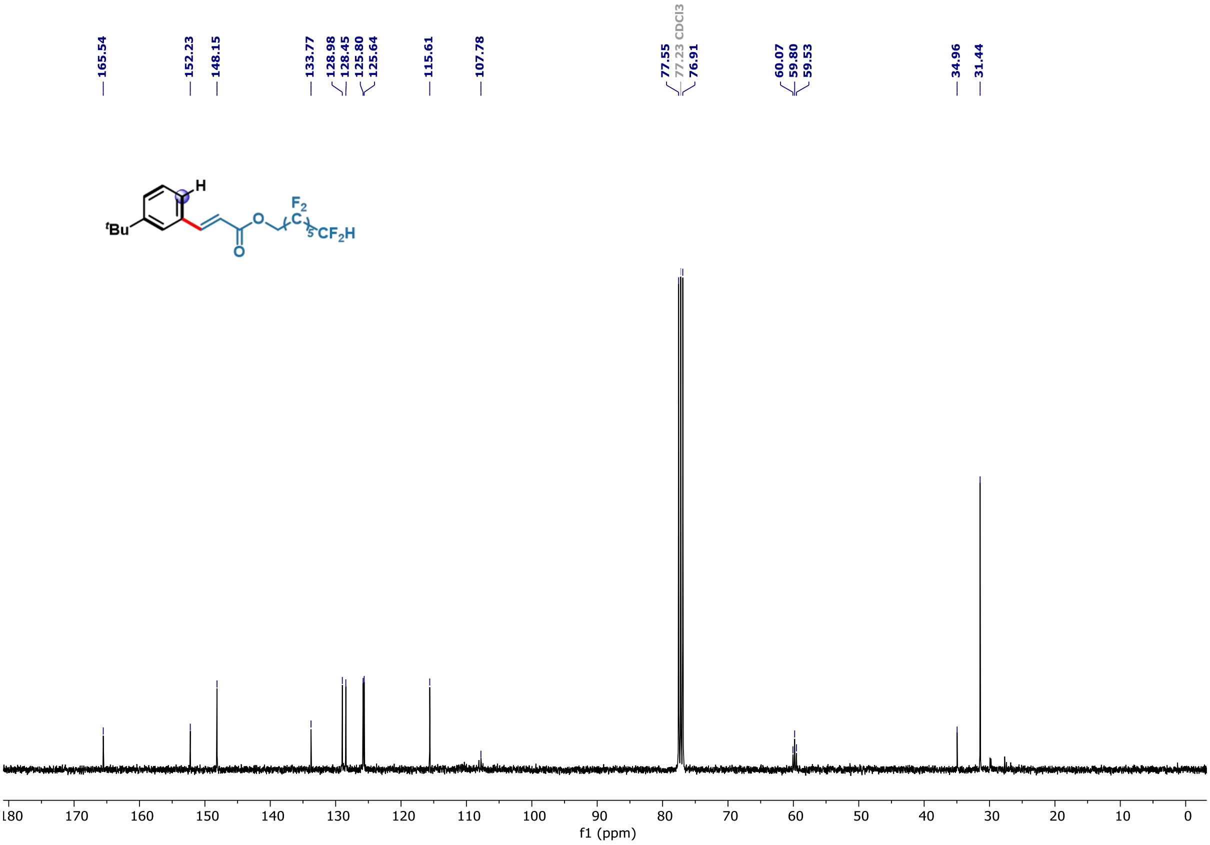

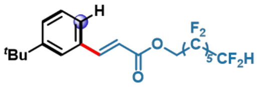

tBu
H
O
O
F2
C
5
CF2H

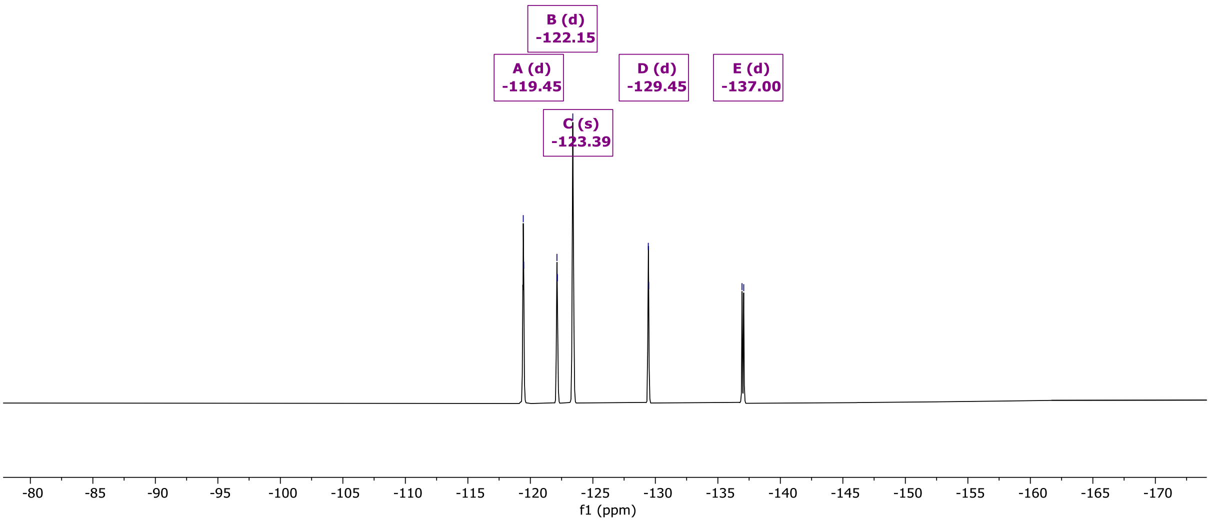

-119.43
-119.47
-122.13
-122.16
-123.39
-129.43
-129.47
-136.93
-137.07
B (d)
-122.15
A (d)
-119.45
D (d)
-129.45
E (d)
-137.00
C (s)
-123.39
-80
-85
-90
-95
-100
-105
-110
-115
-120
-125
-130
-135
-140
-145
-150
-155
-160
-165
-170
f1 (ppm)

Methyl-5-phenylcyclopent-1-ene-1-carboxylate (32)

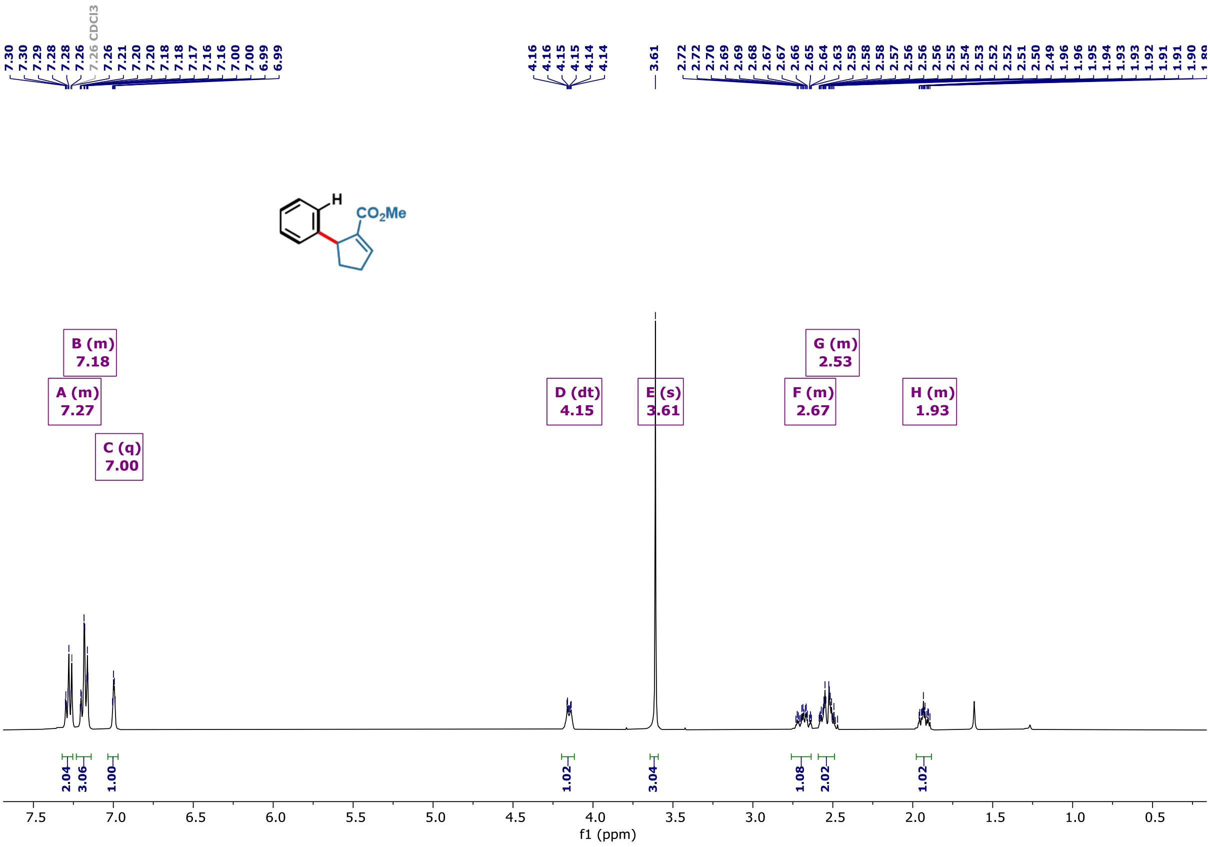

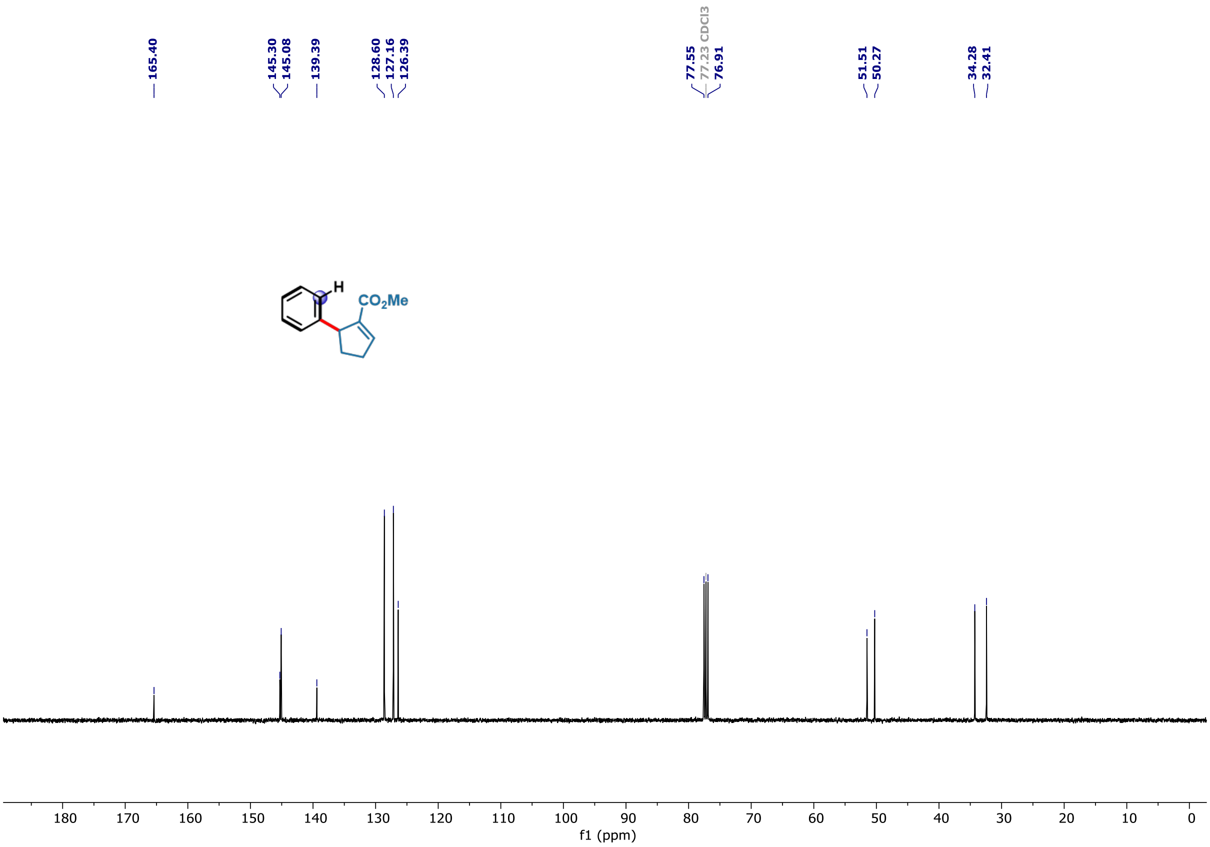

Methyl-1,4,5,6-tetrahydro-[1,1'-biphenyl]-2-carboxylate (33)

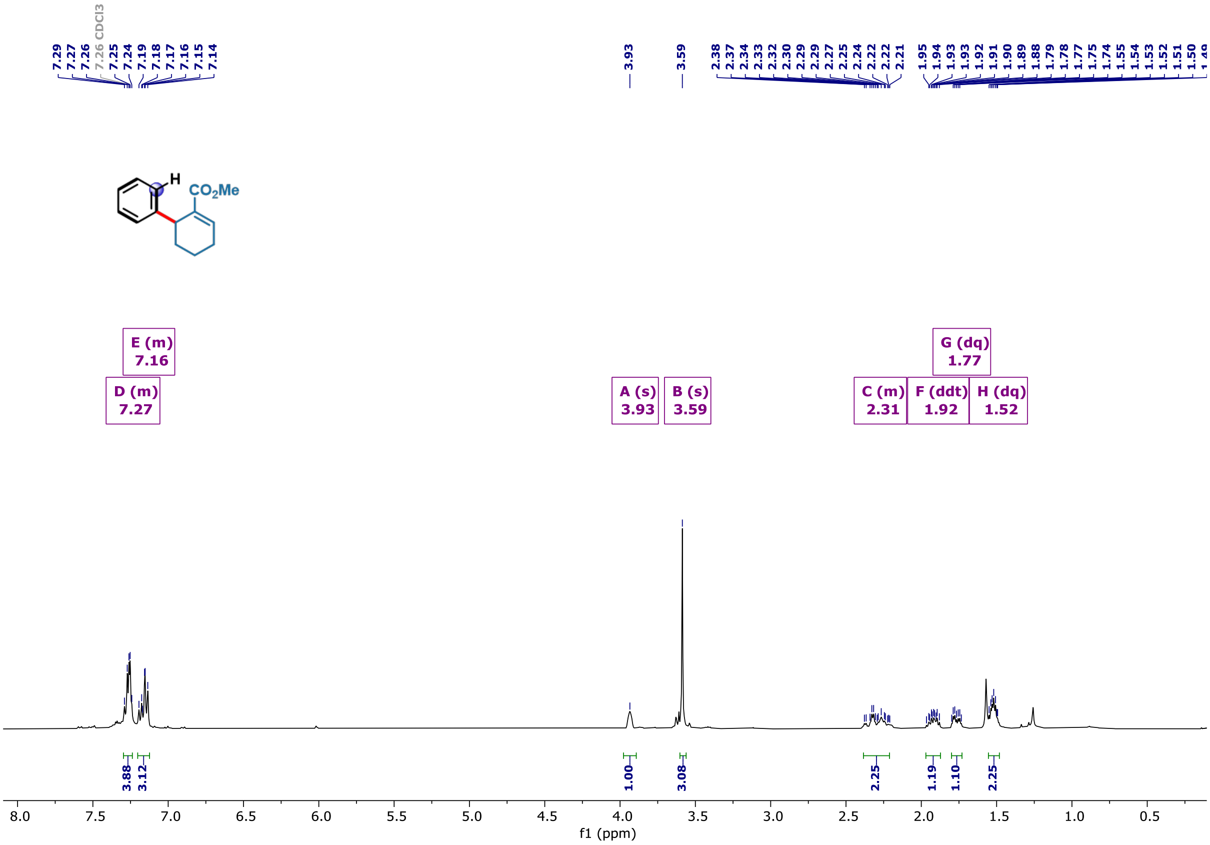

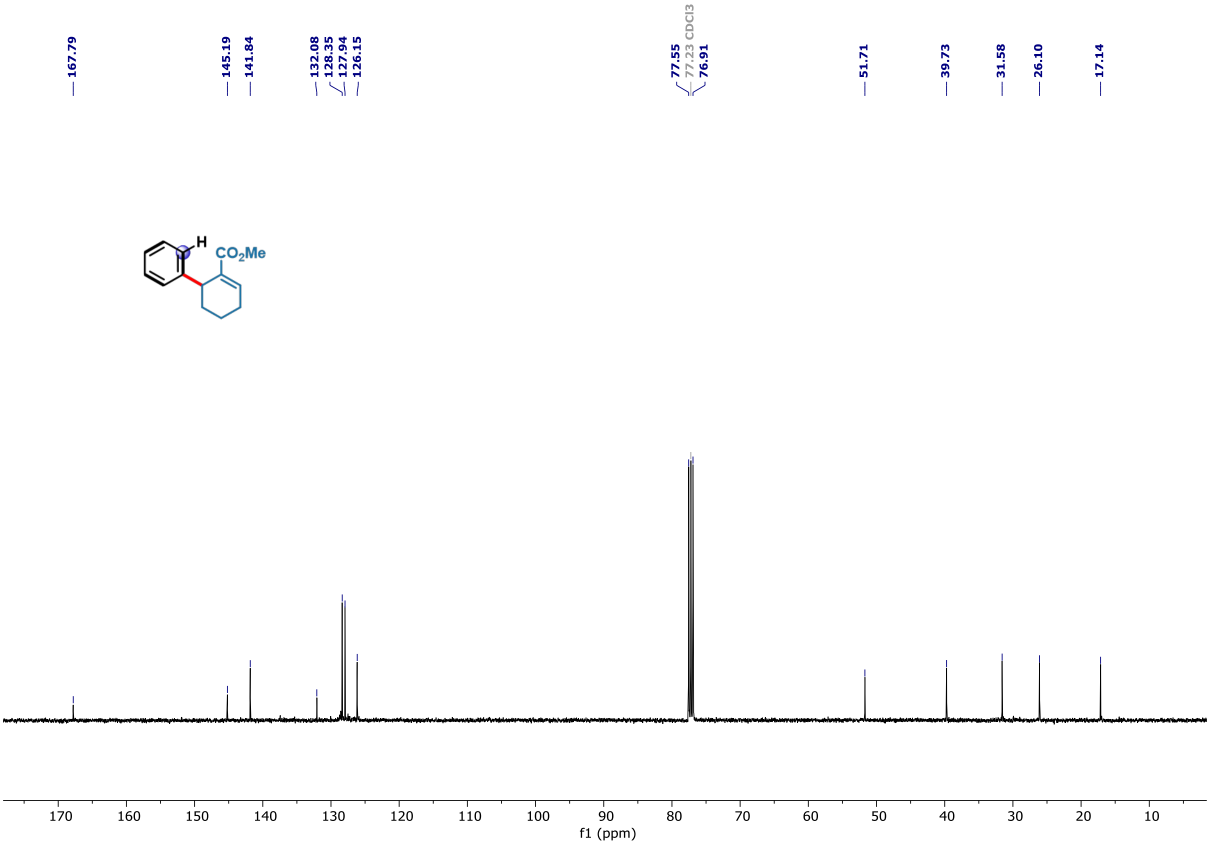

(E)-4-(3-(tert-butyl)phenyl)but-3-en-2-one (34)

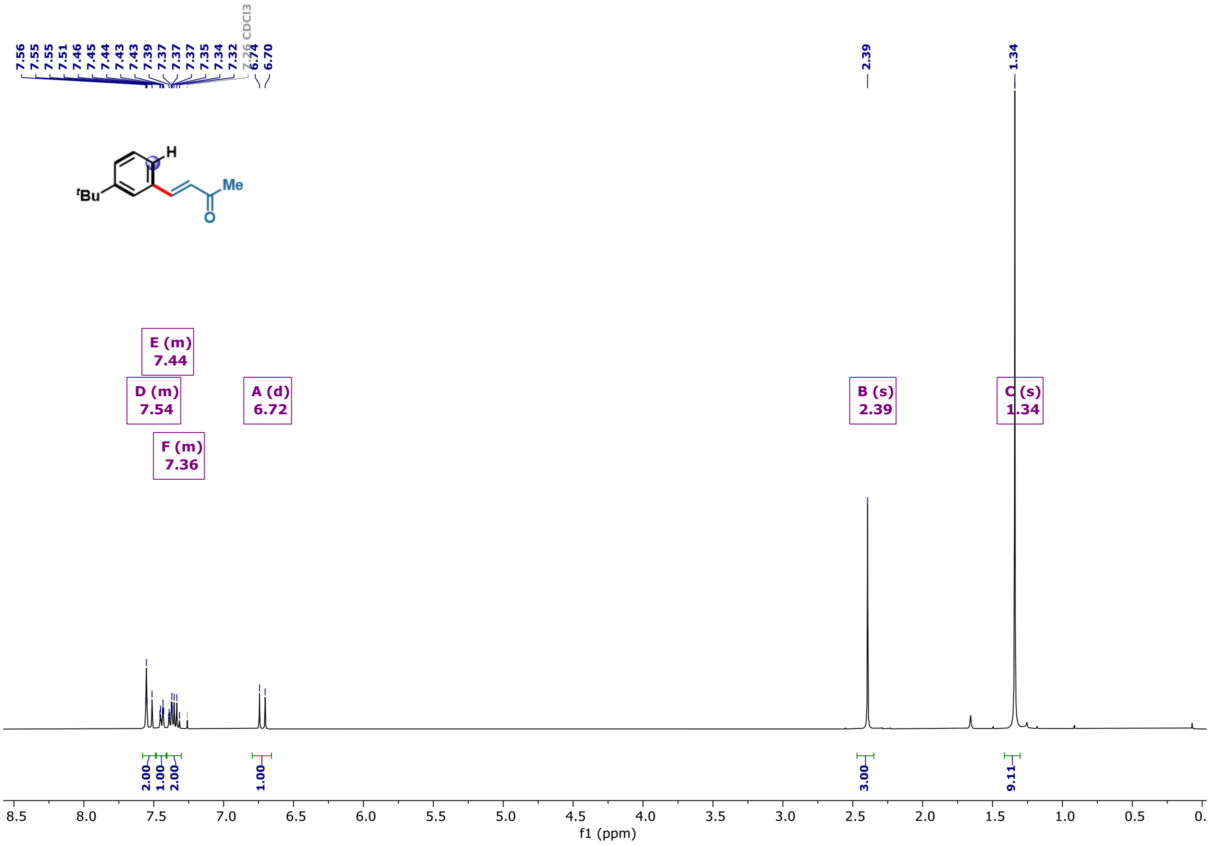

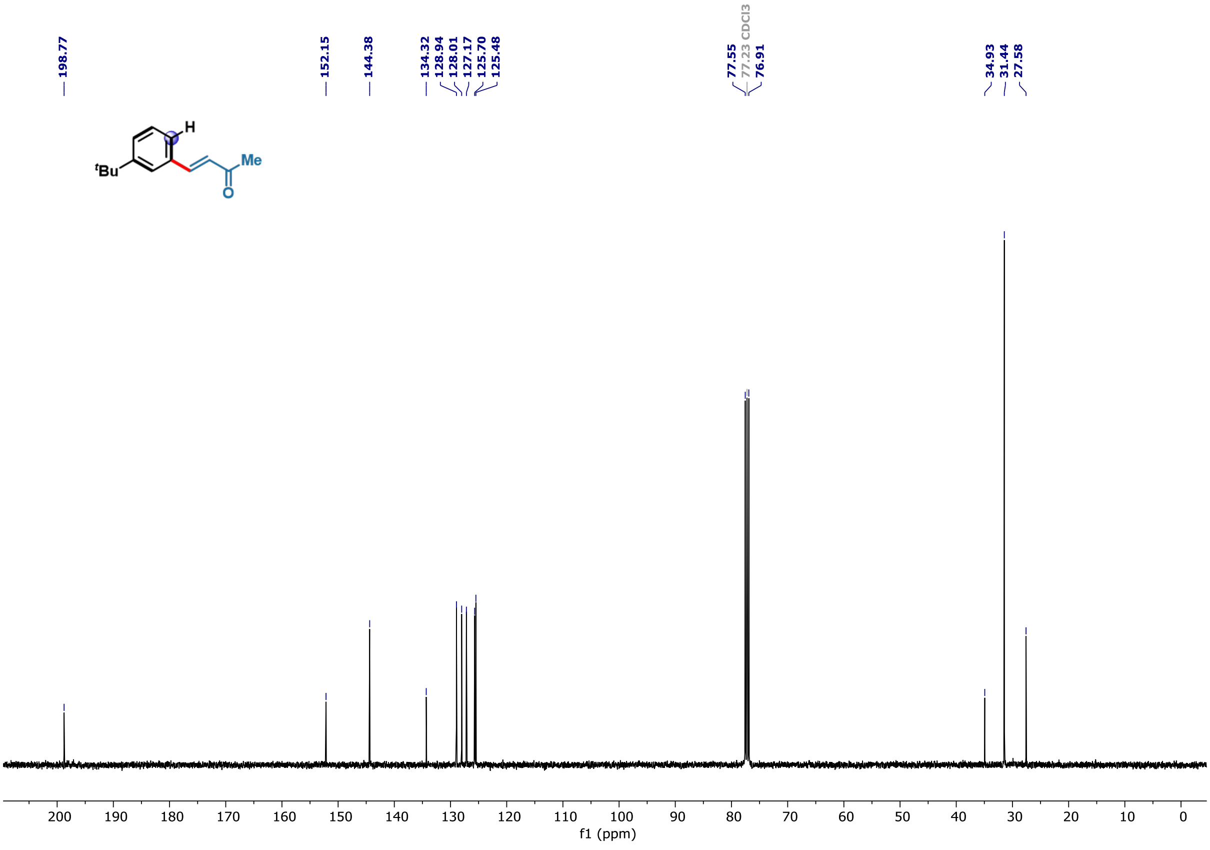

(E)-3-(3-(tert-butyl)phenyl)acrylonitrile (35)

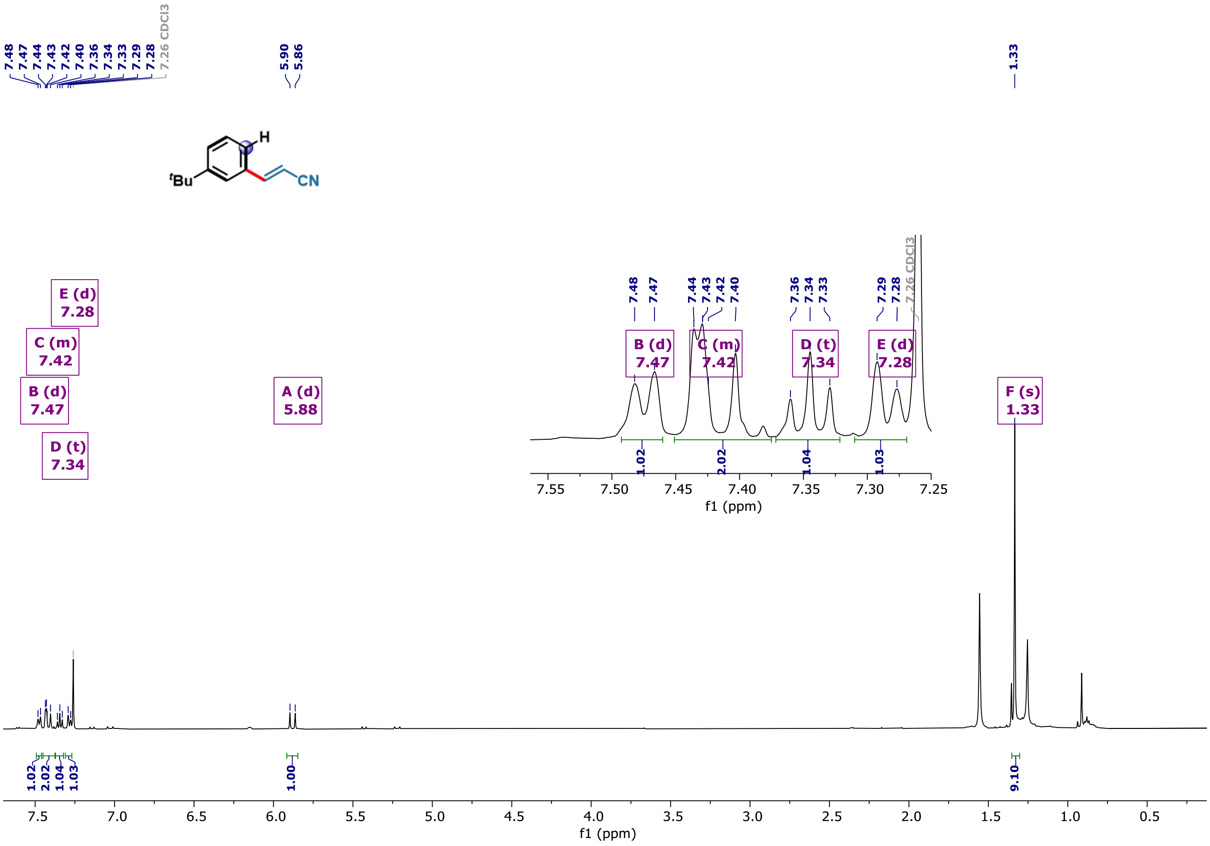

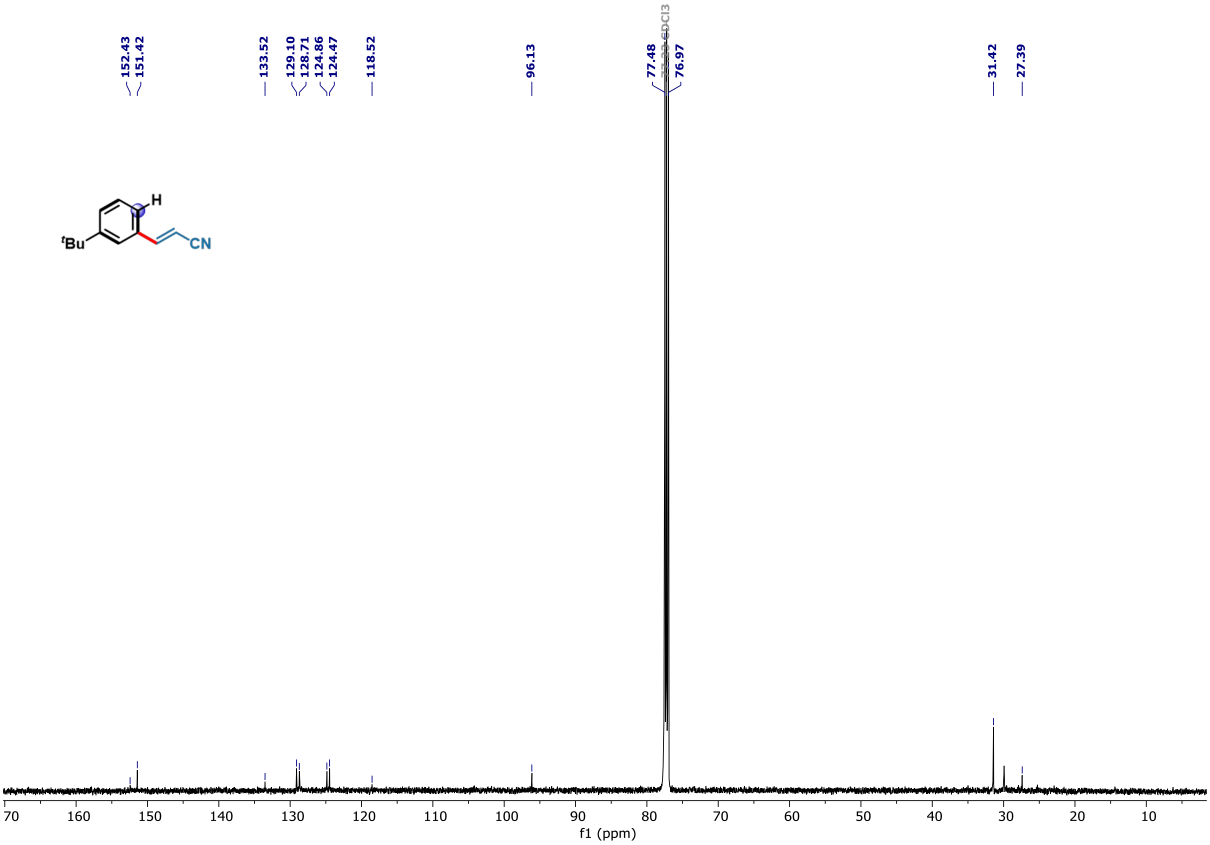

Cinnamonitrile (36)

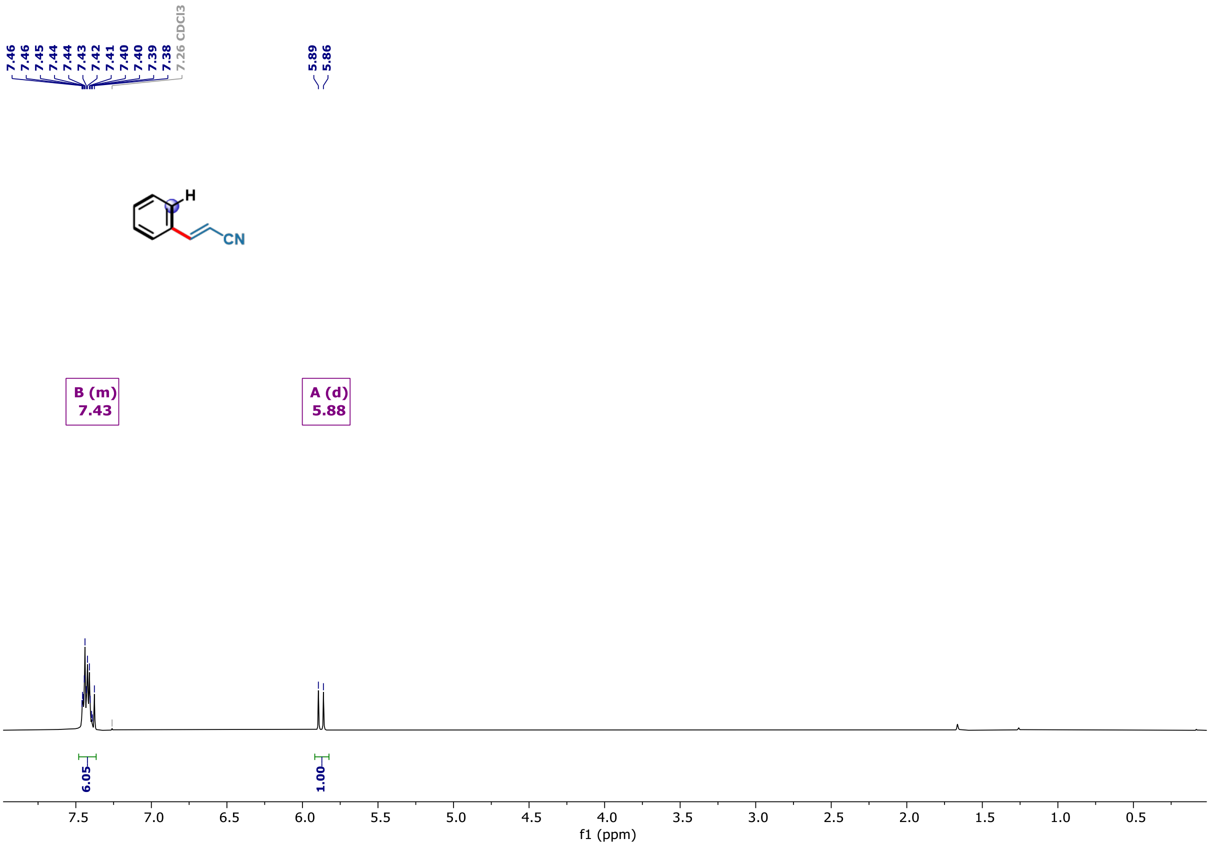

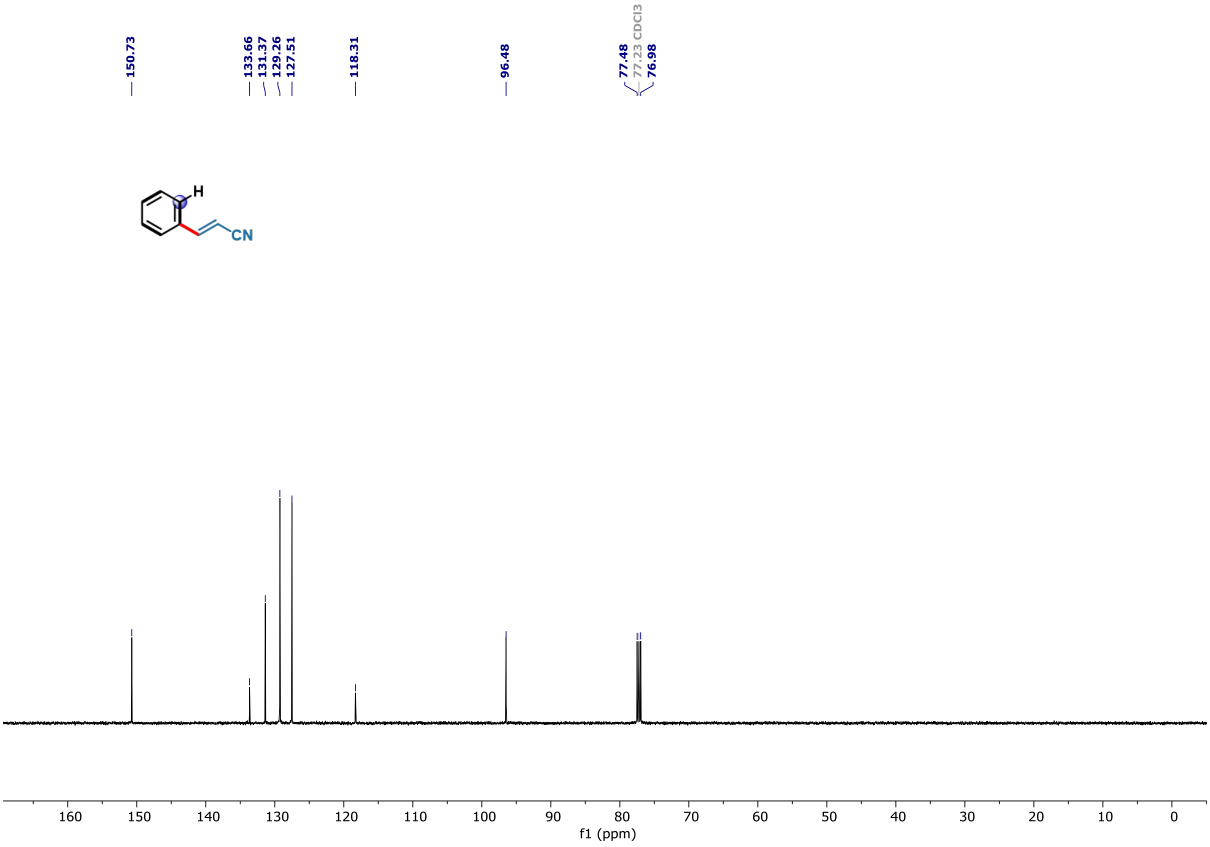

N,N-dimethylcinnamamide (37)

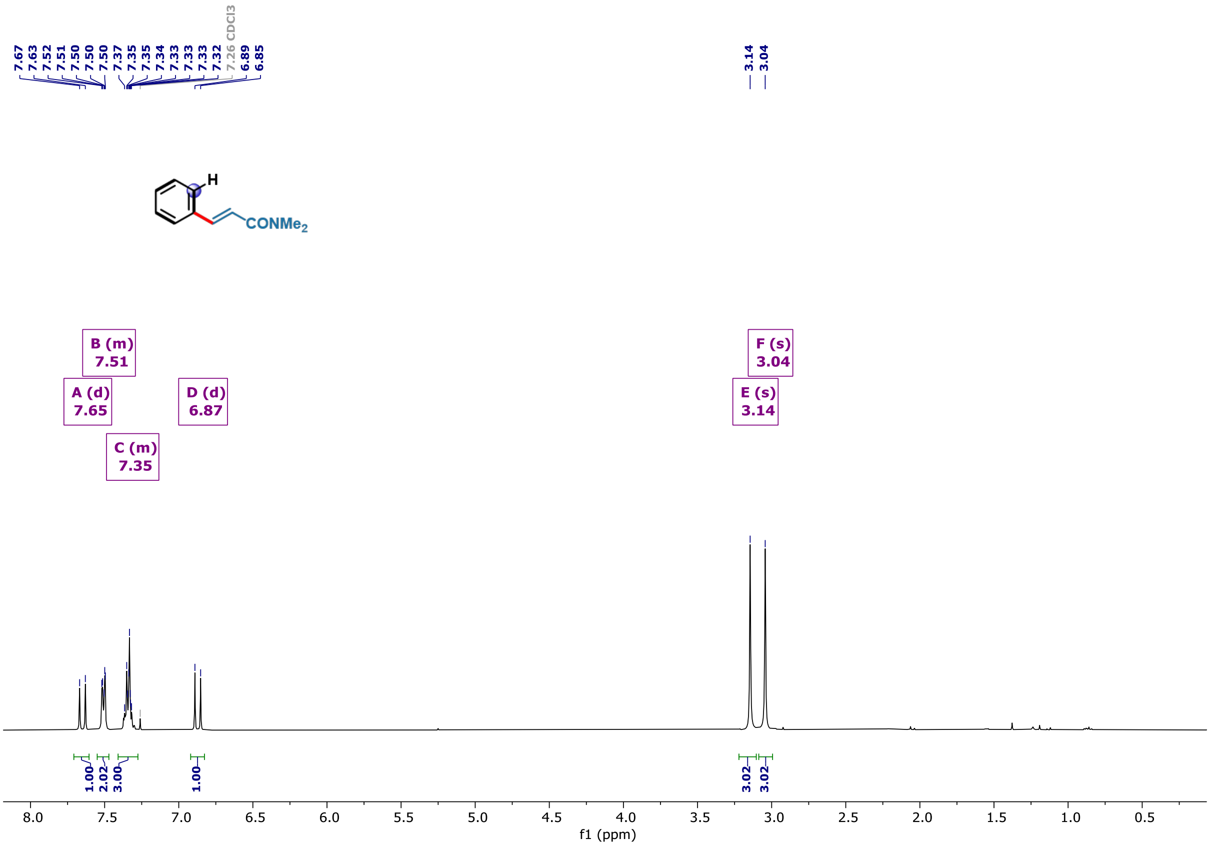

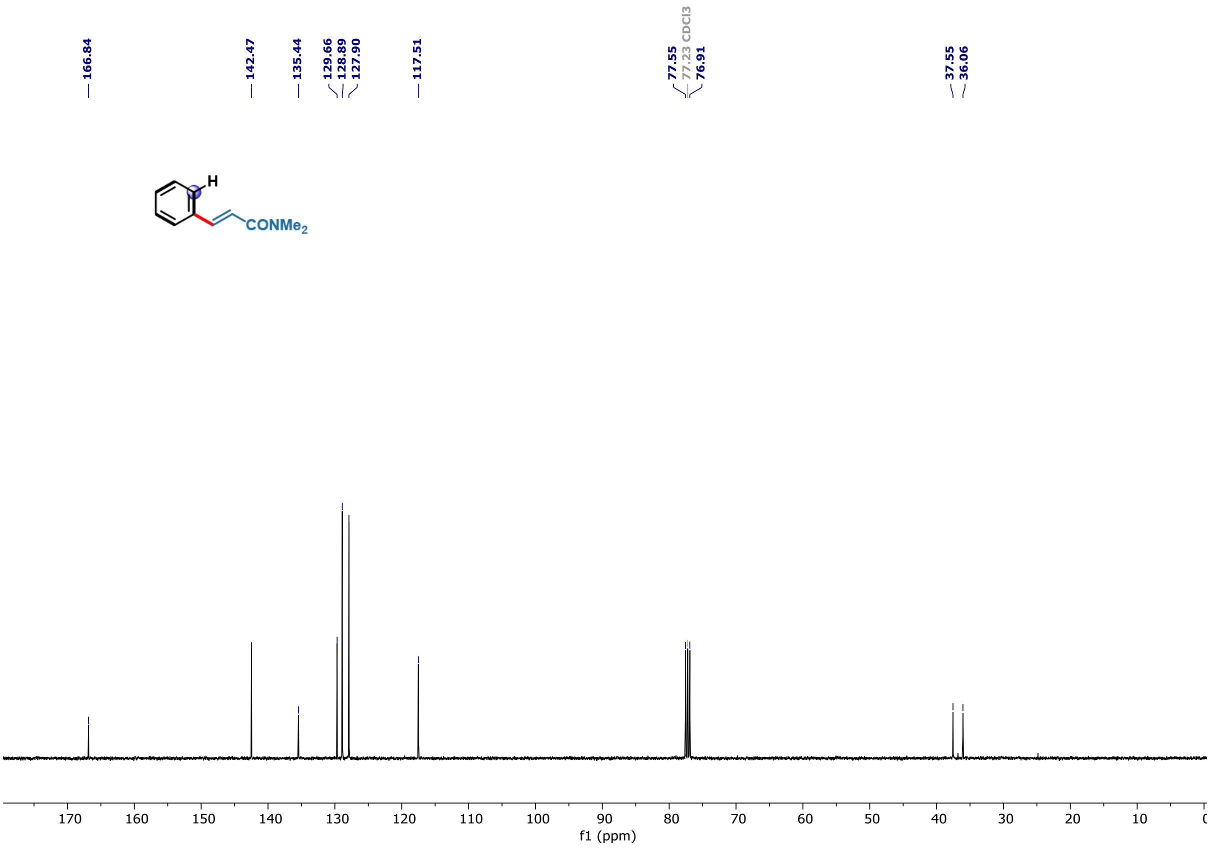

(E)-3-(3-(tert-butyl)phenyl)-N,N-dimethylacrylamide (38)

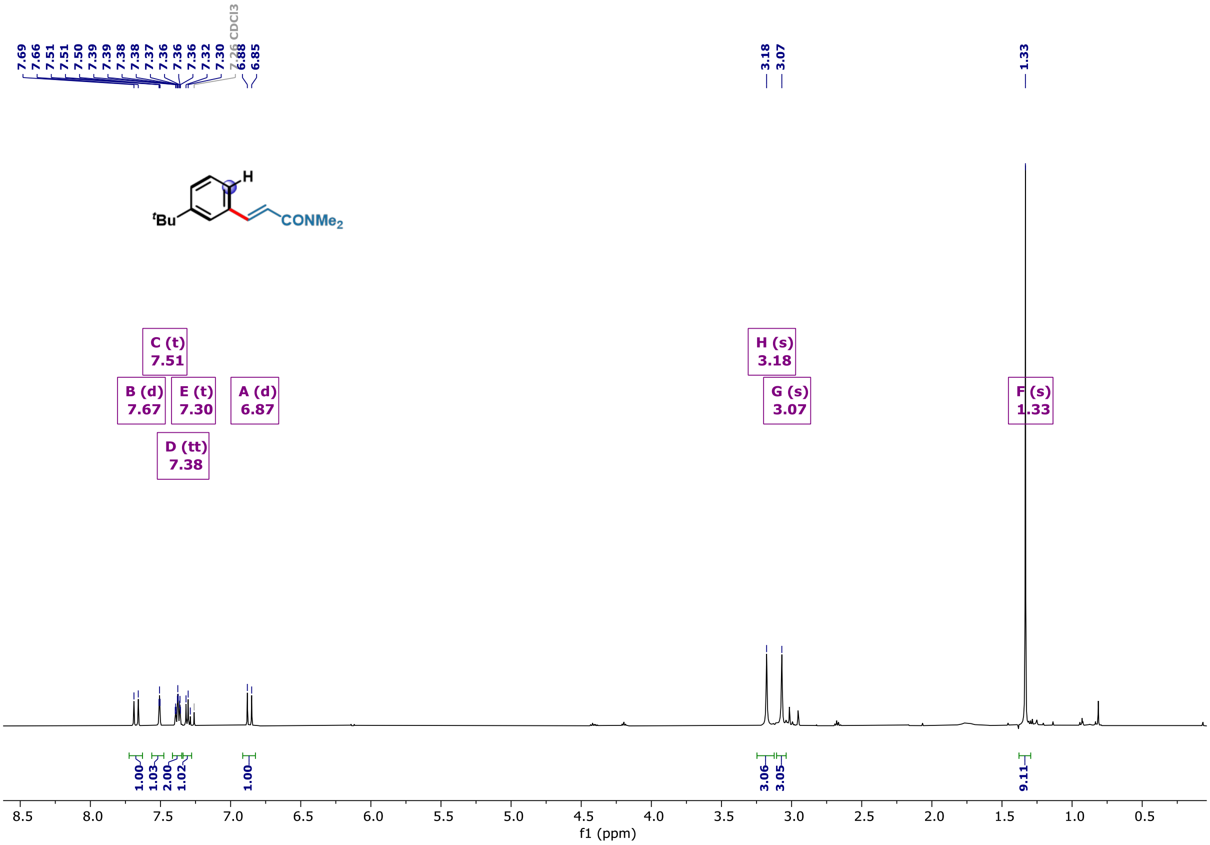

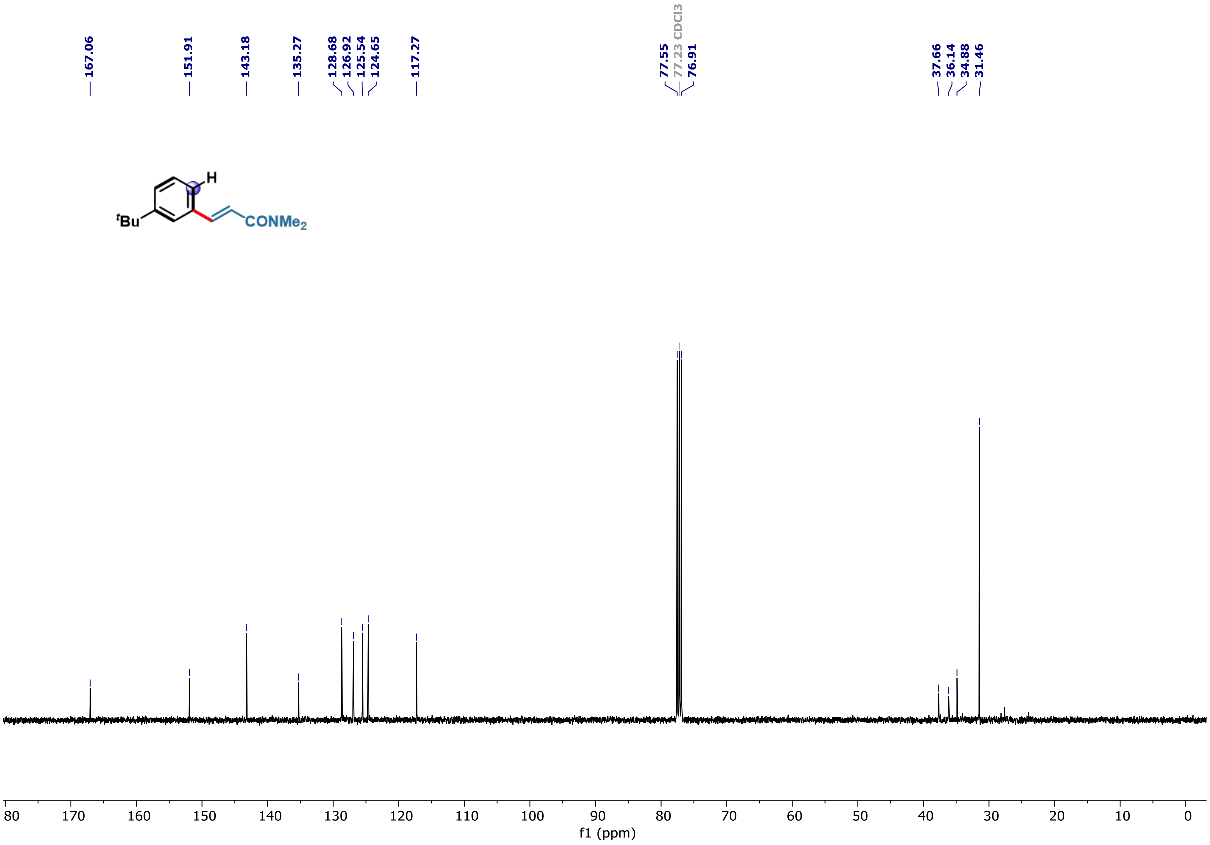

(E)-N,N-dimethyl-3-(naphthalen-1-yl)acrylamide (39)

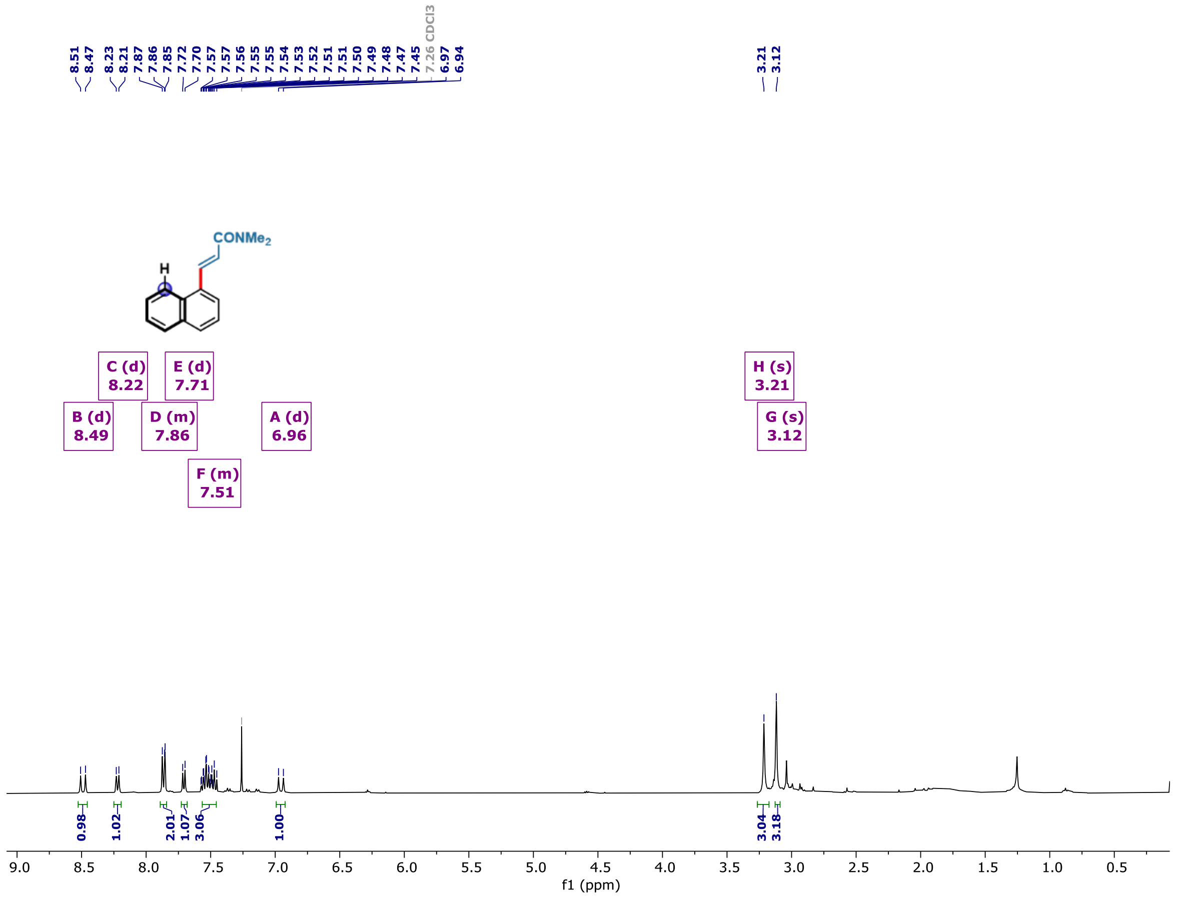

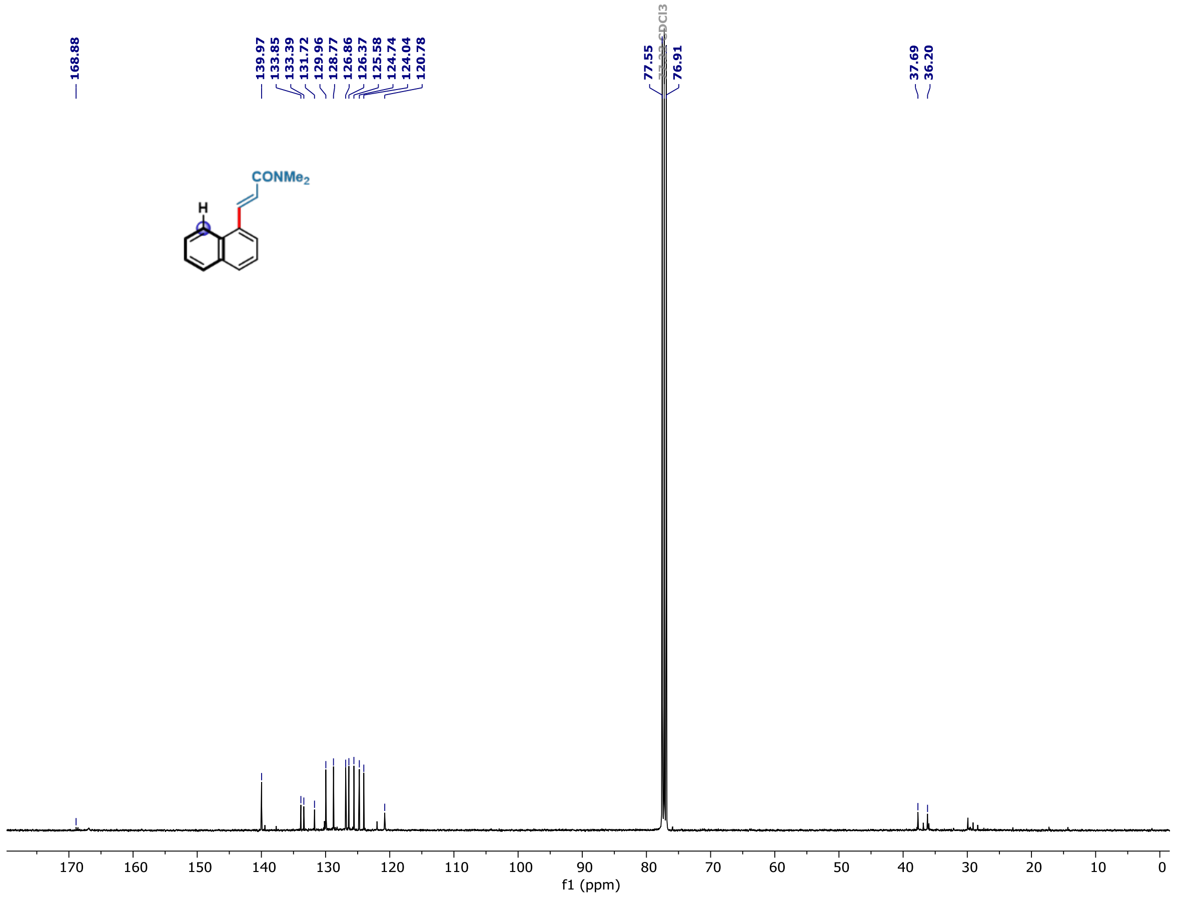

(E)-3-phenyl-1-(pyrrolidin-1-yl)prop-2-en-1-one (40)

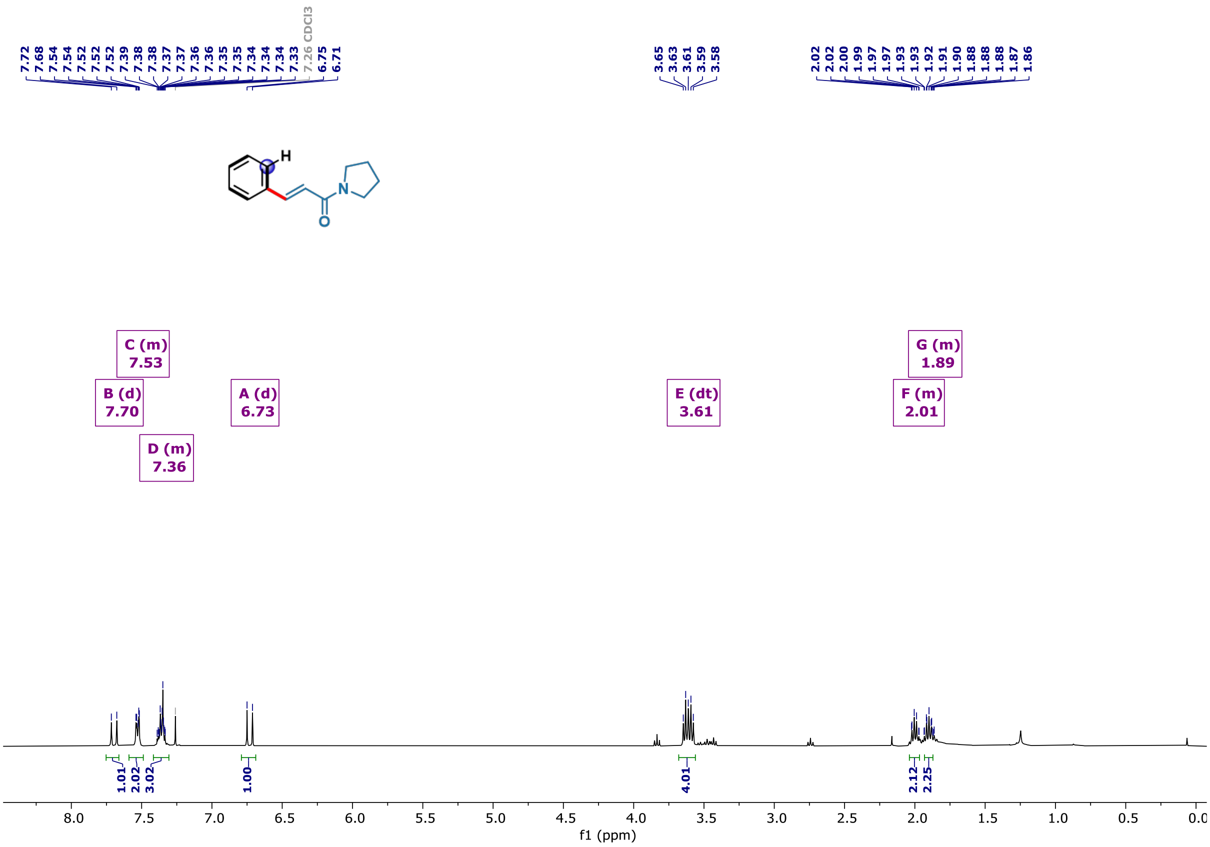

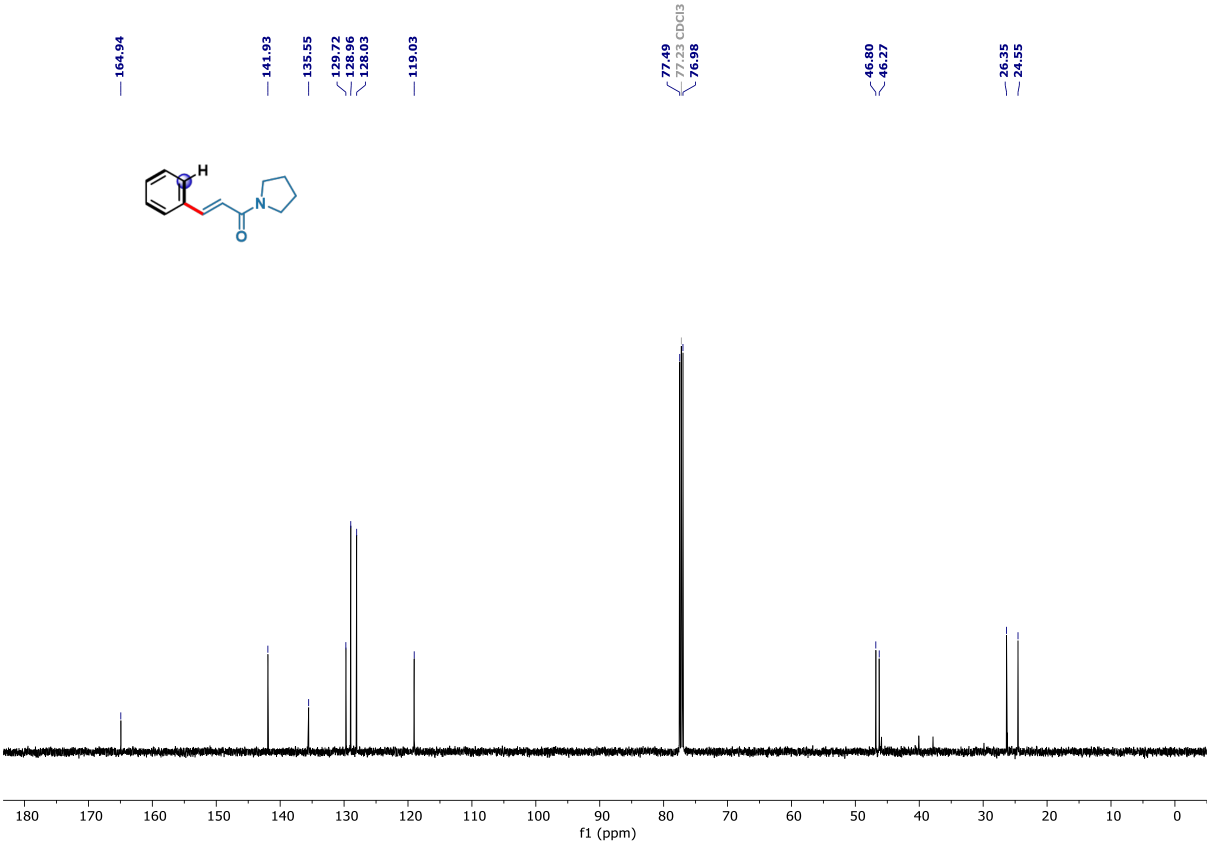

(E)-3-phenyl-1-(piperidin-1-yl)prop-2-en-1-one (41)

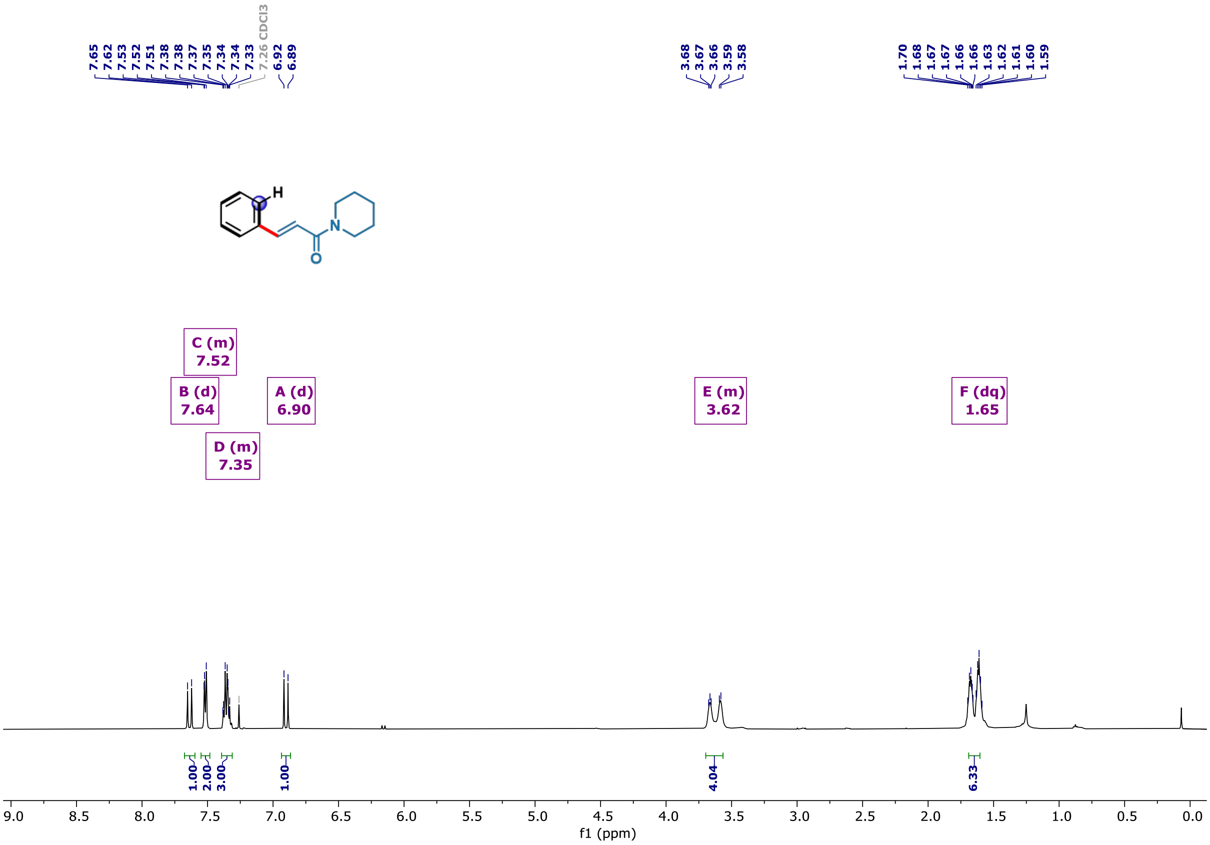

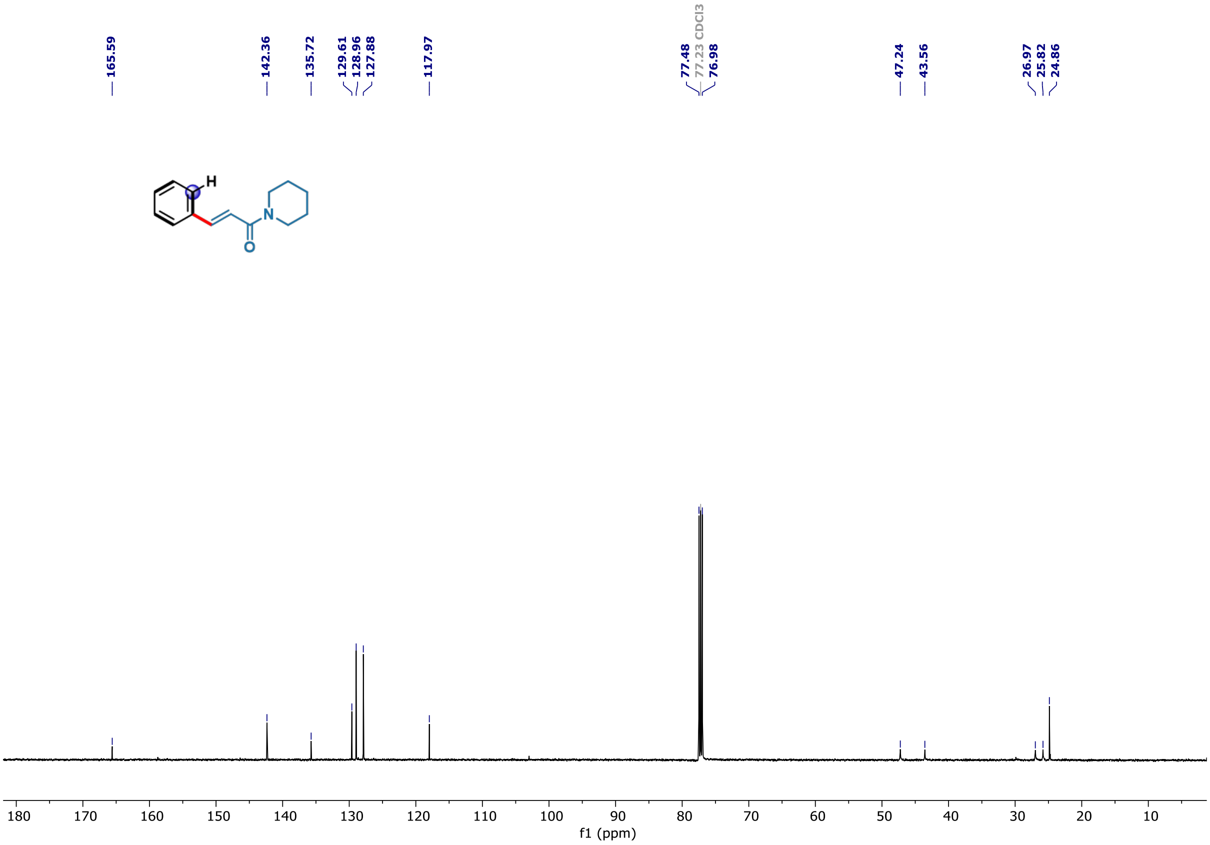

Diethyl (E)-(3-methylstyryl)phosphonate (42)

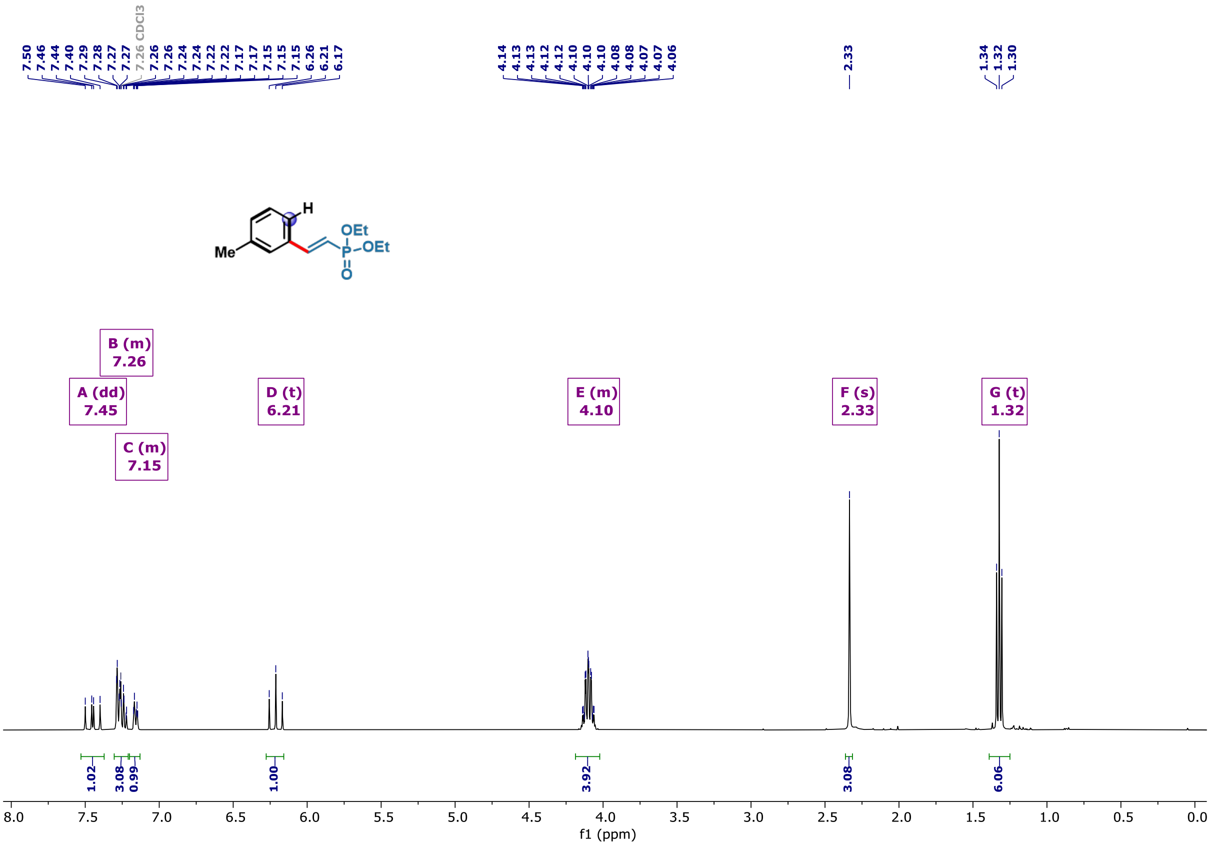

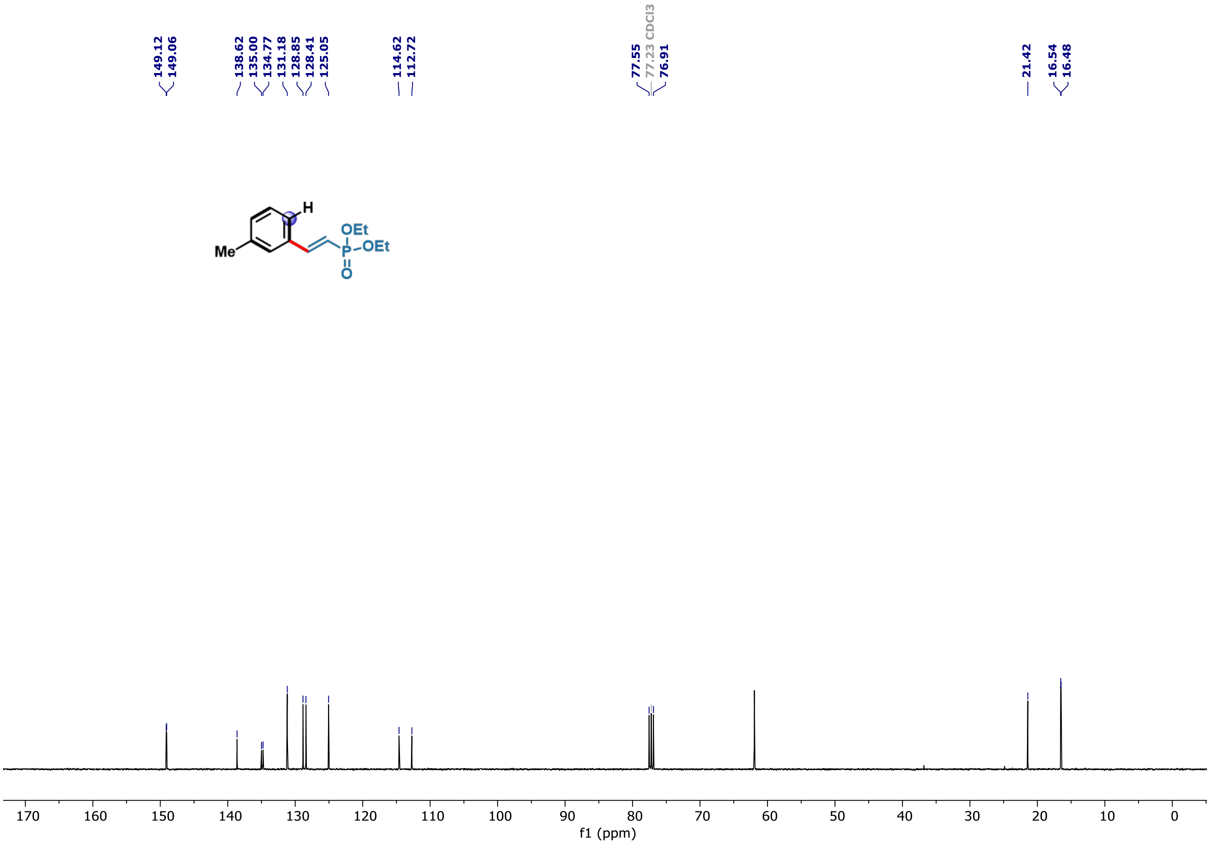

Diethyl (E)-styrylphosphonate (43)

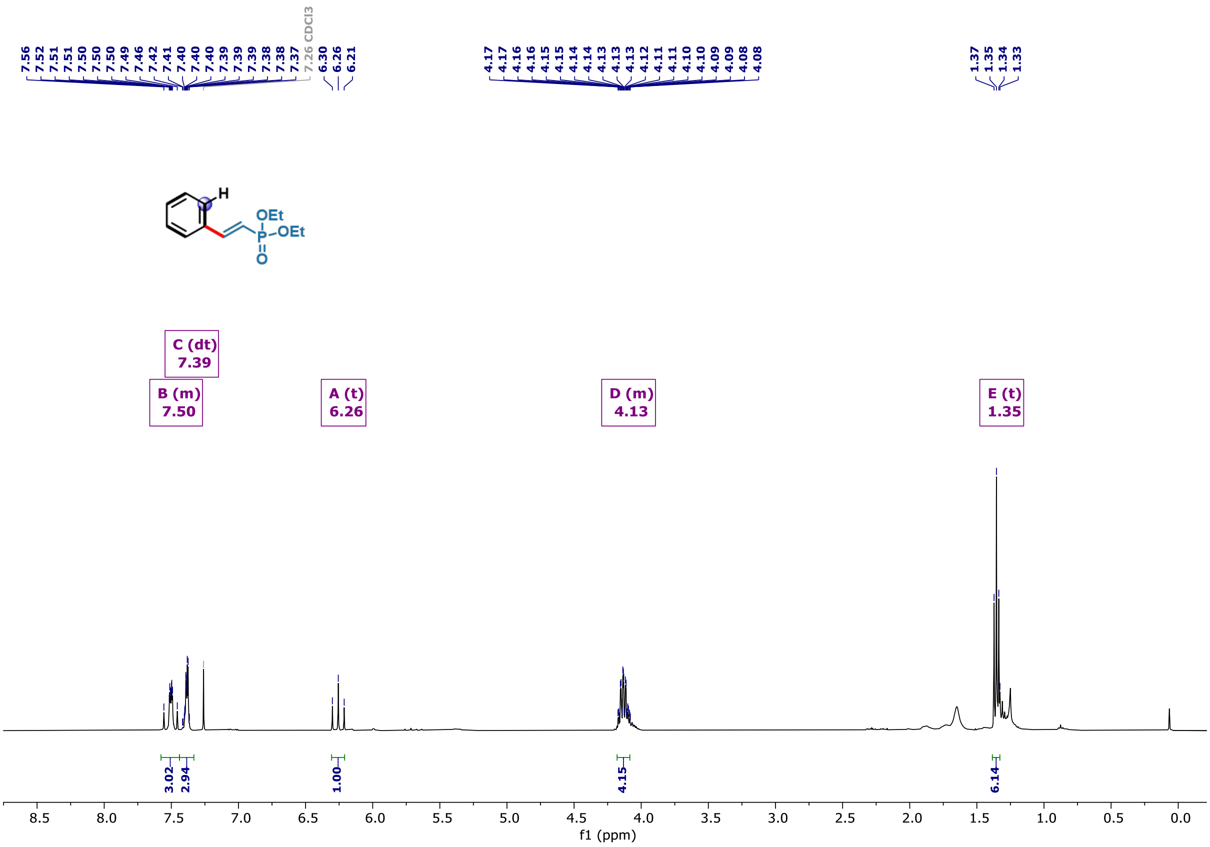

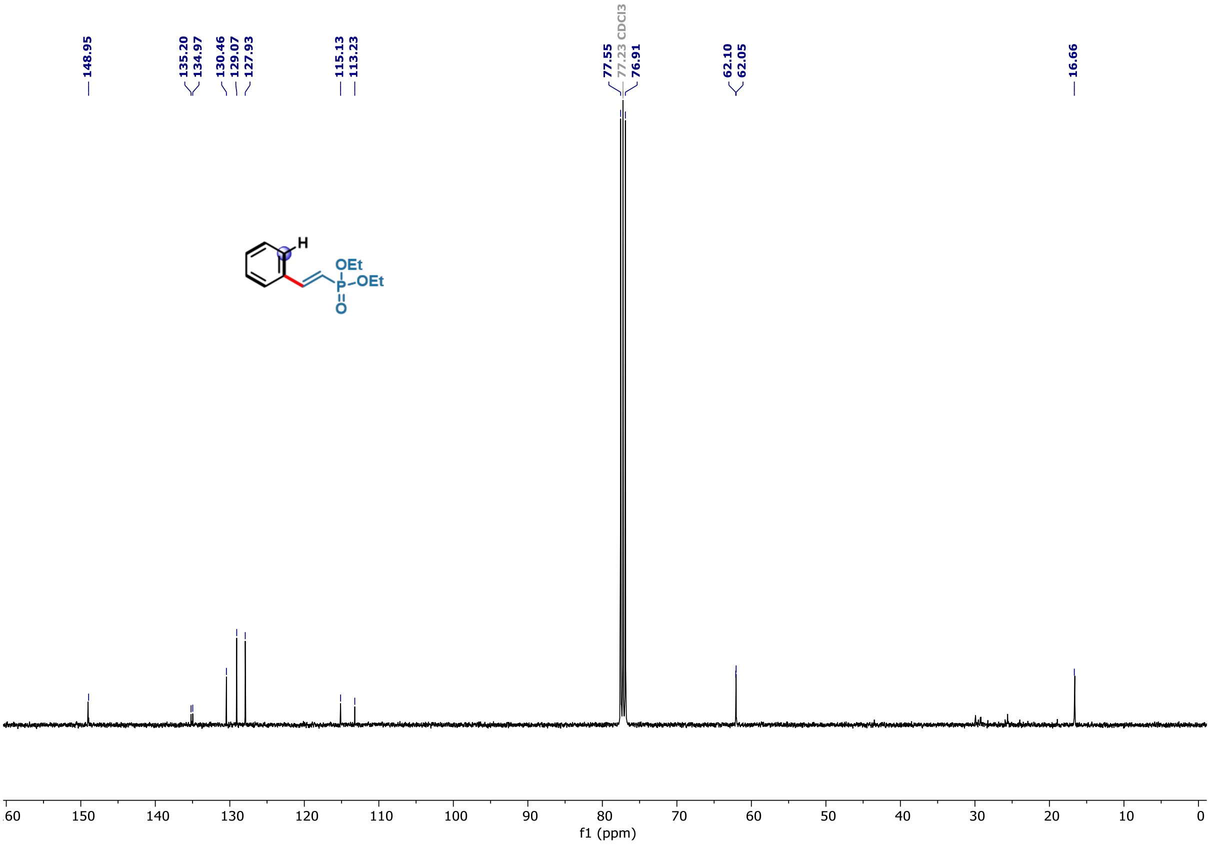

Diethyl (E)-(3-(tert-butyl)styryl)phosphonate (44)

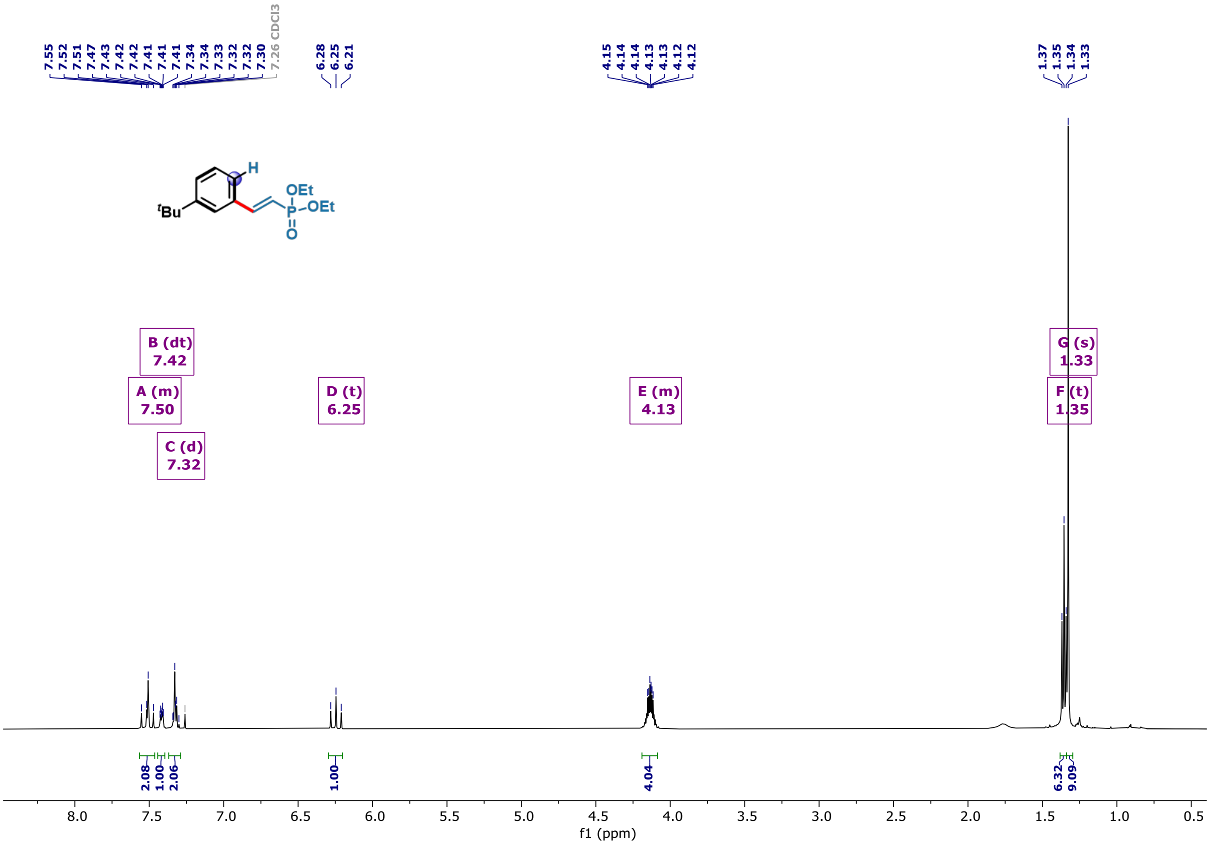

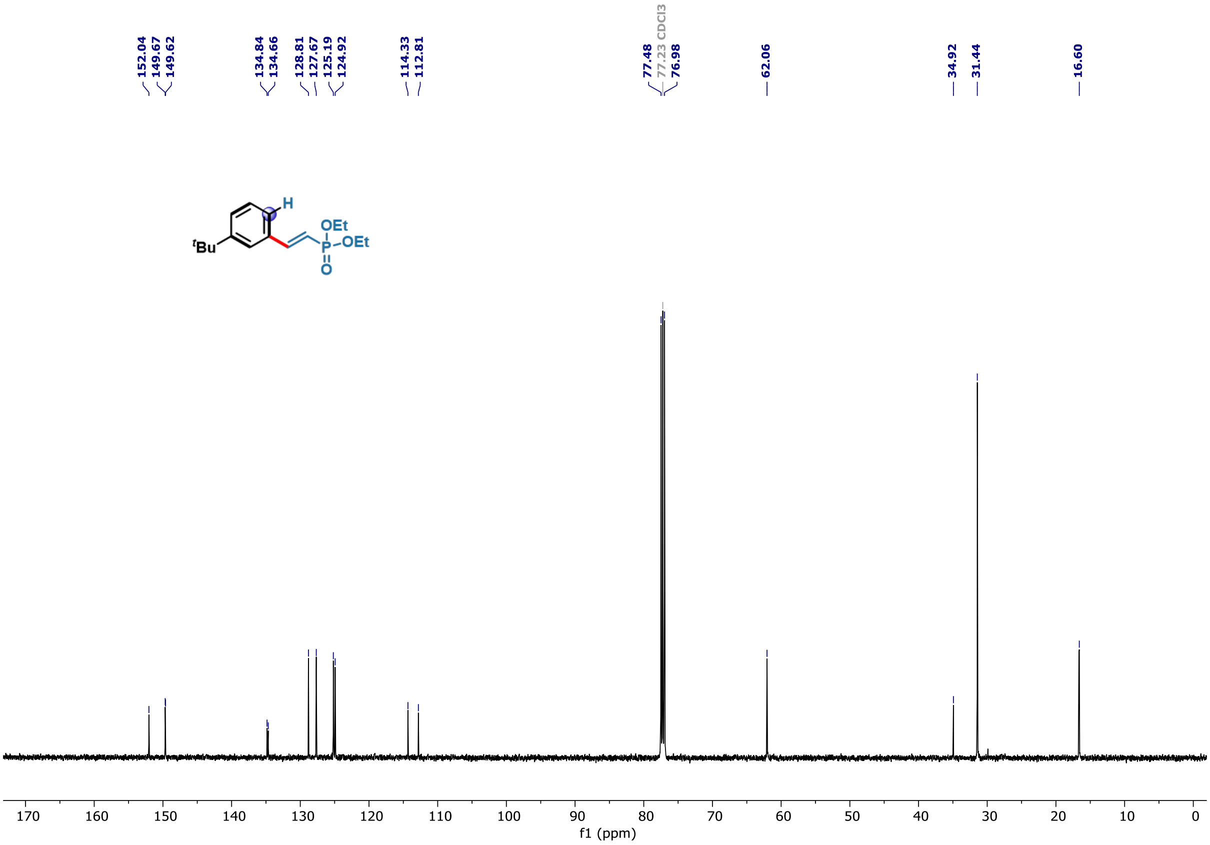

(E)-(2-(phenylsulfonyl)vinyl)benzene (45)

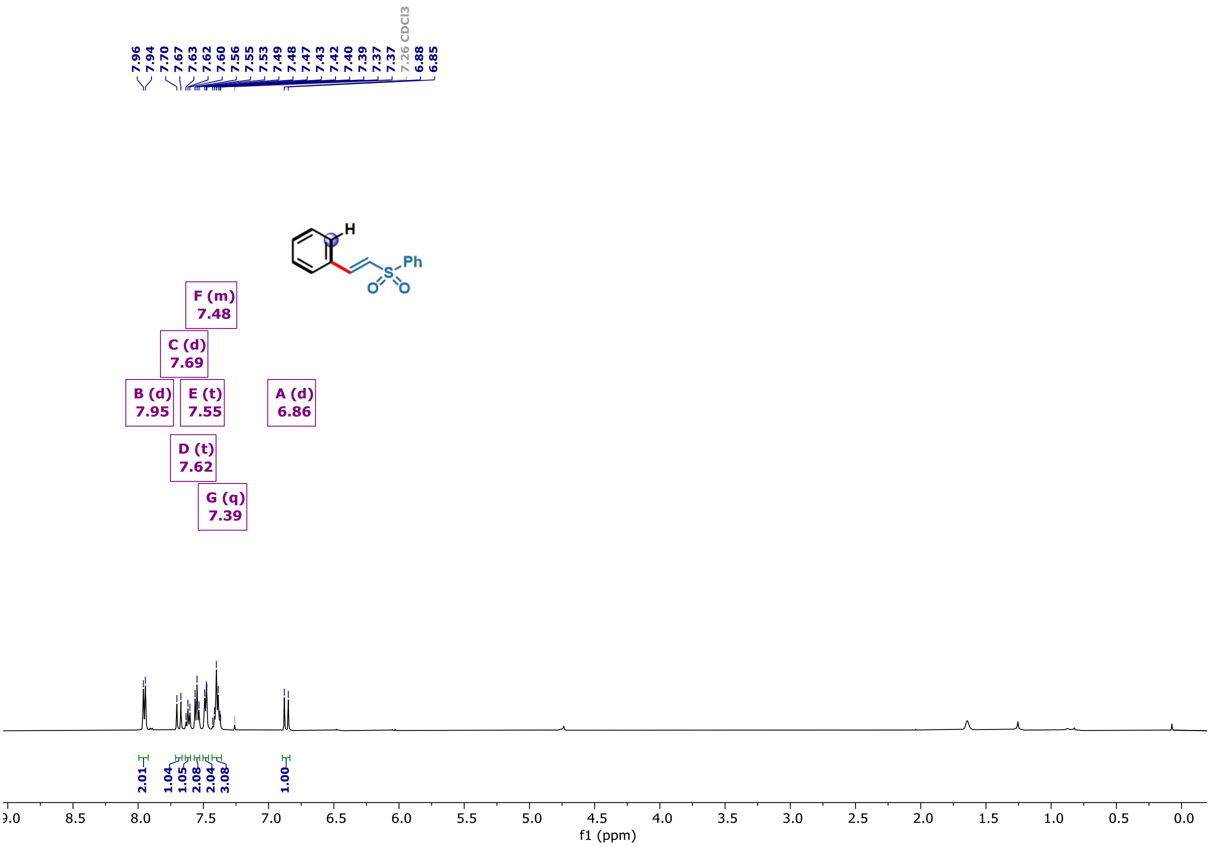

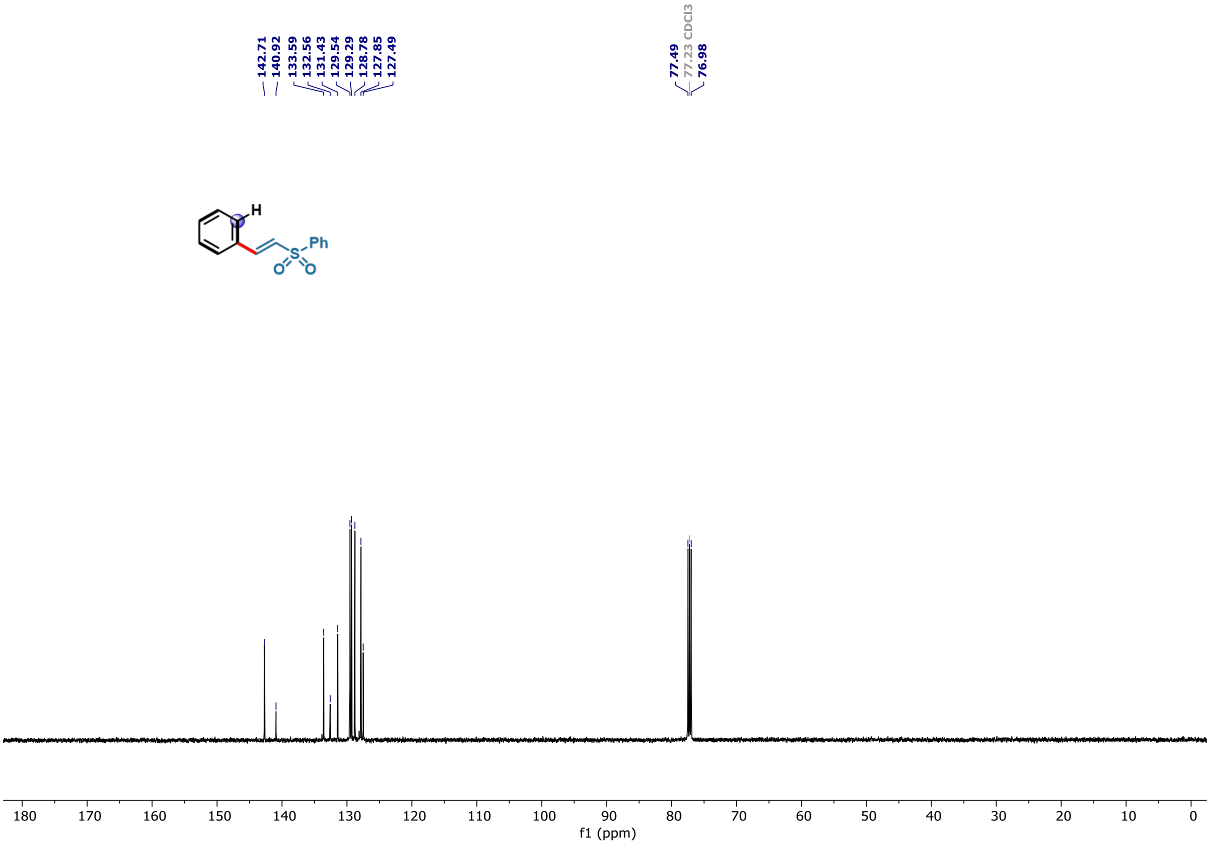

Dimethyl 2-(3-(tert-butyl)phenyl)maleate (46)

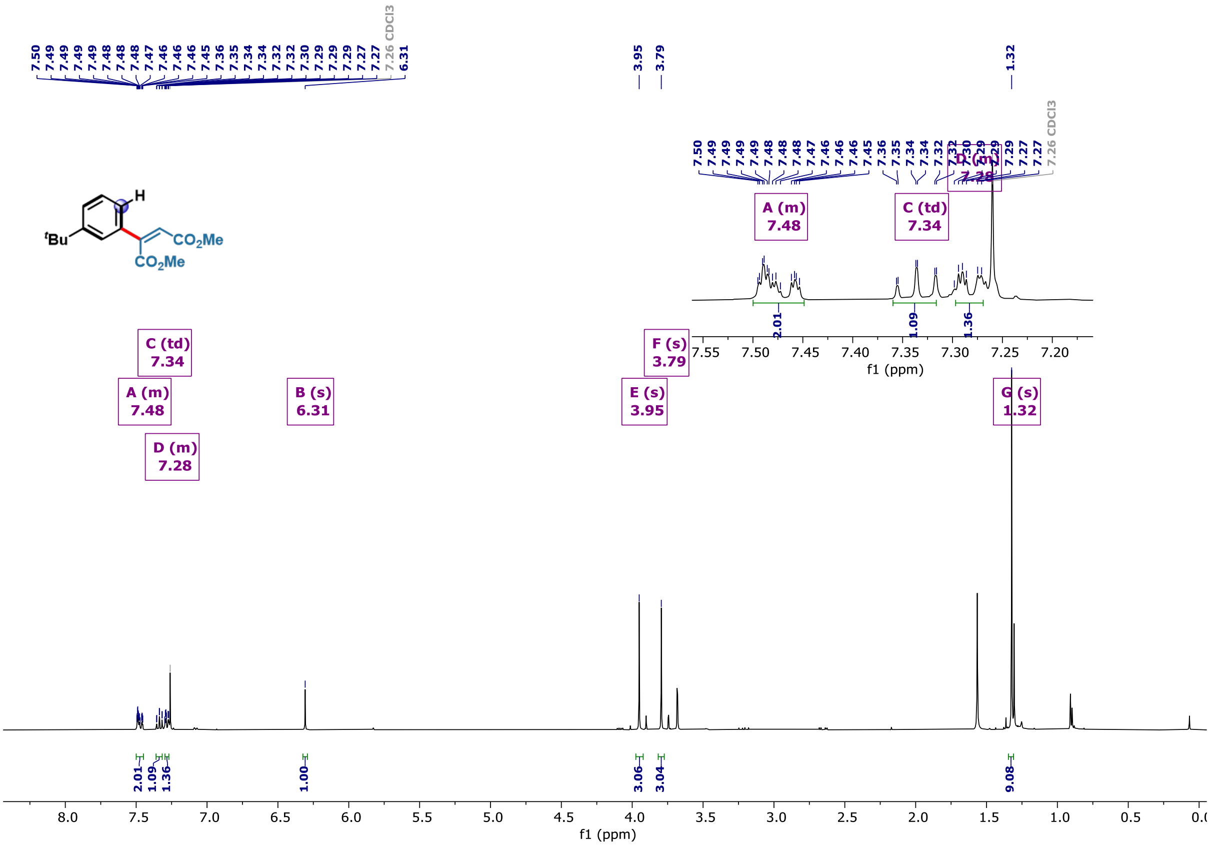

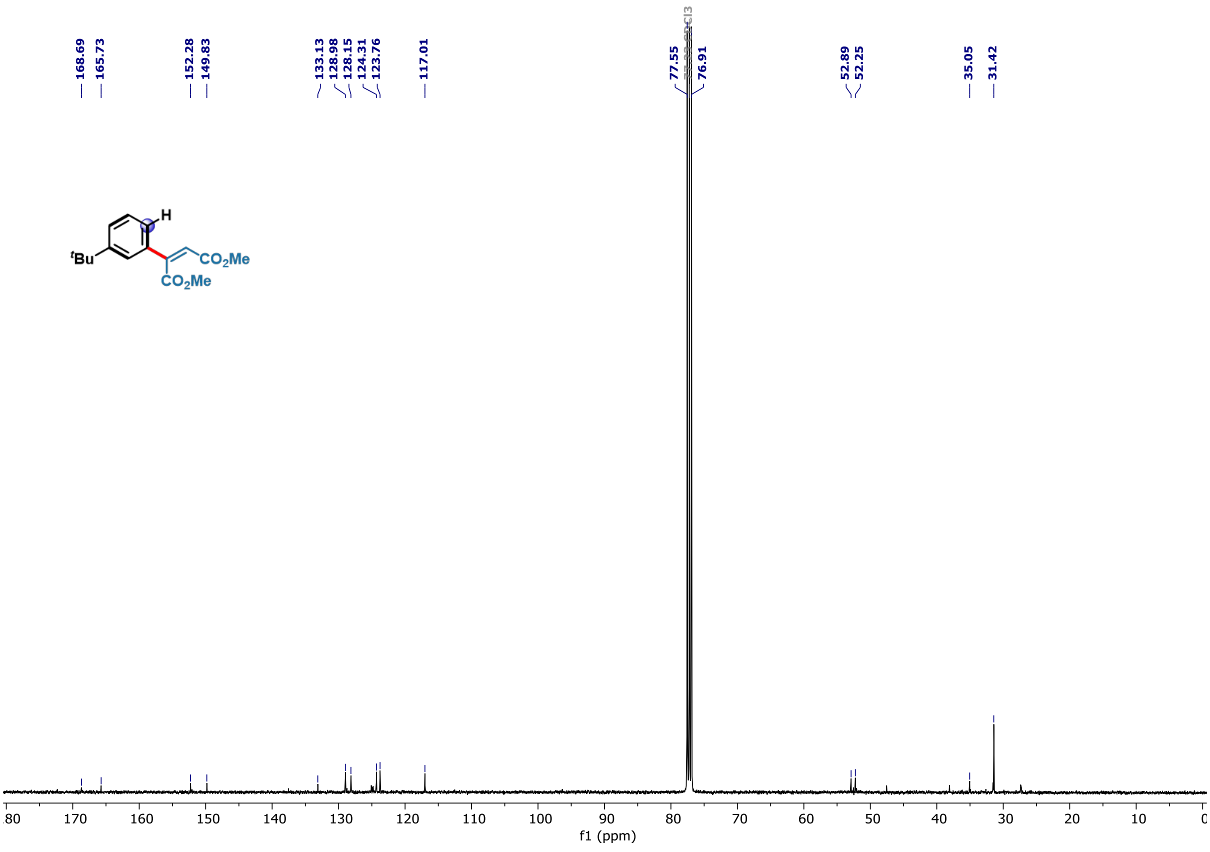

(E)-1-(3-(tert-butyl)styryl)-2,3,4,5,6-pentafluorobenzene (47)

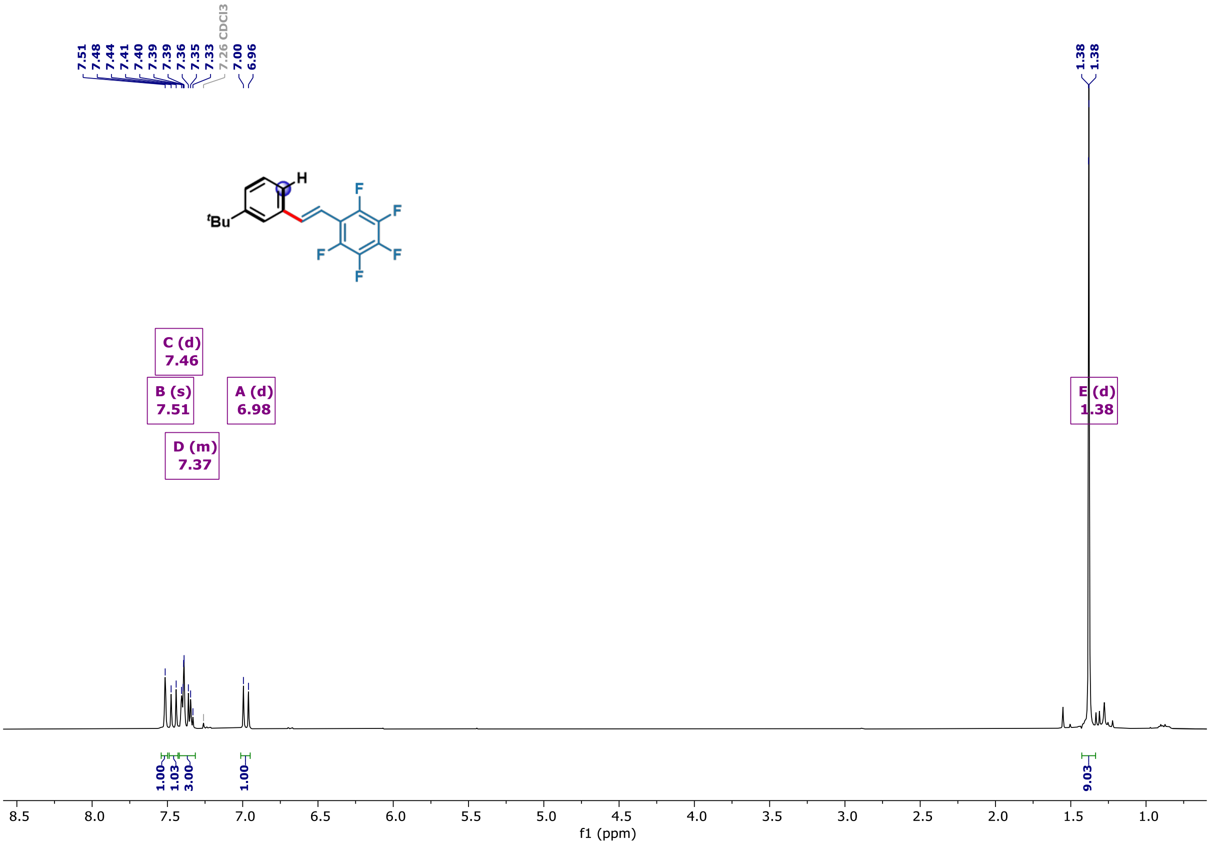

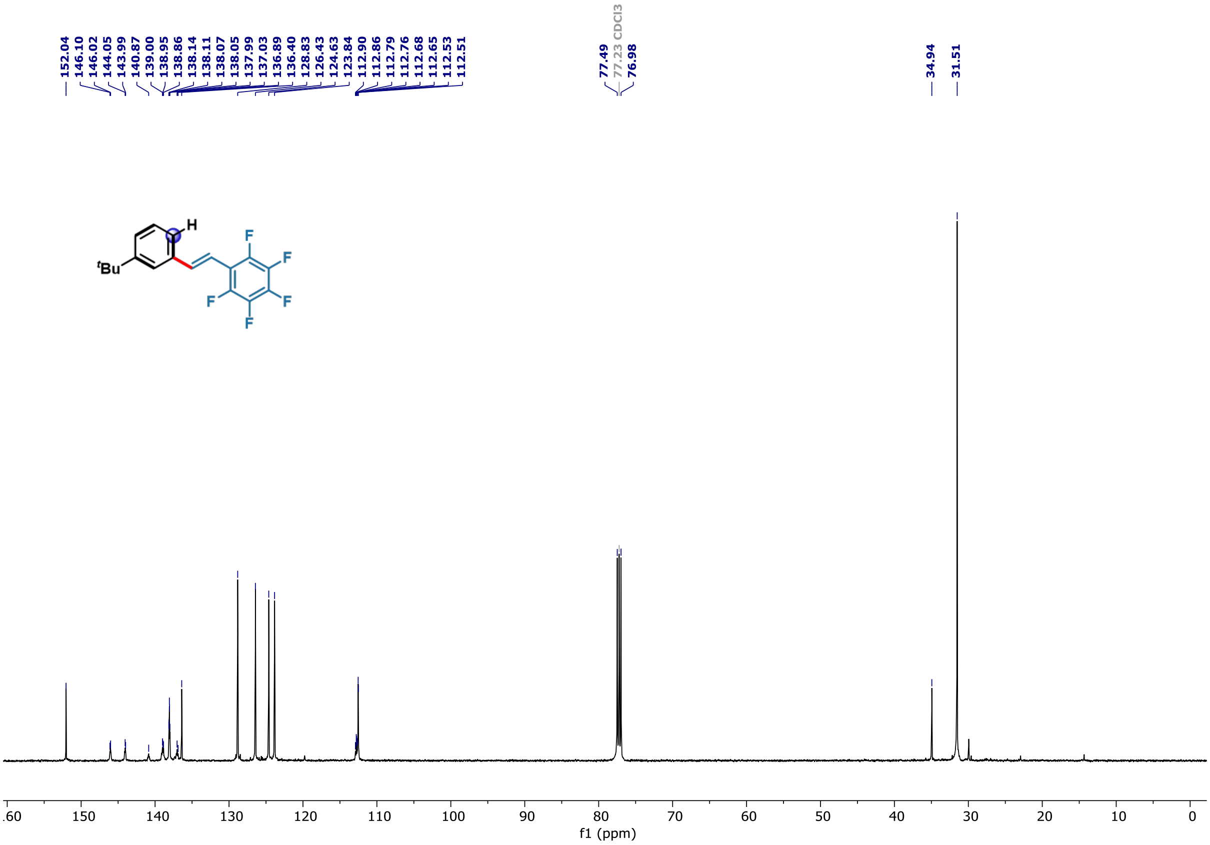

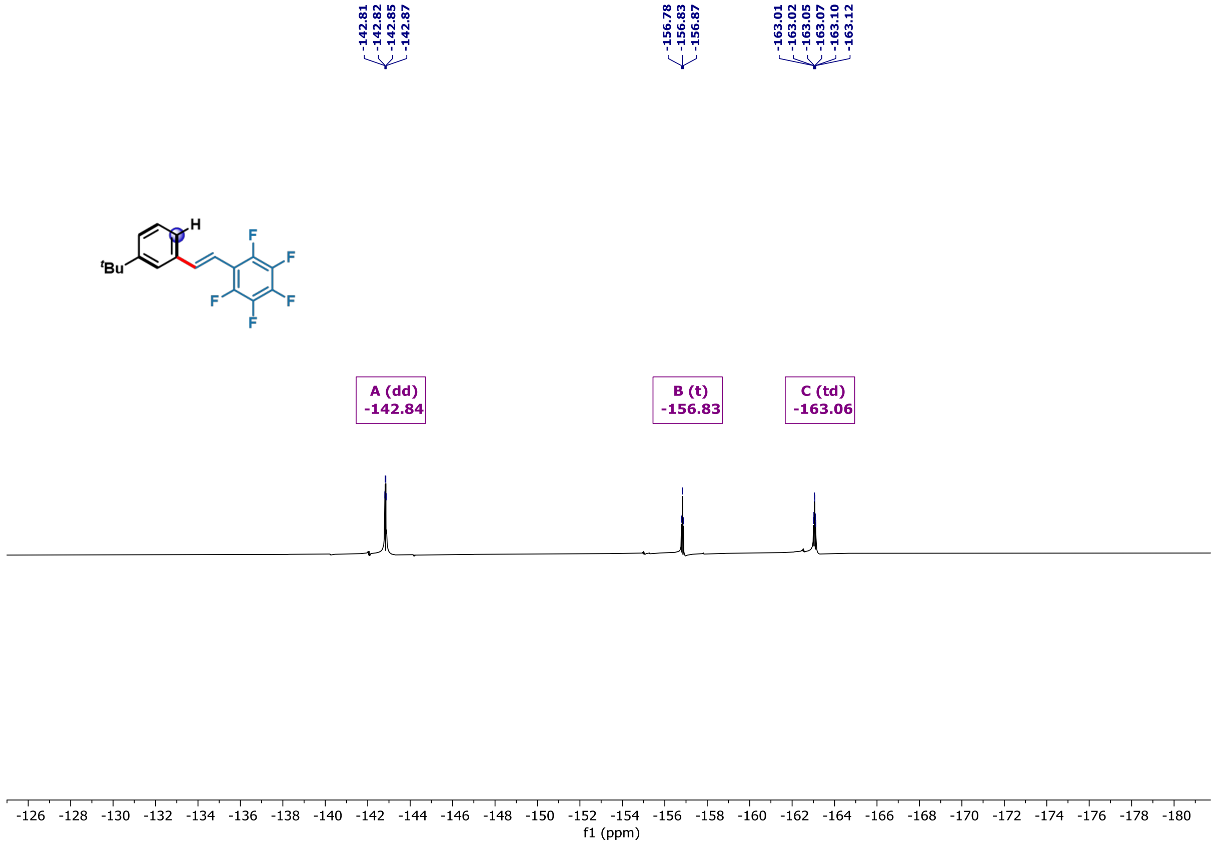
-142.81
-142.82
-142.85
-142.87
-156.78
-156.83
-156.87
-163.01
-163.02
-163.05
-163.07
-163.10
-163.12
tBu
H
F
F
F
F
F
A (dd)
-142.84
B (t)
-156.83
C (td)
-163.06
-126 -128 -130 -132 -134 -136 -138 -140 -142 -144 -146 -148 -150 -152 -154 -156 -158 -160 -162 -164 -166 -168 -170 -172 -174 -176 -178 -180
f1 (ppm)

(E)-1-(2-(perfluorophenyl)vinyl)naphthalene (48)

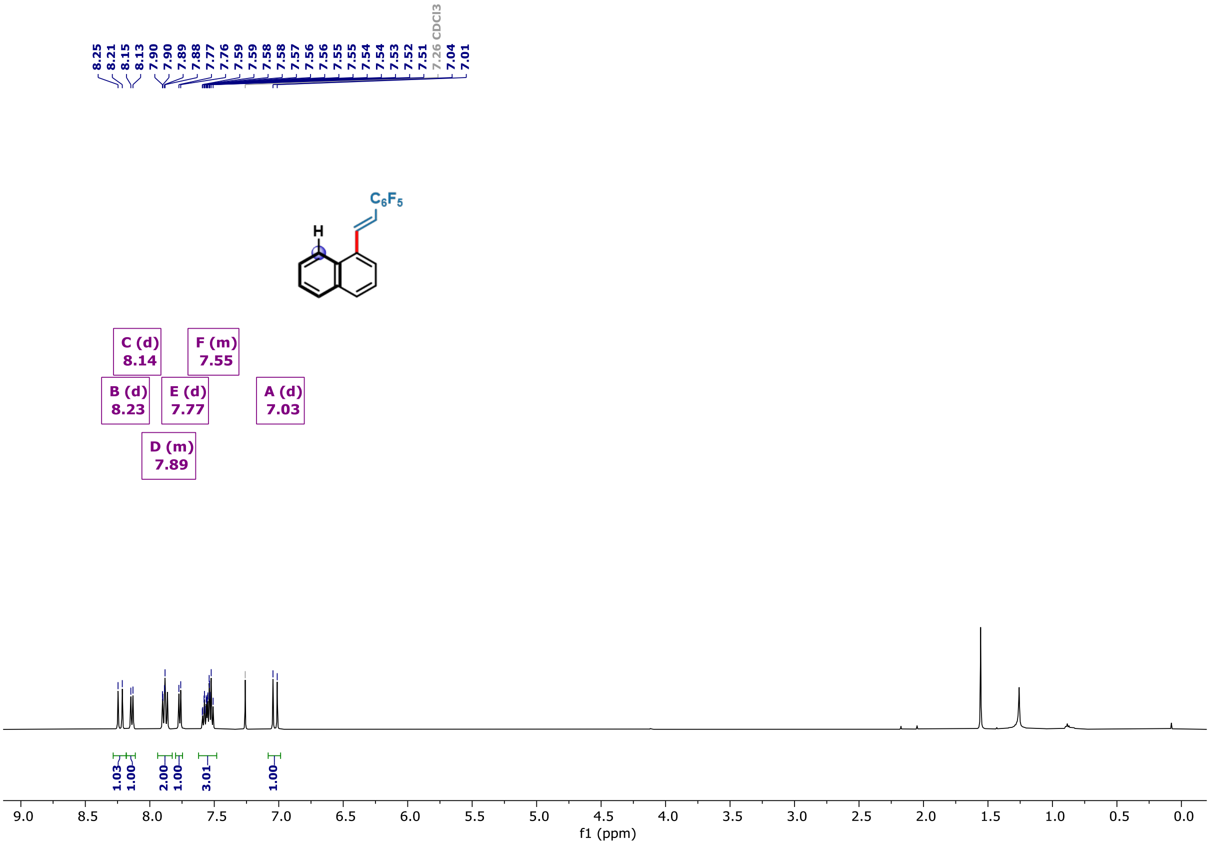

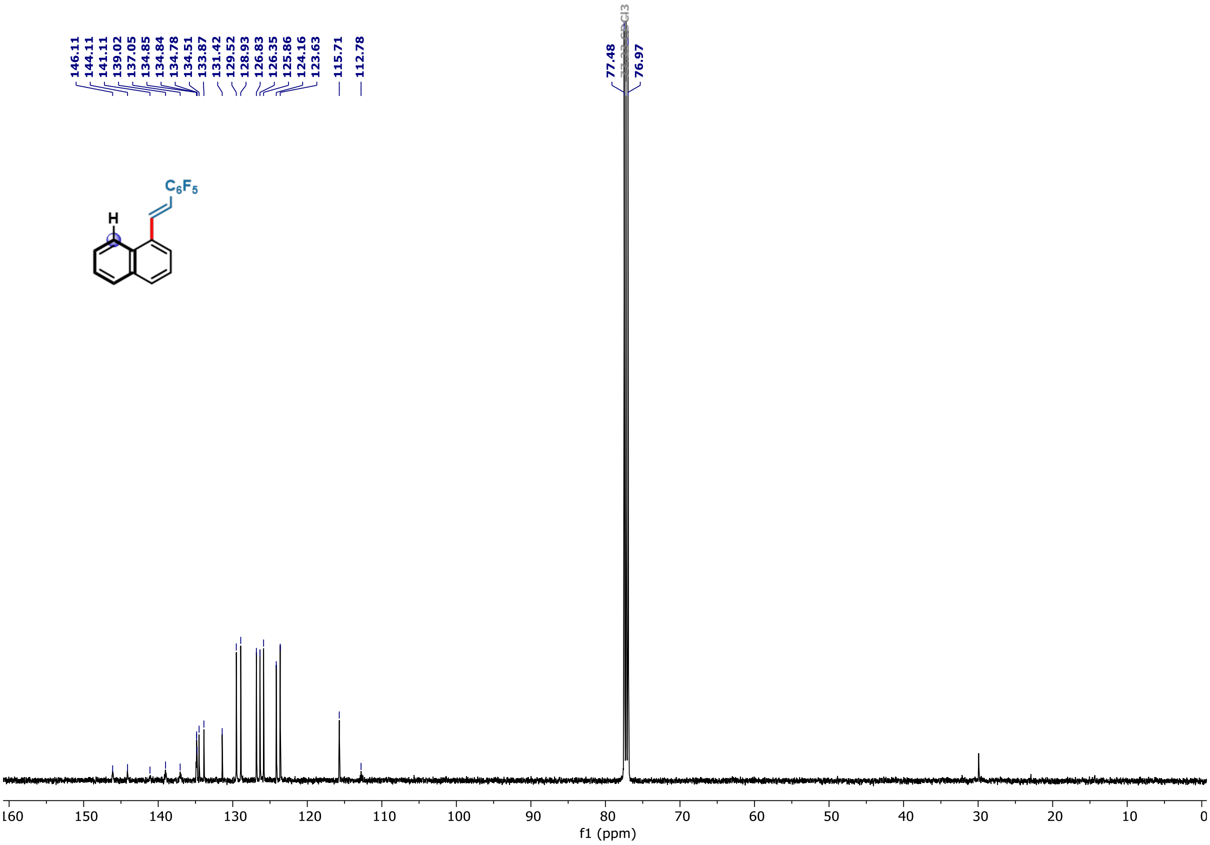

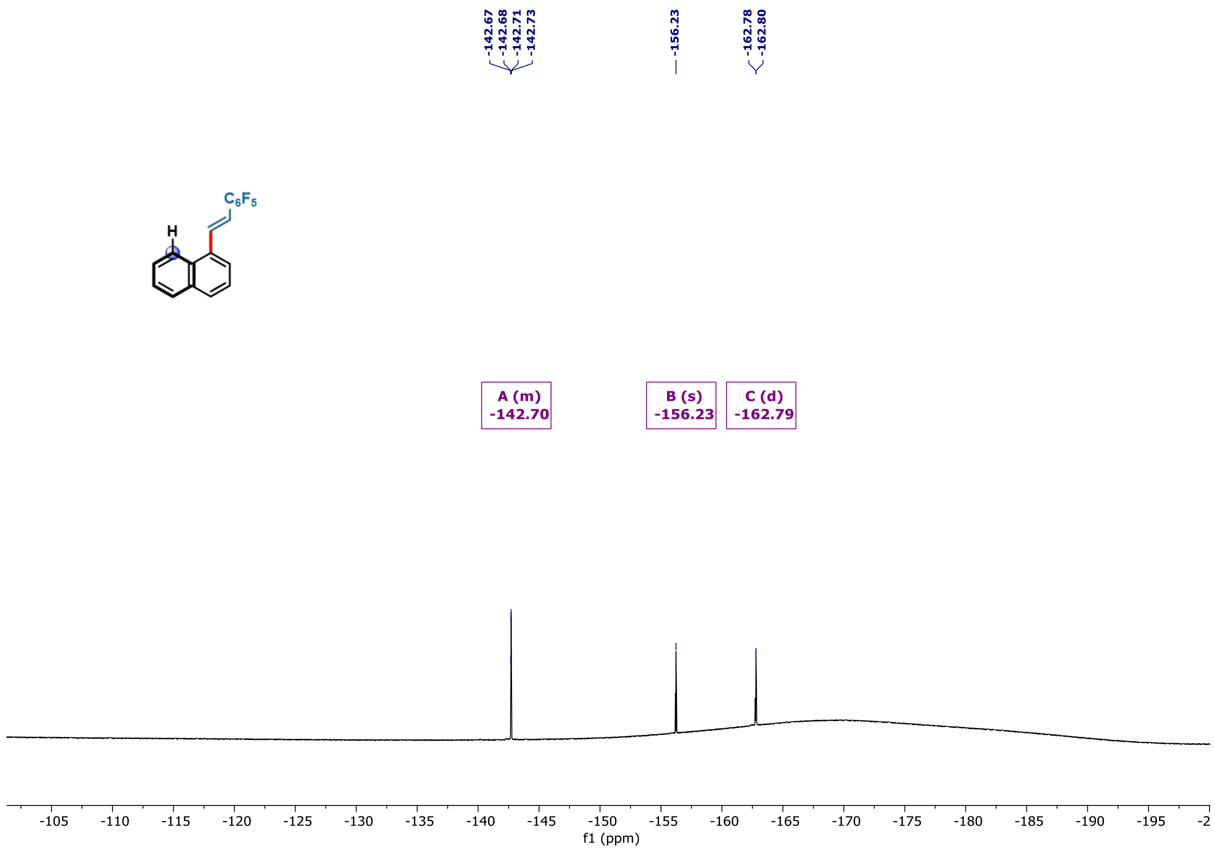
-142.67
-142.68
-142.71
-142.73
-156.23
-162.78
-162.80
C6F5
H
A (m)
-142.70
B (s)
-156.23
C (d)
-162.79
-105 -110 -115 -120 -125 -130 -135 -140 -145 -150 -155 -160 -165 -170 -175 -180 -185 -190 -195 -2
f1 (ppm)

(E)-1-(tert-butyl)-3-(4-nitrostyryl)benzene (49)

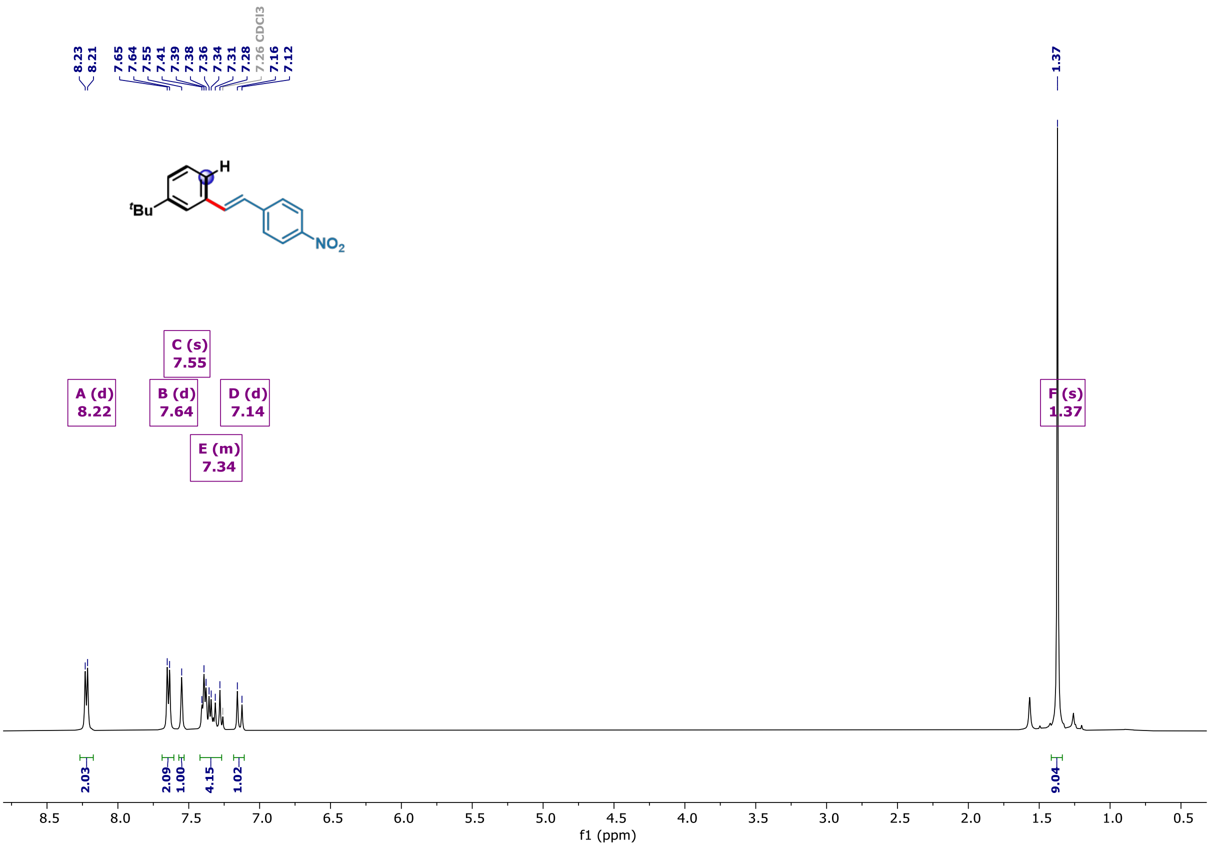

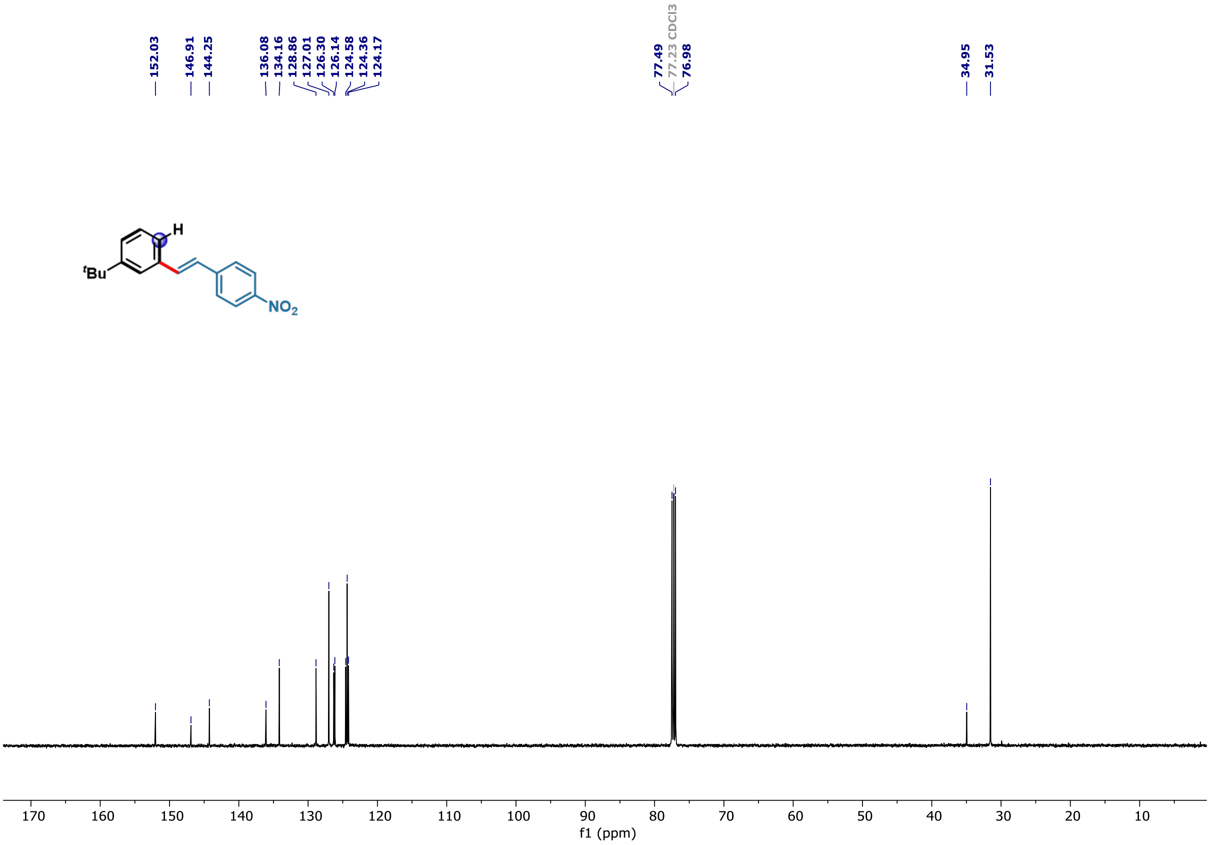

(E)-1,2-difluoro-4-styrylbenzene (50)

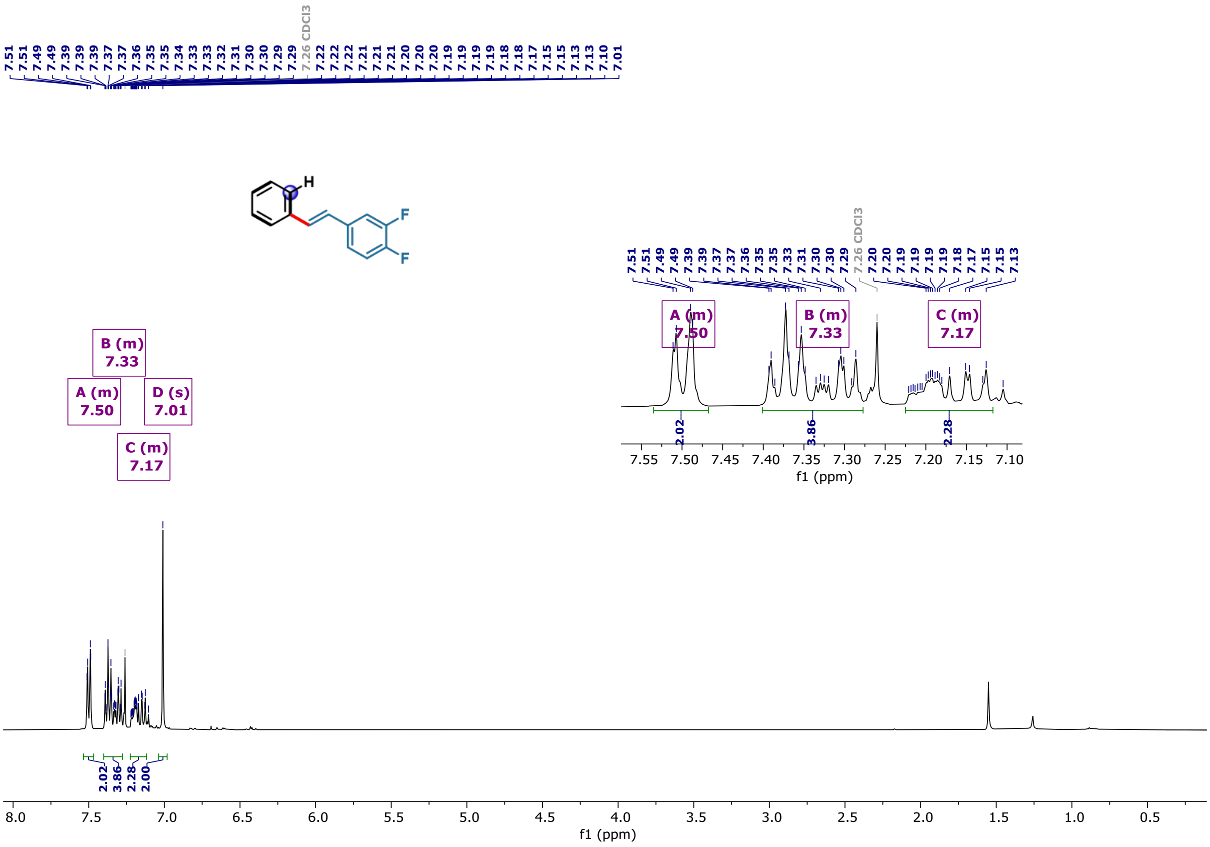

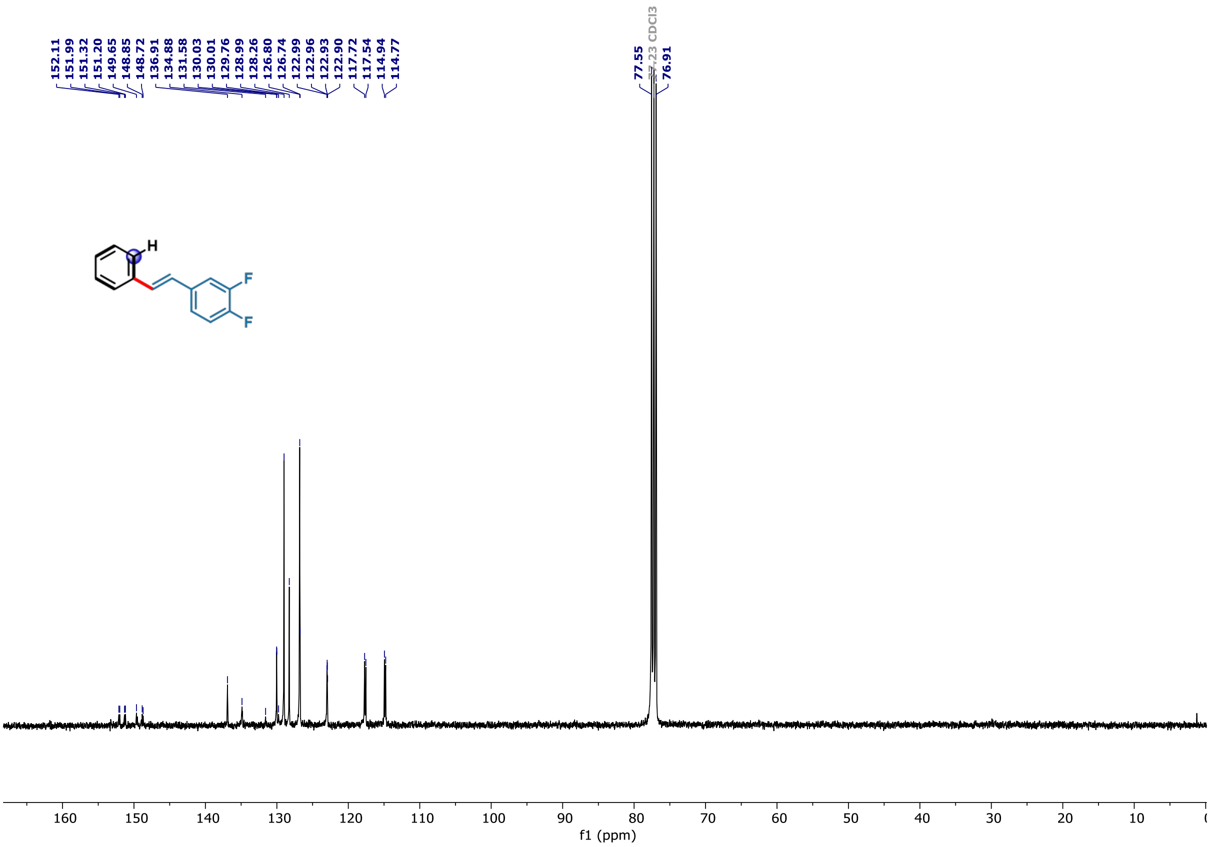

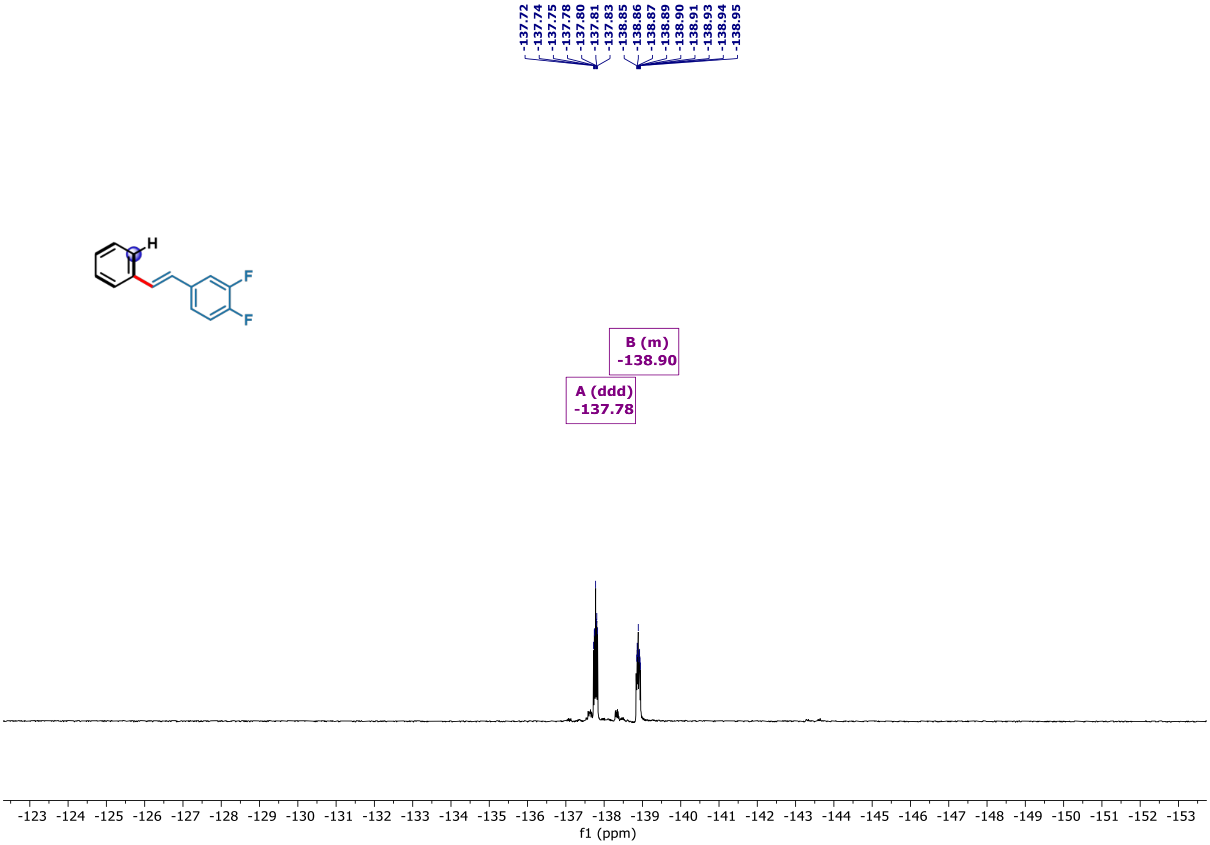
H
F
F
-137.72
-137.74
-137.75
-137.78
-137.80
-137.81
-137.83
-138.85
-138.86
-138.87
-138.89
-138.90
-138.91
-138.93
-138.94
-138.95
B (m)
-138.90
A (ddd)
-137.78
-123 -124 -125 -126 -127 -128 -129 -130 -131 -132 -133 -134 -135 -136 -137 -138 -139 -140 -141 -142 -143 -144 -145 -146 -147 -148 -149 -150 -151 -152 -153
f1 (ppm)

(E)-1-fluoro-4-styrylbenzene (51)

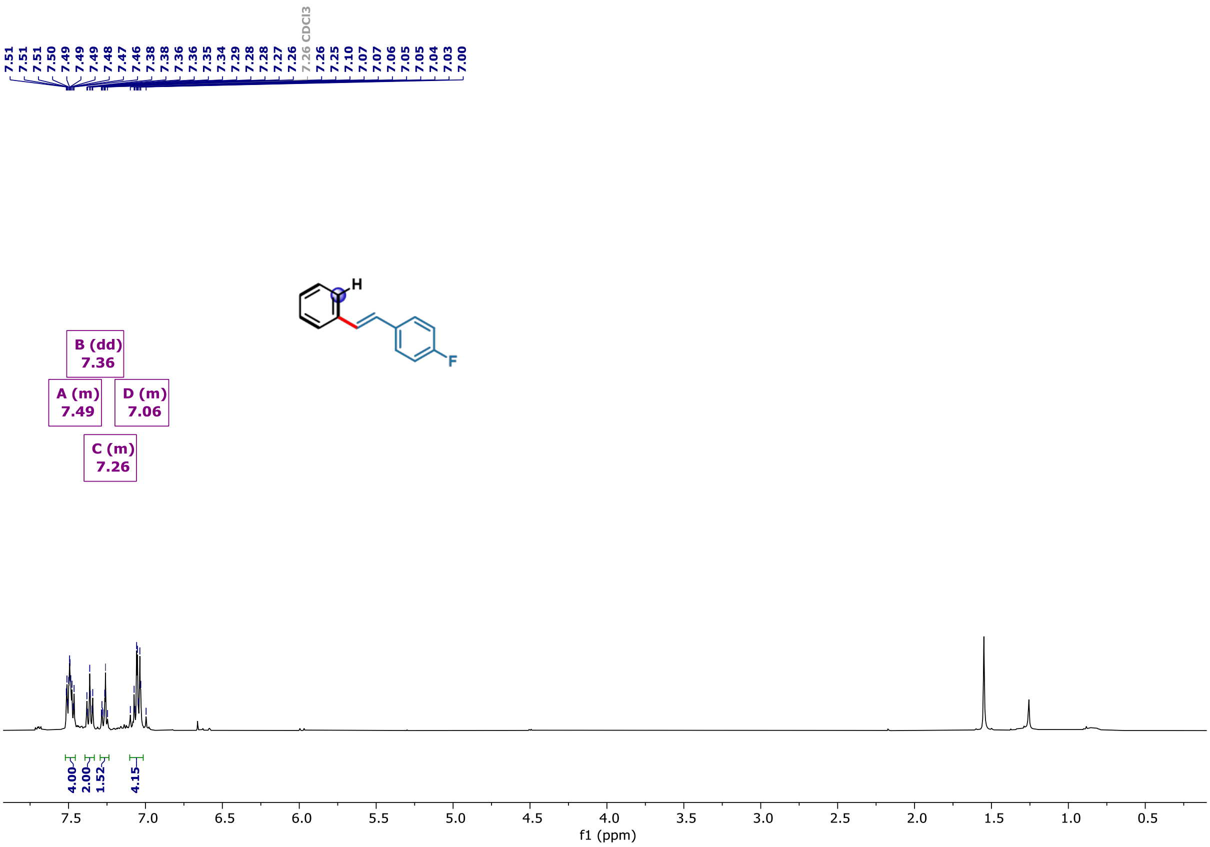

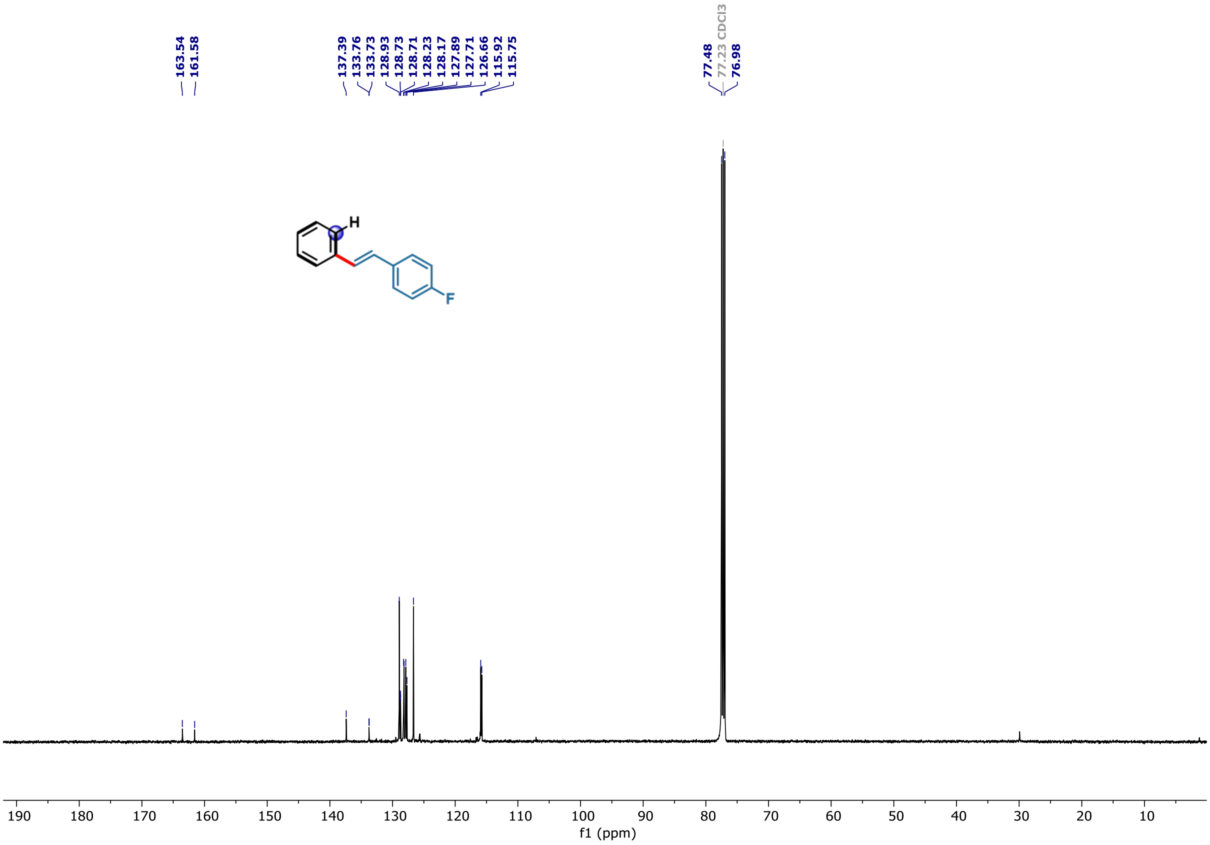

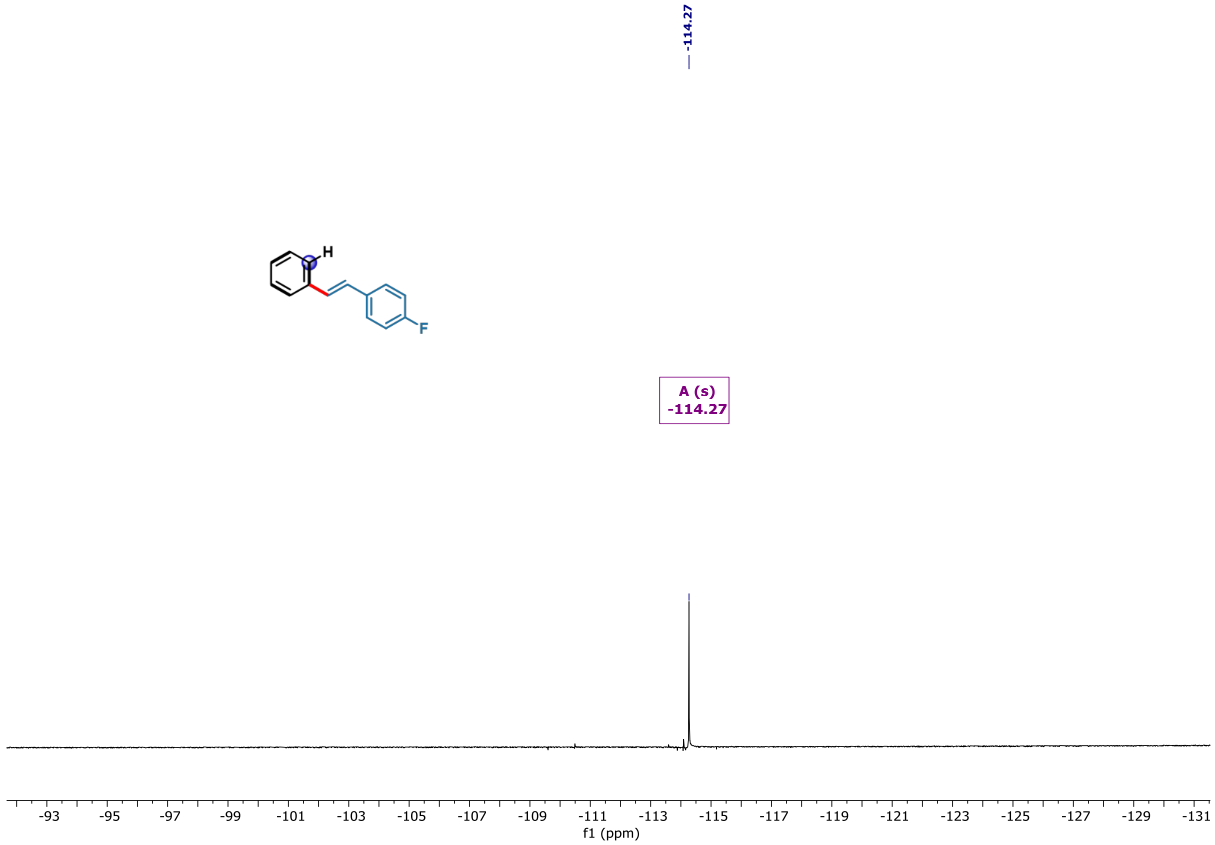
-114.27
H
F
A (s)
-114.27
-93
-95
-97
-99
-101
-103
-105
-107
-109
-111
-113
-115
-117
-119
-121
-123
-125
-127
-129
-131
f1 (ppm)

(E)-1-chloro-4-styrylbenzene (52)

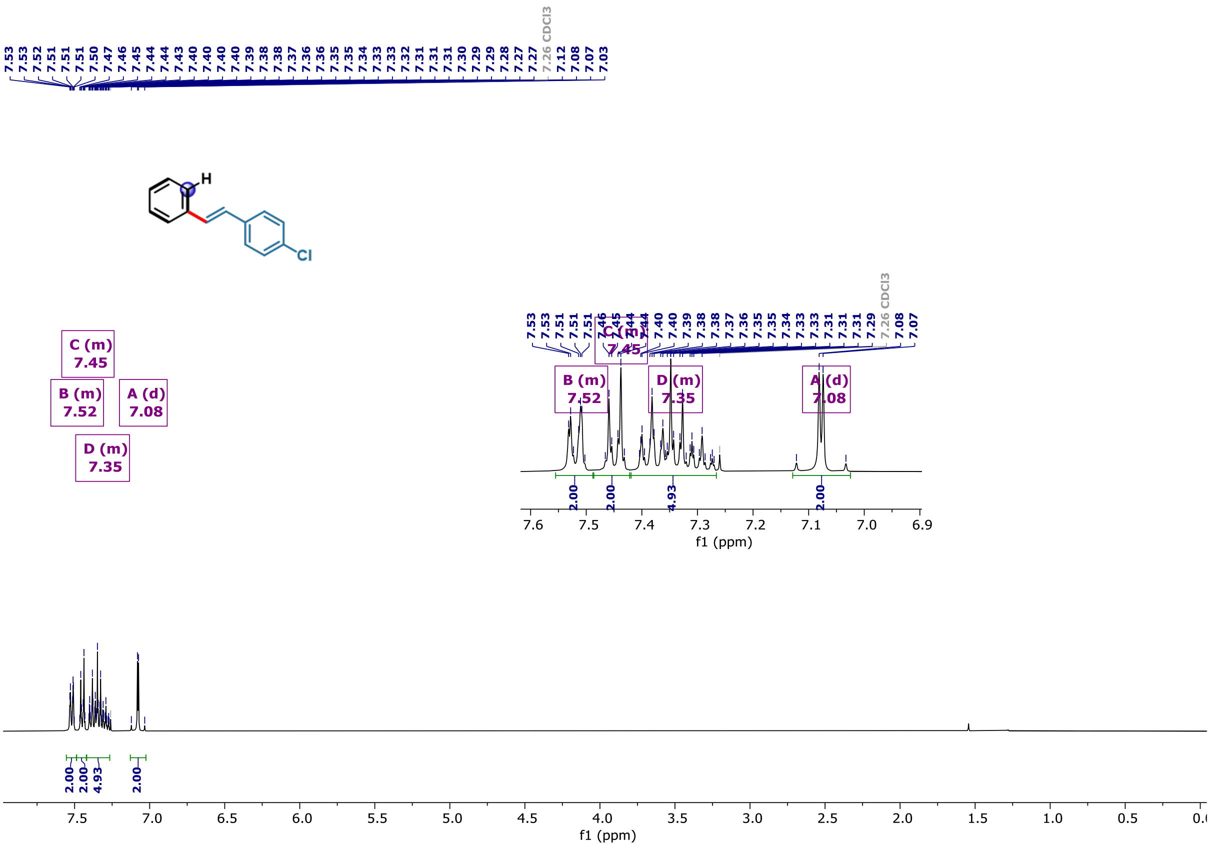

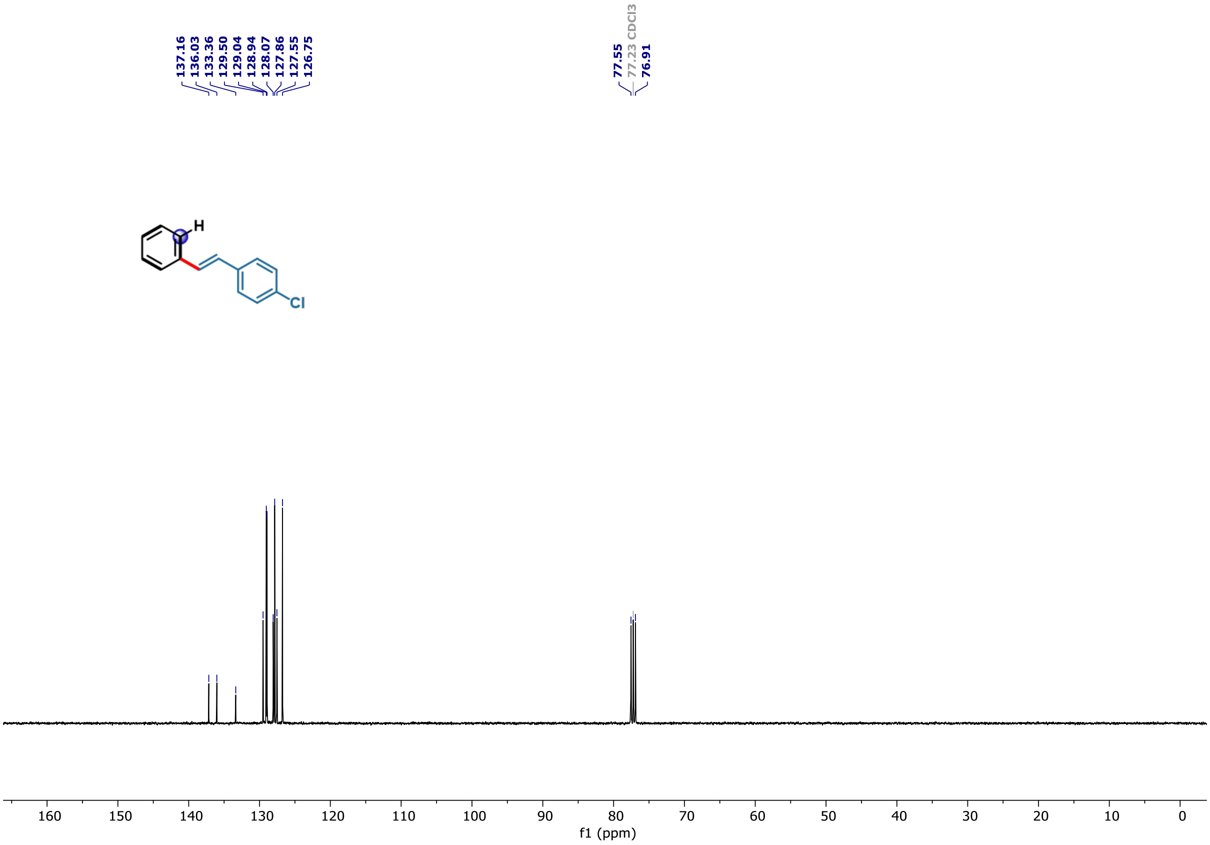

(E)-4-styrylphenyl acetate (53)

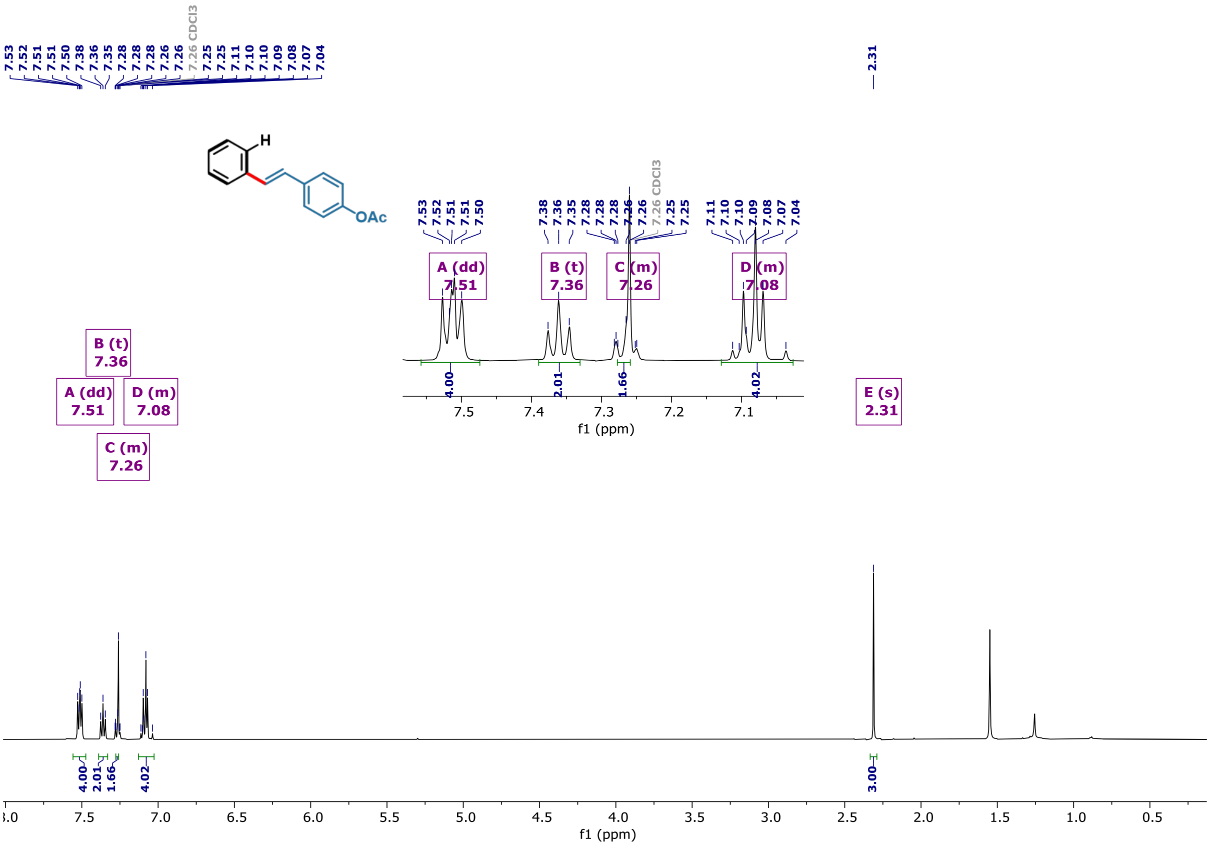

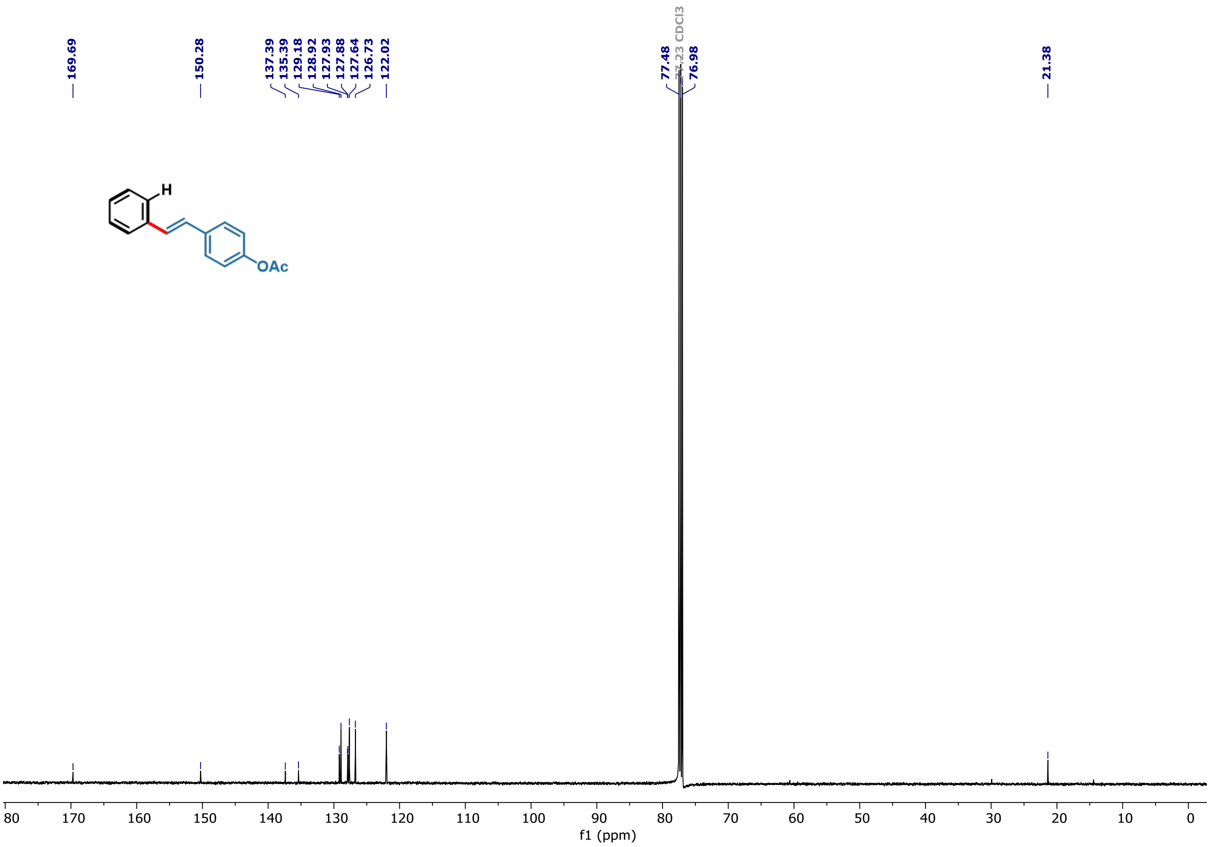

(E)-1,2-diphenylethene (54)

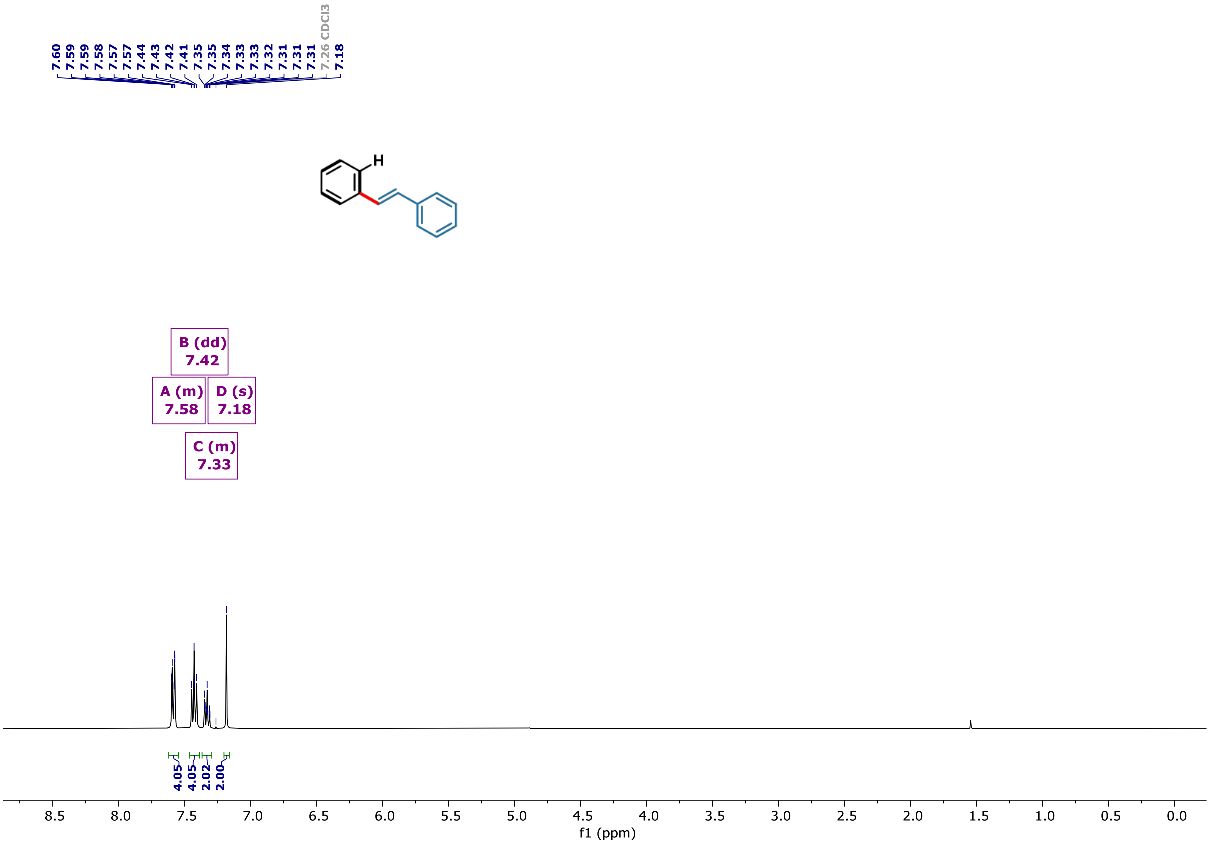

Ethene-1,1,2-triyltribenzene (55)

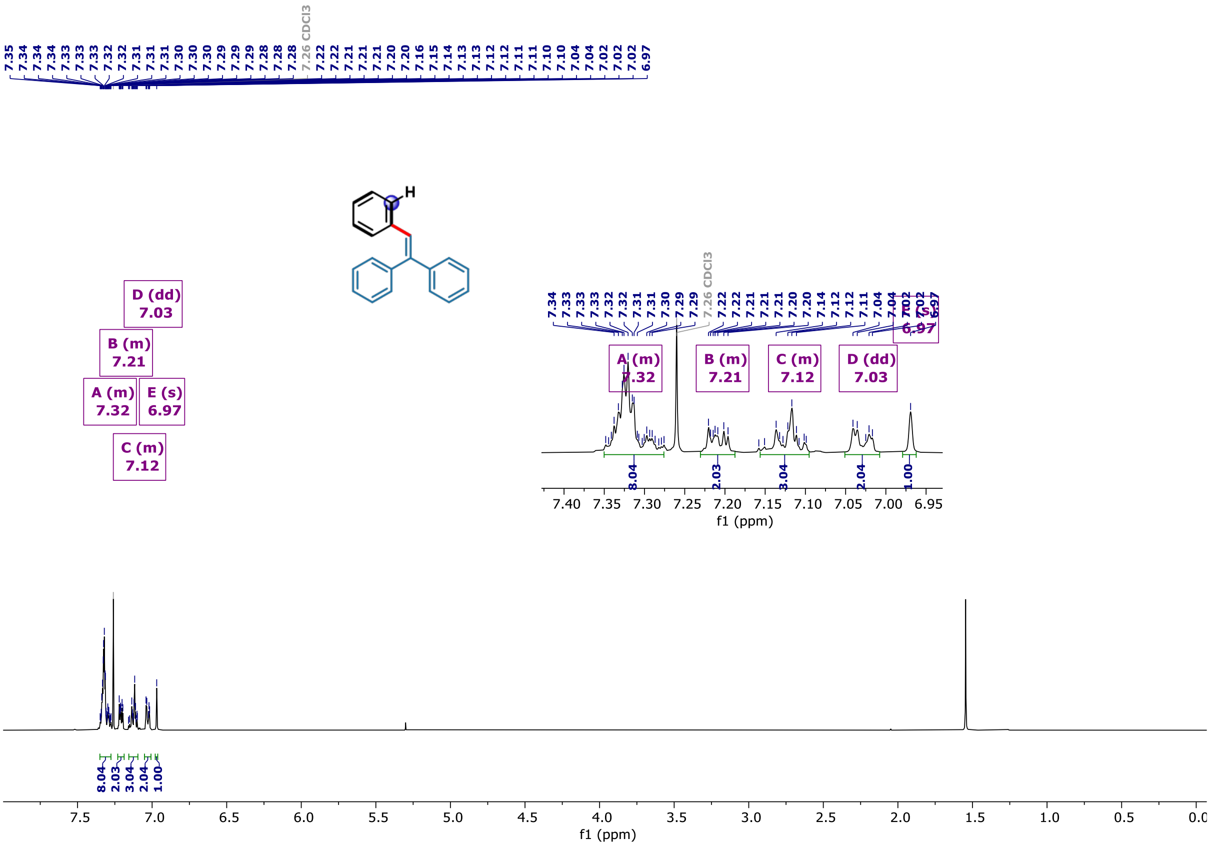

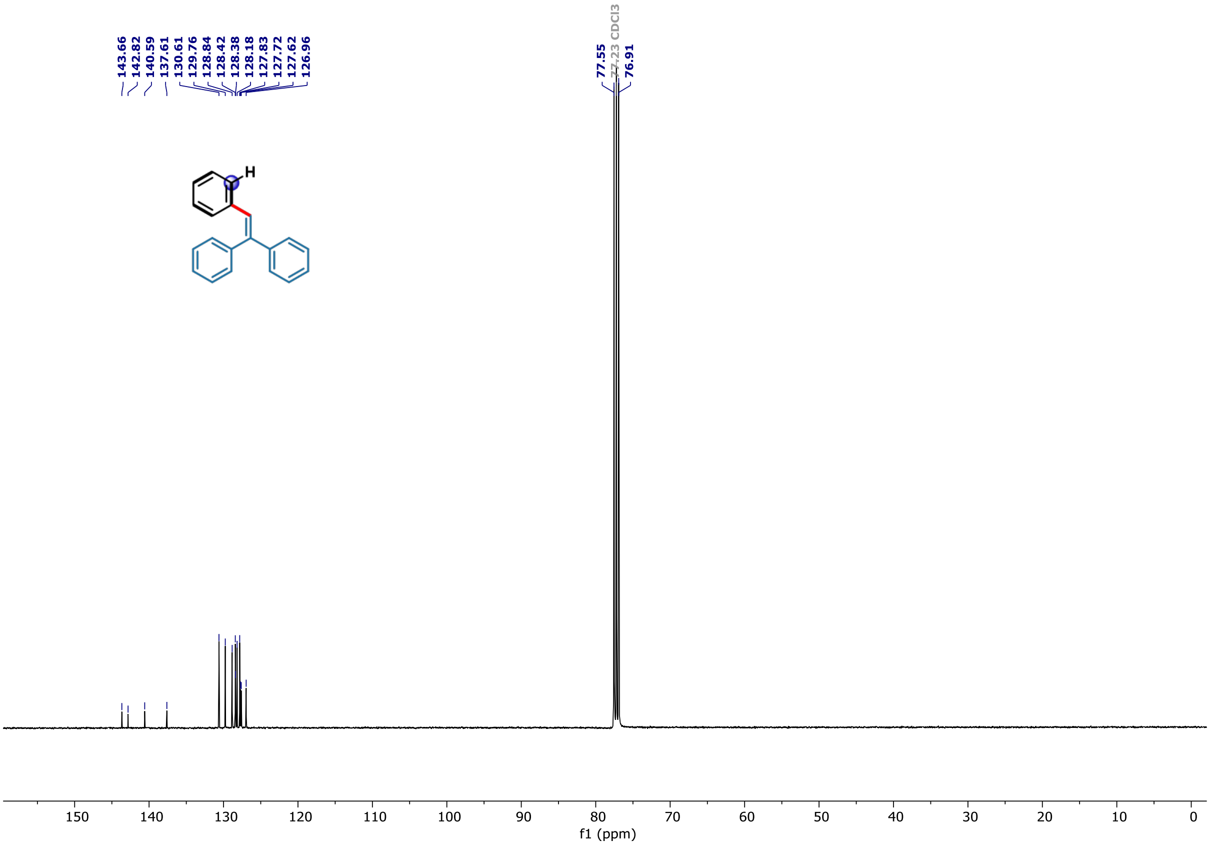

(E)-4-phenylbut-3-en-2-one (56)

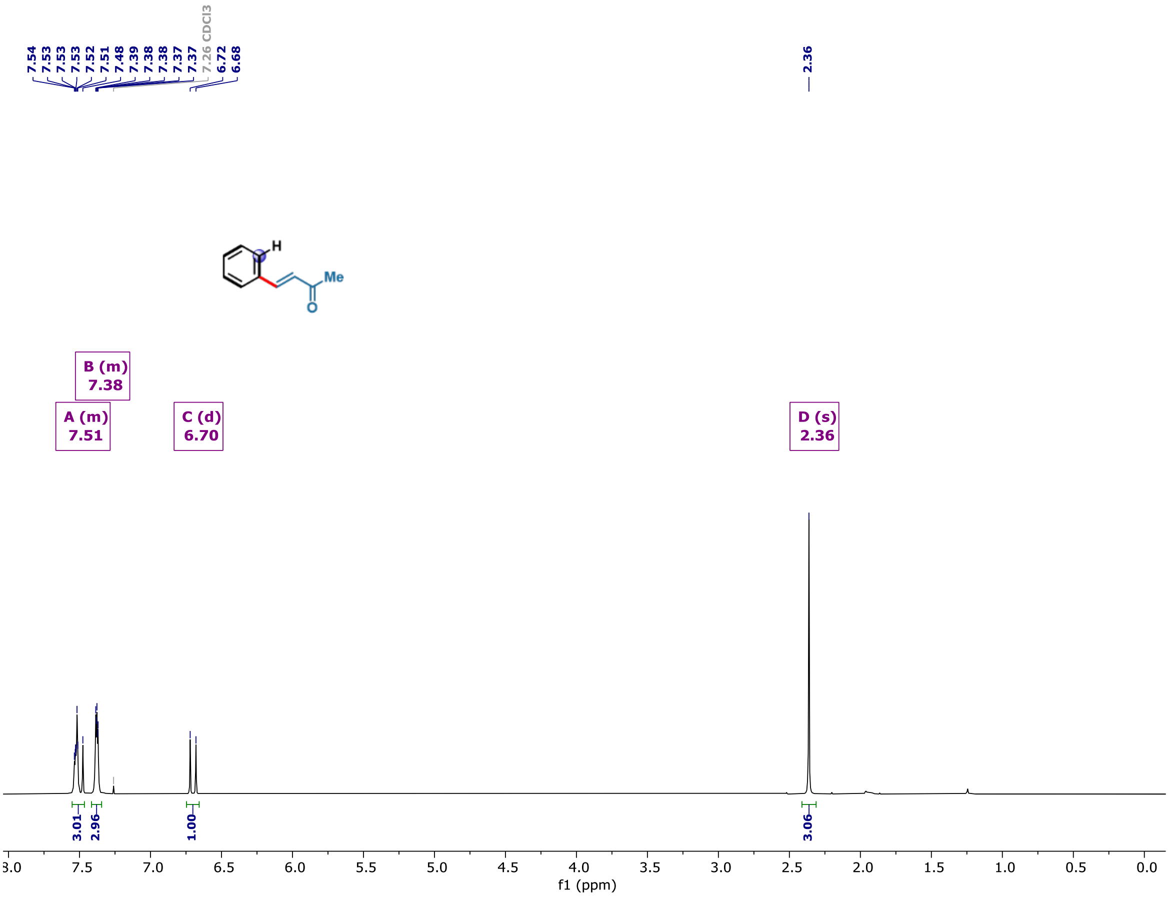

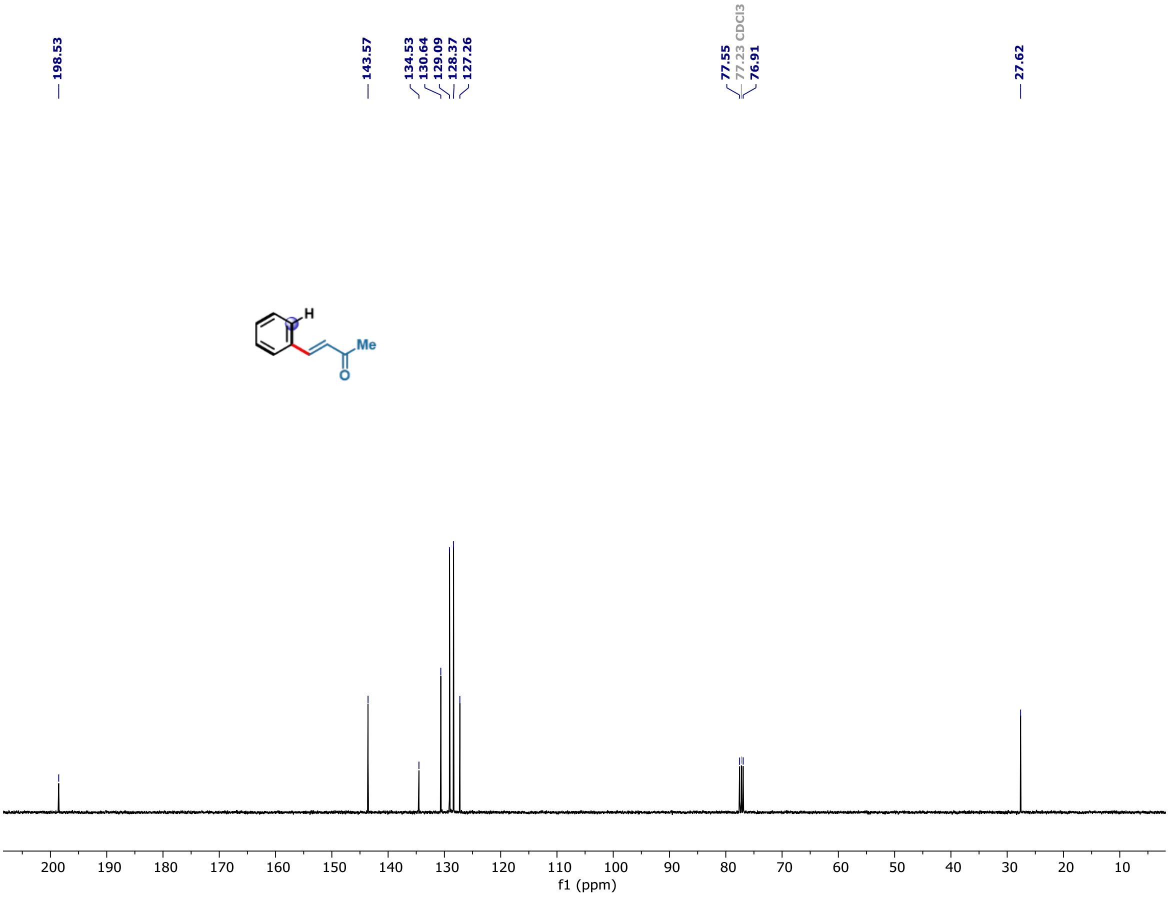

(E)-4-(m-tolyl)but-3-en-2-one (57)

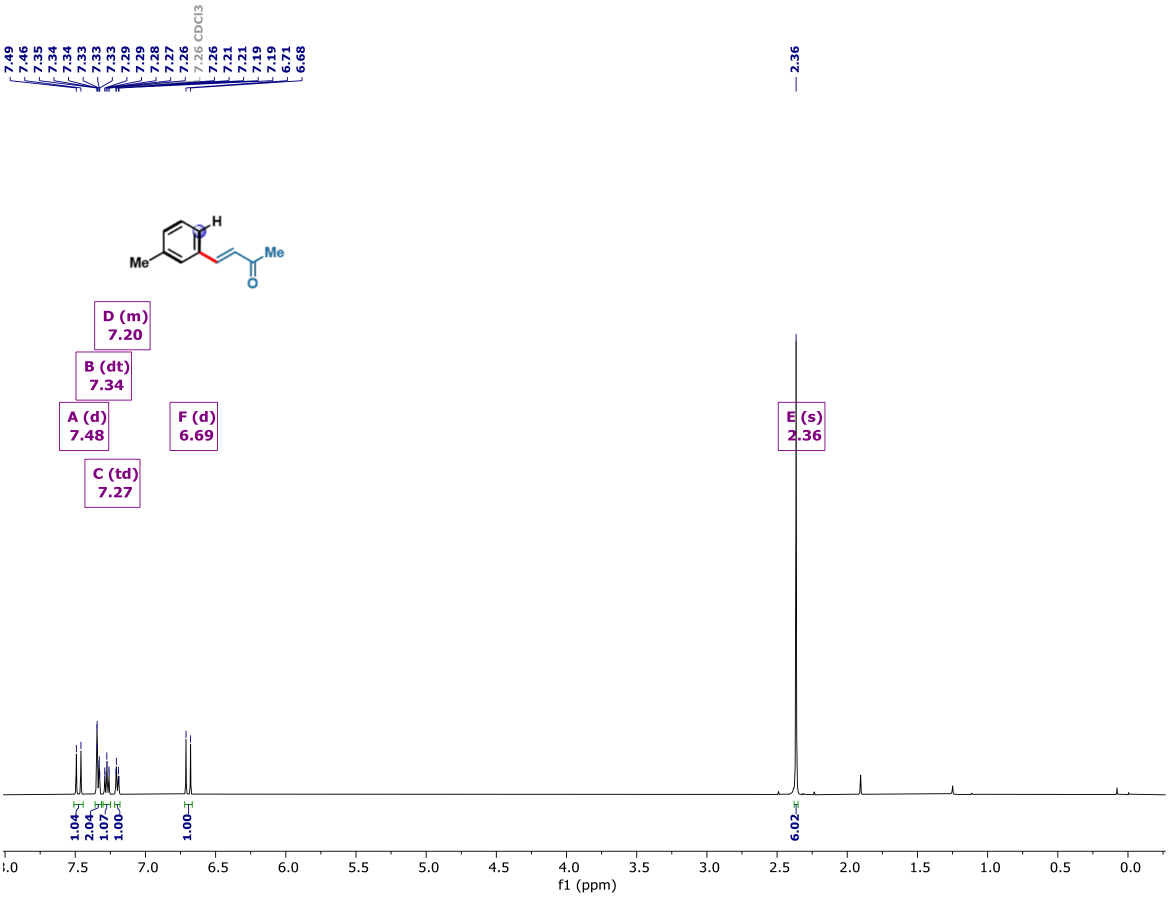

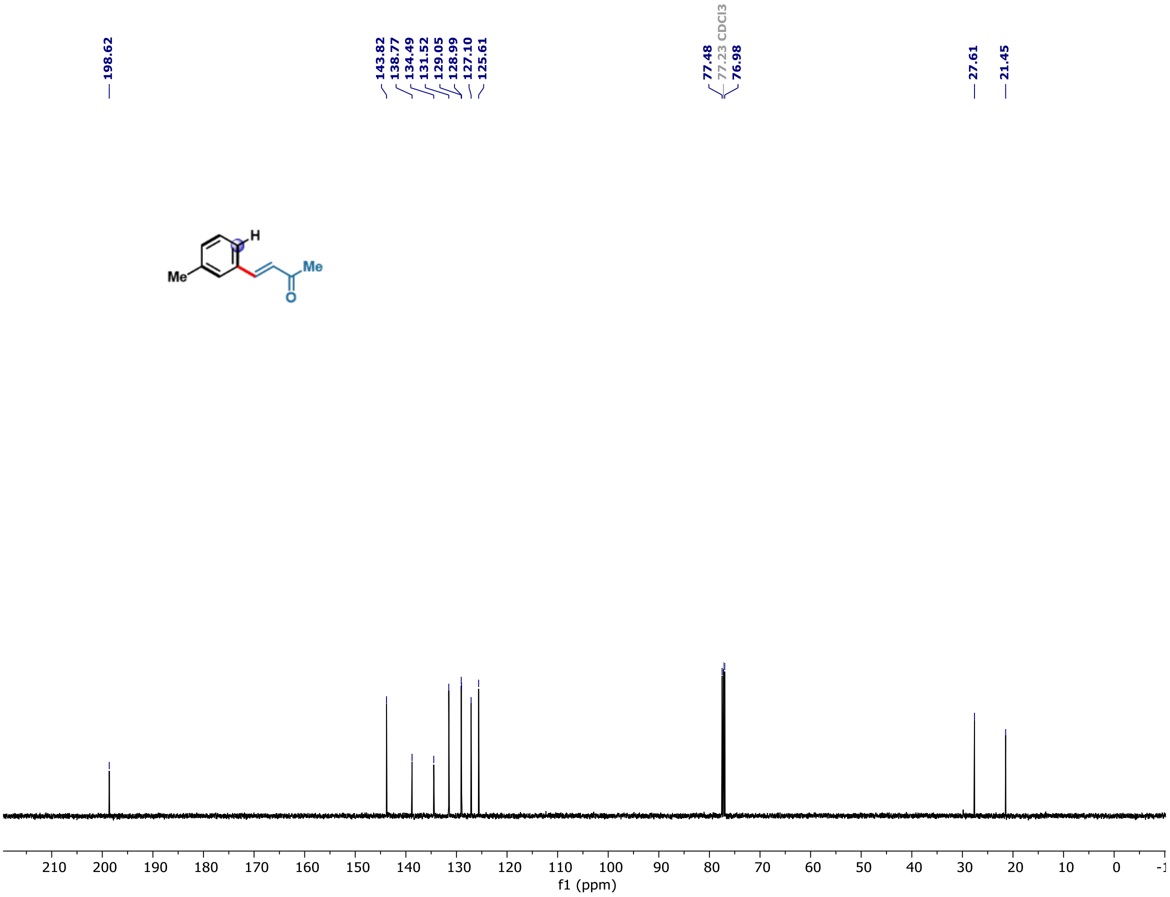

(E)-4-(p-tolyl)but-3-en-2-one (58)

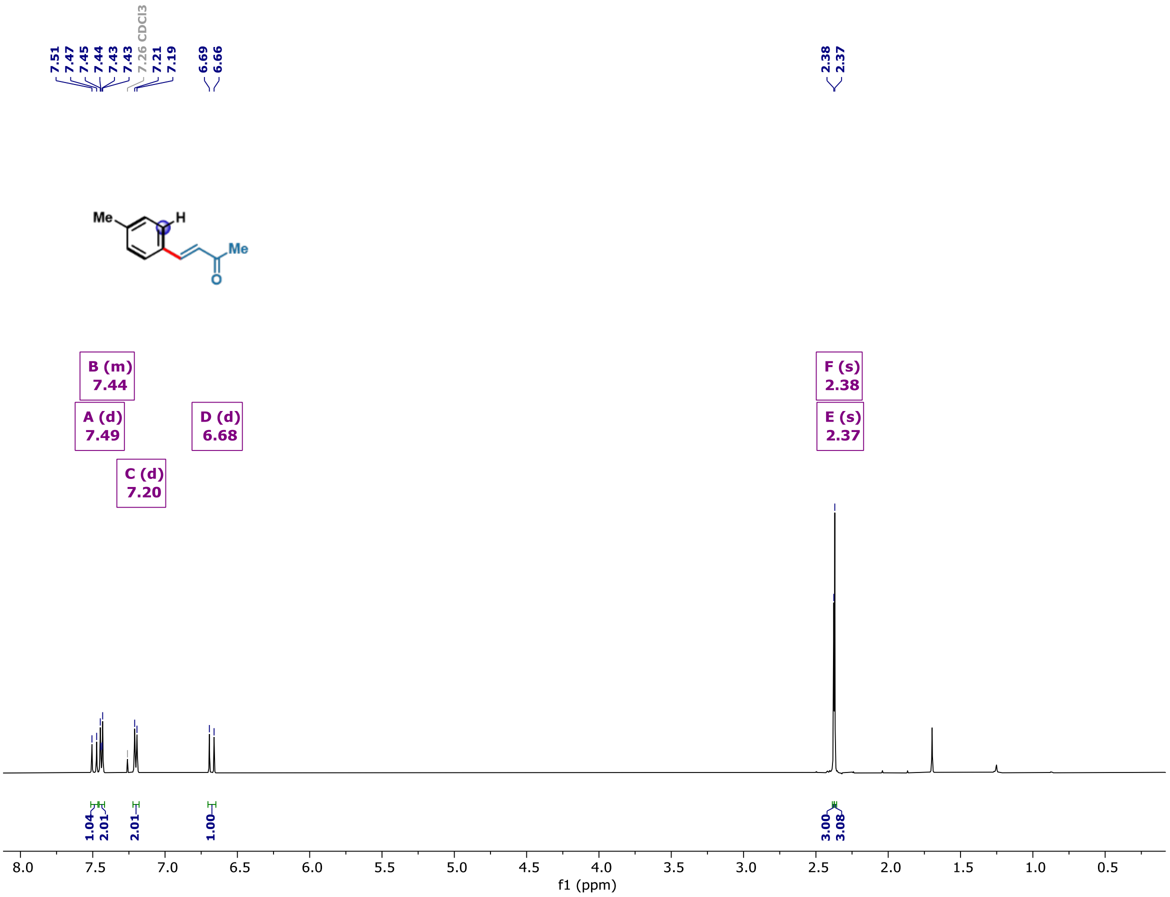

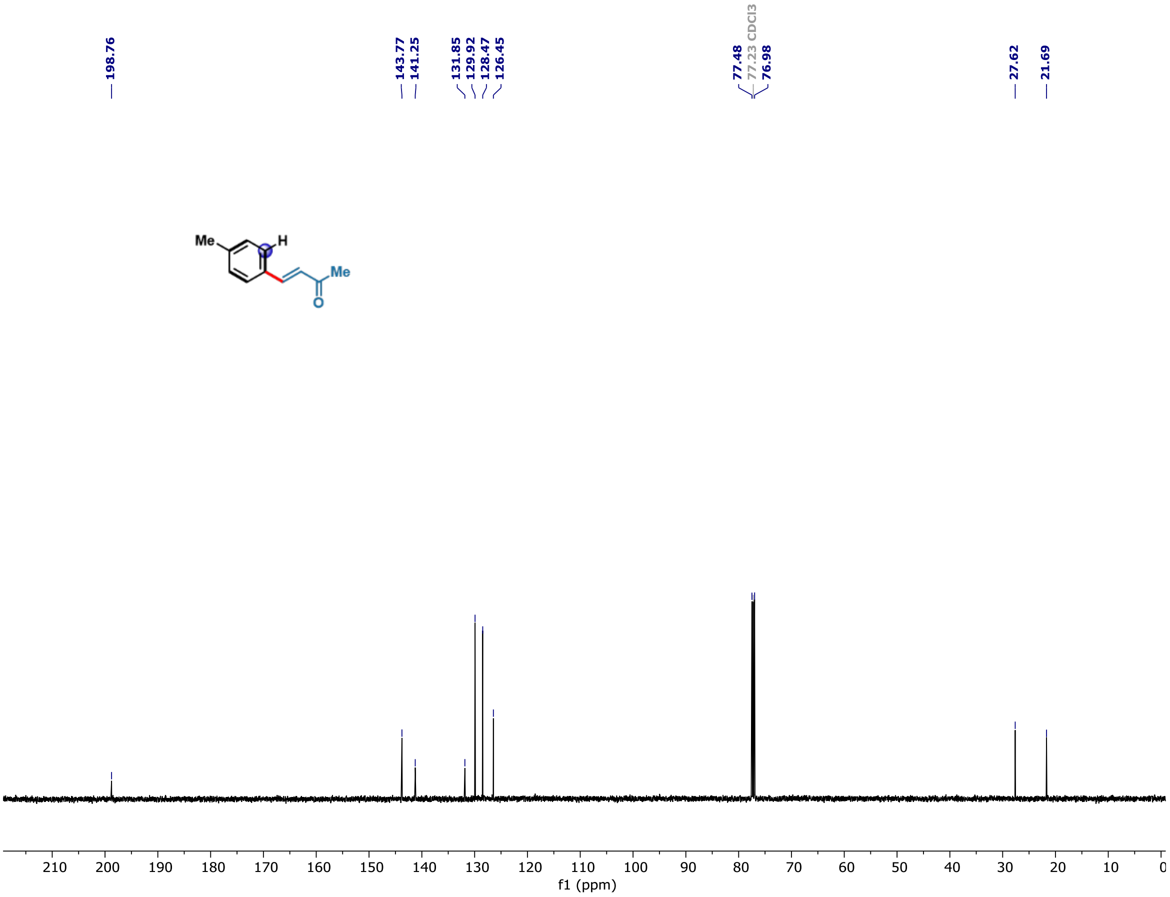

(E)-4-(3-(trifluoromethyl)phenyl)but-3-en-2-one (59)

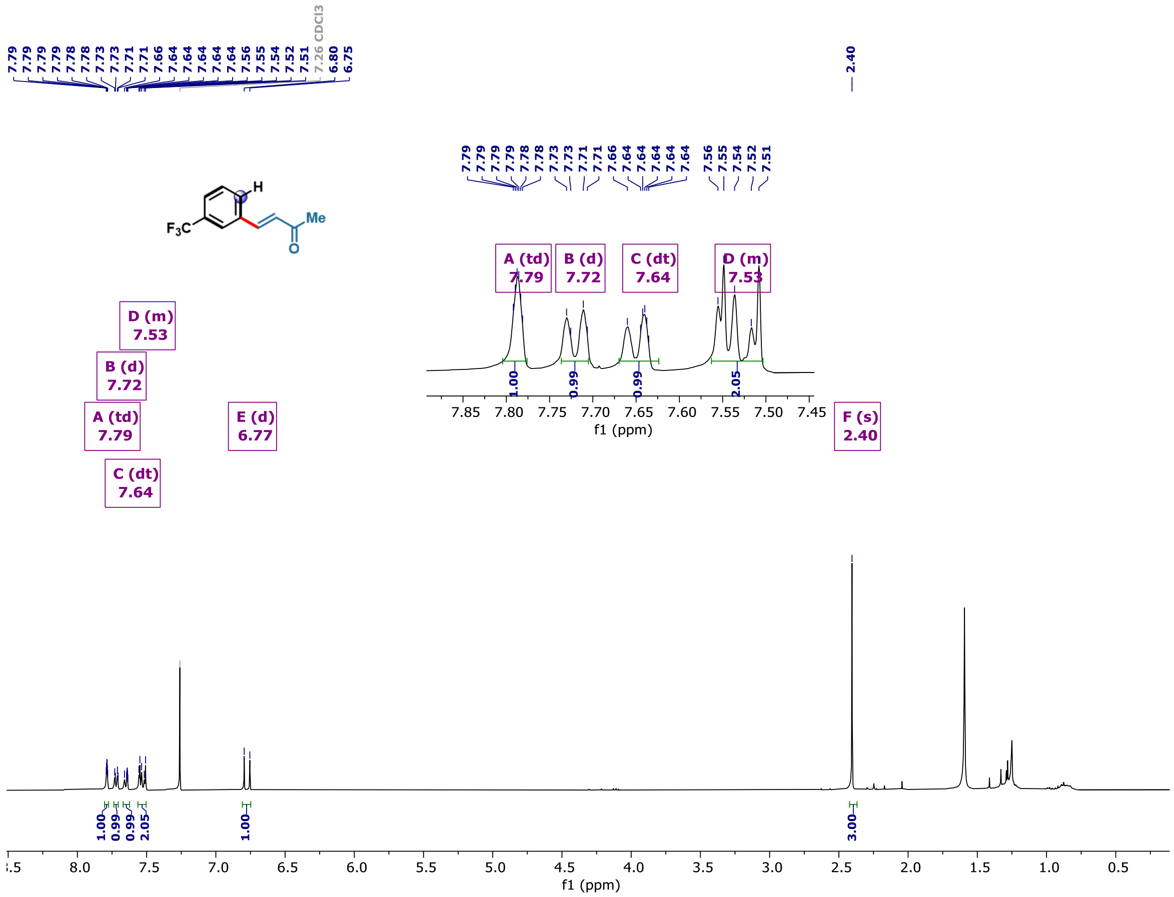

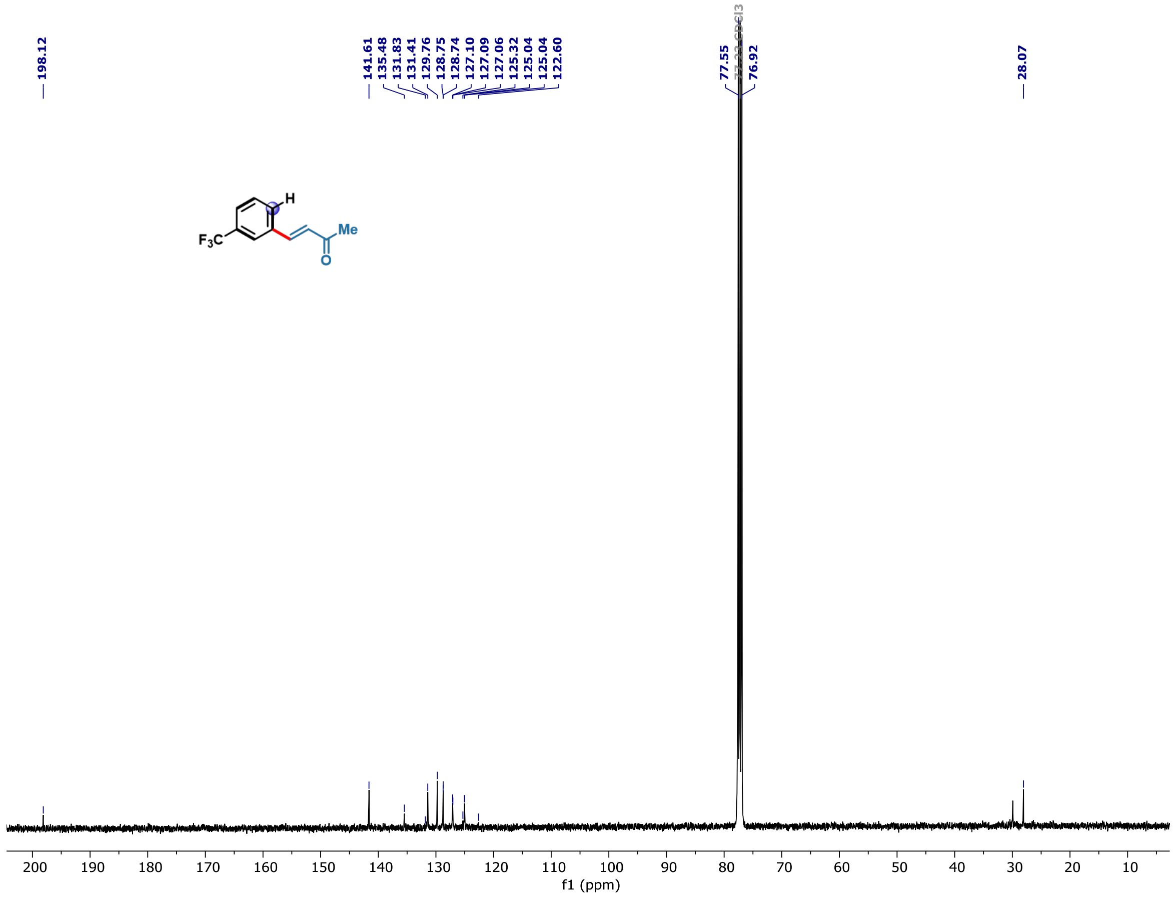

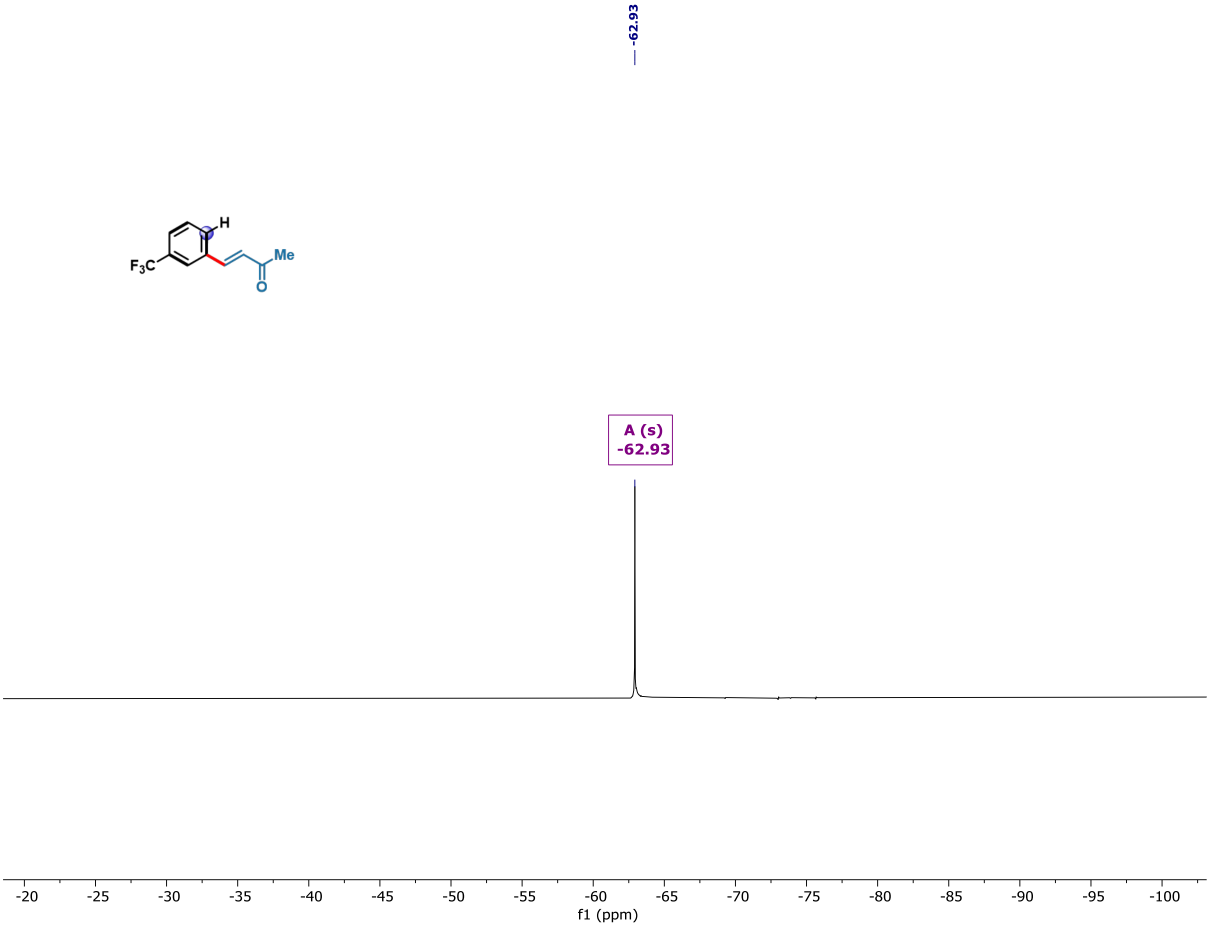
-62.93
H
F3C
Me
O
A (s)
-62.93
-20 -25 -30 -35 -40 -45 -50 -55 -60 -65 -70 -75 -80 -85 -90 -95 -100
f1 (ppm)

(E)-4-(3-pentylphenyl)but-3-en-2-one (60)

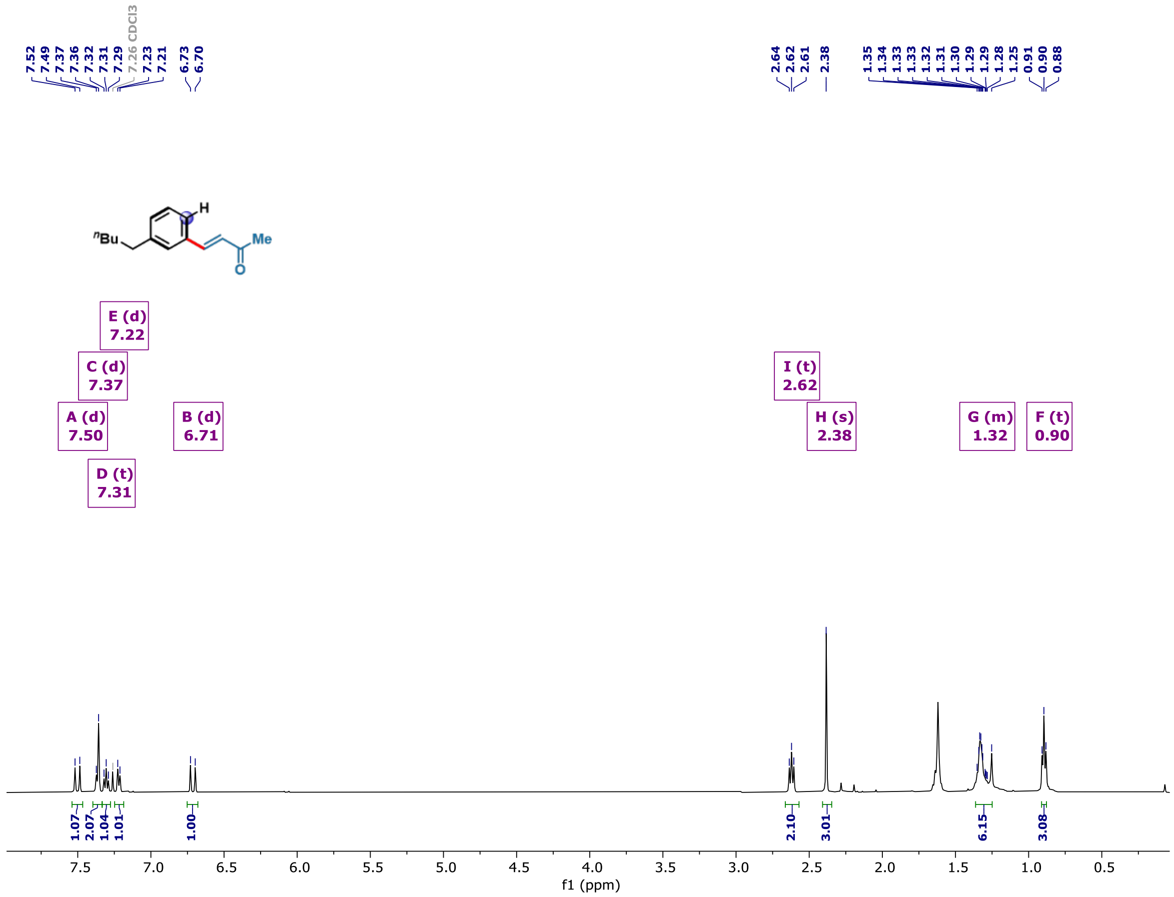

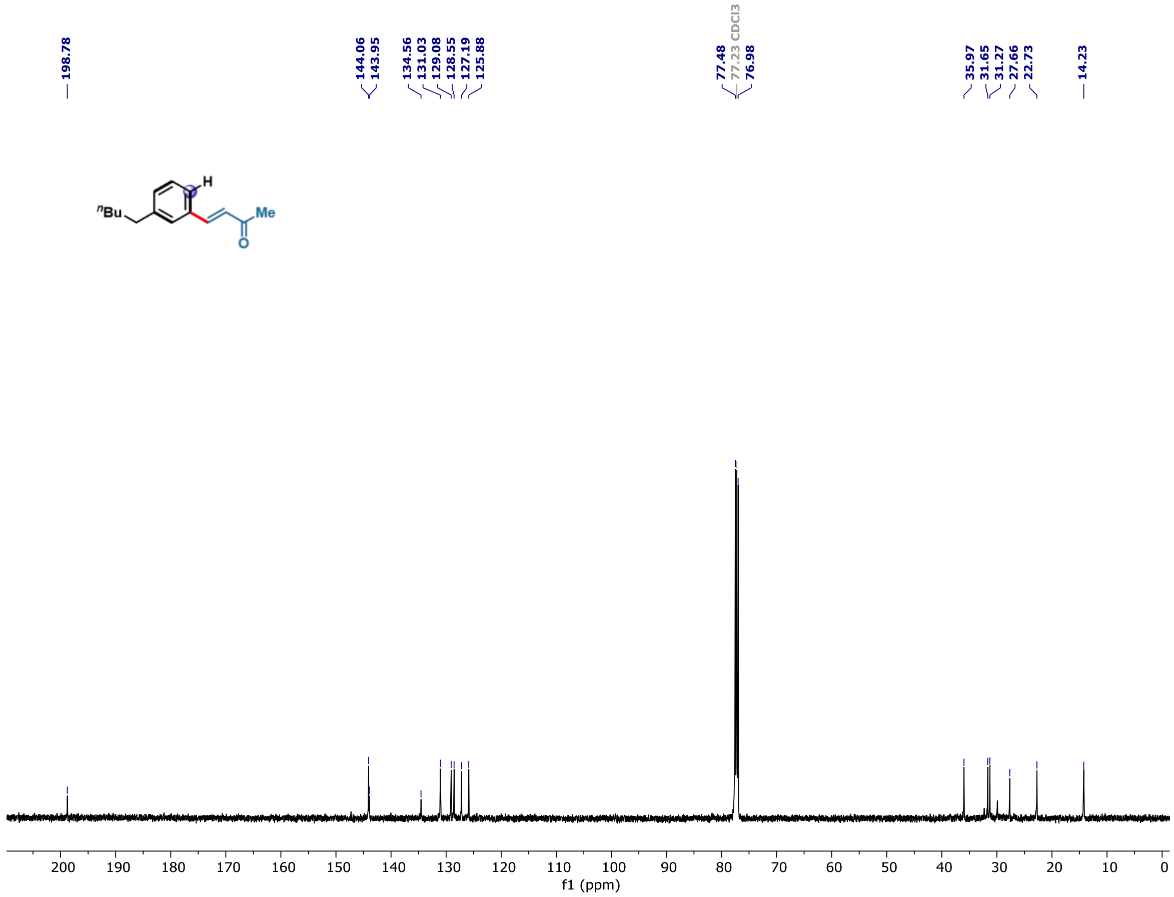

Methyl (E)-3-(3-oxobut-1-en-1-yl)benzoate (61)

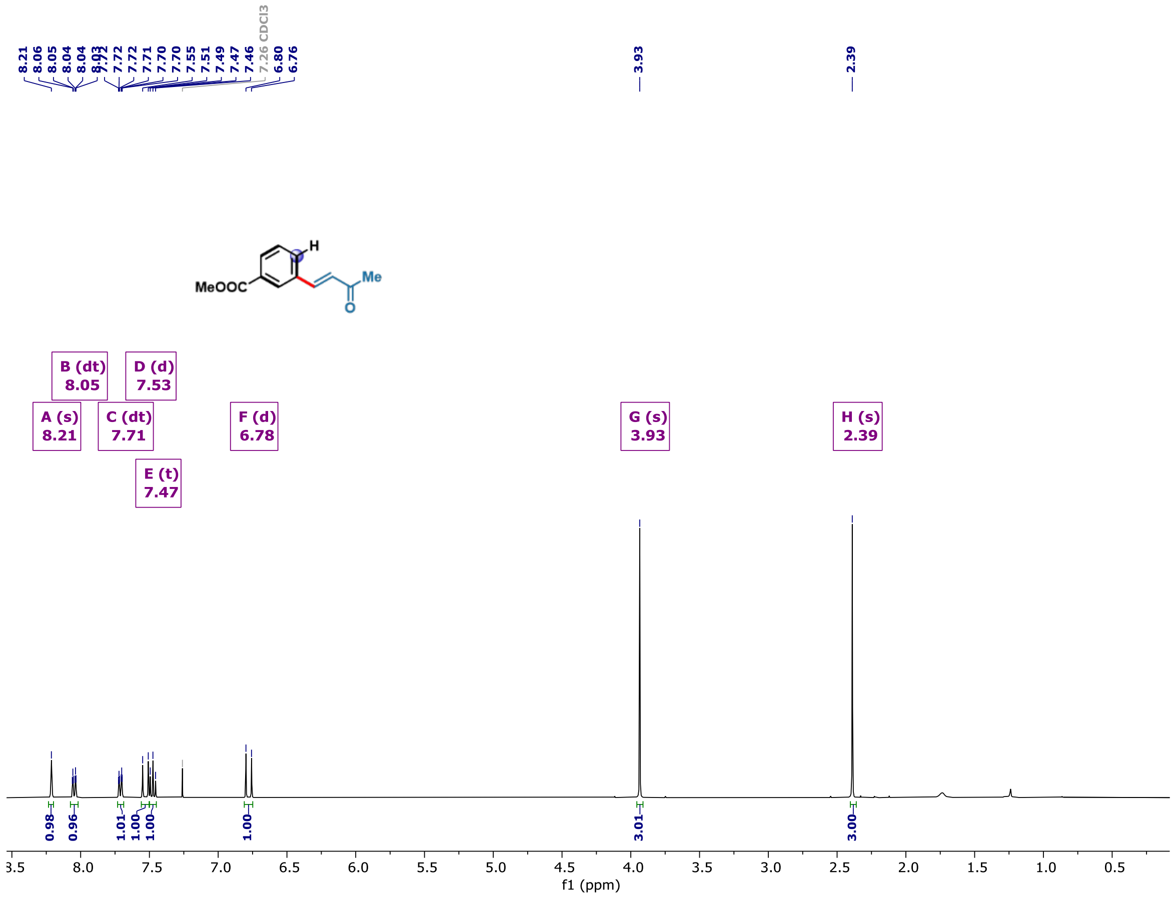

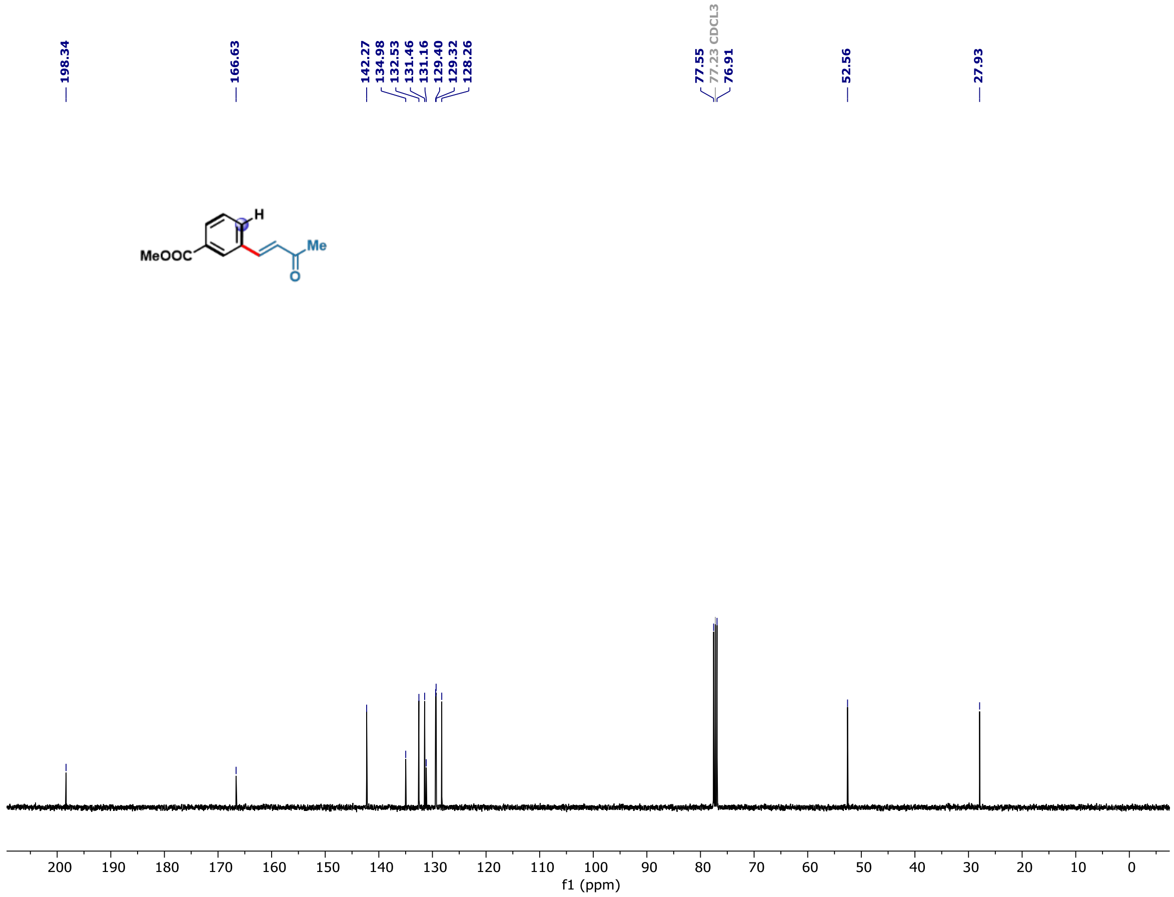

(E)-4-(4'-chloro-[1,1'-biphenyl]-3-yl)but-3-en-2-one (62)

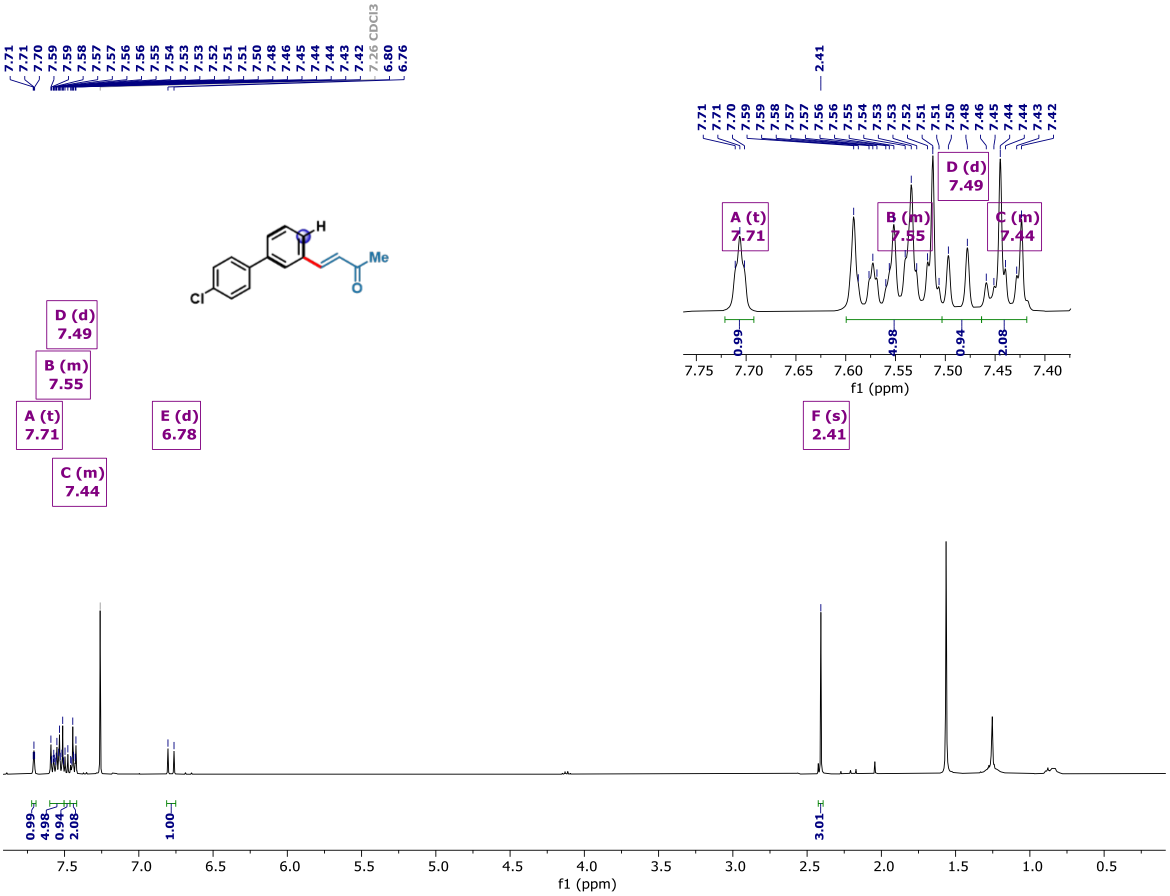

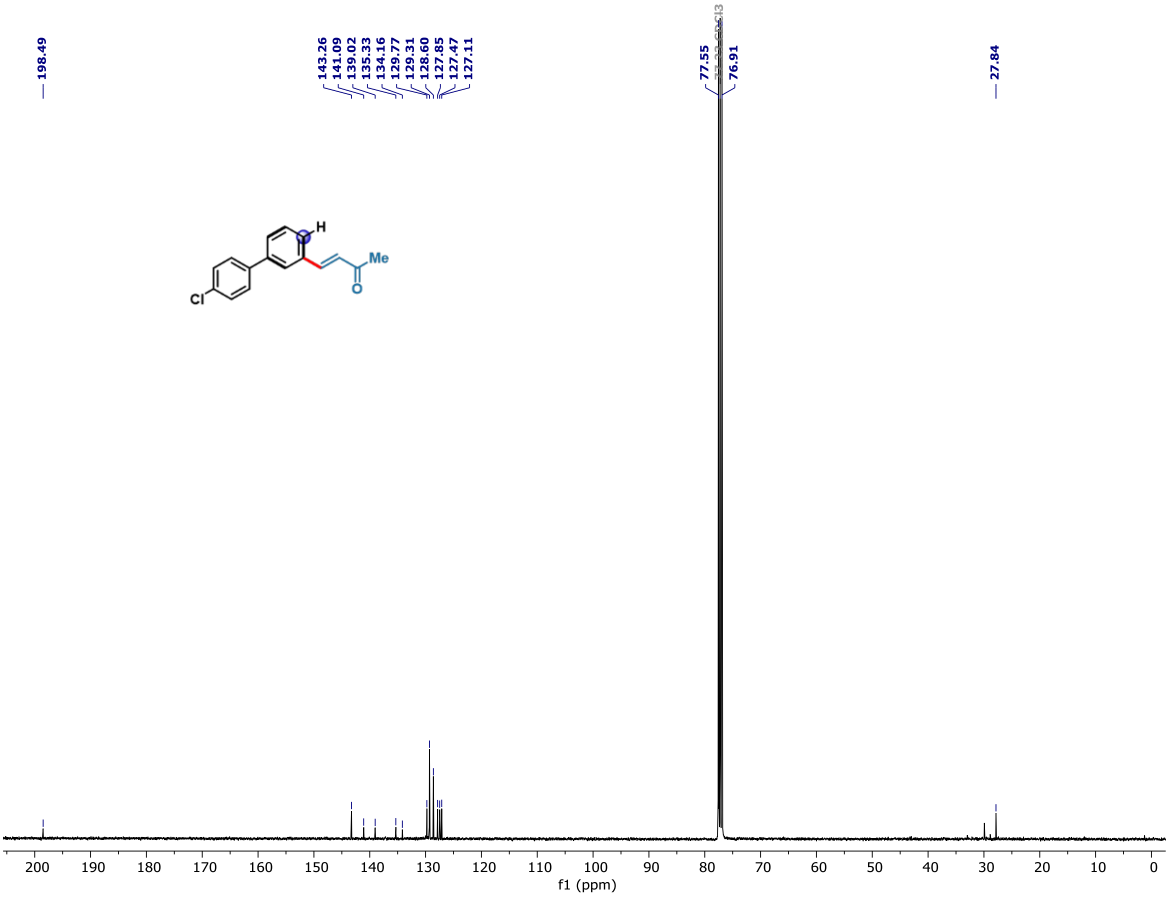

(E)-1,2,3,4,5-pentafluoro-6-styrylbenzene (63)

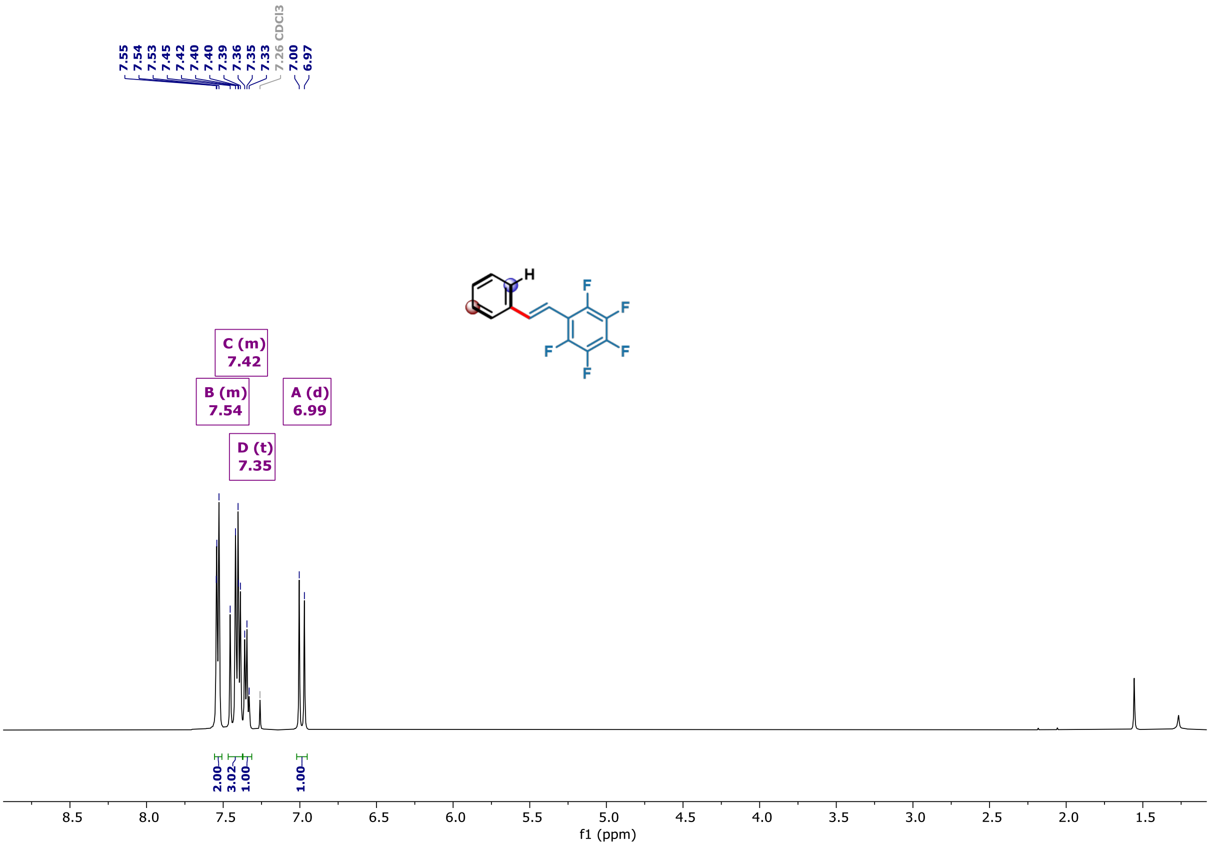

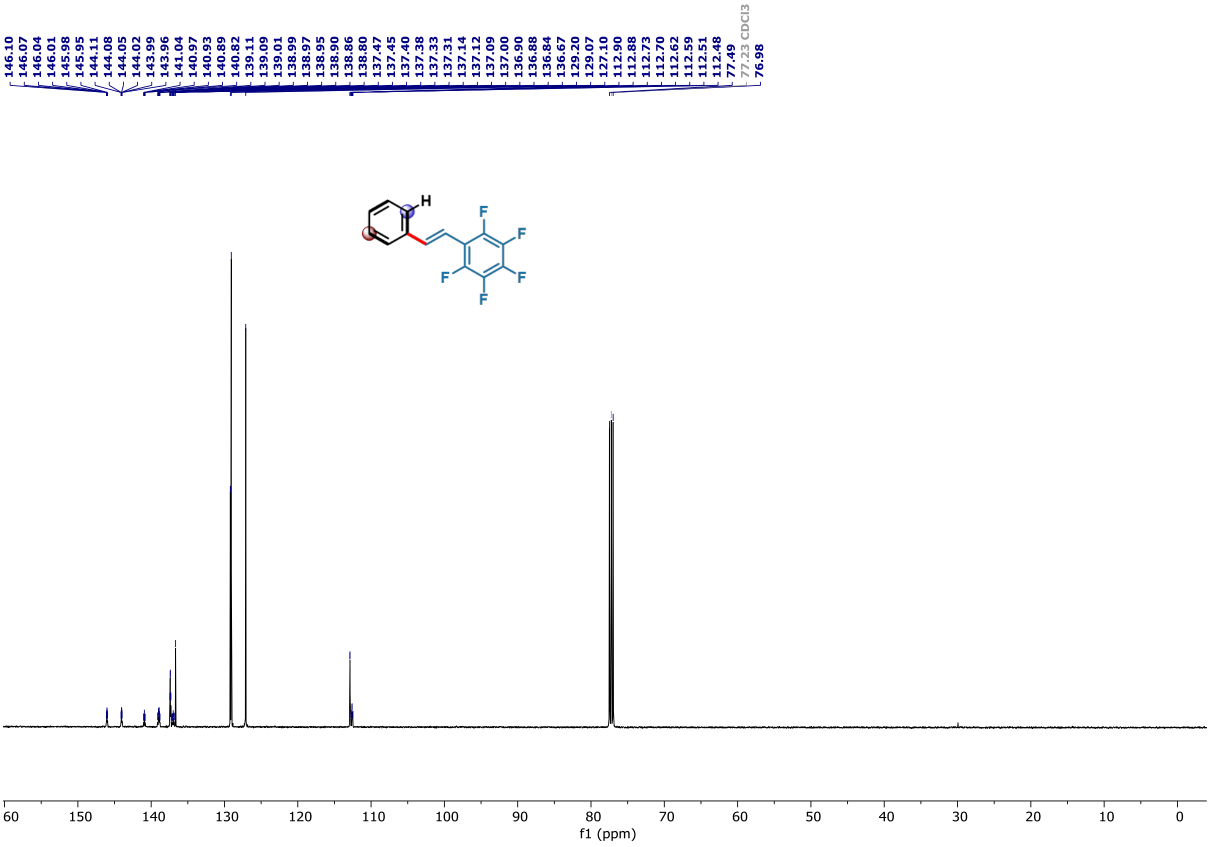

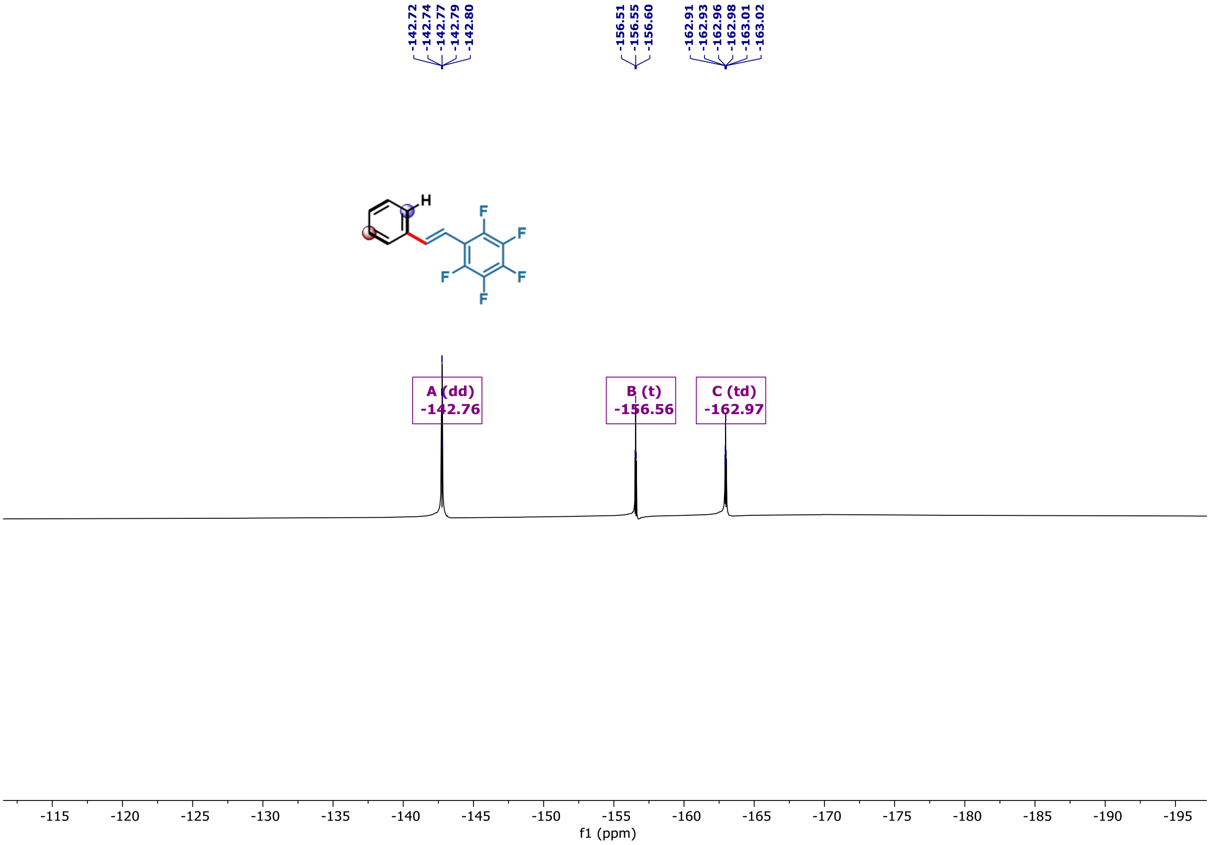
-142.72
-142.74
-142.77
-142.79
-142.80
-156.51
-156.55
-156.60
-162.91
-162.93
-162.96
-162.98
-163.01
-163.02
H
F
F
F
F
F
A (dd)
-142.76
B (t)
-156.56
C (td)
-162.97
-115
-120
-125
-130
-135
-140
-145
-150
-155
-160
-165
-170
-175
-180
-185
-190
-195
f1 (ppm)

4-chloro-1,1'-biphenyl (69)

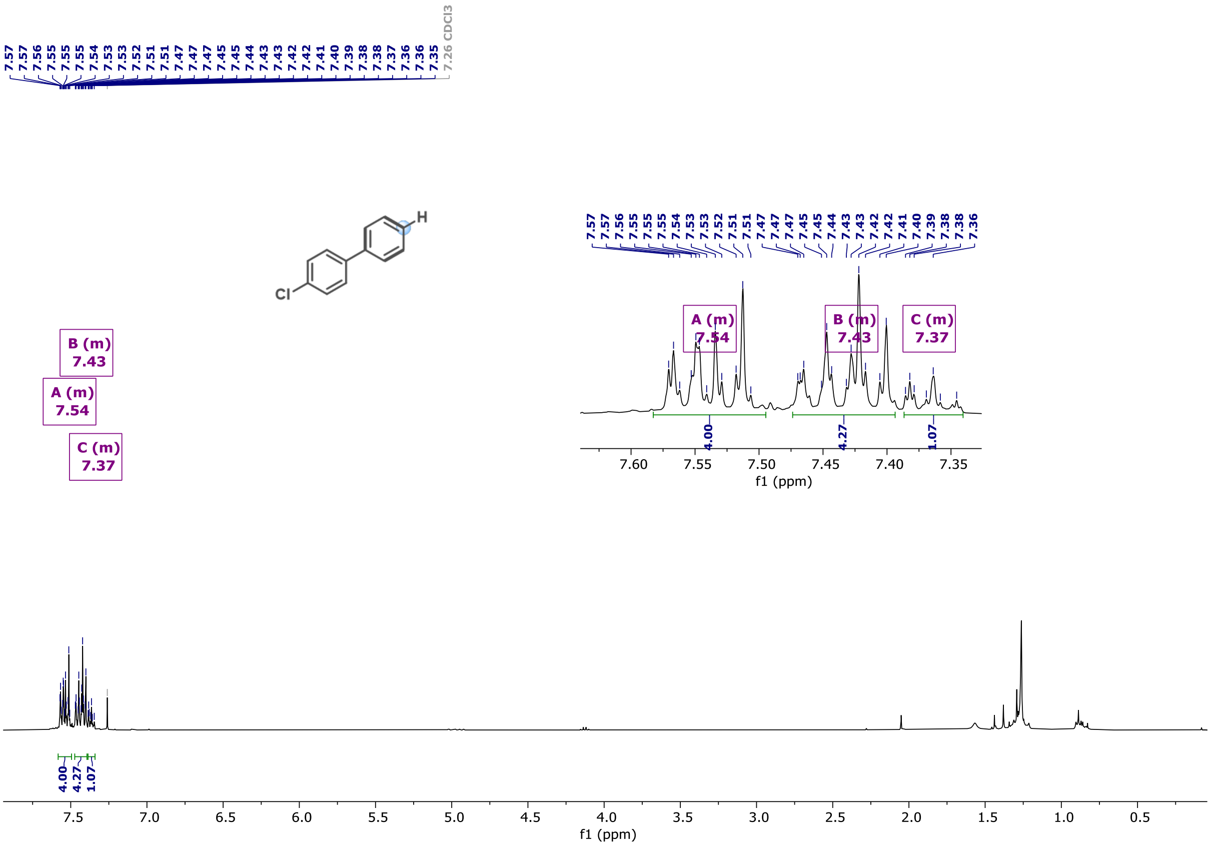

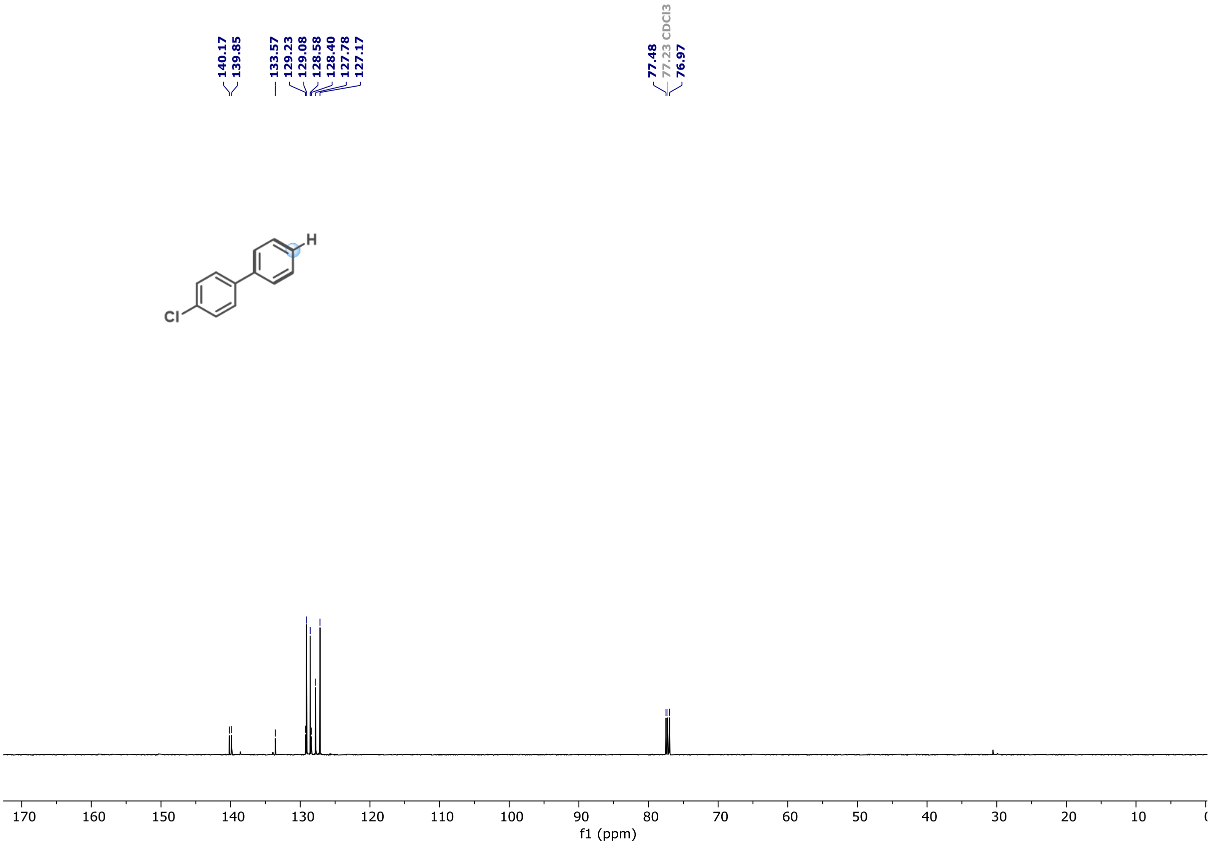

1-(p-tolyl)naphthalene (22')

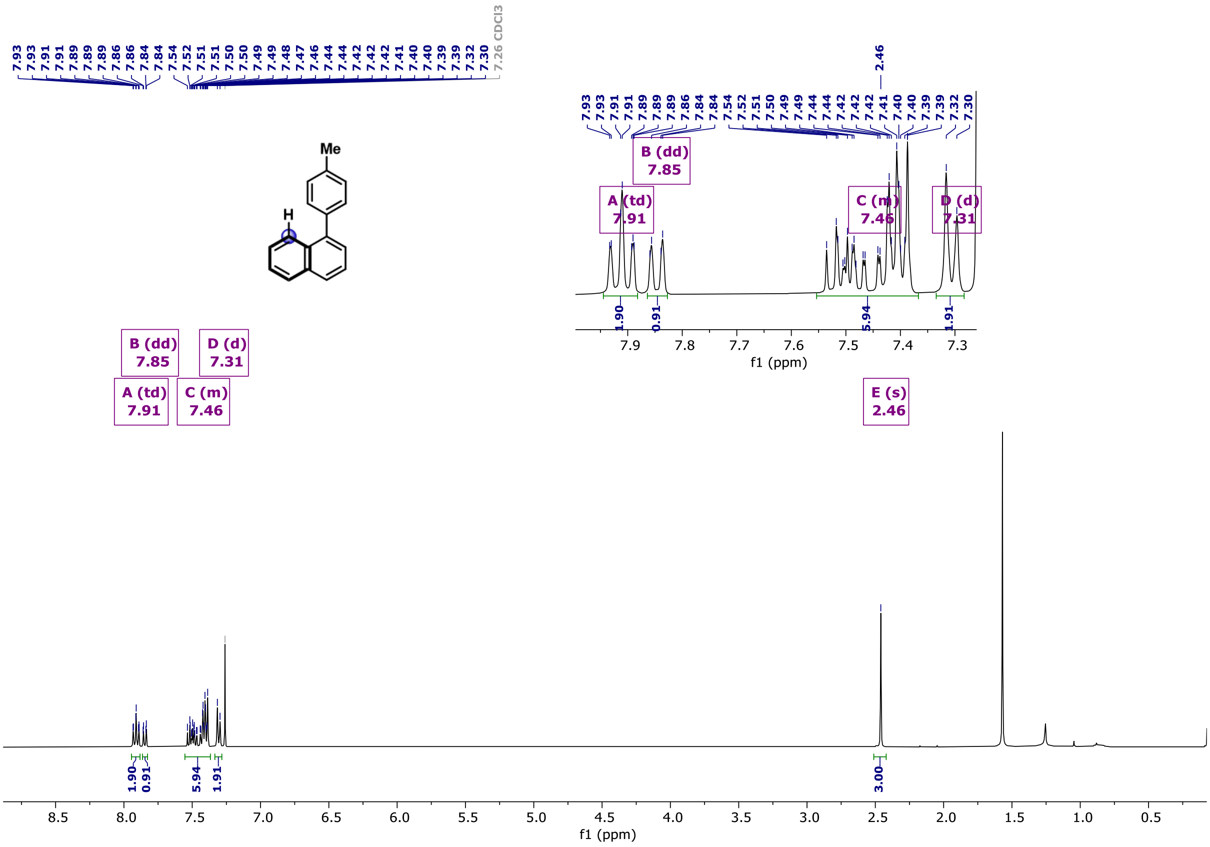

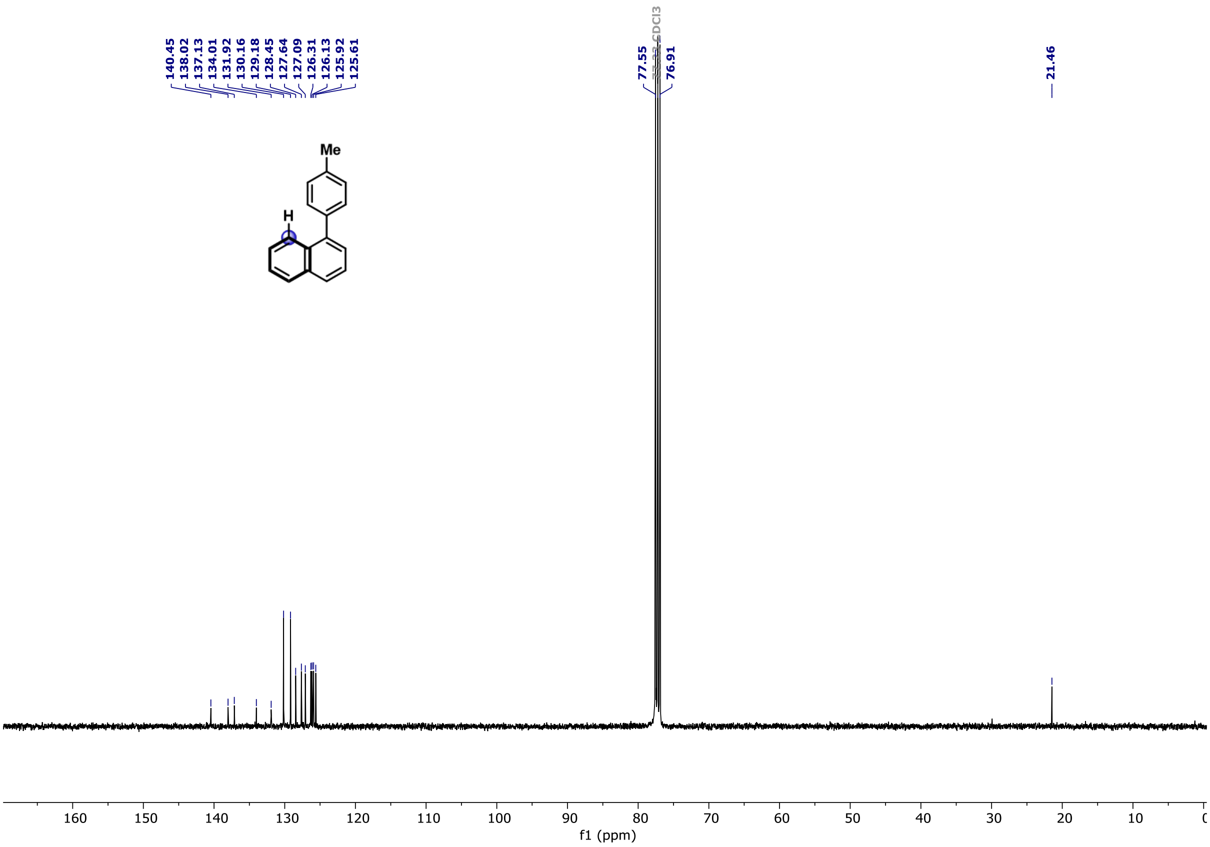

(E)-2-(3-ethoxy-3-oxoprop-1-en-1-yl)cyclopent-2-en-1-yl cyclopentanecarboxylate (71)

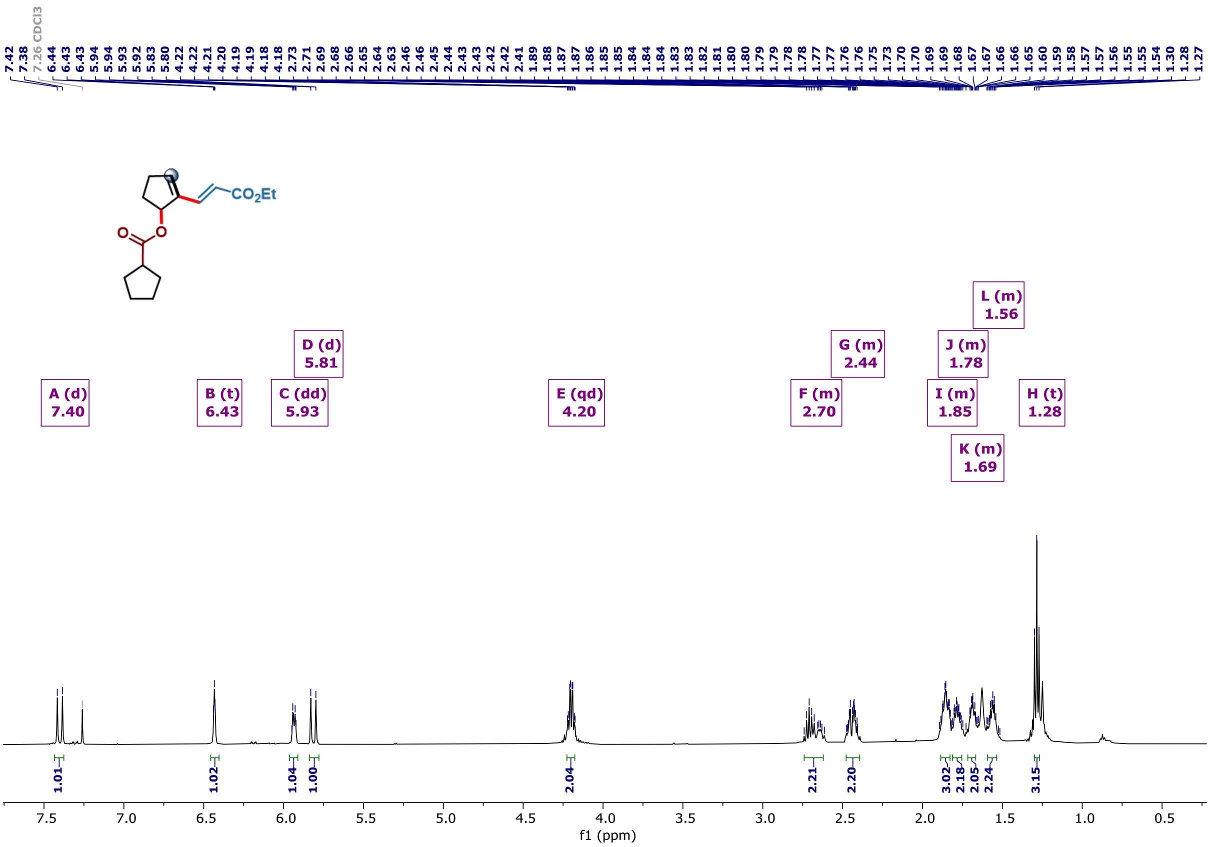

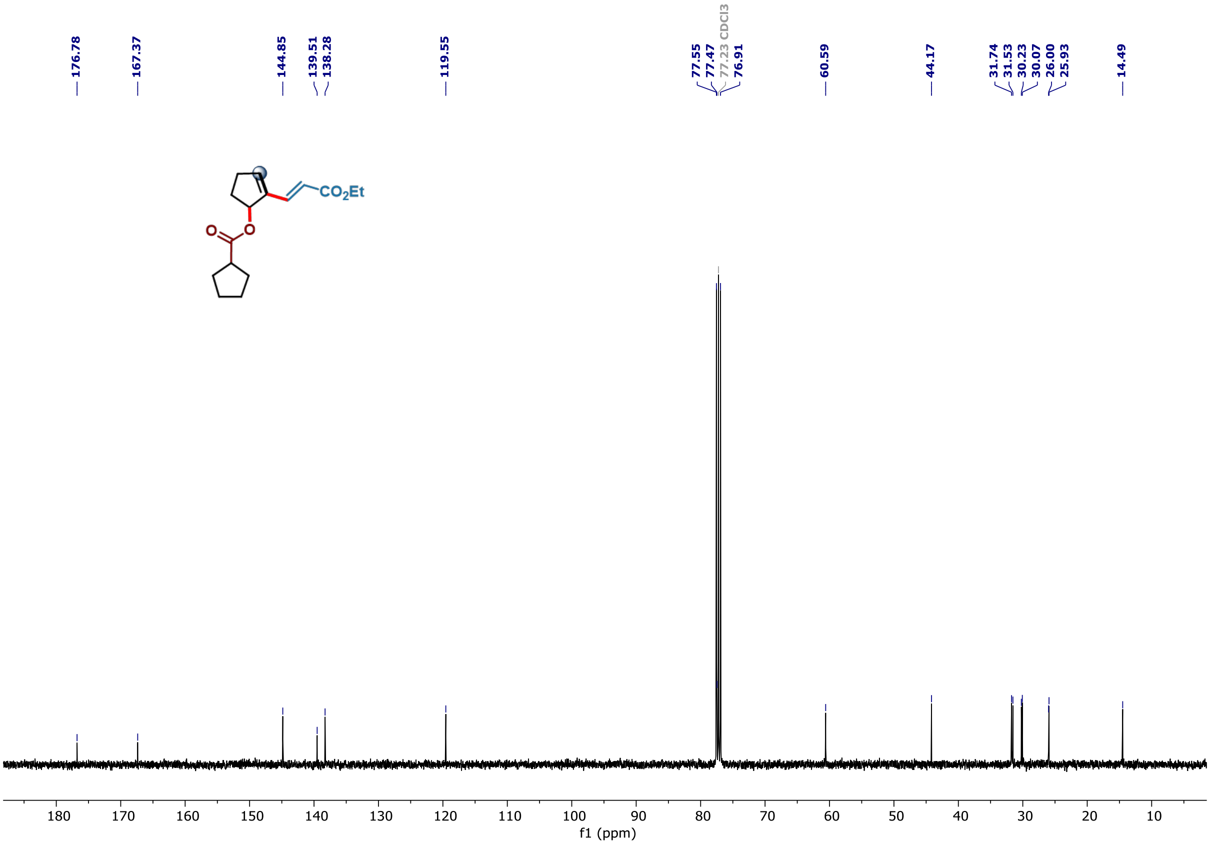

(E)-2-(3-butoxy-3-oxoprop-1-en-1-yl)cyclopent-2-en-1-yl cyclopentanecarboxylate (72)

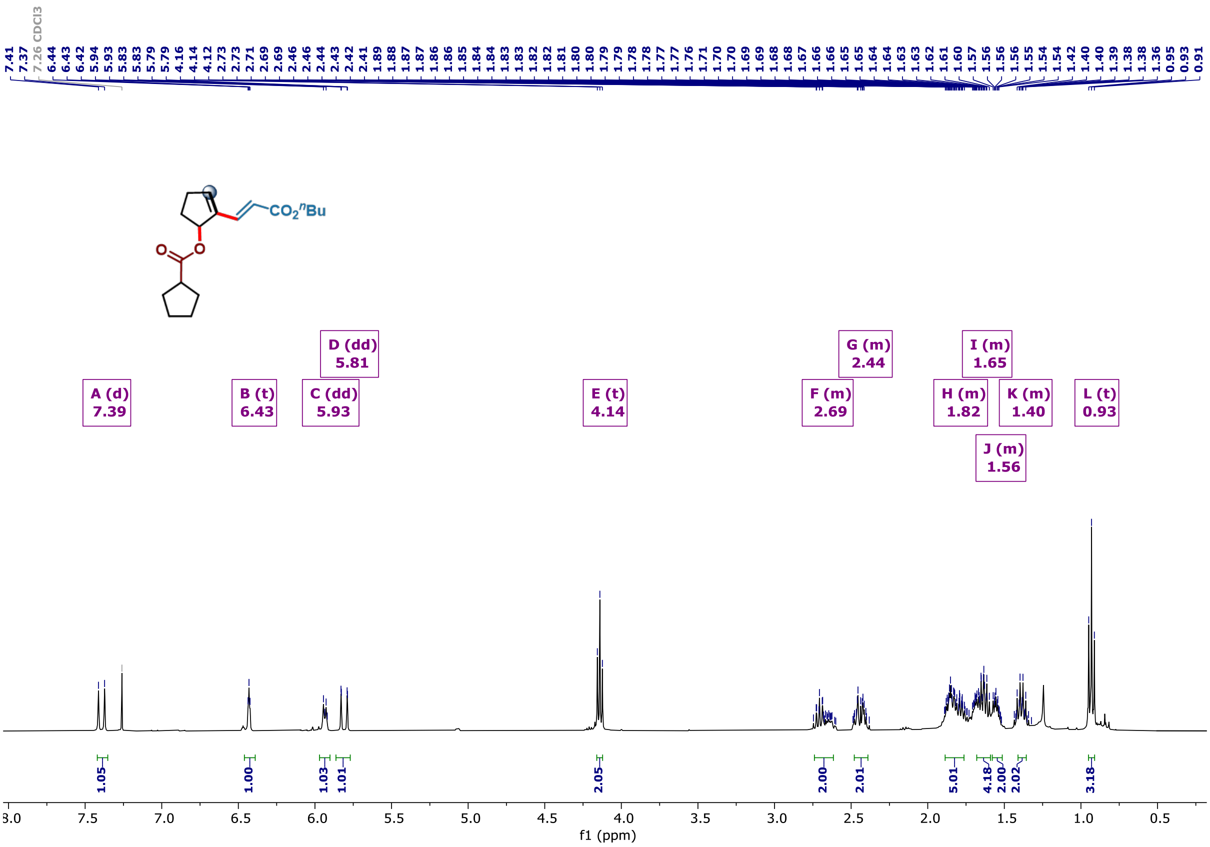

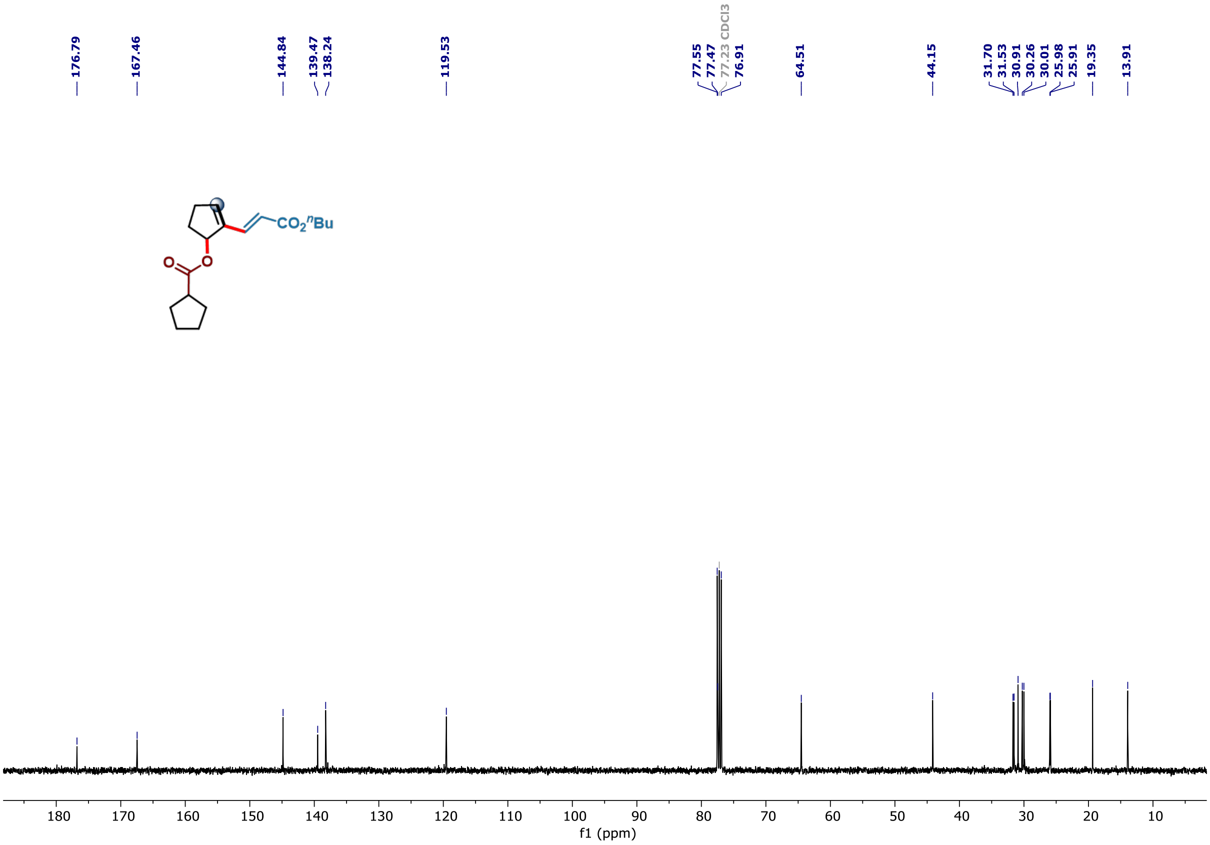

(E)-2-(3-(cyclohexyloxy)-3-oxoprop-1-en-1-yl)cyclopent-2-en-1-yl cyclopentanecarboxylate (73)

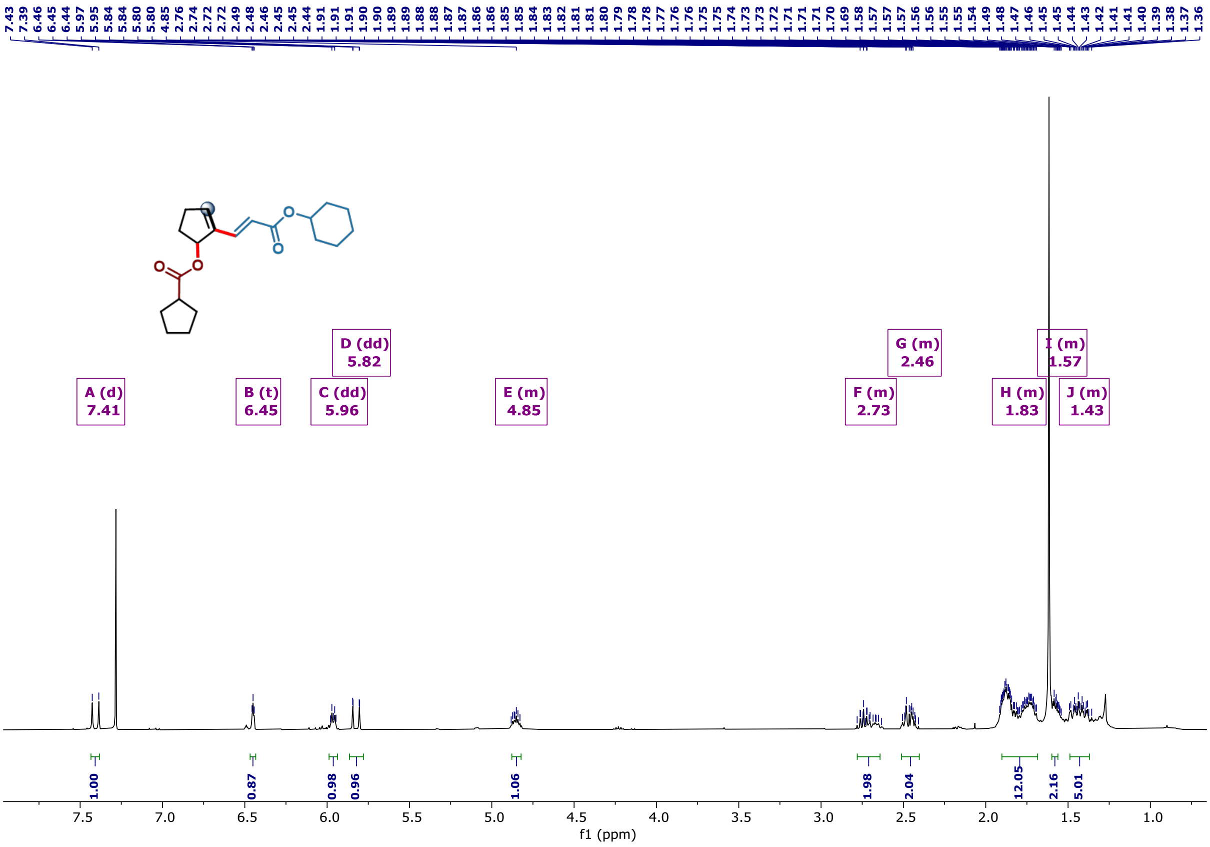

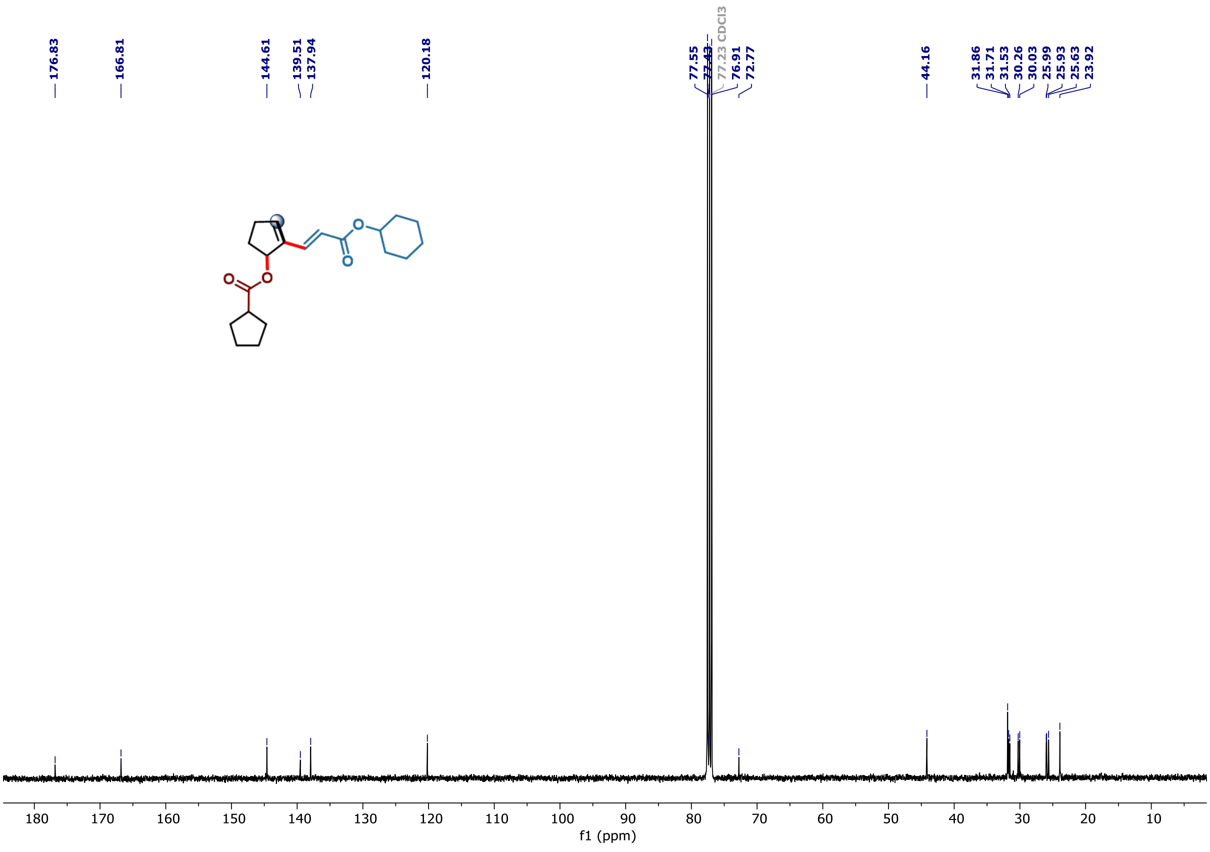

(E)-2-(3-methoxy-3-oxoprop-1-en-1-yl)cyclopent-2-en-1-yl cyclopentanecarboxylate (74)

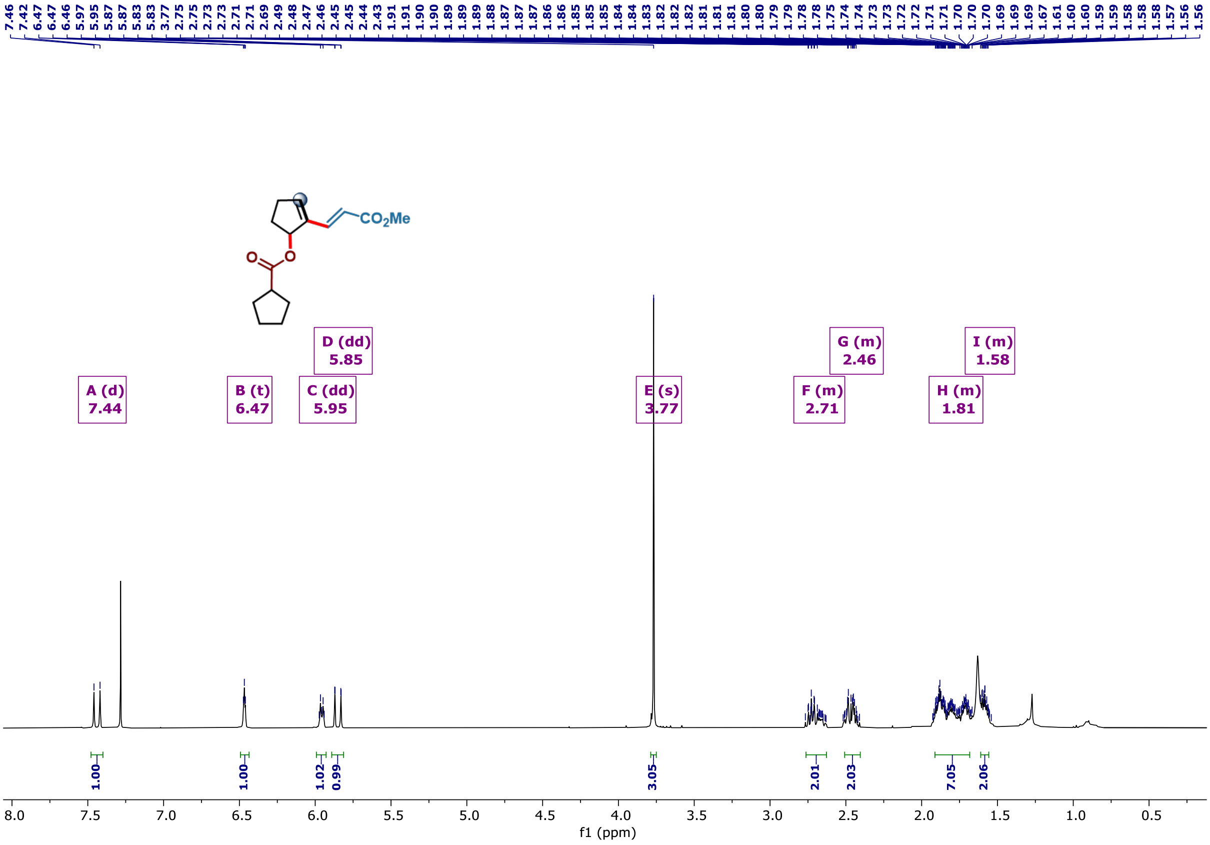

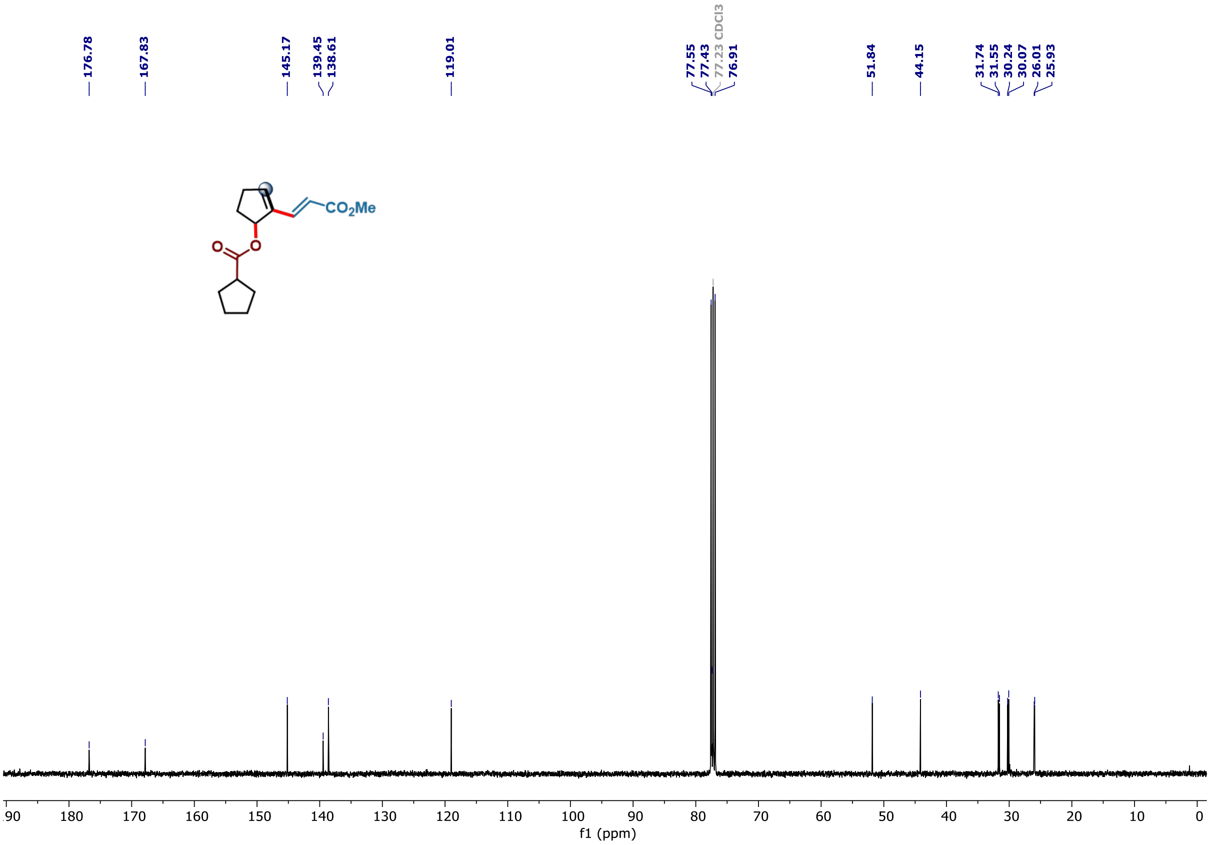

(E)-2-(3-(benzyloxy)-3-oxoprop-1-en-1-yl)cyclopent-2-en-1-yl cyclopentanecarboxylate (75)

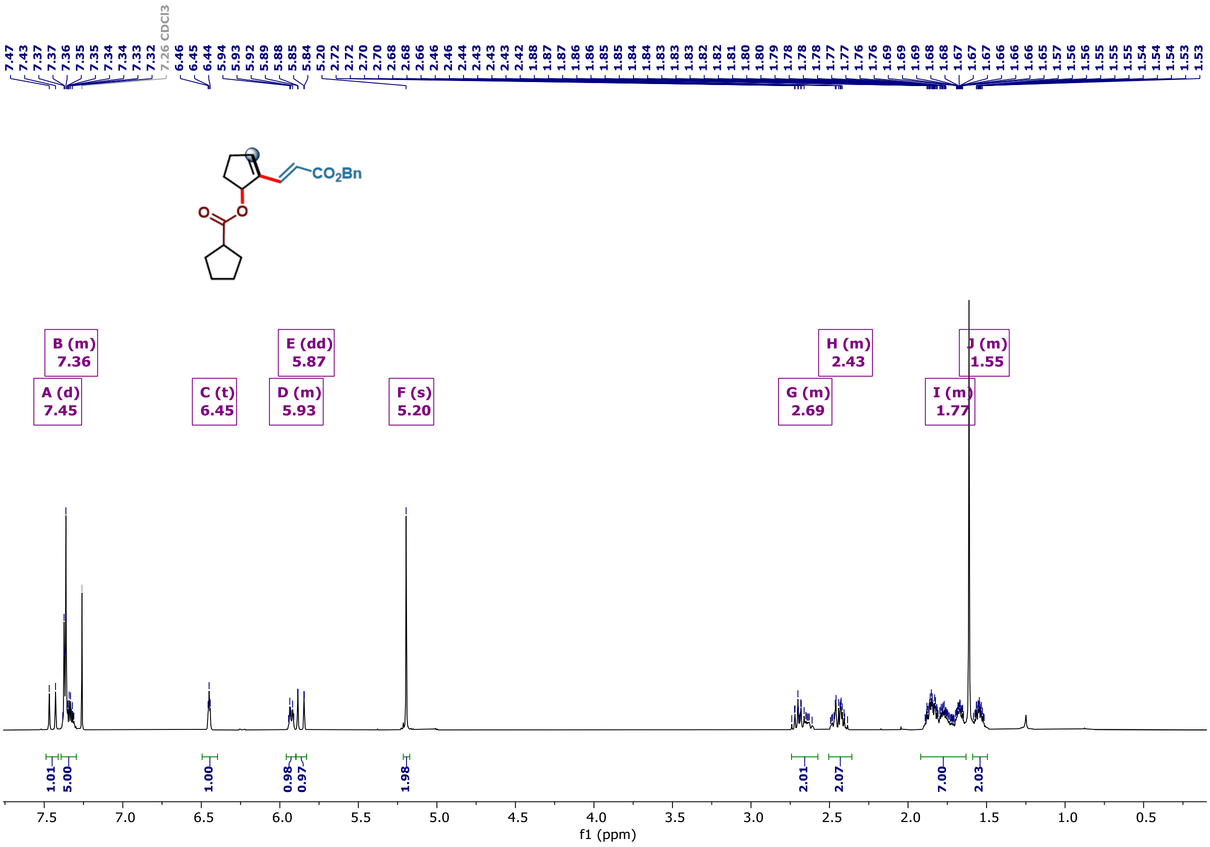

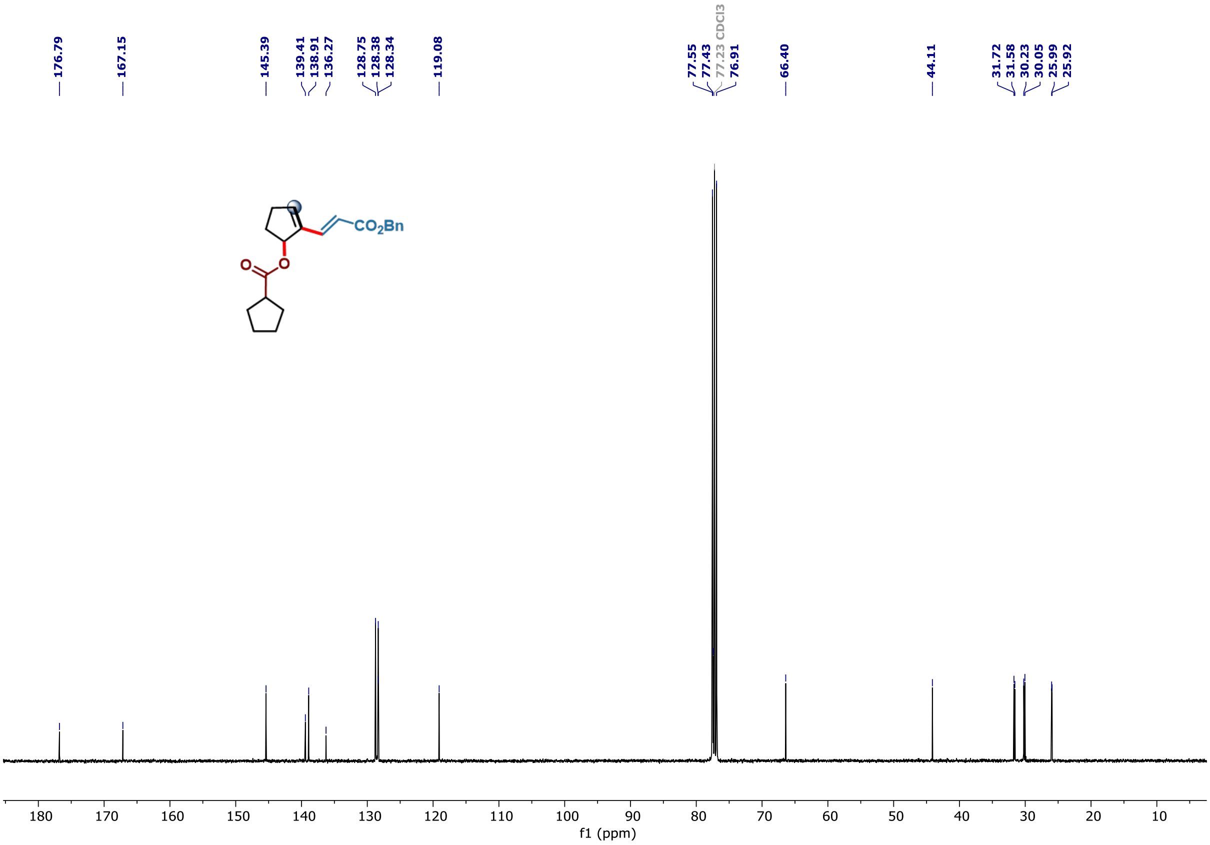

(E)-2-(3-oxobut-1-en-1-yl)cyclopent-2-en-1-yl cyclopentanecarboxylate (76)

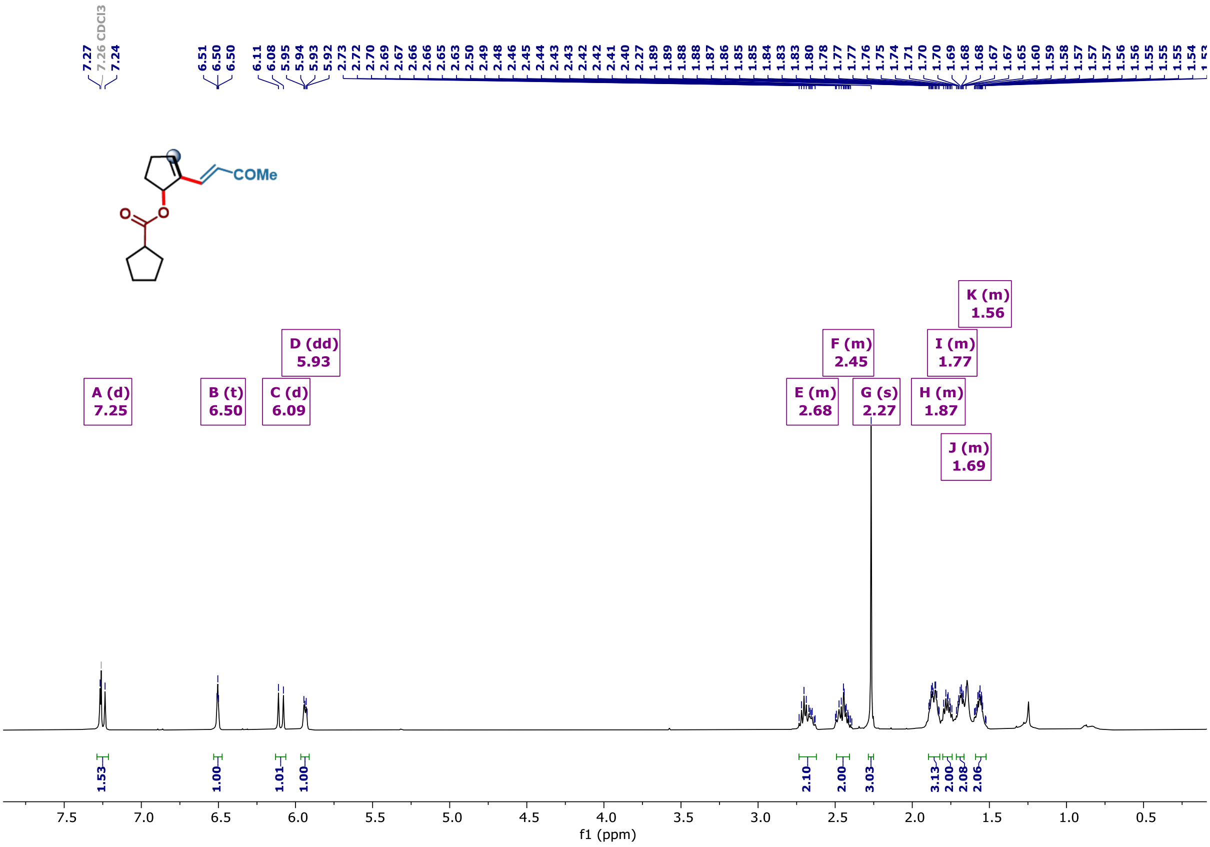

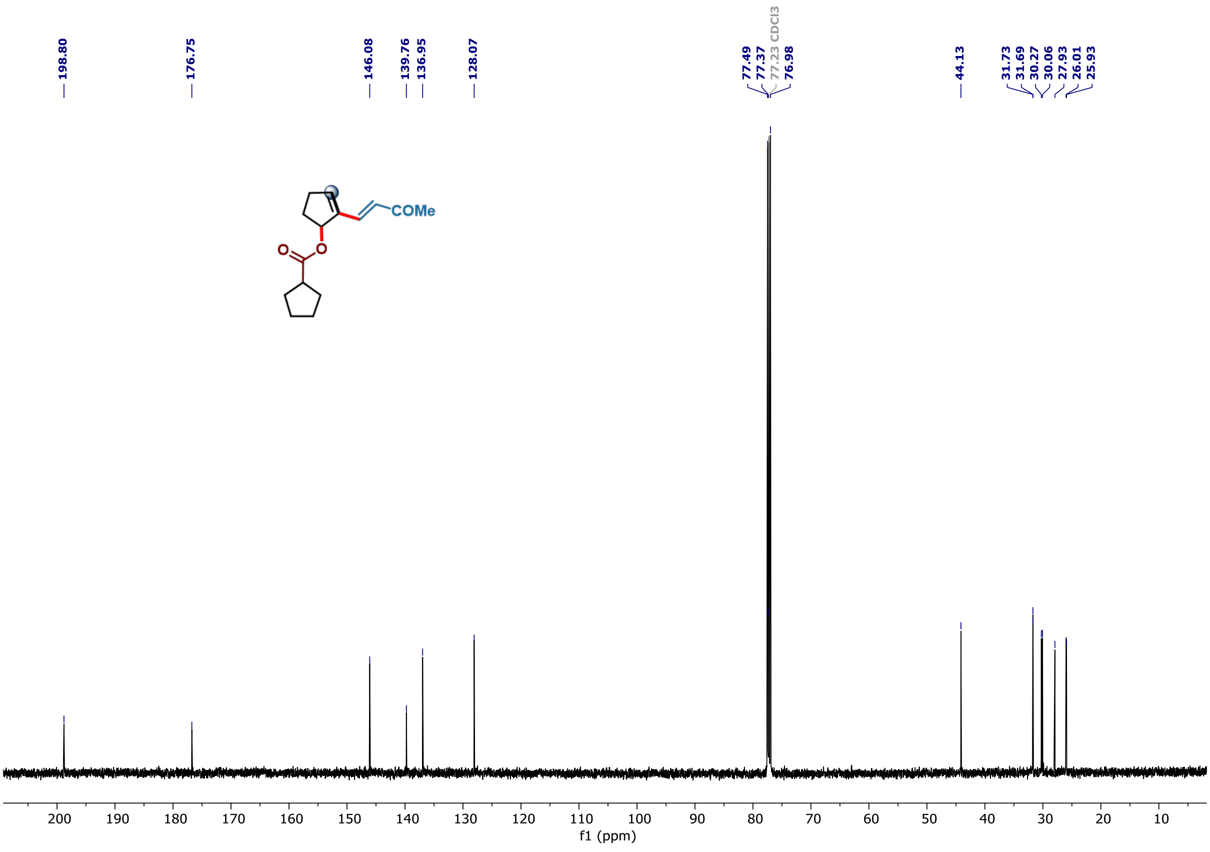

(E)-2-(3-ethoxy-3-oxoprop-1-en-1-yl)cyclopent-2-en-1-yl cyclobutanecarboxylate (77) & (E)-2-(3-ethoxy-3-oxoprop-1-en-1-yl)cyclopent-2-en-1-yl cyclopentanecarboxylate (71) (inseparable mixture of products)

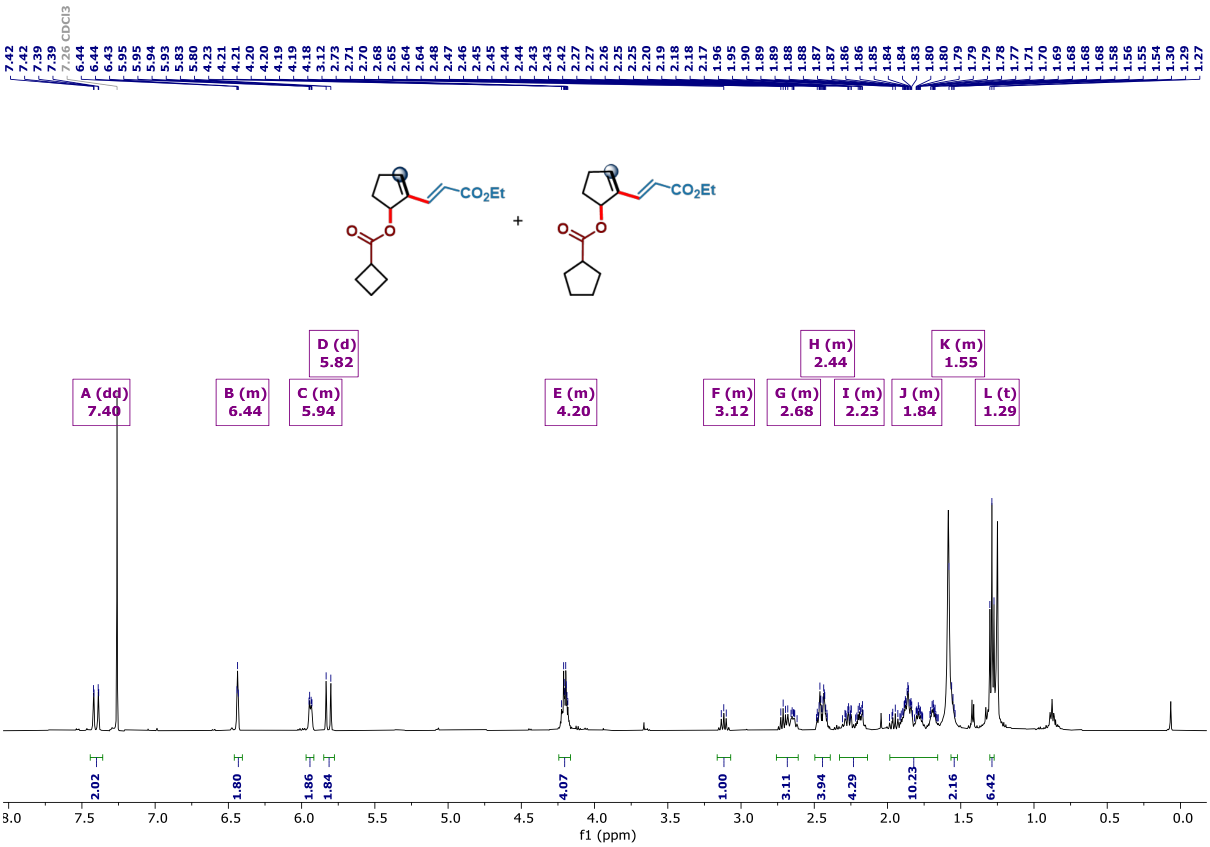

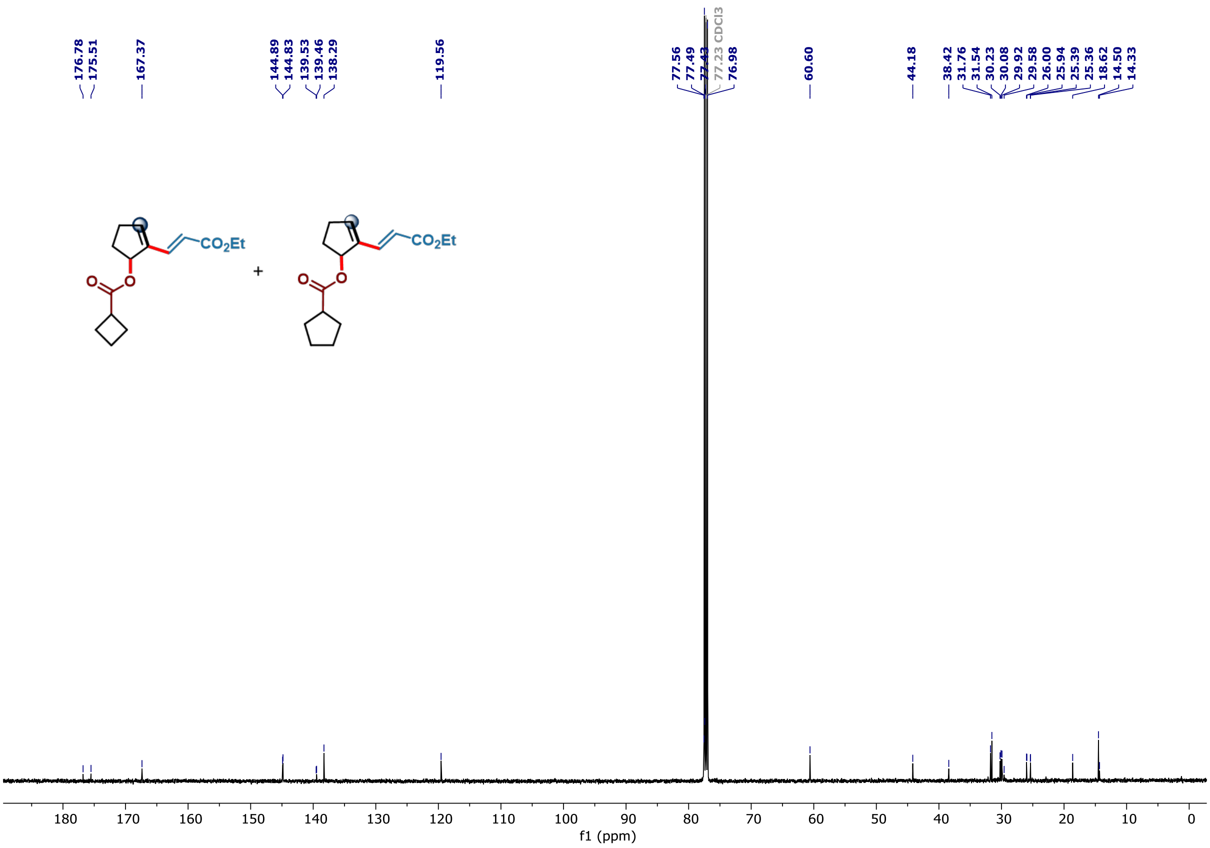

2-((E)-3-ethoxy-3-oxoprop-1-en-1-yl)cyclopent-2-en-1-yl 2-methylcyclohexane-1-carboxylate (78)

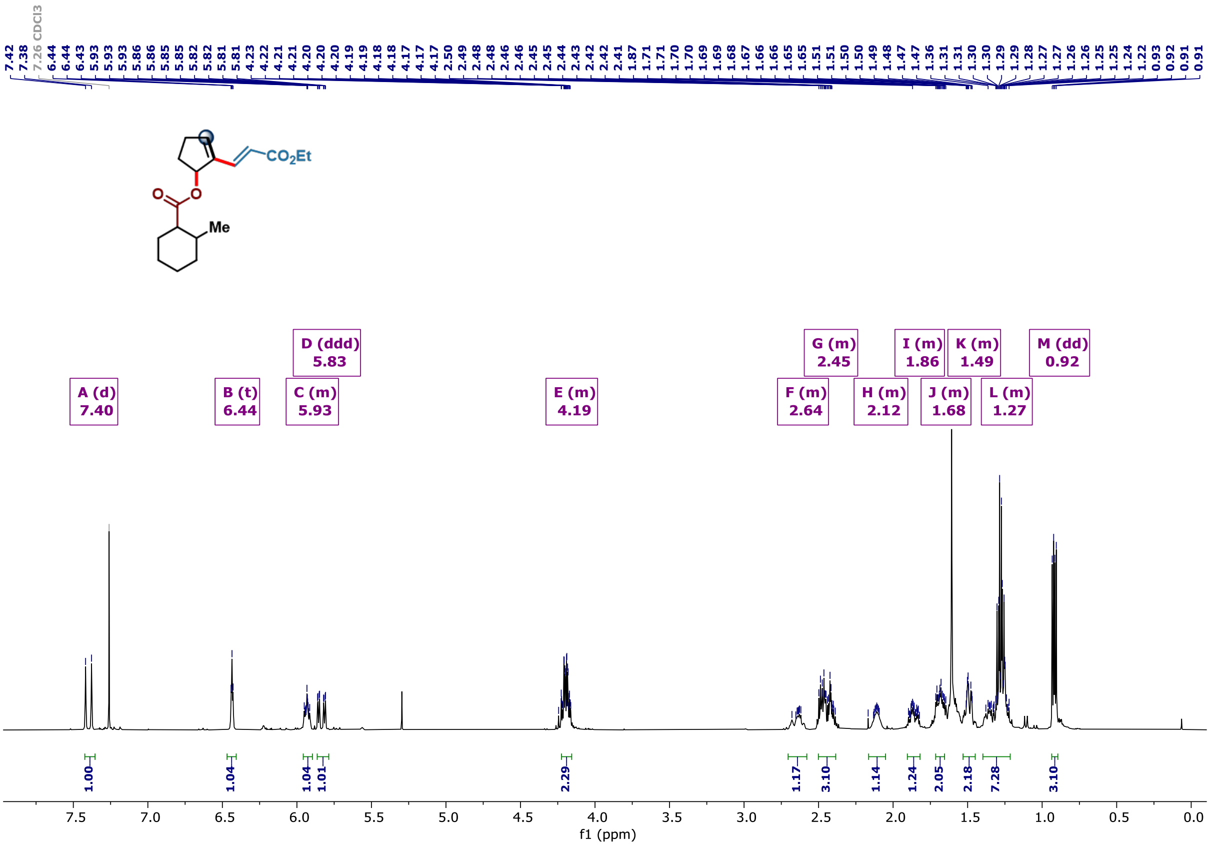

(*Peak at 5.30 corresponds to DCM impurity)

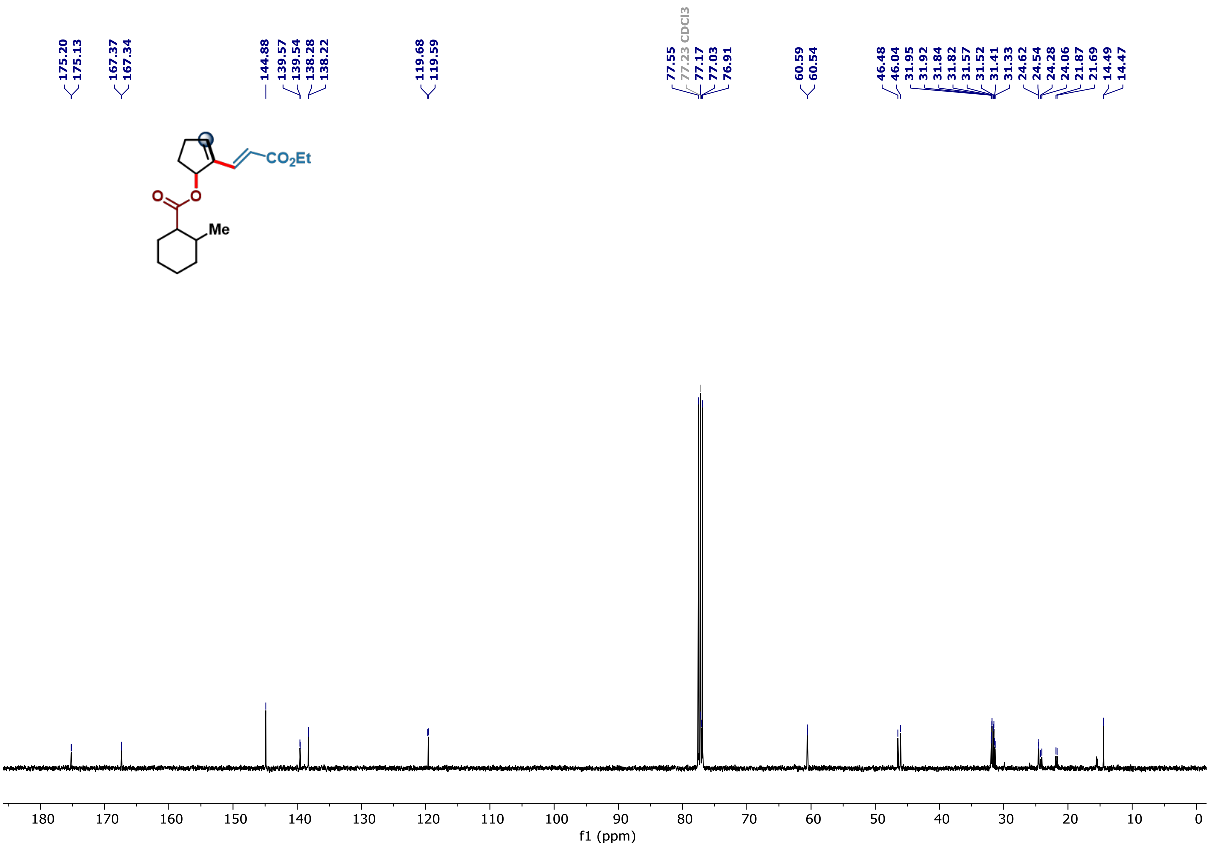

2-((E)-3-ethoxy-3-oxoprop-1-en-1-yl)cyclopent-2-en-1-yl 5-isopropyl-2-methylcyclohexane-1-carboxylate (79)

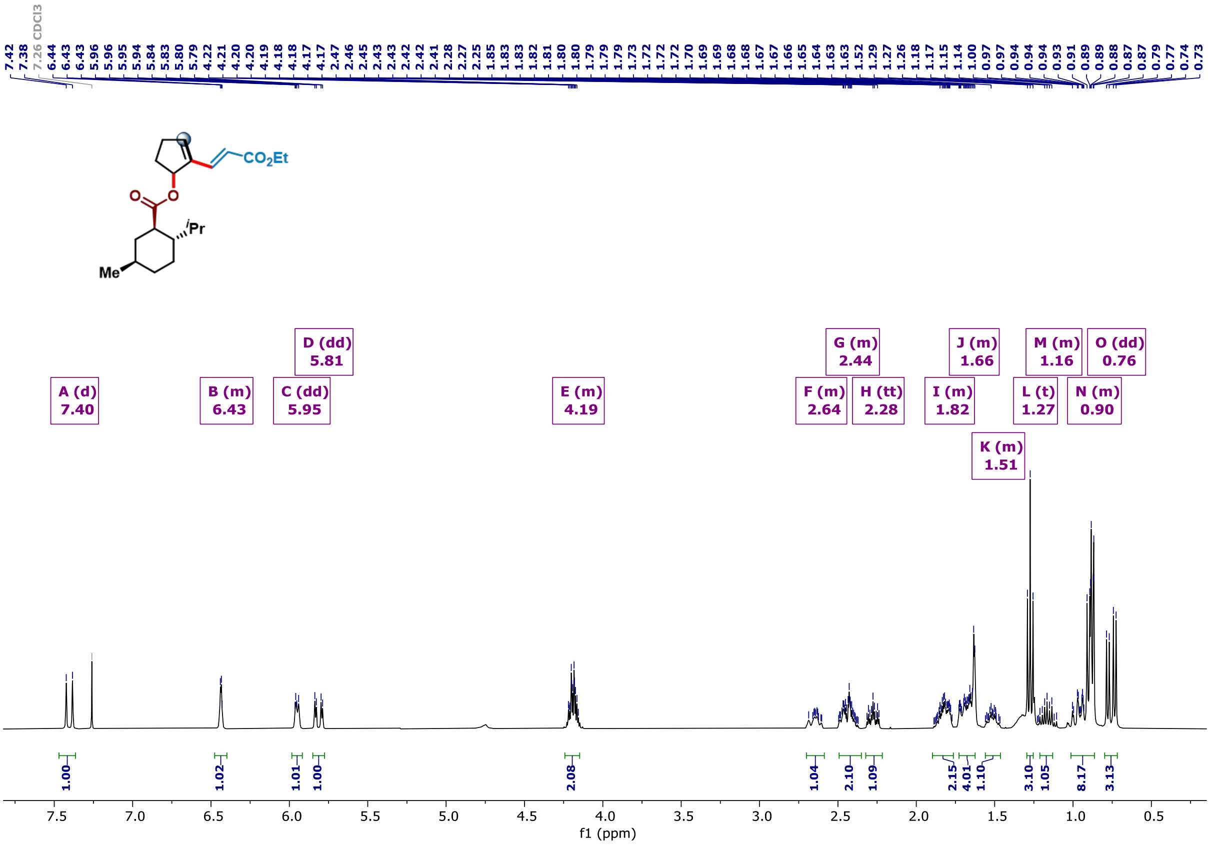

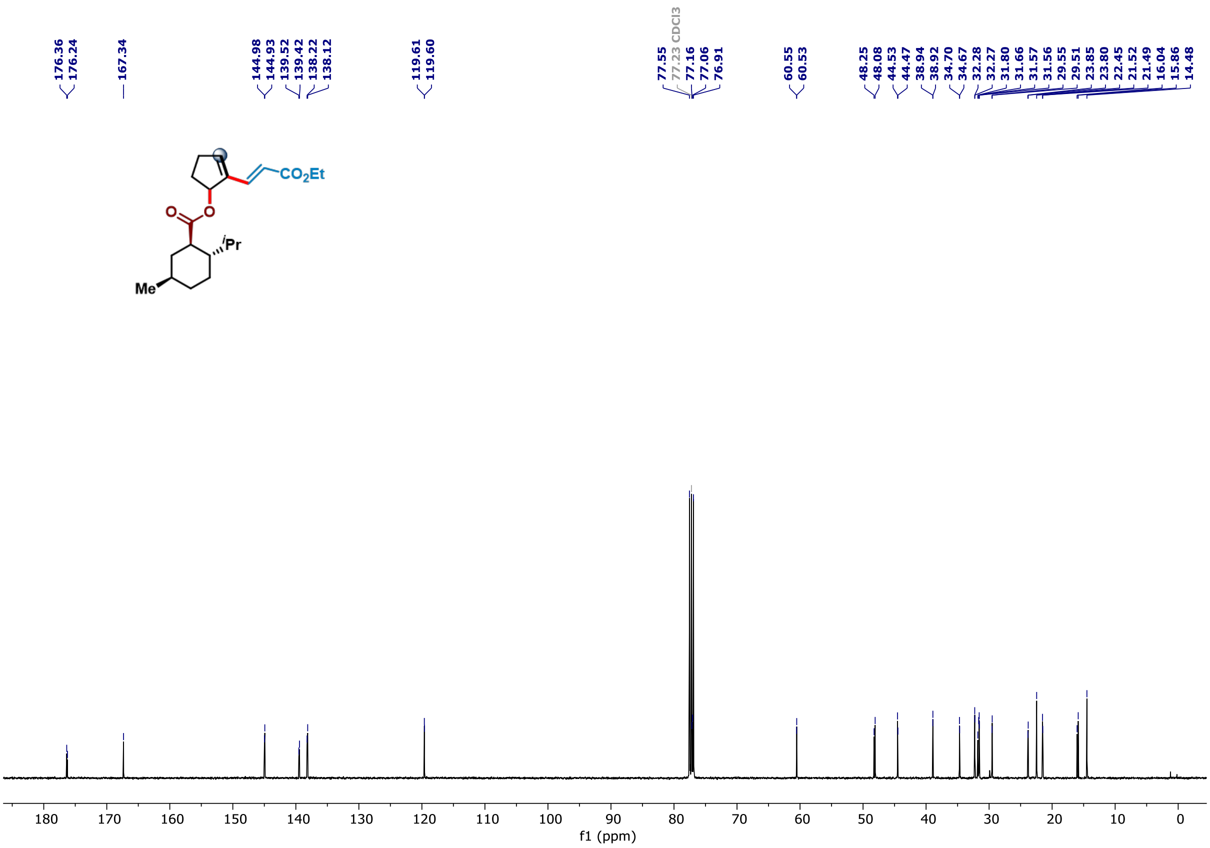

2-((E)-3-oxobut-1-en-1-yl)cyclopent-2-en-1-yl (2S,5R)-2-isopropyl-5-methylcyclohexane-1-carboxylate (80)

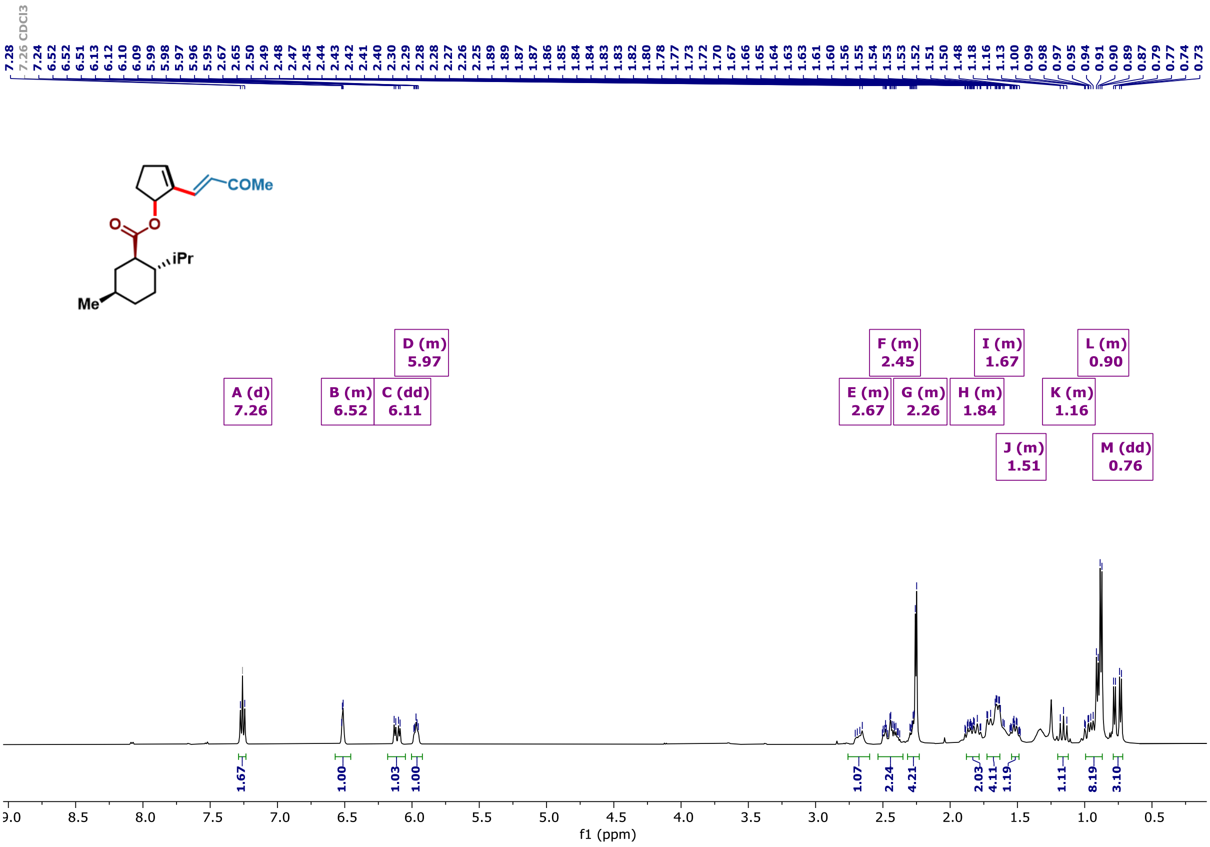

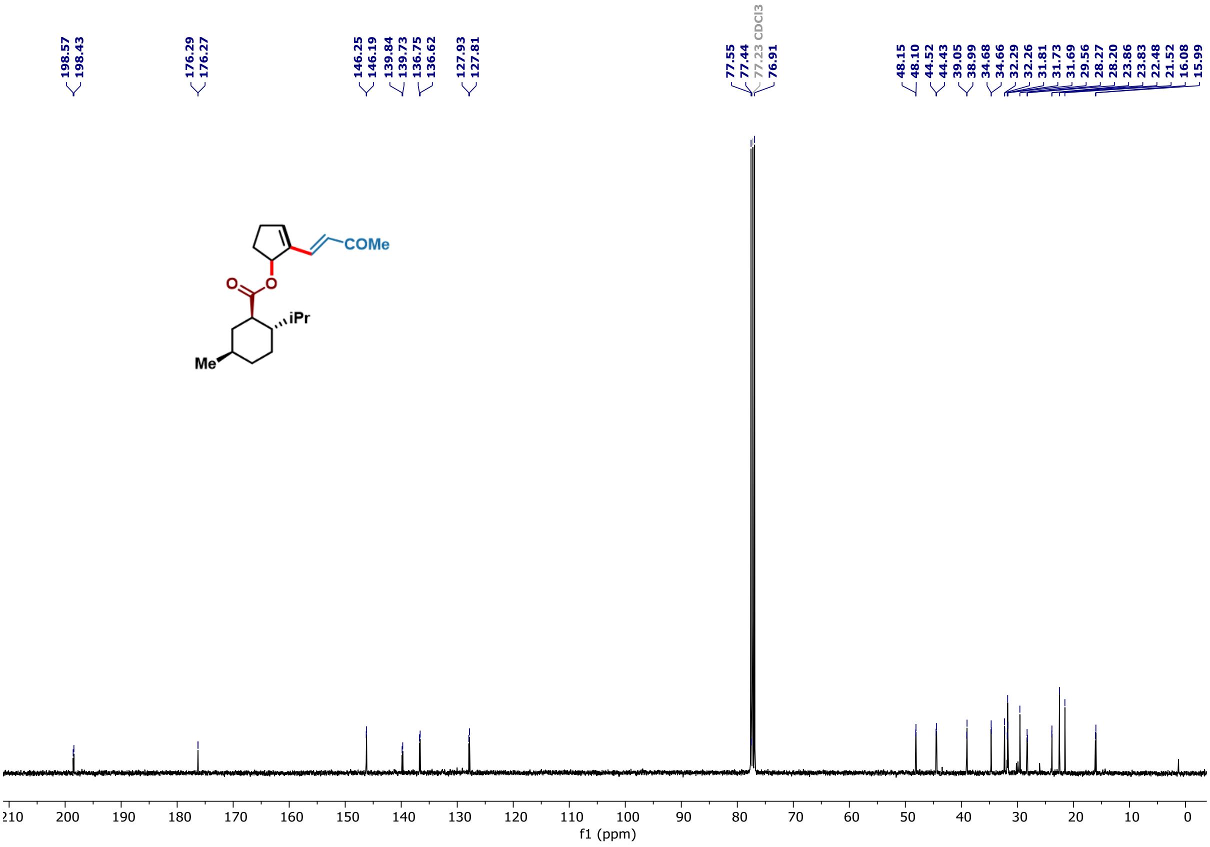

Ethyl (E)-3-(3-((4-methoxyphenoxy)methyl)phenyl)acrylate (81)

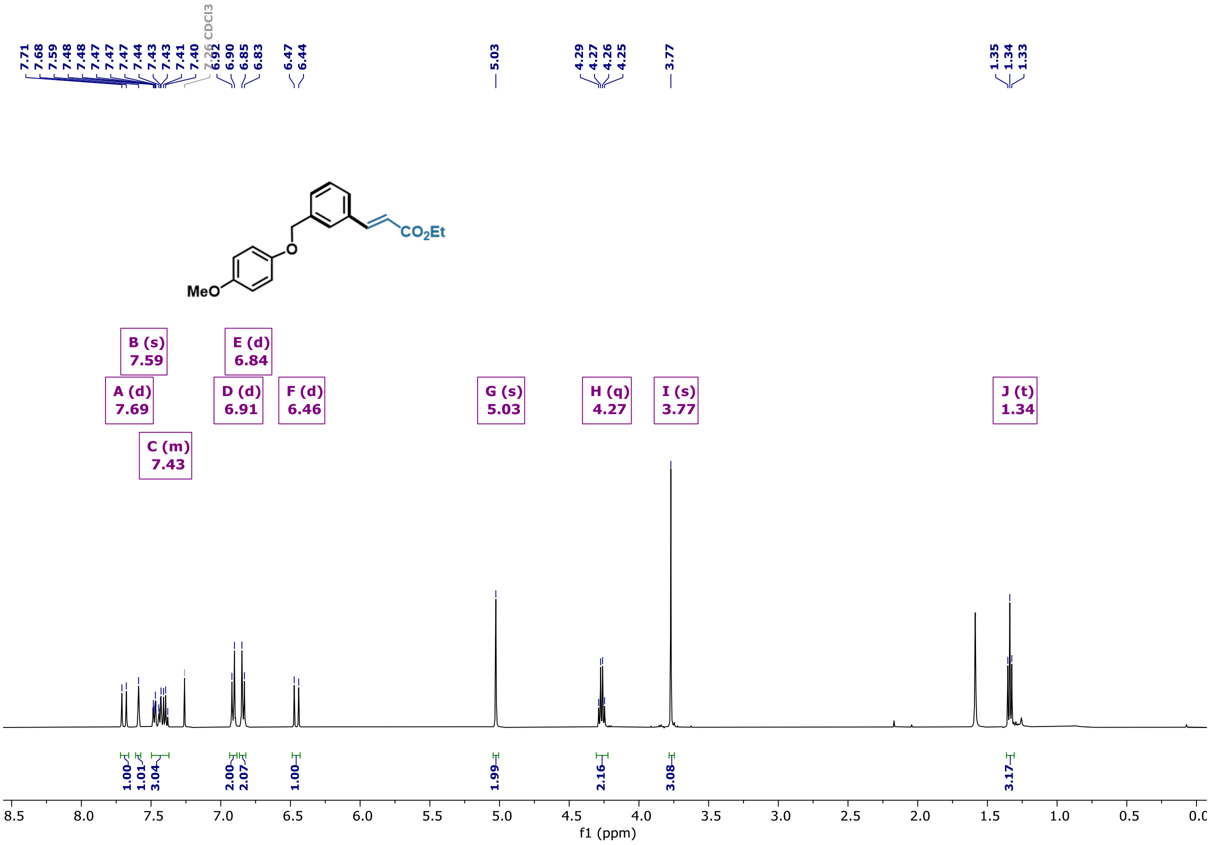

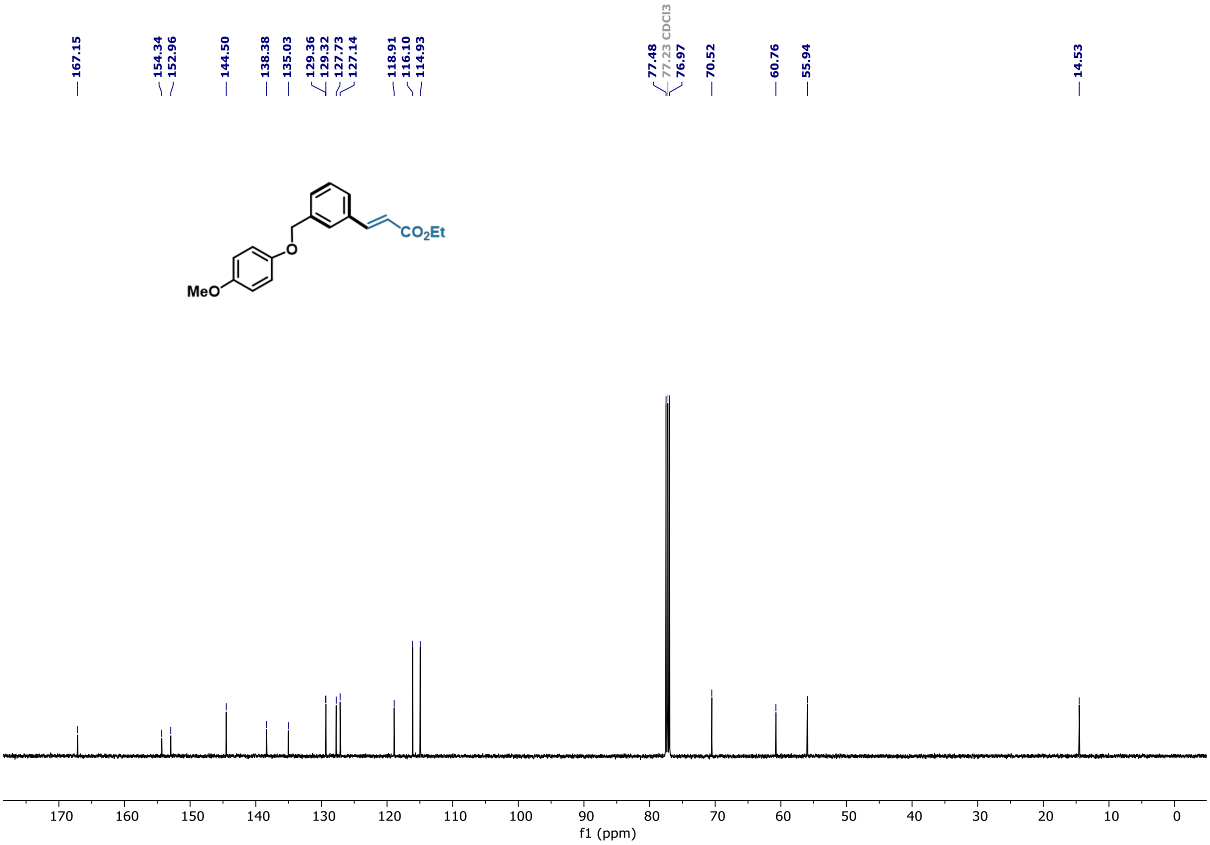

(E)-1-(pyrrolidin-1-yl)-3-(p-tolyl)prop-2-en-1-one (82)

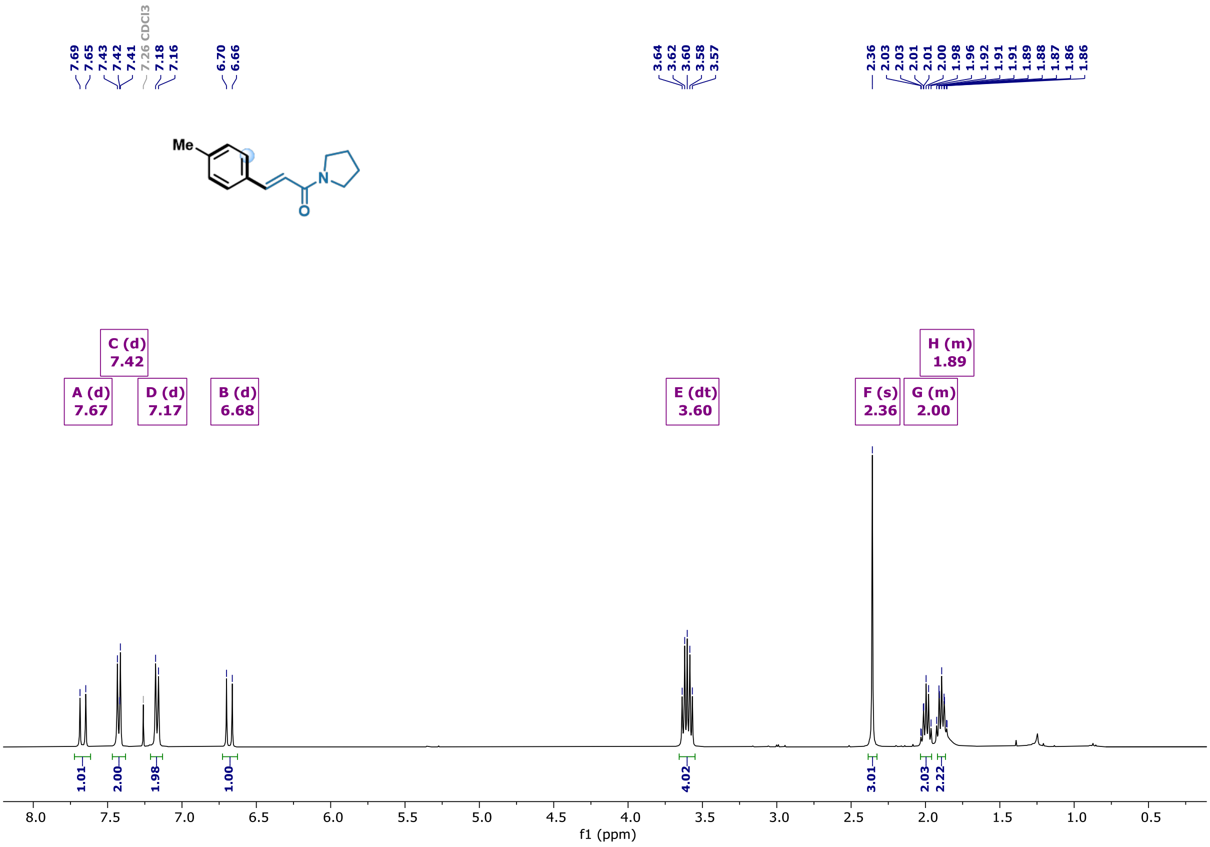

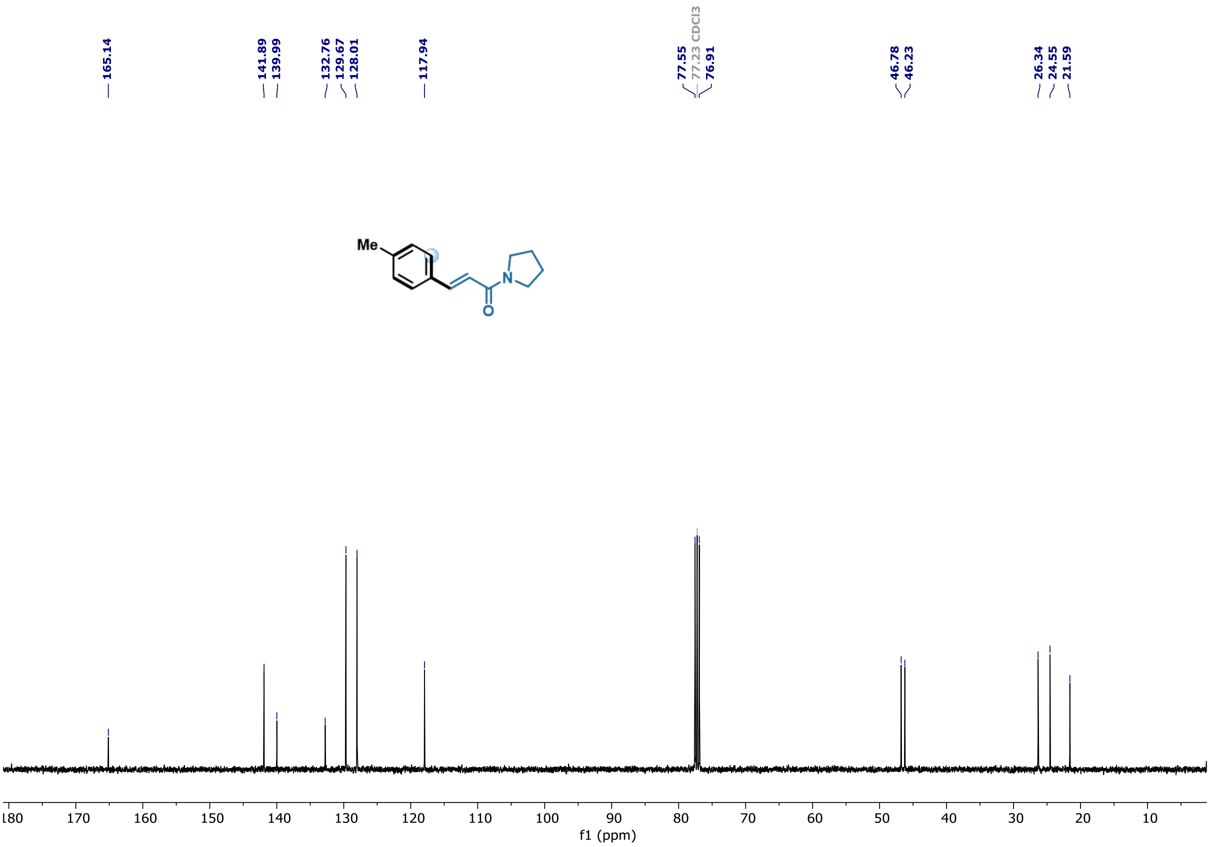

1-(pyrrolidin-1-yl)-3-(p-tolyl)propan-1-one (83)

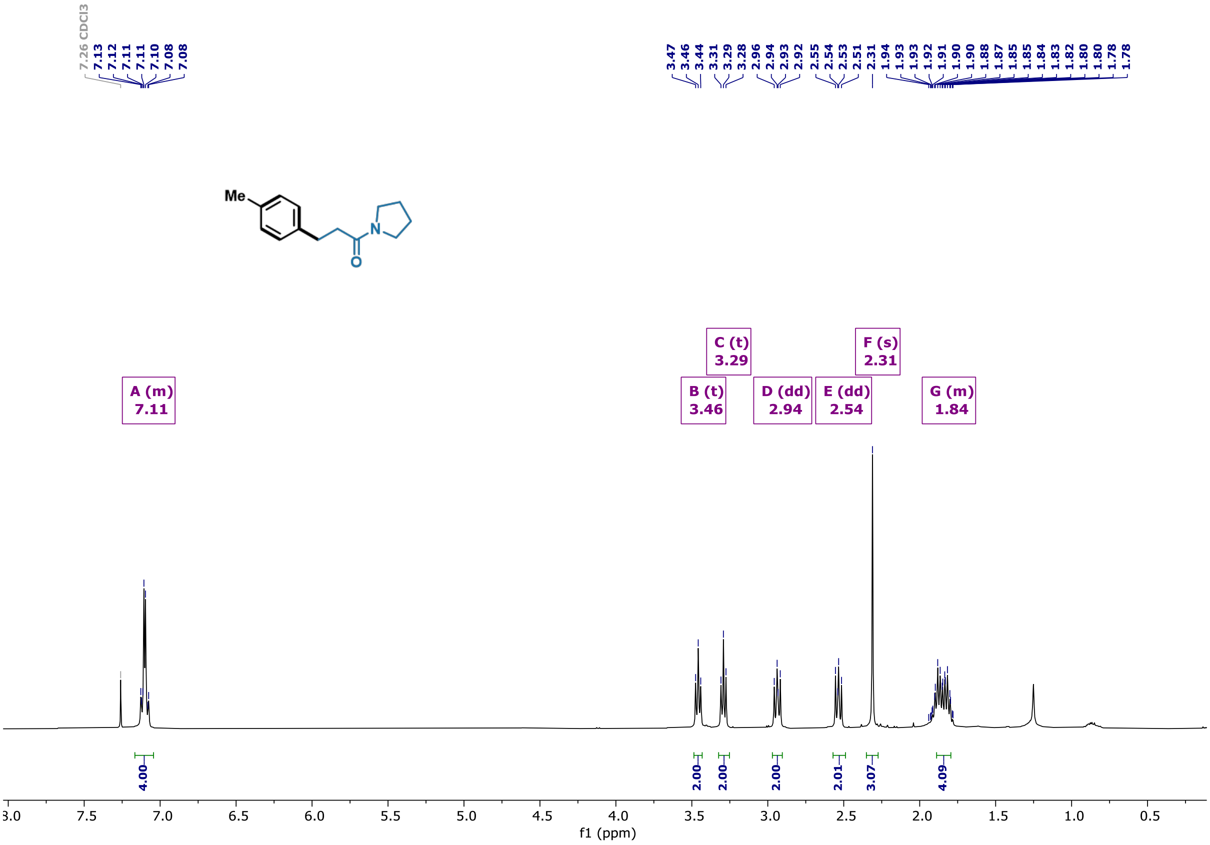

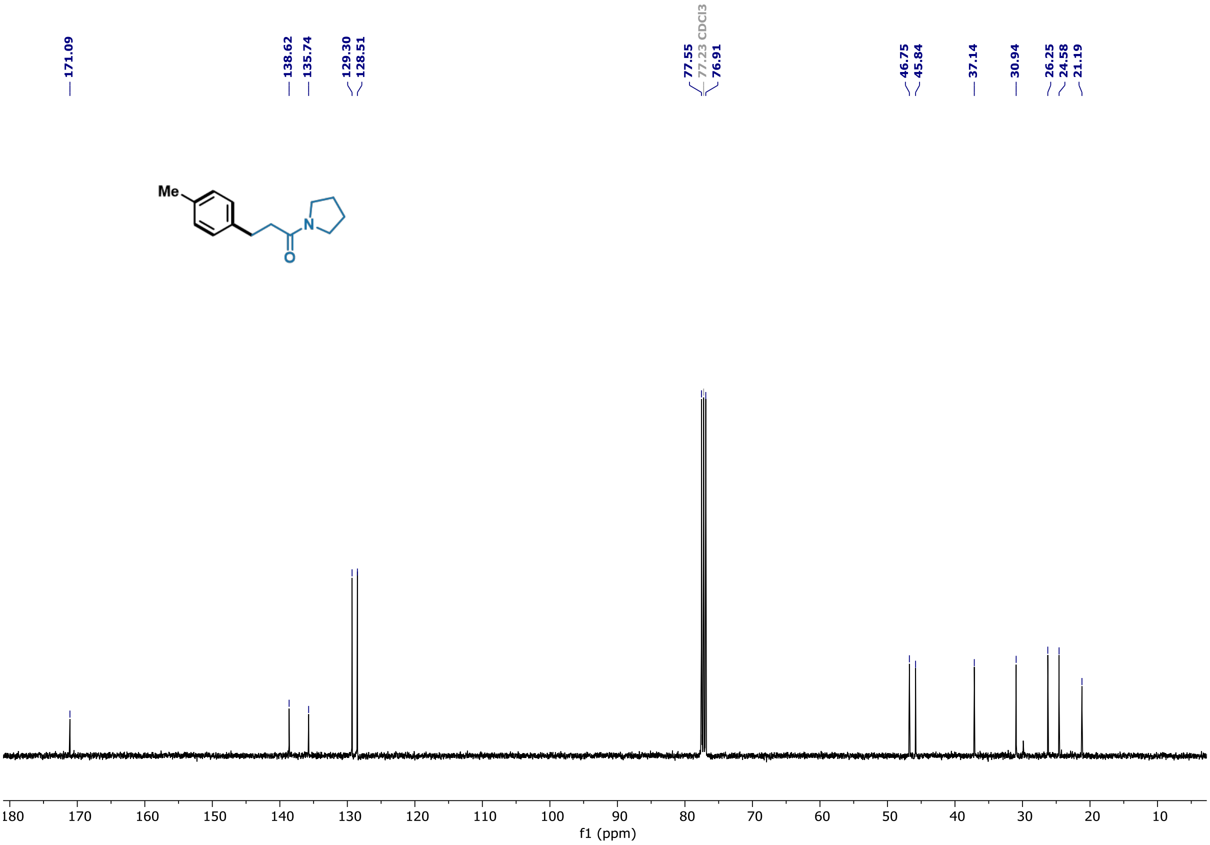

1,4,5,6-tetrahydro-[1,1'-biphenyl]-2-carboxylic acid (84)

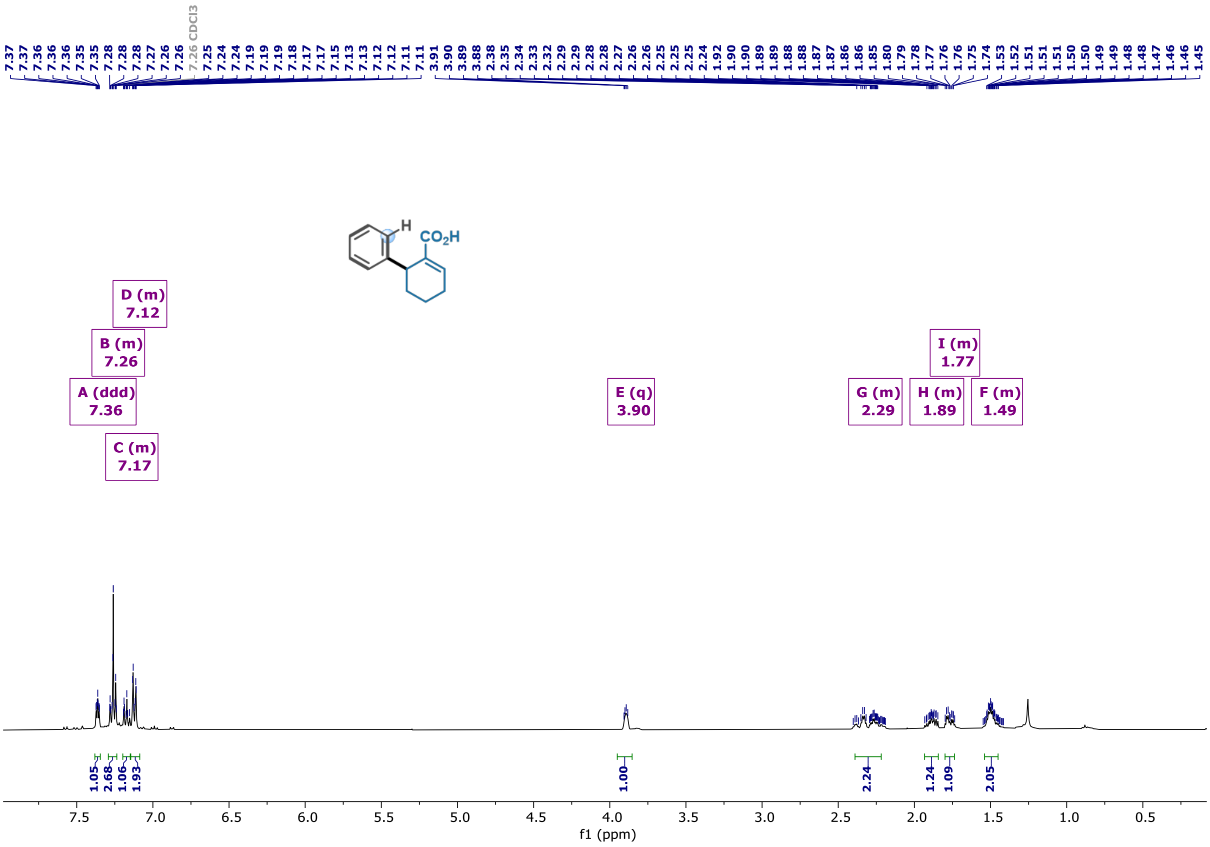

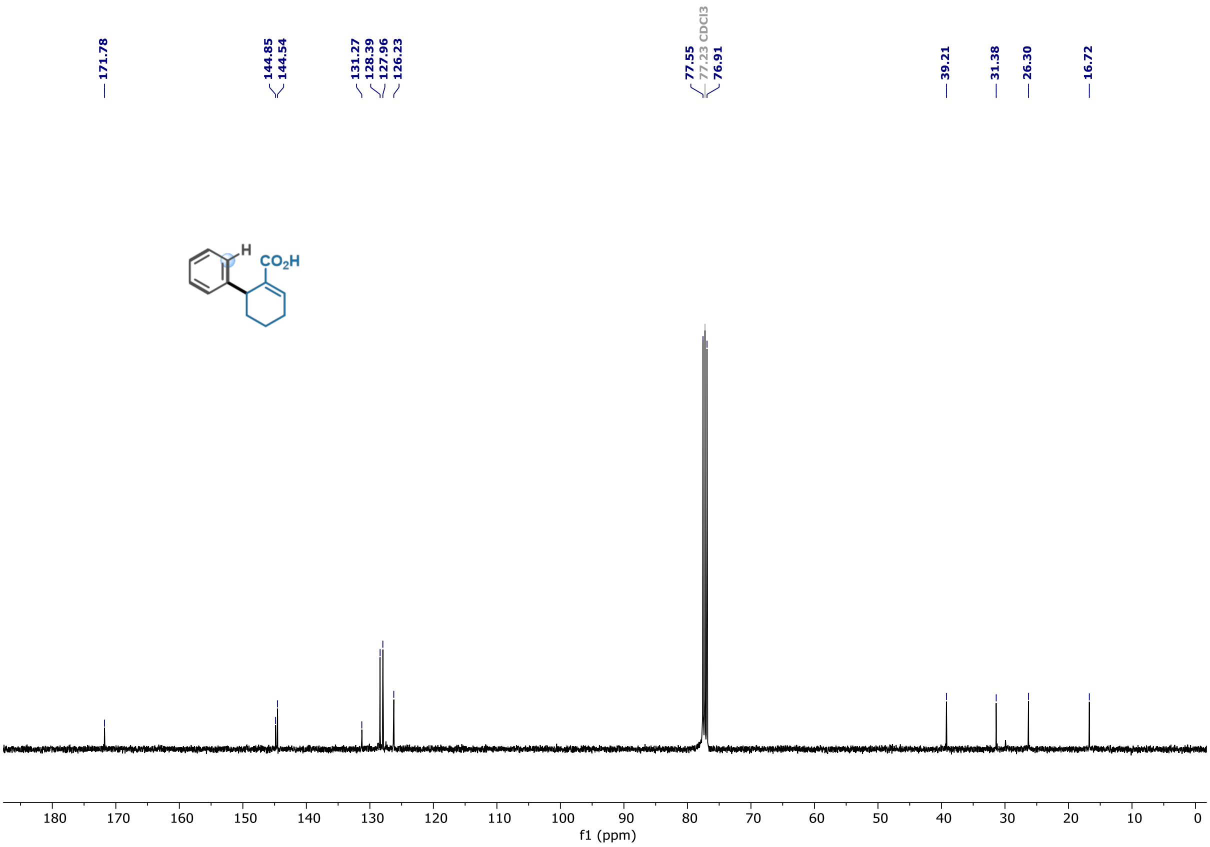

2,3,4,4a-tetrahydro-9H-fluoren-9-one (85)

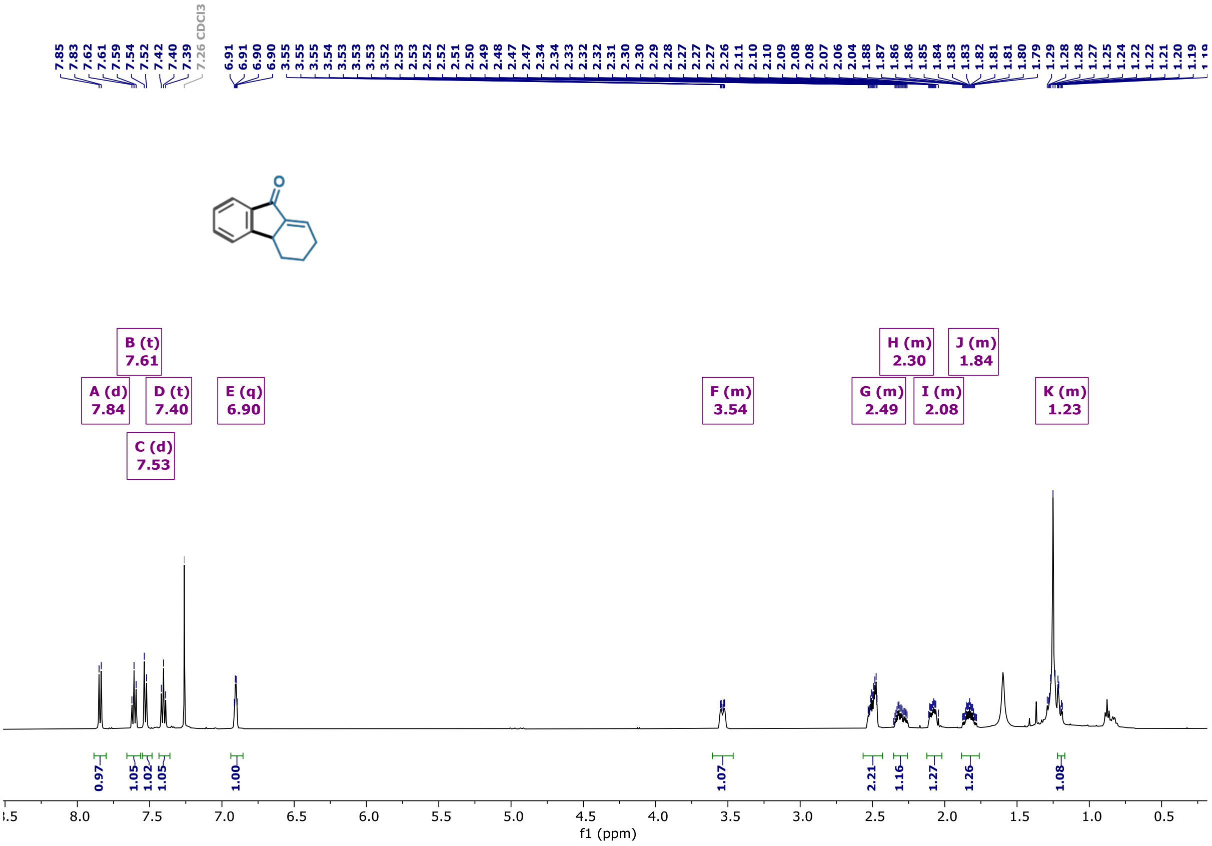

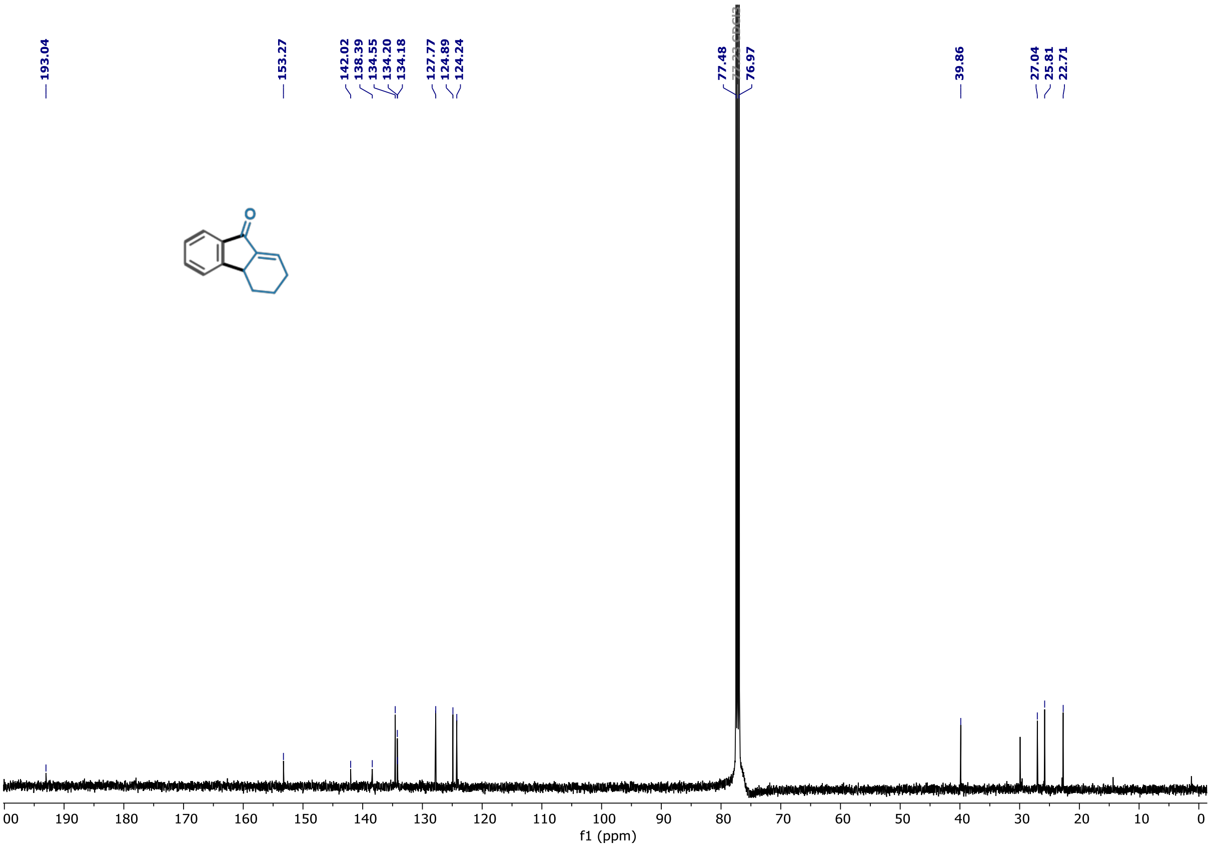

5-phenylcyclopent-1-ene-1-carboxylic acid (86)

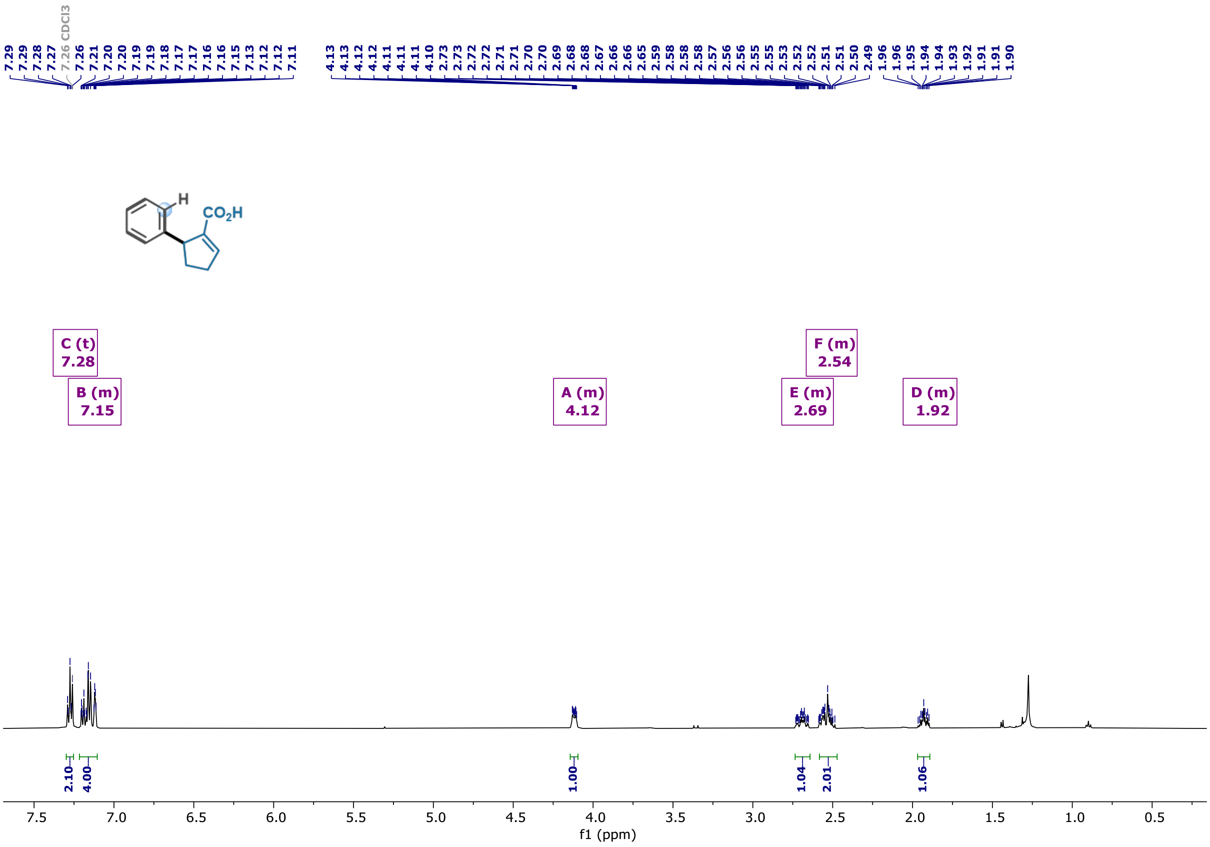

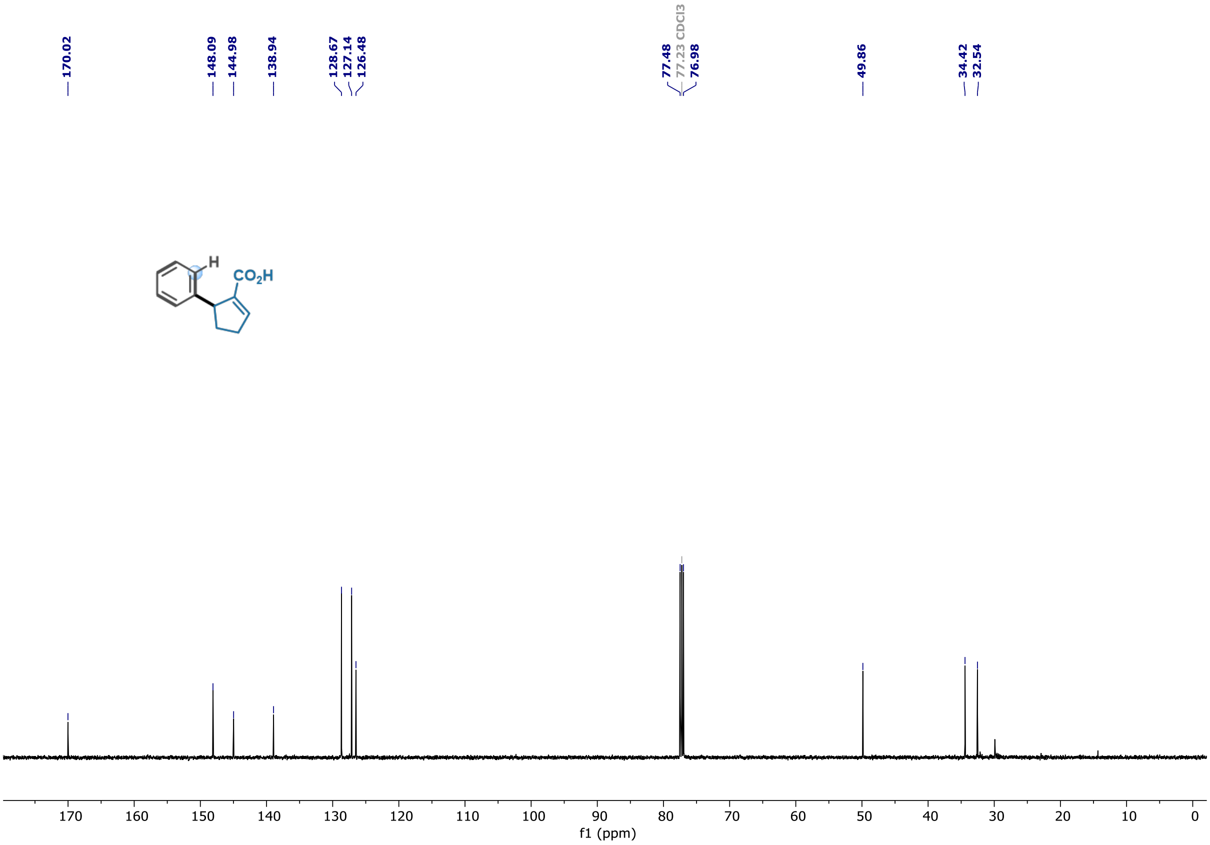

(E)-2-(3-ethoxy-3-oxoprop-1-en-1-yl)-5-phenylcyclopent-1-ene-1-carboxylic acid (87)

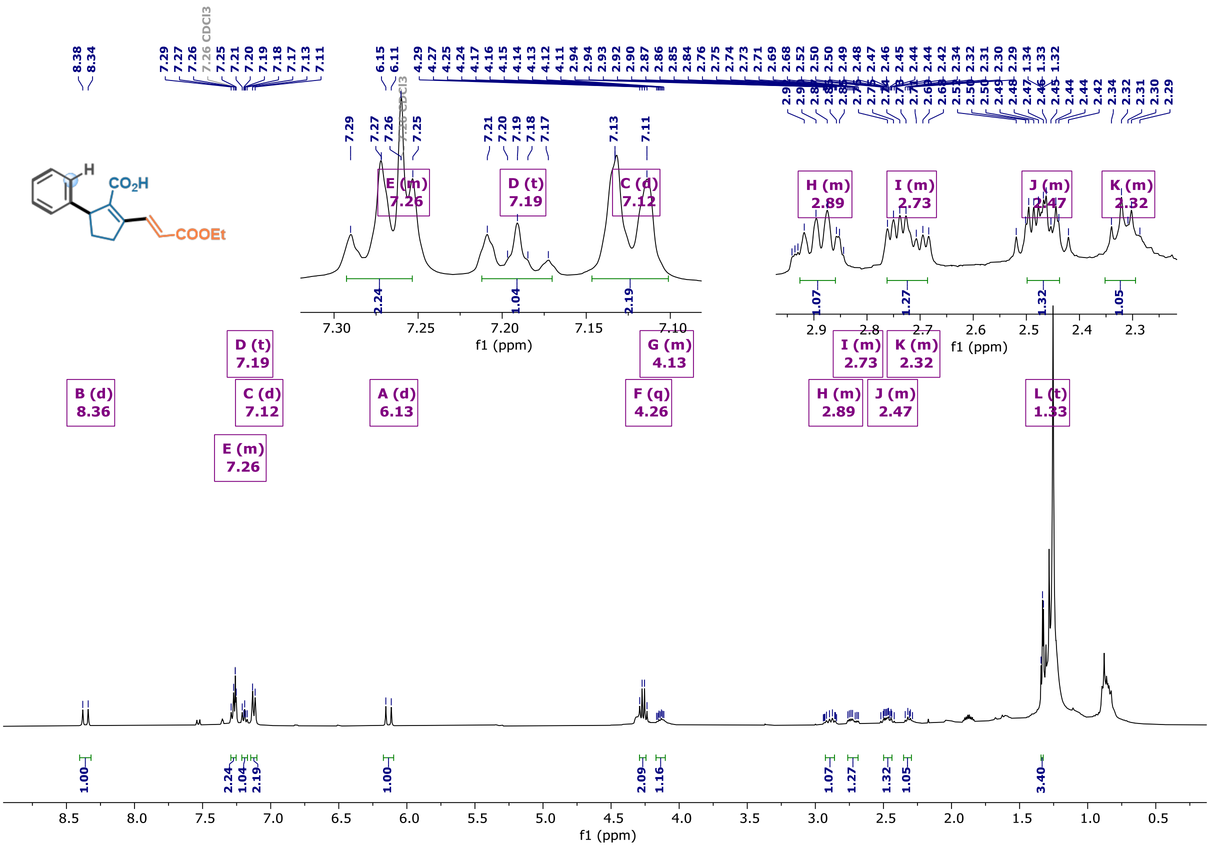

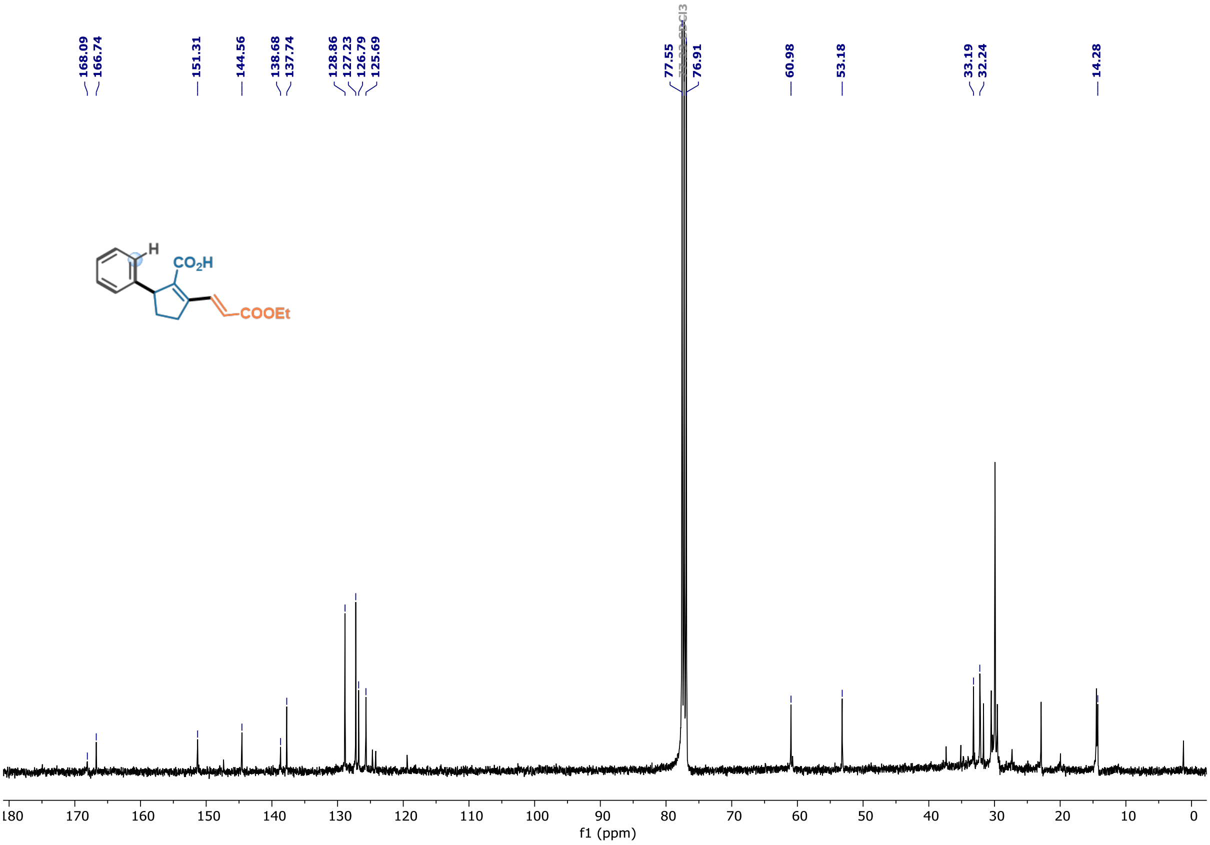

(E)-3-(3-(bromomethyl)phenyl)acrylic acid (88)

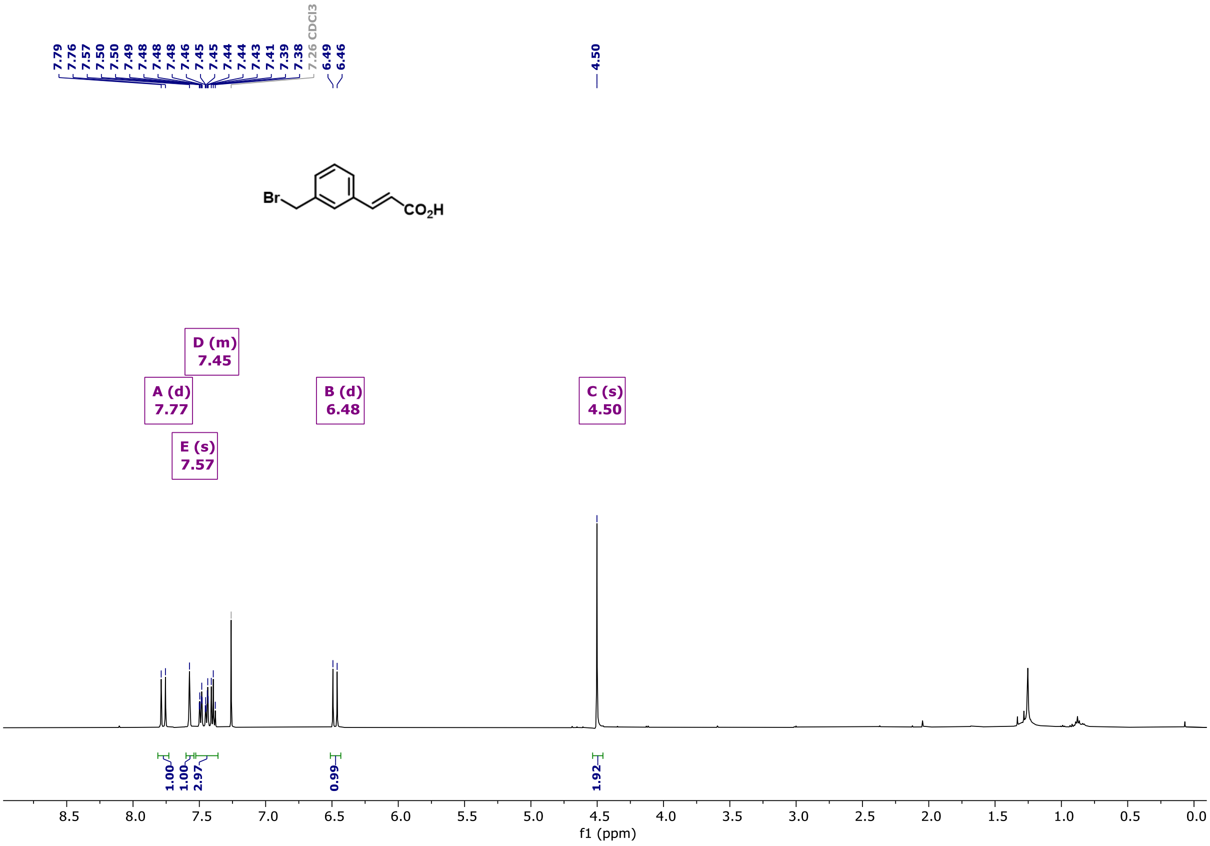

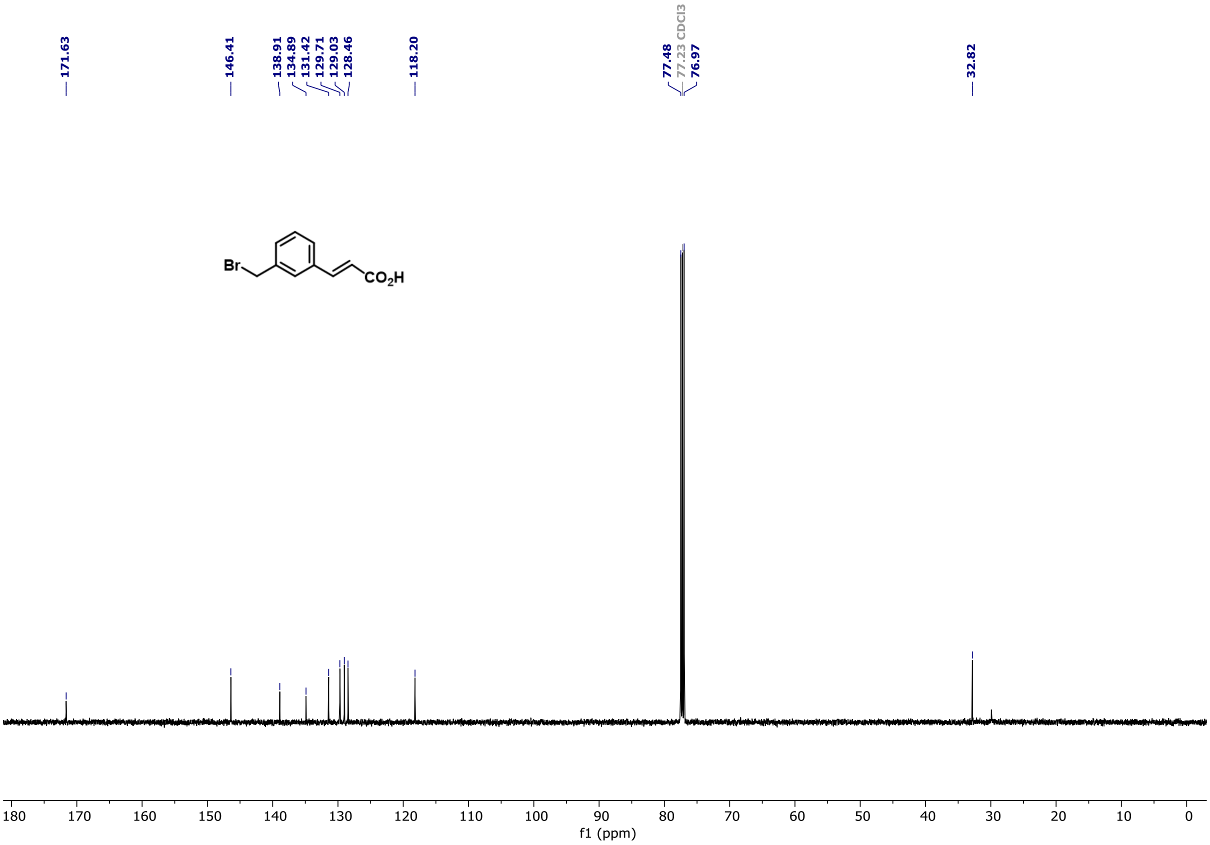

(E)-2-(3-ethoxy-3-oxoprop-1-en-1-yl)benzoic acid

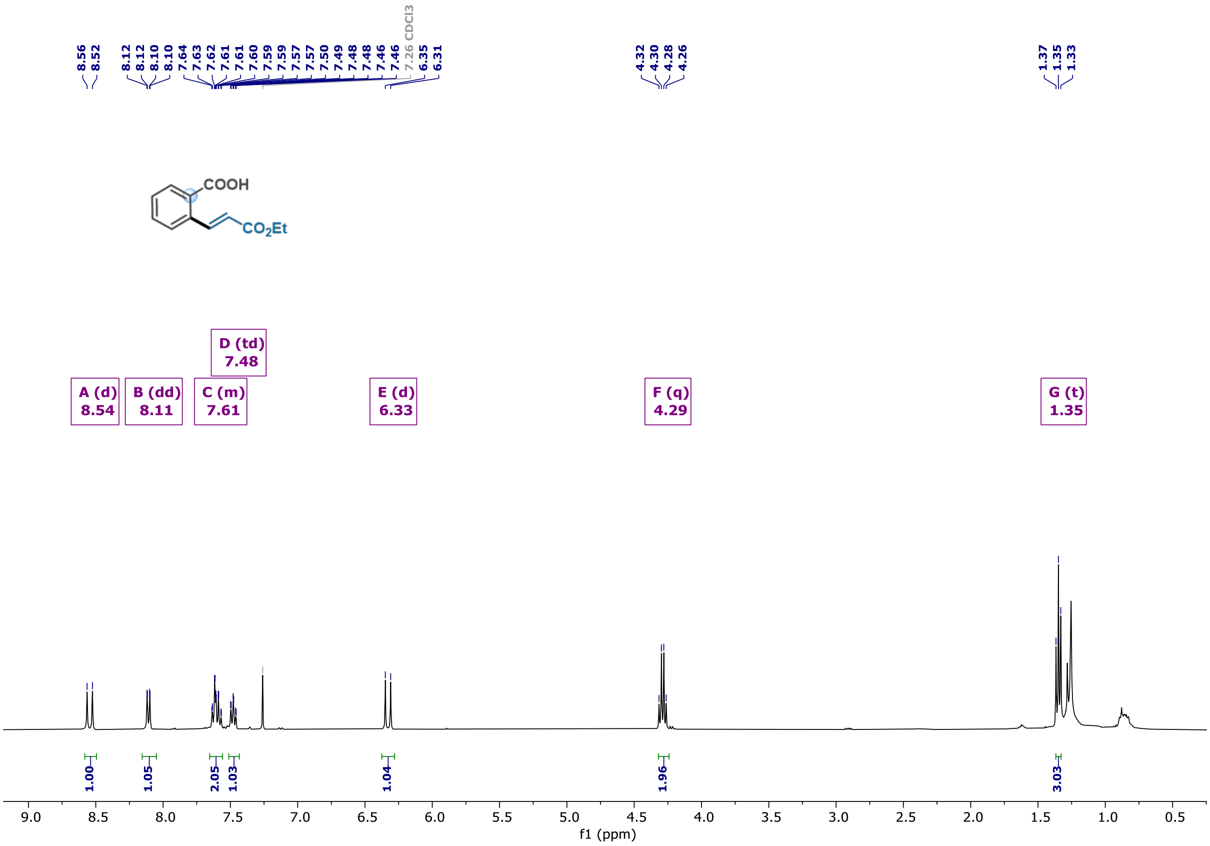

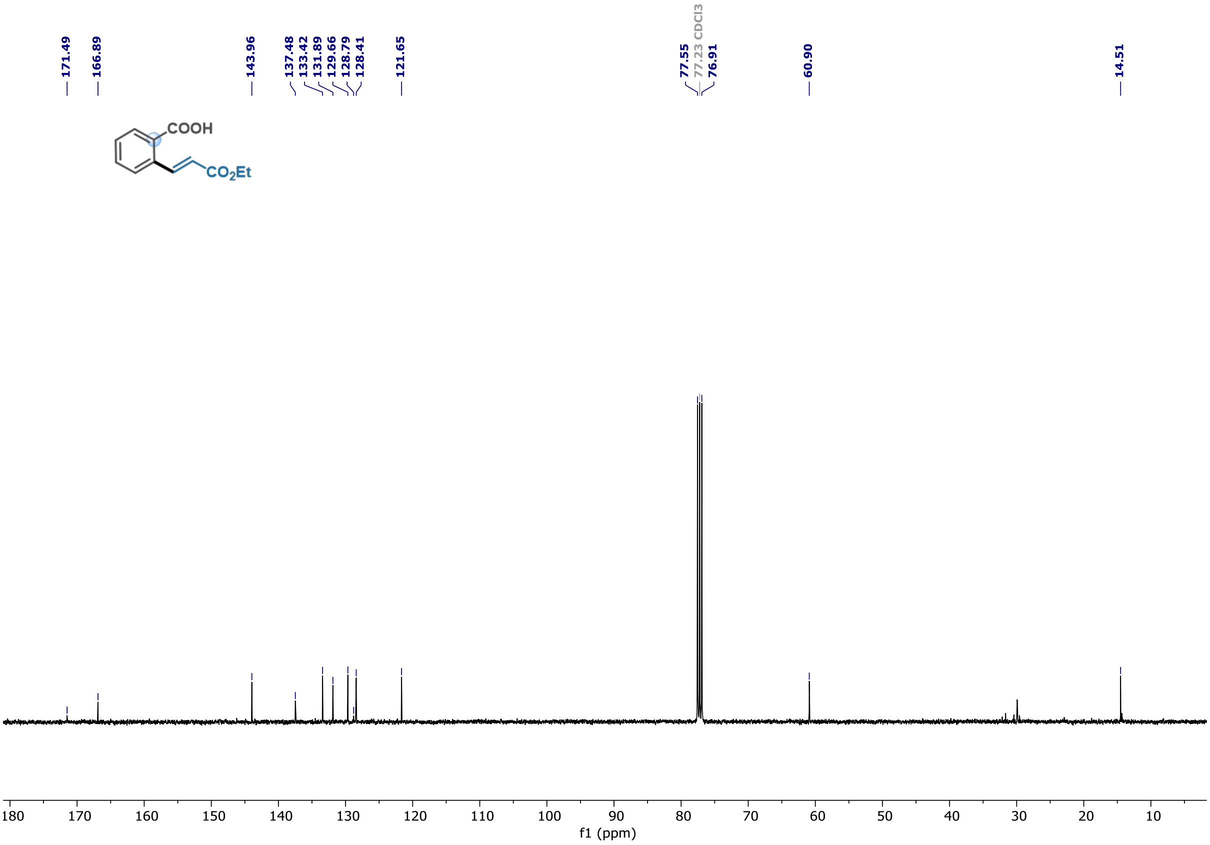

Cyclohex-2-ene-1-carboxylic acid

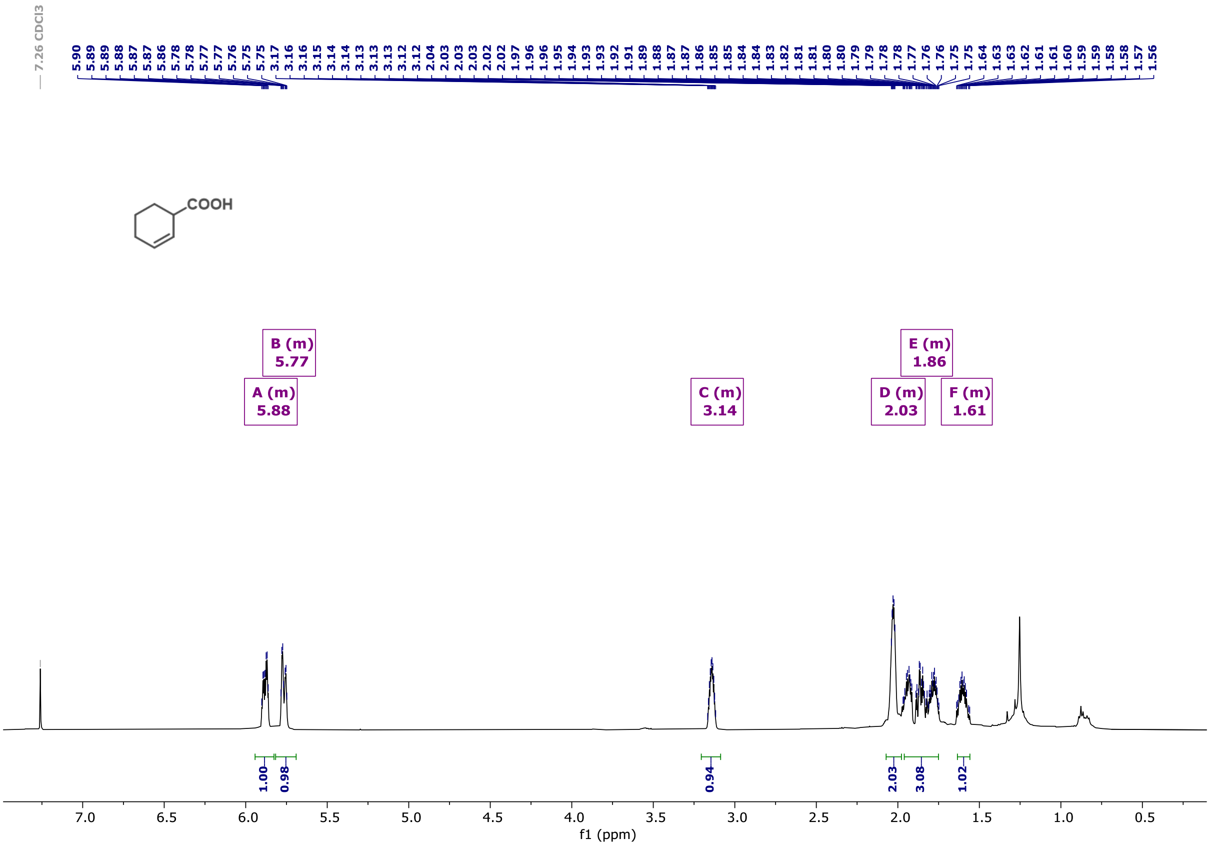

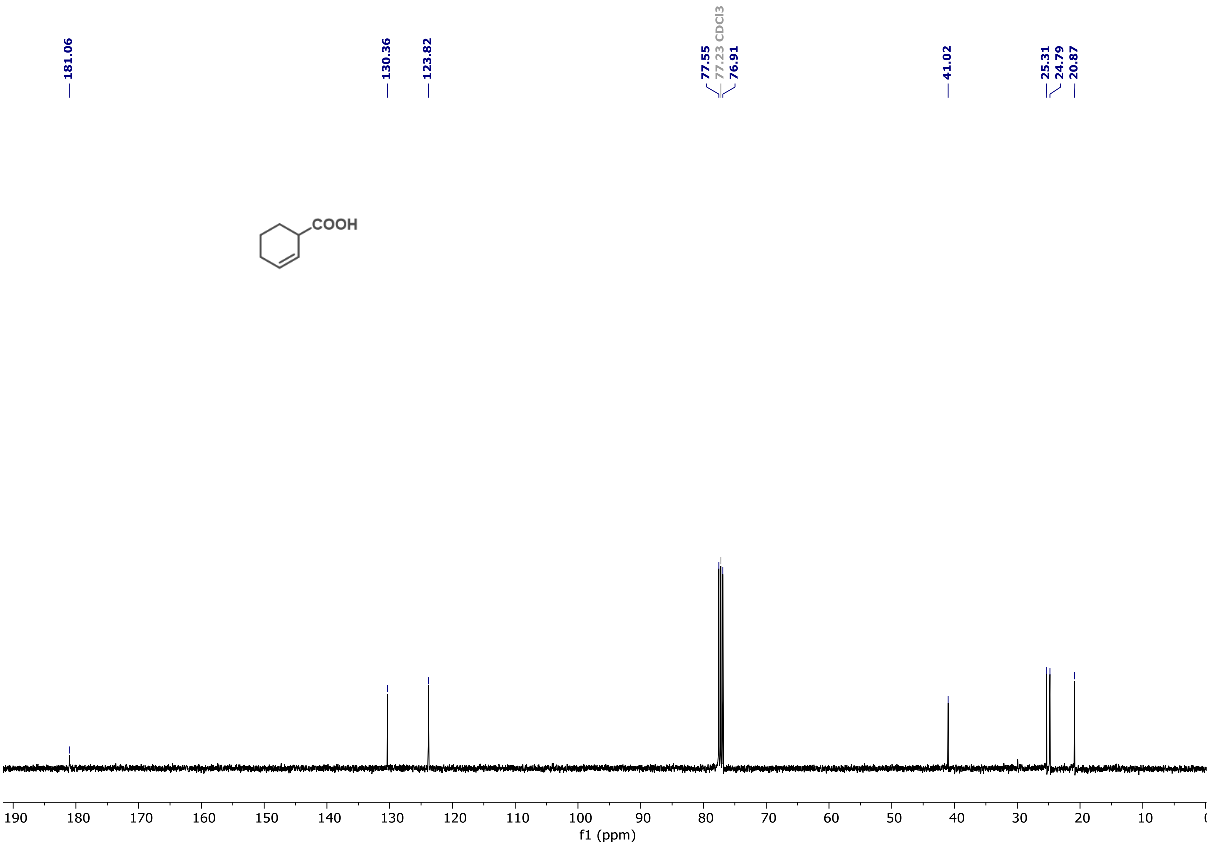

Ethyl (S)-2-(3-oxo-1,3-dihydroisobenzofuran-1-yl)acetate (89)

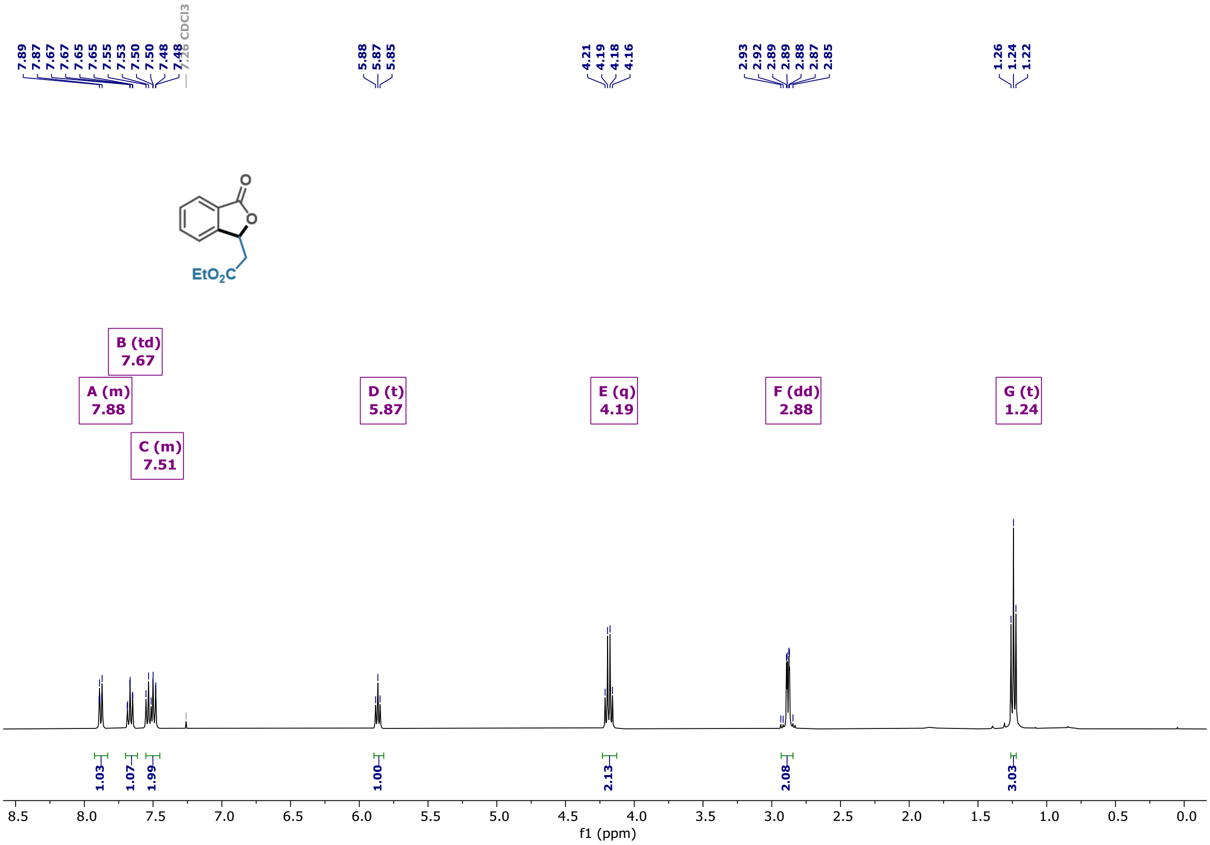

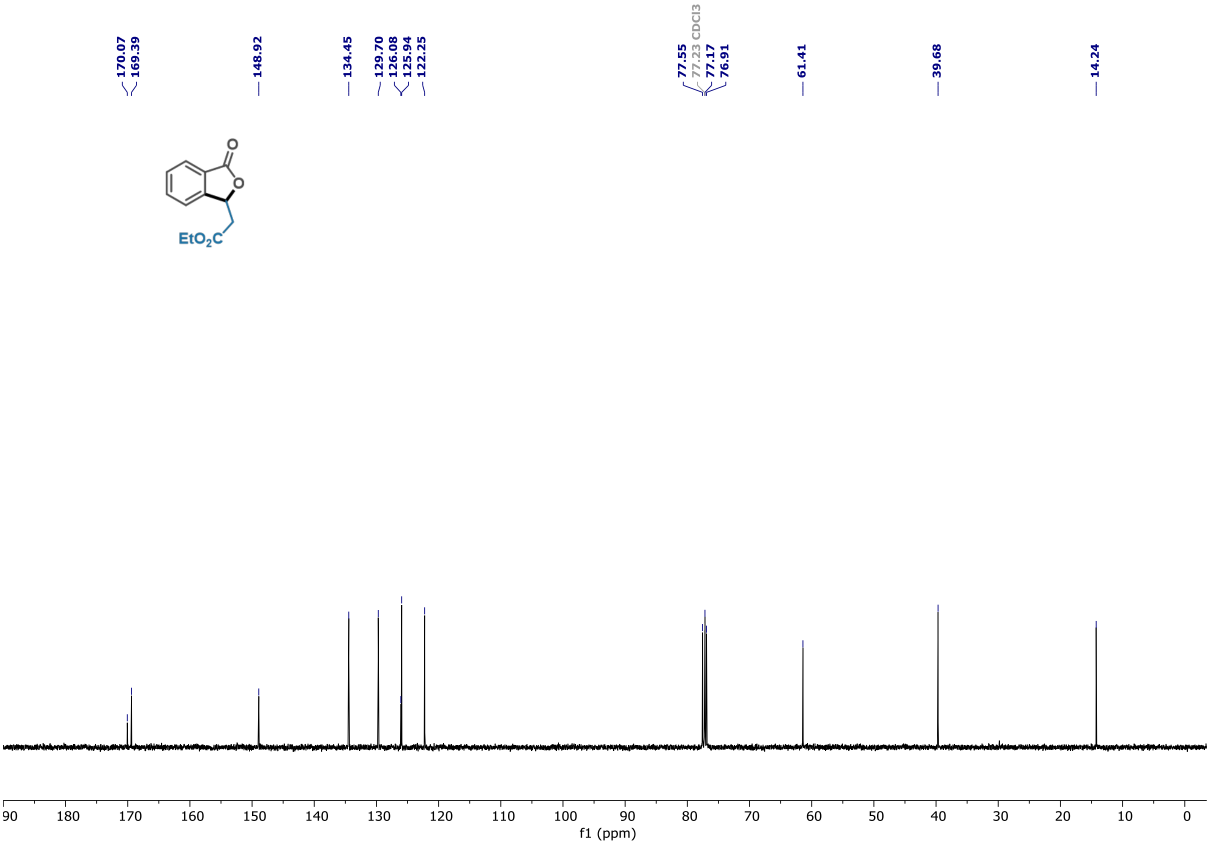

Ethyl (S)-2-(3-oxo-1,3,4,5,6,7-hexahydroisobenzofuran-1-yl)acetate (90)

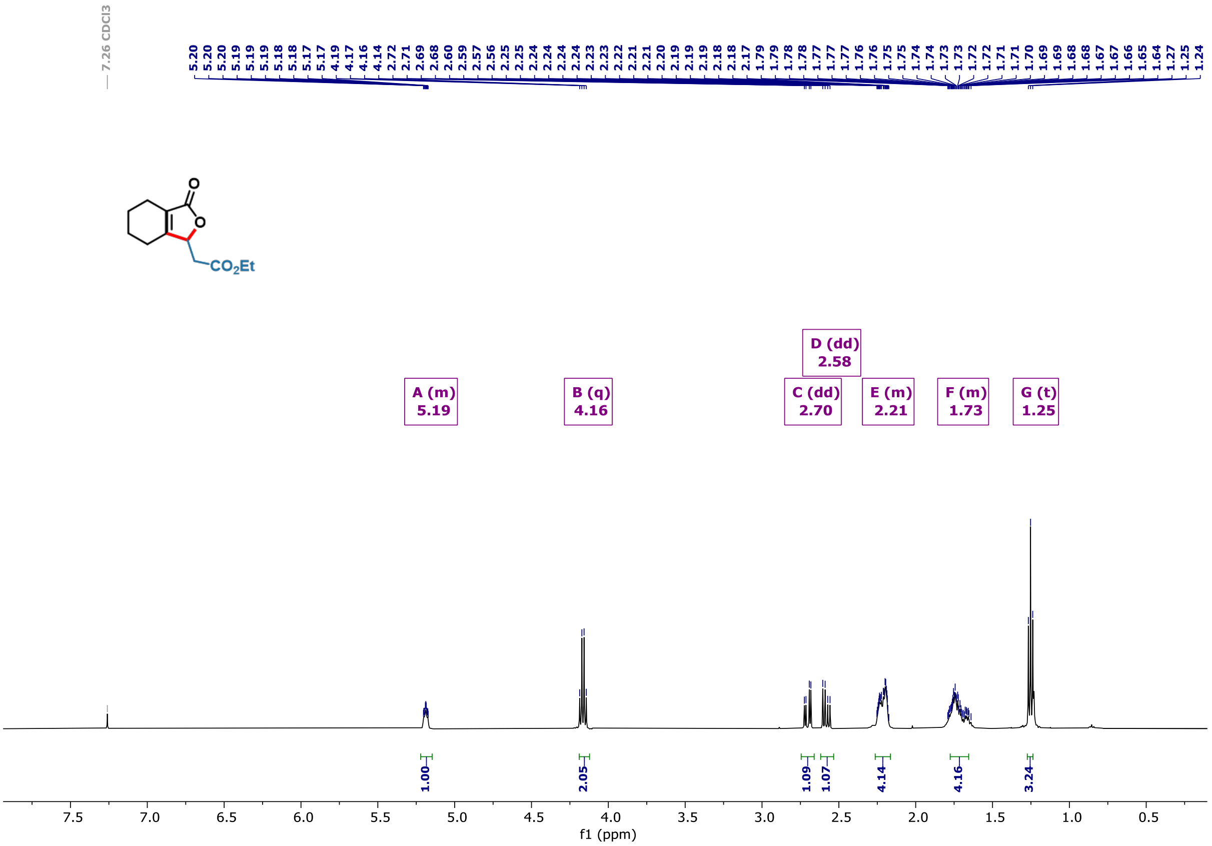

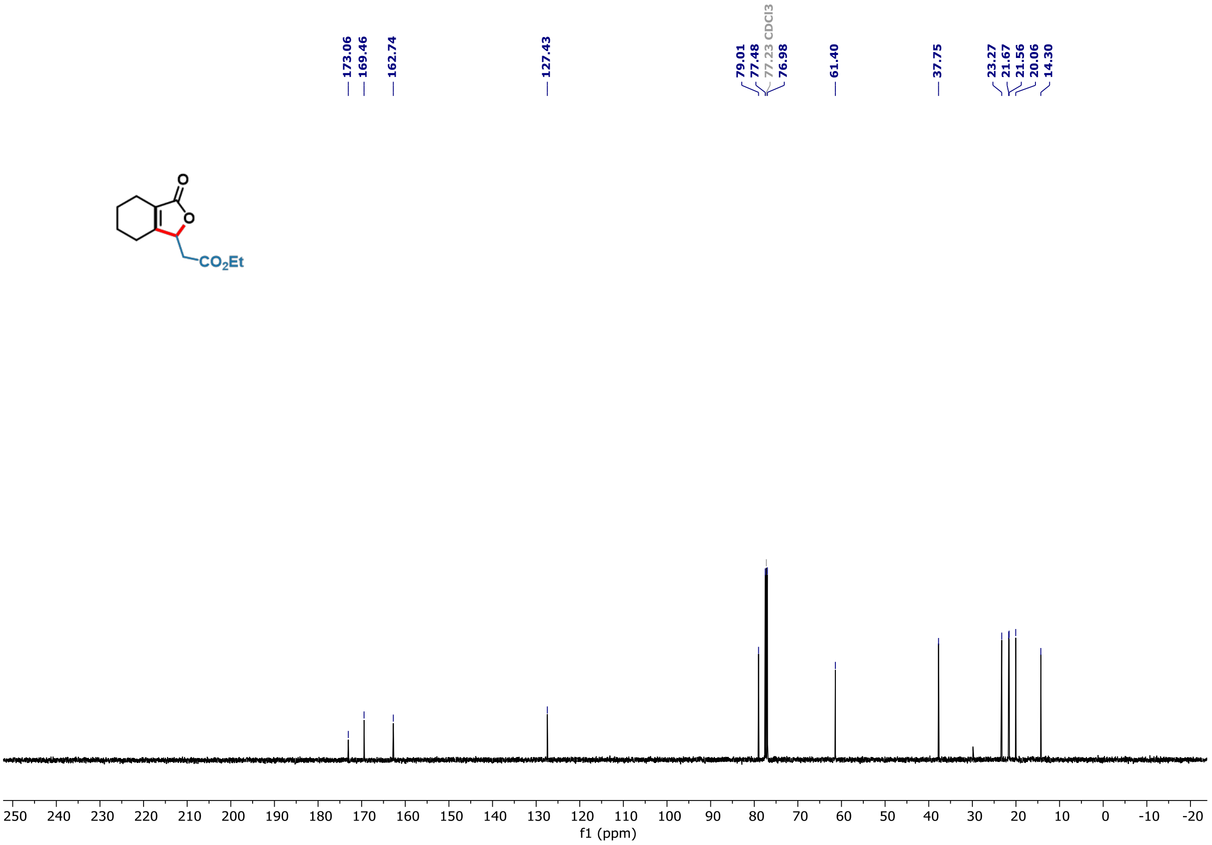

Ethyl (E)-3-(5-(trifluoromethyl)cyclohex-1-en-1-yl)acrylate (91)

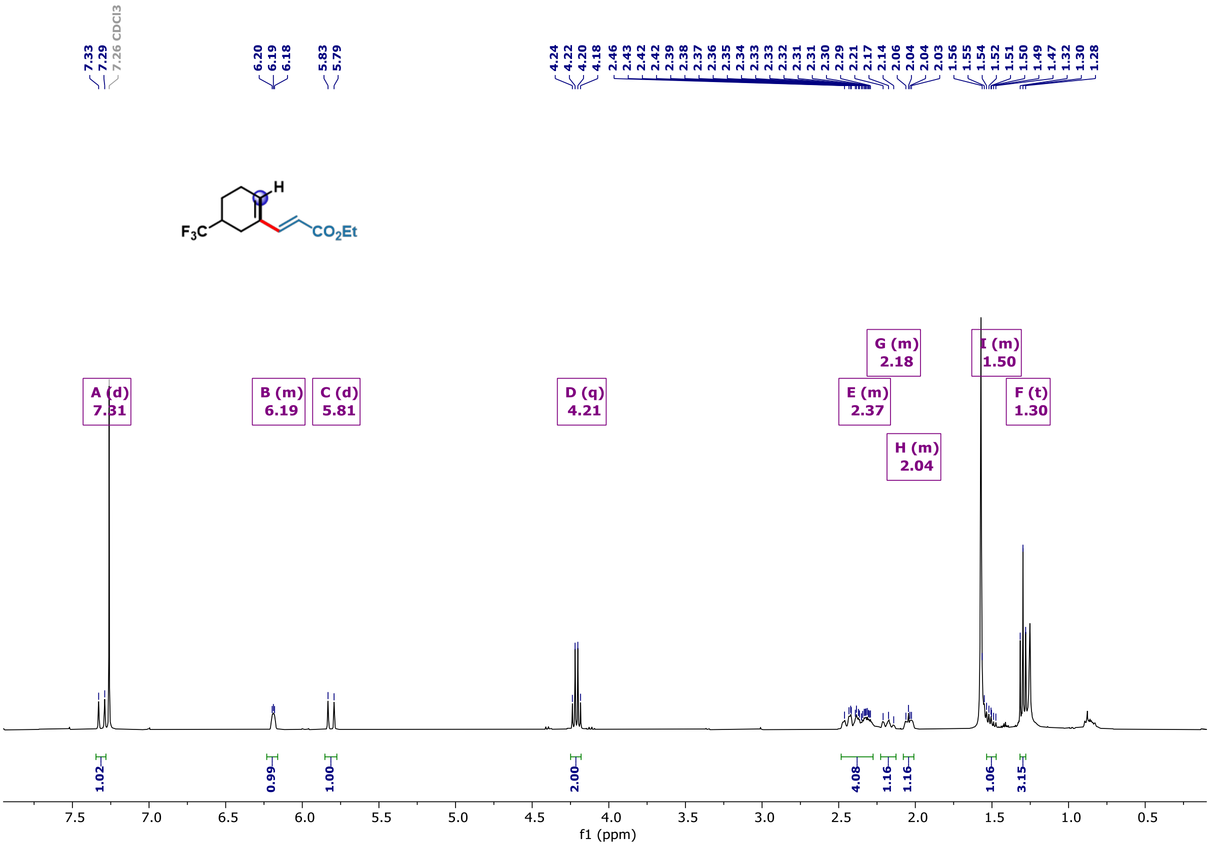

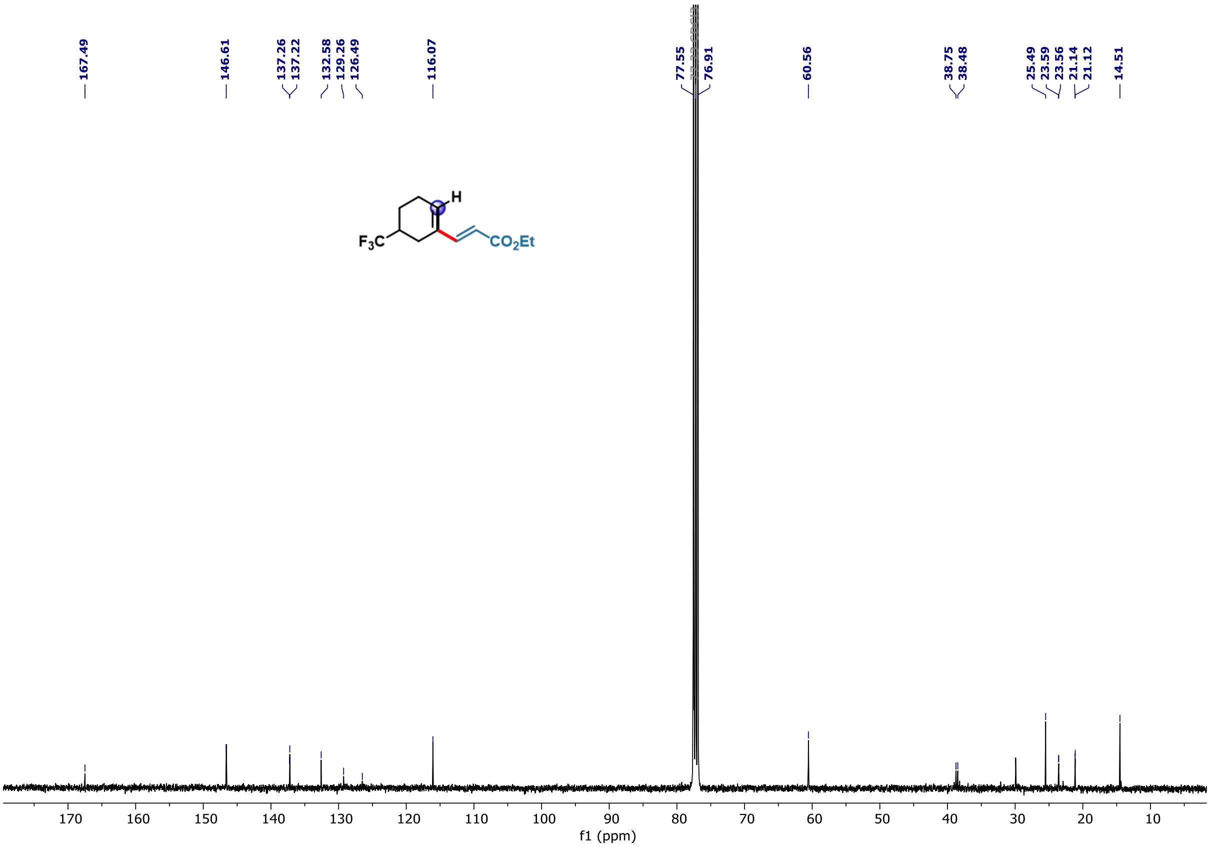

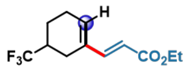
H
F3C
CO2Et

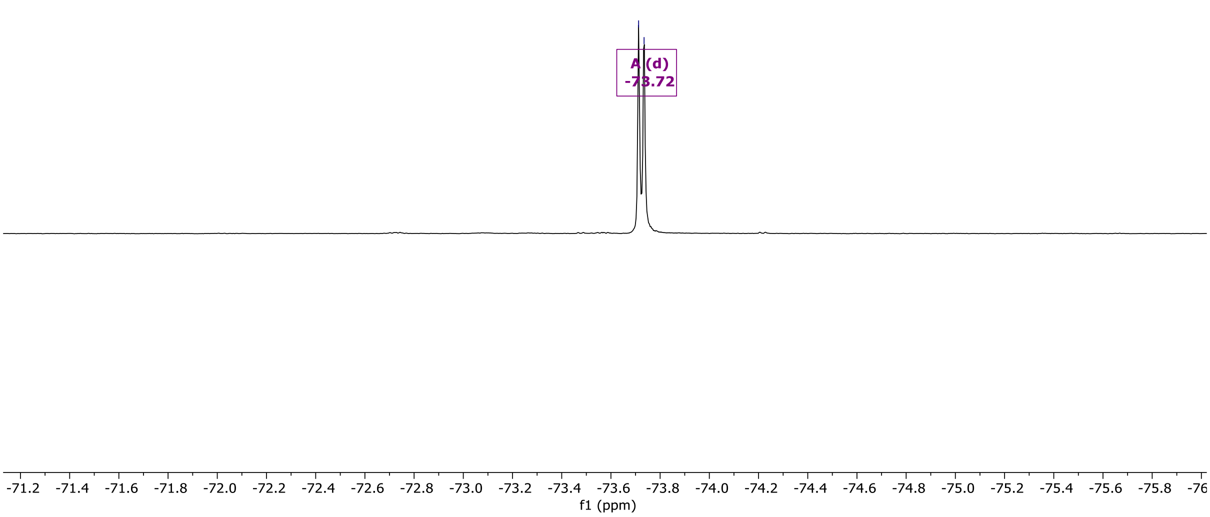
-73.71
-73.73
A (d)
-73.72
-71.2 -71.4 -71.6 -71.8 -72.0 -72.2 -72.4 -72.6 -72.8 -73.0 -73.2 -73.4 -73.6 -73.8 -74.0 -74.2 -74.4 -74.6 -74.8 -75.0 -75.2 -75.4 -75.6 -75.8 -76
f1 (ppm)

Methyl (E)-4-(tert-butyl)-2-(3-oxobut-1-en-1-yl)cyclohex-2-ene-1-carboxylate (92)

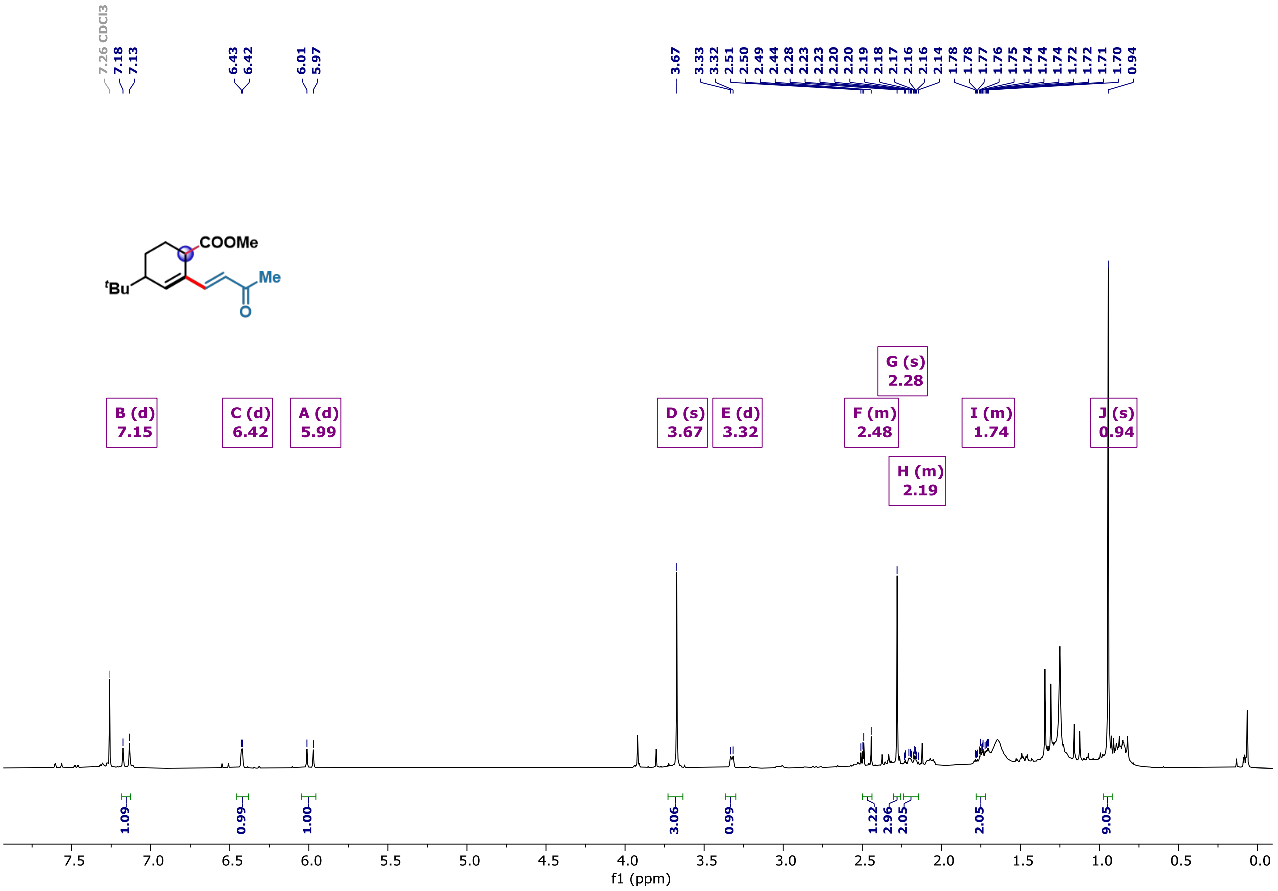

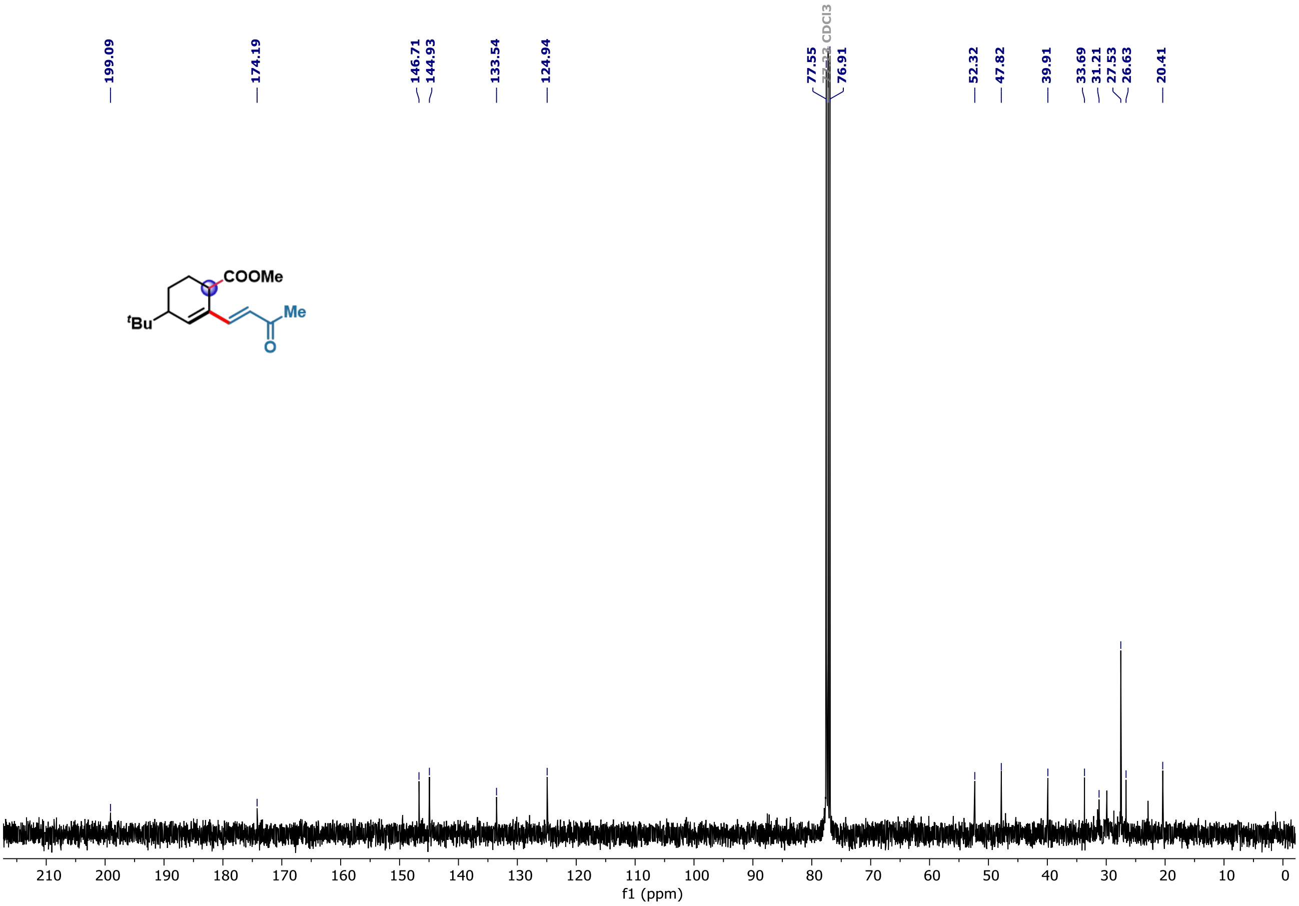

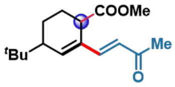
COOMe
Me
O
tBu

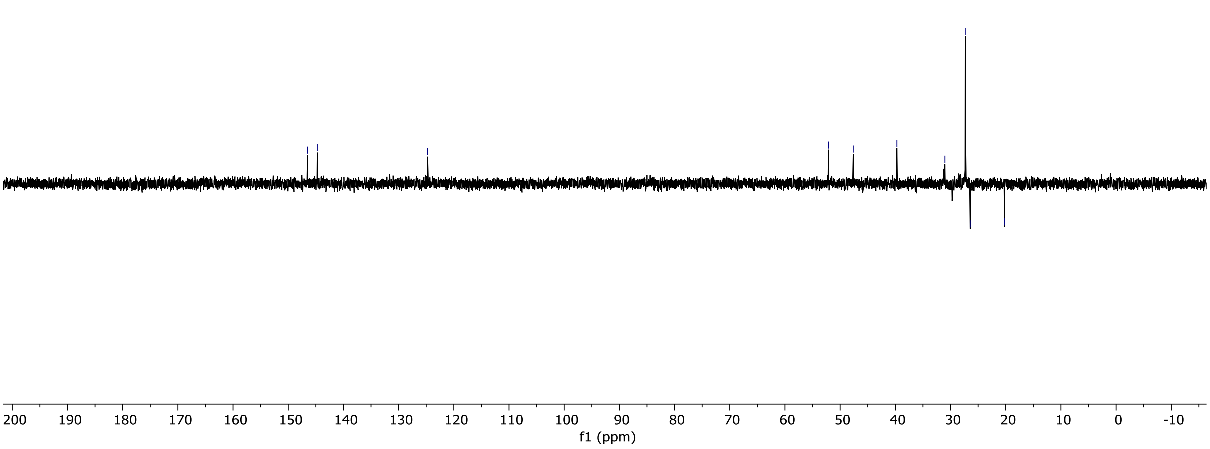
146.51
144.74
124.74
52.12
47.63
39.72
31.02
27.34
26.44
20.21
200 190 180 170 160 150 140 130 120 110 100 90 80 70 60 50 40 30 20 10 0 -10
f1 (ppm)

4-(tert-butyl)cyclohex-1-ene-1-carboxylic acid

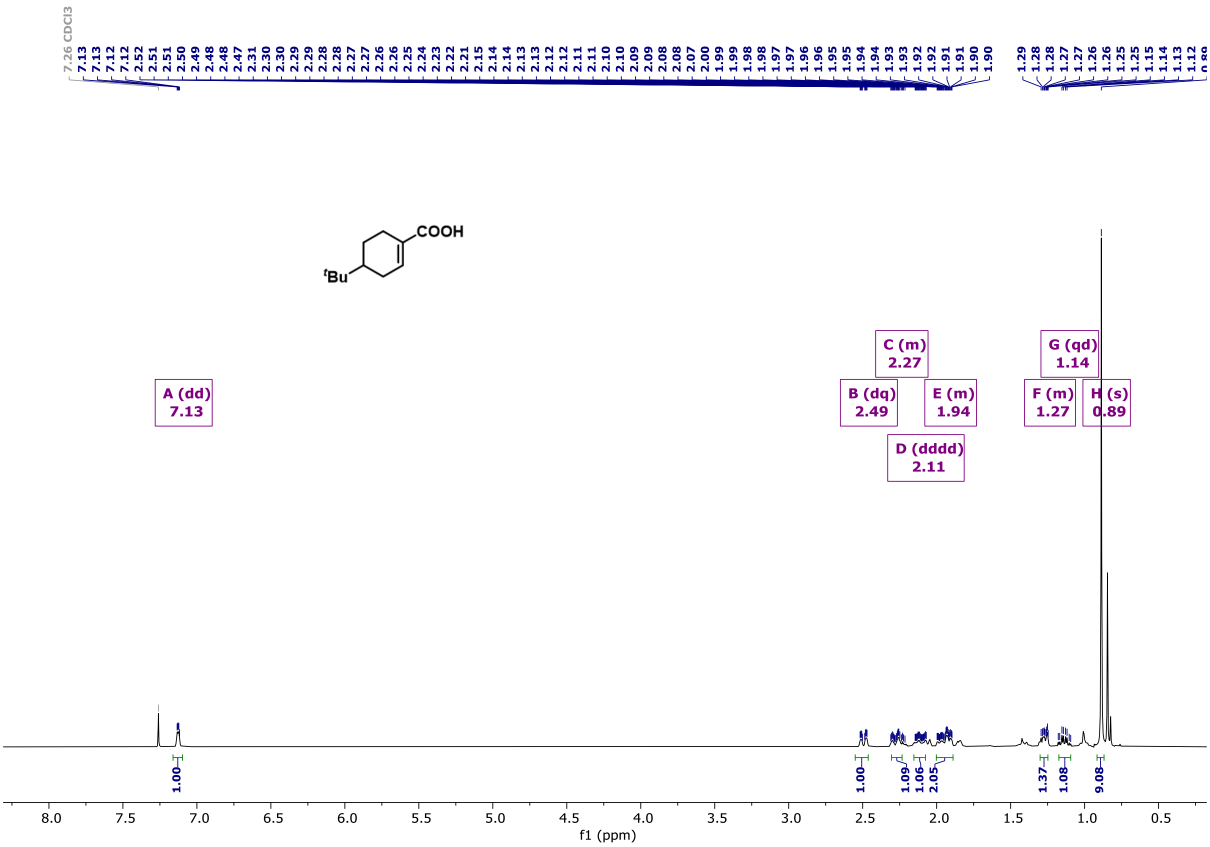

Ethyl-2-(6-(tert-butyl)-3-oxo-1,3,4,5-tetrahydroisobenzofuran-1-yl)acetate (1')

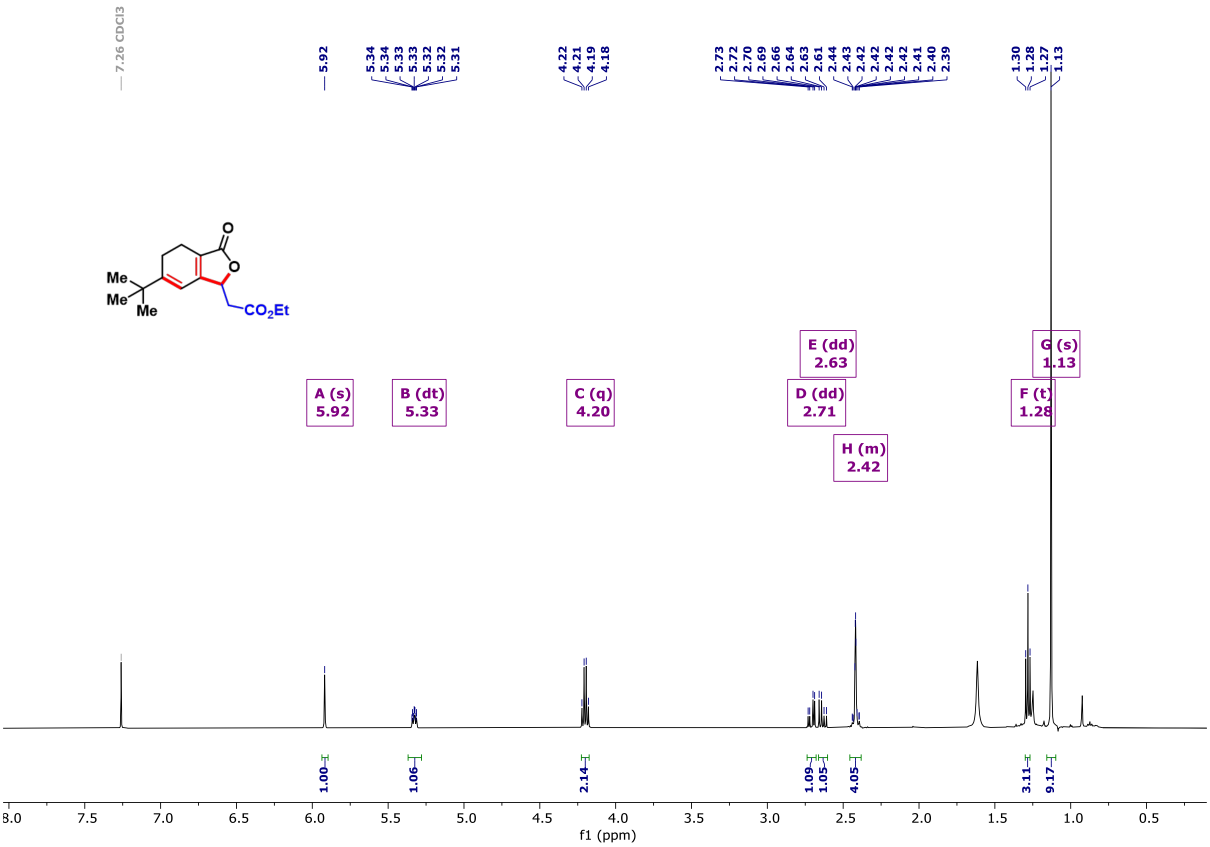

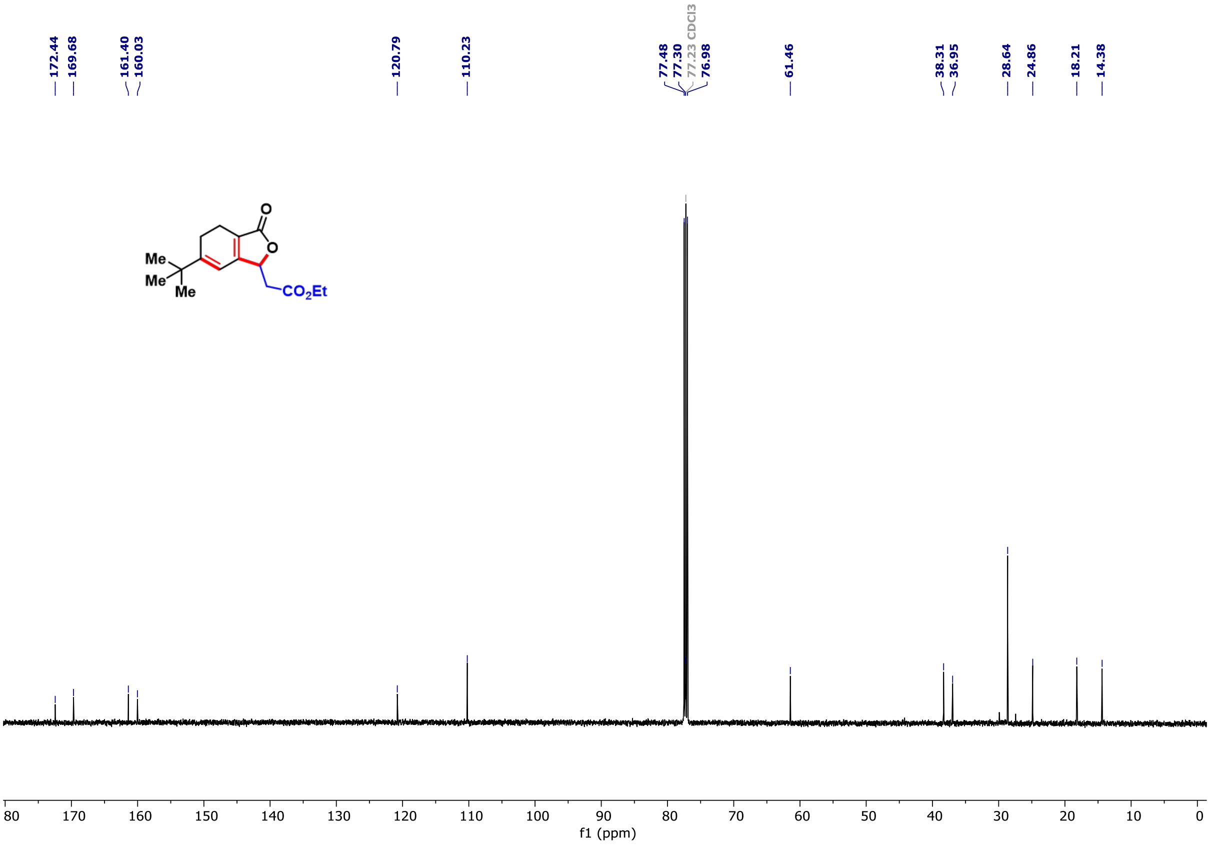

Ethyl (E)-3-(5-methoxycyclohex-1-en-1-yl)acrylate (93)

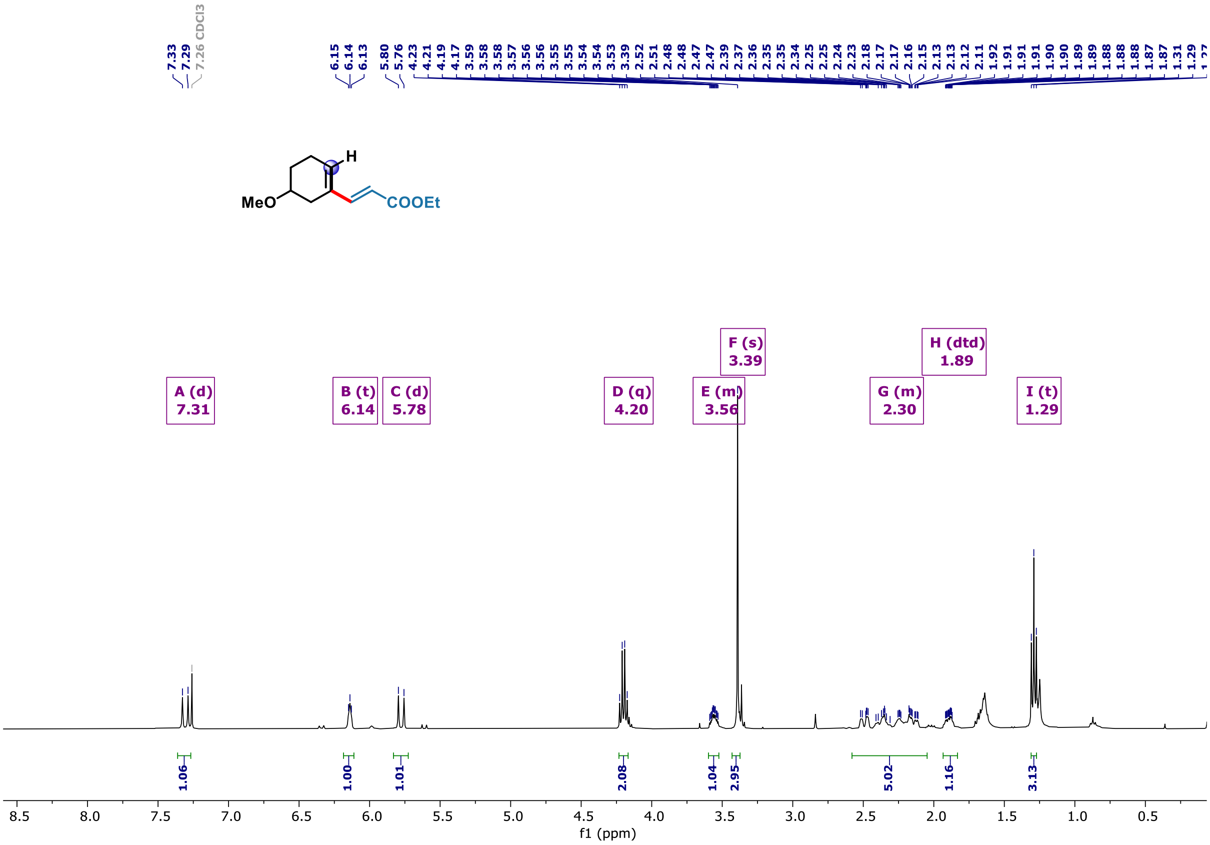

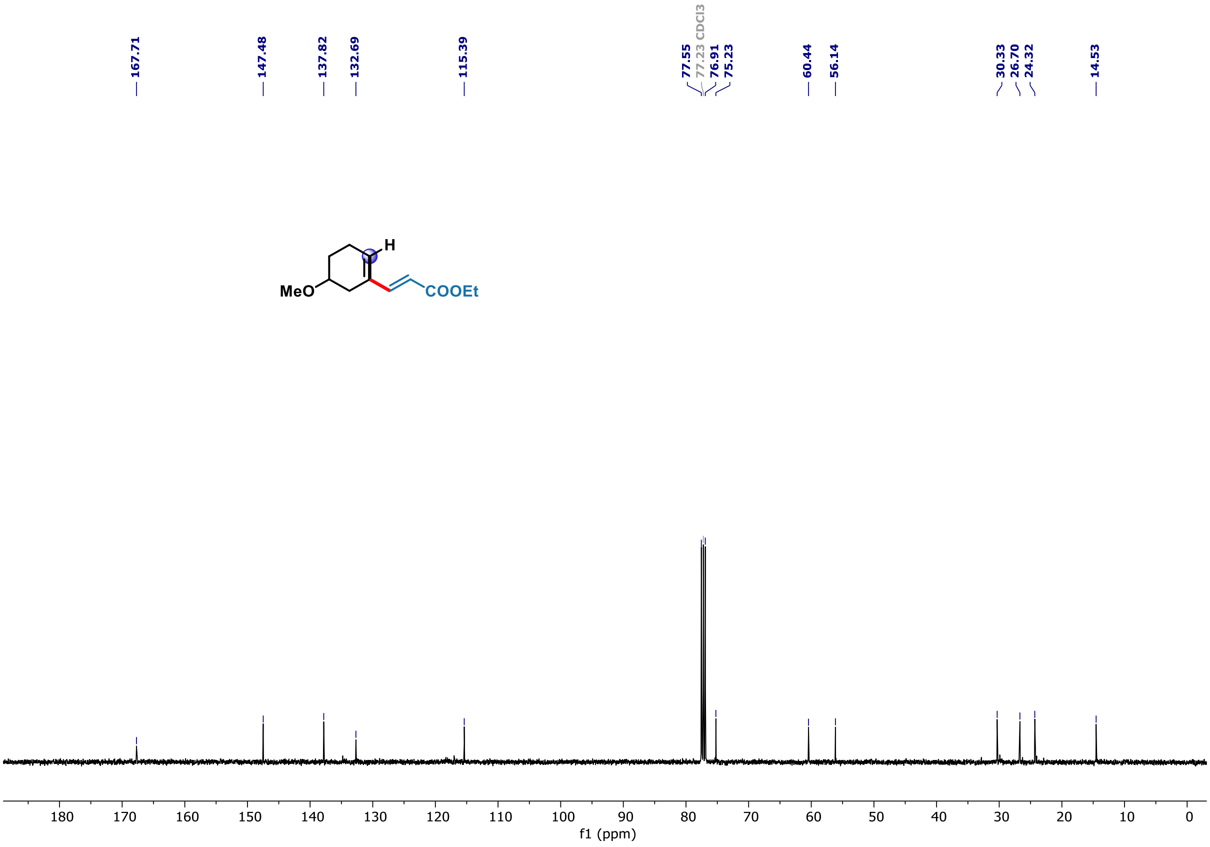

2-oxabicyclo[2.2.1]heptan-3-one (94)

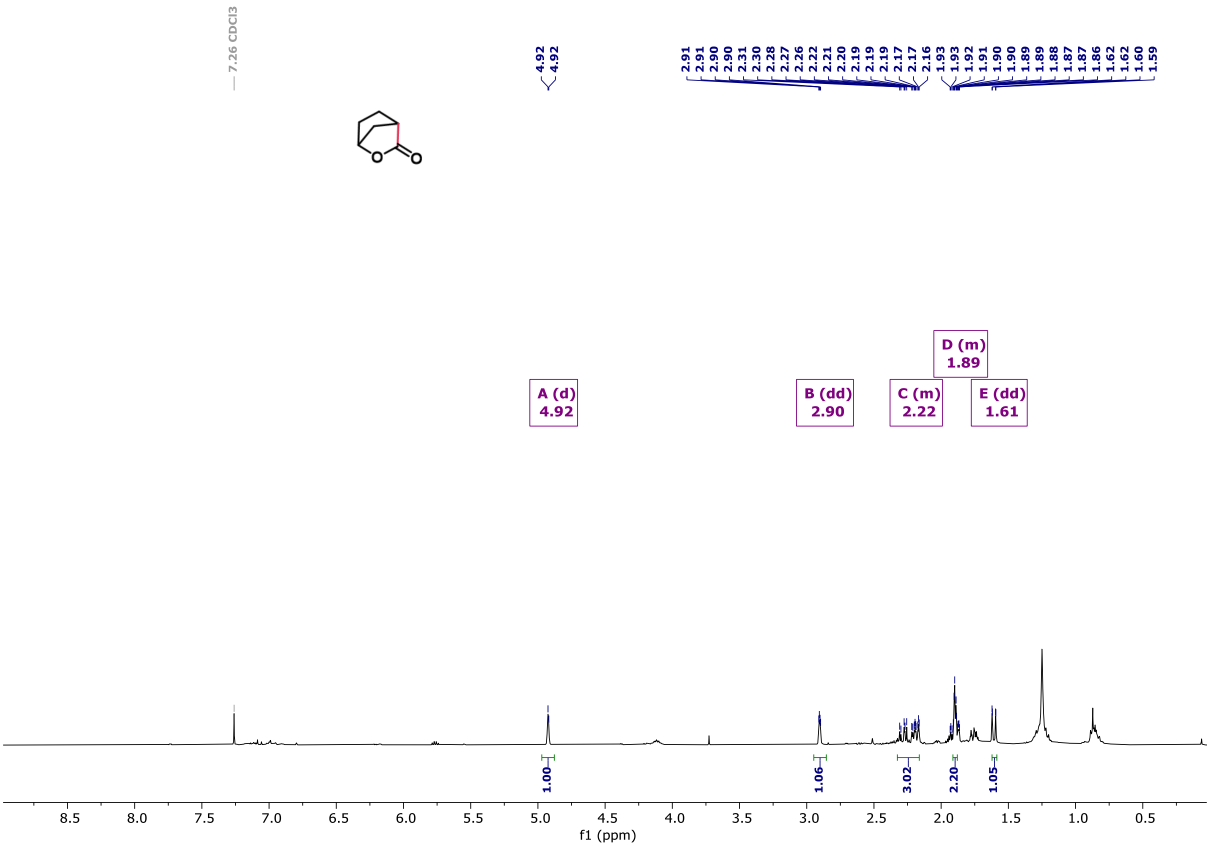

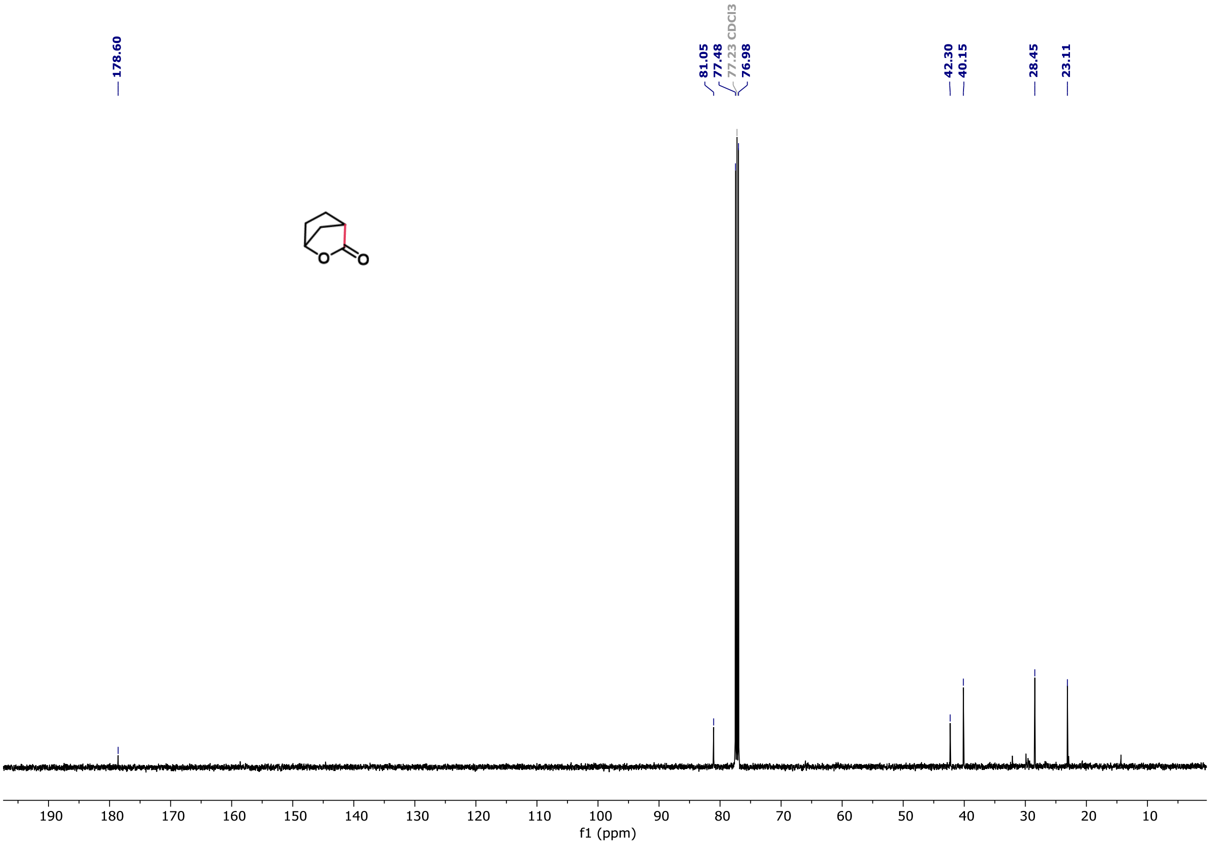

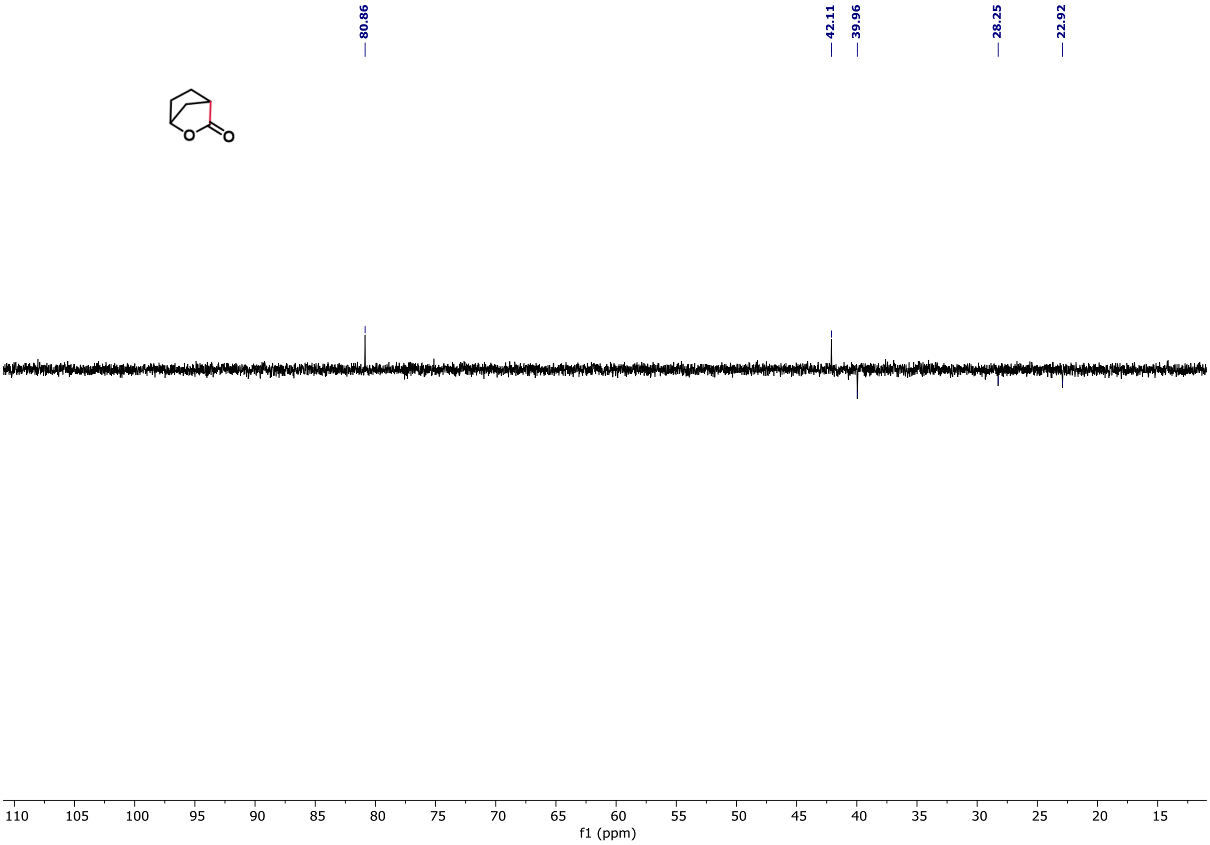
80.86
42.11
39.96
28.25
22.92
110 105 100 95 90 85 80 75 70 65 60 55 50 45 40 35 30 25 20 15
f1 (ppm)

4. Supplementary References:

1. Izawa, Y., Pun, D., Stahl, S. S. Palladium-Catalyzed Aerobic Dehydrogenation of Substituted Cyclohexanones to Phenols. *Science*, **333**, 209-213 (2011).
2. Iosub, A. V., Stahl, S. S. Palladium-Catalyzed Aerobic Dehydrogenation of Cyclic Hydrocarbons for the Synthesis of Substituted Aromatics and Other Unsaturated Products. *ACS. Catal.* **6**, 8201-8213 (2016).
3. Motiwala, H. F., Armaly, A. M., Cacioppo, J. G., Coombs, T. C., Koehn, K. R. K., Norwood IV, V. M., Aubé, J. HFIP in Organic Synthesis. *Chem. Rev.* **122**, 12544–12747 (2022).
4. Greene, T. W., Wuts, P. G. M. Protective Groups in Organic Synthesis, 4th ed.; *Wiley*, pp. 223–238 (2007).
5. Dastbaravardeh, N., Toba, T., Farmer, M. E., Yu, J. -Q. Monoselective o-C−H Functionalizations of Mandelic Acid and α-Phenylglycine. *J. Am. Chem. Soc.* **137**, 9877−9884 (2015).
6. Qi, Q. -R., Pan, J., Guo, X. -Q.; Weng, L. -L., Liang, Y. -F. Synthesis and antibacterial activity of new fluoroquinolones containing a cis- or trans-cyclohexane moiety. *Bioorganic Med. Chem. Lett*. **22**, 7688-7692 (2012).
7. Cao, P., Li, C. -Y., Kang, Y. -B., Xie, Z., Sun, X. -L., Tang. Y. Ph_3As-Catalyzed Wittig-Type Olefination of Aldehydes with Diazoacetate in the Presence of $Na_2S_2O_4$. *J. Org. Chem.* **72**, 6628-6630 (2007).
8. Adolfsson, D. E., Tyagi, M., Singh, P., Deuschmann, A., Ådén, J., Gharibyan, A. L., Jayaweera, S. W., Lindgren, A. E. G., Olofsson, A., Almqvist, F. Intramolecular Povarov Reactions for the Synthesis of Chromenopyridine Fused 2-Pyridone Polyheterocycles Binding to *α*–Synuclein and Amyloid-*β* Fibrils. *J. Org. Chem*. **85**, 14174−14189 (2020).
9. Wang, P., Liu, C. -R., Sun, X. -L., Chen, S, -S., Li, J. -F., Xie, Z., Tang, Y. A newly-designed PE-supported arsine for efficient and practical catalytic Wittig olefination. *Chem. Commun.*, **48**, 290-292 (2012).
10. Sheng, T., Zhuang, Z., Wang, Z., Hu, L., Herron, A. N., Qiao, J. X., Yu, J. -Q. One-Step Synthesis of *β*-Alkylidene-*γ*-lactones via Ligand-Enabled *β,γ*-Dehydrogenation of Aliphatic Acids. *J. Am. Chem. Soc*. **144**, 12924-12933 (2022).
11. Yang, T., Lu, M., Lin, Z., Huang, M., Cai. S. Visible-light-promoted oxidation/condensation of benzyl alcohols with dialkylacetamides to cinnamides. *Org. Biomol. Chem.* **17**, 449-453 (2019).

12. Onneken, C., Bussmann, K., Gilmour, R. Inverting External Asymmetric Induction *via* Selective Energy Transfer Catalysis: A Strategy to *β*-Chiral Phosphonate Antipodes. *Angew. Chem. Int. Ed.* **59**, 330-334 (2020).
13. Jiang, Q., Xu, B., Jia, J., Zhao, A., Zhao, Y. -R., Li, Y. -Y., He, N. -N., Guo. C. -C. Copper-Catalyzed Aerobic Decarboxylative Sulfonylation of Cinnamic Acids with Sodium Sulfinates: Stereospecific Synthesis of (E)-Alkenyl Sulfones. *J. Org. Chem.* **79**, 7372-7379 (2014).
14. Li, Z., Twieg, R. J. Photocyclodehydrofluorination. *Chem. - Eur. J.* **21**, 15534− 15539 (2015).
15. Wang, S., Hu, D., Hua, W., Gu, J., Zhang, Q., Jia, X., Xi, K. Palladium salt and functional reduced graphene oxide complex: in situ preparation of a generally applicable catalyst for C–C coupling reactions *RSC Adv*. **5**, 53935-53939 (2015).
16. Zhang, K., Provot, O., Alami, M., Tran, C., Hamze, A. Pd-Catalyzed Coupling of N-Tosylhydrazones with Benzylic Phosphates: Toward the Synthesis of Di- or Tri-Substituted Alkenes. *J. Org. Chem.* **87**, 1249-1261 (2022).
17. Amini, M., Bagherzadeh, M., Moradi-Shoeili, Z., Boghaei, D. M. Pd(OAc)$_2$ without added ligand as an active catalyst for Mizoroki–Heck reaction in aqueous media. *RSC Adv.*, **2**, 12091-12095 (2012).
18. Liu, J., Zhu, X. R., Ren, J., Chen, W, -D., Zeng, B. -B. Copper-Catalyzed One-Pot Oxidation–Aldol/Henry Reaction of Benzylic -Amines to *α,β*-Unsaturated Methyl Ketone/Nitro Compounds. *Synlett*. **24**, 2740-2742 (2013).
19. Zhu, Y. -Q., Han, T. -F., He, J. -L., Li, M., Li, J. -X., Zhu, K. Route to Substituted Furan-2(5H)-ones from cyclo-Alkenecarboxylic Acids and Acrylates *via* C–H Activation. *J. Org. Chem*. **82**, 8598-8603 (2017).
20. Wu, N., Messinis, A., Batsanov, A. S., Yang, Z., Whiting, A., Marder, T. B. Palladium(II)-catalysed tandem cyclisation of electron-deficient aromatic enynes. *Chem. Commun*. **48**, 9986-9988 (2012).
21. Vellalath, S., Romo, D. Telescoped Synthesis of *γ*-Bromo-*β*-Lactones from Allylic Bromides Employing Carbon Dioxide. *Isr. J. Chem.*, **57**, 335-339 (2017).
22. Wang, Z., Hu, L., Chekshin, N., Zhuang, Z., Qian, S., Qiao, J. X., Yu, J. -Q. Ligand-controlled divergent dehydrogenative reactions of carboxylic acids via C–H activation. *Science*. **374**, 1281-1285 (2021).

23. Sheng, T., Kang, G., Zhuang, Z., Chekshin, N., Wang, Z., Hu, L., Yu, J. -Q. Synthesis of *β, γ*-Unsaturated Aliphatic Acids via Ligand-Enabled Dehydrogenation. *J. Am. Chem. Soc.* **145**, 20951–20958 (2023).
